# Supplementary material for: Annotation-free delineation of prokaryotic homology groups
Source: PLoS Comput Biol. 2022 Jun 8;18(6):e1010216. doi: 10.1371/journal.pcbi.1010216 (PMC9212150; doi:10.1371/journal.pcbi.1010216)
Supplement: S1 Appendix — Each marker gene is labeled with the UniProt accession number of its reference protein. MHG sequences are drawn as colored arrows overlapping the sequences from each species for each marker gene. MHG sequences drawn with the same color belong to the same MHG, although colors are repeated when more than 20 MHGs overlap a marker gene. Arrowheads indicate the relative orientation of MHG sequences to show inversions. (PDF) [file pcbi.1010216.s001.pdf]

Appendix to "Annotation-free Delineation of Prokaryotic Homology Groups"

Yongze Yin*[1], Huw A. Ogilvie†[1], and Luay Nakhleh‡[1,2]

[1]Department of Computer Science, Rice University
[2]Department of BioSciences, Rice University

April 11, 2022

*yy70@rice.edu
†huw.a.ogilvie@rice.edu
‡nakhleh@rice.edu

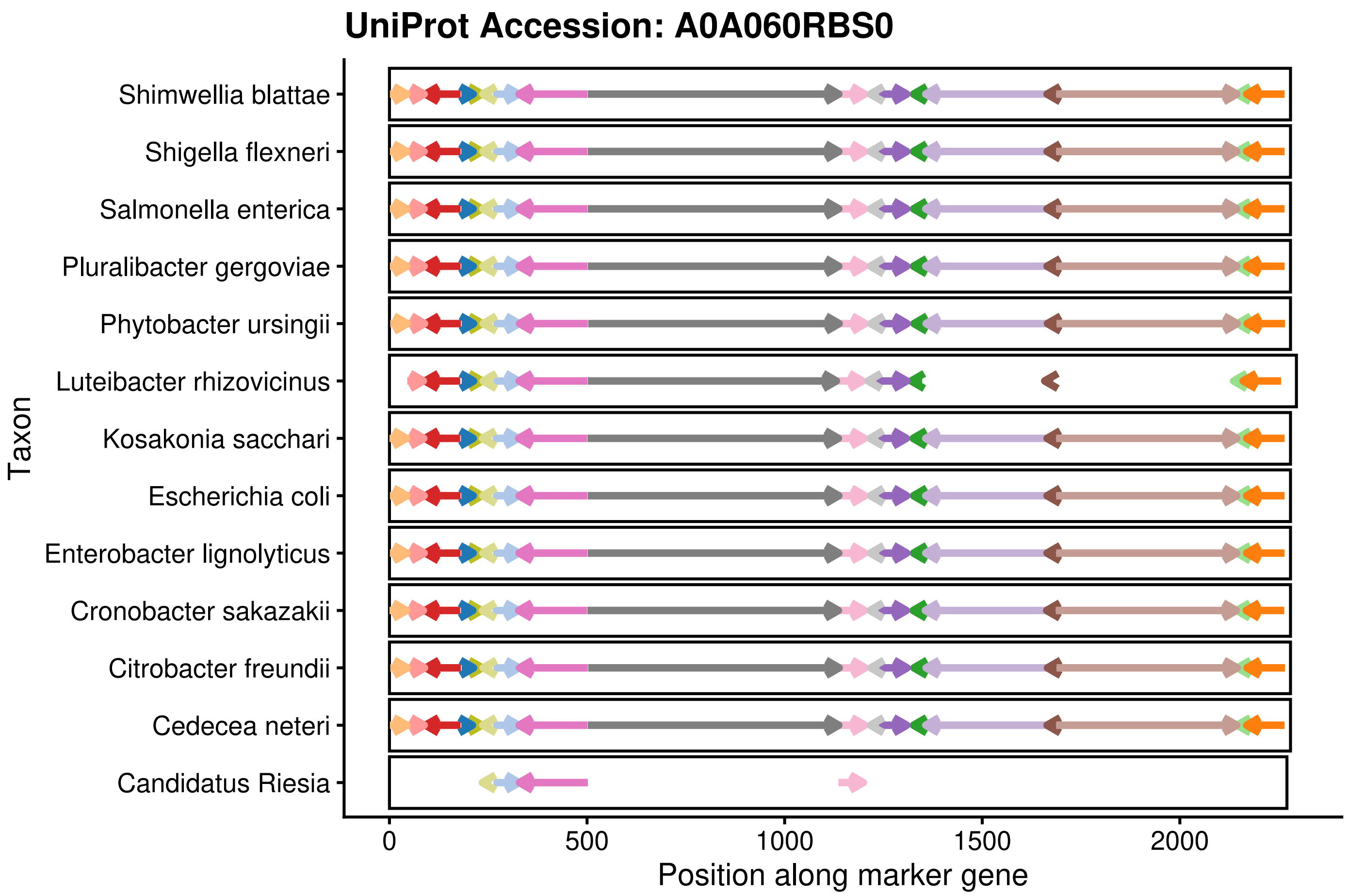
UniProt Accession: A0A060RBS0
Shimwellia blattae
Shigella flexneri
Salmonella enterica
Pluralibacter gergoviae
Phytobacter ursingii
Luteibacter rhizovicinus
Kosakonia sacchari
Escherichia coli
Enterobacter lignolyticus
Cronobacter sakazakii
Citrobacter freundii
Cedecea neteri
Candidatus Riesia
Taxon
0
500
1000
1500
2000
Position along marker gene

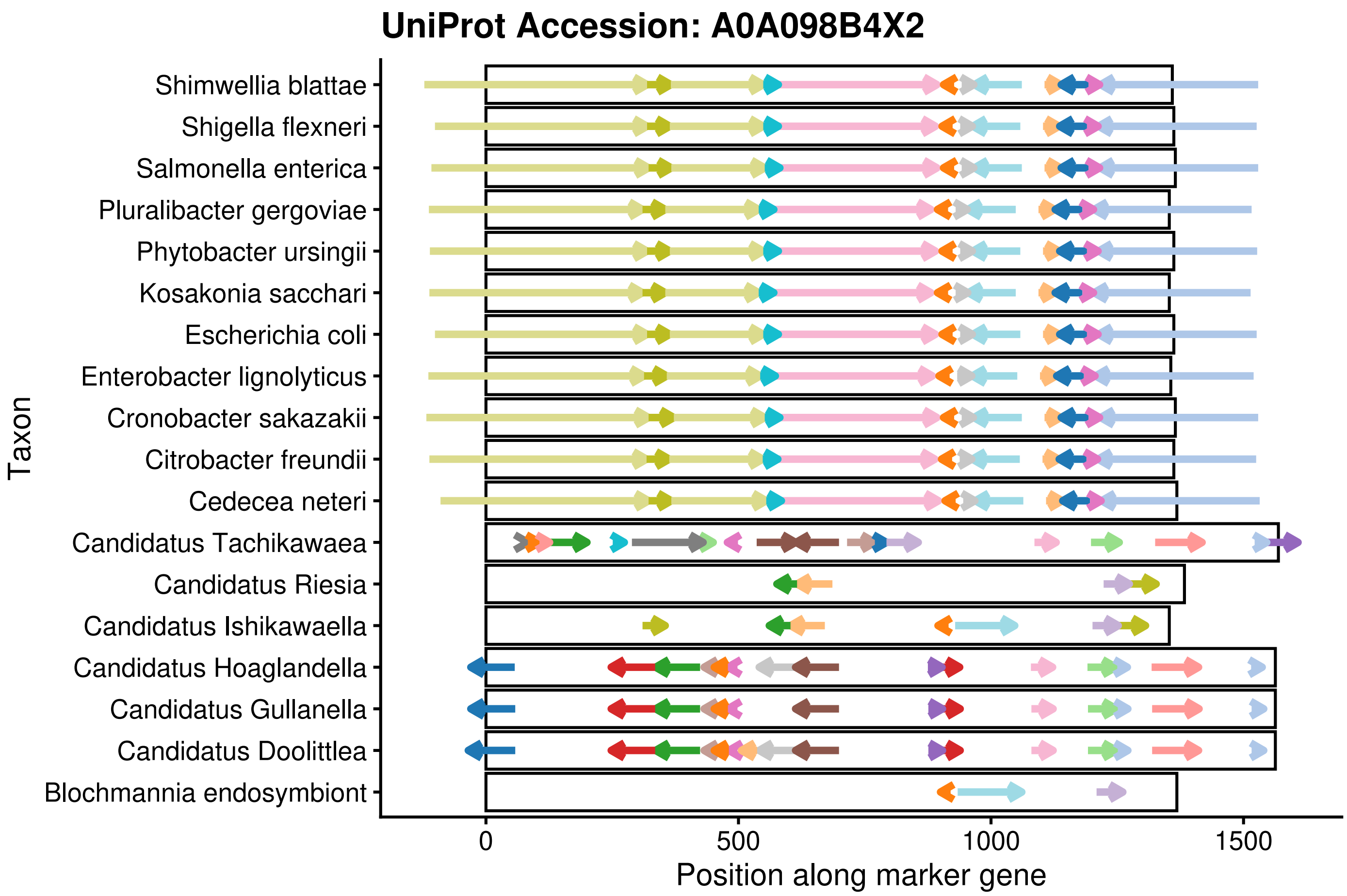
UniProt Accession: A0A098B4X2
Shimwellia blattae
Shigella flexneri
Salmonella enterica
Pluralibacter gergoviae
Phytobacter ursingii
Kosakonia sacchari
Escherichia coli
Enterobacter lignolyticus
Cronobacter sakazakii
Citrobacter freundii
Cedecea neteri
Candidatus Tachikawaea
Candidatus Riesia
Candidatus Ishikawaella
Candidatus Hoaglandella
Candidatus Gullanella
Candidatus Doolittlea
Blochmannia endosymbiont
Taxon
0
500
1000
1500
Position along marker gene

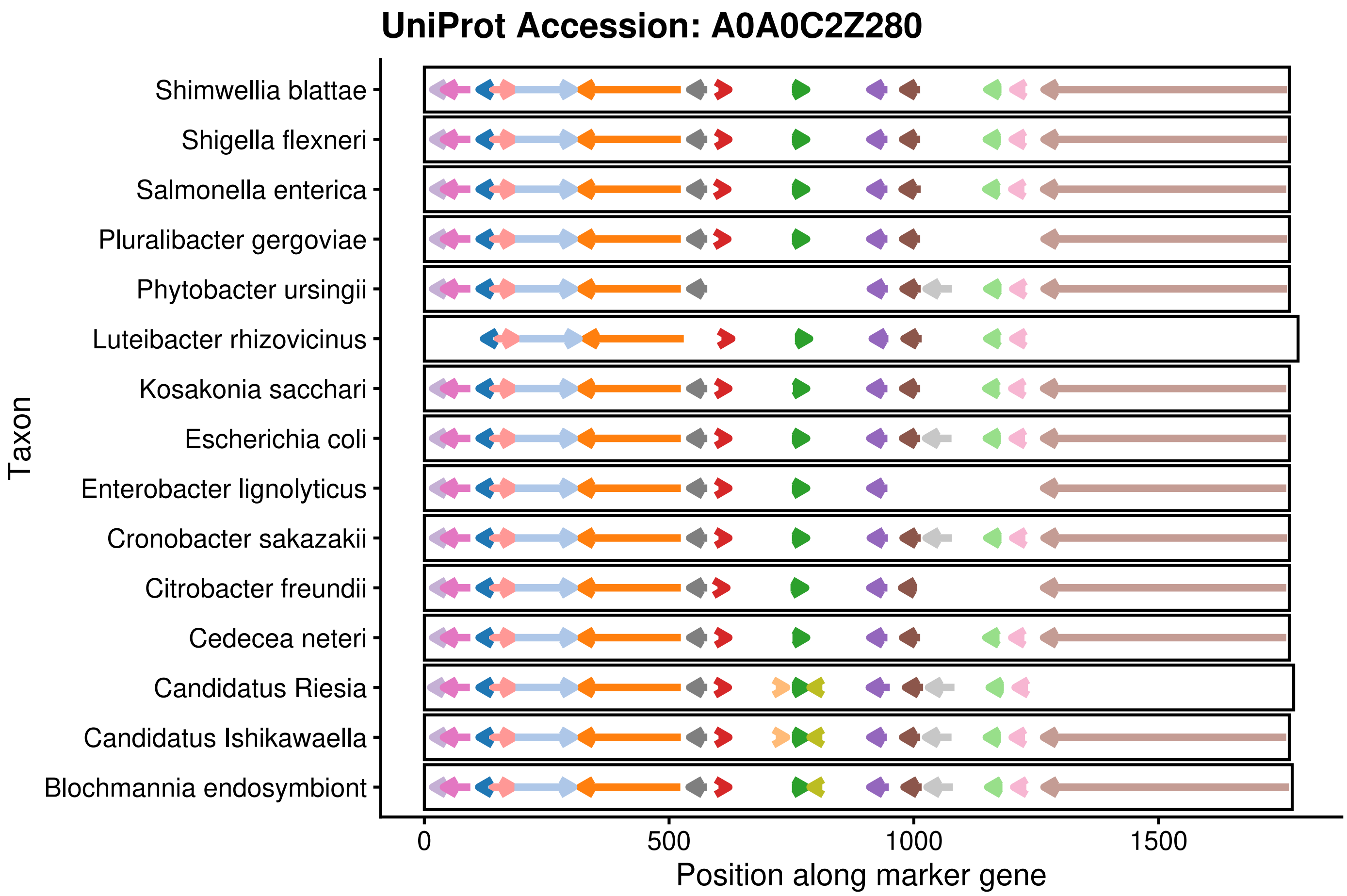
UniProt Accession: A0A0C2Z280
Taxon
Shimwellia blattae
Shigella flexneri
Salmonella enterica
Pluralibacter gergoviae
Phytobacter ursingii
Luteibacter rhizovicinus
Kosakonia sacchari
Escherichia coli
Enterobacter lignolyticus
Cronobacter sakazakii
Citrobacter freundii
Cedecea neteri
Candidatus Riesia
Candidatus Ishikawaella
Blochmannia endosymbiont
0
500
1000
1500
Position along marker gene

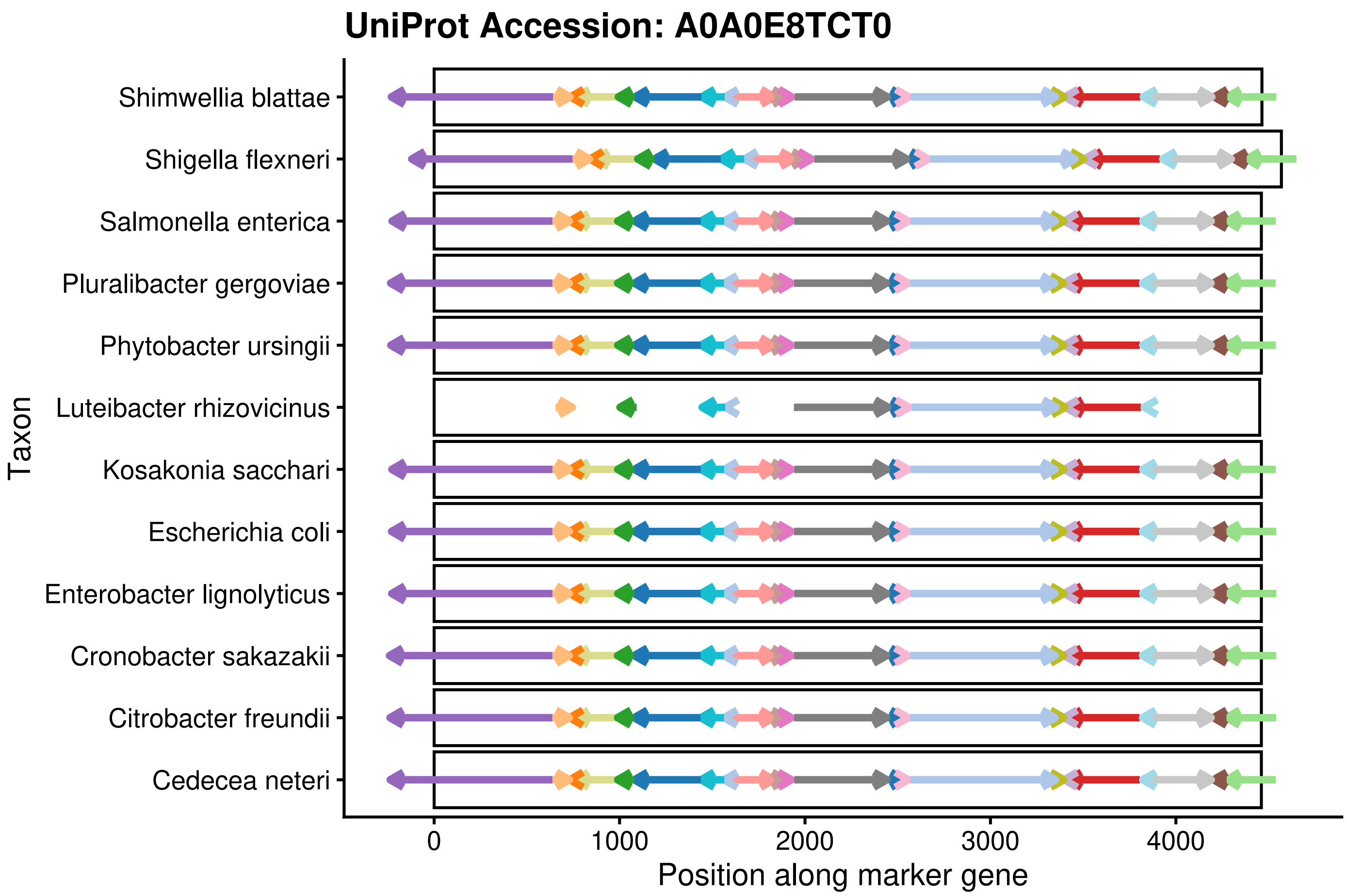
UniProt Accession: A0A0E8TCT0
Shimwellia blattae
Shigella flexneri
Salmonella enterica
Pluralibacter gergoviae
Phytobacter ursingii
Luteibacter rhizovicinus
Kosakonia sacchari
Escherichia coli
Enterobacter lignolyticus
Cronobacter sakazakii
Citrobacter freundii
Cedecea neteri
Taxon
0
1000
2000
3000
4000
Position along marker gene

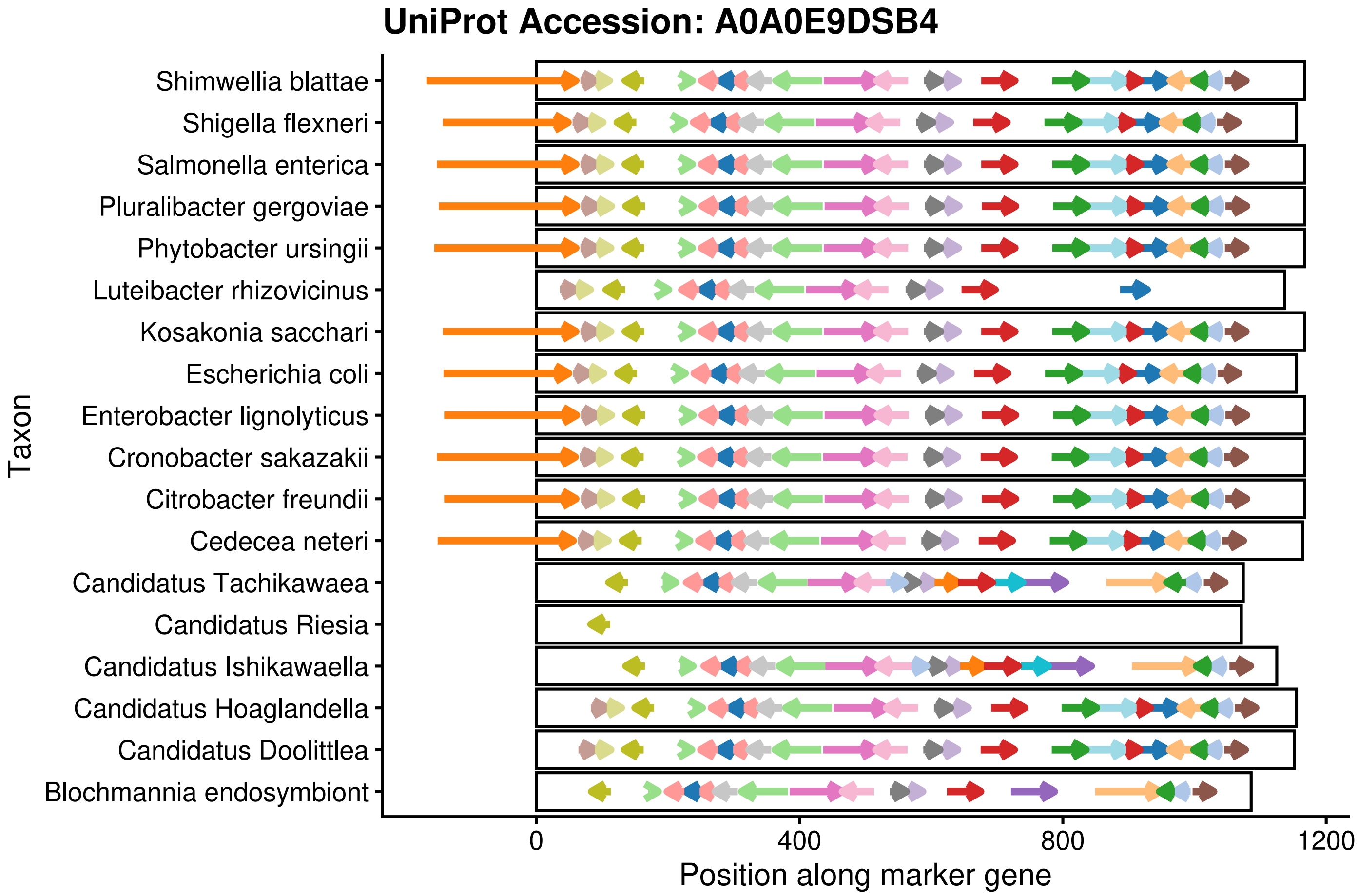
UniProt Accession: A0A0E9DSB4
Shimwellia blattae
Shigella flexneri
Salmonella enterica
Pluralibacter gergoviae
Phytobacter ursingii
Luteibacter rhizovicinus
Kosakonia sacchari
Escherichia coli
Enterobacter lignolyticus
Cronobacter sakazakii
Citrobacter freundii
Cedecea neteri
Candidatus Tachikawaea
Candidatus Riesia
Candidatus Ishikawaella
Candidatus Hoaglandella
Candidatus Doolittlea
Blochmannia endosymbiont
Taxon
0
400
800
1200
Position along marker gene

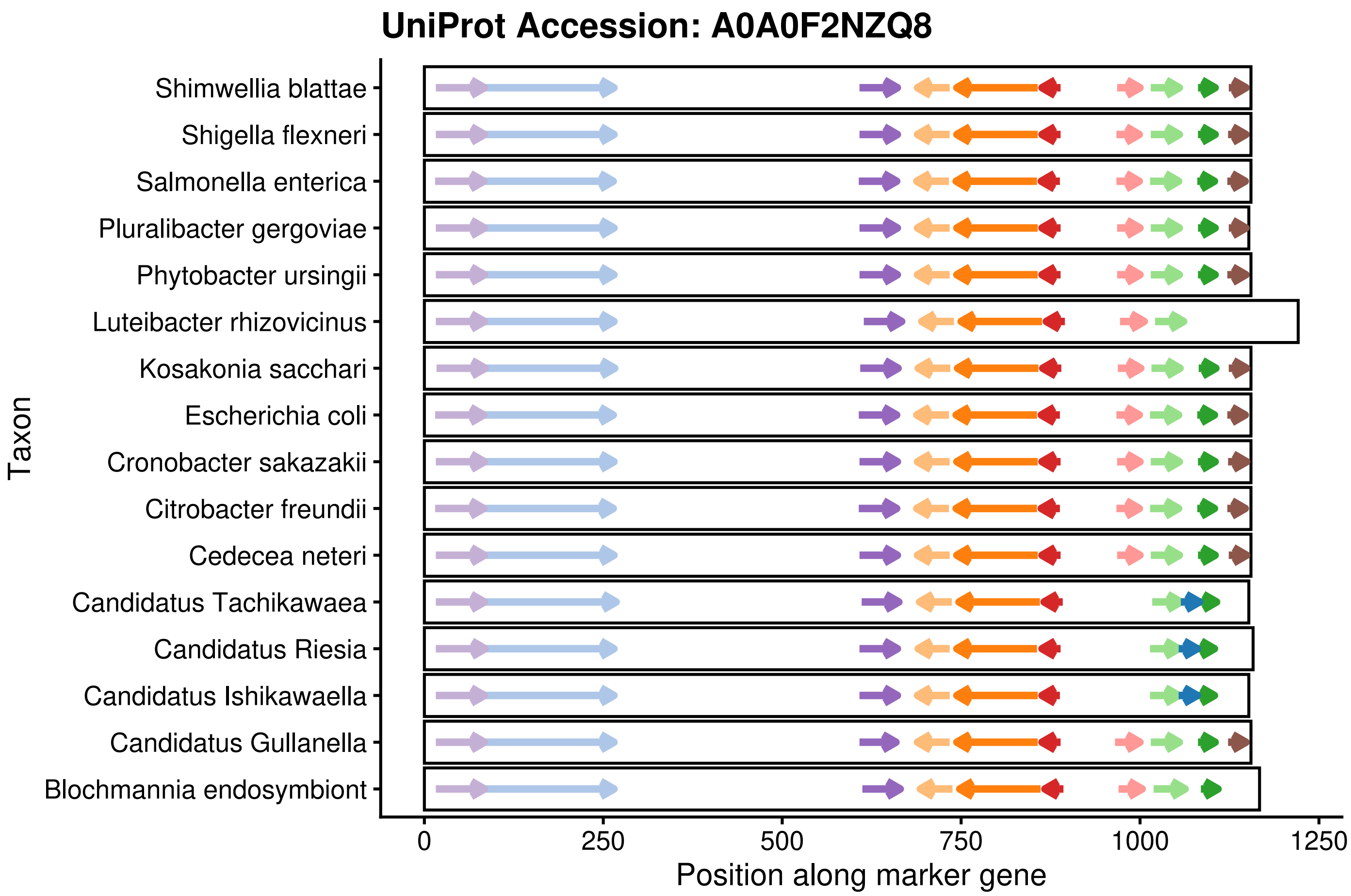

UniProt Accession: A0A0F2NZQ8
Shimwellia blattae
Shigella flexneri
Salmonella enterica
Pluralibacter gergoviae
Phytobacter ursingii
Luteibacter rhizovicinus
Kosakonia sacchari
Escherichia coli
Cronobacter sakazakii
Citrobacter freundii
Cedecea neteri
Candidatus Tachikawaea
Candidatus Riesia
Candidatus Ishikawaella
Candidatus Gullanella
Blochmannia endosymbiont
Taxon
0
250
500
750
1000
1250
Position along marker gene

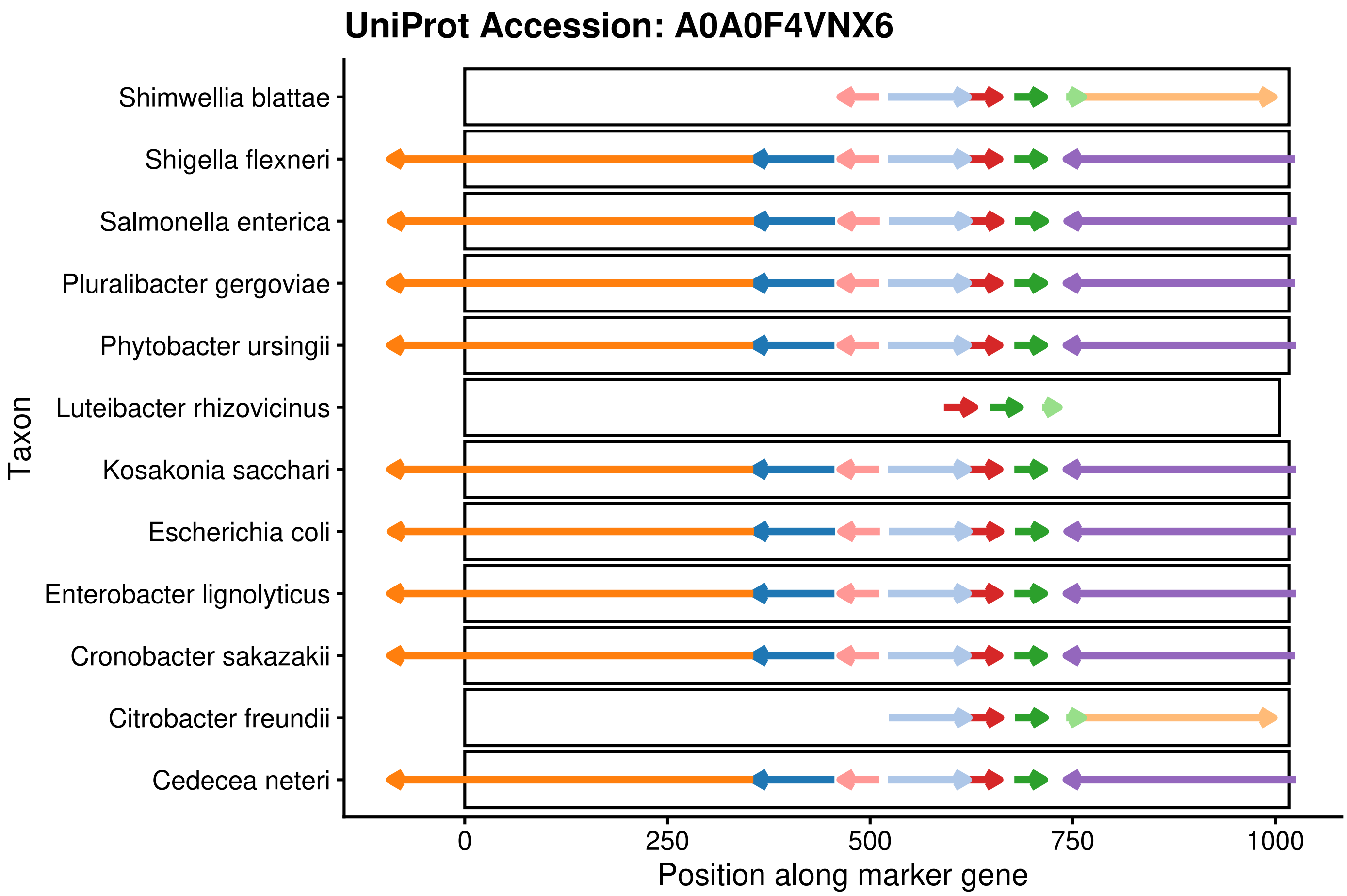
UniProt Accession: A0A0F4VNX6
Taxon
Shimwellia blattae
Shigella flexneri
Salmonella enterica
Pluralibacter gergoviae
Phytobacter ursingii
Luteibacter rhizovicinus
Kosakonia sacchari
Escherichia coli
Enterobacter lignolyticus
Cronobacter sakazakii
Citrobacter freundii
Cedecea neteri
0
250
500
750
1000
Position along marker gene

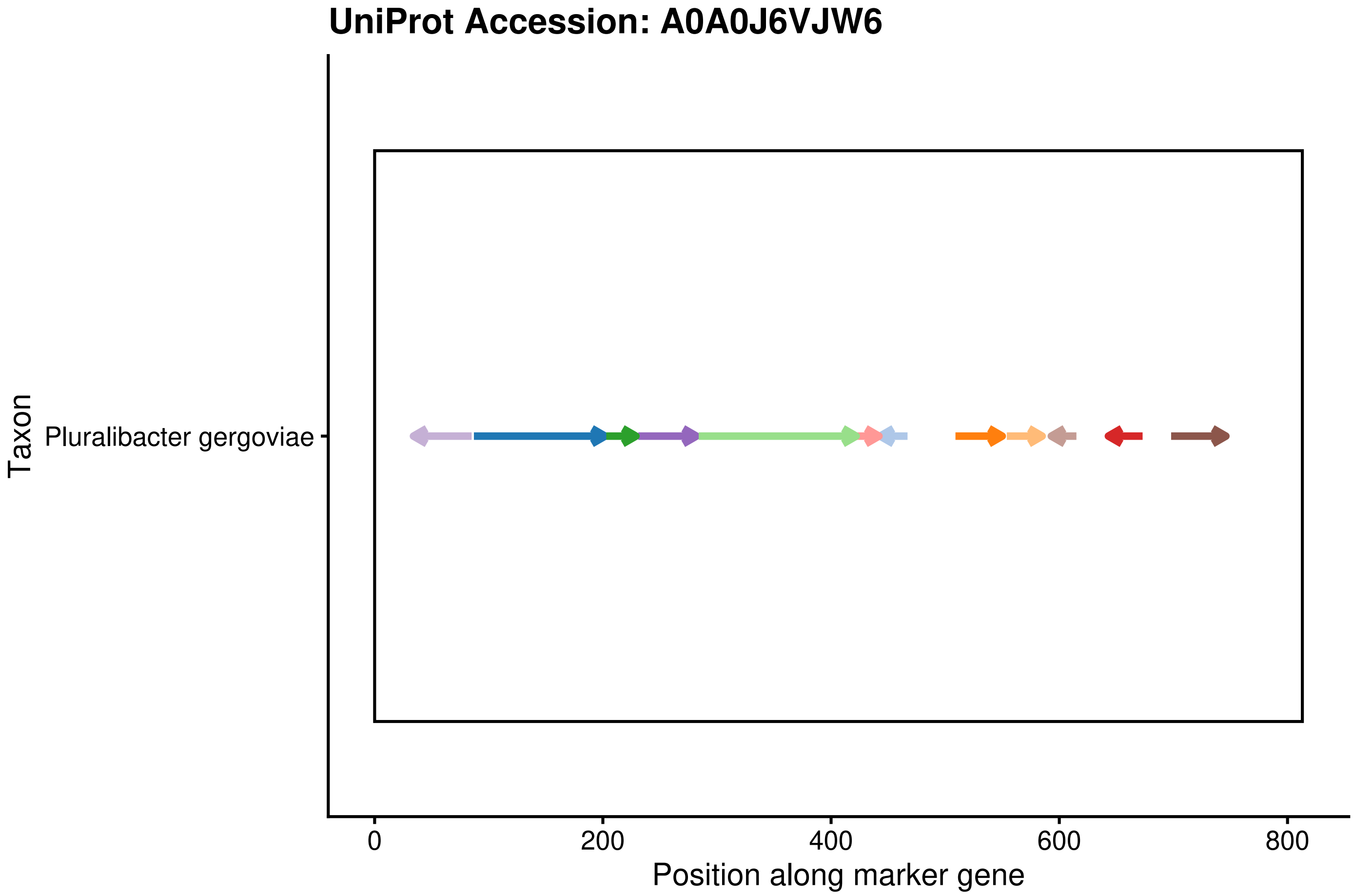
UniProt Accession: A0A0J6VJW6
Taxon
Pluralibacter gergoviae
0
200
400
600
800
Position along marker gene

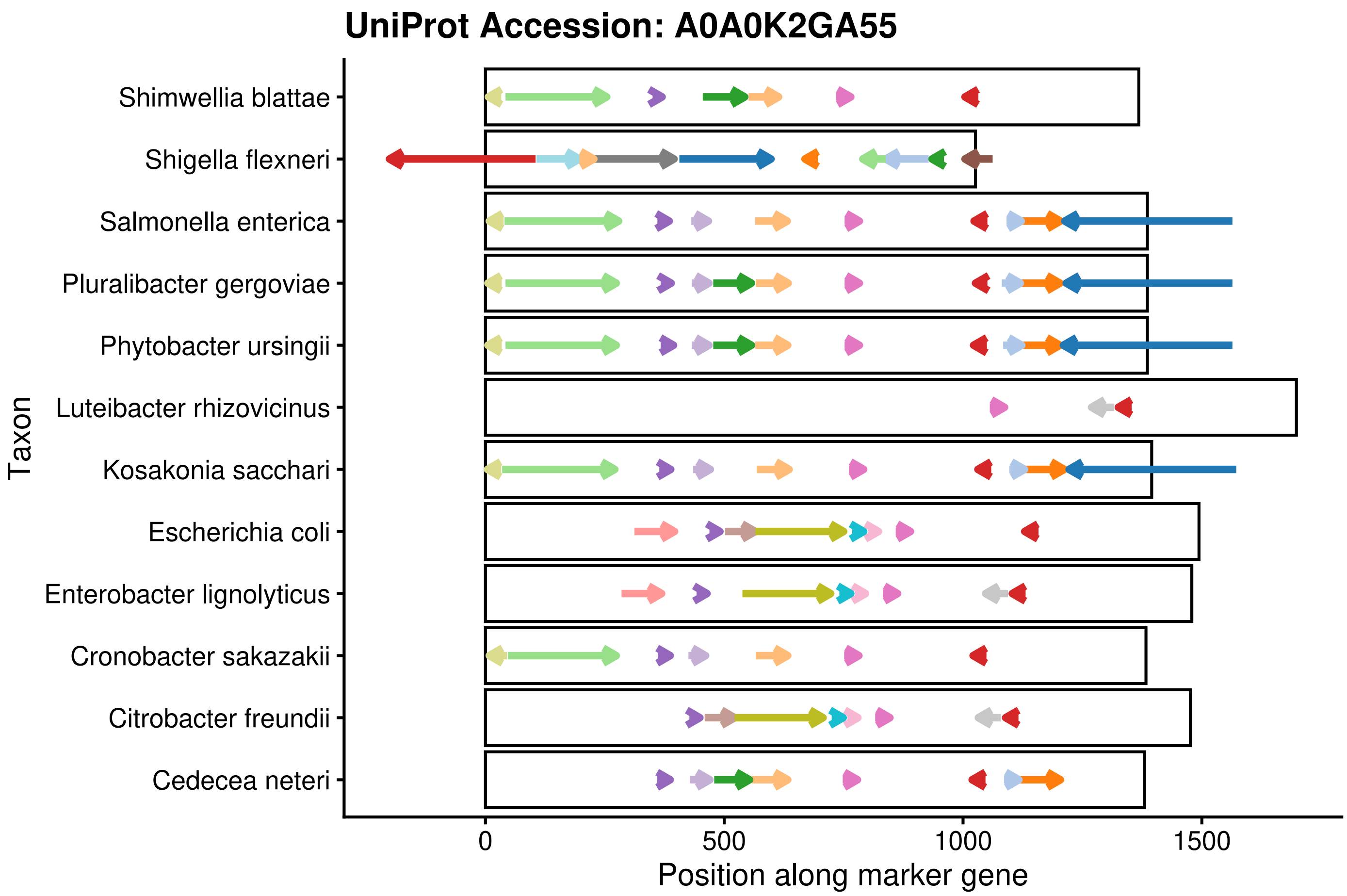
UniProt Accession: A0A0K2GA55
Shimwellia blattae
Shigella flexneri
Salmonella enterica
Pluralibacter gergoviae
Phytobacter ursingii
Luteibacter rhizovicinus
Kosakonia sacchari
Escherichia coli
Enterobacter lignolyticus
Cronobacter sakazakii
Citrobacter freundii
Cedecea neteri
Taxon
0
500
1000
1500
Position along marker gene

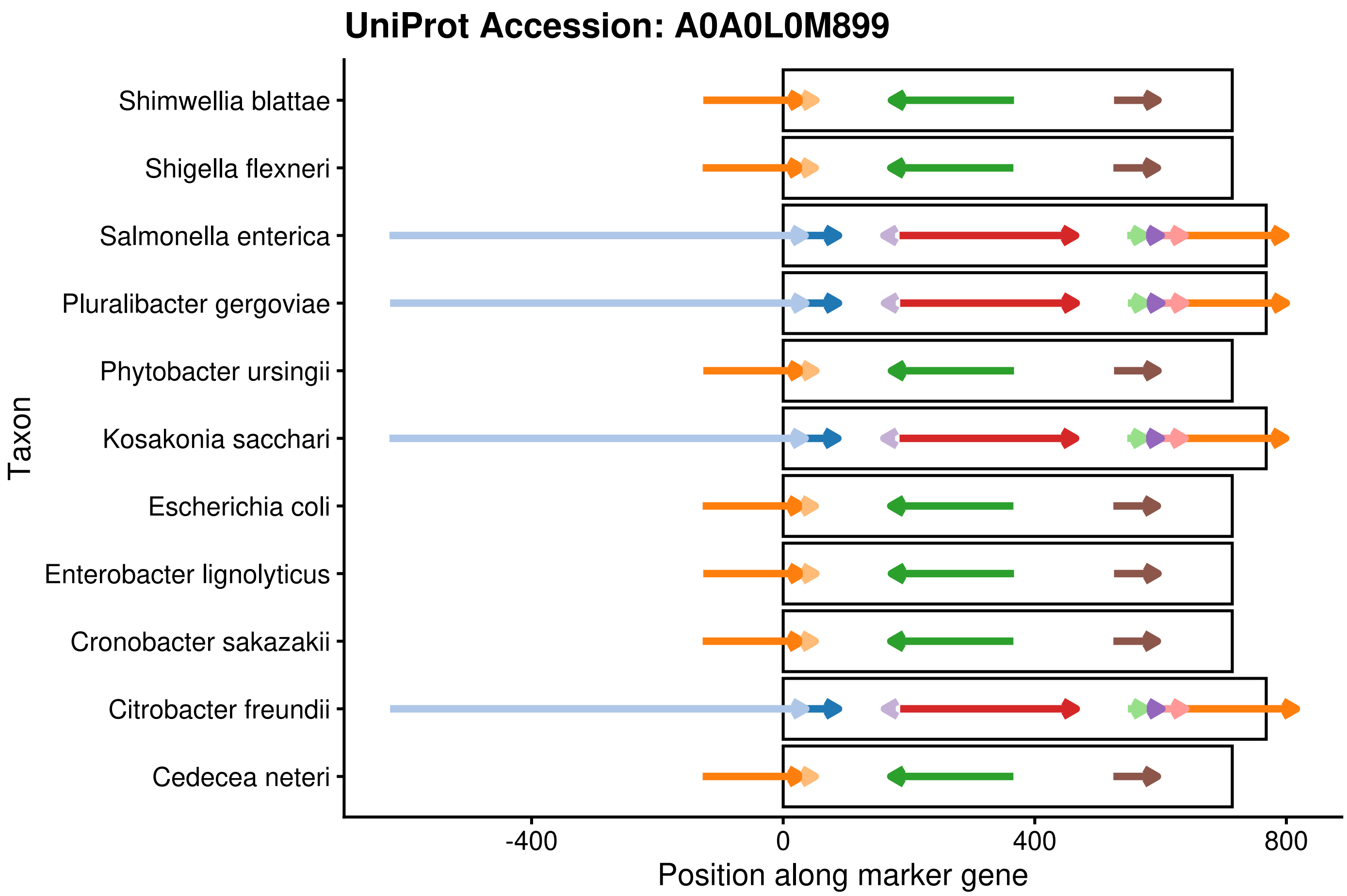

UniProt Accession: A0A0L0M899
Shimwellia blattae
Shigella flexneri
Salmonella enterica
Pluralibacter gergoviae
Phytobacter ursingii
Kosakonia sacchari
Escherichia coli
Enterobacter lignolyticus
Cronobacter sakazakii
Citrobacter freundii
Cedecea neteri
Taxon
-400
0
400
800
Position along marker gene

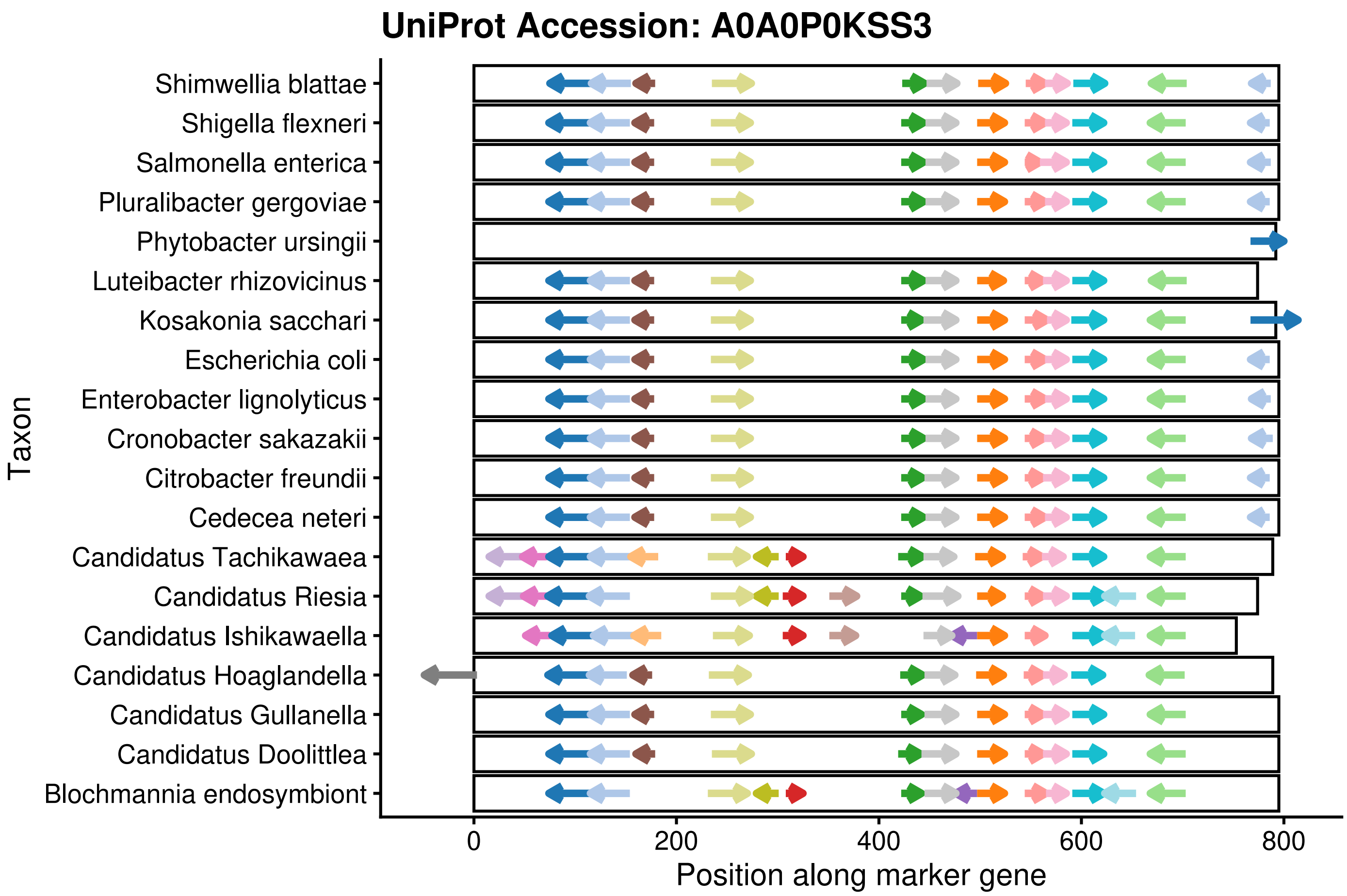
UniProt Accession: A0A0P0KSS3
Shimwellia blattae
Shigella flexneri
Salmonella enterica
Pluralibacter gergoviae
Phytobacter ursingii
Luteibacter rhizovicinus
Kosakonia sacchari
Escherichia coli
Enterobacter lignolyticus
Cronobacter sakazakii
Citrobacter freundii
Cedecea neteri
Candidatus Tachikawaea
Candidatus Riesia
Candidatus Ishikawaella
Candidatus Hoaglandella
Candidatus Gullanella
Candidatus Doolittlea
Blochmannia endosymbiont
Taxon
0
200
400
600
800
Position along marker gene

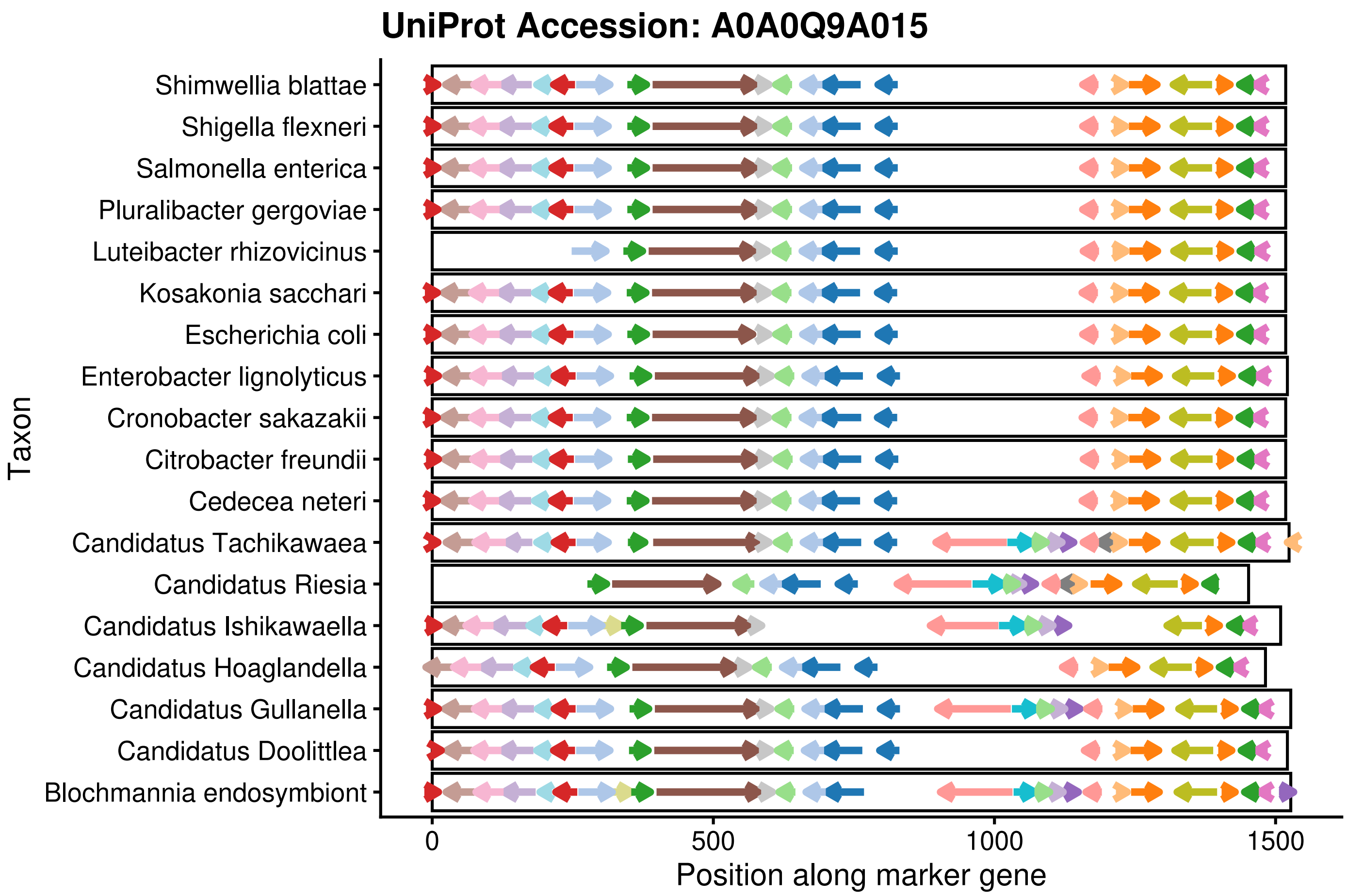

UniProt Accession: A0A0Q9A015
Taxon
Shimwellia blattae
Shigella flexneri
Salmonella enterica
Pluralibacter gergoviae
Luteibacter rhizovicinus
Kosakonia sacchari
Escherichia coli
Enterobacter lignolyticus
Cronobacter sakazakii
Citrobacter freundii
Cedecea neteri
Candidatus Tachikawaea
Candidatus Riesia
Candidatus Ishikawaella
Candidatus Hoaglandella
Candidatus Gullanella
Candidatus Doolittlea
Blochmannia endosymbiont
0
500
1000
1500
Position along marker gene

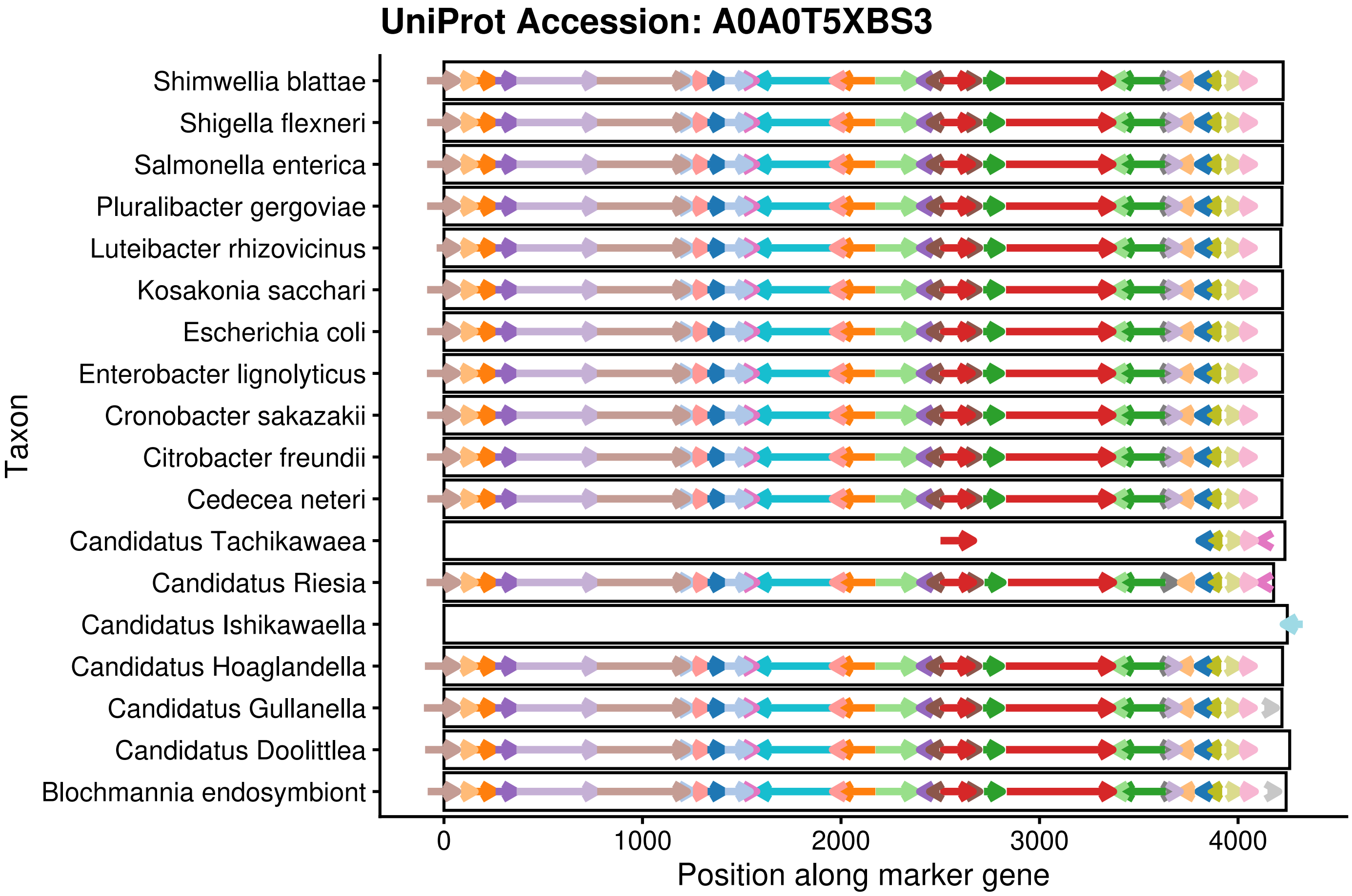

UniProt Accession: A0A0T5XBS3
Taxon
Shimwellia blattae
Shigella flexneri
Salmonella enterica
Pluralibacter gergoviae
Luteibacter rhizovicinus
Kosakonia sacchari
Escherichia coli
Enterobacter lignolyticus
Cronobacter sakazakii
Citrobacter freundii
Cedecea neteri
Candidatus Tachikawaea
Candidatus Riesia
Candidatus Ishikawaella
Candidatus Hoaglandella
Candidatus Gullanella
Candidatus Doolittlea
Blochmannia endosymbiont
0
1000
2000
3000
4000
Position along marker gene

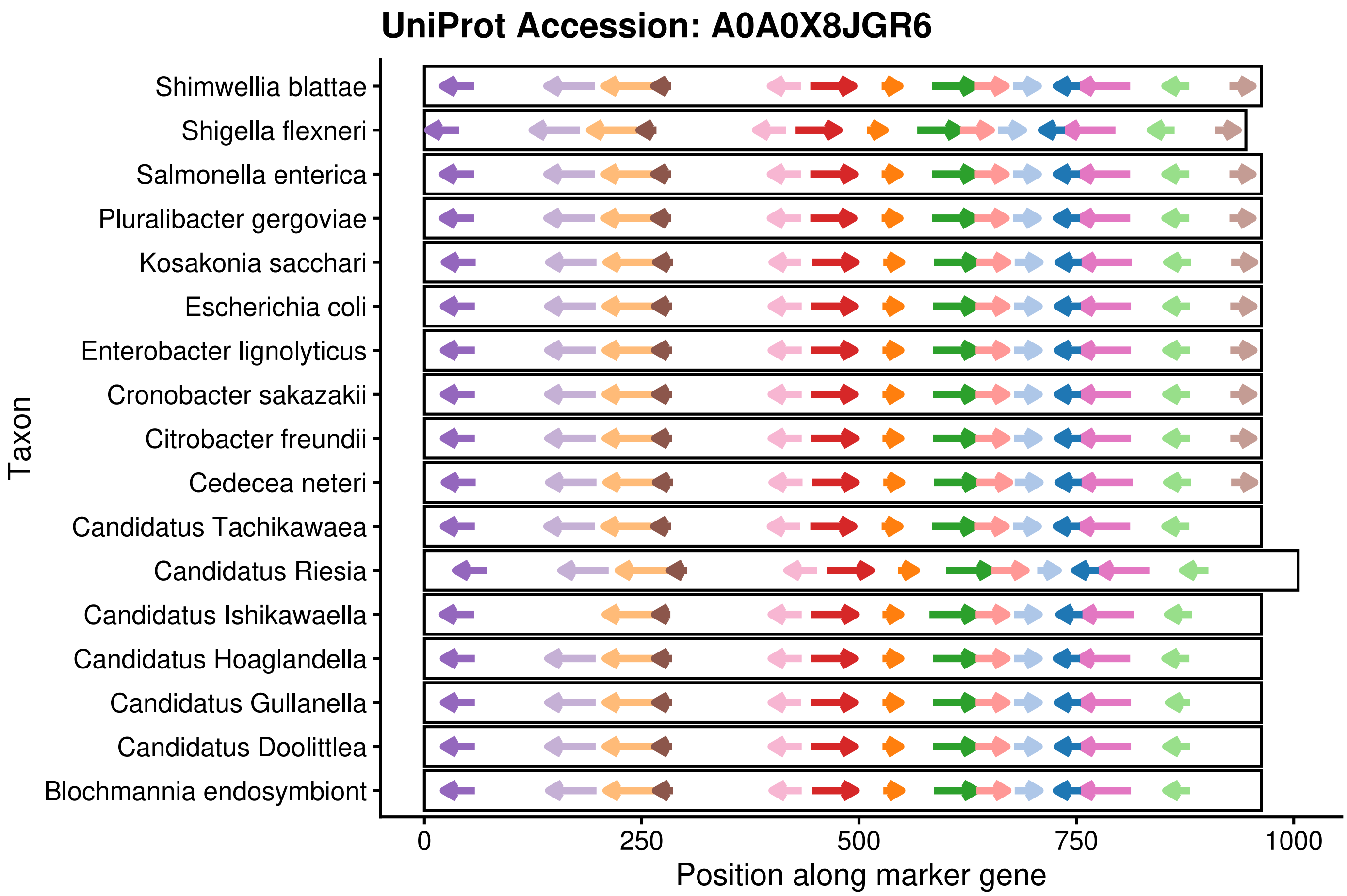
UniProt Accession: A0A0X8JGR6
Taxon
Shimwellia blattae
Shigella flexneri
Salmonella enterica
Pluralibacter gergoviae
Kosakonia sacchari
Escherichia coli
Enterobacter lignolyticus
Cronobacter sakazakii
Citrobacter freundii
Cedecea neteri
Candidatus Tachikawaea
Candidatus Riesia
Candidatus Ishikawaella
Candidatus Hoaglandella
Candidatus Gullanella
Candidatus Doolittlea
Blochmannia endosymbiont
0
250
500
750
1000
Position along marker gene

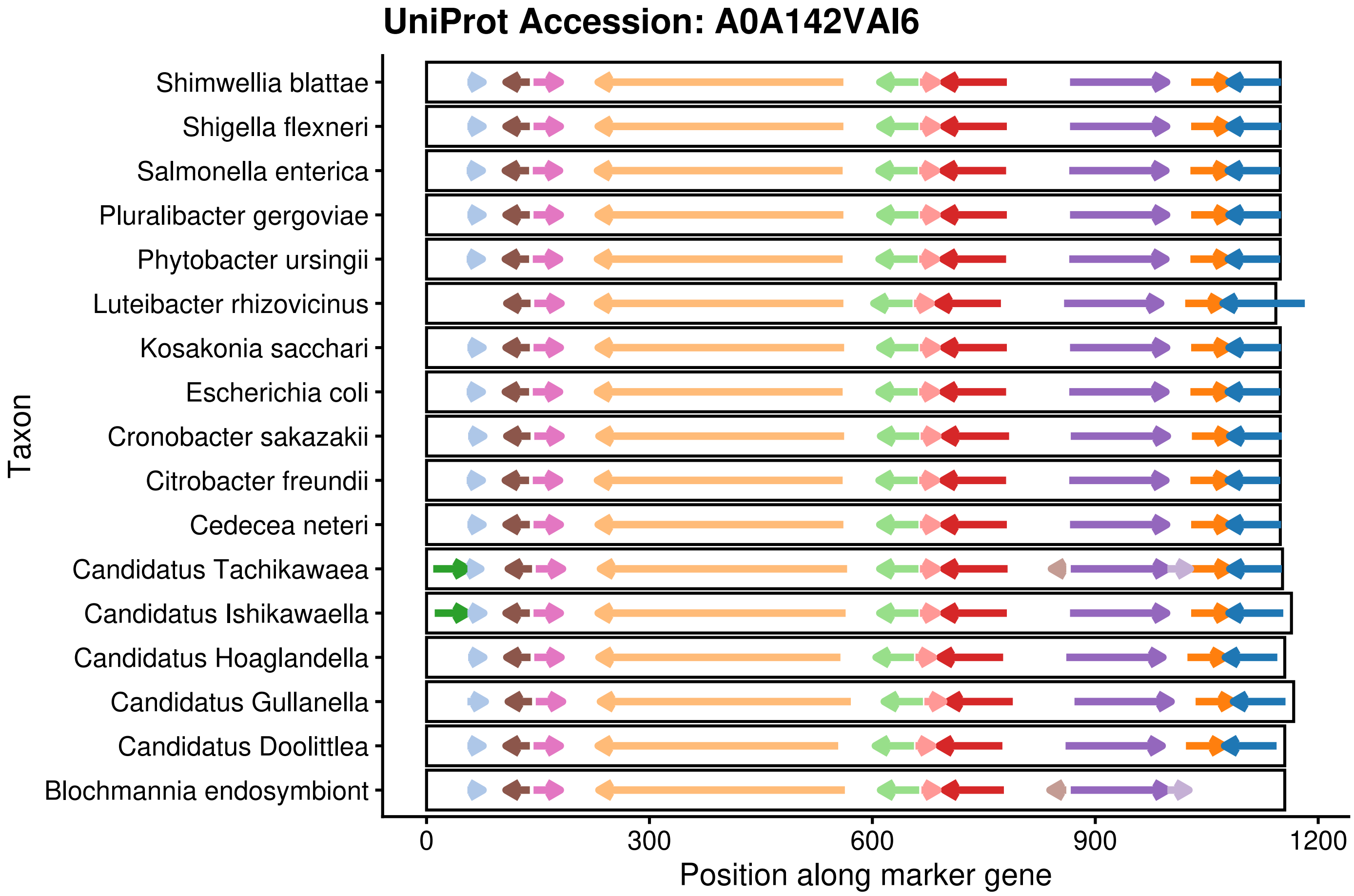

UniProt Accession: A0A142VAI6
Shimwellia blattae
Shigella flexneri
Salmonella enterica
Pluralibacter gergoviae
Phytobacter ursingii
Luteibacter rhizovicinus
Kosakonia sacchari
Escherichia coli
Cronobacter sakazakii
Citrobacter freundii
Cedecea neteri
Candidatus Tachikawaea
Candidatus Ishikawaella
Candidatus Hoaglandella
Candidatus Gullanella
Candidatus Doolittlea
Blochmannia endosymbiont
Taxon
0
300
600
900
1200
Position along marker gene

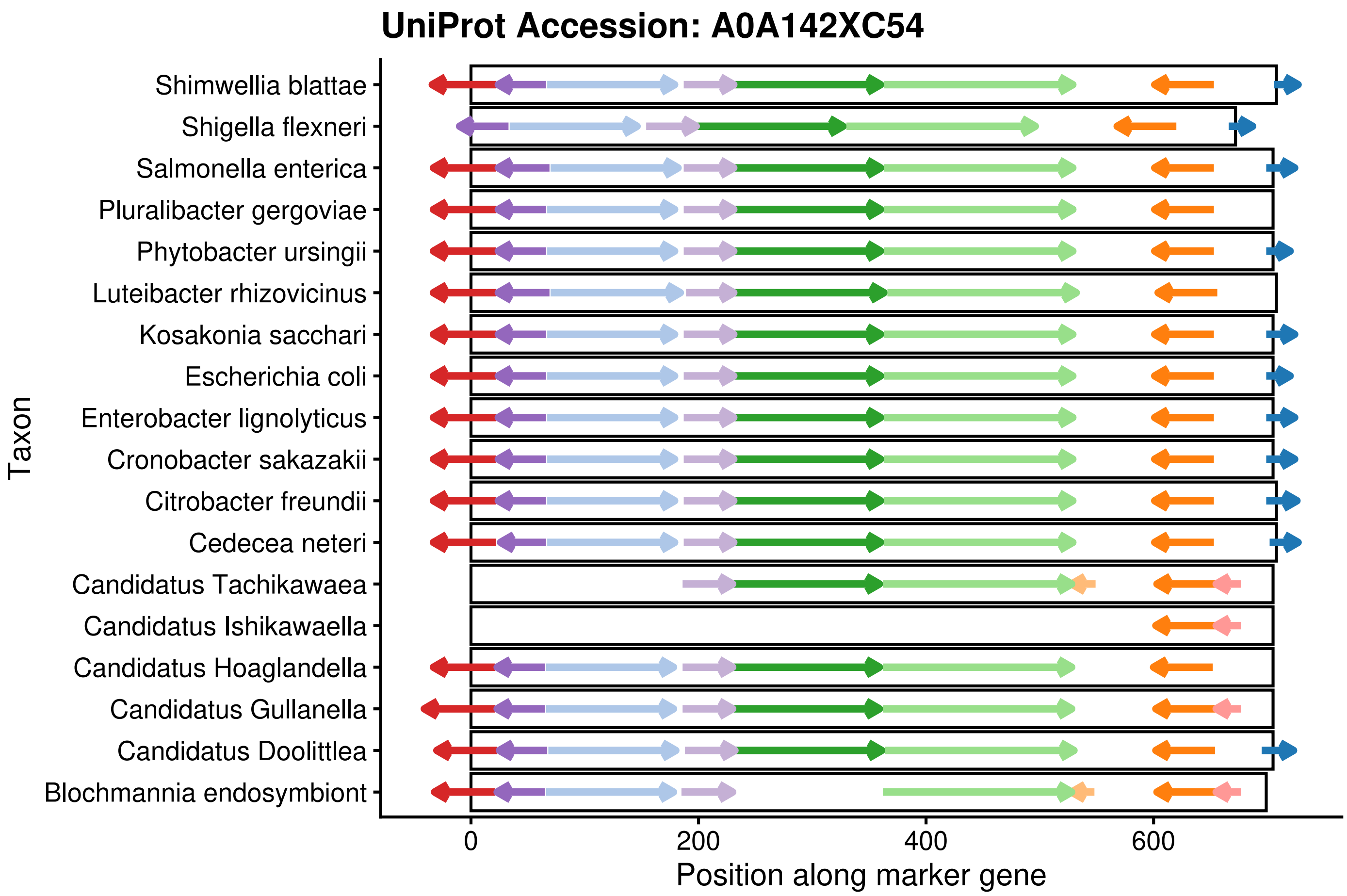

UniProt Accession: A0A142XC54
Shimwellia blattae
Shigella flexneri
Salmonella enterica
Pluralibacter gergoviae
Phytobacter ursingii
Luteibacter rhizovicinus
Kosakonia sacchari
Escherichia coli
Enterobacter lignolyticus
Cronobacter sakazakii
Citrobacter freundii
Cedecea neteri
Candidatus Tachikawaea
Candidatus Ishikawaella
Candidatus Hoaglandella
Candidatus Gullanella
Candidatus Doolittlea
Blochmannia endosymbiont
Taxon
0
200
400
600
Position along marker gene

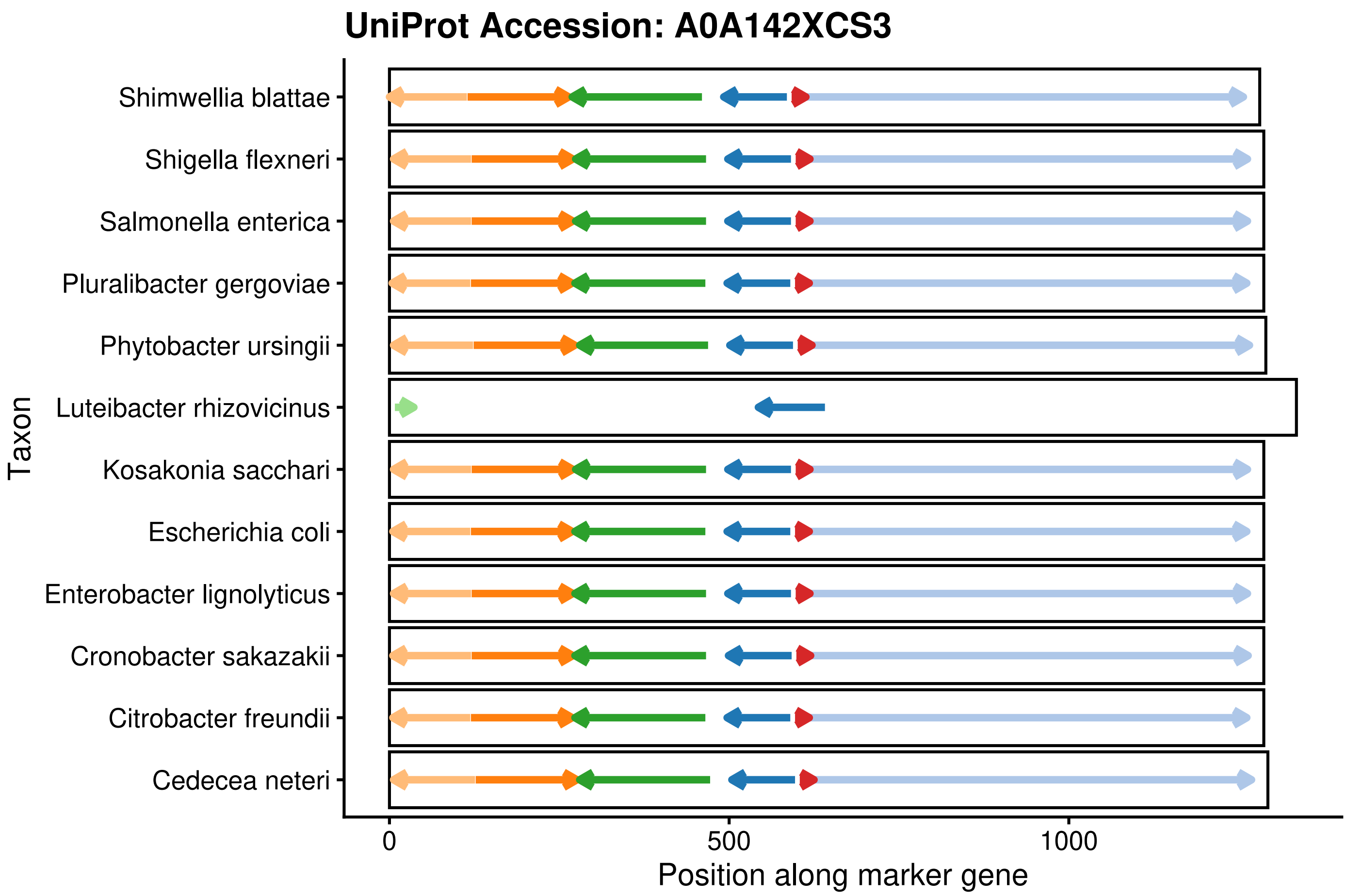
UniProt Accession: A0A142XCS3
Shimwellia blattae
Shigella flexneri
Salmonella enterica
Pluralibacter gergoviae
Phytobacter ursingii
Luteibacter rhizovicinus
Kosakonia sacchari
Escherichia coli
Enterobacter lignolyticus
Cronobacter sakazakii
Citrobacter freundii
Cedecea neteri
Taxon
0
500
1000
Position along marker gene

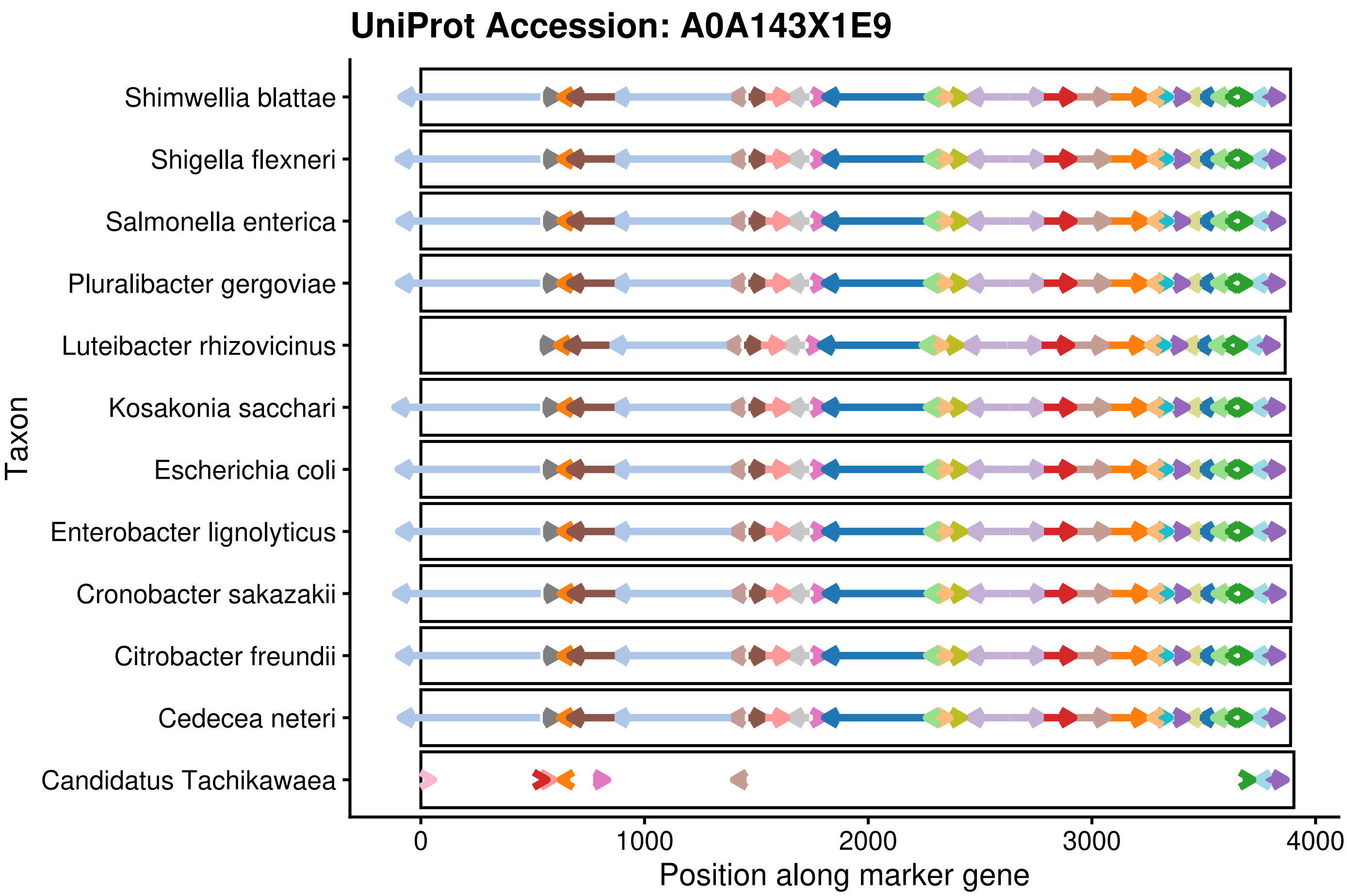
UniProt Accession: A0A143X1E9
Shimwellia blattae
Shigella flexneri
Salmonella enterica
Pluralibacter gergoviae
Luteibacter rhizovicinus
Kosakonia sacchari
Escherichia coli
Enterobacter lignolyticus
Cronobacter sakazakii
Citrobacter freundii
Cedecea neteri
Candidatus Tachikawaea
Taxon
0
1000
2000
3000
4000
Position along marker gene

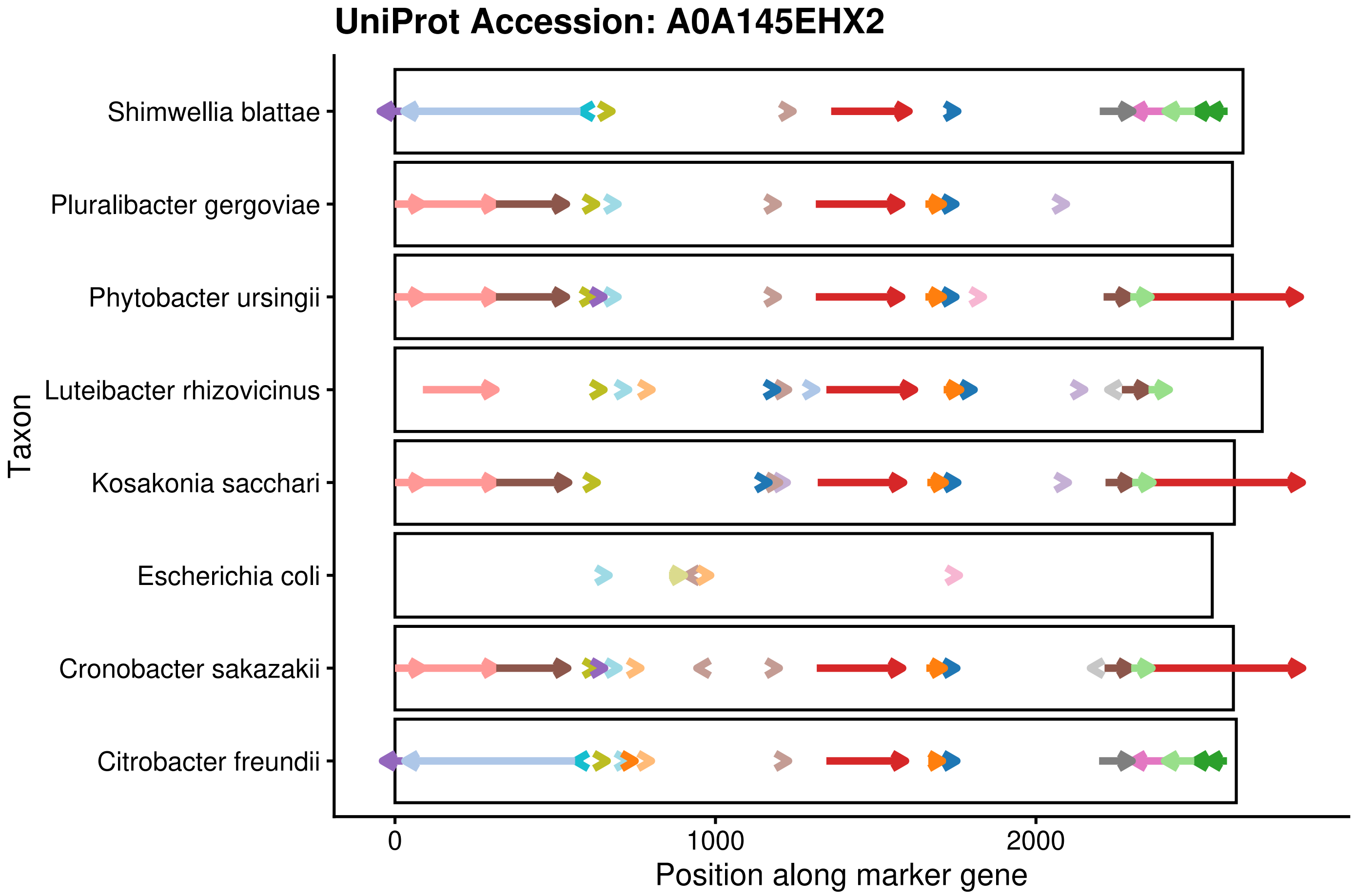
UniProt Accession: A0A145EHX2
Taxon
Shimwellia blattae
Pluralibacter gergoviae
Phytobacter ursingii
Luteibacter rhizovicinus
Kosakonia sacchari
Escherichia coli
Cronobacter sakazakii
Citrobacter freundii
0
1000
2000
Position along marker gene

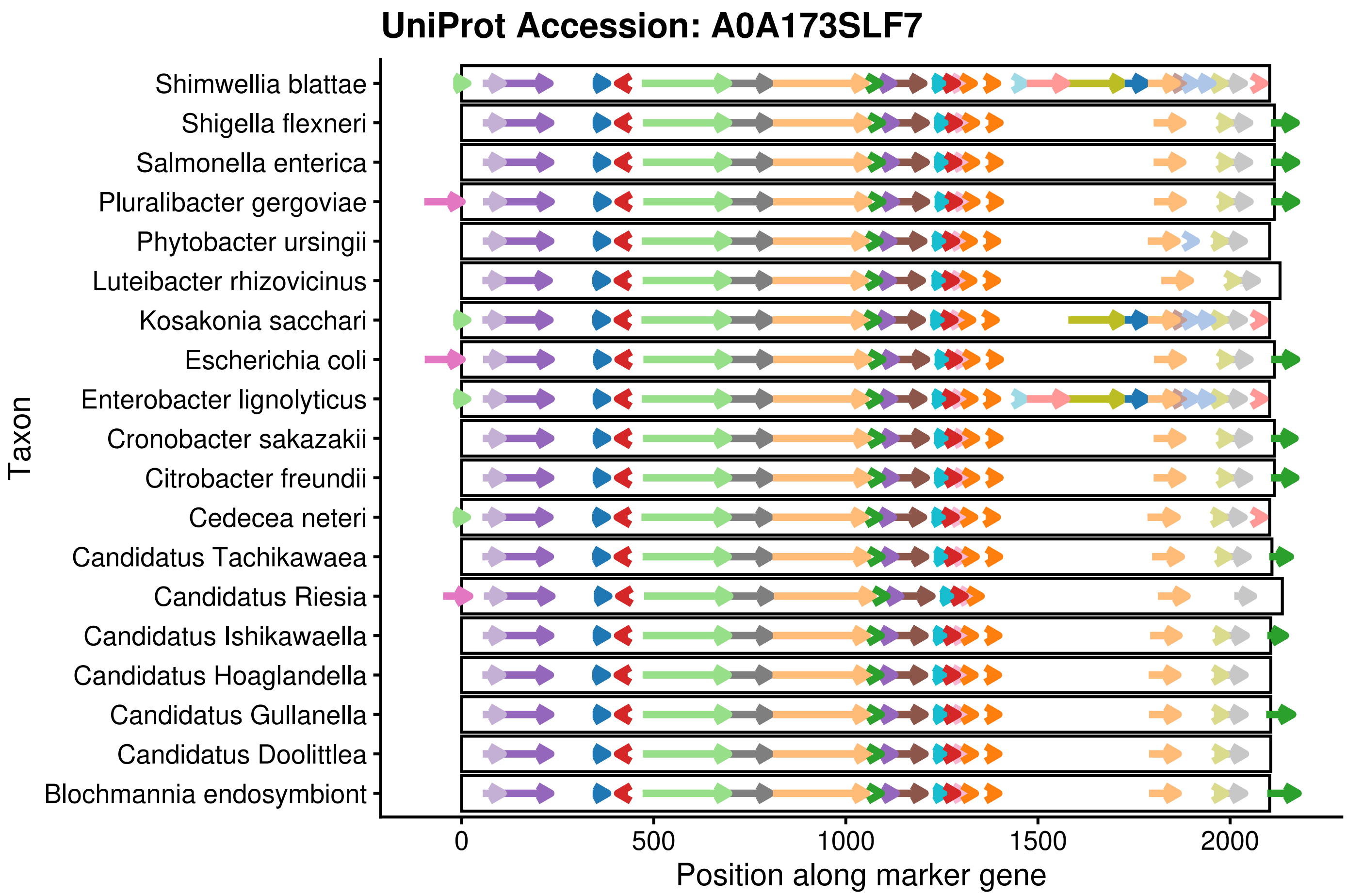
UniProt Accession: A0A173SLF7
Shimwellia blattae
Shigella flexneri
Salmonella enterica
Pluralibacter gergoviae
Phytobacter ursingii
Luteibacter rhizovicinus
Kosakonia sacchari
Escherichia coli
Enterobacter lignolyticus
Cronobacter sakazakii
Citrobacter freundii
Cedecea neteri
Candidatus Tachikawaea
Candidatus Riesia
Candidatus Ishikawaella
Candidatus Hoaglandella
Candidatus Gullanella
Candidatus Doolittlea
Blochmannia endosymbiont
Taxon
0
500
1000
1500
2000
Position along marker gene

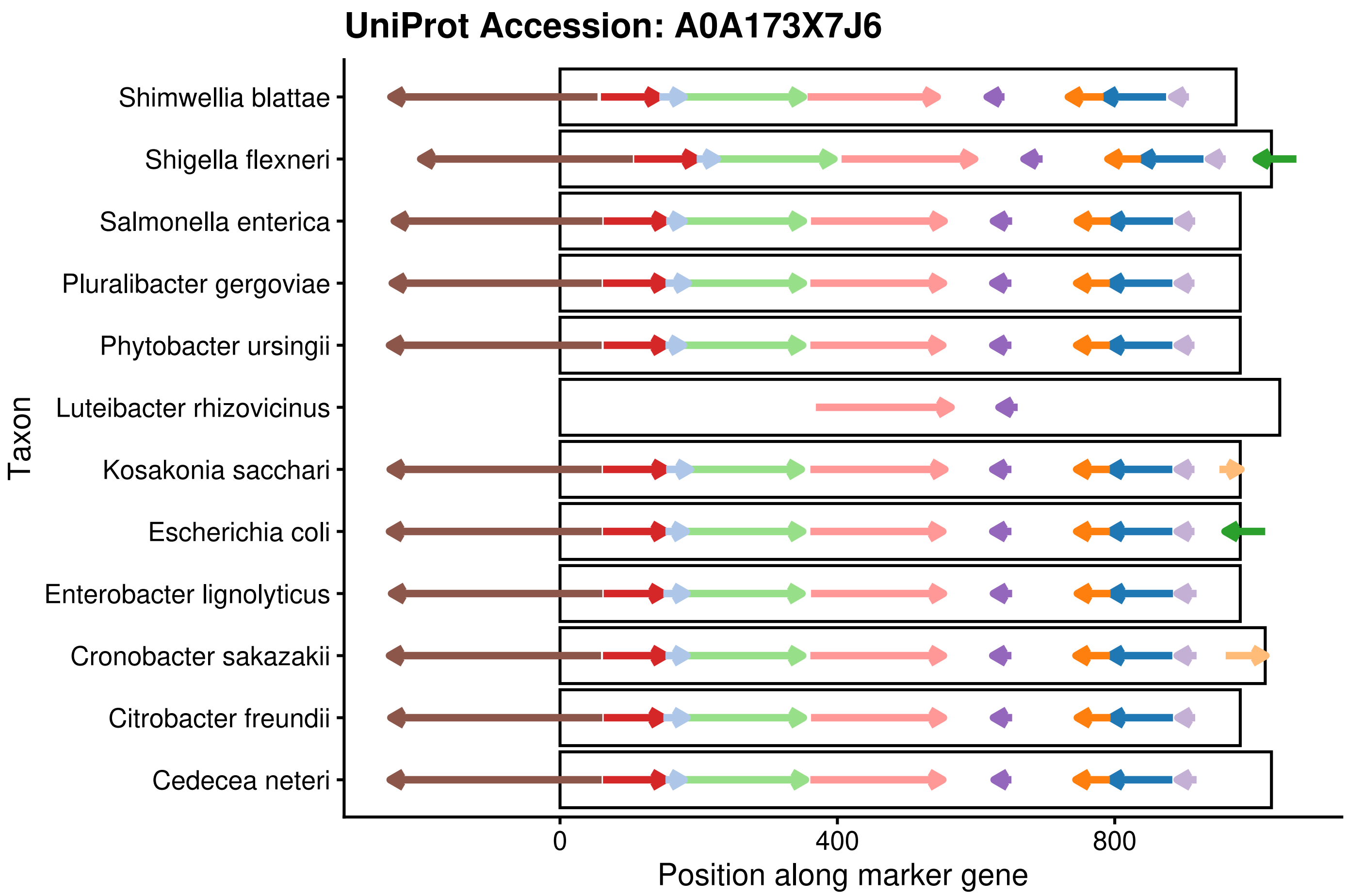
UniProt Accession: A0A173X7J6
Shimwellia blattae
Shigella flexneri
Salmonella enterica
Pluralibacter gergoviae
Phytobacter ursingii
Luteibacter rhizovicinus
Kosakonia sacchari
Escherichia coli
Enterobacter lignolyticus
Cronobacter sakazakii
Citrobacter freundii
Cedecea neteri
Taxon
0
400
800
Position along marker gene

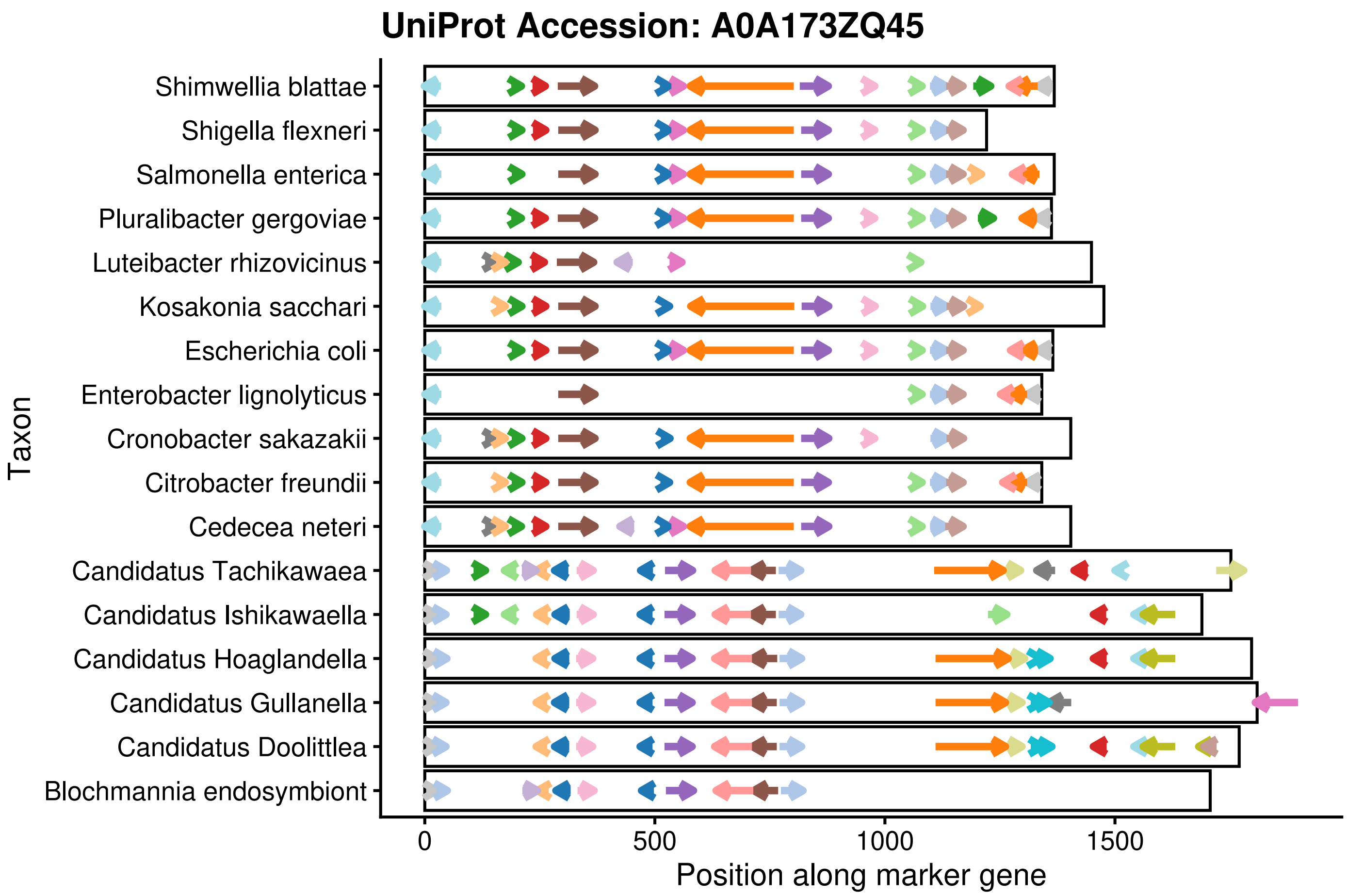
UniProt Accession: A0A173ZQ45
Shimwellia blattae
Shigella flexneri
Salmonella enterica
Pluralibacter gergoviae
Luteibacter rhizovicinus
Kosakonia sacchari
Escherichia coli
Enterobacter lignolyticus
Cronobacter sakazakii
Citrobacter freundii
Cedecea neteri
Candidatus Tachikawaea
Candidatus Ishikawaella
Candidatus Hoaglandella
Candidatus Gullanella
Candidatus Doolittlea
Blochmannia endosymbiont
Taxon
0
500
1000
1500
Position along marker gene

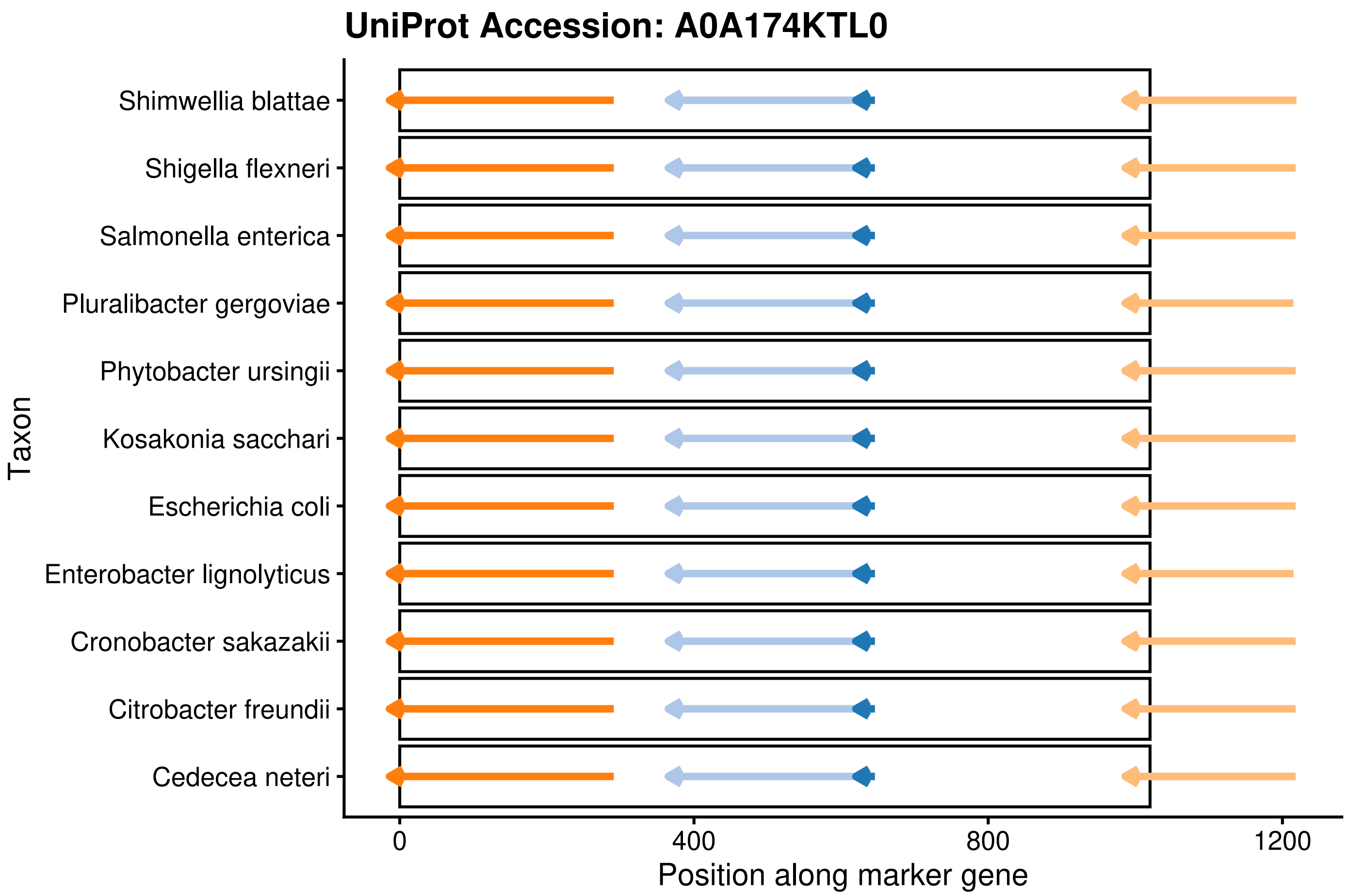
UniProt Accession: A0A174KTL0
Shimwellia blattae
Shigella flexneri
Salmonella enterica
Pluralibacter gergoviae
Phytobacter ursingii
Kosakonia sacchari
Escherichia coli
Enterobacter lignolyticus
Cronobacter sakazakii
Citrobacter freundii
Cedecea neteri
Taxon
0
400
800
1200
Position along marker gene

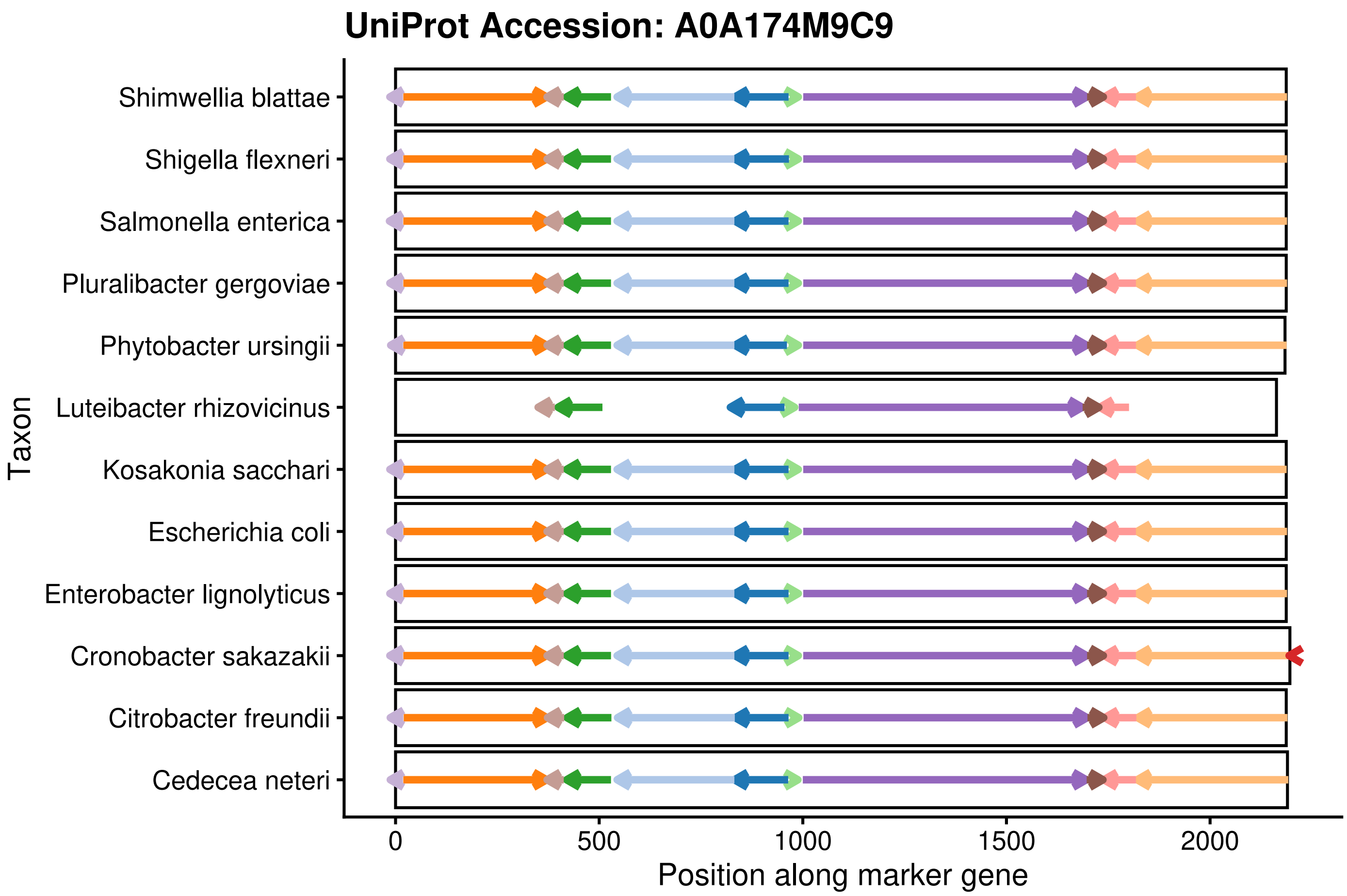
UniProt Accession: A0A174M9C9
Shimwellia blattae
Shigella flexneri
Salmonella enterica
Pluralibacter gergoviae
Phytobacter ursingii
Luteibacter rhizovicinus
Kosakonia sacchari
Escherichia coli
Enterobacter lignolyticus
Cronobacter sakazakii
Citrobacter freundii
Cedecea neteri
Taxon
0
500
1000
1500
2000
Position along marker gene

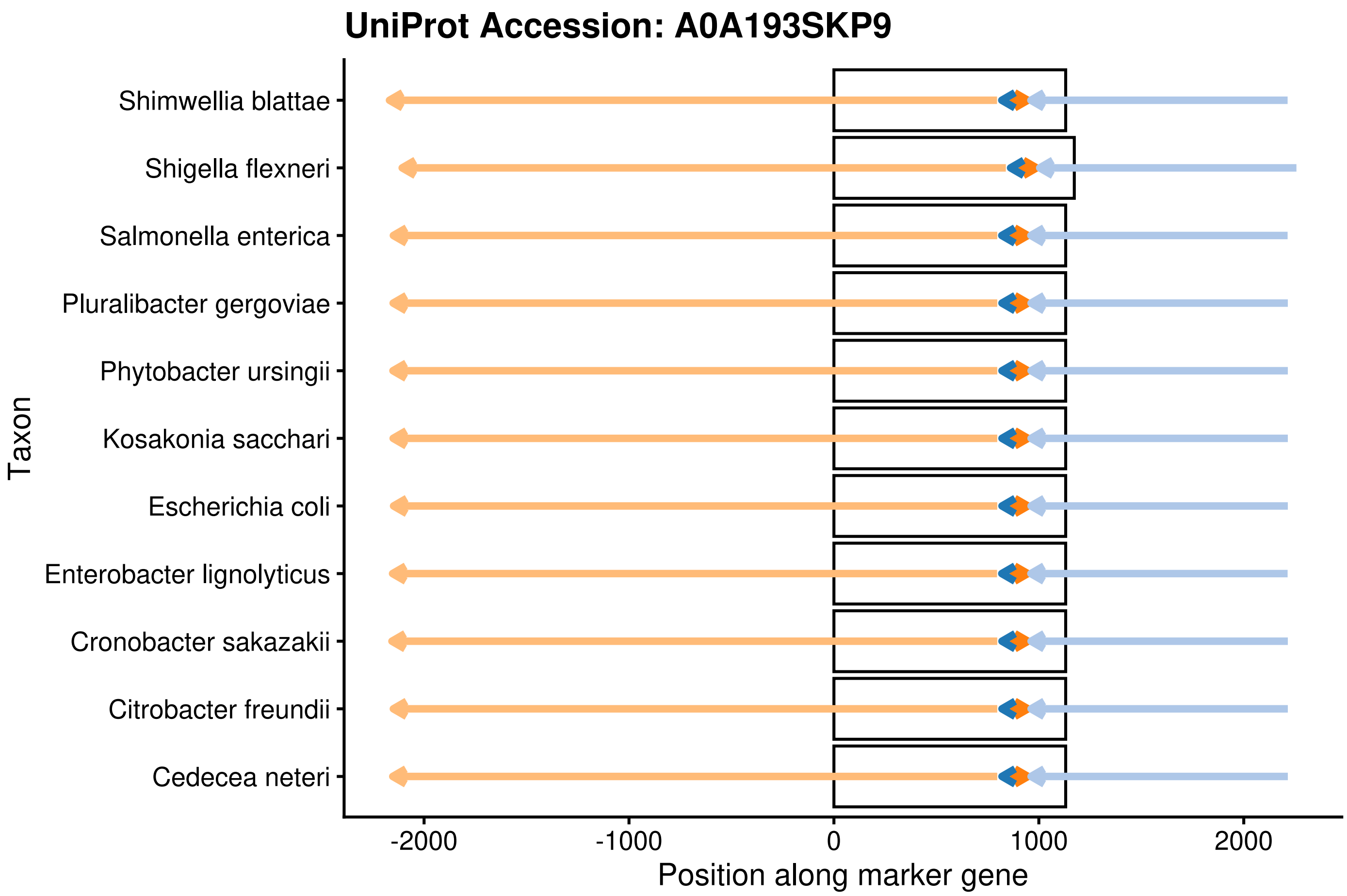
UniProt Accession: A0A193SKP9
Shimwellia blattae
Shigella flexneri
Salmonella enterica
Pluralibacter gergoviae
Phytobacter ursingii
Kosakonia sacchari
Escherichia coli
Enterobacter lignolyticus
Cronobacter sakazakii
Citrobacter freundii
Cedecea neteri
Taxon
-2000
-1000
0
1000
2000
Position along marker gene

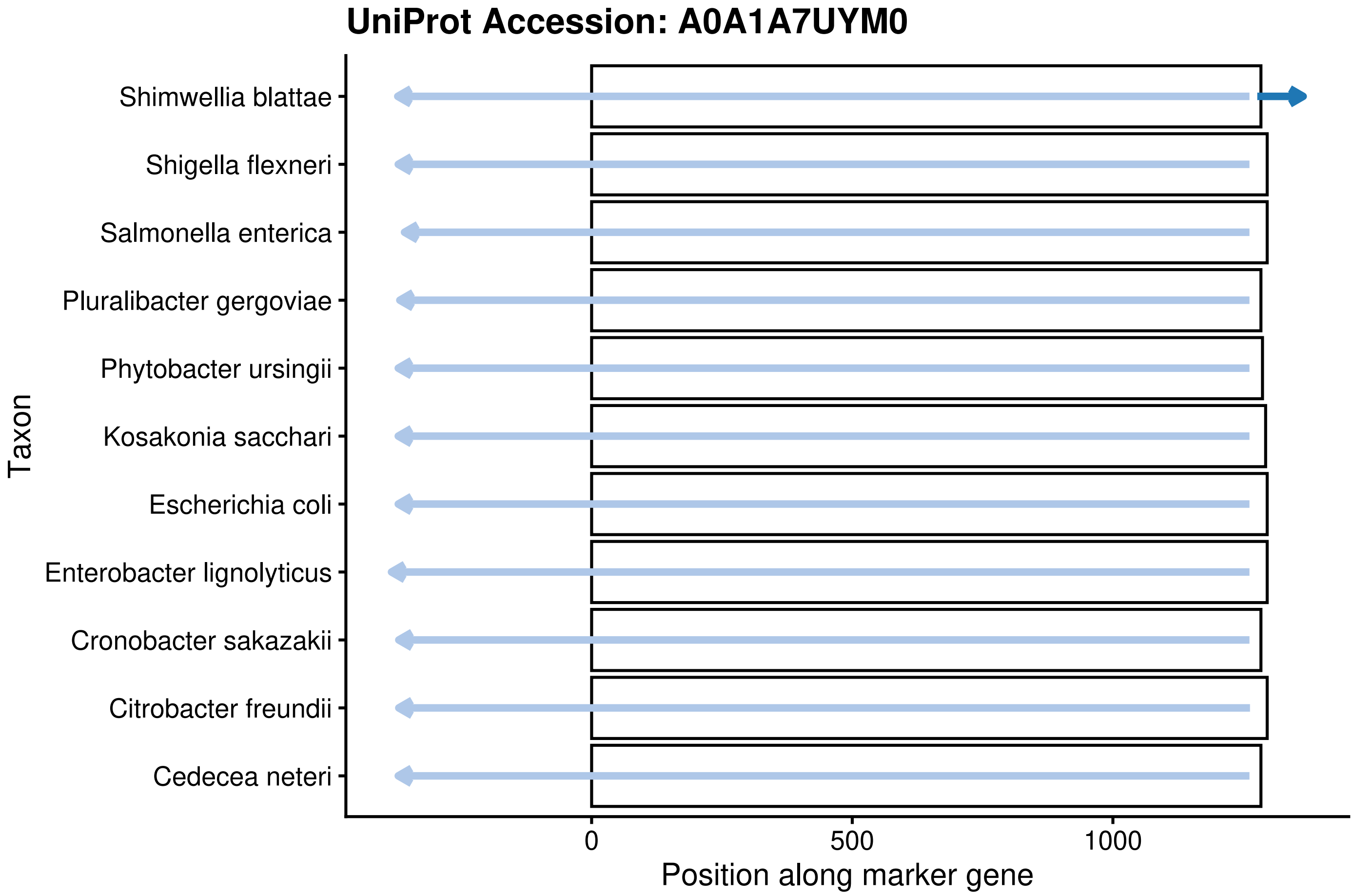
UniProt Accession: A0A1A7UYM0
Taxon
Shimwellia blattae
Shigella flexneri
Salmonella enterica
Pluralibacter gergoviae
Phytobacter ursingii
Kosakonia sacchari
Escherichia coli
Enterobacter lignolyticus
Cronobacter sakazakii
Citrobacter freundii
Cedecea neteri
0
500
1000
Position along marker gene

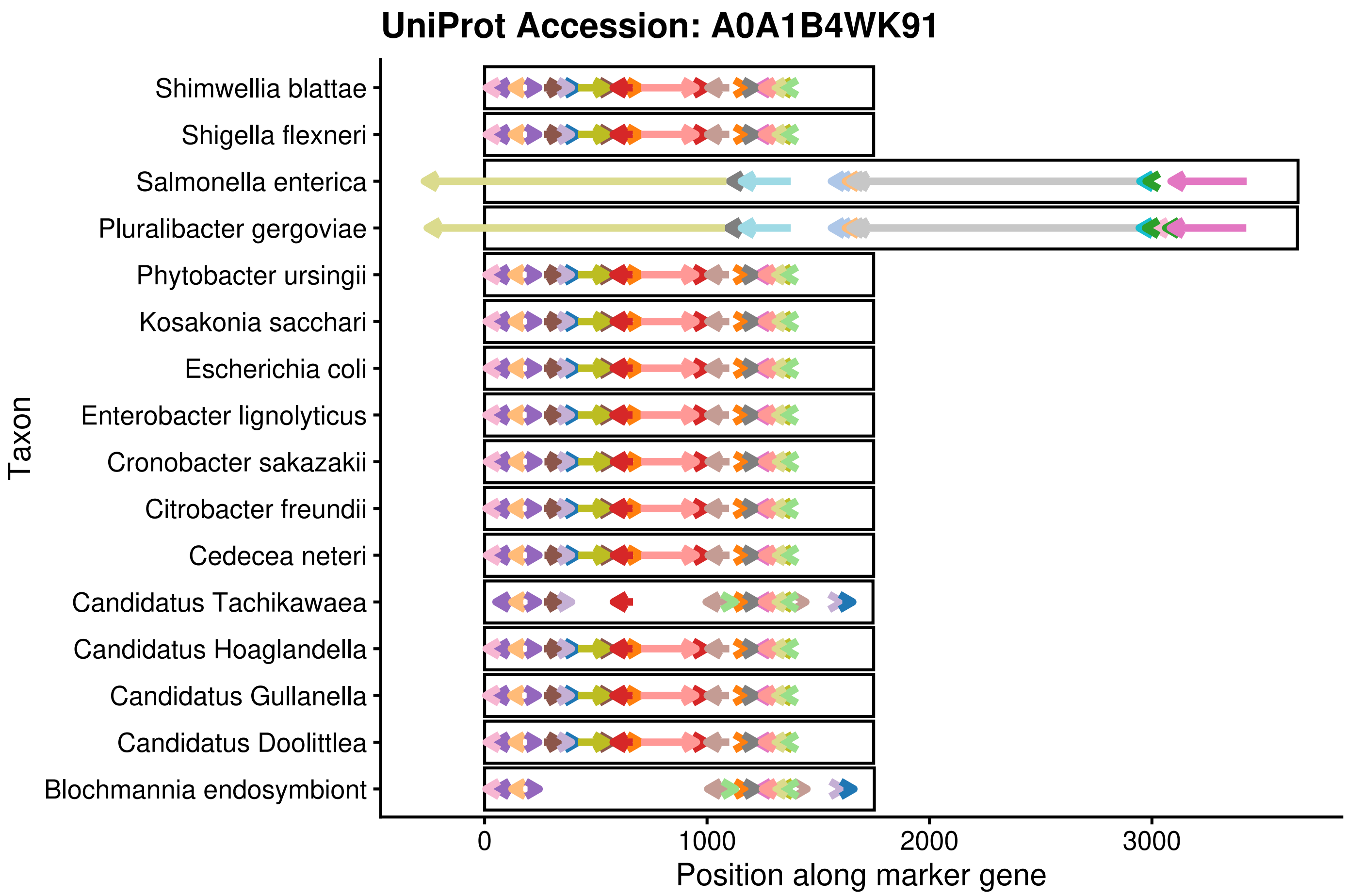

UniProt Accession: A0A1B4WK91
Shimwellia blattae
Shigella flexneri
Salmonella enterica
Pluralibacter gergoviae
Phytobacter ursingii
Kosakonia sacchari
Escherichia coli
Enterobacter lignolyticus
Cronobacter sakazakii
Citrobacter freundii
Cedecea neteri
Candidatus Tachikawaea
Candidatus Hoaglandella
Candidatus Gullanella
Candidatus Doolittlea
Blochmannia endosymbiont
Taxon
0
1000
2000
3000
Position along marker gene

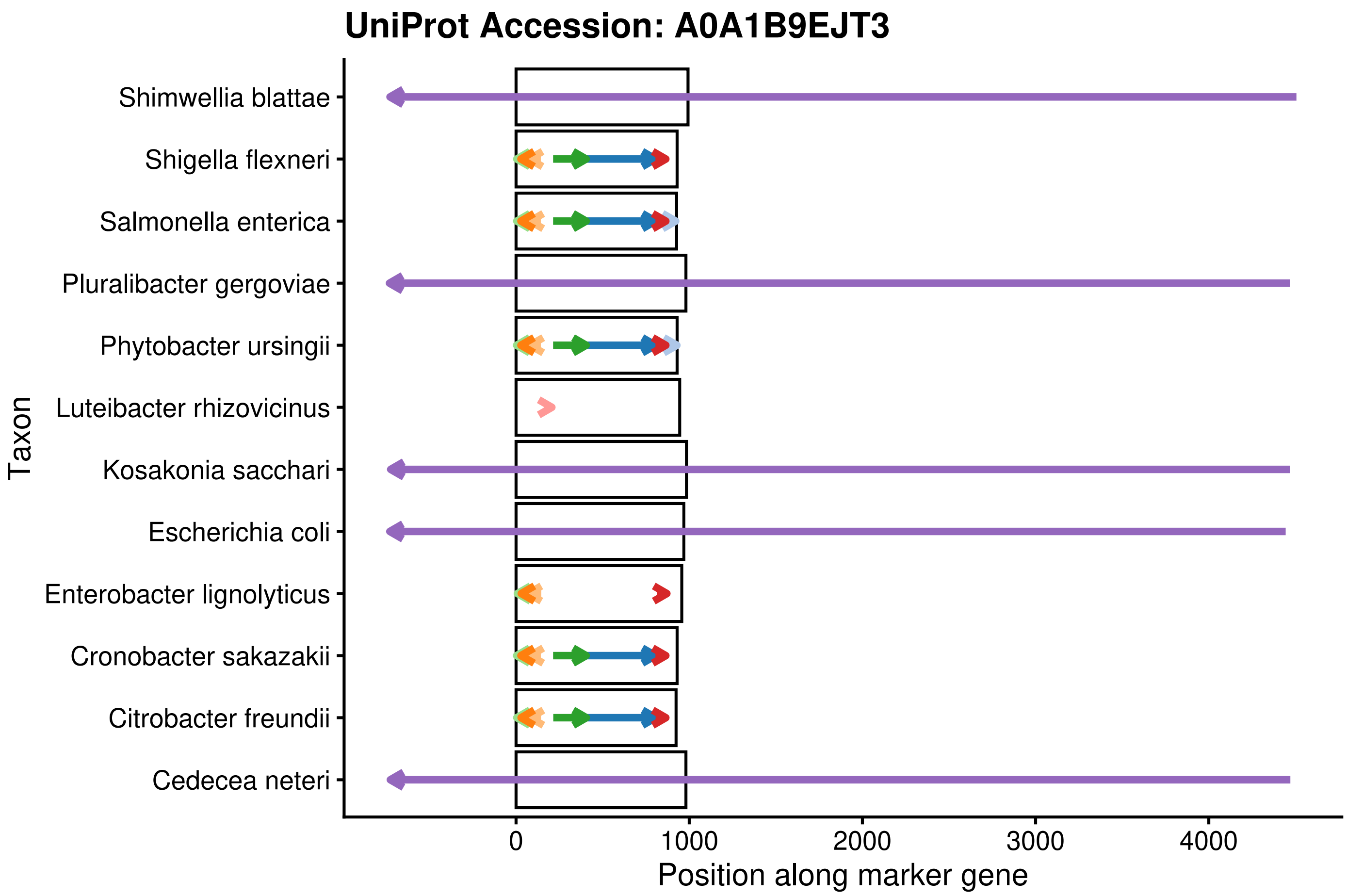

UniProt Accession: A0A1B9EJT3
Shimwellia blattae
Shigella flexneri
Salmonella enterica
Pluralibacter gergoviae
Phytobacter ursingii
Luteibacter rhizovicinus
Kosakonia sacchari
Escherichia coli
Enterobacter lignolyticus
Cronobacter sakazakii
Citrobacter freundii
Cedecea neteri
Taxon
0
1000
2000
3000
4000
Position along marker gene

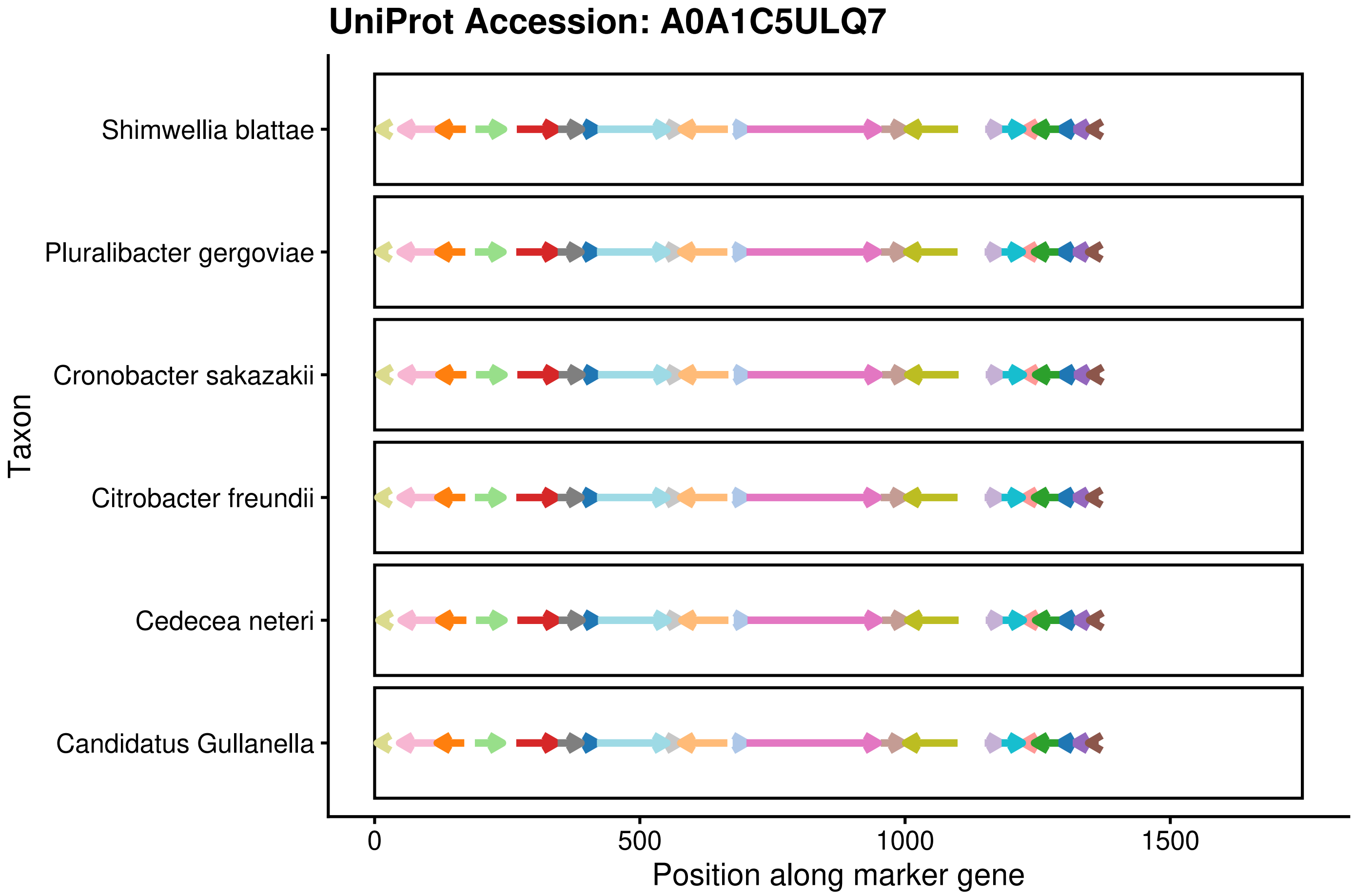

UniProt Accession: A0A1C5ULQ7
Taxon
Shimwellia blattae
Pluralibacter gergoviae
Cronobacter sakazakii
Citrobacter freundii
Cedecea neteri
Candidatus Gullanella
0
500
1000
1500
Position along marker gene

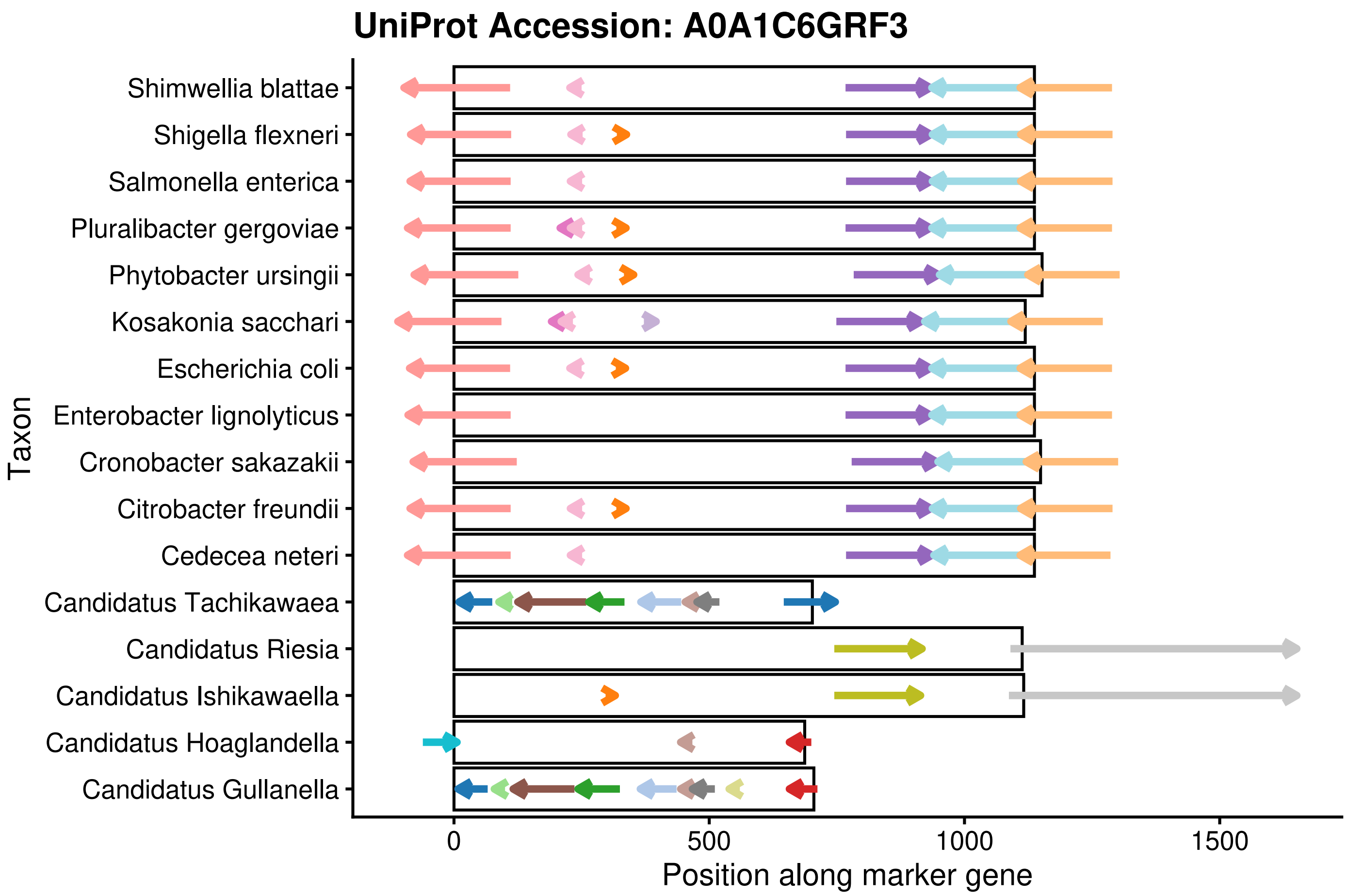

UniProt Accession: A0A1C6GRF3
Taxon
Shimwellia blattae
Shigella flexneri
Salmonella enterica
Pluralibacter gergoviae
Phytobacter ursingii
Kosakonia sacchari
Escherichia coli
Enterobacter lignolyticus
Cronobacter sakazakii
Citrobacter freundii
Cedecea neteri
Candidatus Tachikawaea
Candidatus Riesia
Candidatus Ishikawaella
Candidatus Hoaglandella
Candidatus Gullanella
0
500
1000
1500
Position along marker gene

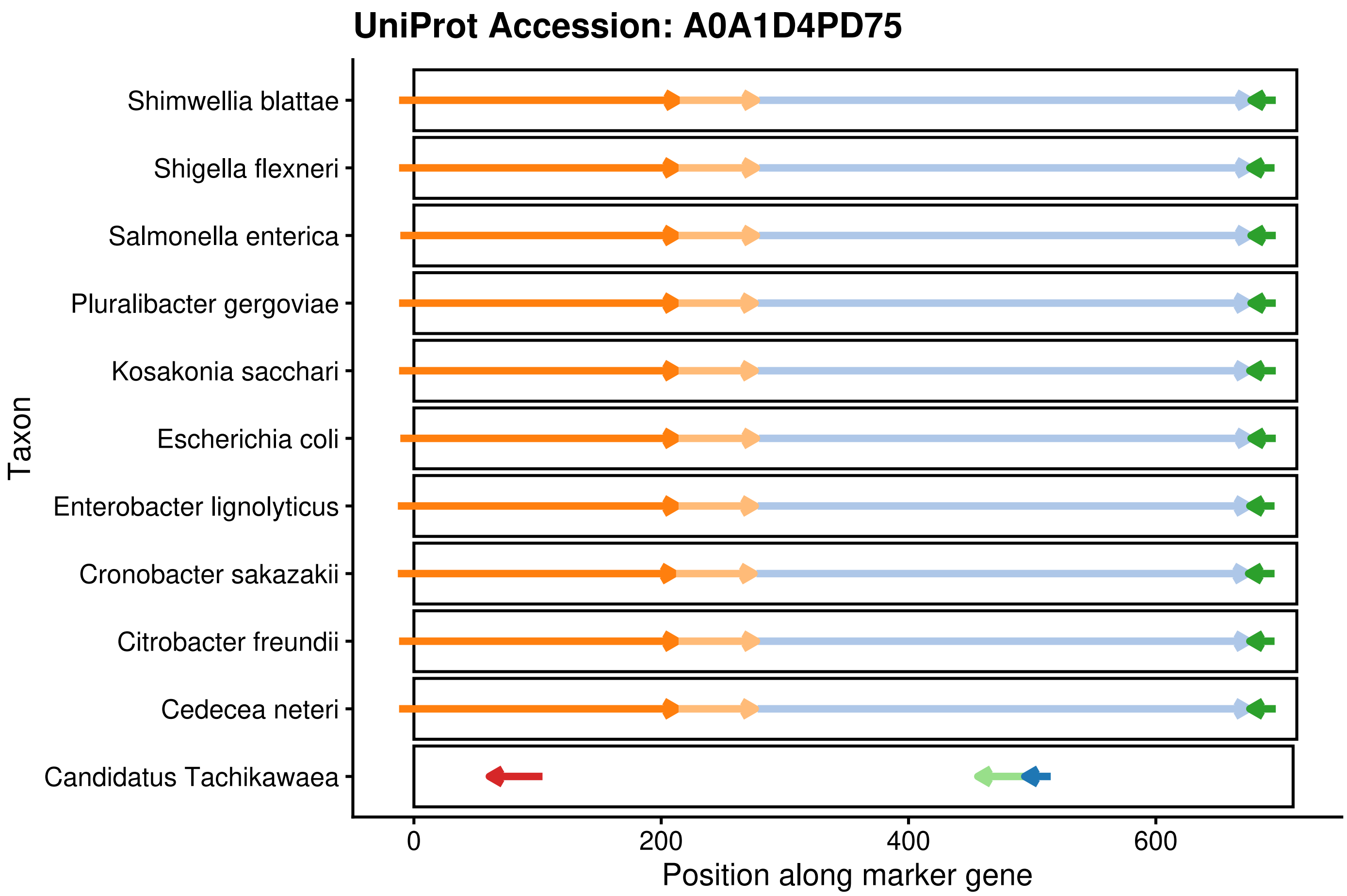
UniProt Accession: A0A1D4PD75
Taxon
Shimwellia blattae
Shigella flexneri
Salmonella enterica
Pluralibacter gergoviae
Kosakonia sacchari
Escherichia coli
Enterobacter lignolyticus
Cronobacter sakazakii
Citrobacter freundii
Cedecea neteri
Candidatus Tachikawaea
0
200
400
600
Position along marker gene

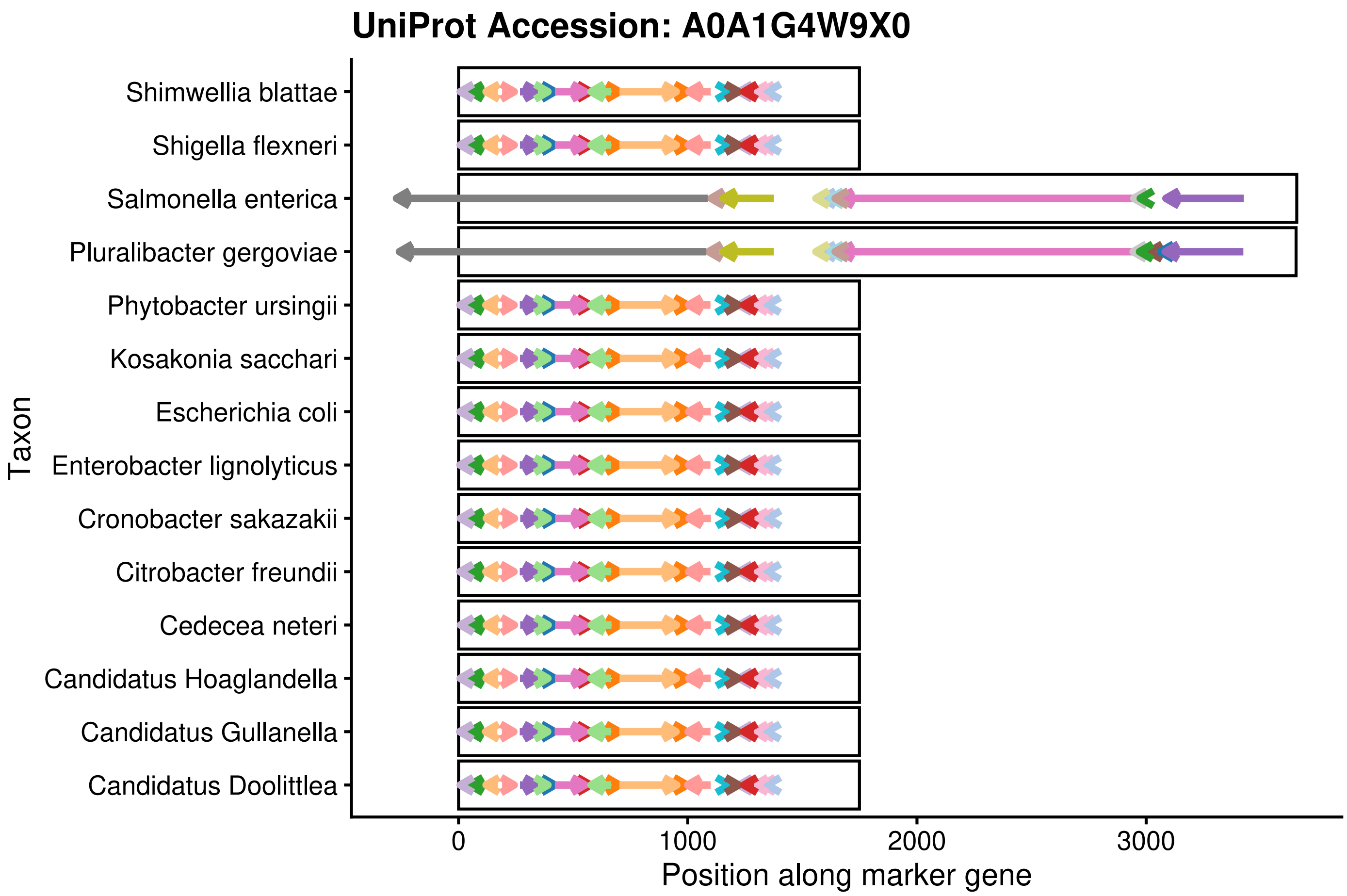

UniProt Accession: A0A1G4W9X0
Taxon
Shimwellia blattae
Shigella flexneri
Salmonella enterica
Pluralibacter gergoviae
Phytobacter ursingii
Kosakonia sacchari
Escherichia coli
Enterobacter lignolyticus
Cronobacter sakazakii
Citrobacter freundii
Cedecea neteri
Candidatus Hoaglandella
Candidatus Gullanella
Candidatus Doolittlea
0
1000
2000
3000
Position along marker gene

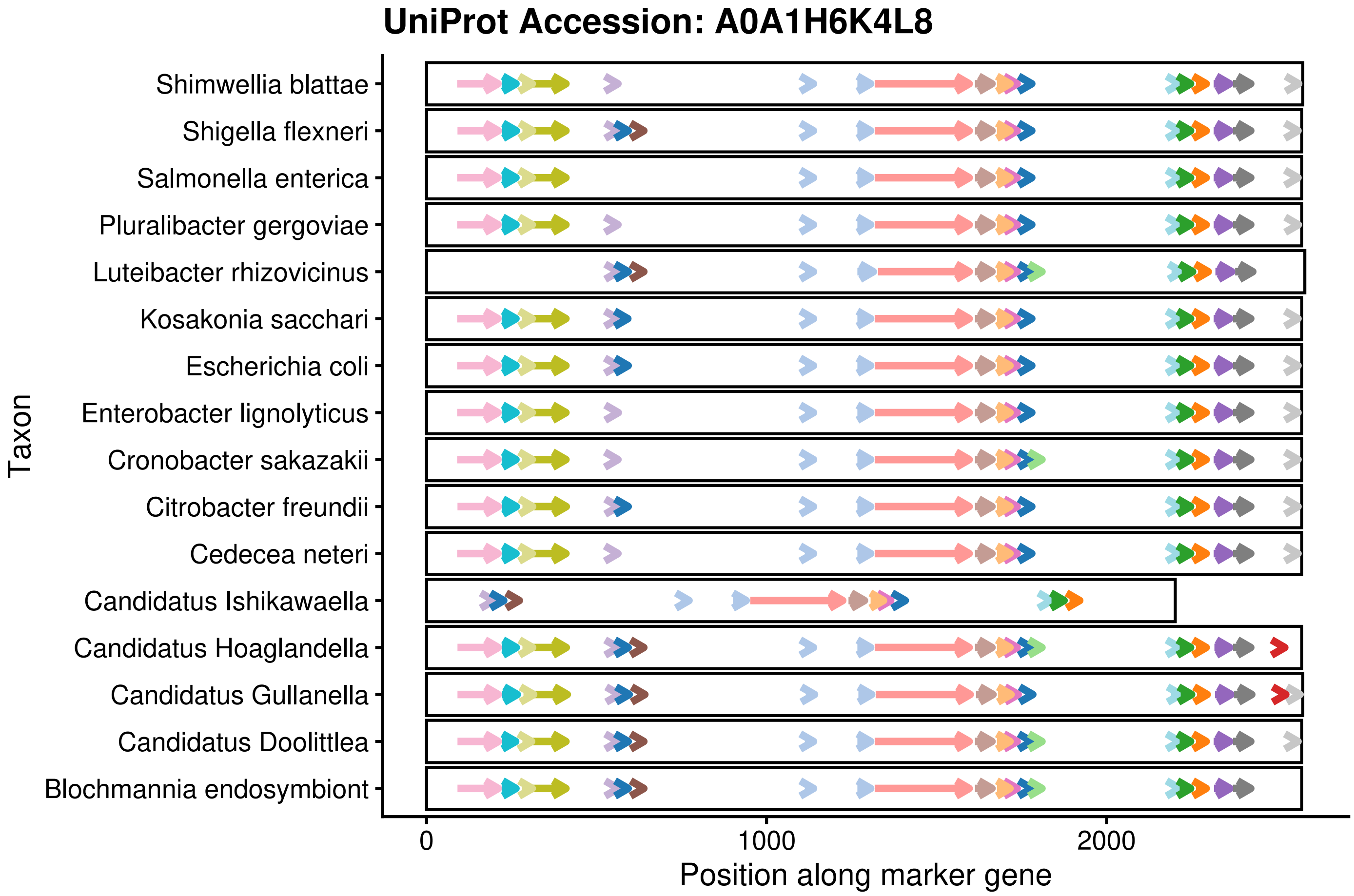
UniProt Accession: A0A1H6K4L8
Taxon
Shimwellia blattae
Shigella flexneri
Salmonella enterica
Pluralibacter gergoviae
Luteibacter rhizovicinus
Kosakonia sacchari
Escherichia coli
Enterobacter lignolyticus
Cronobacter sakazakii
Citrobacter freundii
Cedecea neteri
Candidatus Ishikawaella
Candidatus Hoaglandella
Candidatus Gullanella
Candidatus Doolittlea
Blochmannia endosymbiont
0
1000
2000
Position along marker gene

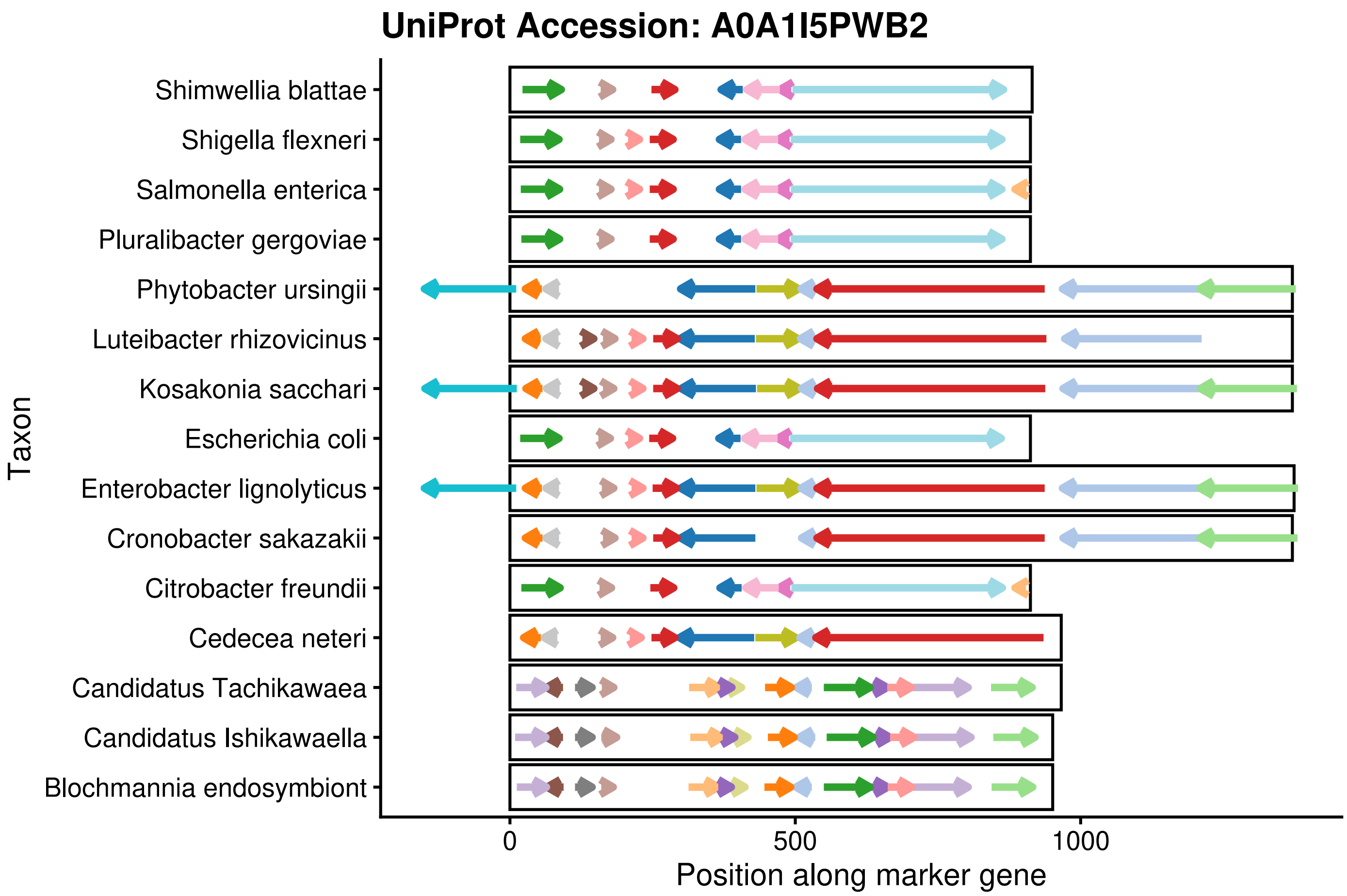

UniProt Accession: A0A1I5PWB2
Shimwellia blattae
Shigella flexneri
Salmonella enterica
Pluralibacter gergoviae
Phytobacter ursingii
Luteibacter rhizovicinus
Kosakonia sacchari
Escherichia coli
Enterobacter lignolyticus
Cronobacter sakazakii
Citrobacter freundii
Cedecea neteri
Candidatus Tachikawaea
Candidatus Ishikawaella
Blochmannia endosymbiont
Taxon
0
500
1000
Position along marker gene

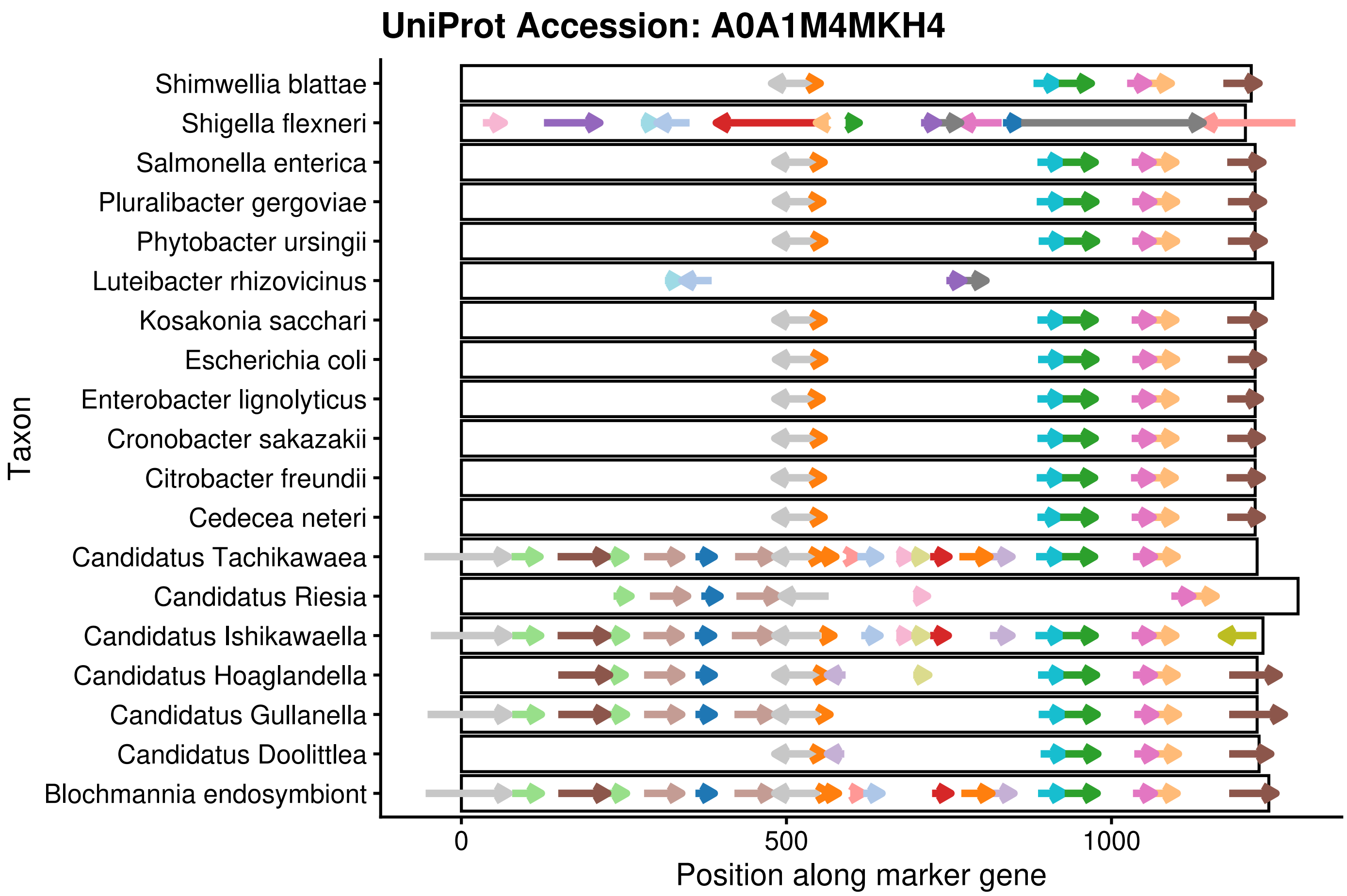

UniProt Accession: A0A1M4MKH4
Shimwellia blattae
Shigella flexneri
Salmonella enterica
Pluralibacter gergoviae
Phytobacter ursingii
Luteibacter rhizovicinus
Kosakonia sacchari
Escherichia coli
Enterobacter lignolyticus
Cronobacter sakazakii
Citrobacter freundii
Cedecea neteri
Candidatus Tachikawaea
Candidatus Riesia
Candidatus Ishikawaella
Candidatus Hoaglandella
Candidatus Gullanella
Candidatus Doolittlea
Blochmannia endosymbiont
Taxon
0
500
1000
Position along marker gene

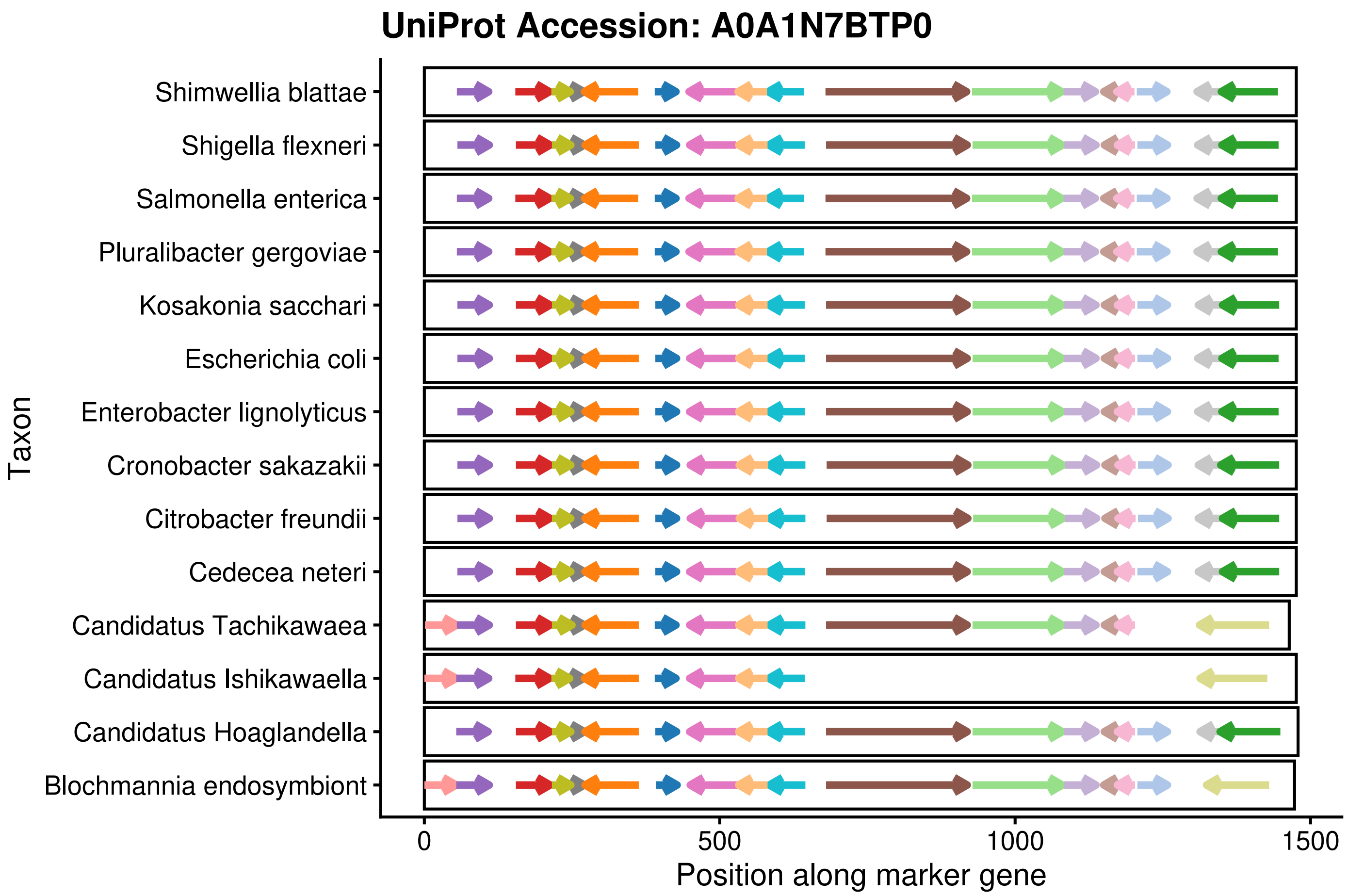
UniProt Accession: A0A1N7BTP0
Taxon
Shimwellia blattae
Shigella flexneri
Salmonella enterica
Pluralibacter gergoviae
Kosakonia sacchari
Escherichia coli
Enterobacter lignolyticus
Cronobacter sakazakii
Citrobacter freundii
Cedecea neteri
Candidatus Tachikawaea
Candidatus Ishikawaella
Candidatus Hoaglandella
Blochmannia endosymbiont
0
500
1000
1500
Position along marker gene

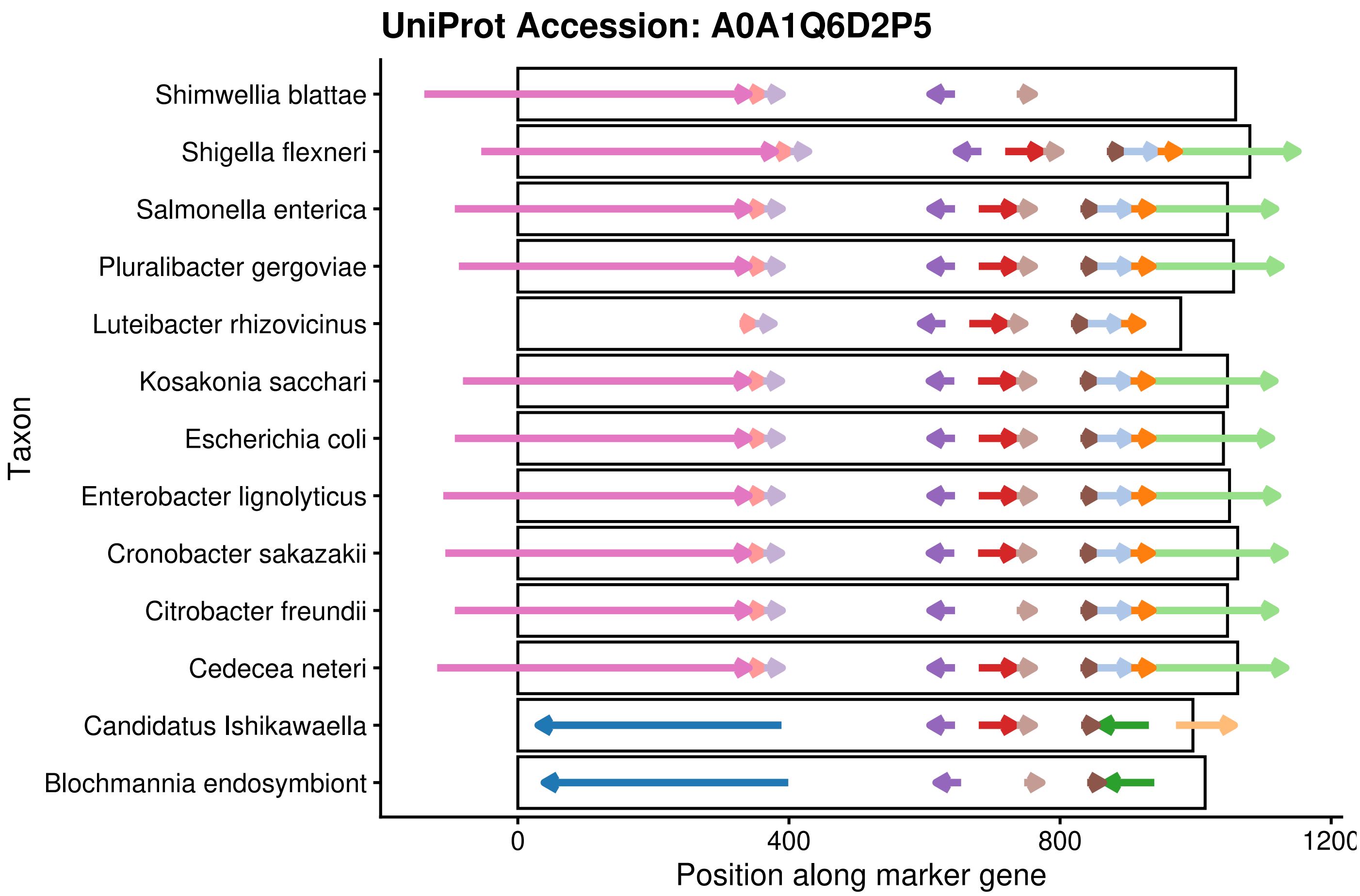
UniProt Accession: A0A1Q6D2P5
Shimwellia blattae
Shigella flexneri
Salmonella enterica
Pluralibacter gergoviae
Luteibacter rhizovicinus
Kosakonia sacchari
Escherichia coli
Enterobacter lignolyticus
Cronobacter sakazakii
Citrobacter freundii
Cedecea neteri
Candidatus Ishikawaella
Blochmannia endosymbiont
Taxon
0
400
800
1200
Position along marker gene

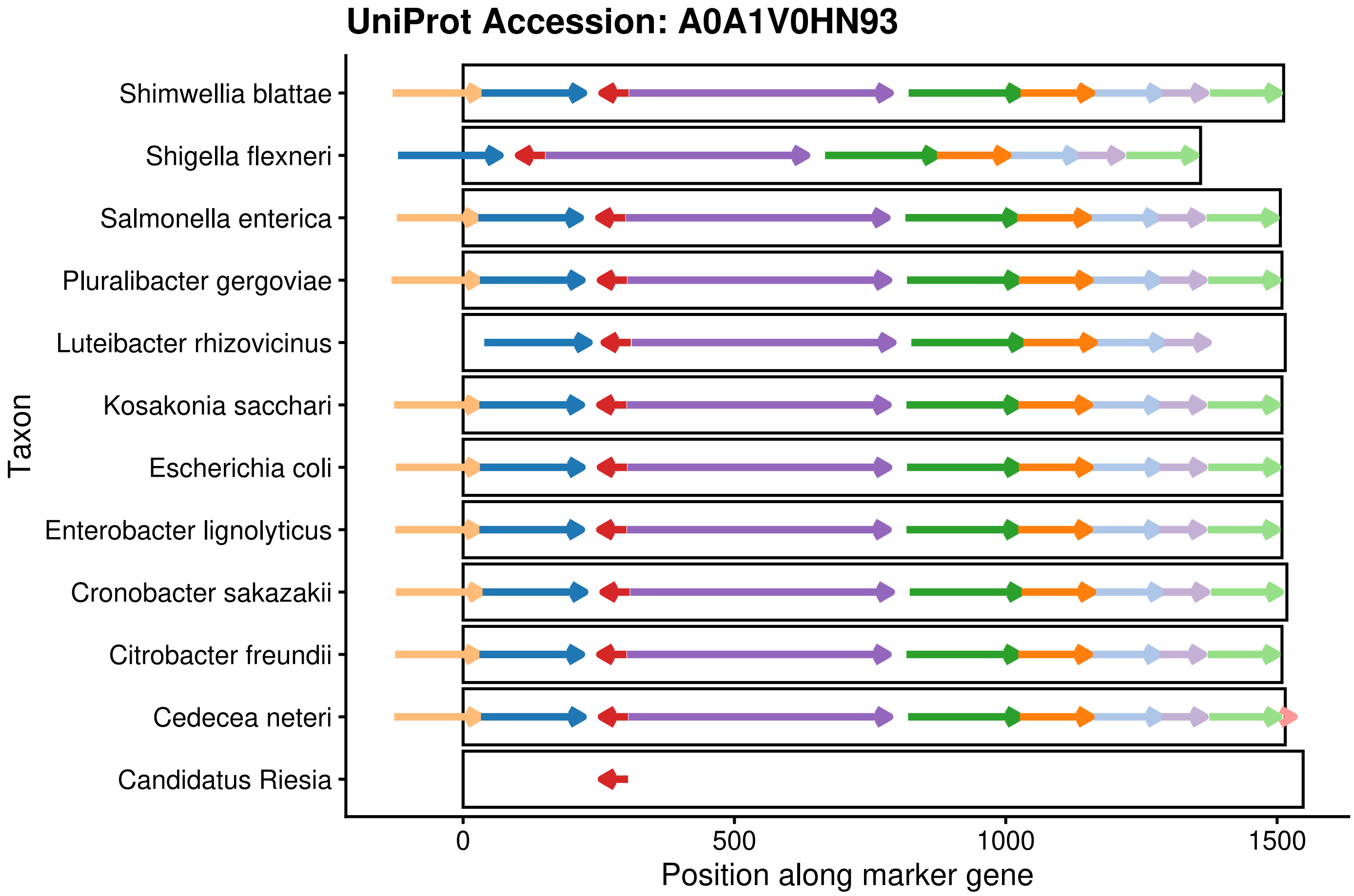
UniProt Accession: A0A1V0HN93
Taxon
Shimwellia blattae
Shigella flexneri
Salmonella enterica
Pluralibacter gergoviae
Luteibacter rhizovicinus
Kosakonia sacchari
Escherichia coli
Enterobacter lignolyticus
Cronobacter sakazakii
Citrobacter freundii
Cedecea neteri
Candidatus Riesia
0
500
1000
1500
Position along marker gene

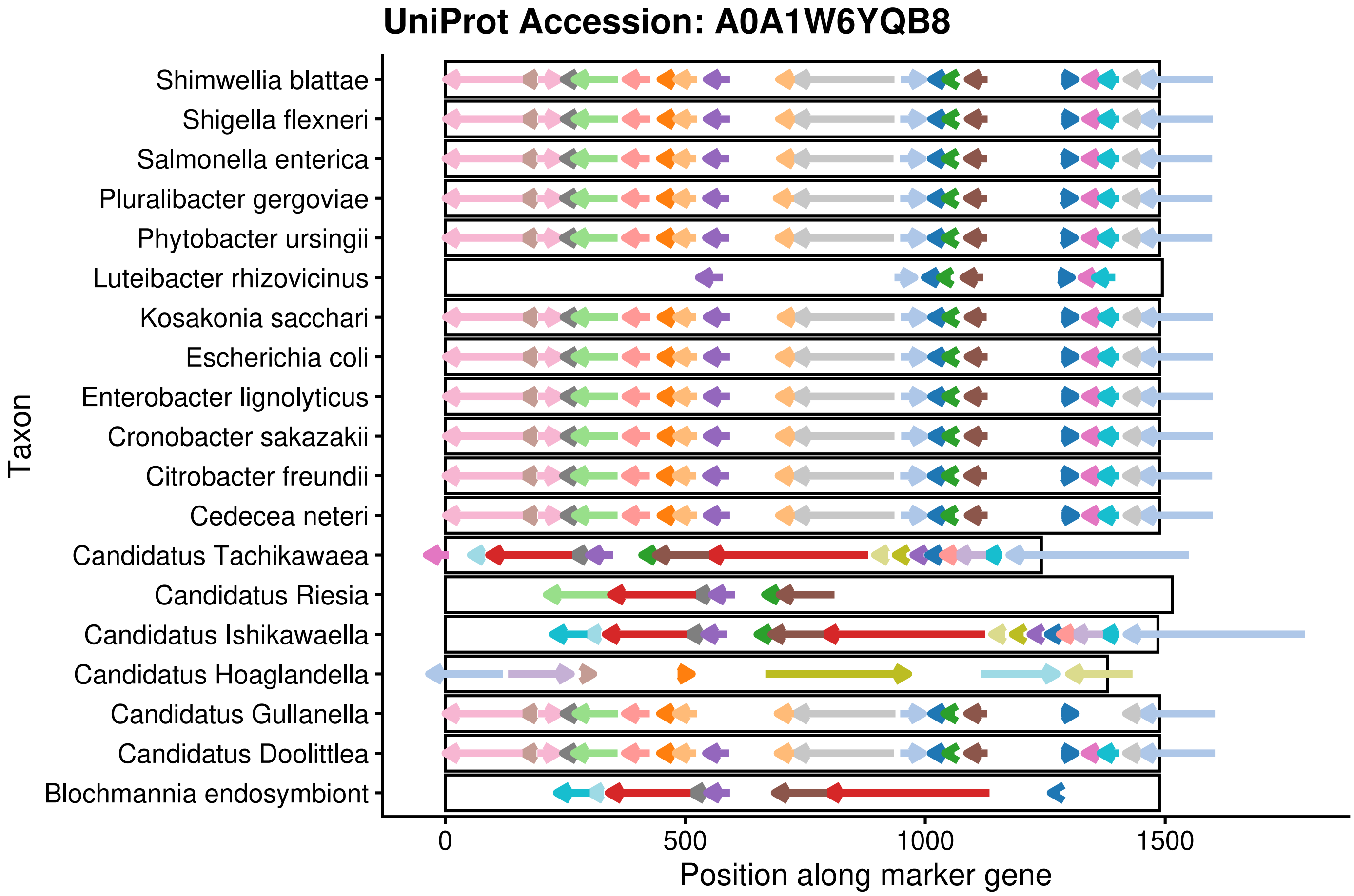
UniProt Accession: A0A1W6YQB8
Shimwellia blattae
Shigella flexneri
Salmonella enterica
Pluralibacter gergoviae
Phytobacter ursingii
Luteibacter rhizovicinus
Kosakonia sacchari
Escherichia coli
Enterobacter lignolyticus
Cronobacter sakazakii
Citrobacter freundii
Cedecea neteri
Candidatus Tachikawaea
Candidatus Riesia
Candidatus Ishikawaella
Candidatus Hoaglandella
Candidatus Gullanella
Candidatus Doolittlea
Blochmannia endosymbiont
Taxon
0
500
1000
1500
Position along marker gene

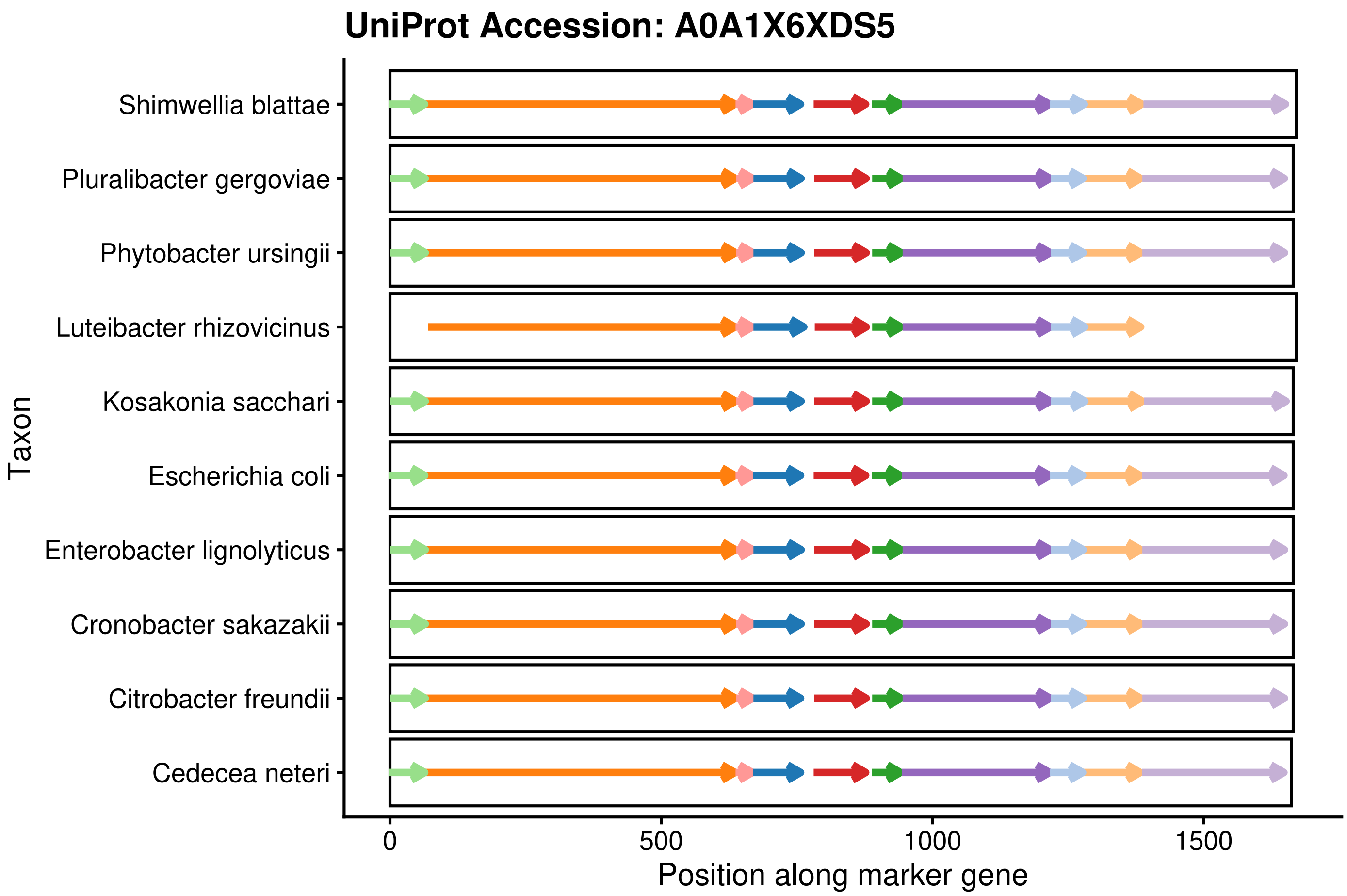

UniProt Accession: A0A1X6XDS5
Taxon
Shimwellia blattae
Pluralibacter gergoviae
Phytobacter ursingii
Luteibacter rhizovicinus
Kosakonia sacchari
Escherichia coli
Enterobacter lignolyticus
Cronobacter sakazakii
Citrobacter freundii
Cedecea neteri
0
500
1000
1500
Position along marker gene

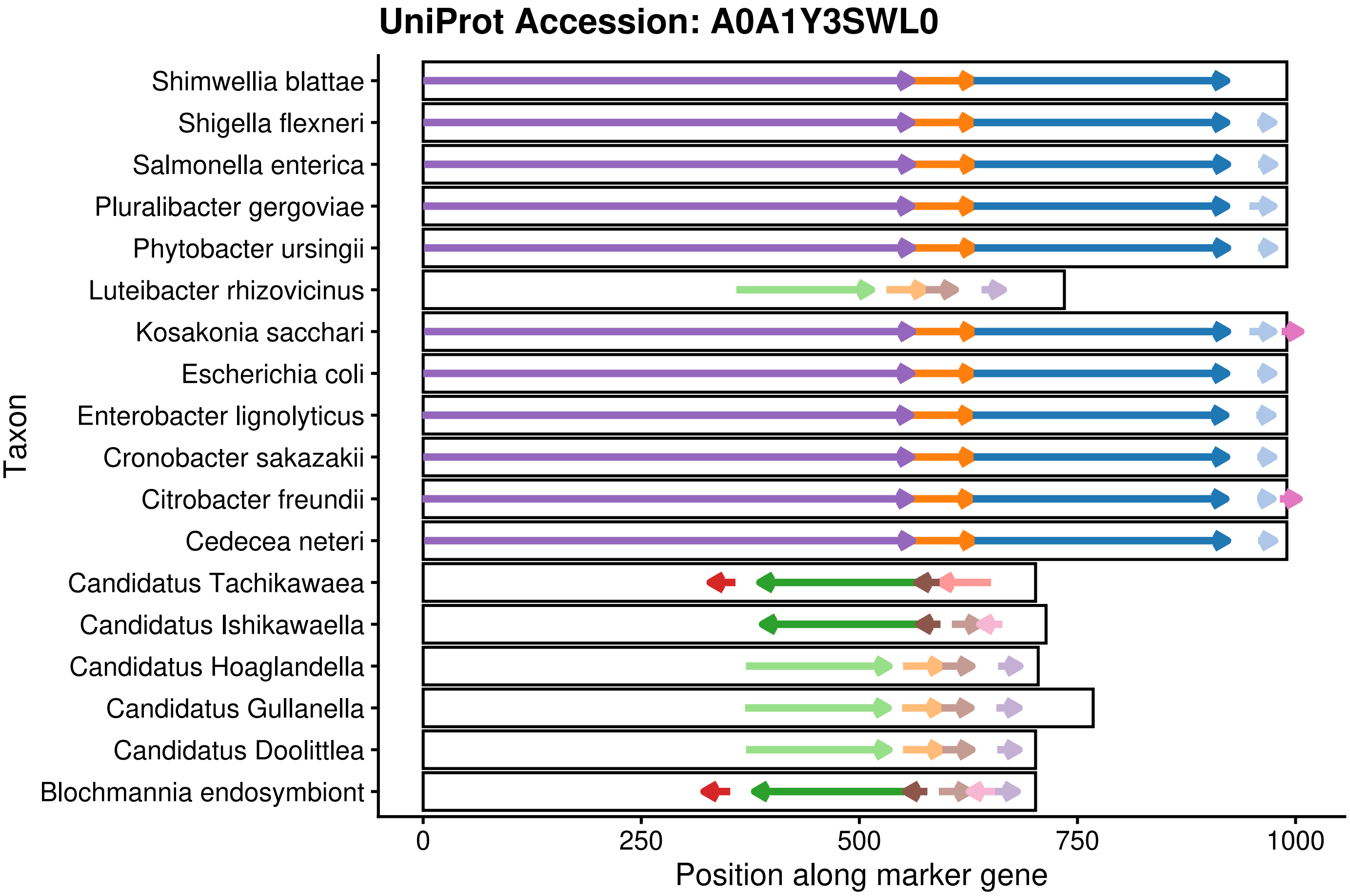

UniProt Accession: A0A1Y3SWL0
Shimwellia blattae
Shigella flexneri
Salmonella enterica
Pluralibacter gergoviae
Phytobacter ursingii
Luteibacter rhizovicinus
Kosakonia sacchari
Escherichia coli
Enterobacter lignolyticus
Cronobacter sakazakii
Citrobacter freundii
Cedecea neteri
Candidatus Tachikawaea
Candidatus Ishikawaella
Candidatus Hoaglandella
Candidatus Gullanella
Candidatus Doolittlea
Blochmannia endosymbiont
Taxon
0
250
500
750
1000
Position along marker gene

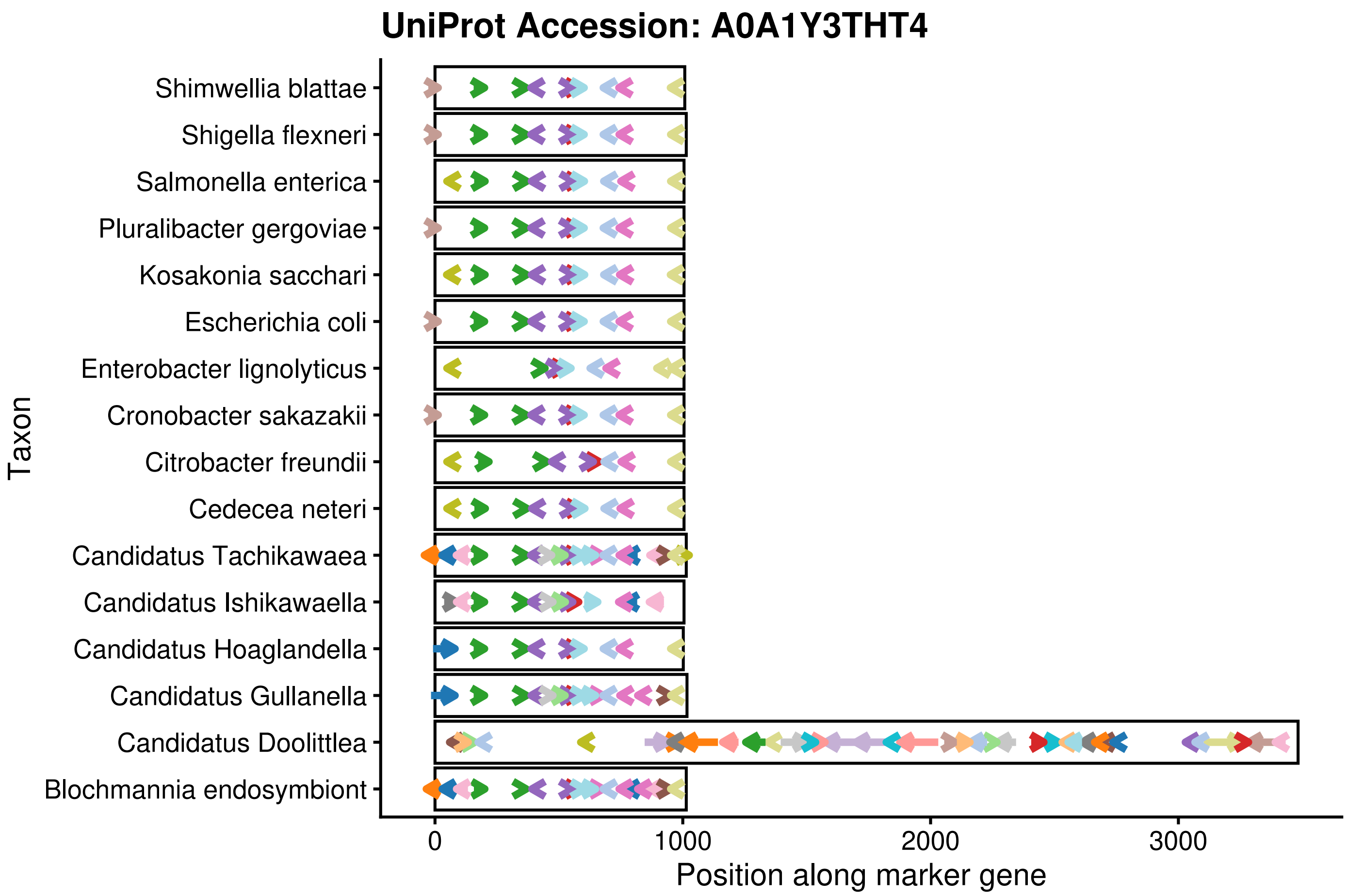
UniProt Accession: A0A1Y3THT4
Taxon
Shimwellia blattae
Shigella flexneri
Salmonella enterica
Pluralibacter gergoviae
Kosakonia sacchari
Escherichia coli
Enterobacter lignolyticus
Cronobacter sakazakii
Citrobacter freundii
Cedecea neteri
Candidatus Tachikawaea
Candidatus Ishikawaella
Candidatus Hoaglandella
Candidatus Gullanella
Candidatus Doolittlea
Blochmannia endosymbiont
0
1000
2000
3000
Position along marker gene

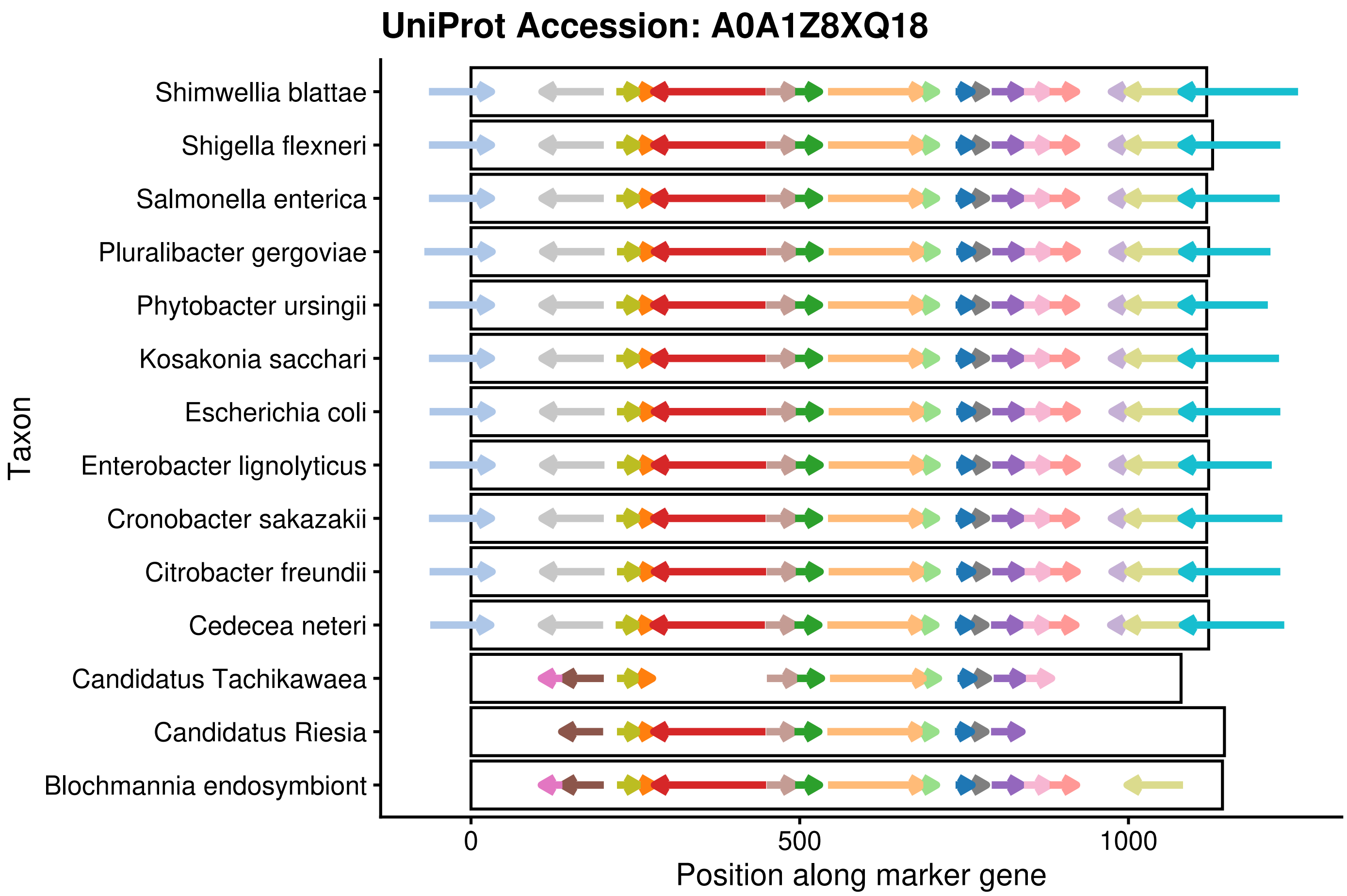
UniProt Accession: A0A1Z8XQ18
Shimwellia blattae
Shigella flexneri
Salmonella enterica
Pluralibacter gergoviae
Phytobacter ursingii
Kosakonia sacchari
Escherichia coli
Enterobacter lignolyticus
Cronobacter sakazakii
Citrobacter freundii
Cedecea neteri
Candidatus Tachikawaea
Candidatus Riesia
Blochmannia endosymbiont
Taxon
0
500
1000
Position along marker gene

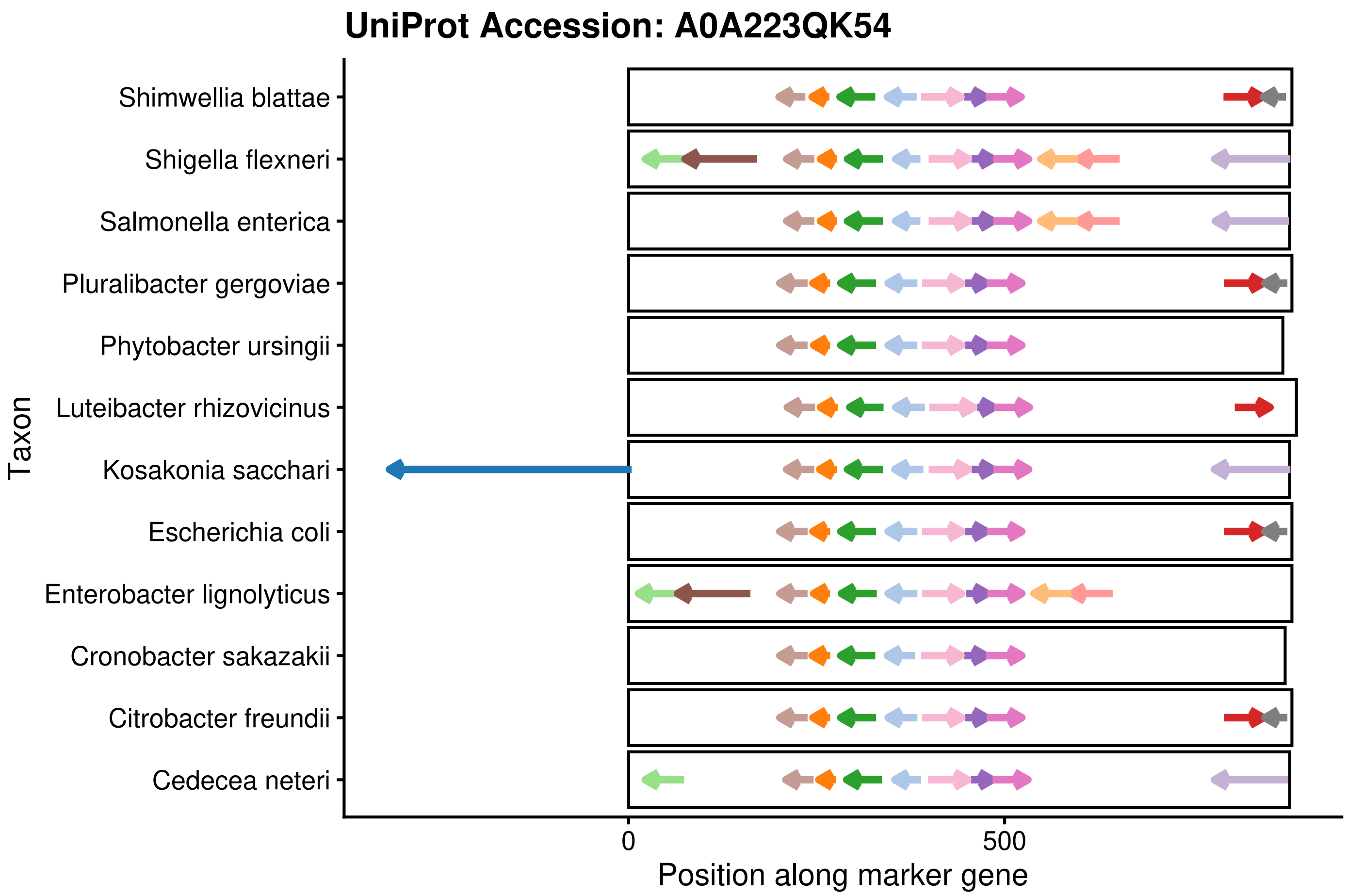

UniProt Accession: A0A223QK54
Taxon
Shimwellia blattae
Shigella flexneri
Salmonella enterica
Pluralibacter gergoviae
Phytobacter ursingii
Luteibacter rhizovicinus
Kosakonia sacchari
Escherichia coli
Enterobacter lignolyticus
Cronobacter sakazakii
Citrobacter freundii
Cedecea neteri
0
500
Position along marker gene

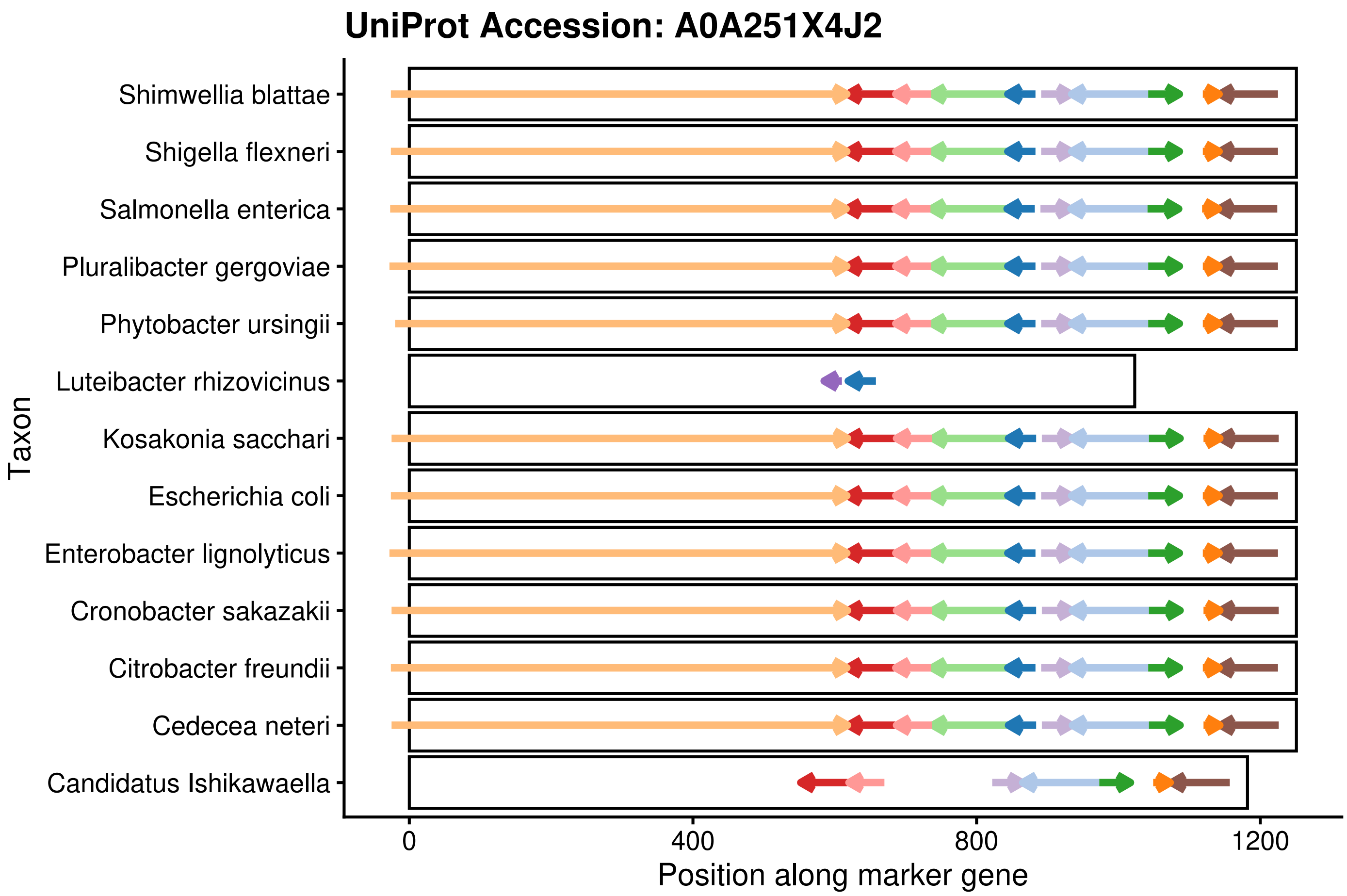
UniProt Accession: A0A251X4J2
Shimwellia blattae
Shigella flexneri
Salmonella enterica
Pluralibacter gergoviae
Phytobacter ursingii
Luteibacter rhizovicinus
Kosakonia sacchari
Escherichia coli
Enterobacter lignolyticus
Cronobacter sakazakii
Citrobacter freundii
Cedecea neteri
Candidatus Ishikawaella
Taxon
0
400
800
1200
Position along marker gene

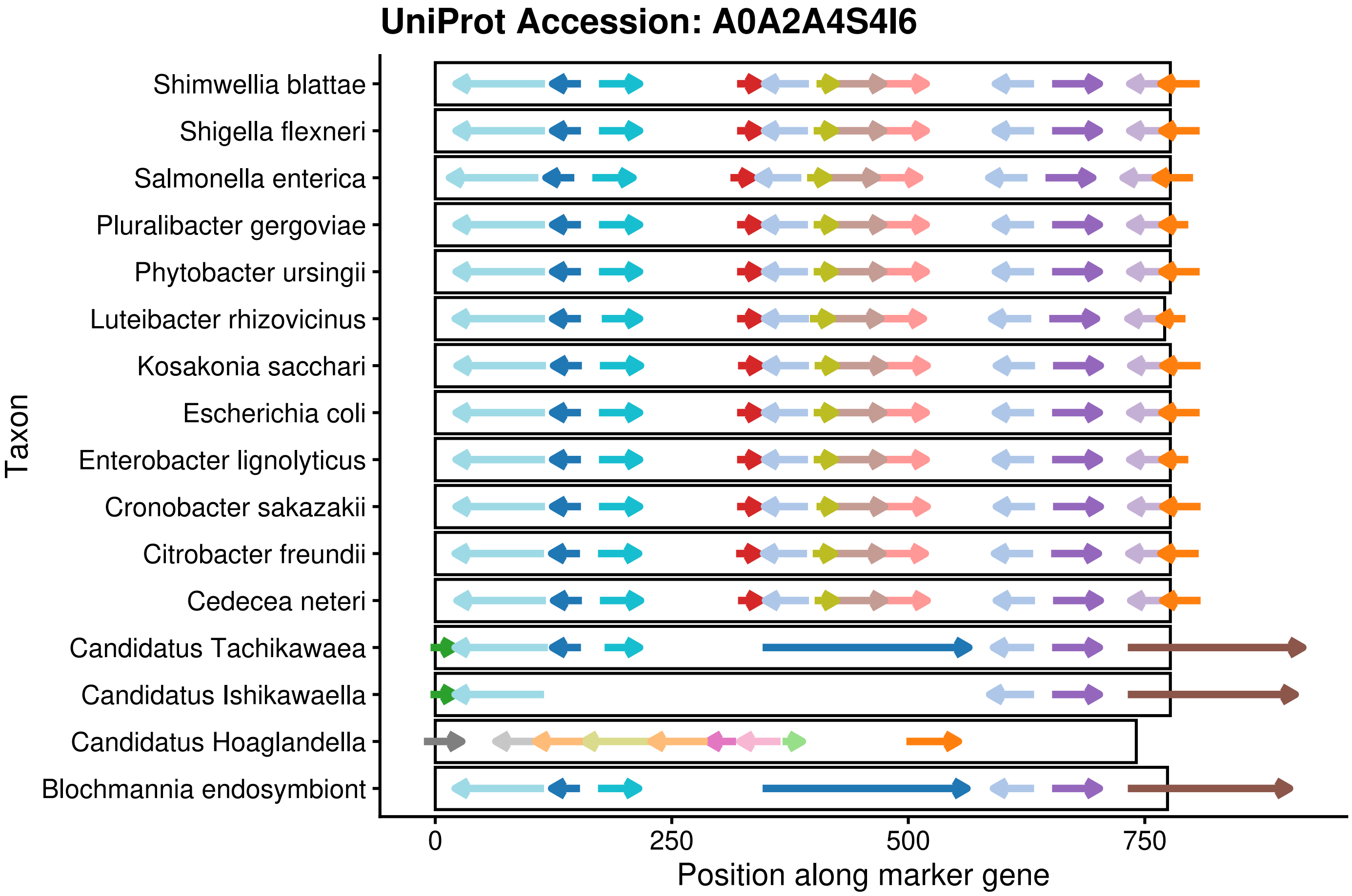

UniProt Accession: A0A2A4S4I6
Taxon
Shimwellia blattae
Shigella flexneri
Salmonella enterica
Pluralibacter gergoviae
Phytobacter ursingii
Luteibacter rhizovicinus
Kosakonia sacchari
Escherichia coli
Enterobacter lignolyticus
Cronobacter sakazakii
Citrobacter freundii
Cedecea neteri
Candidatus Tachikawaea
Candidatus Ishikawaella
Candidatus Hoaglandella
Blochmannia endosymbiont
0
250
500
750
Position along marker gene

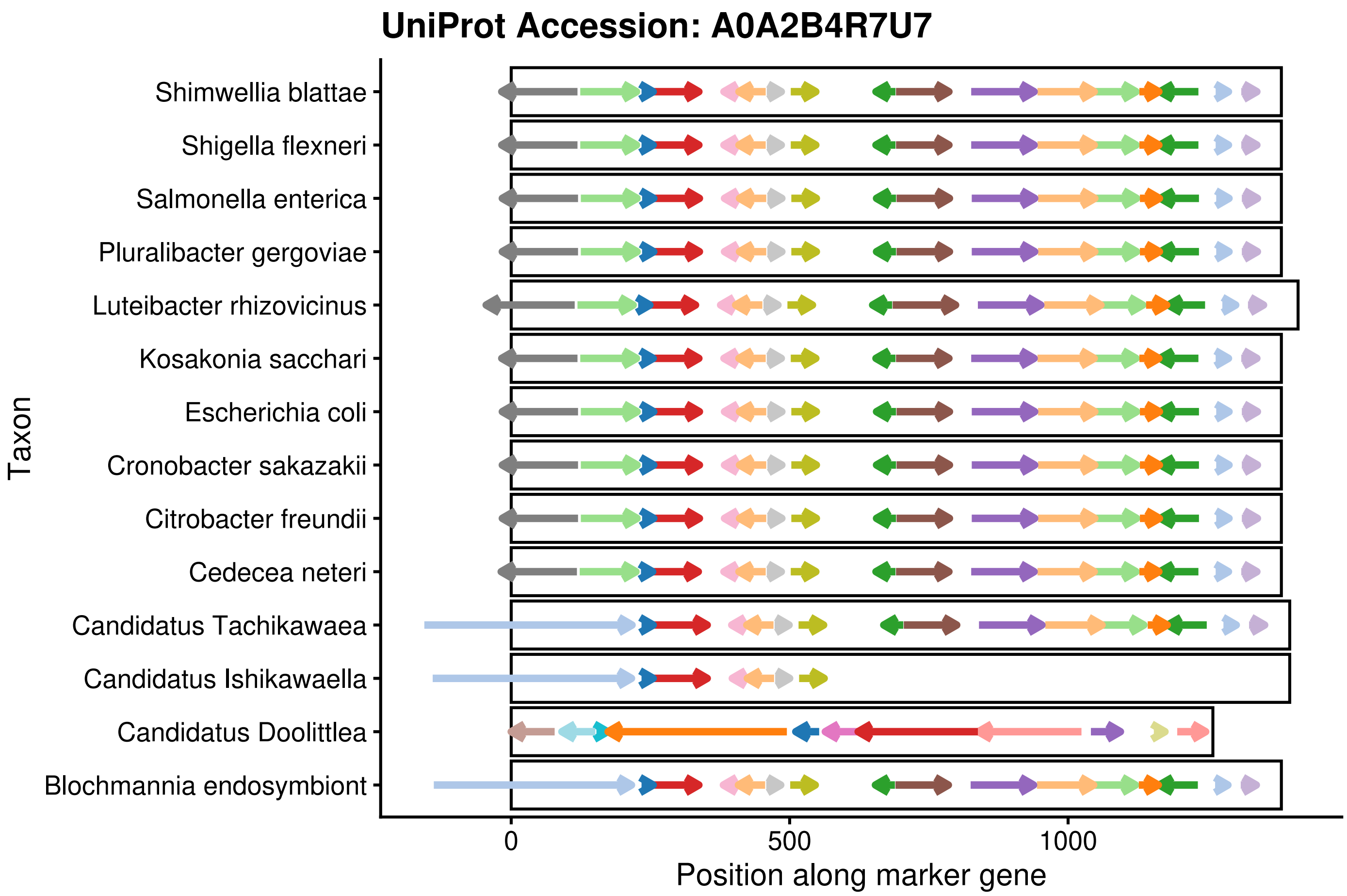

UniProt Accession: A0A2B4R7U7
Shimwellia blattae
Shigella flexneri
Salmonella enterica
Pluralibacter gergoviae
Luteibacter rhizovicinus
Kosakonia sacchari
Escherichia coli
Cronobacter sakazakii
Citrobacter freundii
Cedecea neteri
Candidatus Tachikawaea
Candidatus Ishikawaella
Candidatus Doolittlea
Blochmannia endosymbiont
Taxon
0
500
1000
Position along marker gene

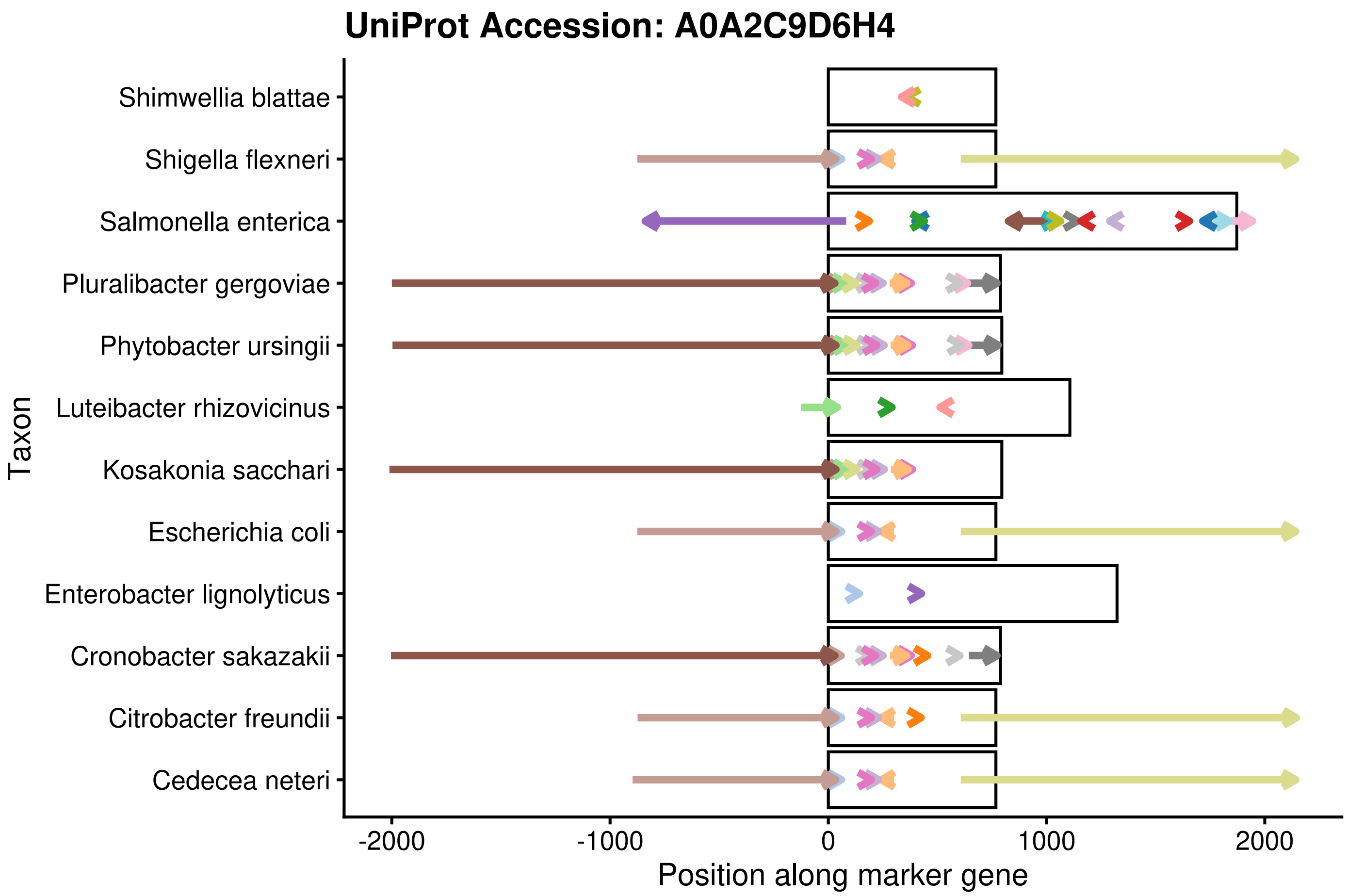
UniProt Accession: A0A2C9D6H4
Shimwellia blattae
Shigella flexneri
Salmonella enterica
Pluralibacter gergoviae
Phytobacter ursingii
Luteibacter rhizovicinus
Kosakonia sacchari
Escherichia coli
Enterobacter lignolyticus
Cronobacter sakazakii
Citrobacter freundii
Cedecea neteri
Taxon
-2000
-1000
0
1000
2000
Position along marker gene

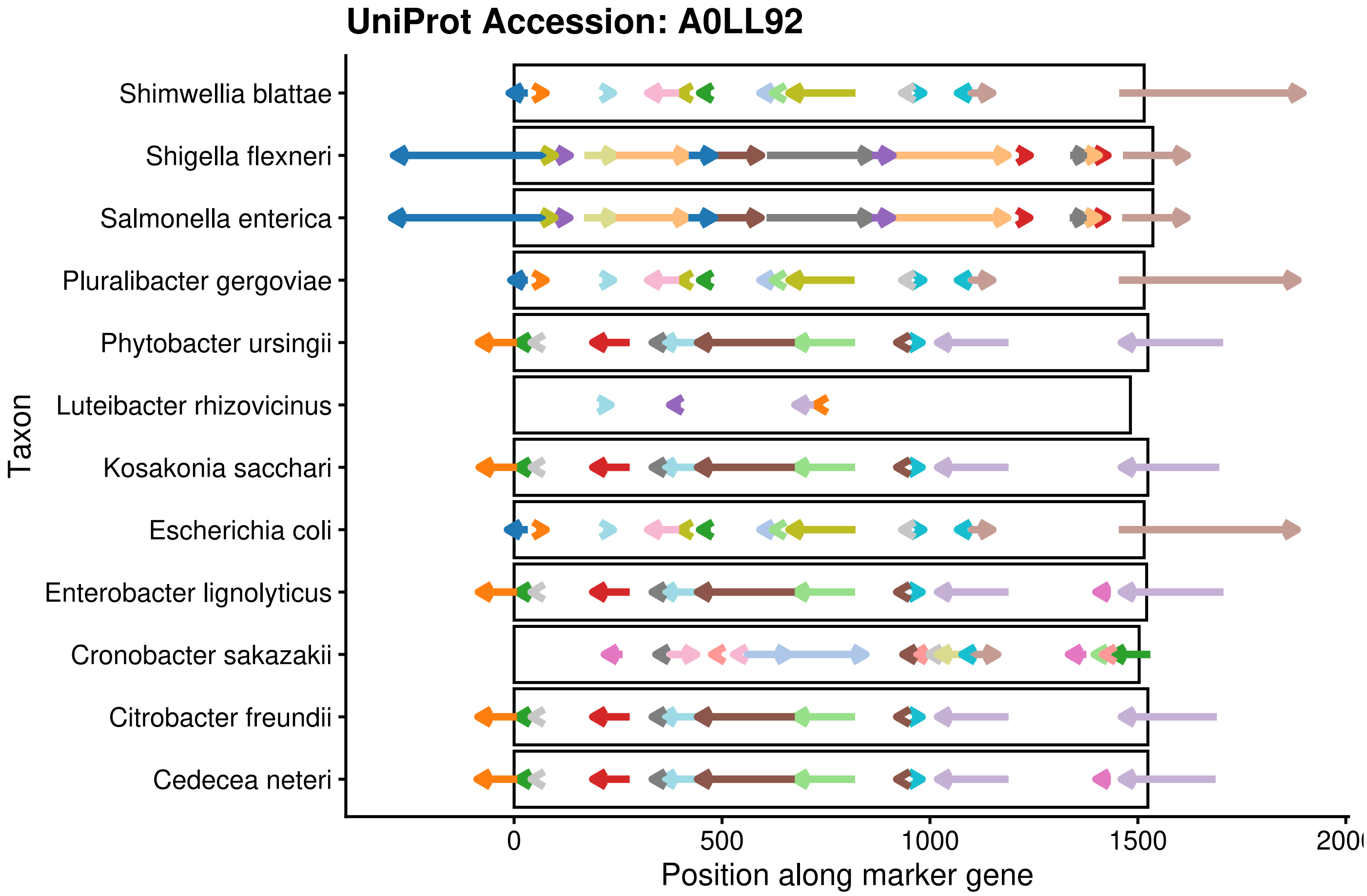

UniProt Accession: A0LL92
Shimwellia blattae
Shigella flexneri
Salmonella enterica
Pluralibacter gergoviae
Phytobacter ursingii
Luteibacter rhizovicinus
Kosakonia sacchari
Escherichia coli
Enterobacter lignolyticus
Cronobacter sakazakii
Citrobacter freundii
Cedecea neteri
Taxon
0
500
1000
1500
Position along marker gene

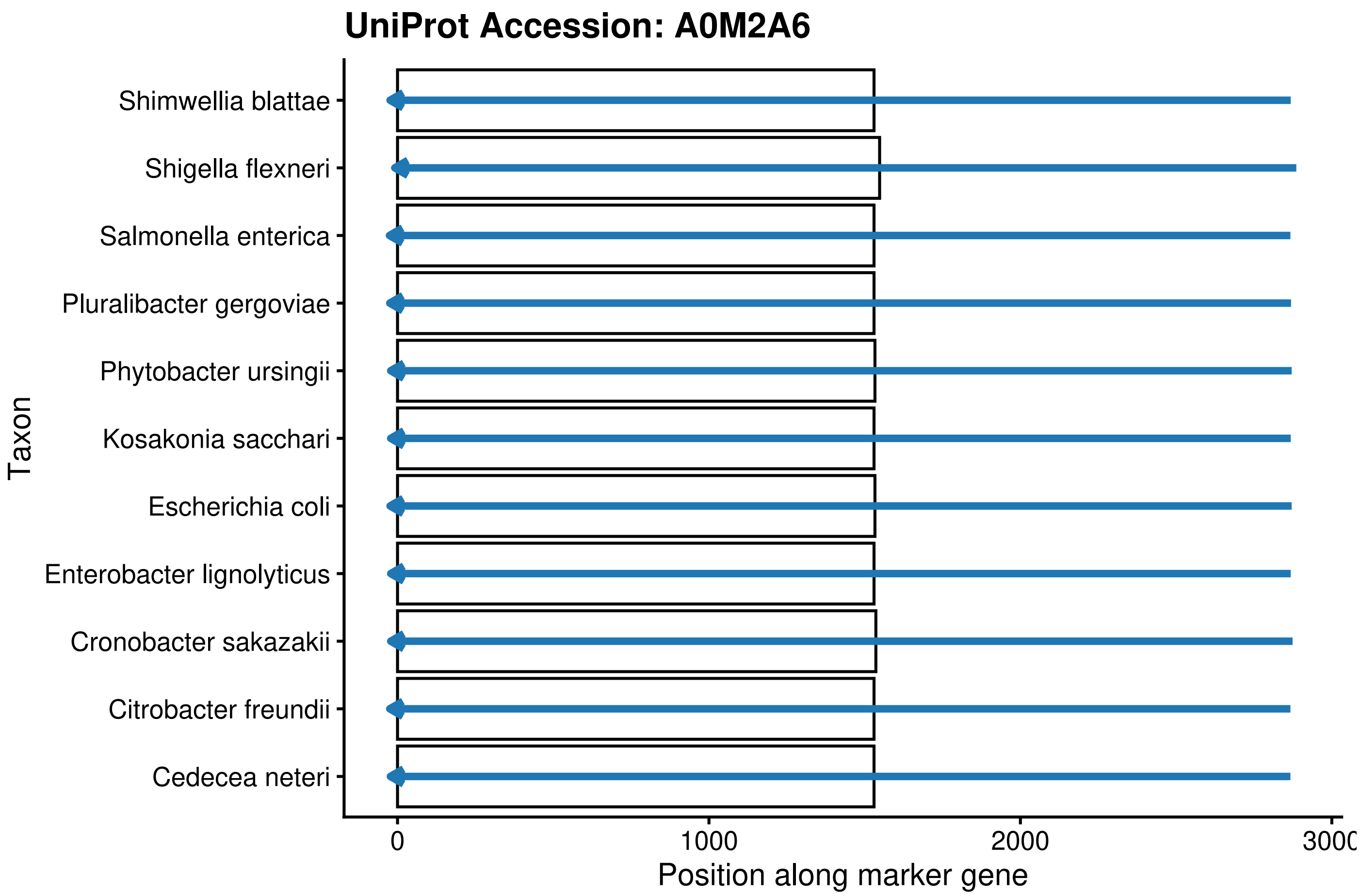
UniProt Accession: A0M2A6
Taxon
Shimwellia blattae
Shigella flexneri
Salmonella enterica
Pluralibacter gergoviae
Phytobacter ursingii
Kosakonia sacchari
Escherichia coli
Enterobacter lignolyticus
Cronobacter sakazakii
Citrobacter freundii
Cedecea neteri
0
1000
2000
3000
Position along marker gene

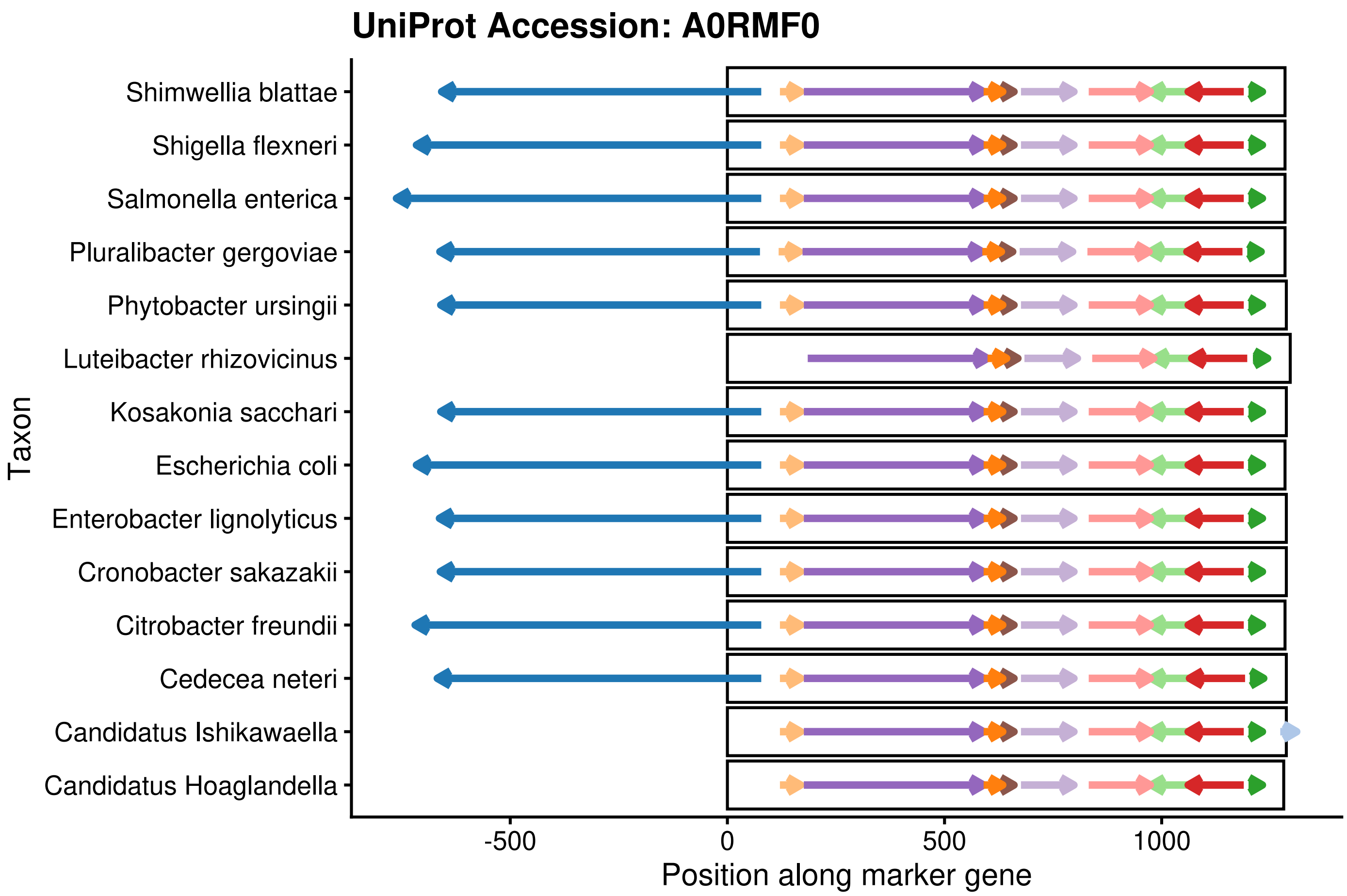
UniProt Accession: A0RMF0
Taxon
Shimwellia blattae
Shigella flexneri
Salmonella enterica
Pluralibacter gergoviae
Phytobacter ursingii
Luteibacter rhizovicinus
Kosakonia sacchari
Escherichia coli
Enterobacter lignolyticus
Cronobacter sakazakii
Citrobacter freundii
Cedecea neteri
Candidatus Ishikawaella
Candidatus Hoaglandella
-500
0
500
1000
Position along marker gene

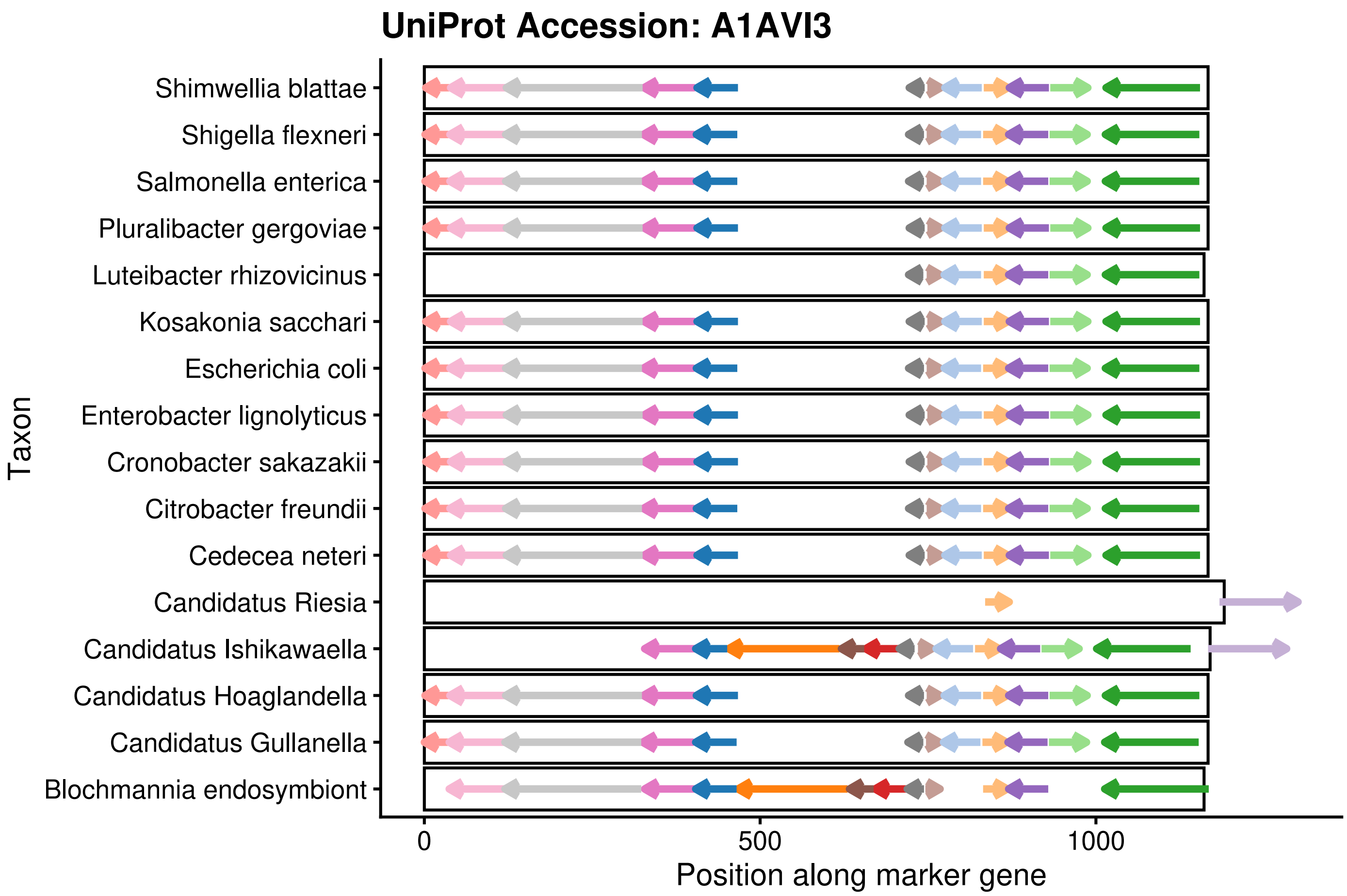
UniProt Accession: A1AVI3
Shimwellia blattae
Shigella flexneri
Salmonella enterica
Pluralibacter gergoviae
Luteibacter rhizovicinus
Kosakonia sacchari
Escherichia coli
Enterobacter lignolyticus
Cronobacter sakazakii
Citrobacter freundii
Cedecea neteri
Candidatus Riesia
Candidatus Ishikawaella
Candidatus Hoaglandella
Candidatus Gullanella
Blochmannia endosymbiont
Taxon
0
500
1000
Position along marker gene

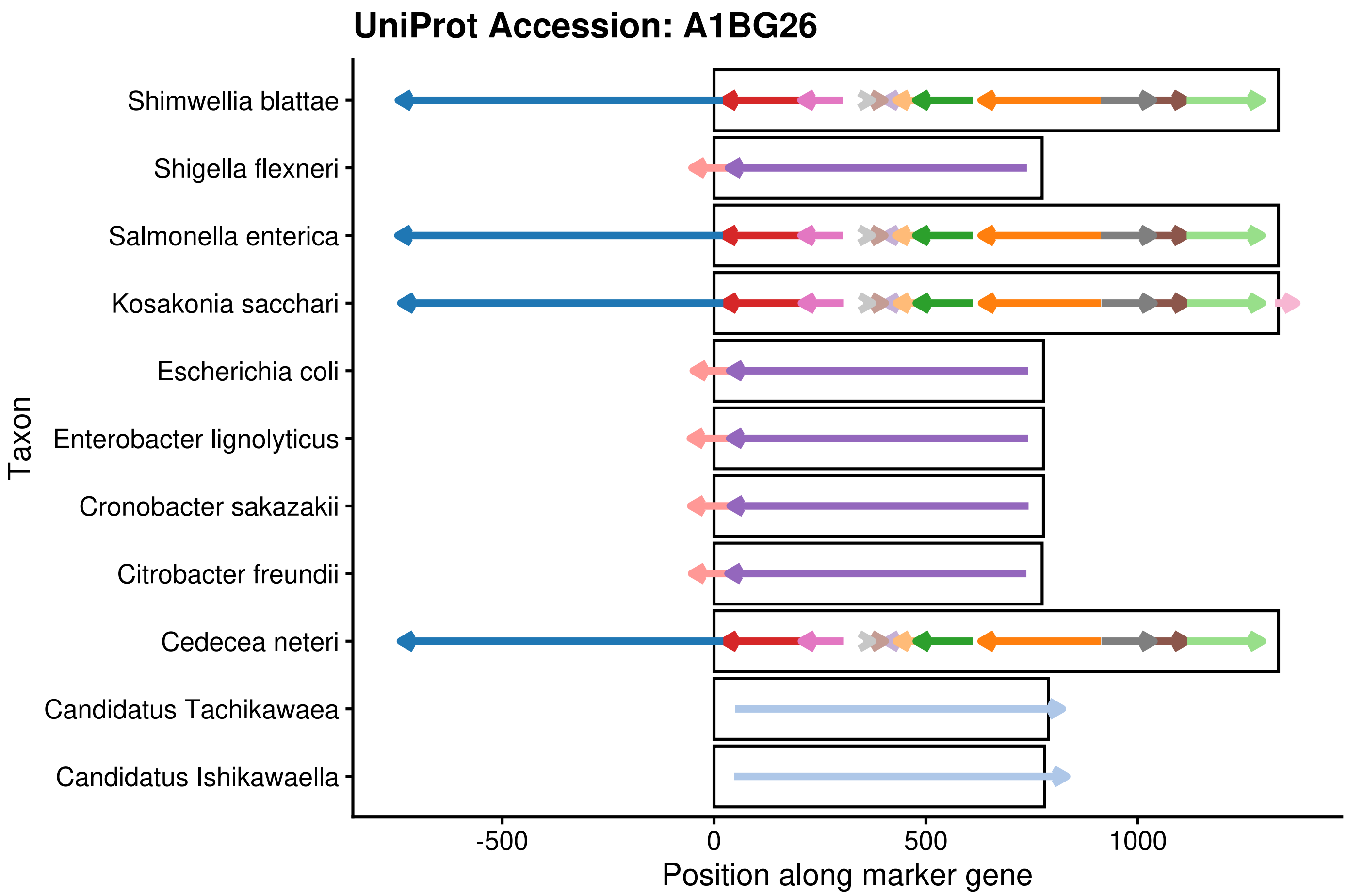

UniProt Accession: A1BG26
Shimwellia blattae
Shigella flexneri
Salmonella enterica
Kosakonia sacchari
Escherichia coli
Enterobacter lignolyticus
Cronobacter sakazakii
Citrobacter freundii
Cedecea neteri
Candidatus Tachikawaea
Candidatus Ishikawaella
Taxon
-500
0
500
1000
Position along marker gene

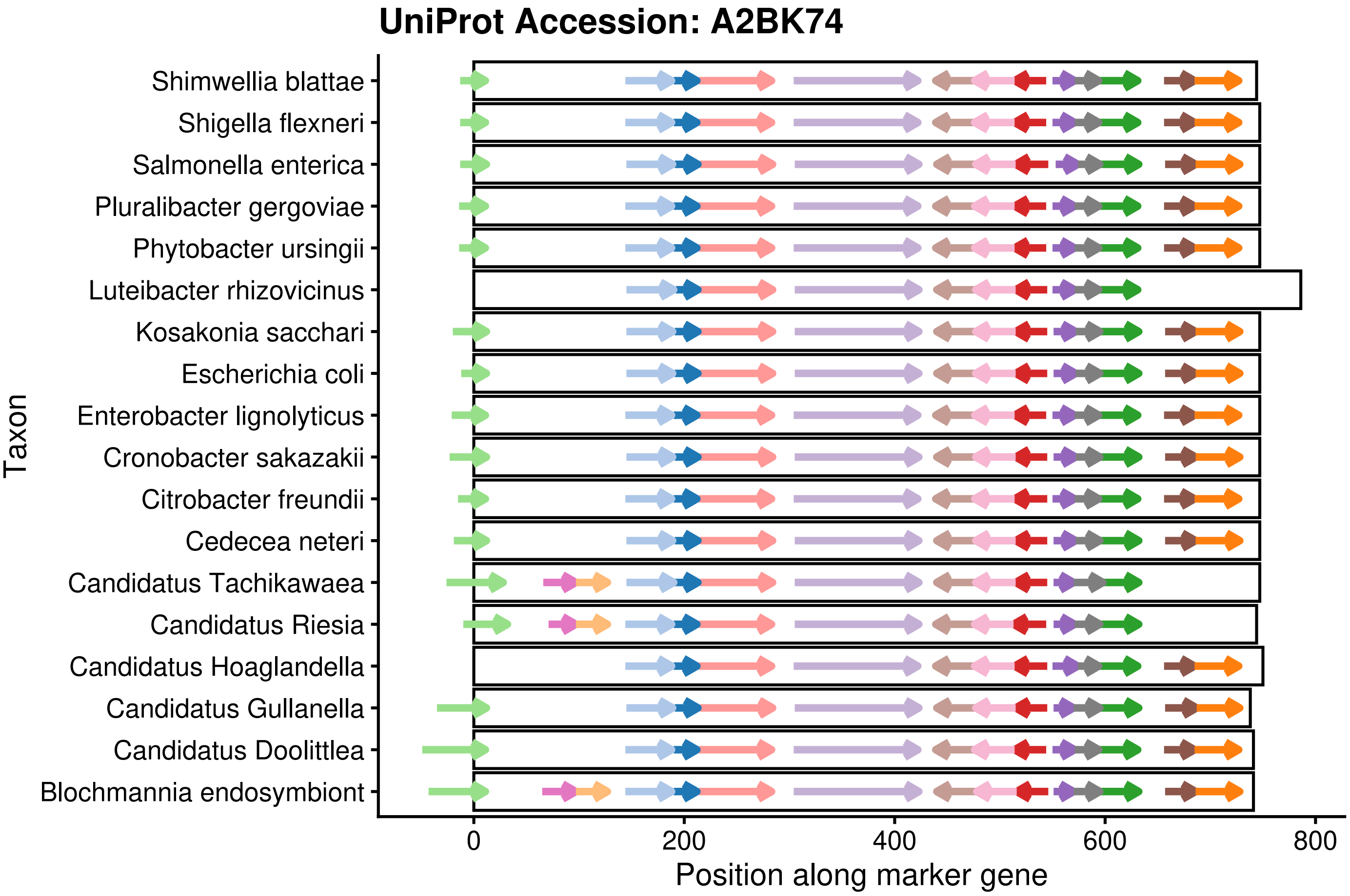
UniProt Accession: A2BK74
Taxon
Shimwellia blattae
Shigella flexneri
Salmonella enterica
Pluralibacter gergoviae
Phytobacter ursingii
Luteibacter rhizovicinus
Kosakonia sacchari
Escherichia coli
Enterobacter lignolyticus
Cronobacter sakazakii
Citrobacter freundii
Cedecea neteri
Candidatus Tachikawaea
Candidatus Riesia
Candidatus Hoaglandella
Candidatus Gullanella
Candidatus Doolittlea
Blochmannia endosymbiont
0
200
400
600
800
Position along marker gene

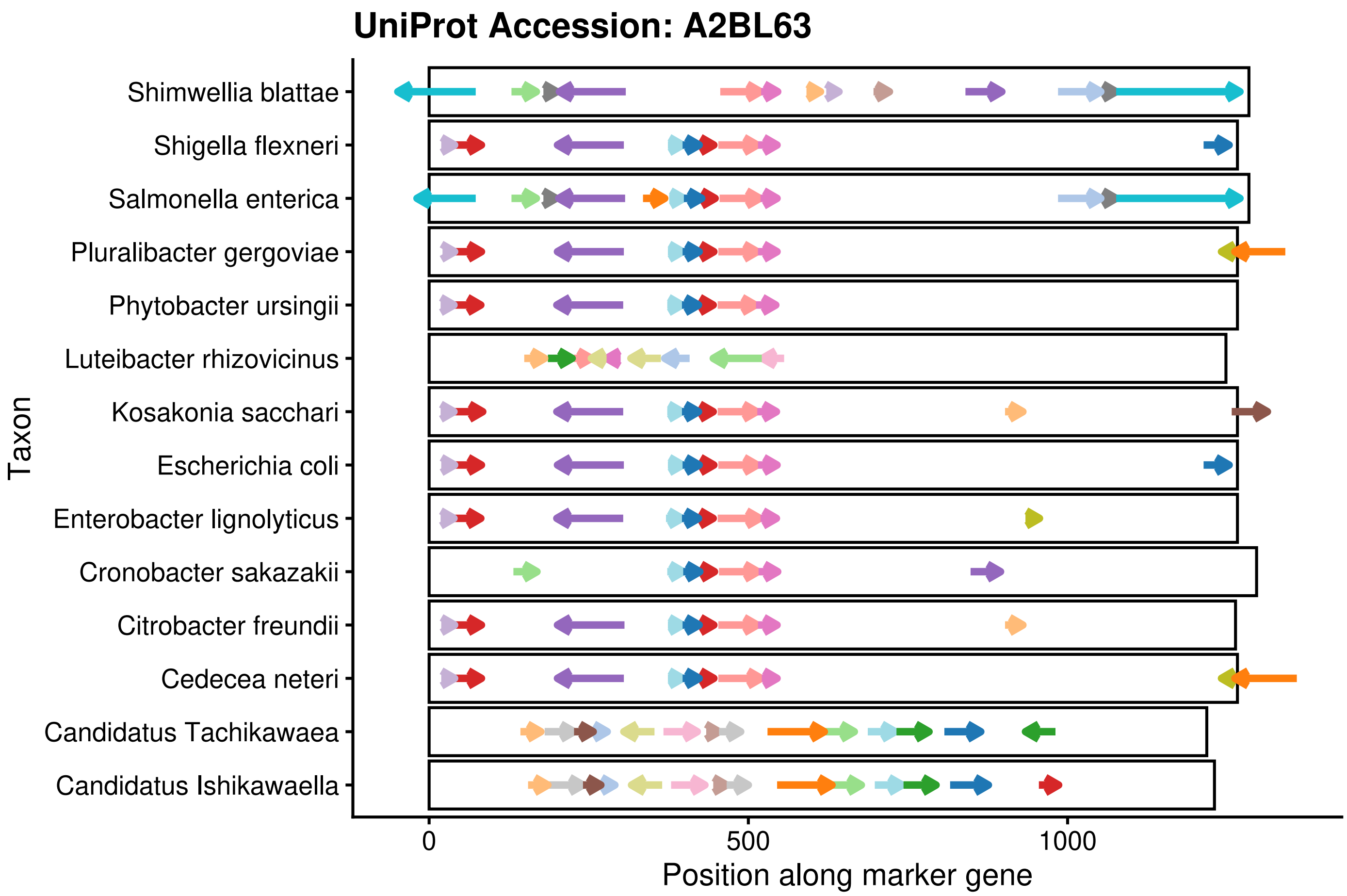

UniProt Accession: A2BL63
Shimwellia blattae
Shigella flexneri
Salmonella enterica
Pluralibacter gergoviae
Phytobacter ursingii
Luteibacter rhizovicinus
Kosakonia sacchari
Escherichia coli
Enterobacter lignolyticus
Cronobacter sakazakii
Citrobacter freundii
Cedecea neteri
Candidatus Tachikawaea
Candidatus Ishikawaella
Taxon
0
500
1000
Position along marker gene

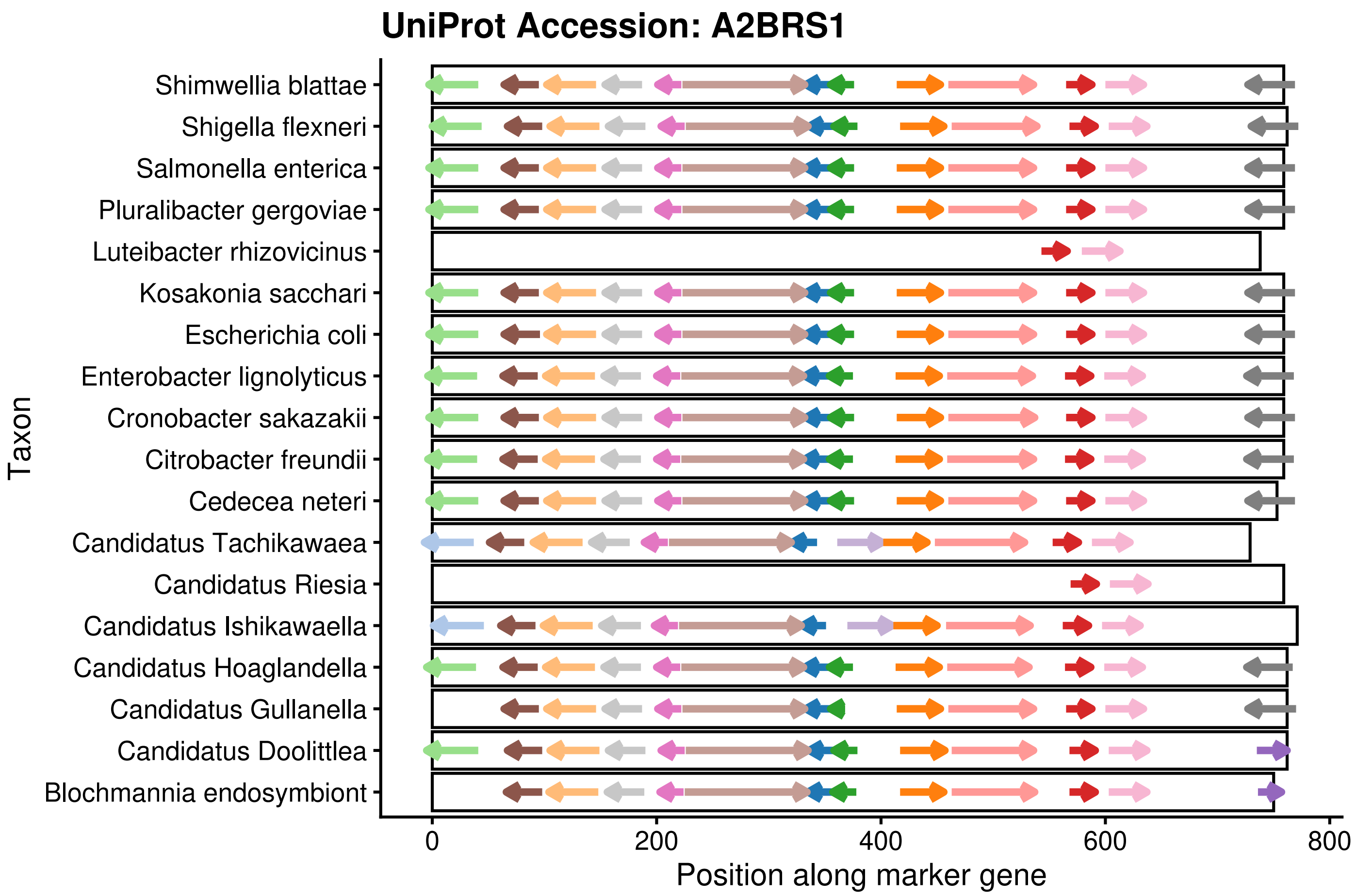

UniProt Accession: A2BRS1
Shimwellia blattae
Shigella flexneri
Salmonella enterica
Pluralibacter gergoviae
Luteibacter rhizovicinus
Kosakonia sacchari
Escherichia coli
Enterobacter lignolyticus
Cronobacter sakazakii
Citrobacter freundii
Cedecea neteri
Candidatus Tachikawaea
Candidatus Riesia
Candidatus Ishikawaella
Candidatus Hoaglandella
Candidatus Gullanella
Candidatus Doolittlea
Blochmannia endosymbiont
Taxon
0
200
400
600
800
Position along marker gene

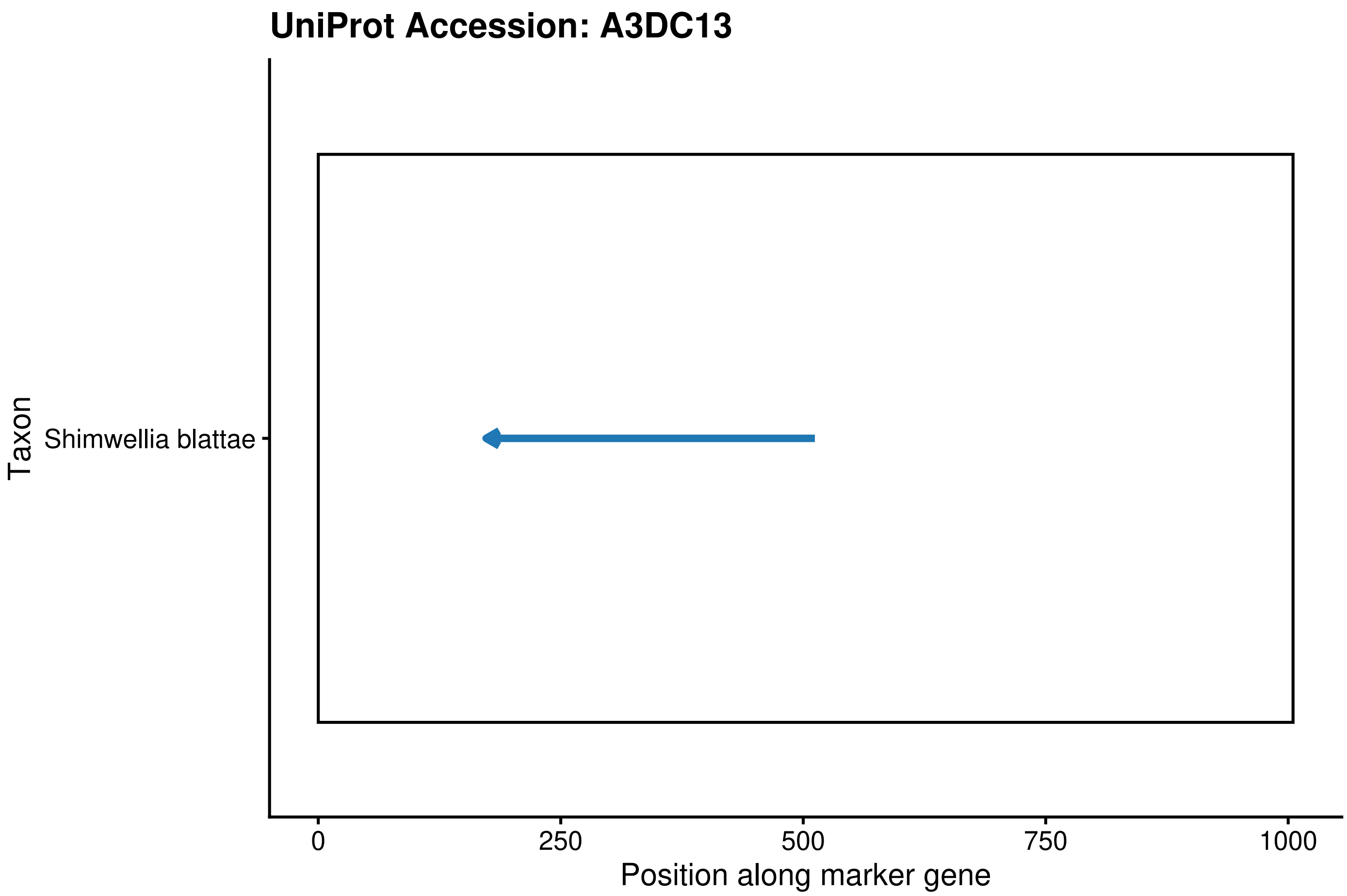
UniProt Accession: A3DC13
Taxon
Shimwellia blattae
0
250
500
750
1000
Position along marker gene

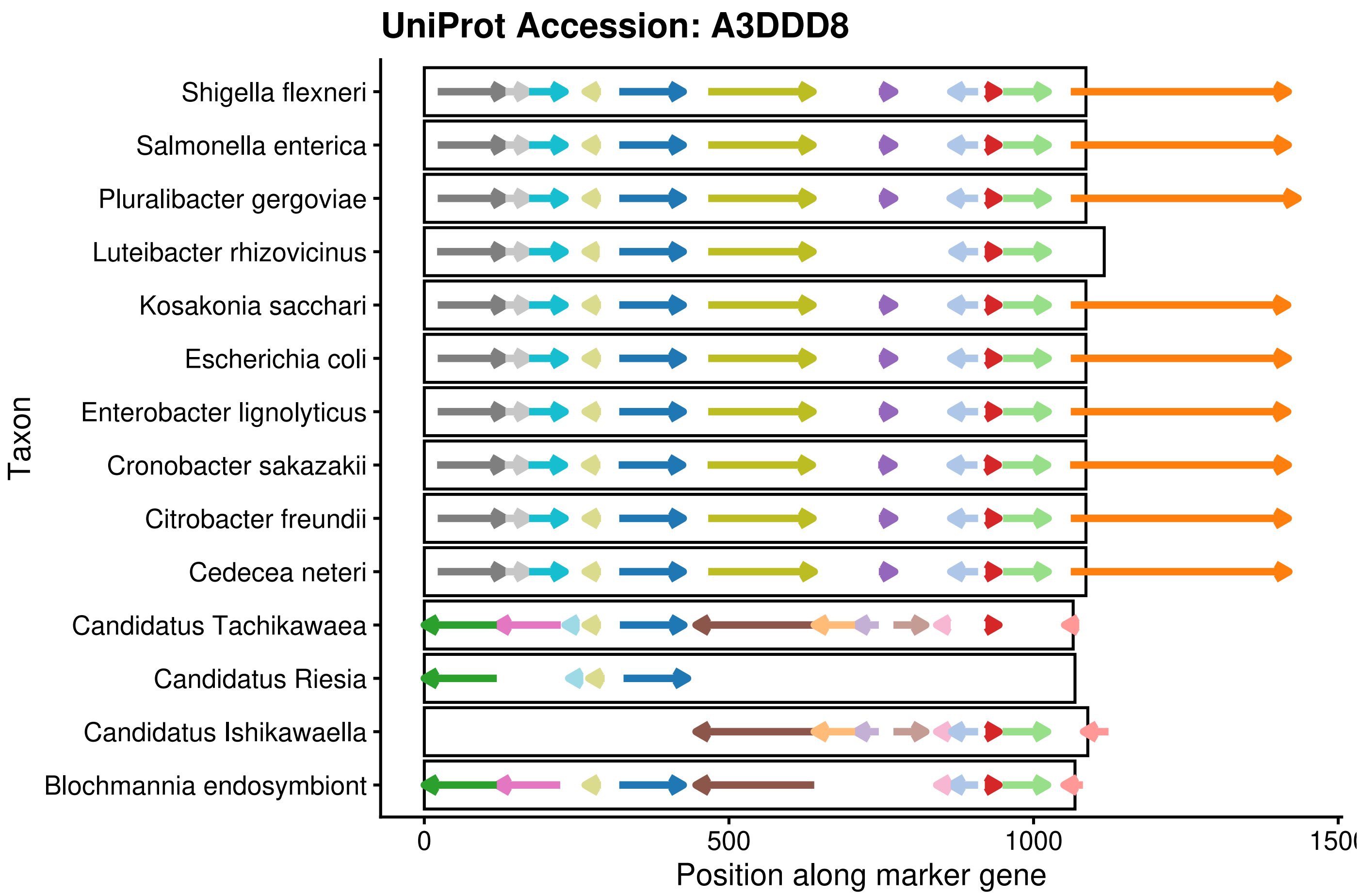
UniProt Accession: A3DDD8
Shigella flexneri
Salmonella enterica
Pluralibacter gergoviae
Luteibacter rhizovicinus
Kosakonia sacchari
Escherichia coli
Enterobacter lignolyticus
Cronobacter sakazakii
Citrobacter freundii
Cedecea neteri
Candidatus Tachikawaea
Candidatus Riesia
Candidatus Ishikawaella
Blochmannia endosymbiont
Taxon
0
500
1000
Position along marker gene

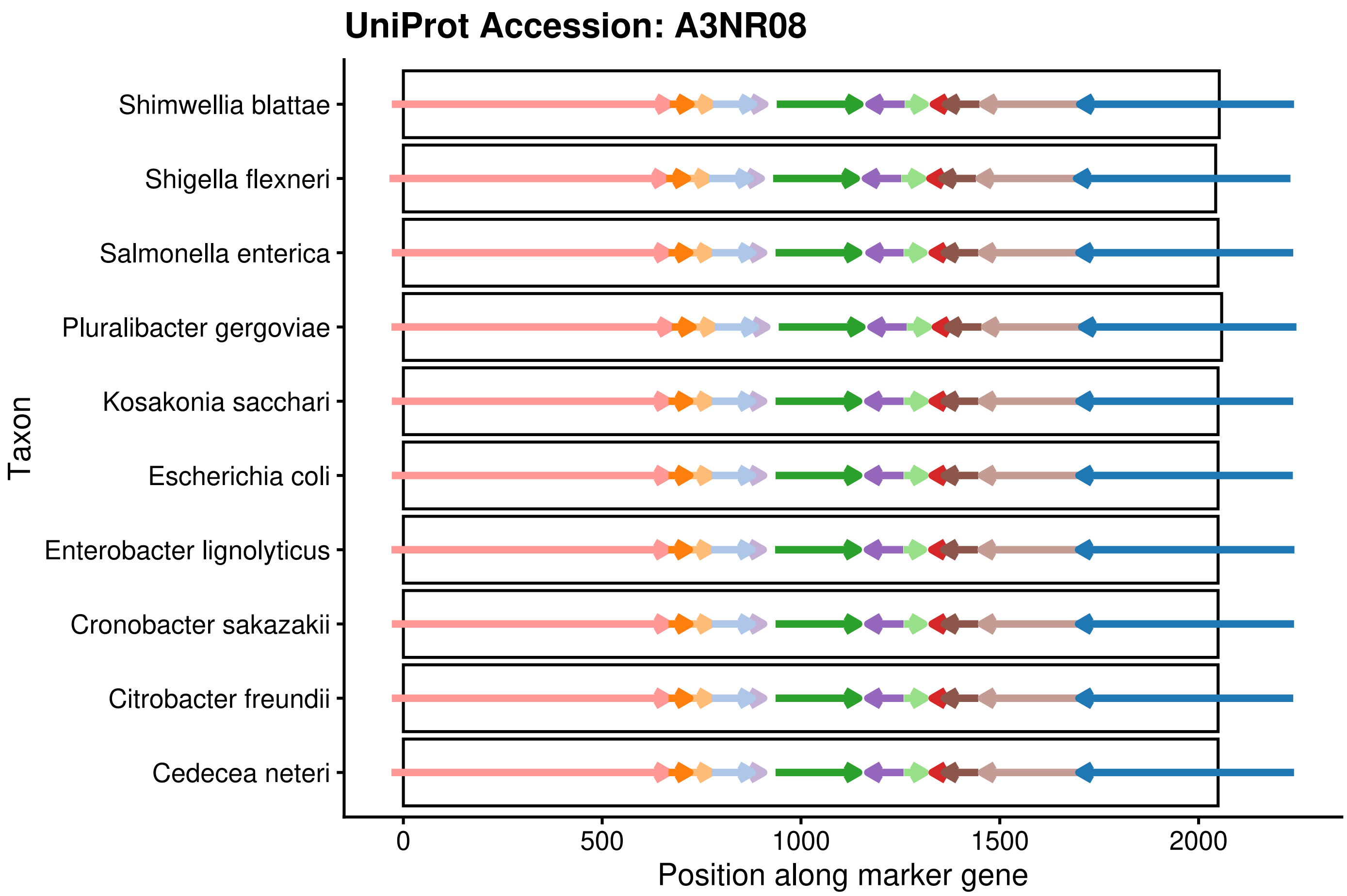
UniProt Accession: A3NR08
Taxon
Shimwellia blattae
Shigella flexneri
Salmonella enterica
Pluralibacter gergoviae
Kosakonia sacchari
Escherichia coli
Enterobacter lignolyticus
Cronobacter sakazakii
Citrobacter freundii
Cedecea neteri
0
500
1000
1500
2000
Position along marker gene

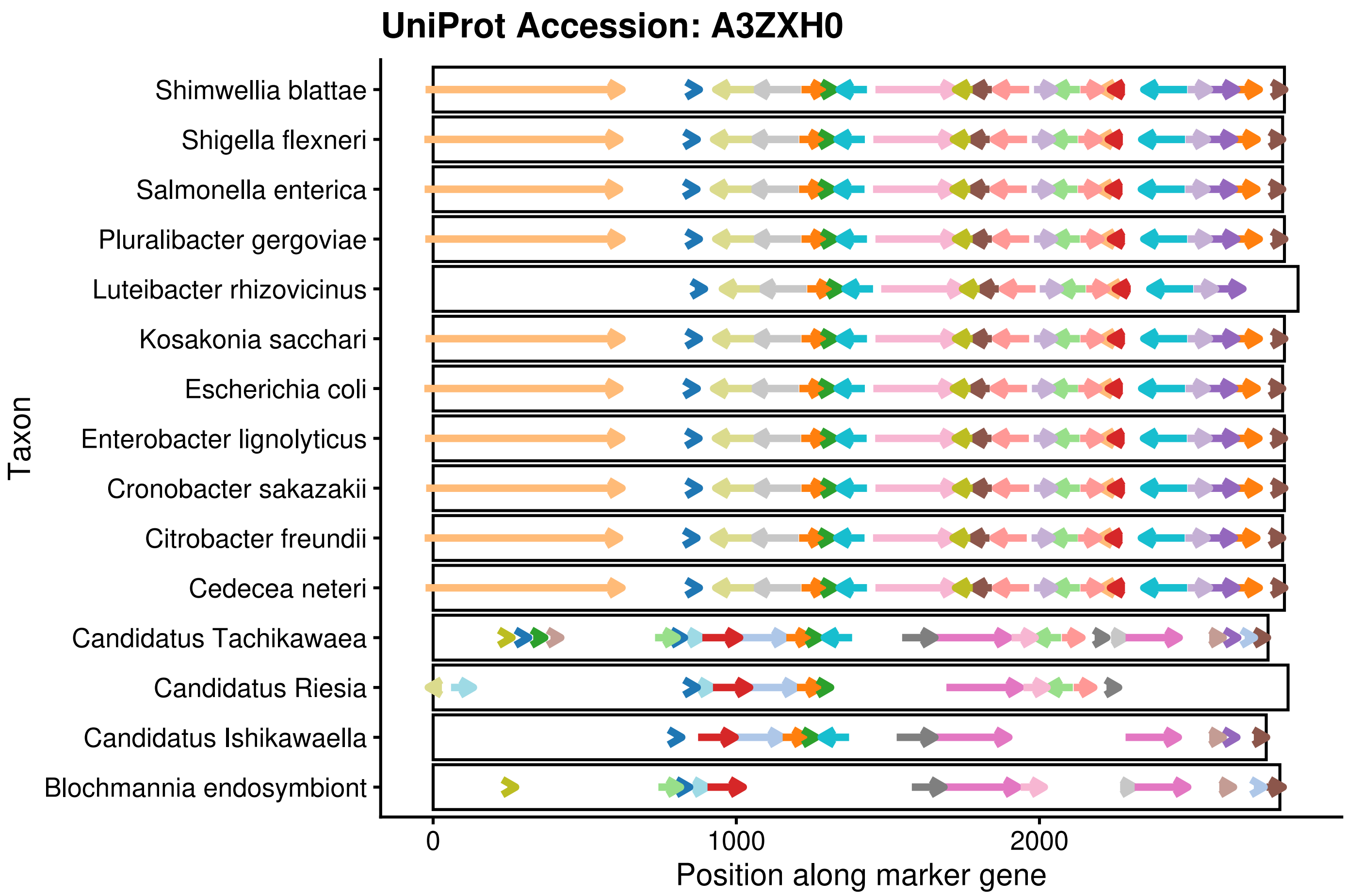
UniProt Accession: A3ZXH0
Taxon
Shimwellia blattae
Shigella flexneri
Salmonella enterica
Pluralibacter gergoviae
Luteibacter rhizovicinus
Kosakonia sacchari
Escherichia coli
Enterobacter lignolyticus
Cronobacter sakazakii
Citrobacter freundii
Cedecea neteri
Candidatus Tachikawaea
Candidatus Riesia
Candidatus Ishikawaella
Blochmannia endosymbiont
0
1000
2000
Position along marker gene

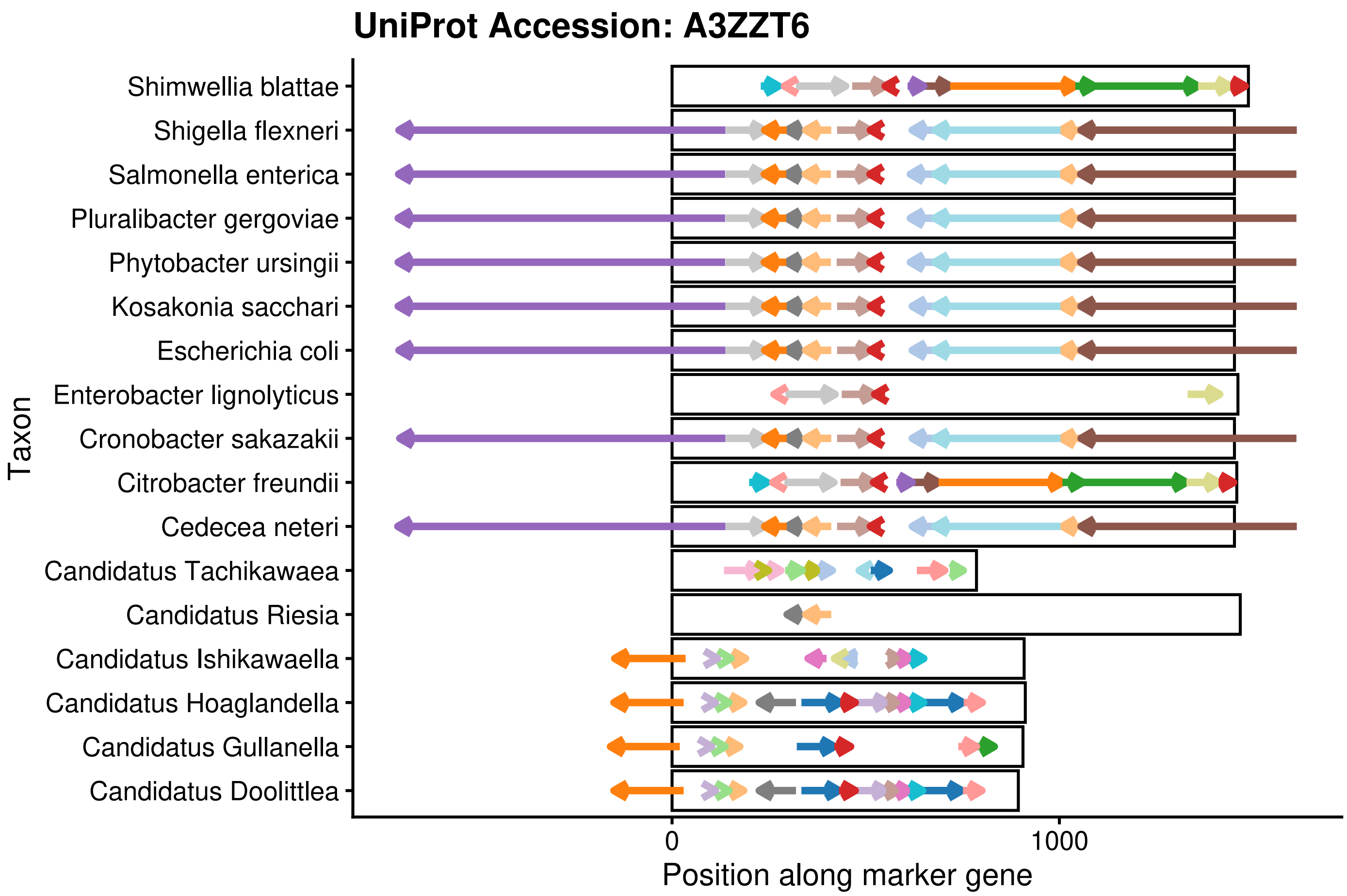

UniProt Accession: A3ZZT6
Shimwellia blattae
Shigella flexneri
Salmonella enterica
Pluralibacter gergoviae
Phytobacter ursingii
Kosakonia sacchari
Escherichia coli
Enterobacter lignolyticus
Cronobacter sakazakii
Citrobacter freundii
Cedecea neteri
Candidatus Tachikawaea
Candidatus Riesia
Candidatus Ishikawaella
Candidatus Hoaglandella
Candidatus Gullanella
Candidatus Doolittlea
Taxon
0
1000
Position along marker gene

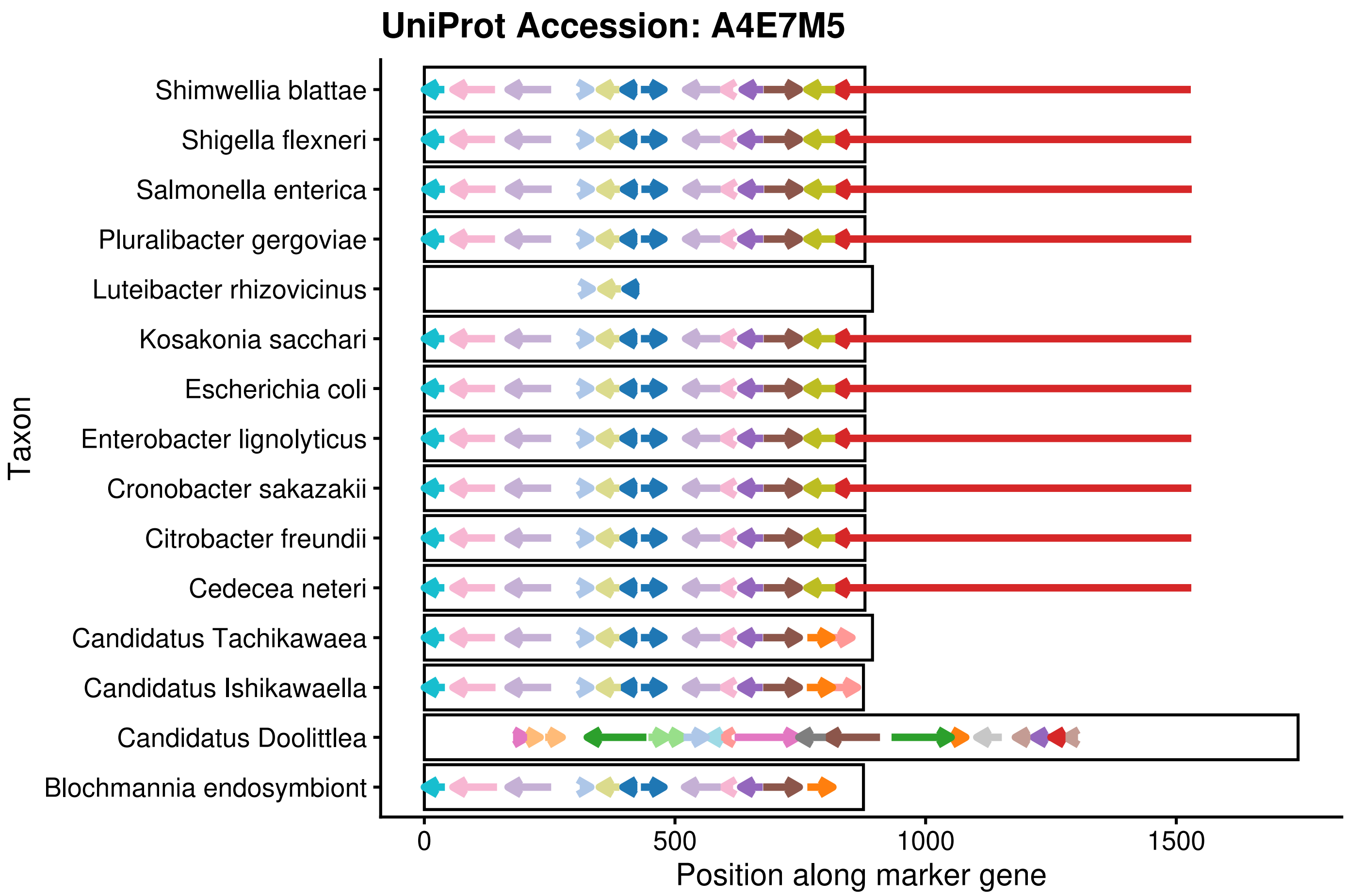

UniProt Accession: A4E7M5
Taxon
Shimwellia blattae
Shigella flexneri
Salmonella enterica
Pluralibacter gergoviae
Luteibacter rhizovicinus
Kosakonia sacchari
Escherichia coli
Enterobacter lignolyticus
Cronobacter sakazakii
Citrobacter freundii
Cedecea neteri
Candidatus Tachikawaea
Candidatus Ishikawaella
Candidatus Doolittlea
Blochmannia endosymbiont
0
500
1000
1500
Position along marker gene

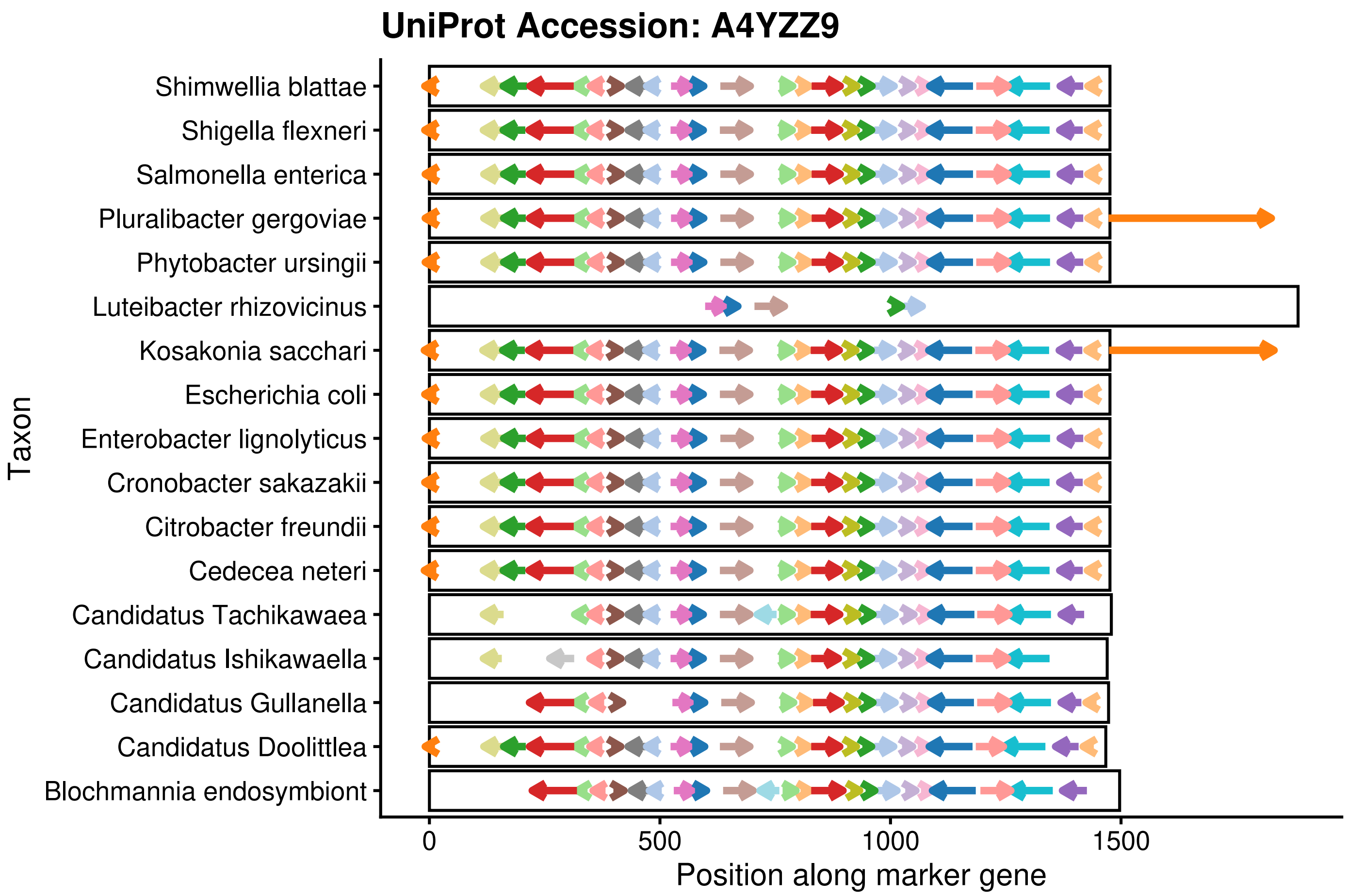
UniProt Accession: A4YZZ9
Shimwellia blattae
Shigella flexneri
Salmonella enterica
Pluralibacter gergoviae
Phytobacter ursingii
Luteibacter rhizovicinus
Kosakonia sacchari
Escherichia coli
Enterobacter lignolyticus
Cronobacter sakazakii
Citrobacter freundii
Cedecea neteri
Candidatus Tachikawaea
Candidatus Ishikawaella
Candidatus Gullanella
Candidatus Doolittlea
Blochmannia endosymbiont
Taxon
0
500
1000
1500
Position along marker gene

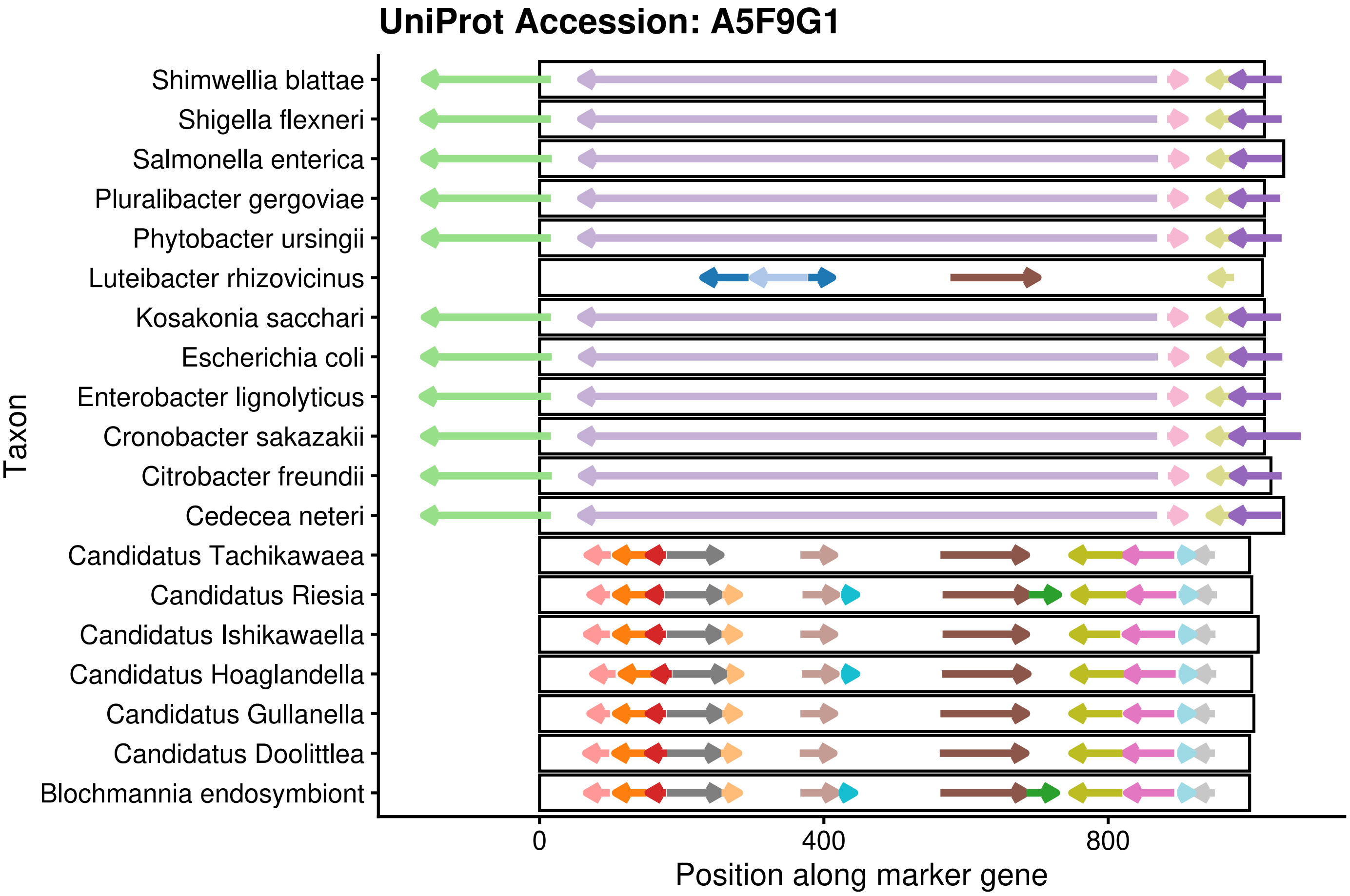
UniProt Accession: A5F9G1
Taxon
Shimwellia blattae
Shigella flexneri
Salmonella enterica
Pluralibacter gergoviae
Phytobacter ursingii
Luteibacter rhizovicinus
Kosakonia sacchari
Escherichia coli
Enterobacter lignolyticus
Cronobacter sakazakii
Citrobacter freundii
Cedecea neteri
Candidatus Tachikawaea
Candidatus Riesia
Candidatus Ishikawaella
Candidatus Hoaglandella
Candidatus Gullanella
Candidatus Doolittlea
Blochmannia endosymbiont
0
400
800
Position along marker gene

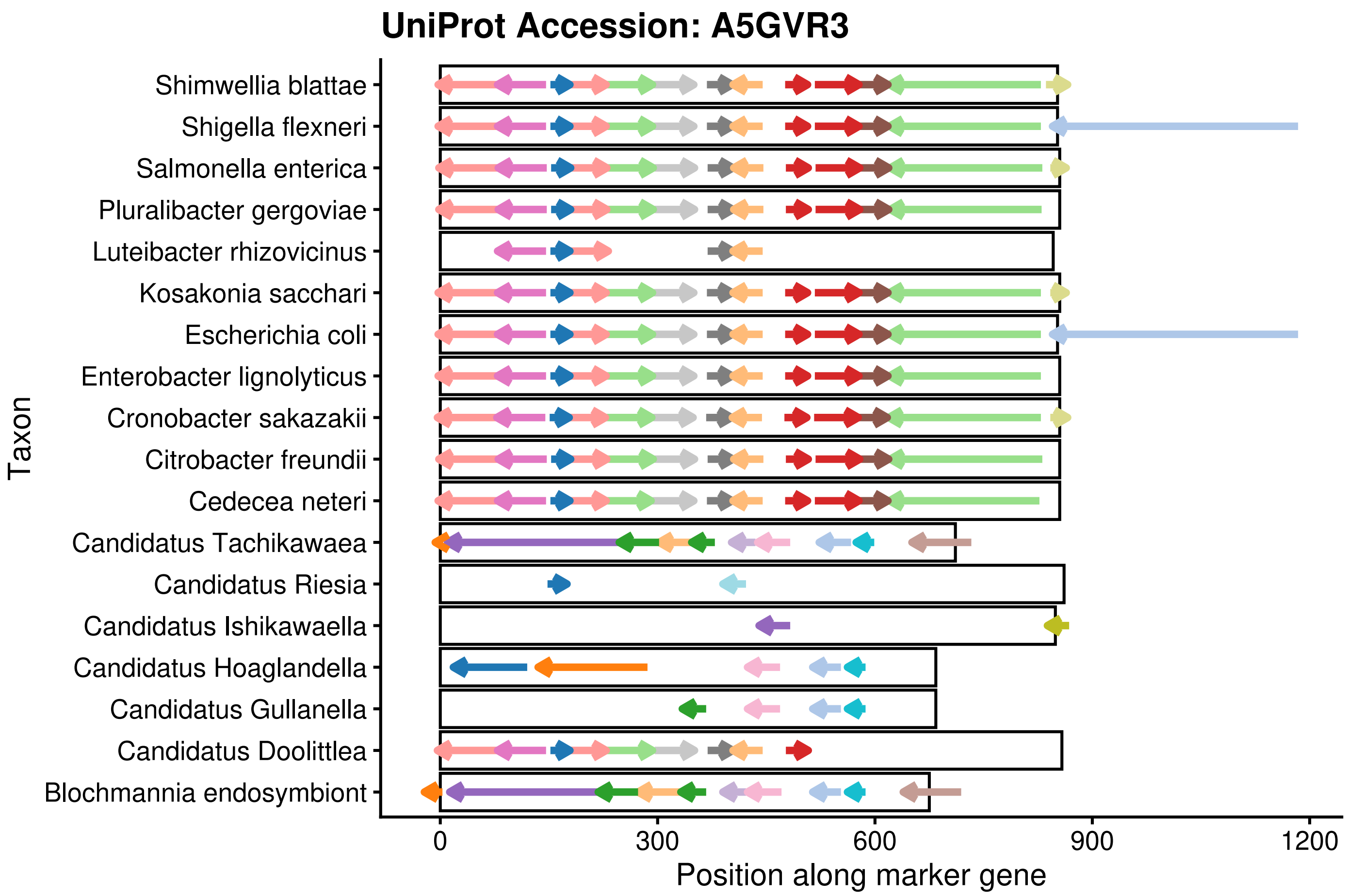
UniProt Accession: A5GVR3
Taxon
Shimwellia blattae
Shigella flexneri
Salmonella enterica
Pluralibacter gergoviae
Luteibacter rhizovicinus
Kosakonia sacchari
Escherichia coli
Enterobacter lignolyticus
Cronobacter sakazakii
Citrobacter freundii
Cedecea neteri
Candidatus Tachikawaea
Candidatus Riesia
Candidatus Ishikawaella
Candidatus Hoaglandella
Candidatus Gullanella
Candidatus Doolittlea
Blochmannia endosymbiont
0
300
600
900
1200
Position along marker gene

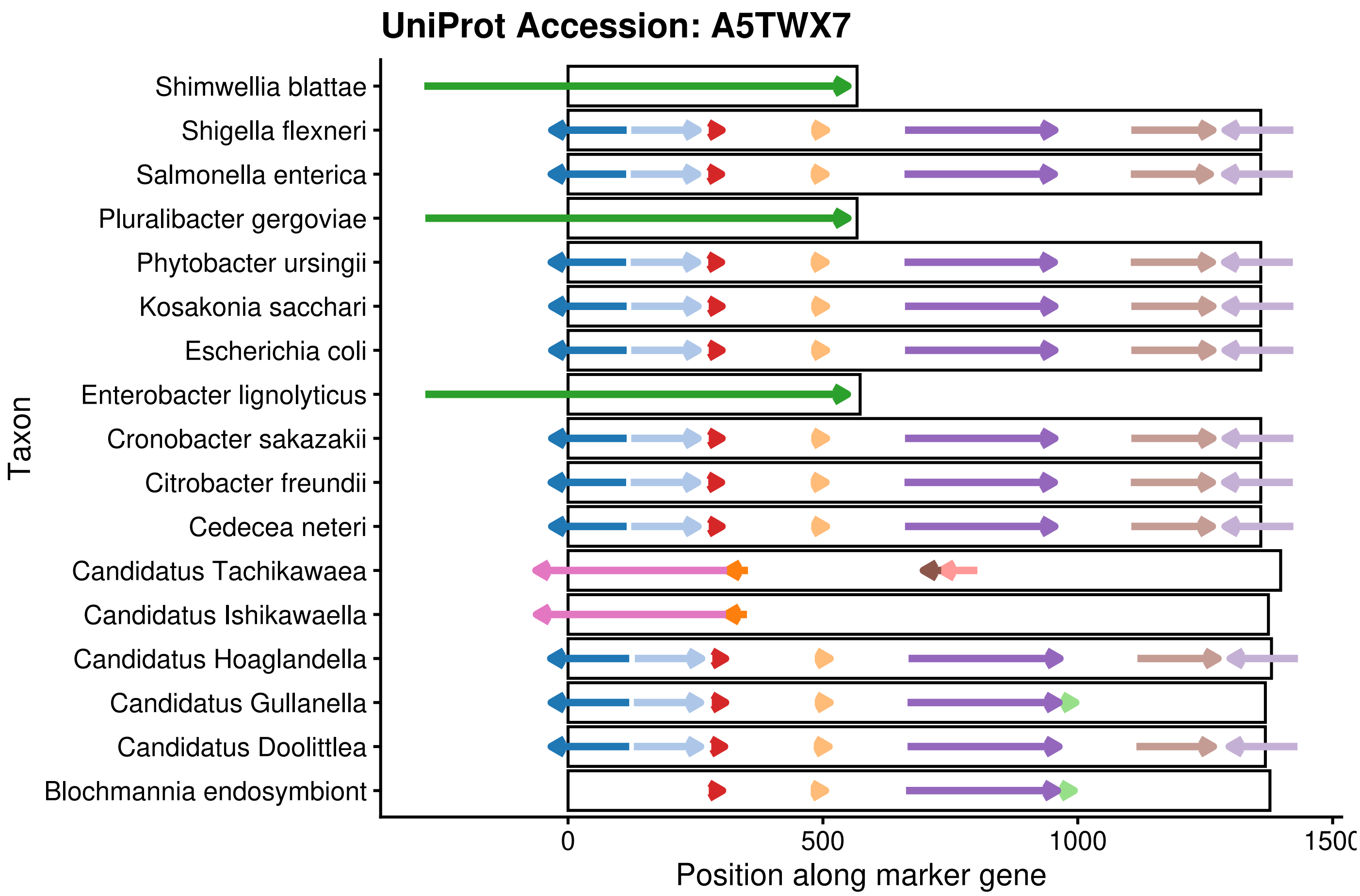
UniProt Accession: A5TWX7
Taxon
Shimwellia blattae
Shigella flexneri
Salmonella enterica
Pluralibacter gergoviae
Phytobacter ursingii
Kosakonia sacchari
Escherichia coli
Enterobacter lignolyticus
Cronobacter sakazakii
Citrobacter freundii
Cedecea neteri
Candidatus Tachikawaea
Candidatus Ishikawaella
Candidatus Hoaglandella
Candidatus Gullanella
Candidatus Doolittlea
Blochmannia endosymbiont
0
500
1000
1500
Position along marker gene

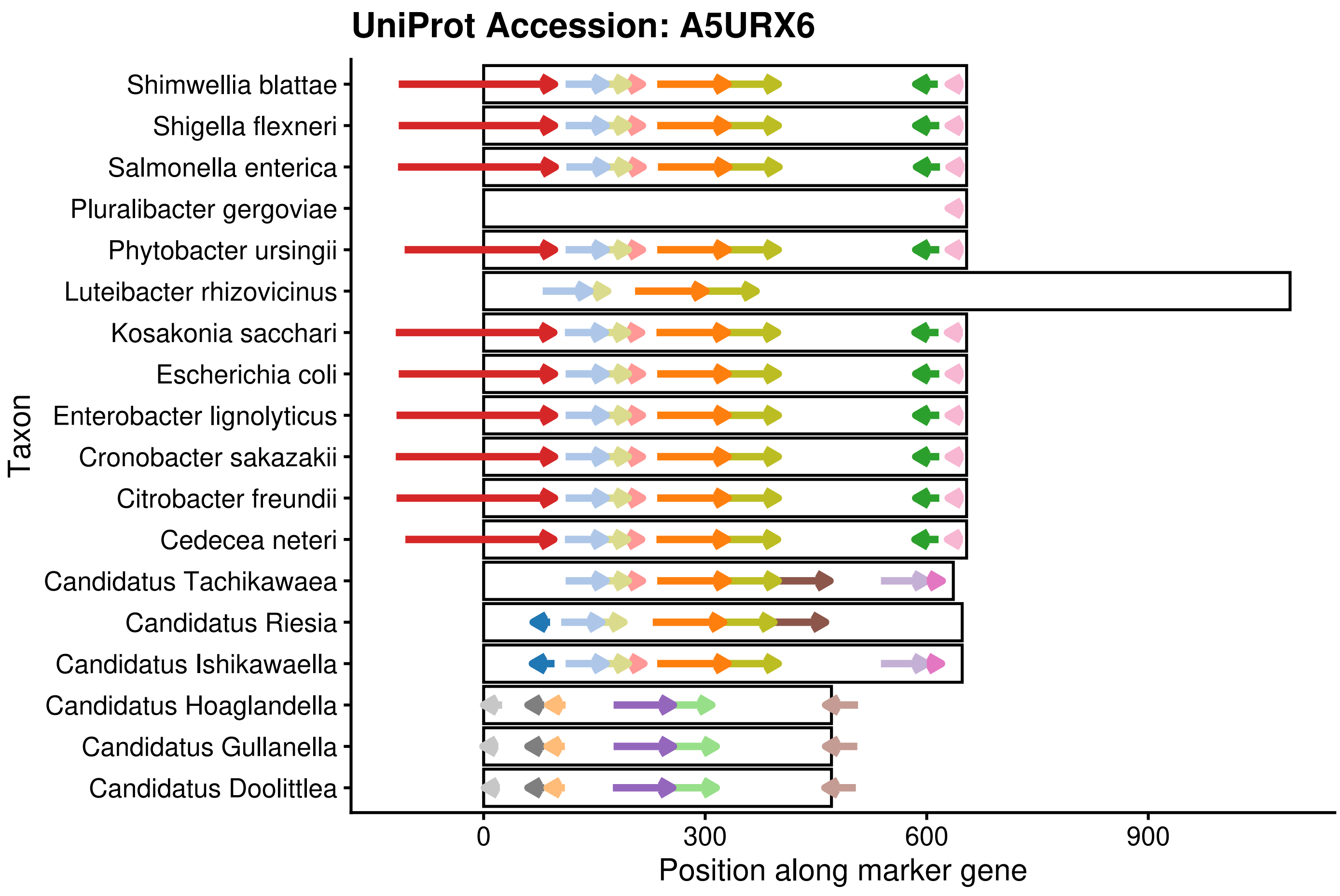

UniProt Accession: A5URX6
Taxon
Shimwellia blattae
Shigella flexneri
Salmonella enterica
Pluralibacter gergoviae
Phytobacter ursingii
Luteibacter rhizovicinus
Kosakonia sacchari
Escherichia coli
Enterobacter lignolyticus
Cronobacter sakazakii
Citrobacter freundii
Cedecea neteri
Candidatus Tachikawaea
Candidatus Riesia
Candidatus Ishikawaella
Candidatus Hoaglandella
Candidatus Gullanella
Candidatus Doolittlea
0
300
600
900
Position along marker gene

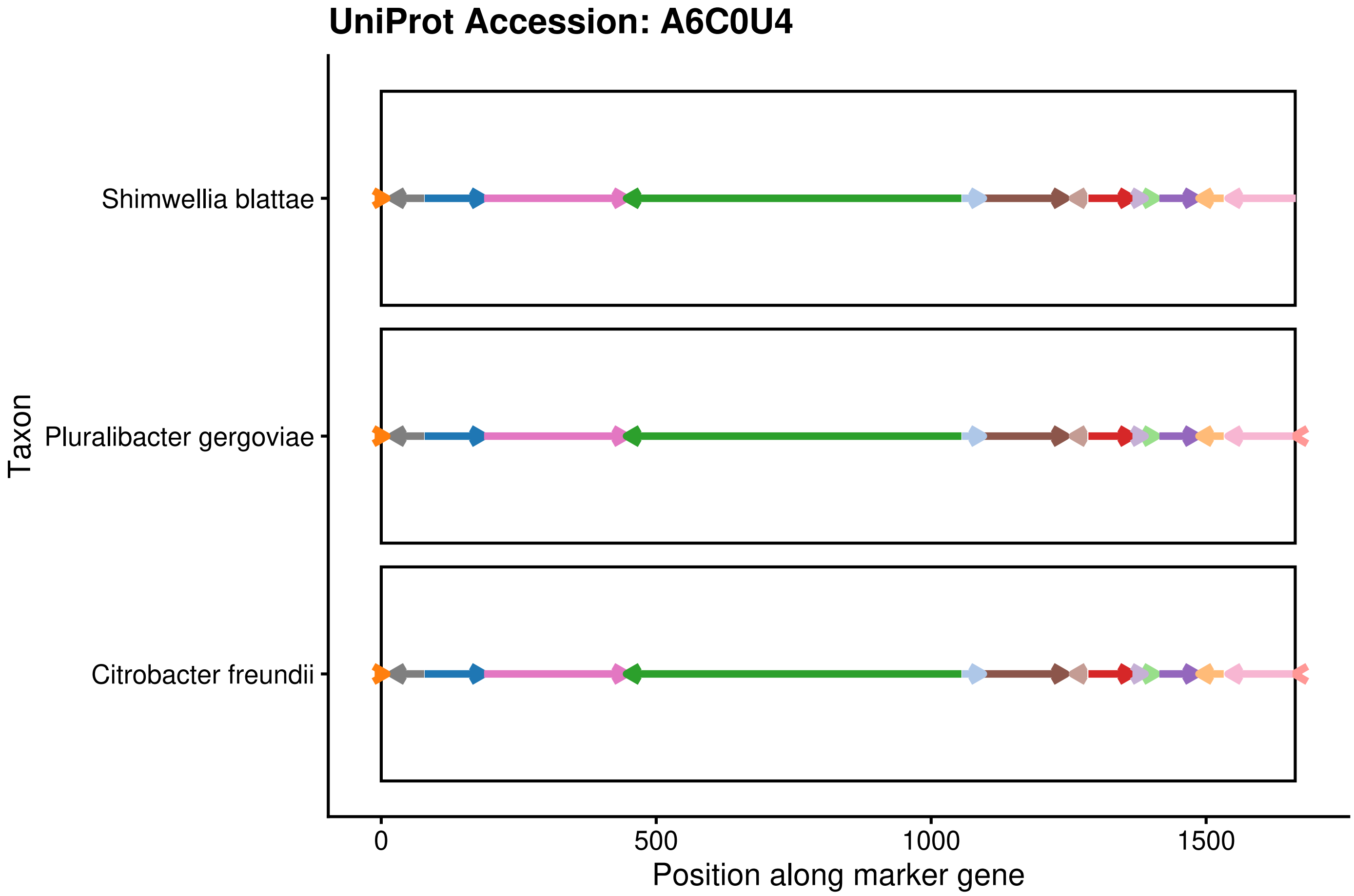

UniProt Accession: A6C0U4
Taxon
Shimwellia blattae
Pluralibacter gergoviae
Citrobacter freundii
0
500
1000
1500
Position along marker gene

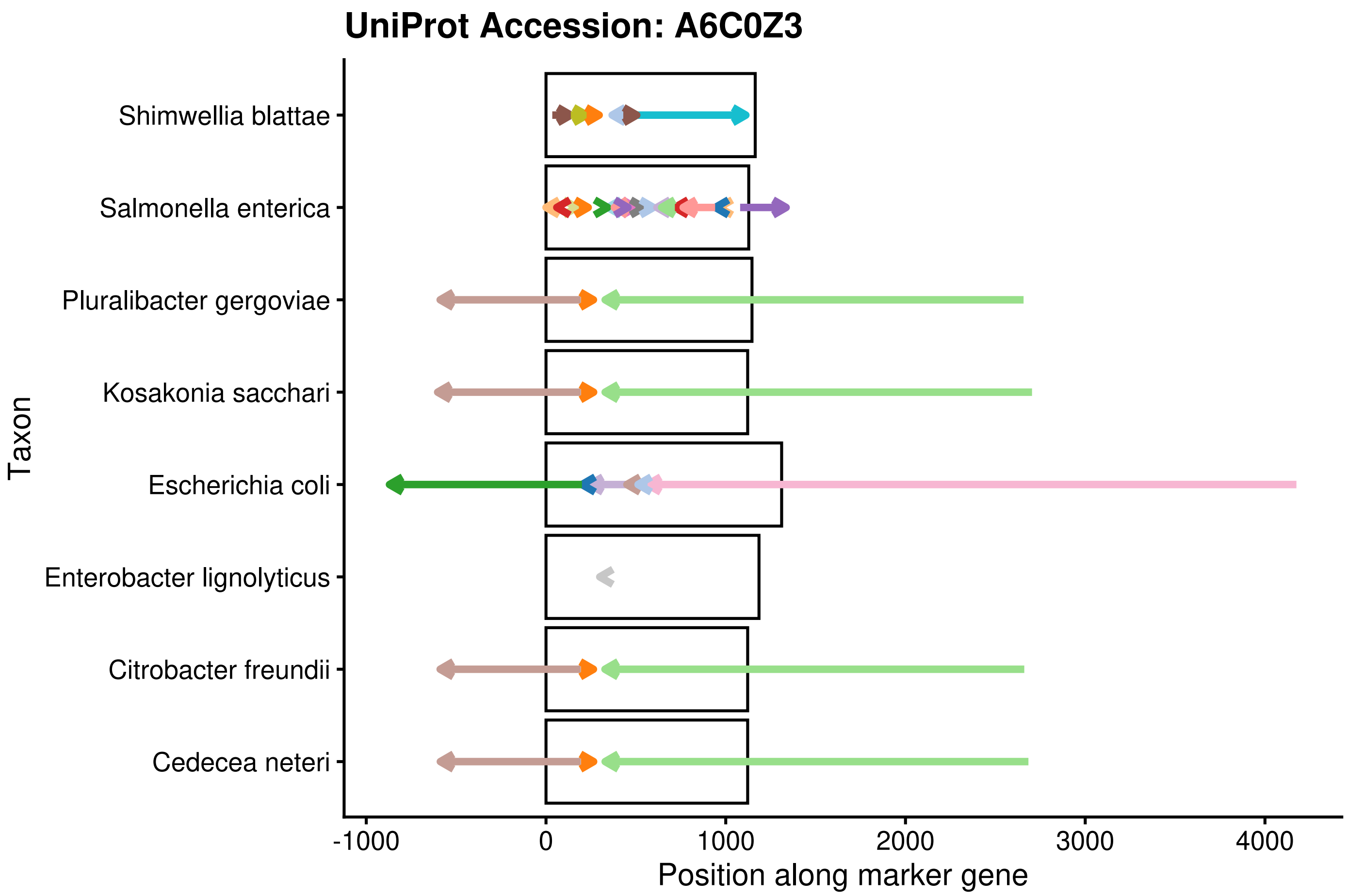
UniProt Accession: A6C0Z3
Shimwellia blattae
Salmonella enterica
Pluralibacter gergoviae
Kosakonia sacchari
Escherichia coli
Enterobacter lignolyticus
Citrobacter freundii
Cedecea neteri
Taxon
-1000
0
1000
2000
3000
4000
Position along marker gene

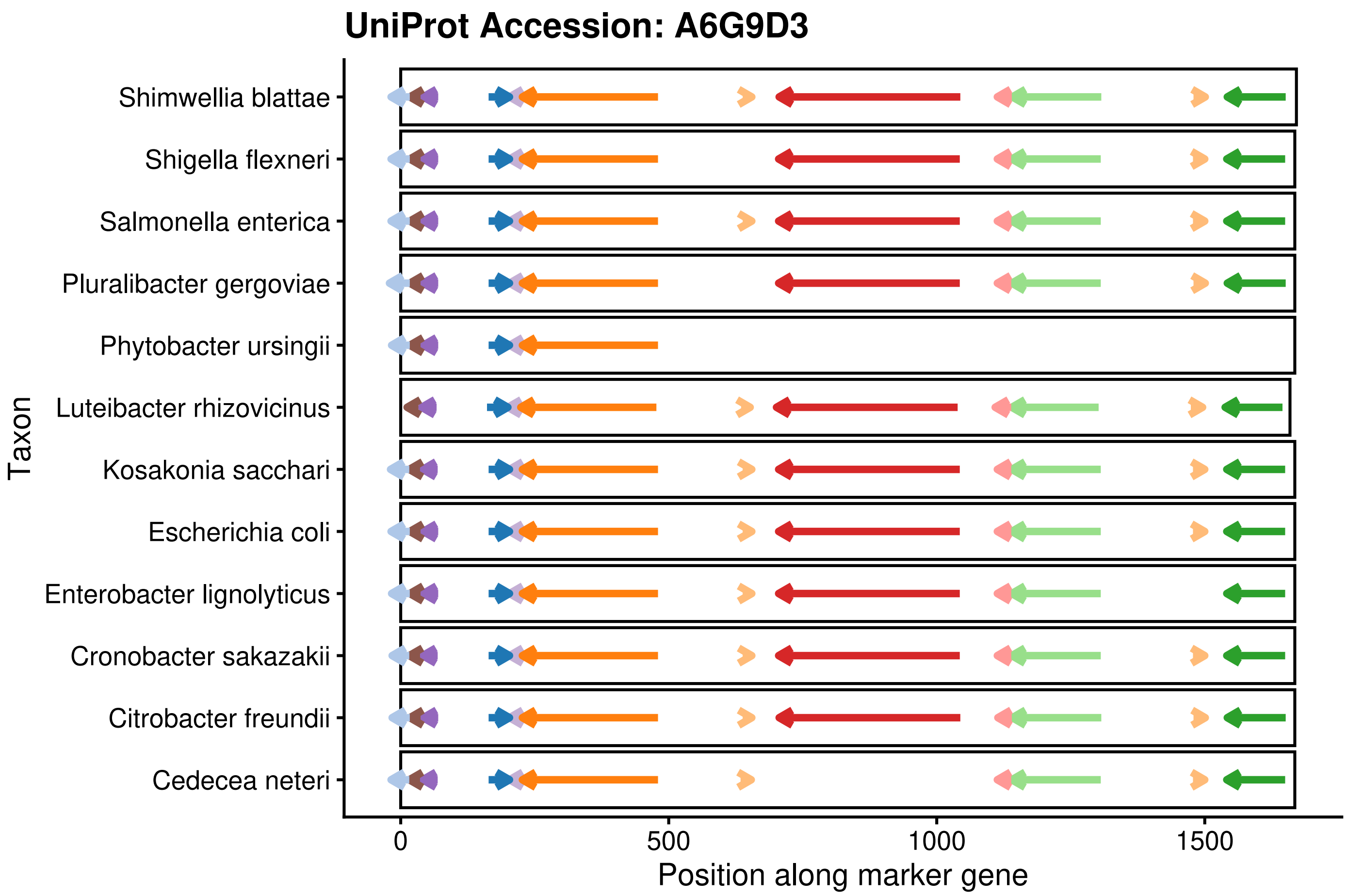
UniProt Accession: A6G9D3
Taxon
Shimwellia blattae
Shigella flexneri
Salmonella enterica
Pluralibacter gergoviae
Phytobacter ursingii
Luteibacter rhizovicinus
Kosakonia sacchari
Escherichia coli
Enterobacter lignolyticus
Cronobacter sakazakii
Citrobacter freundii
Cedecea neteri
0
500
1000
1500
Position along marker gene

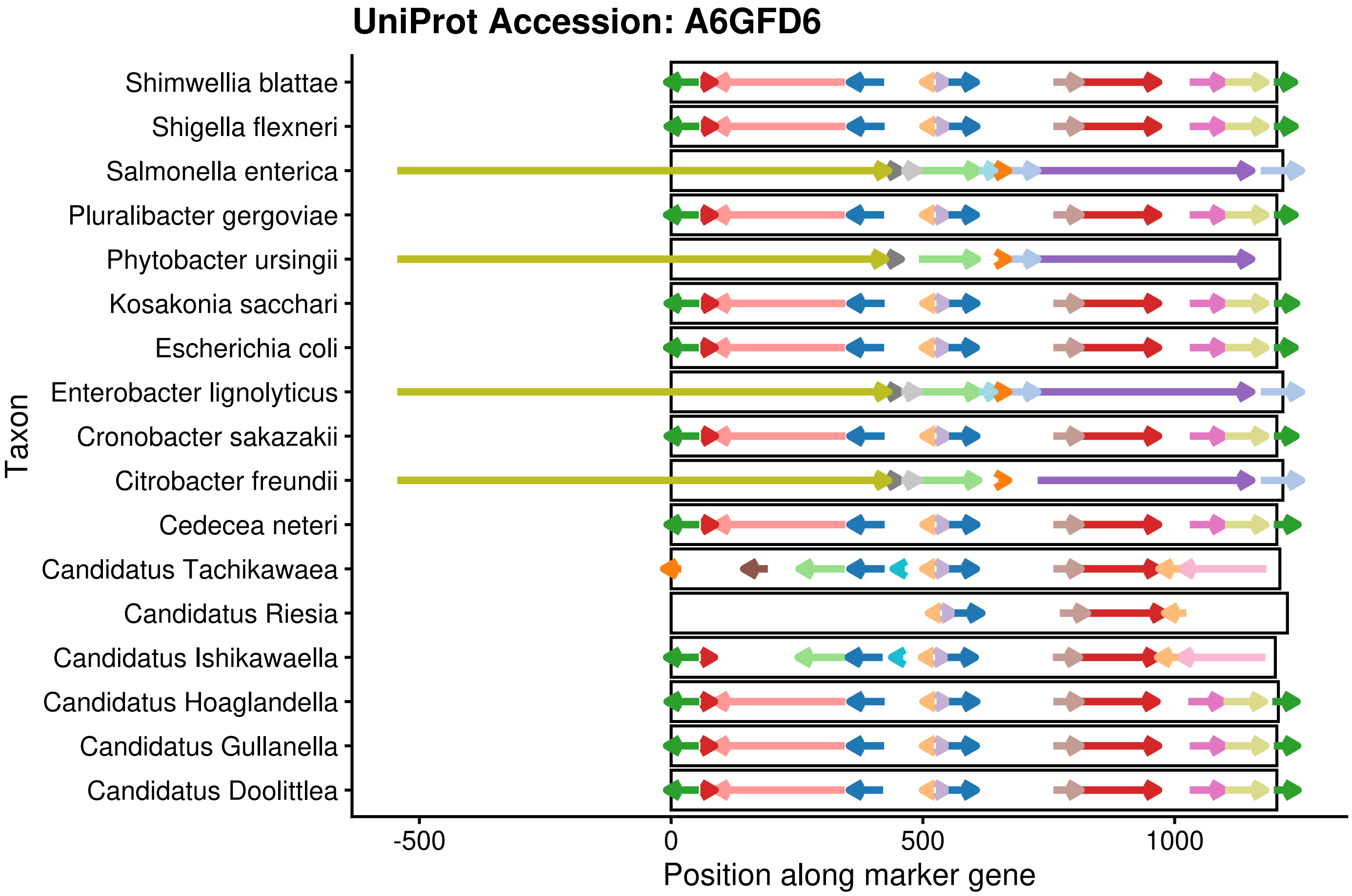
UniProt Accession: A6GFD6
Shimwellia blattae
Shigella flexneri
Salmonella enterica
Pluralibacter gergoviae
Phytobacter ursingii
Kosakonia sacchari
Escherichia coli
Enterobacter lignolyticus
Cronobacter sakazakii
Citrobacter freundii
Cedecea neteri
Candidatus Tachikawaea
Candidatus Riesia
Candidatus Ishikawaella
Candidatus Hoaglandella
Candidatus Gullanella
Candidatus Doolittlea
Taxon
-500
0
500
1000
Position along marker gene

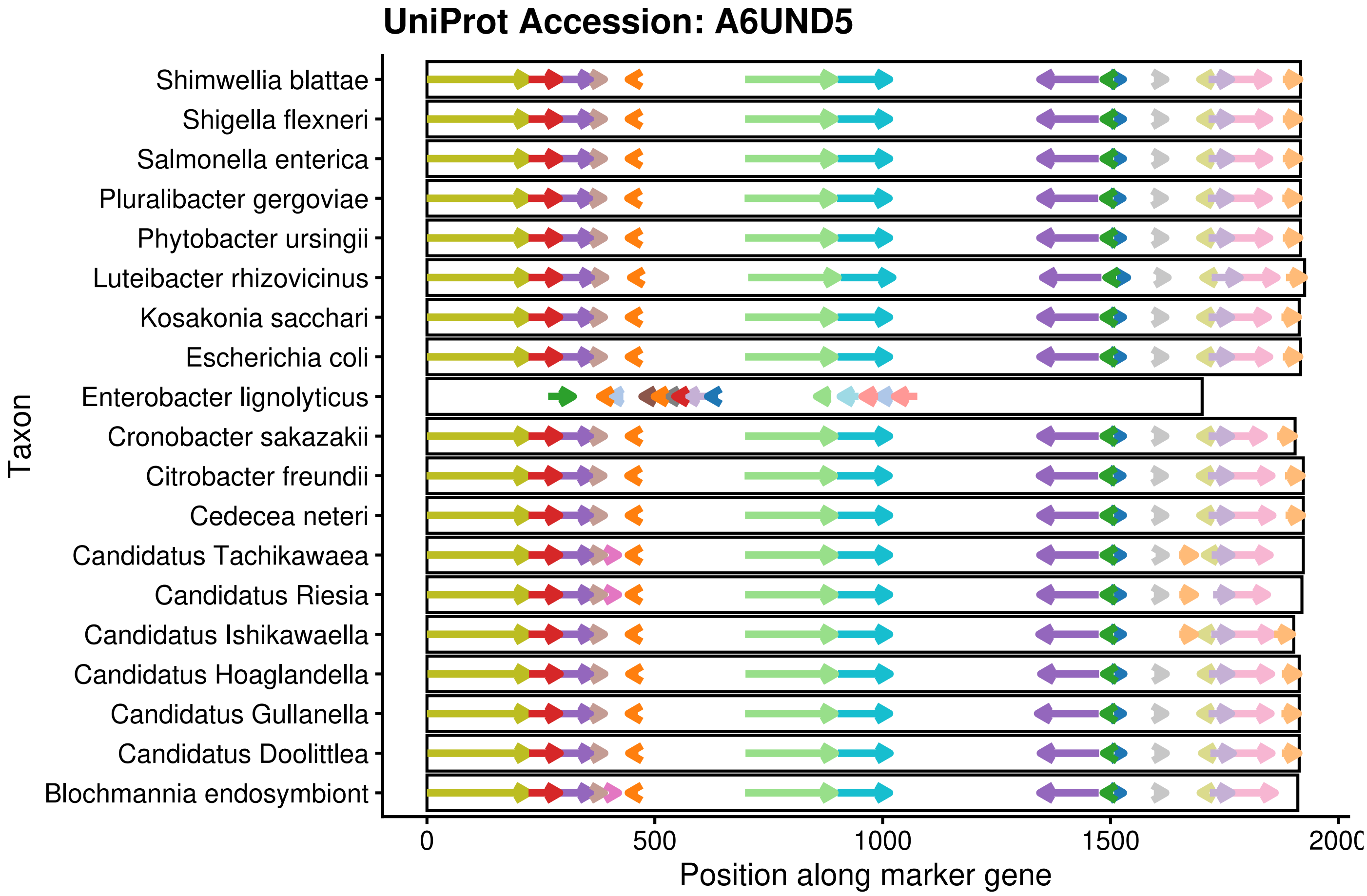
UniProt Accession: A6UND5
Shimwellia blattae
Shigella flexneri
Salmonella enterica
Pluralibacter gergoviae
Phytobacter ursingii
Luteibacter rhizovicinus
Kosakonia sacchari
Escherichia coli
Enterobacter lignolyticus
Cronobacter sakazakii
Citrobacter freundii
Cedecea neteri
Candidatus Tachikawaea
Candidatus Riesia
Candidatus Ishikawaella
Candidatus Hoaglandella
Candidatus Gullanella
Candidatus Doolittlea
Blochmannia endosymbiont
Taxon
0
500
1000
1500
2000
Position along marker gene

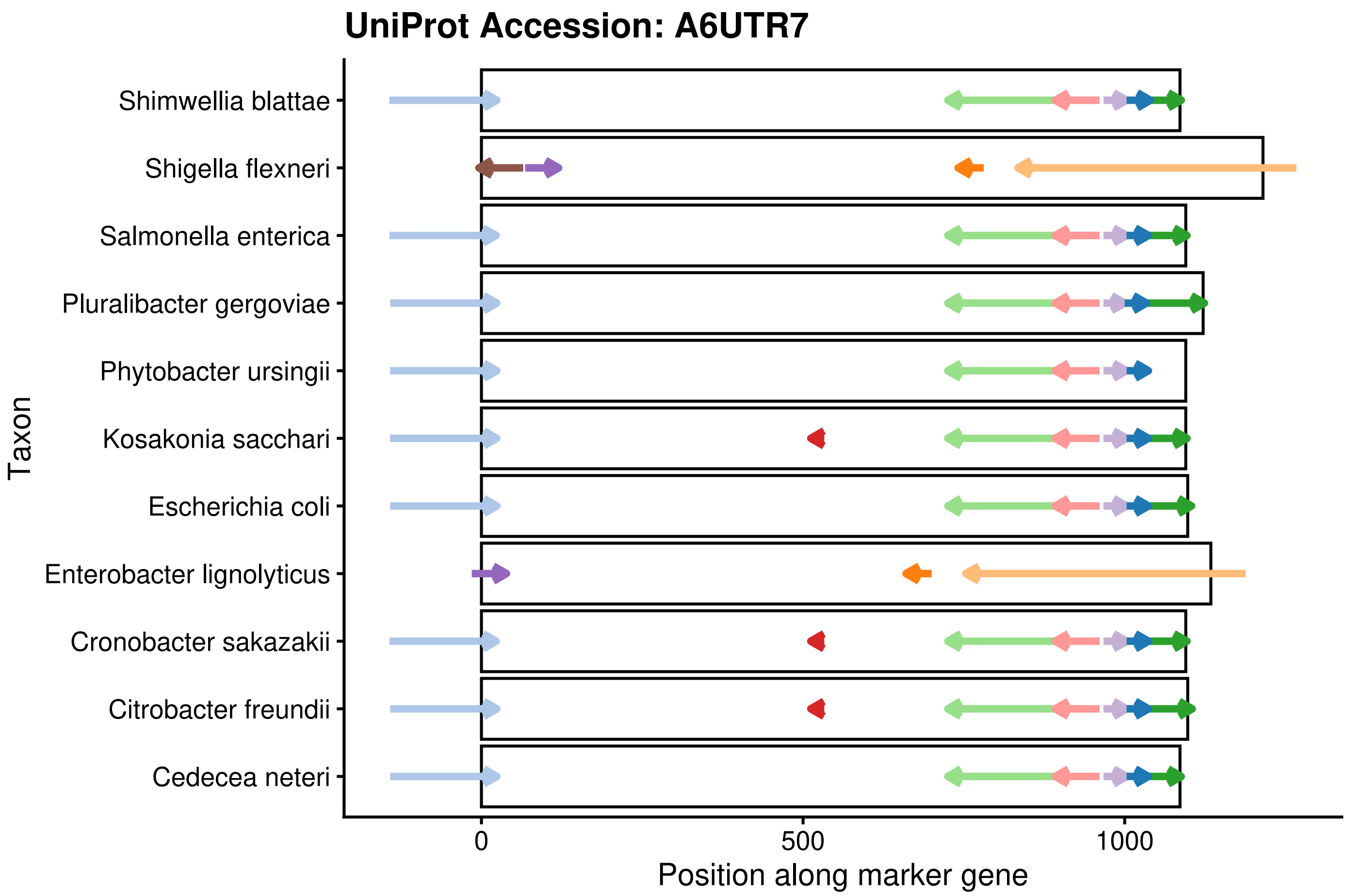

UniProt Accession: A6UTR7
Shimwellia blattae
Shigella flexneri
Salmonella enterica
Pluralibacter gergoviae
Phytobacter ursingii
Kosakonia sacchari
Escherichia coli
Enterobacter lignolyticus
Cronobacter sakazakii
Citrobacter freundii
Cedecea neteri
Taxon
0
500
1000
Position along marker gene

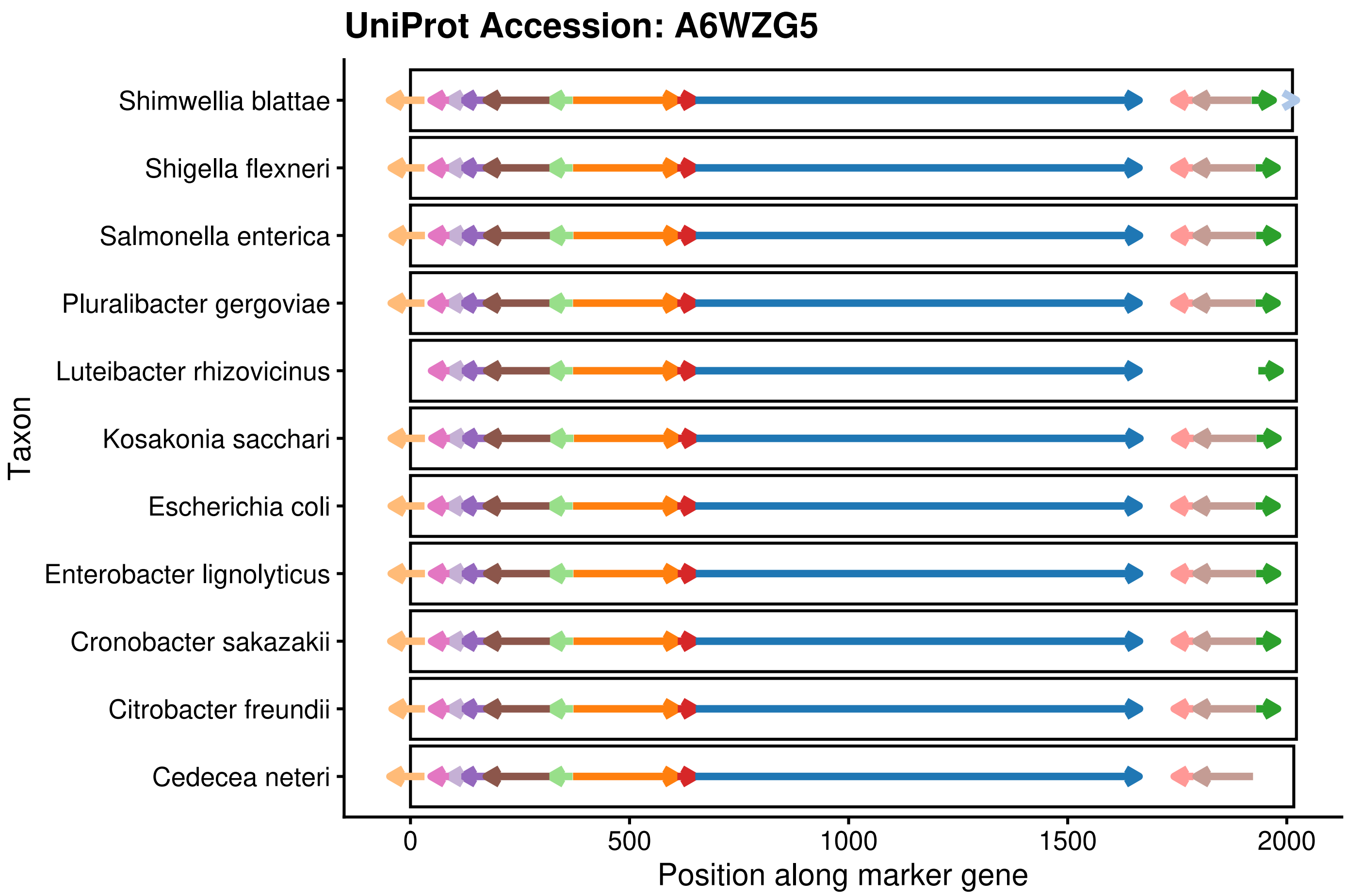

UniProt Accession: A6WZG5
Shimwellia blattae
Shigella flexneri
Salmonella enterica
Pluralibacter gergoviae
Luteibacter rhizovicinus
Kosakonia sacchari
Escherichia coli
Enterobacter lignolyticus
Cronobacter sakazakii
Citrobacter freundii
Cedecea neteri
Taxon
0
500
1000
1500
2000
Position along marker gene

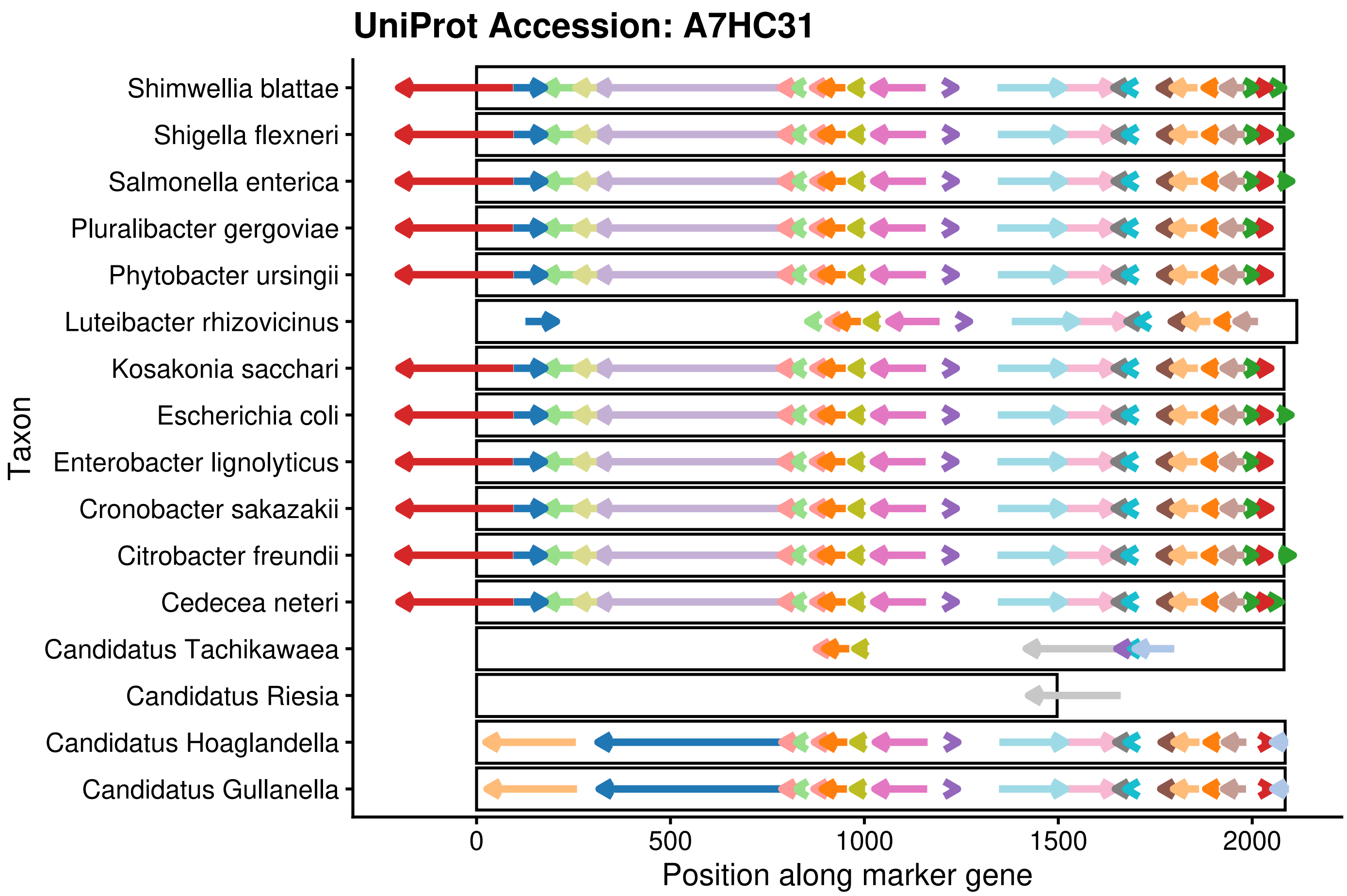
UniProt Accession: A7HC31
Taxon
Shimwellia blattae
Shigella flexneri
Salmonella enterica
Pluralibacter gergoviae
Phytobacter ursingii
Luteibacter rhizovicinus
Kosakonia sacchari
Escherichia coli
Enterobacter lignolyticus
Cronobacter sakazakii
Citrobacter freundii
Cedecea neteri
Candidatus Tachikawaea
Candidatus Riesia
Candidatus Hoaglandella
Candidatus Gullanella
0
500
1000
1500
2000
Position along marker gene

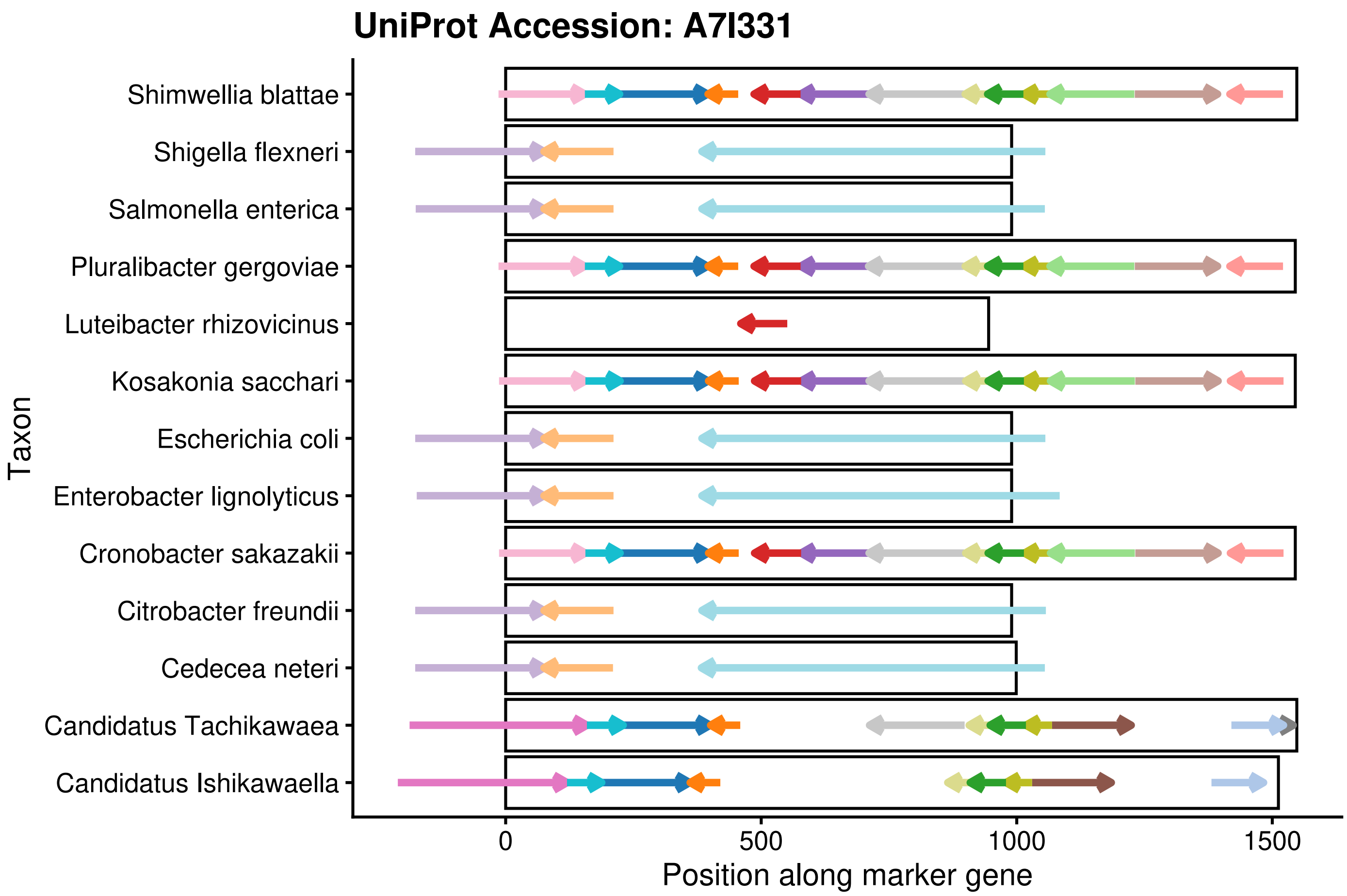
UniProt Accession: A7I331
Shimwellia blattae
Shigella flexneri
Salmonella enterica
Pluralibacter gergoviae
Luteibacter rhizovicinus
Kosakonia sacchari
Escherichia coli
Enterobacter lignolyticus
Cronobacter sakazakii
Citrobacter freundii
Cedecea neteri
Candidatus Tachikawaea
Candidatus Ishikawaella
Taxon
0
500
1000
1500
Position along marker gene

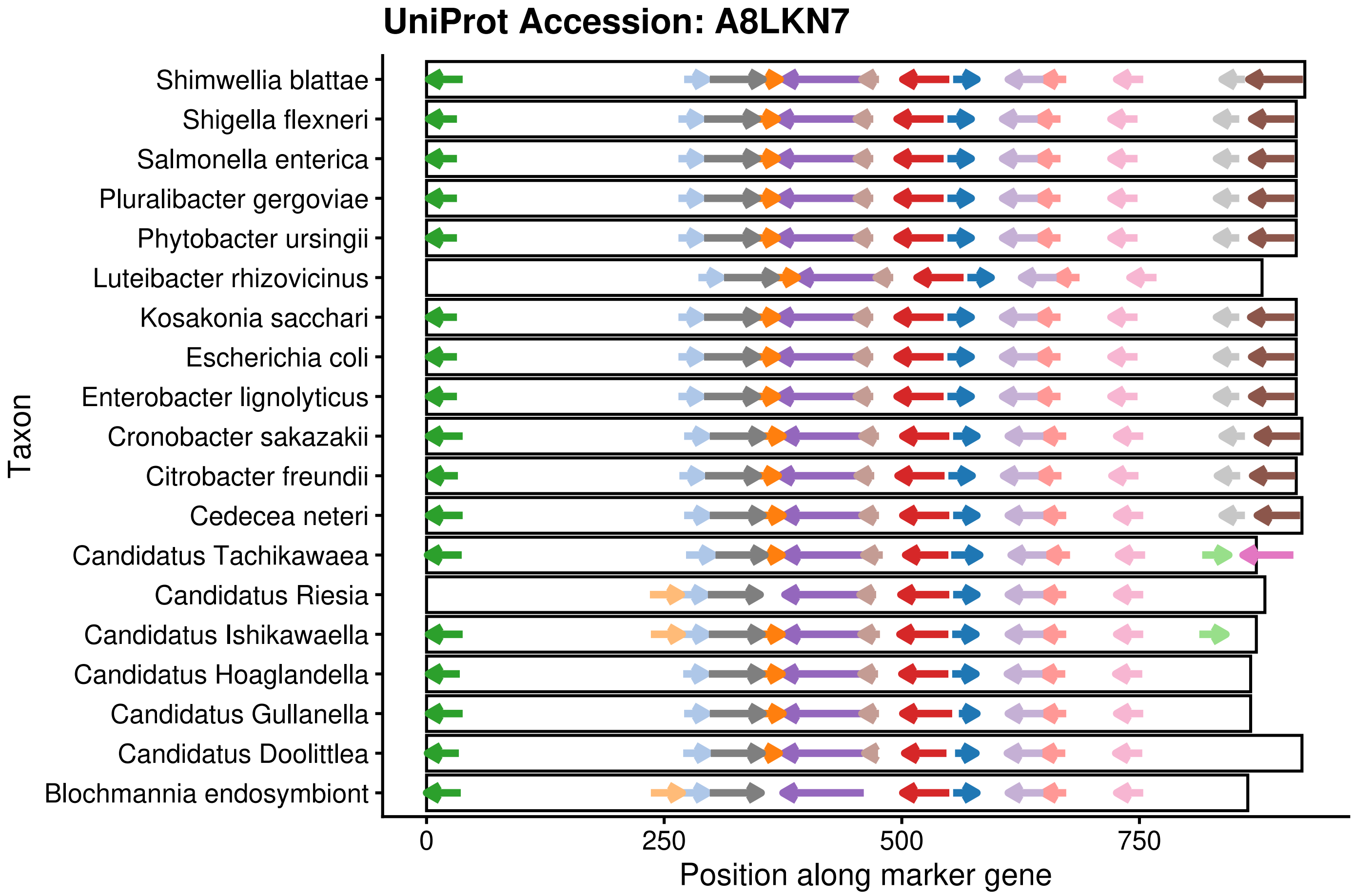

UniProt Accession: A8LKN7
Shimwellia blattae
Shigella flexneri
Salmonella enterica
Pluralibacter gergoviae
Phytobacter ursingii
Luteibacter rhizovicinus
Kosakonia sacchari
Escherichia coli
Enterobacter lignolyticus
Cronobacter sakazakii
Citrobacter freundii
Cedecea neteri
Candidatus Tachikawaea
Candidatus Riesia
Candidatus Ishikawaella
Candidatus Hoaglandella
Candidatus Gullanella
Candidatus Doolittlea
Blochmannia endosymbiont
Taxon
0
250
500
750
Position along marker gene

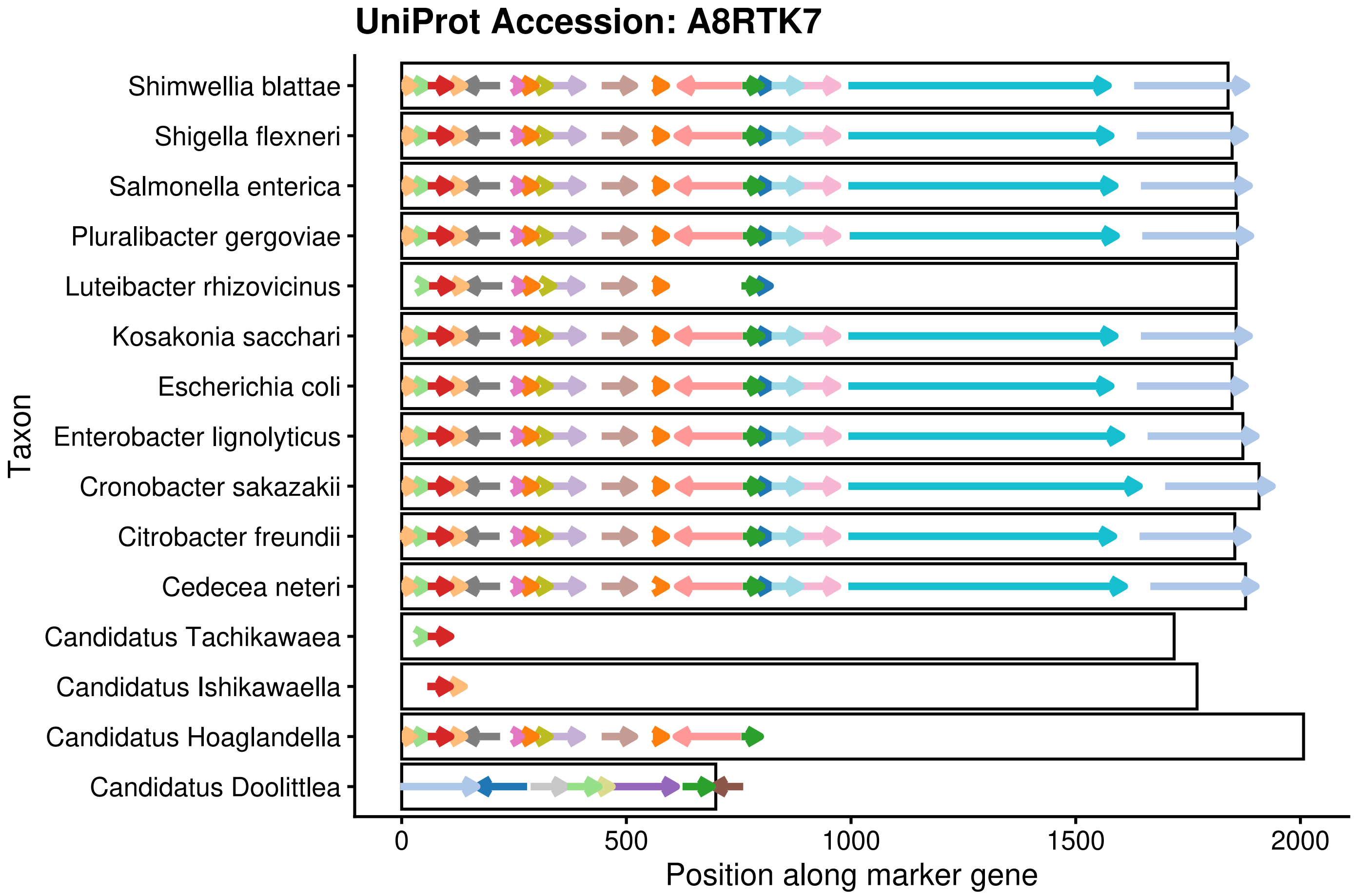

UniProt Accession: A8RTK7
Shimwellia blattae
Shigella flexneri
Salmonella enterica
Pluralibacter gergoviae
Luteibacter rhizovicinus
Kosakonia sacchari
Escherichia coli
Enterobacter lignolyticus
Cronobacter sakazakii
Citrobacter freundii
Cedecea neteri
Candidatus Tachikawaea
Candidatus Ishikawaella
Candidatus Hoaglandella
Candidatus Doolittlea
Taxon
0
500
1000
1500
2000
Position along marker gene

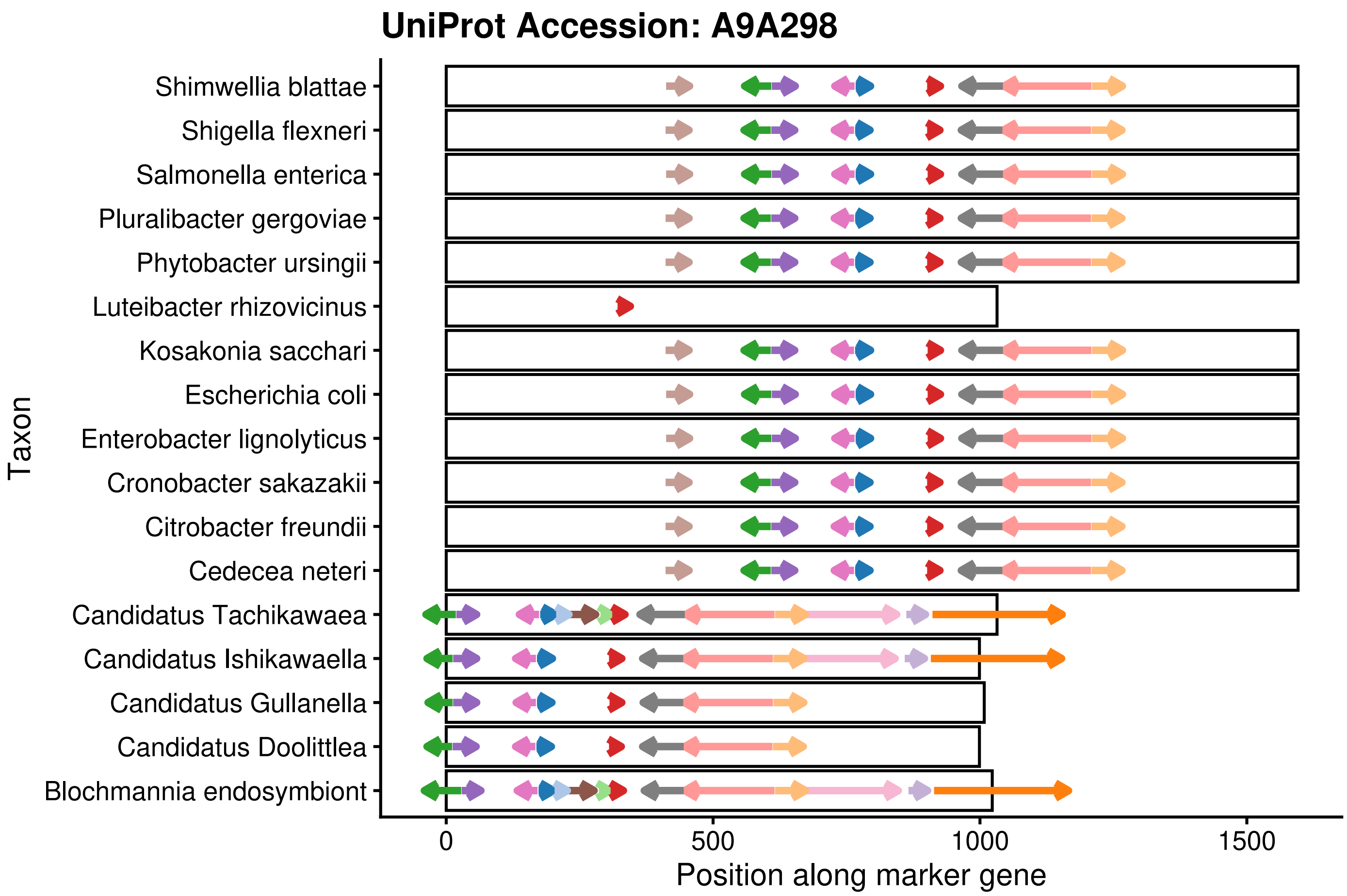

UniProt Accession: A9A298
Shimwellia blattae
Shigella flexneri
Salmonella enterica
Pluralibacter gergoviae
Phytobacter ursingii
Luteibacter rhizovicinus
Kosakonia sacchari
Escherichia coli
Enterobacter lignolyticus
Cronobacter sakazakii
Citrobacter freundii
Cedecea neteri
Candidatus Tachikawaea
Candidatus Ishikawaella
Candidatus Gullanella
Candidatus Doolittlea
Blochmannia endosymbiont
Taxon
0
500
1000
1500
Position along marker gene

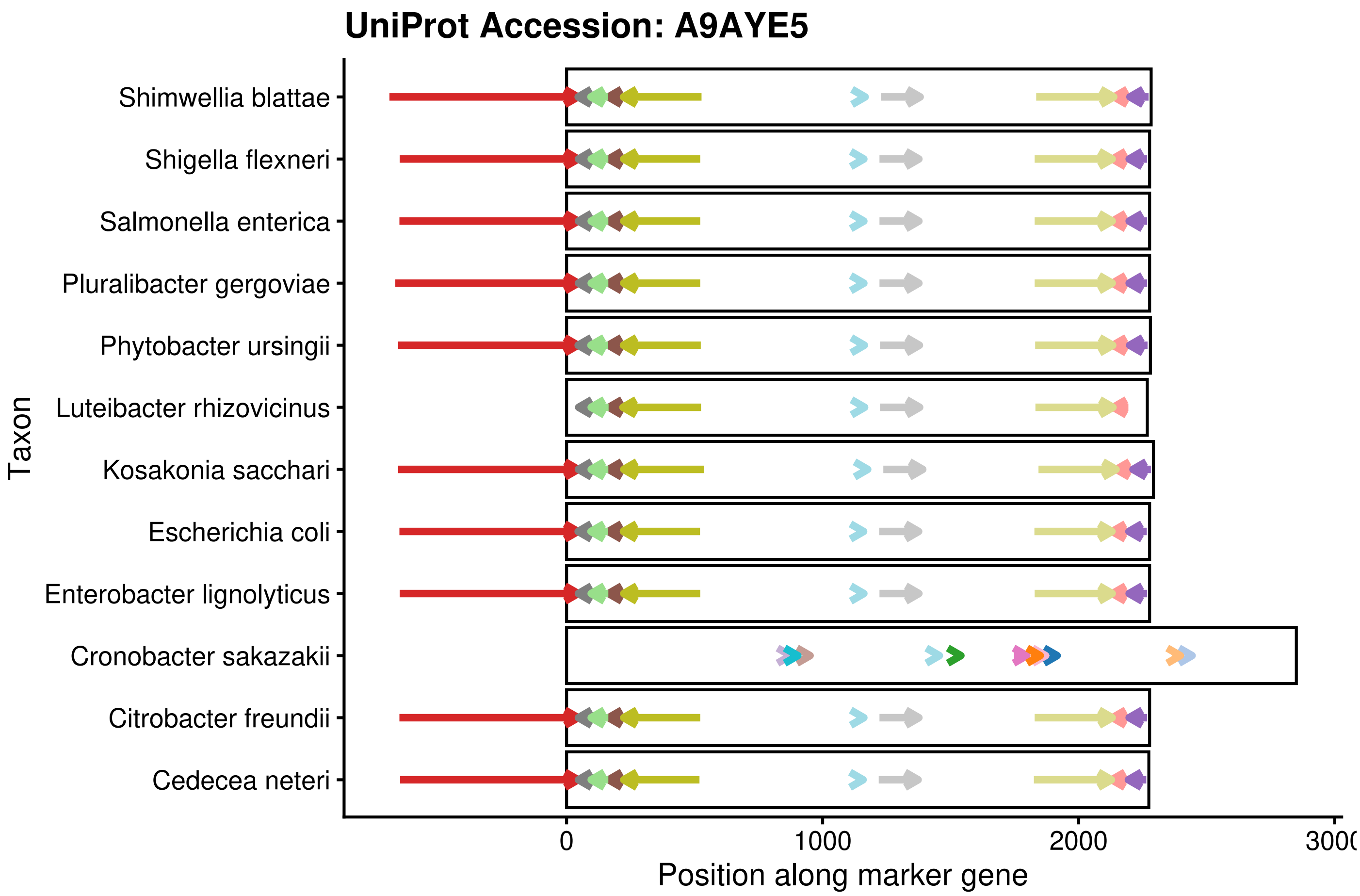
UniProt Accession: A9AYE5
Taxon
Shimwellia blattae
Shigella flexneri
Salmonella enterica
Pluralibacter gergoviae
Phytobacter ursingii
Luteibacter rhizovicinus
Kosakonia sacchari
Escherichia coli
Enterobacter lignolyticus
Cronobacter sakazakii
Citrobacter freundii
Cedecea neteri
0
1000
2000
Position along marker gene

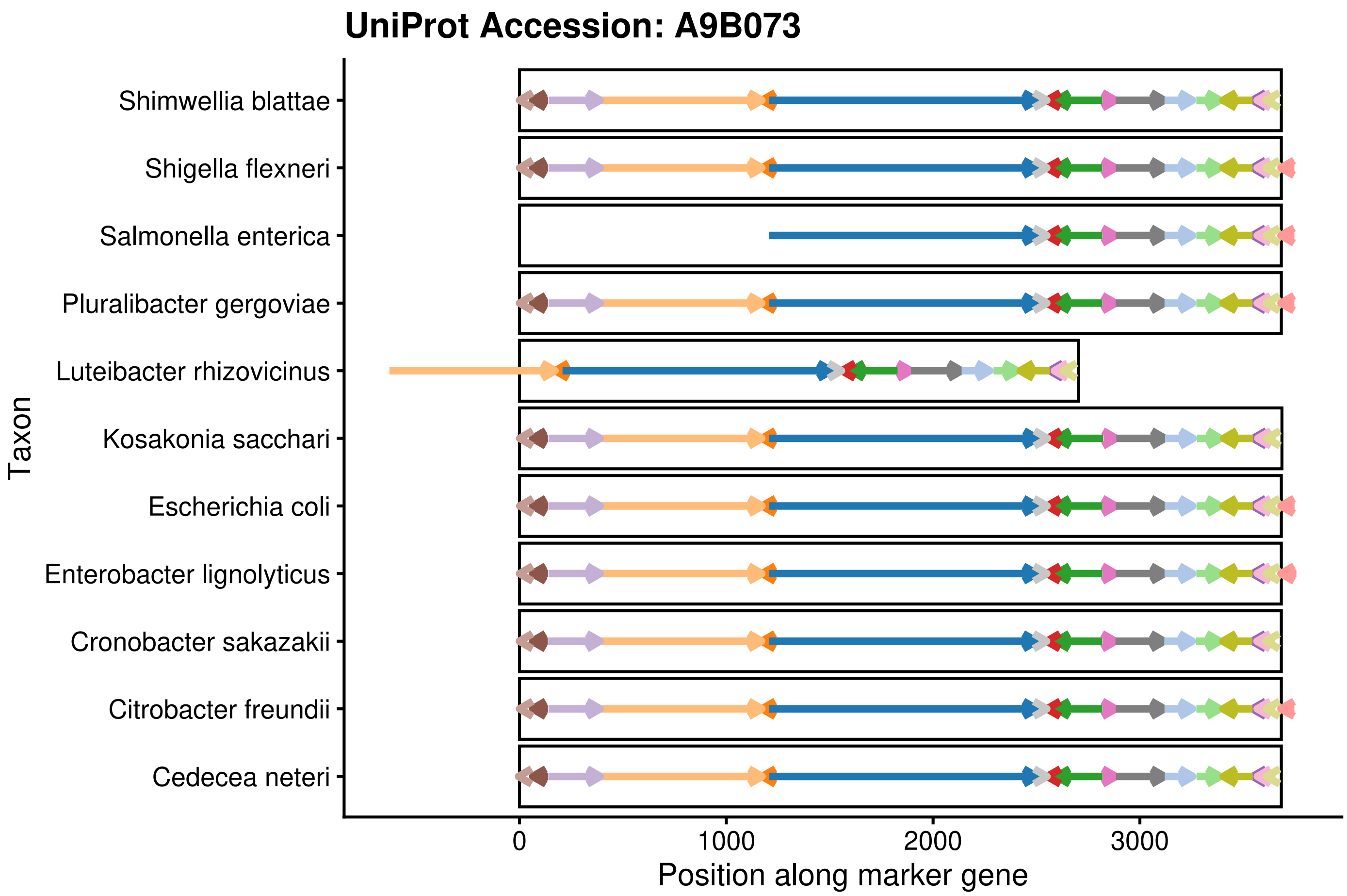
UniProt Accession: A9B073
Shimwellia blattae
Shigella flexneri
Salmonella enterica
Pluralibacter gergoviae
Luteibacter rhizovicinus
Kosakonia sacchari
Escherichia coli
Enterobacter lignolyticus
Cronobacter sakazakii
Citrobacter freundii
Cedecea neteri
Taxon
0
1000
2000
3000
Position along marker gene

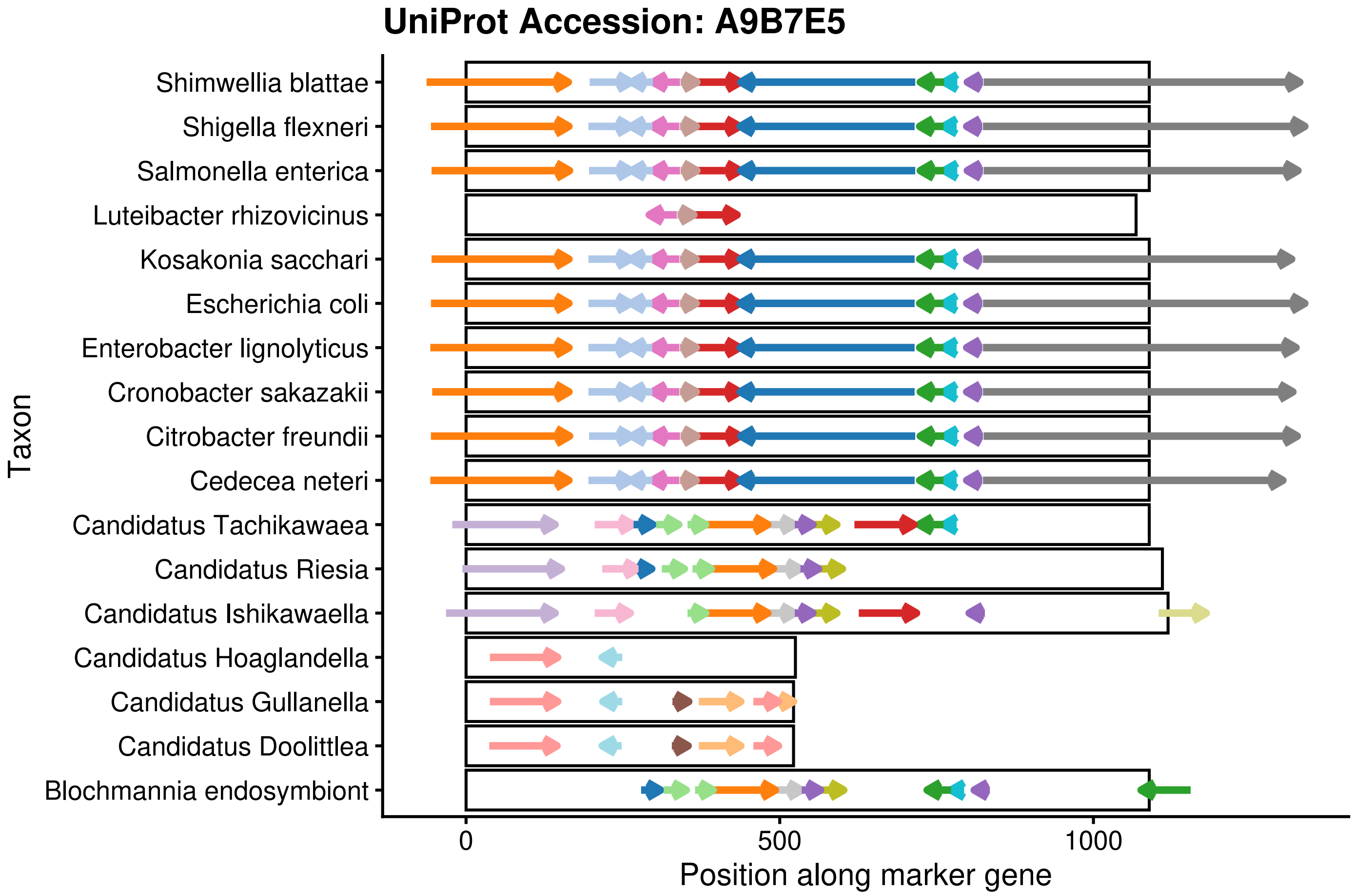

UniProt Accession: A9B7E5
Taxon
Shimwellia blattae
Shigella flexneri
Salmonella enterica
Luteibacter rhizovicinus
Kosakonia sacchari
Escherichia coli
Enterobacter lignolyticus
Cronobacter sakazakii
Citrobacter freundii
Cedecea neteri
Candidatus Tachikawaea
Candidatus Riesia
Candidatus Ishikawaella
Candidatus Hoaglandella
Candidatus Gullanella
Candidatus Doolittlea
Blochmannia endosymbiont
0
500
1000
Position along marker gene

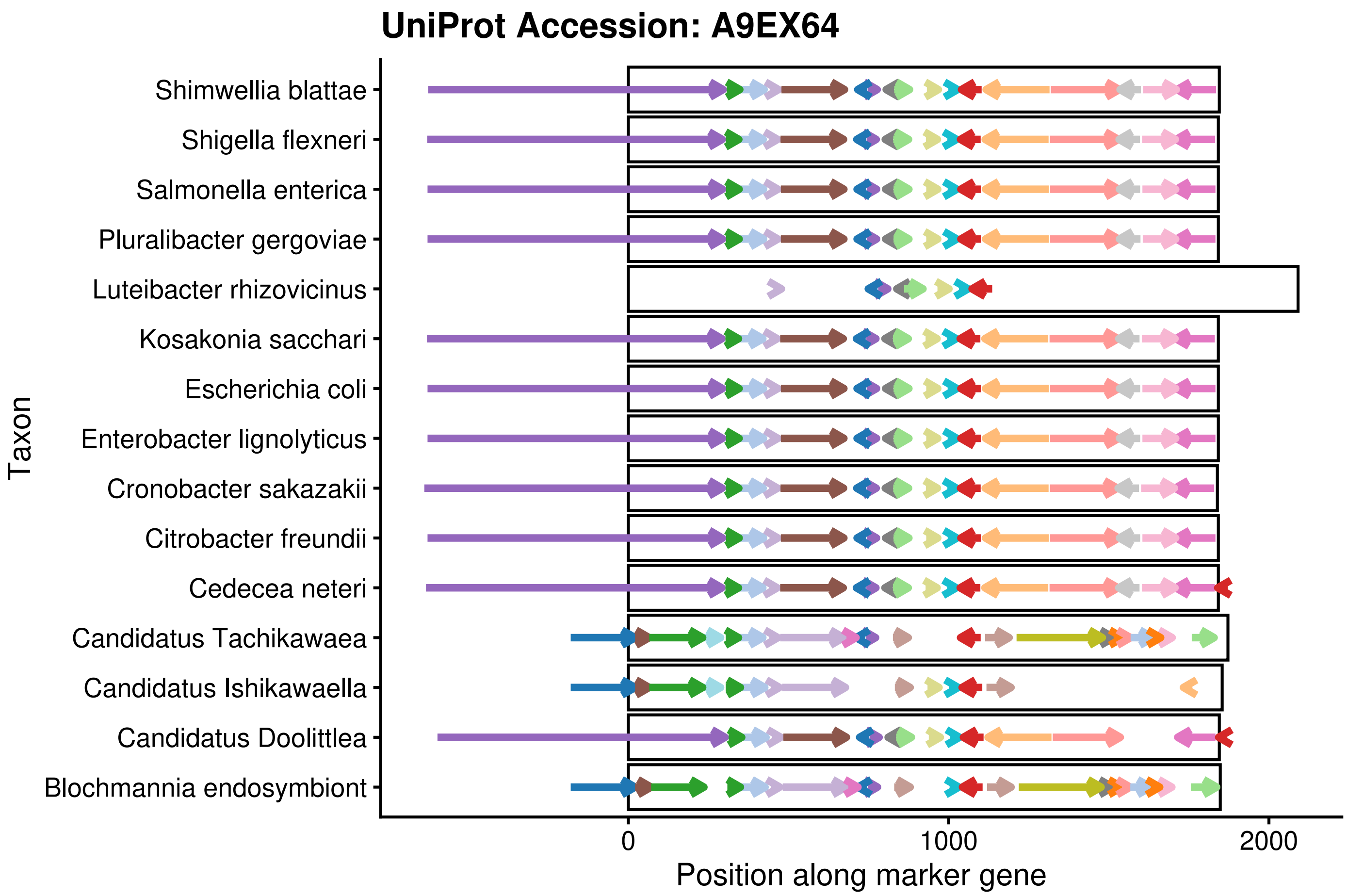
UniProt Accession: A9EX64
Shimwellia blattae
Shigella flexneri
Salmonella enterica
Pluralibacter gergoviae
Luteibacter rhizovicinus
Kosakonia sacchari
Escherichia coli
Enterobacter lignolyticus
Cronobacter sakazakii
Citrobacter freundii
Cedecea neteri
Candidatus Tachikawaea
Candidatus Ishikawaella
Candidatus Doolittlea
Blochmannia endosymbiont
Taxon
0
1000
2000
Position along marker gene

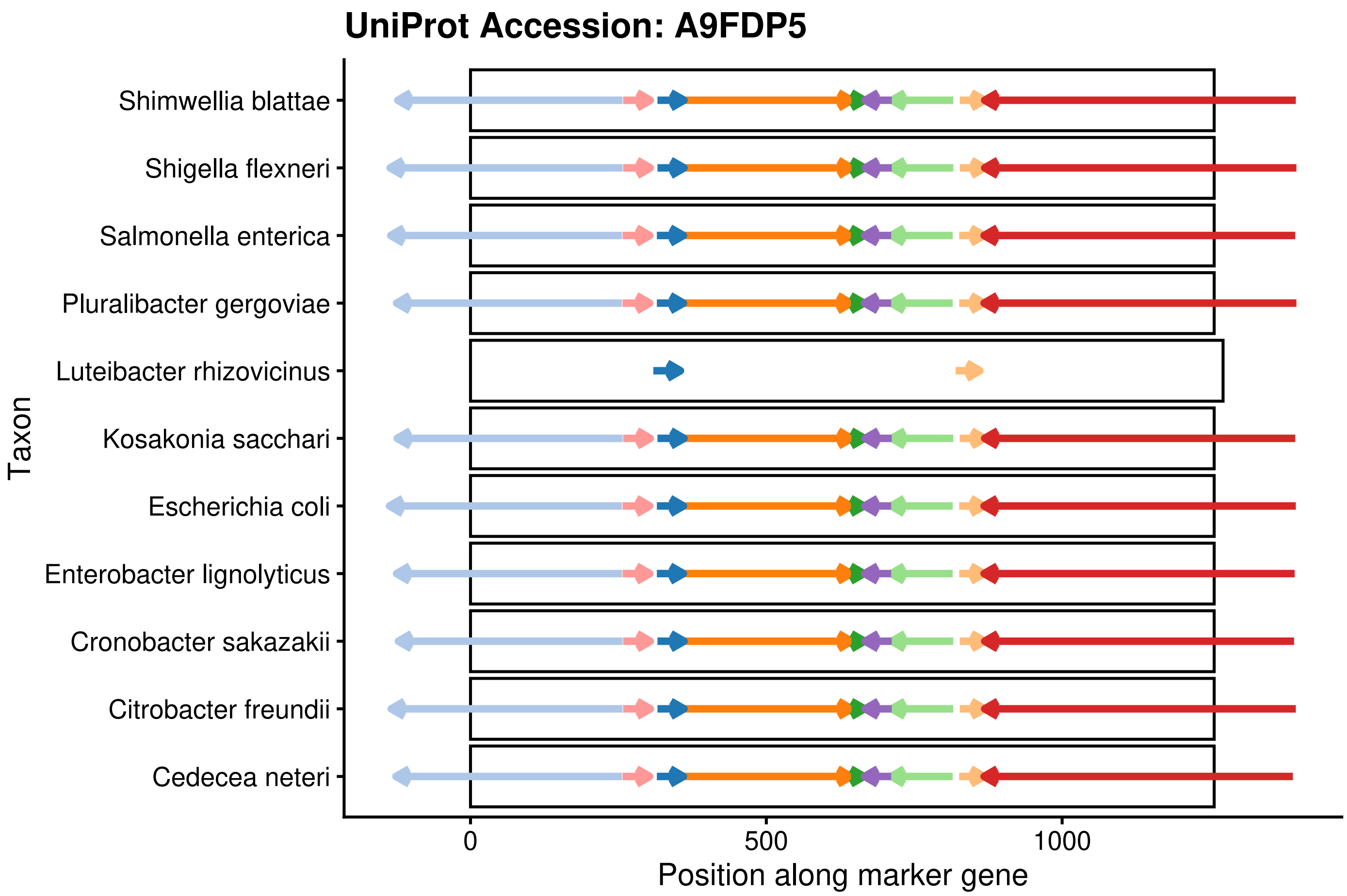
UniProt Accession: A9FDP5
Taxon
Shimwellia blattae
Shigella flexneri
Salmonella enterica
Pluralibacter gergoviae
Luteibacter rhizovicinus
Kosakonia sacchari
Escherichia coli
Enterobacter lignolyticus
Cronobacter sakazakii
Citrobacter freundii
Cedecea neteri
0
500
1000
Position along marker gene

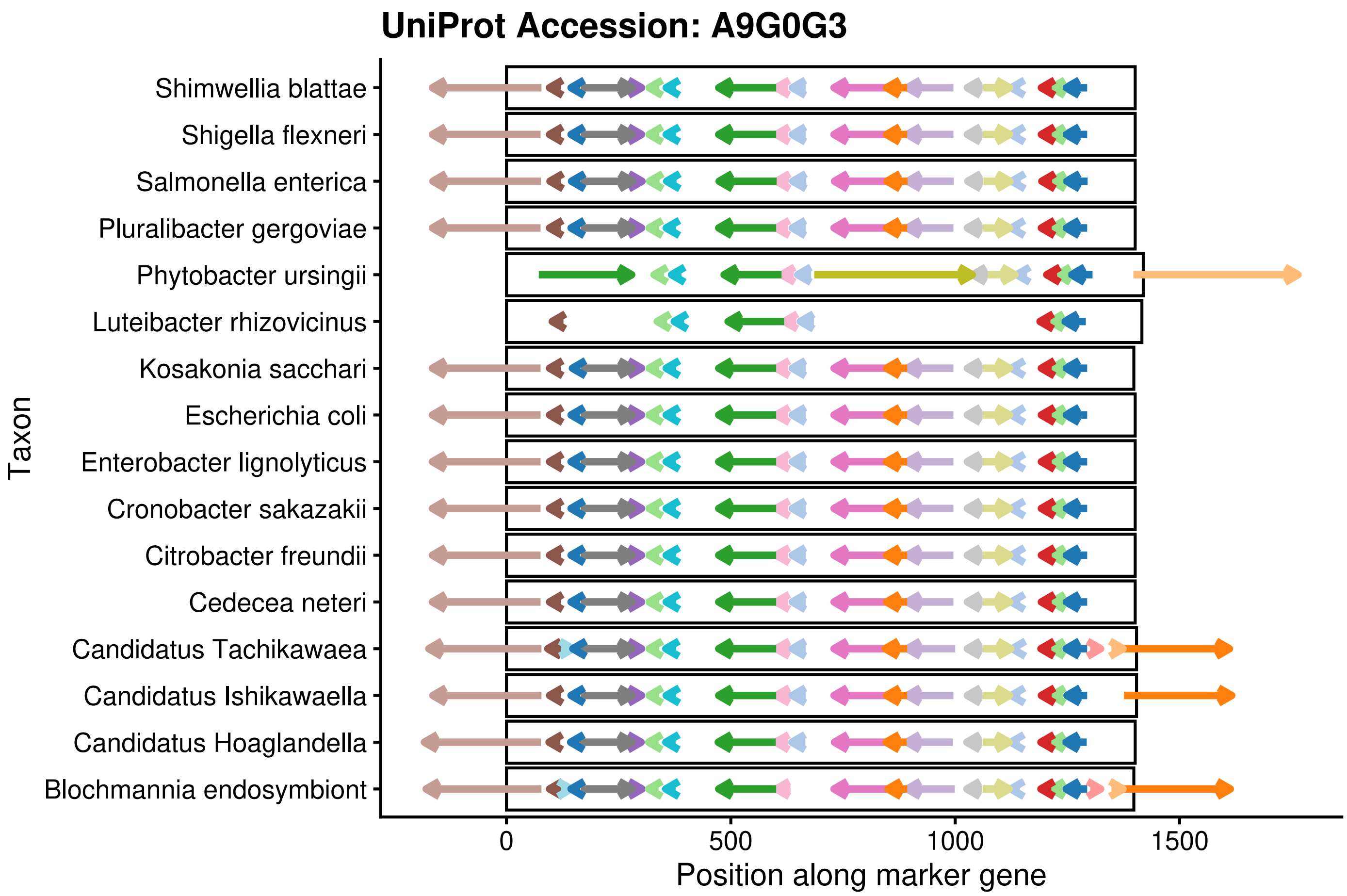
UniProt Accession: A9G0G3
Shimwellia blattae
Shigella flexneri
Salmonella enterica
Pluralibacter gergoviae
Phytobacter ursingii
Luteibacter rhizovicinus
Kosakonia sacchari
Escherichia coli
Enterobacter lignolyticus
Cronobacter sakazakii
Citrobacter freundii
Cedecea neteri
Candidatus Tachikawaea
Candidatus Ishikawaella
Candidatus Hoaglandella
Blochmannia endosymbiont
Taxon
0
500
1000
1500
Position along marker gene

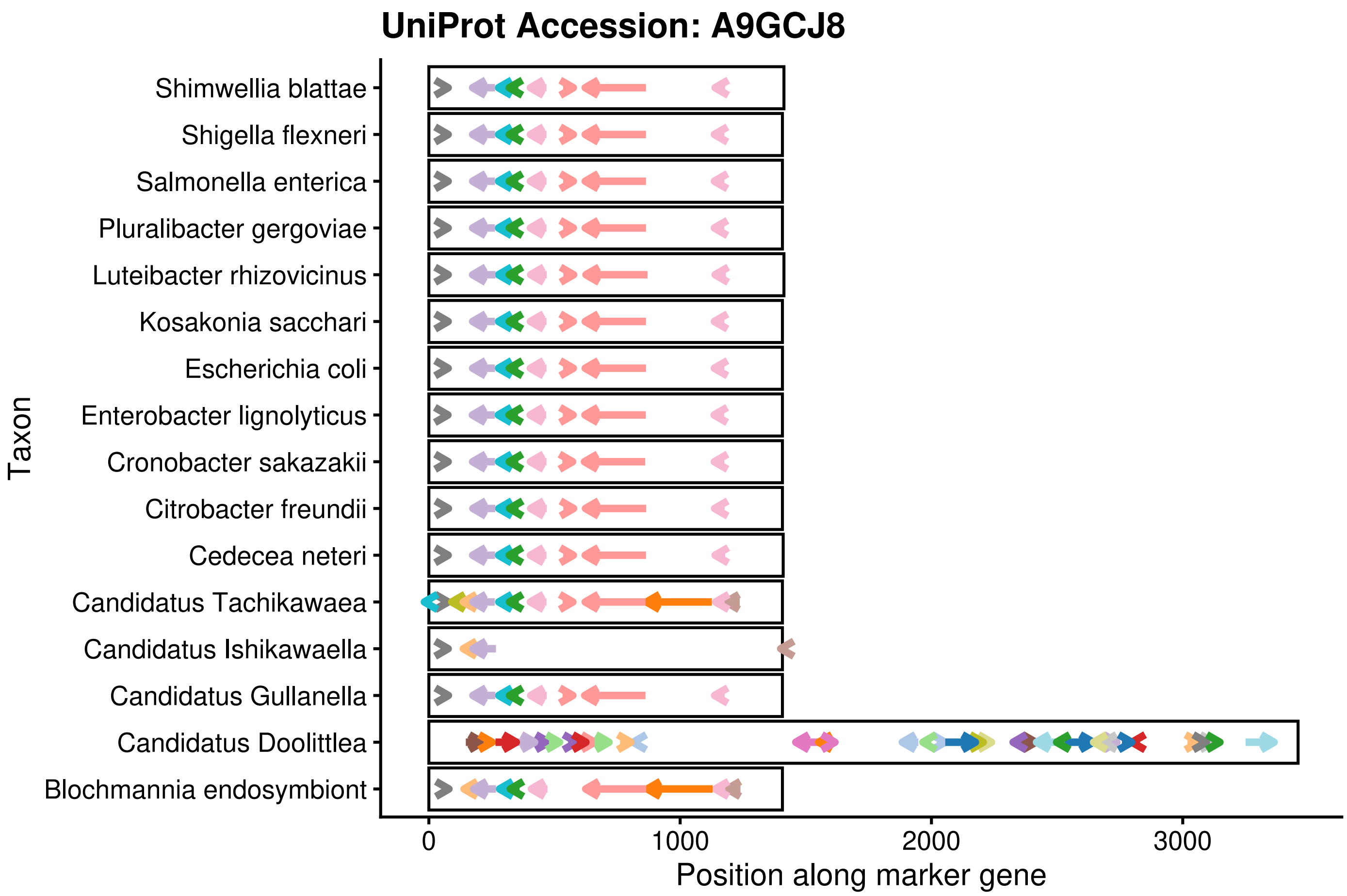
UniProt Accession: A9GCJ8
Taxon
Shimwellia blattae
Shigella flexneri
Salmonella enterica
Pluralibacter gergoviae
Luteibacter rhizovicinus
Kosakonia sacchari
Escherichia coli
Enterobacter lignolyticus
Cronobacter sakazakii
Citrobacter freundii
Cedecea neteri
Candidatus Tachikawaea
Candidatus Ishikawaella
Candidatus Gullanella
Candidatus Doolittlea
Blochmannia endosymbiont
0
1000
2000
3000
Position along marker gene

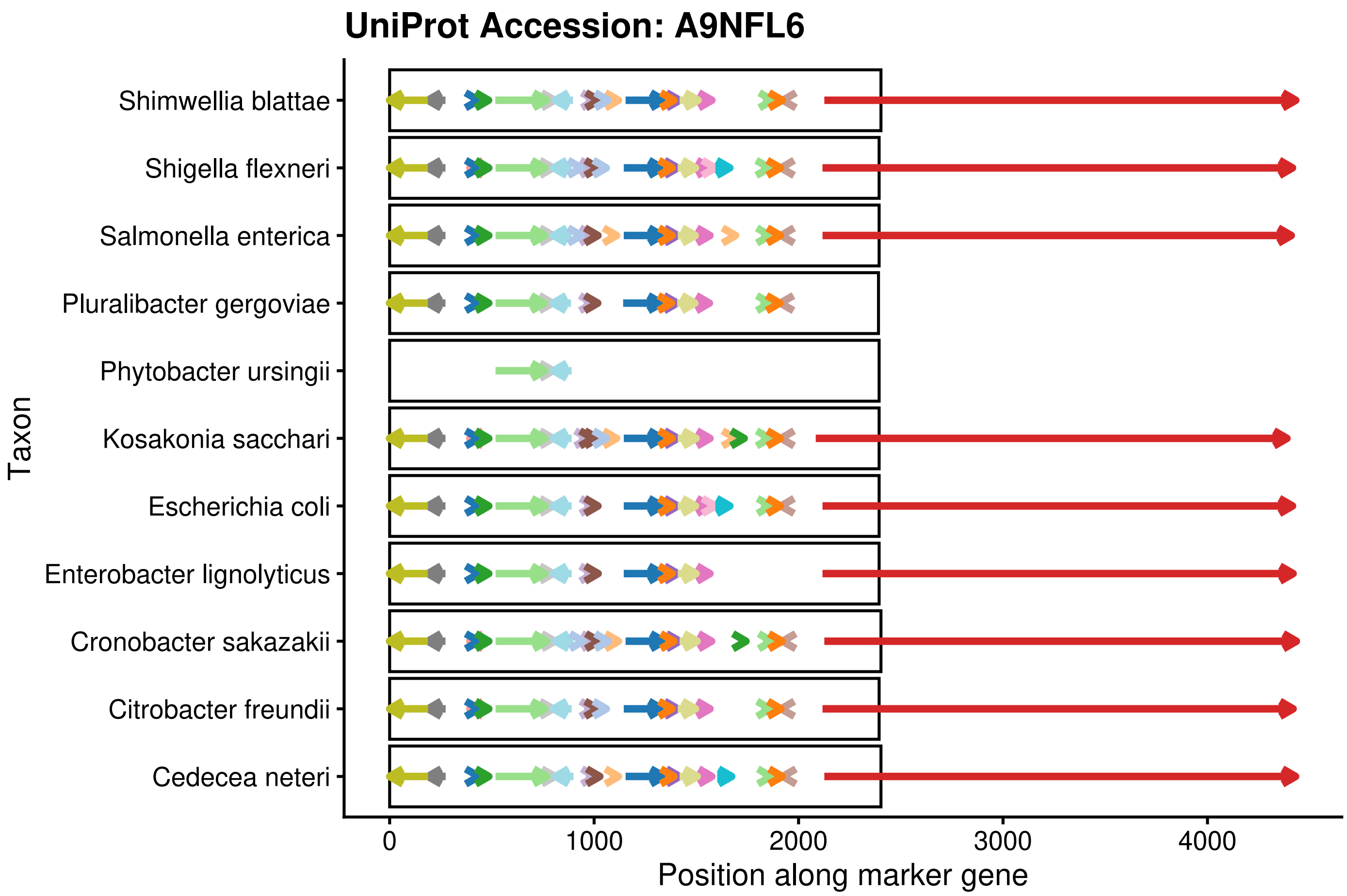
UniProt Accession: A9NFL6
Shimwellia blattae
Shigella flexneri
Salmonella enterica
Pluralibacter gergoviae
Phytobacter ursingii
Kosakonia sacchari
Escherichia coli
Enterobacter lignolyticus
Cronobacter sakazakii
Citrobacter freundii
Cedecea neteri
Taxon
0
1000
2000
3000
4000
Position along marker gene

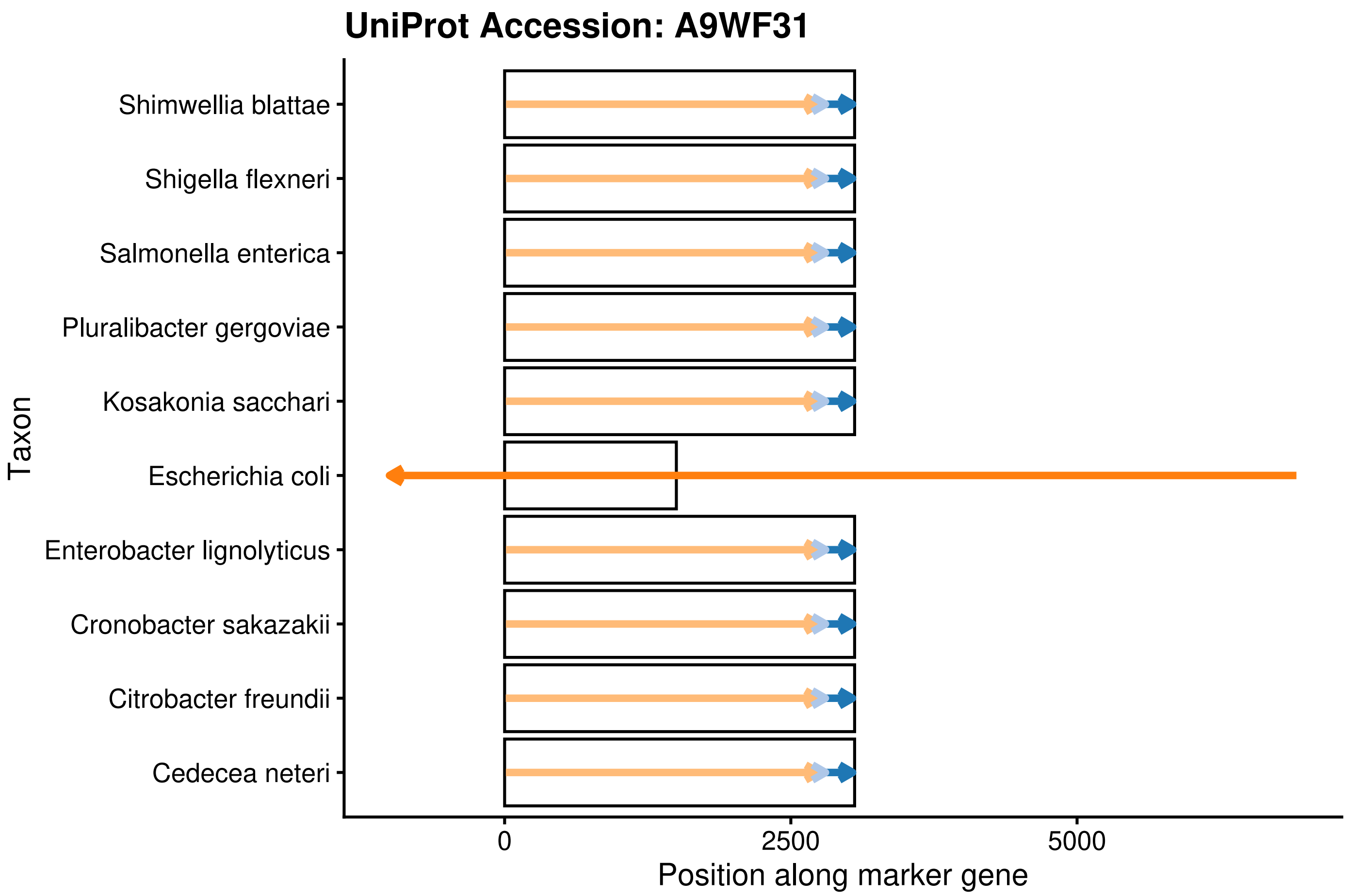
UniProt Accession: A9WF31
Shimwellia blattae
Shigella flexneri
Salmonella enterica
Pluralibacter gergoviae
Kosakonia sacchari
Escherichia coli
Enterobacter lignolyticus
Cronobacter sakazakii
Citrobacter freundii
Cedecea neteri
Taxon
0
2500
5000
Position along marker gene

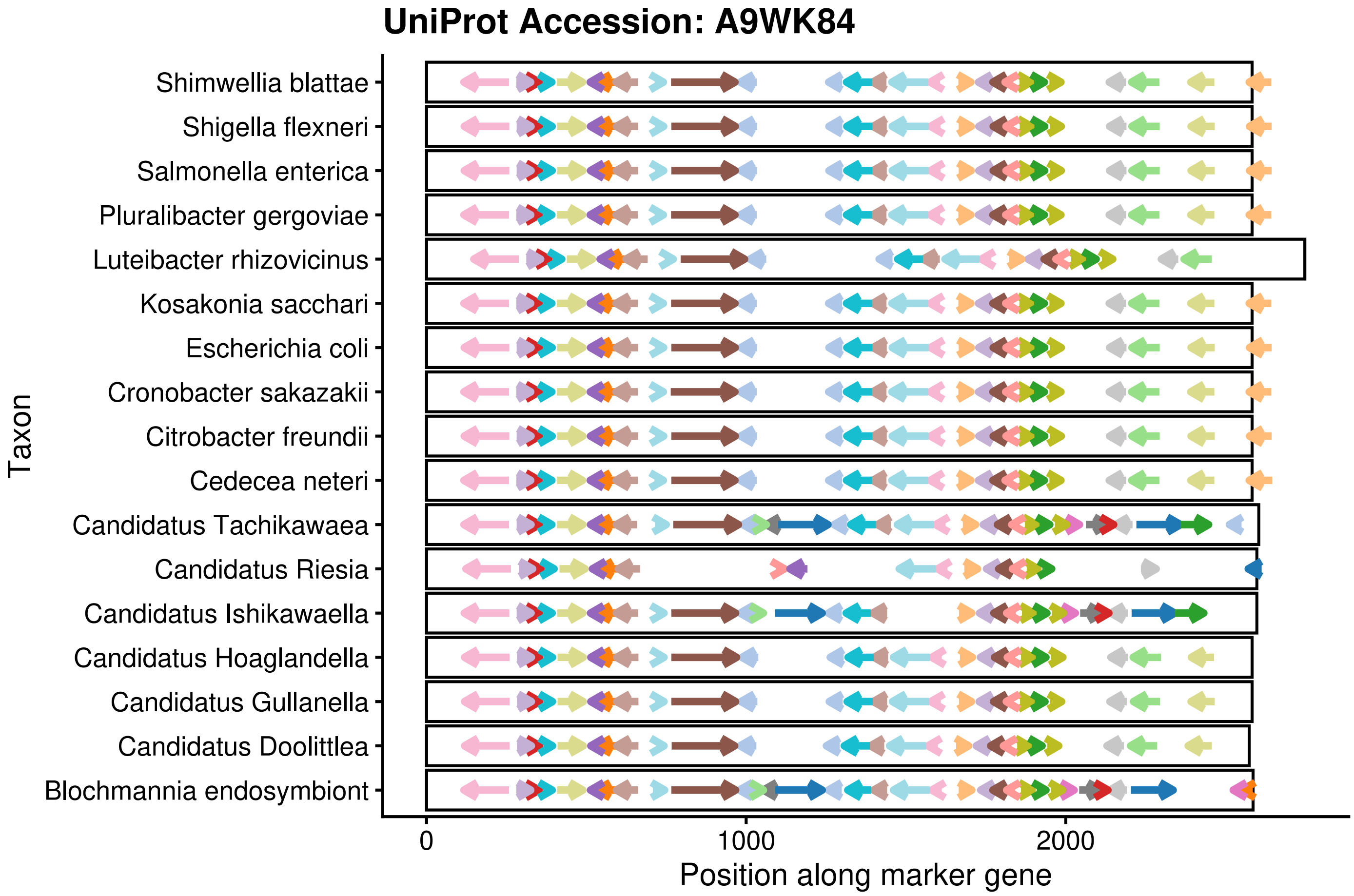
UniProt Accession: A9WK84
Taxon
Shimwellia blattae
Shigella flexneri
Salmonella enterica
Pluralibacter gergoviae
Luteibacter rhizovicinus
Kosakonia sacchari
Escherichia coli
Cronobacter sakazakii
Citrobacter freundii
Cedecea neteri
Candidatus Tachikawaea
Candidatus Riesia
Candidatus Ishikawaella
Candidatus Hoaglandella
Candidatus Gullanella
Candidatus Doolittlea
Blochmannia endosymbiont
0
1000
2000
Position along marker gene

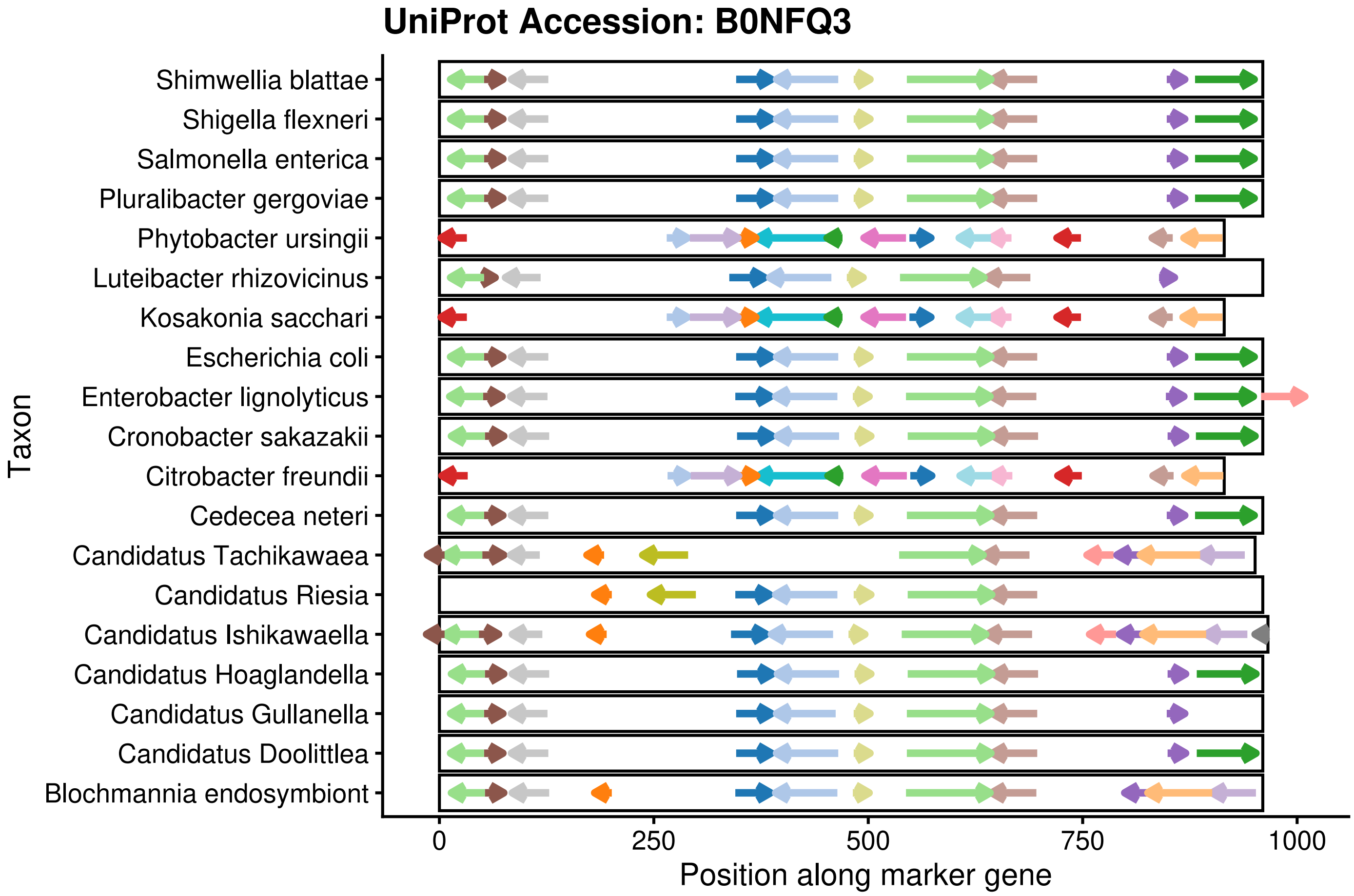
UniProt Accession: B0NFQ3
Shimwellia blattae
Shigella flexneri
Salmonella enterica
Pluralibacter gergoviae
Phytobacter ursingii
Luteibacter rhizovicinus
Kosakonia sacchari
Escherichia coli
Enterobacter lignolyticus
Cronobacter sakazakii
Citrobacter freundii
Cedecea neteri
Candidatus Tachikawaea
Candidatus Riesia
Candidatus Ishikawaella
Candidatus Hoaglandella
Candidatus Gullanella
Candidatus Doolittlea
Blochmannia endosymbiont
Taxon
0
250
500
750
1000
Position along marker gene

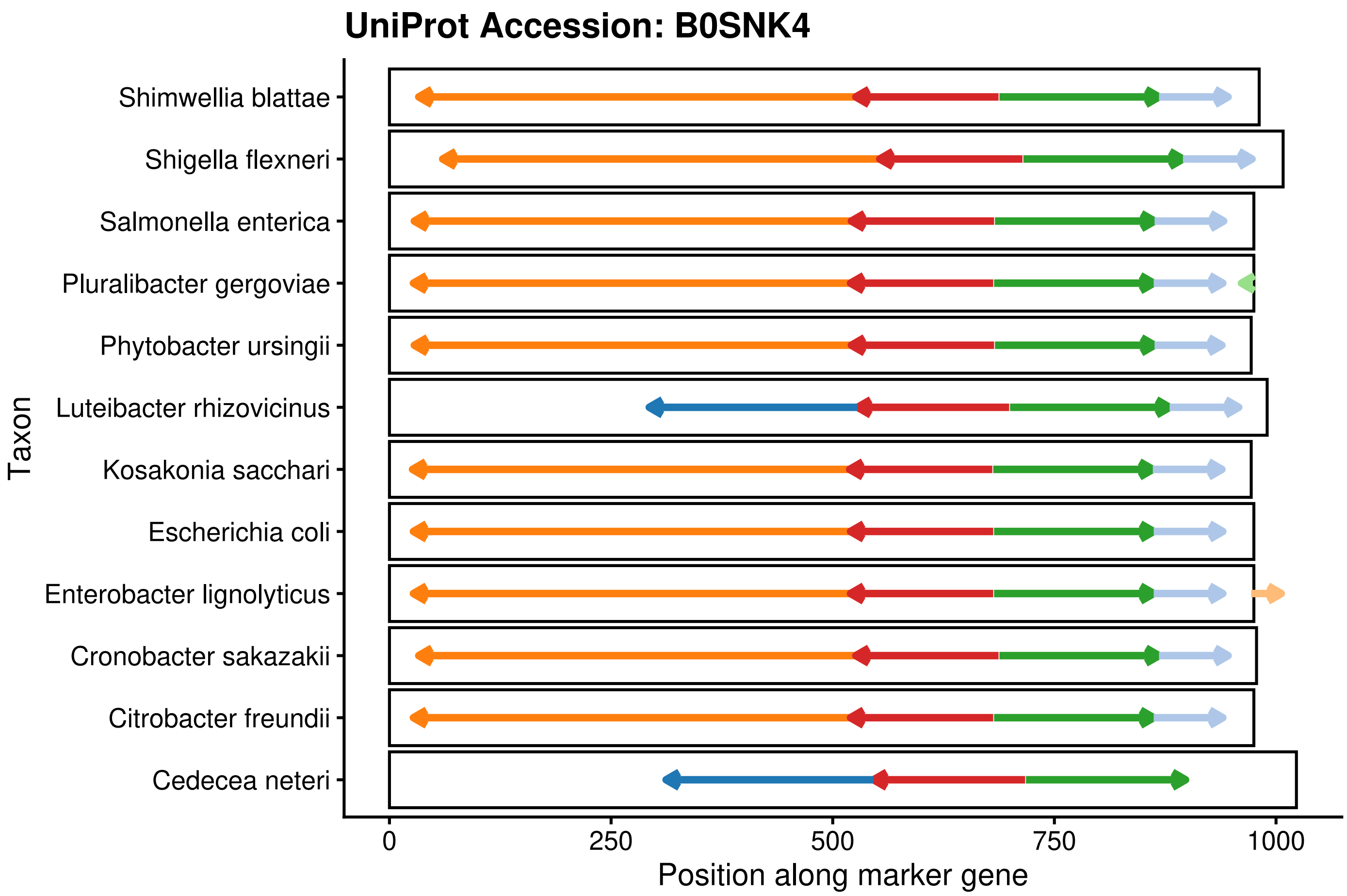

UniProt Accession: B0SNK4
Shimwellia blattae
Shigella flexneri
Salmonella enterica
Pluralibacter gergoviae
Phytobacter ursingii
Luteibacter rhizovicinus
Kosakonia sacchari
Escherichia coli
Enterobacter lignolyticus
Cronobacter sakazakii
Citrobacter freundii
Cedecea neteri
Taxon
0
250
500
750
1000
Position along marker gene

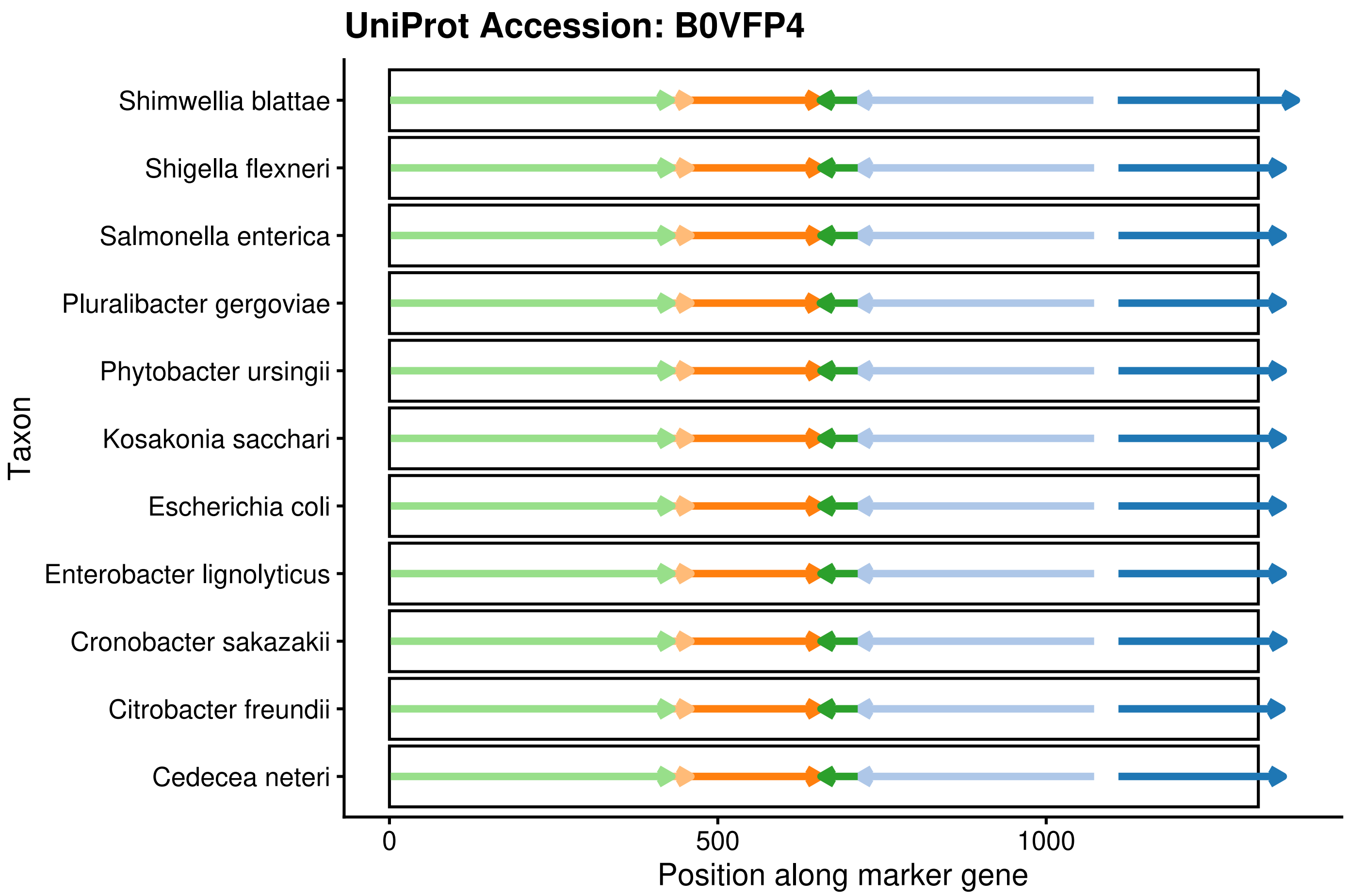
UniProt Accession: B0VFP4
Shimwellia blattae
Shigella flexneri
Salmonella enterica
Pluralibacter gergoviae
Phytobacter ursingii
Kosakonia sacchari
Escherichia coli
Enterobacter lignolyticus
Cronobacter sakazakii
Citrobacter freundii
Cedecea neteri
Taxon
0
500
1000
Position along marker gene

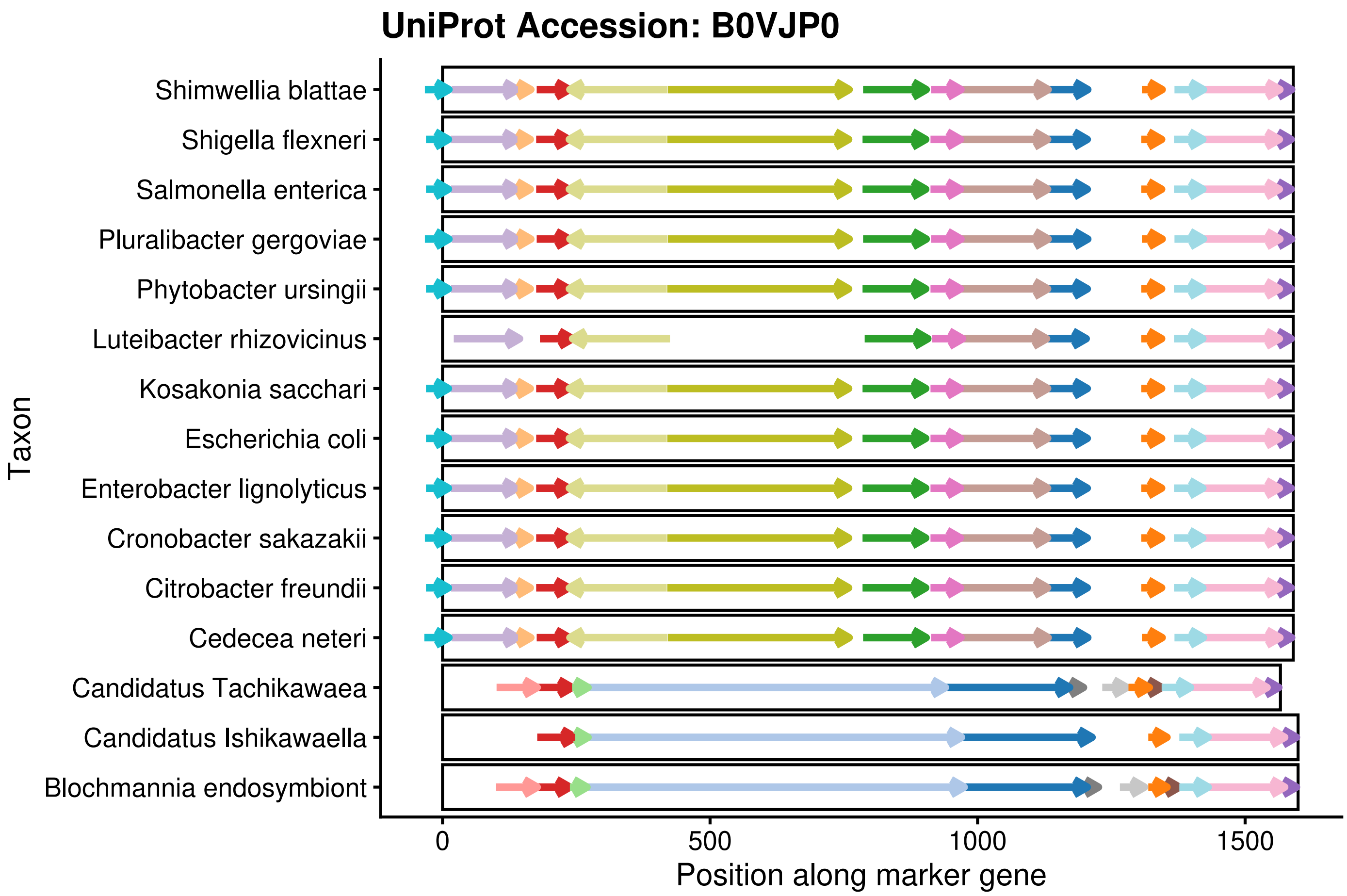
UniProt Accession: B0VJP0
Shimwellia blattae
Shigella flexneri
Salmonella enterica
Pluralibacter gergoviae
Phytobacter ursingii
Luteibacter rhizovicinus
Kosakonia sacchari
Escherichia coli
Enterobacter lignolyticus
Cronobacter sakazakii
Citrobacter freundii
Cedecea neteri
Candidatus Tachikawaea
Candidatus Ishikawaella
Blochmannia endosymbiont
Taxon
0
500
1000
1500
Position along marker gene

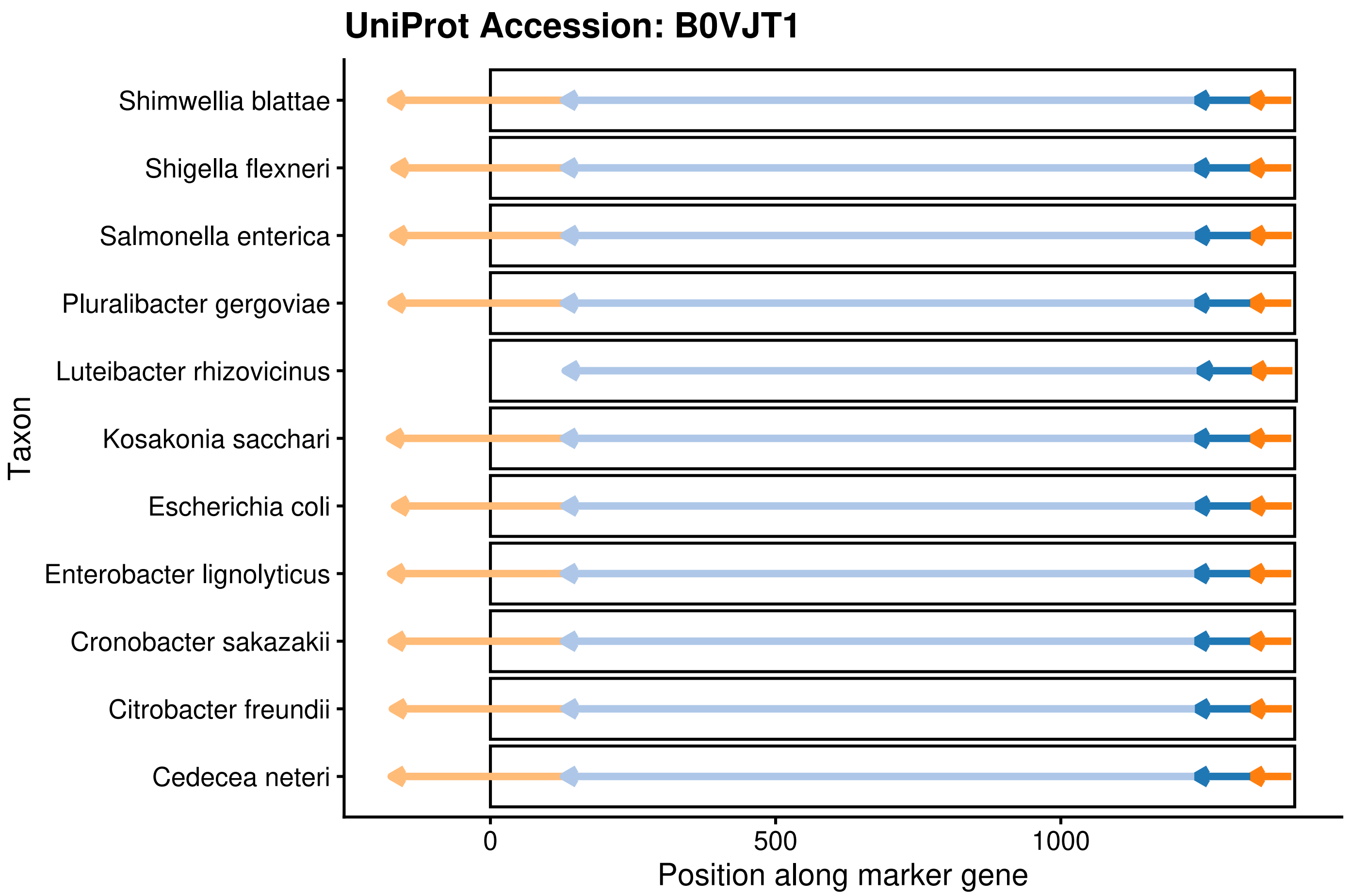

UniProt Accession: B0VJT1
Shimwellia blattae
Shigella flexneri
Salmonella enterica
Pluralibacter gergoviae
Luteibacter rhizovicinus
Kosakonia sacchari
Escherichia coli
Enterobacter lignolyticus
Cronobacter sakazakii
Citrobacter freundii
Cedecea neteri
Taxon
0
500
1000
Position along marker gene

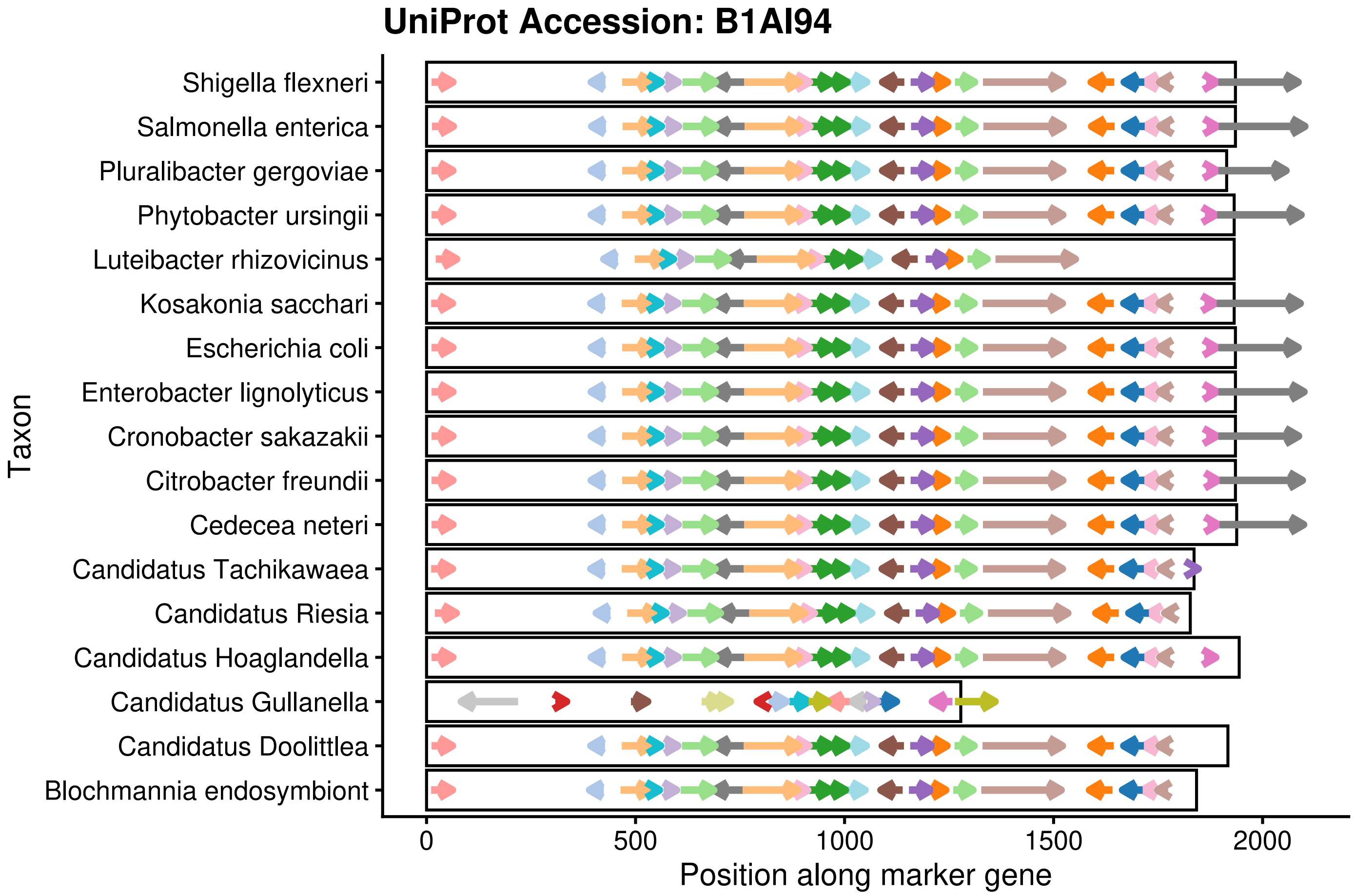
UniProt Accession: B1AI94
Shigella flexneri
Salmonella enterica
Pluralibacter gergoviae
Phytobacter ursingii
Luteibacter rhizovicinus
Kosakonia sacchari
Escherichia coli
Enterobacter lignolyticus
Cronobacter sakazakii
Citrobacter freundii
Cedecea neteri
Candidatus Tachikawaea
Candidatus Riesia
Candidatus Hoaglandella
Candidatus Gullanella
Candidatus Doolittlea
Blochmannia endosymbiont
Taxon
0
500
1000
1500
2000
Position along marker gene

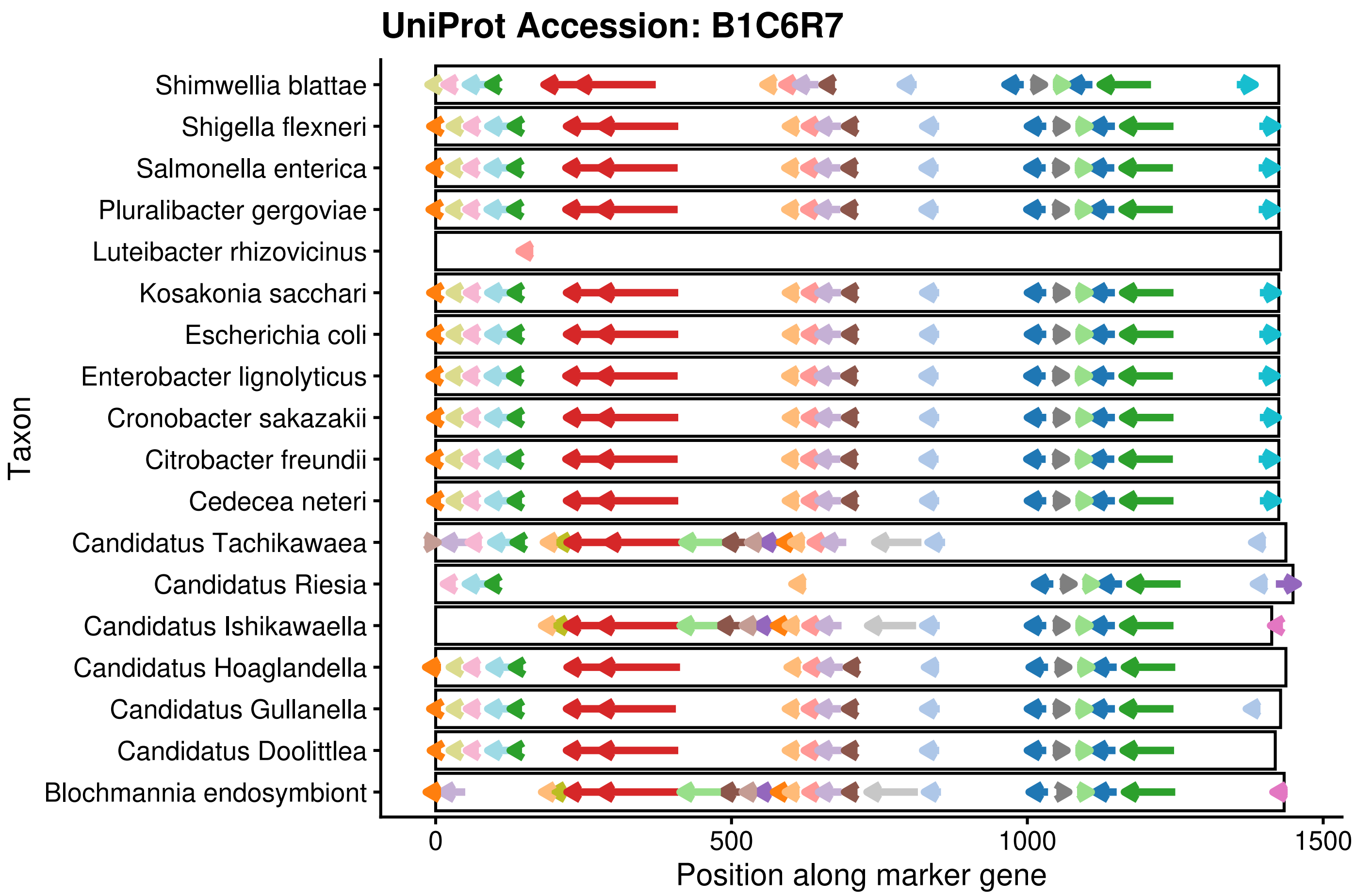

UniProt Accession: B1C6R7
Shimwellia blattae
Shigella flexneri
Salmonella enterica
Pluralibacter gergoviae
Luteibacter rhizovicinus
Kosakonia sacchari
Escherichia coli
Enterobacter lignolyticus
Cronobacter sakazakii
Citrobacter freundii
Cedecea neteri
Candidatus Tachikawaea
Candidatus Riesia
Candidatus Ishikawaella
Candidatus Hoaglandella
Candidatus Gullanella
Candidatus Doolittlea
Blochmannia endosymbiont
Taxon
0
500
1000
1500
Position along marker gene

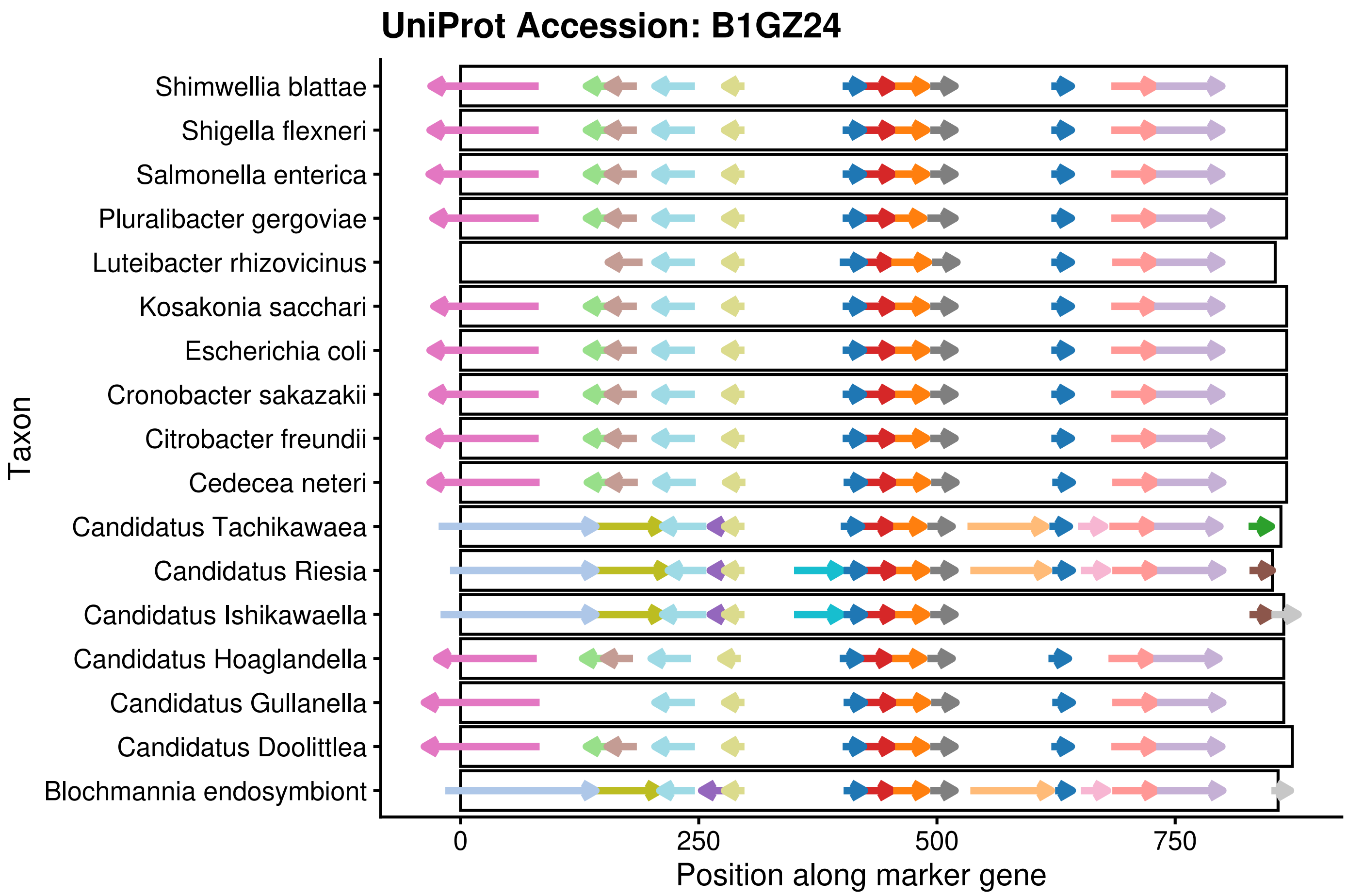
UniProt Accession: B1GZ24
Taxon
Shimwellia blattae
Shigella flexneri
Salmonella enterica
Pluralibacter gergoviae
Luteibacter rhizovicinus
Kosakonia sacchari
Escherichia coli
Cronobacter sakazakii
Citrobacter freundii
Cedecea neteri
Candidatus Tachikawaea
Candidatus Riesia
Candidatus Ishikawaella
Candidatus Hoaglandella
Candidatus Gullanella
Candidatus Doolittlea
Blochmannia endosymbiont
0
250
500
750
Position along marker gene

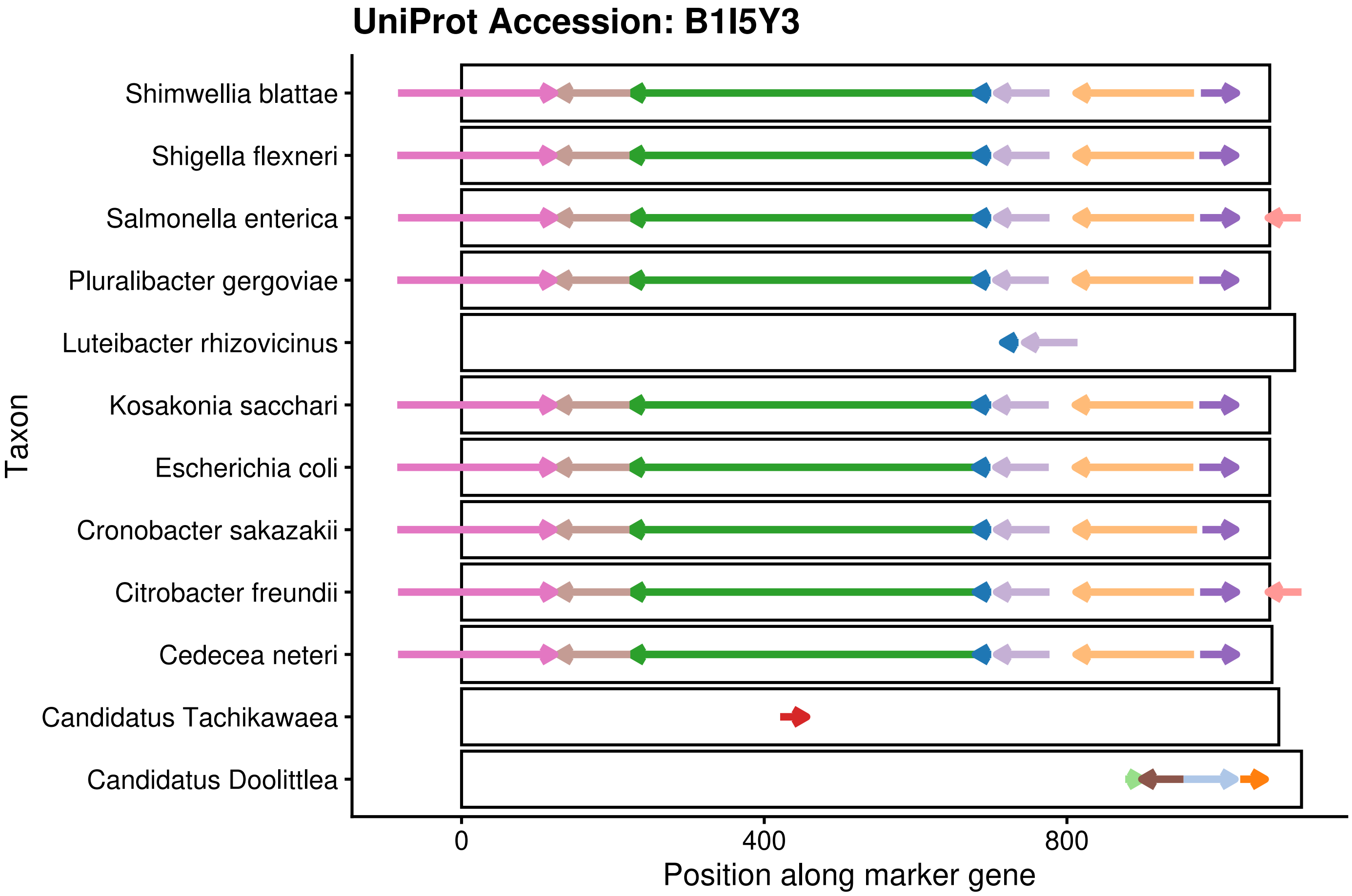

UniProt Accession: B1I5Y3
Shimwellia blattae
Shigella flexneri
Salmonella enterica
Pluralibacter gergoviae
Luteibacter rhizovicinus
Kosakonia sacchari
Escherichia coli
Cronobacter sakazakii
Citrobacter freundii
Cedecea neteri
Candidatus Tachikawaea
Candidatus Doolittlea
Taxon
0
400
800
Position along marker gene

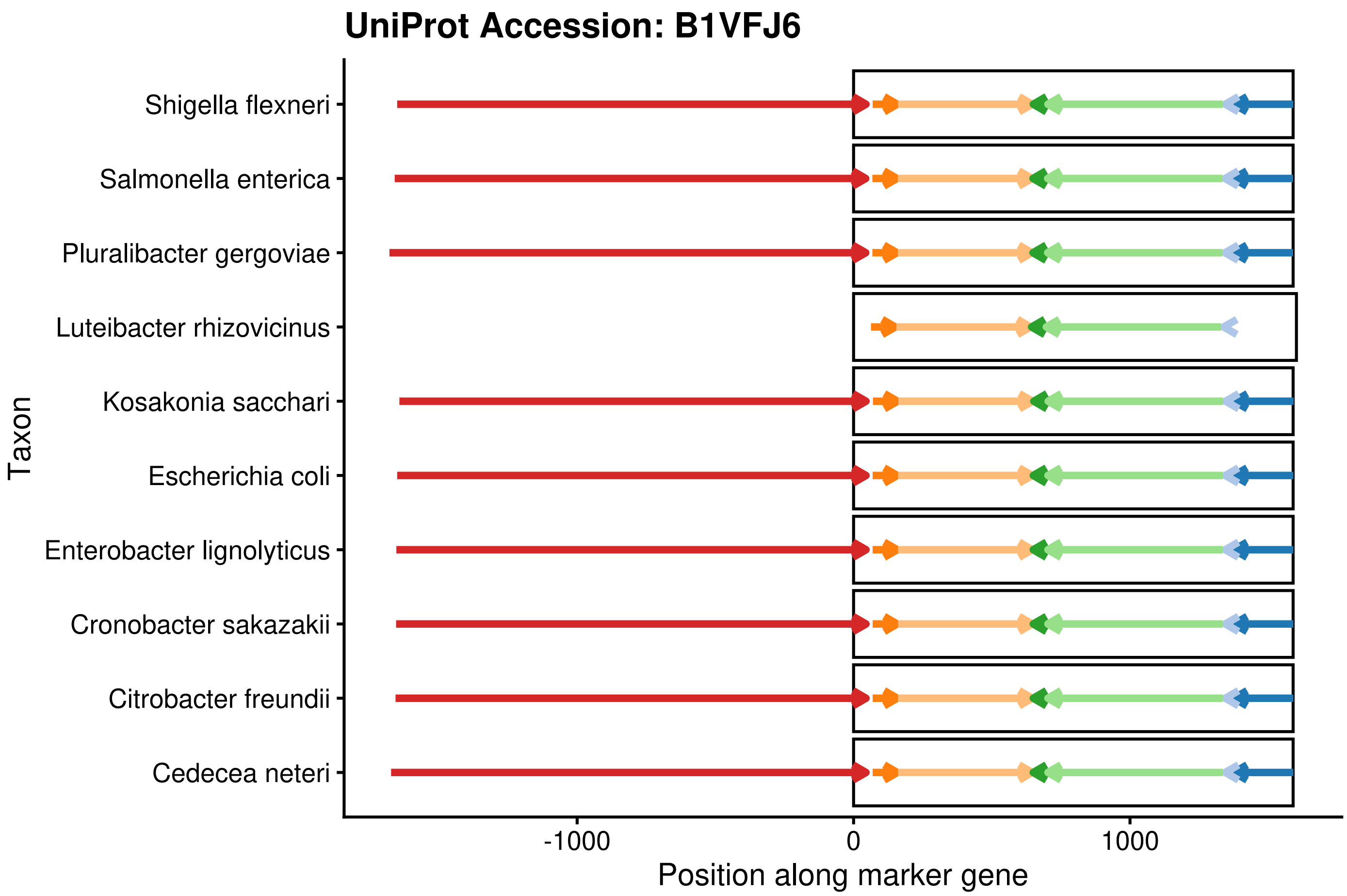
UniProt Accession: B1VFJ6
Shigella flexneri
Salmonella enterica
Pluralibacter gergoviae
Luteibacter rhizovicinus
Kosakonia sacchari
Escherichia coli
Enterobacter lignolyticus
Cronobacter sakazakii
Citrobacter freundii
Cedecea neteri
Taxon
-1000
0
1000
Position along marker gene

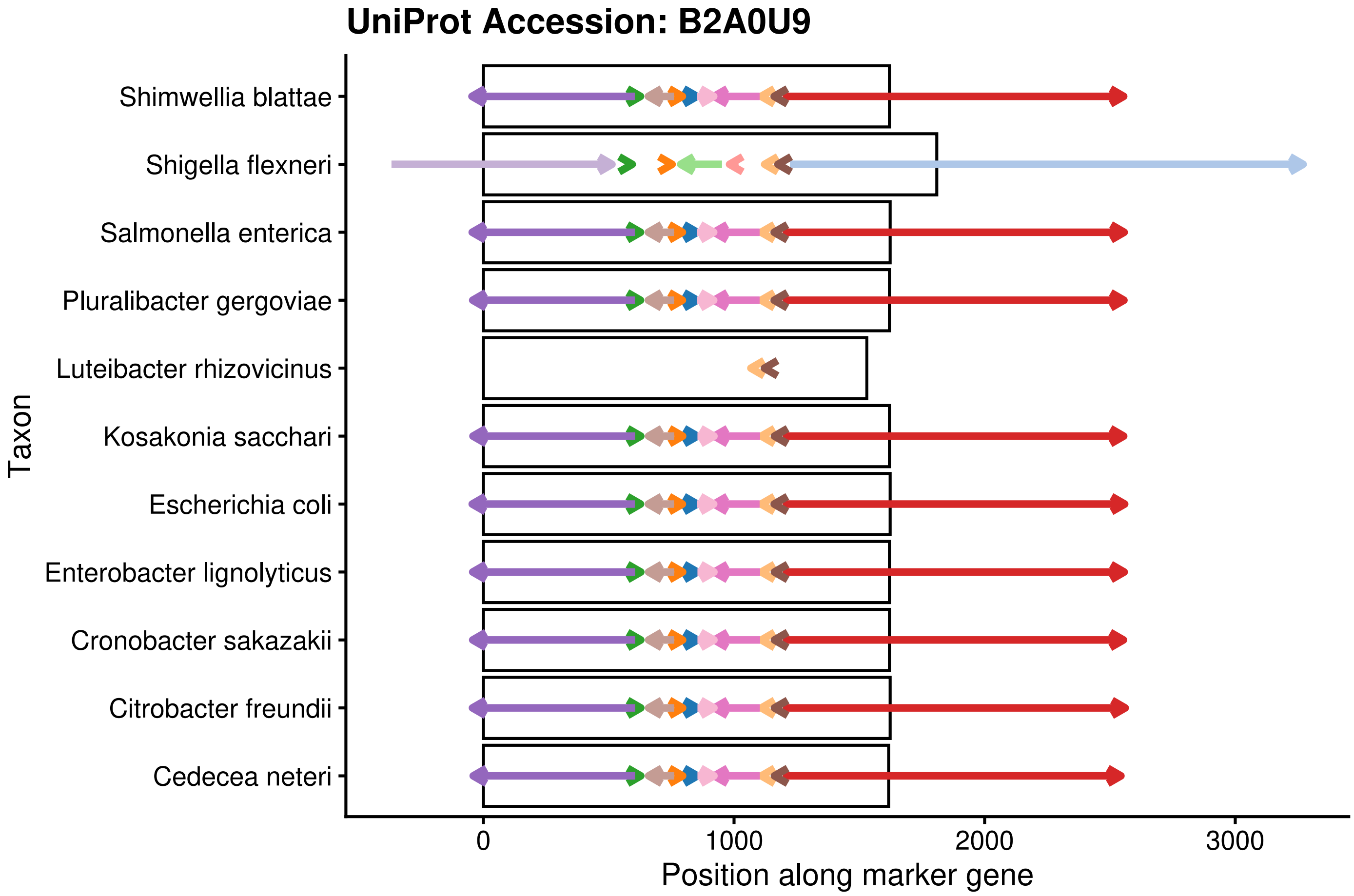
UniProt Accession: B2A0U9
Shimwellia blattae
Shigella flexneri
Salmonella enterica
Pluralibacter gergoviae
Luteibacter rhizovicinus
Kosakonia sacchari
Escherichia coli
Enterobacter lignolyticus
Cronobacter sakazakii
Citrobacter freundii
Cedecea neteri
Taxon
0
1000
2000
3000
Position along marker gene

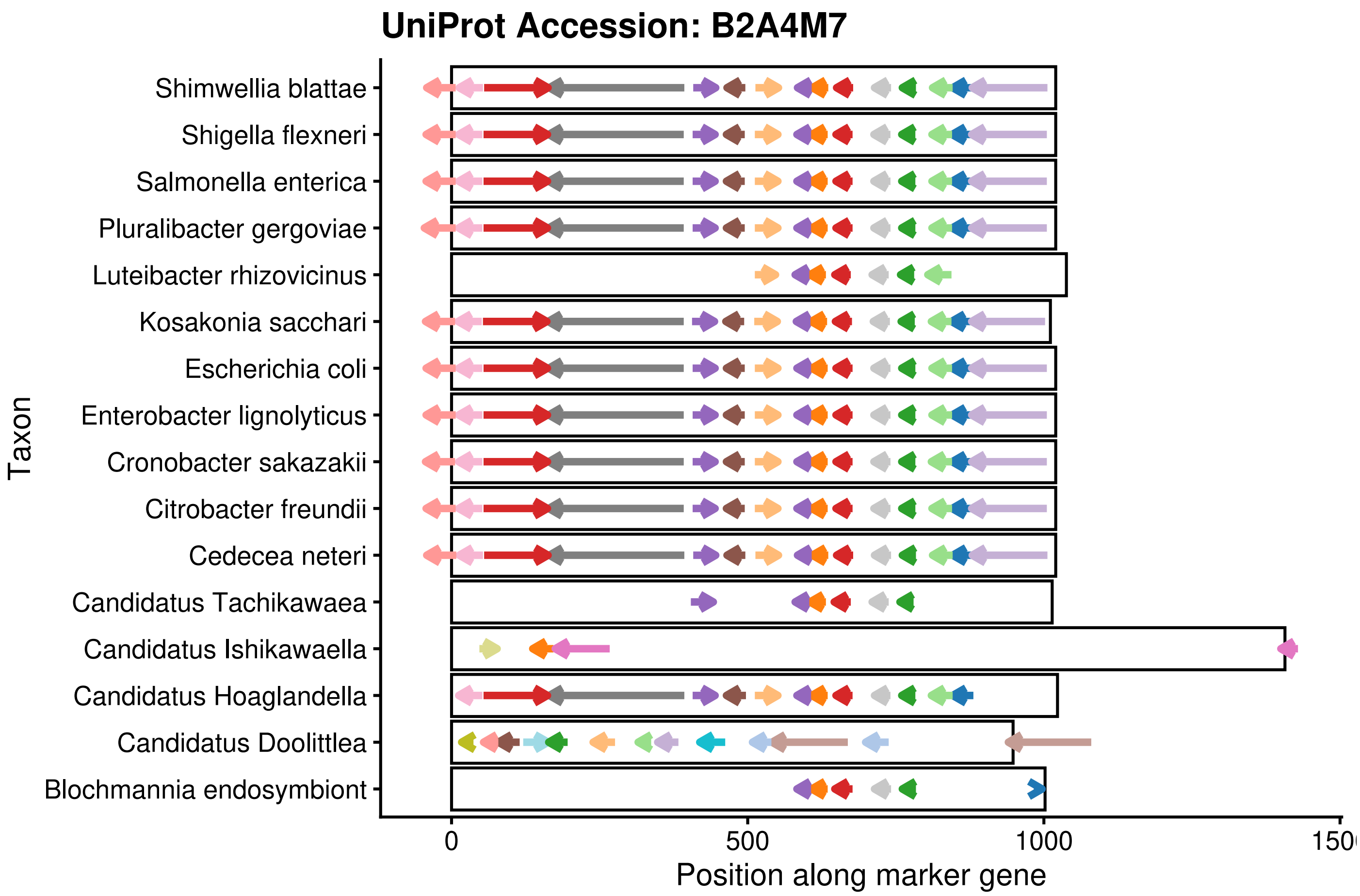

UniProt Accession: B2A4M7
Shimwellia blattae
Shigella flexneri
Salmonella enterica
Pluralibacter gergoviae
Luteibacter rhizovicinus
Kosakonia sacchari
Escherichia coli
Enterobacter lignolyticus
Cronobacter sakazakii
Citrobacter freundii
Cedecea neteri
Candidatus Tachikawaea
Candidatus Ishikawaella
Candidatus Hoaglandella
Candidatus Doolittlea
Blochmannia endosymbiont
Taxon
0
500
1000
150
Position along marker gene

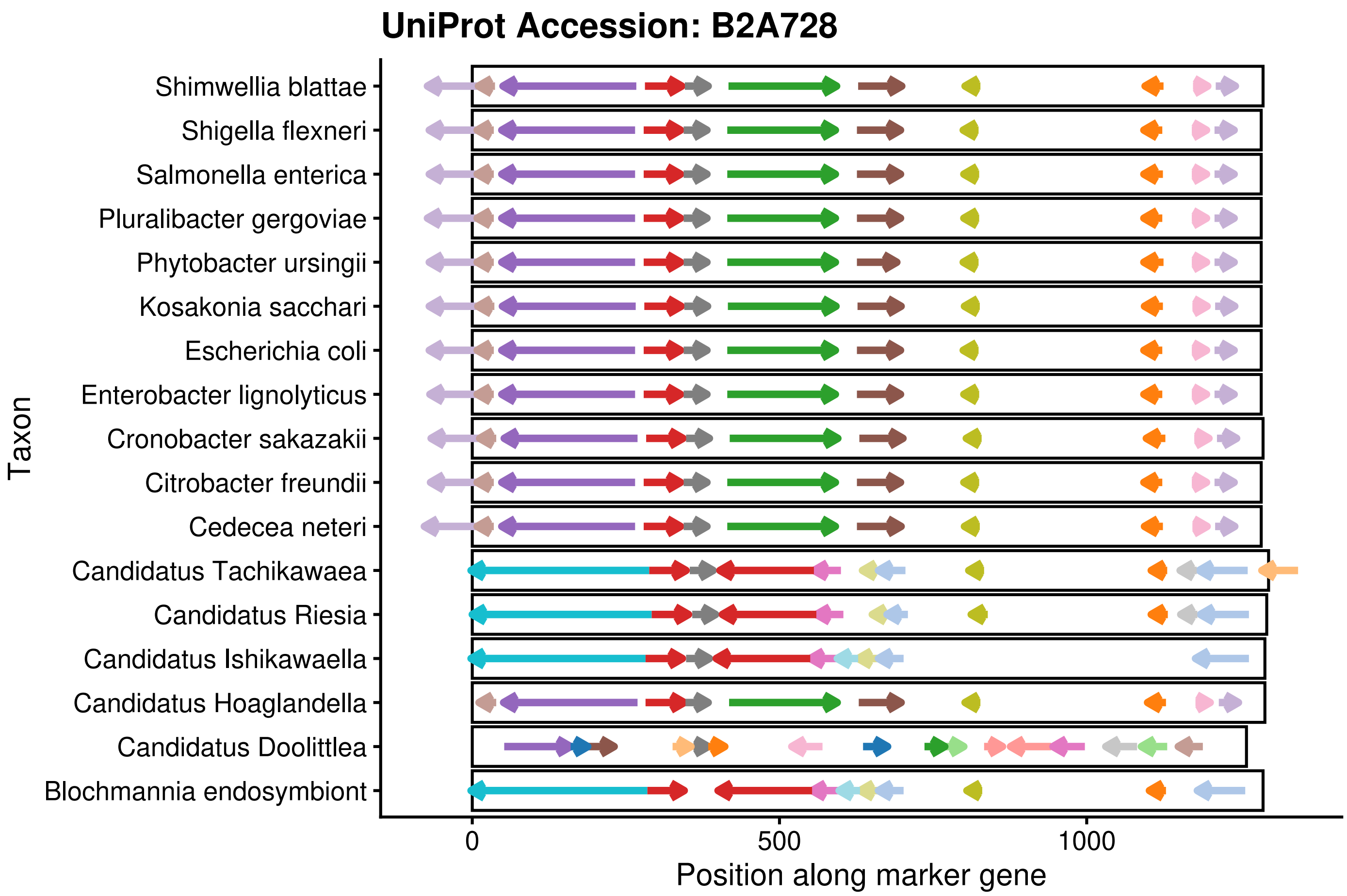

UniProt Accession: B2A728
Taxon
Shimwellia blattae
Shigella flexneri
Salmonella enterica
Pluralibacter gergoviae
Phytobacter ursingii
Kosakonia sacchari
Escherichia coli
Enterobacter lignolyticus
Cronobacter sakazakii
Citrobacter freundii
Cedecea neteri
Candidatus Tachikawaea
Candidatus Riesia
Candidatus Ishikawaella
Candidatus Hoaglandella
Candidatus Doolittlea
Blochmannia endosymbiont
0
500
1000
Position along marker gene

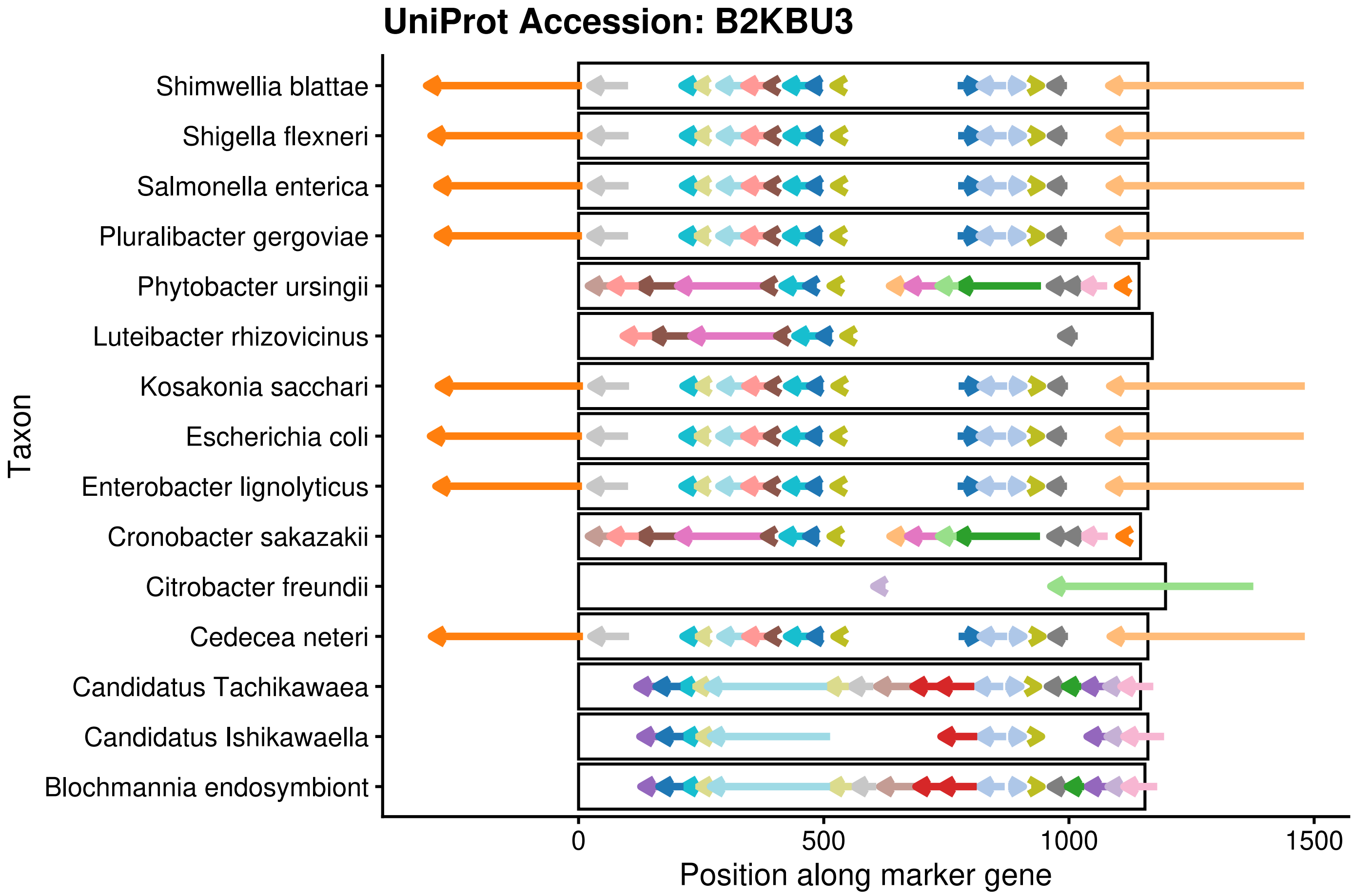
UniProt Accession: B2KBU3
Taxon
Shimwellia blattae
Shigella flexneri
Salmonella enterica
Pluralibacter gergoviae
Phytobacter ursingii
Luteibacter rhizovicinus
Kosakonia sacchari
Escherichia coli
Enterobacter lignolyticus
Cronobacter sakazakii
Citrobacter freundii
Cedecea neteri
Candidatus Tachikawaea
Candidatus Ishikawaella
Blochmannia endosymbiont
0
500
1000
1500
Position along marker gene

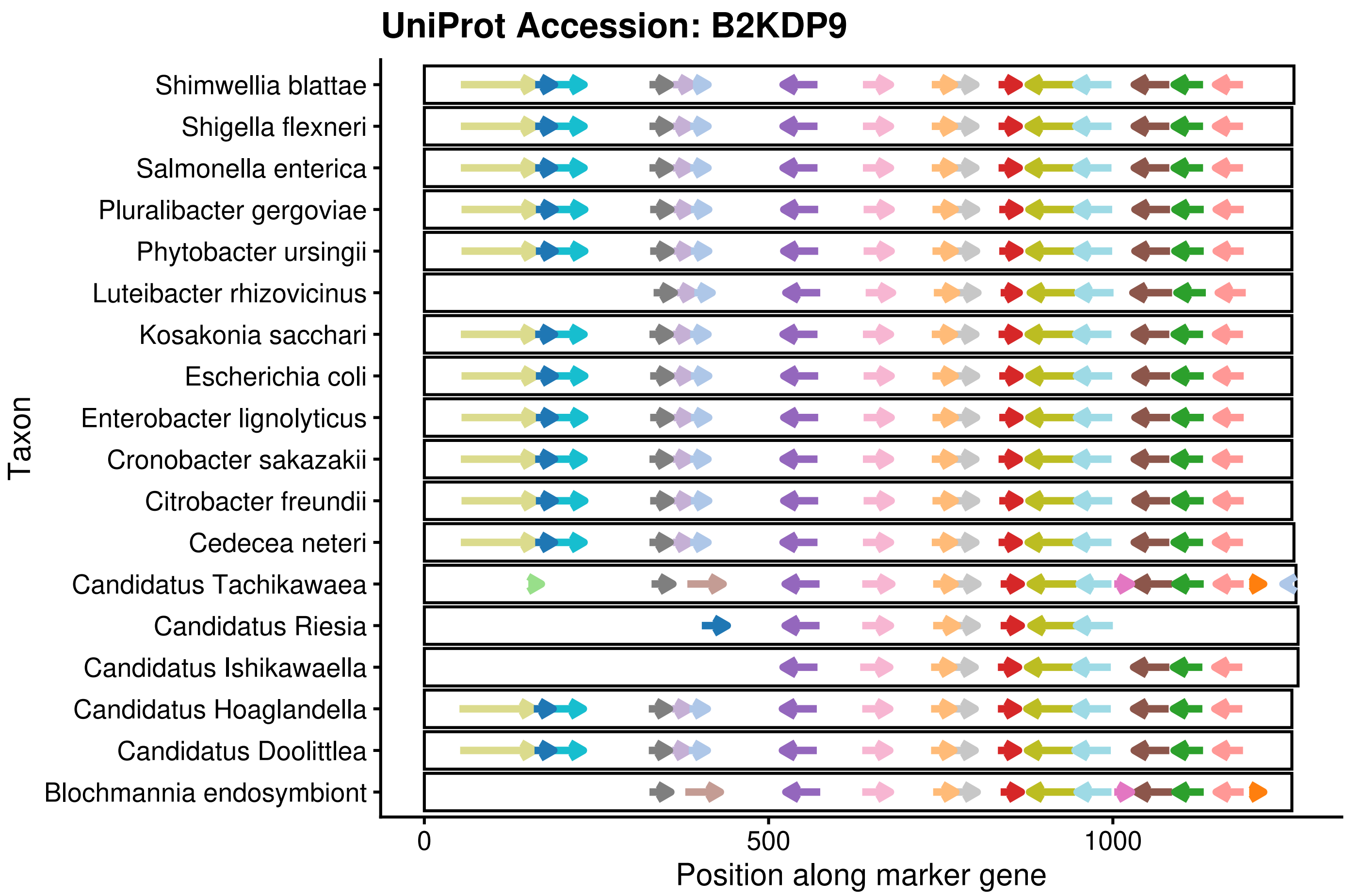
UniProt Accession: B2KDP9
Taxon
Shimwellia blattae
Shigella flexneri
Salmonella enterica
Pluralibacter gergoviae
Phytobacter ursingii
Luteibacter rhizovicinus
Kosakonia sacchari
Escherichia coli
Enterobacter lignolyticus
Cronobacter sakazakii
Citrobacter freundii
Cedecea neteri
Candidatus Tachikawaea
Candidatus Riesia
Candidatus Ishikawaella
Candidatus Hoaglandella
Candidatus Doolittlea
Blochmannia endosymbiont
0
500
1000
Position along marker gene

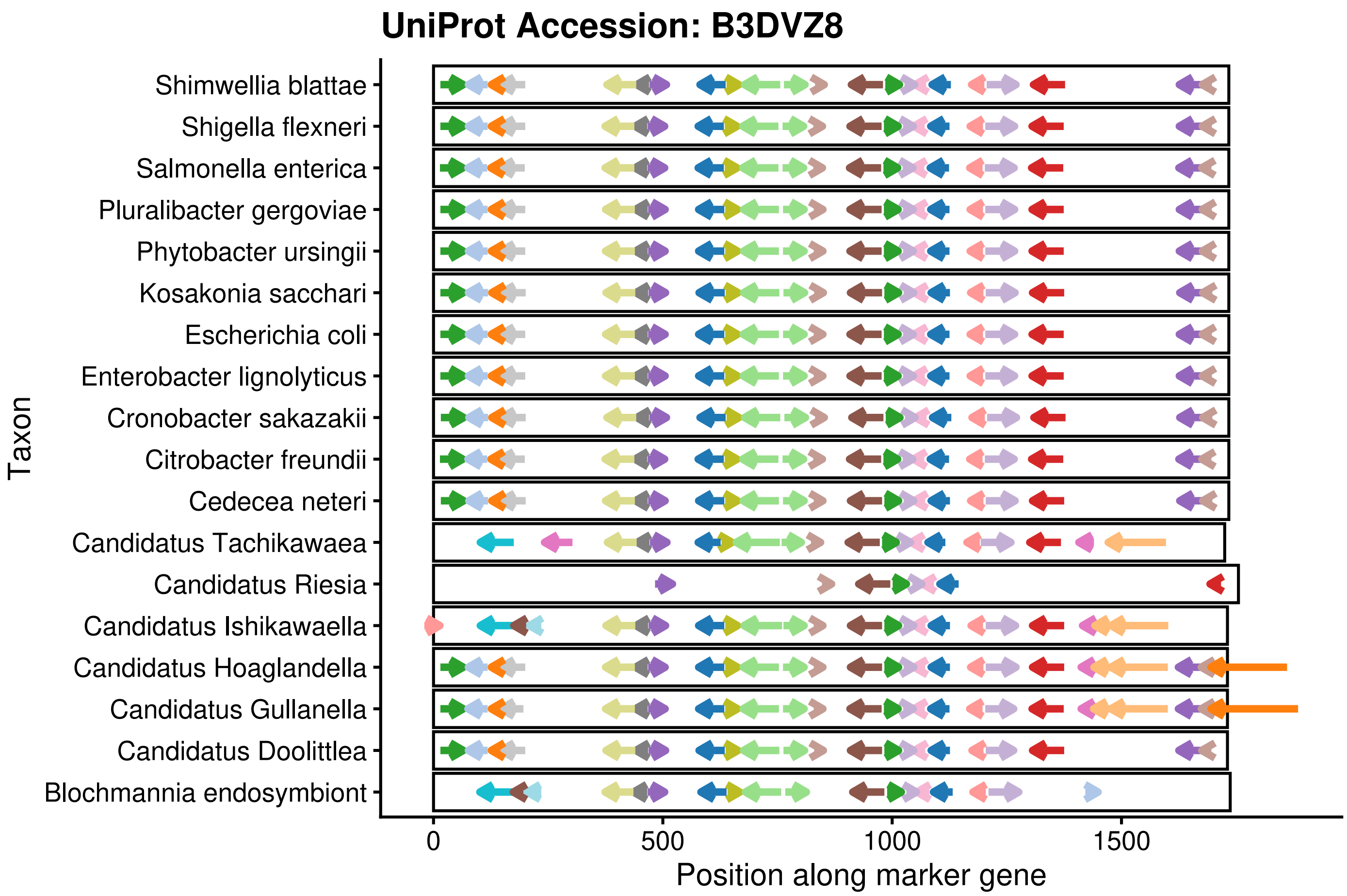
UniProt Accession: B3DVZ8
Shimwellia blattae
Shigella flexneri
Salmonella enterica
Pluralibacter gergoviae
Phytobacter ursingii
Kosakonia sacchari
Escherichia coli
Enterobacter lignolyticus
Cronobacter sakazakii
Citrobacter freundii
Cedecea neteri
Candidatus Tachikawaea
Candidatus Riesia
Candidatus Ishikawaella
Candidatus Hoaglandella
Candidatus Gullanella
Candidatus Doolittlea
Blochmannia endosymbiont
Taxon
0
500
1000
1500
Position along marker gene

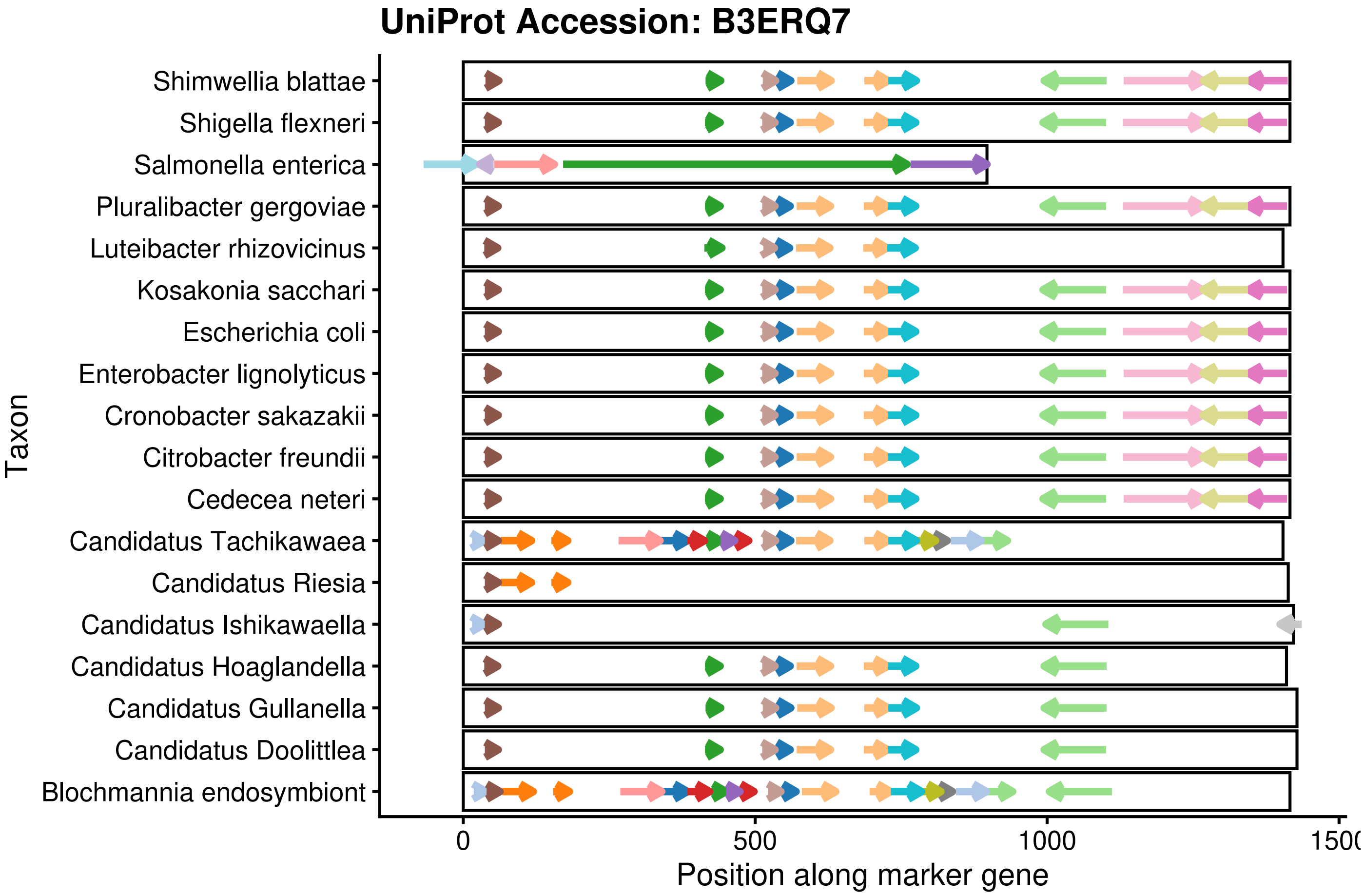
UniProt Accession: B3ERQ7
Taxon
Shimwellia blattae
Shigella flexneri
Salmonella enterica
Pluralibacter gergoviae
Luteibacter rhizovicinus
Kosakonia sacchari
Escherichia coli
Enterobacter lignolyticus
Cronobacter sakazakii
Citrobacter freundii
Cedecea neteri
Candidatus Tachikawaea
Candidatus Riesia
Candidatus Ishikawaella
Candidatus Hoaglandella
Candidatus Gullanella
Candidatus Doolittlea
Blochmannia endosymbiont
0
500
1000
Position along marker gene

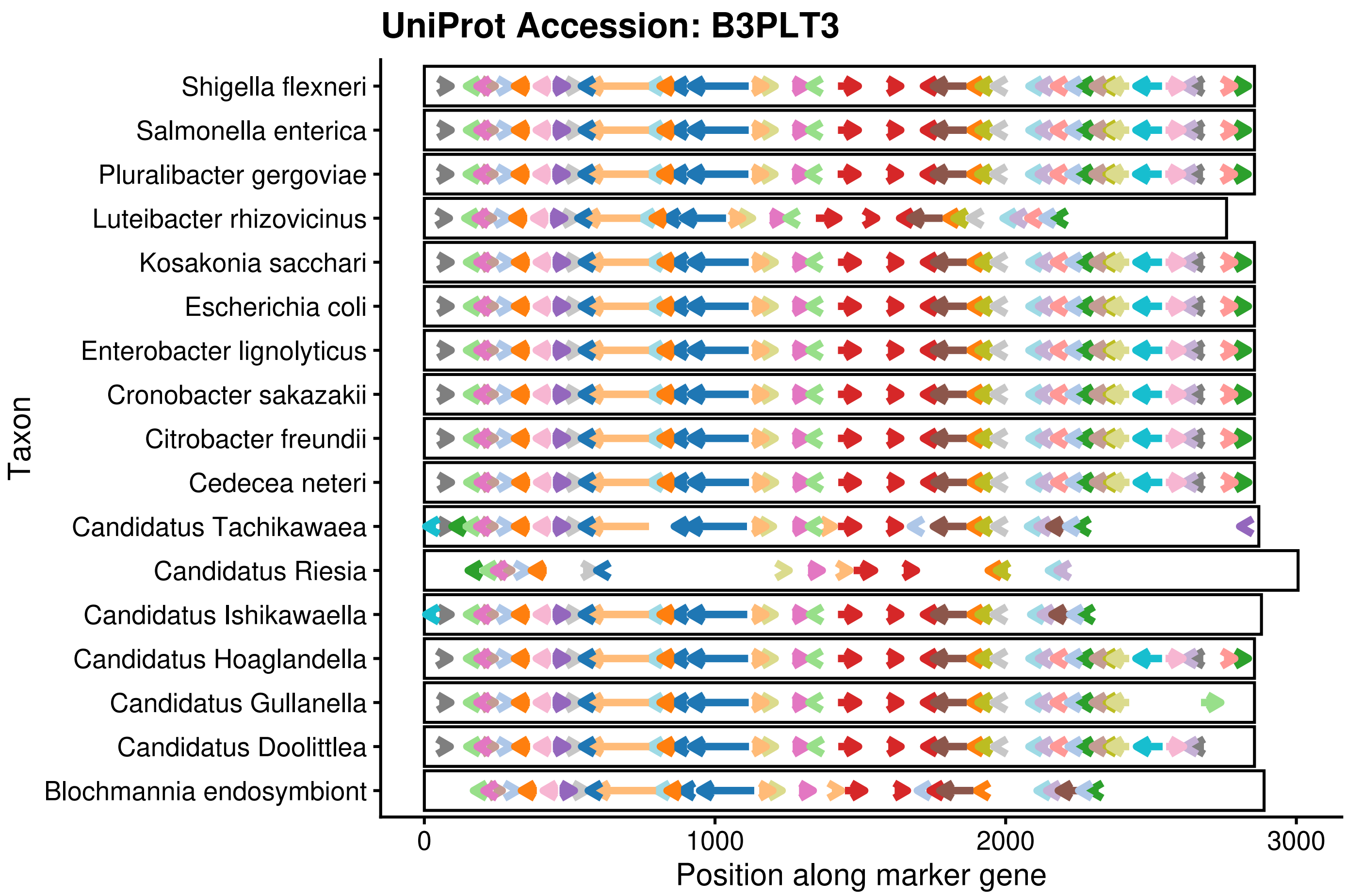

UniProt Accession: B3PLT3
Shigella flexneri
Salmonella enterica
Pluralibacter gergoviae
Luteibacter rhizovicinus
Kosakonia sacchari
Escherichia coli
Enterobacter lignolyticus
Cronobacter sakazakii
Citrobacter freundii
Cedecea neteri
Candidatus Tachikawaea
Candidatus Riesia
Candidatus Ishikawaella
Candidatus Hoaglandella
Candidatus Gullanella
Candidatus Doolittlea
Blochmannia endosymbiont
Taxon
0
1000
2000
3000
Position along marker gene

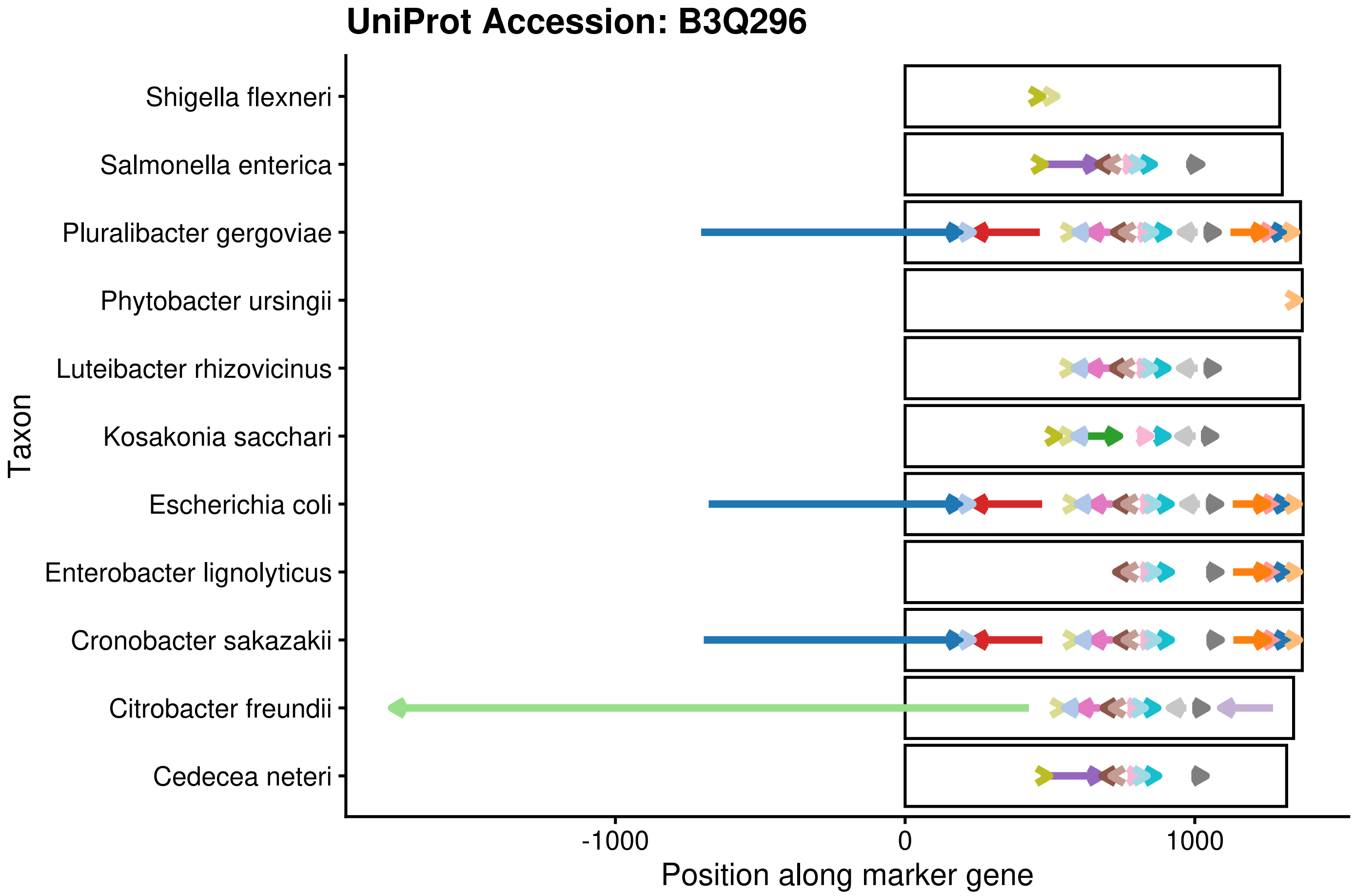
UniProt Accession: B3Q296
Shigella flexneri
Salmonella enterica
Pluralibacter gergoviae
Phytobacter ursingii
Luteibacter rhizovicinus
Kosakonia sacchari
Escherichia coli
Enterobacter lignolyticus
Cronobacter sakazakii
Citrobacter freundii
Cedecea neteri
Taxon
-1000
0
1000
Position along marker gene

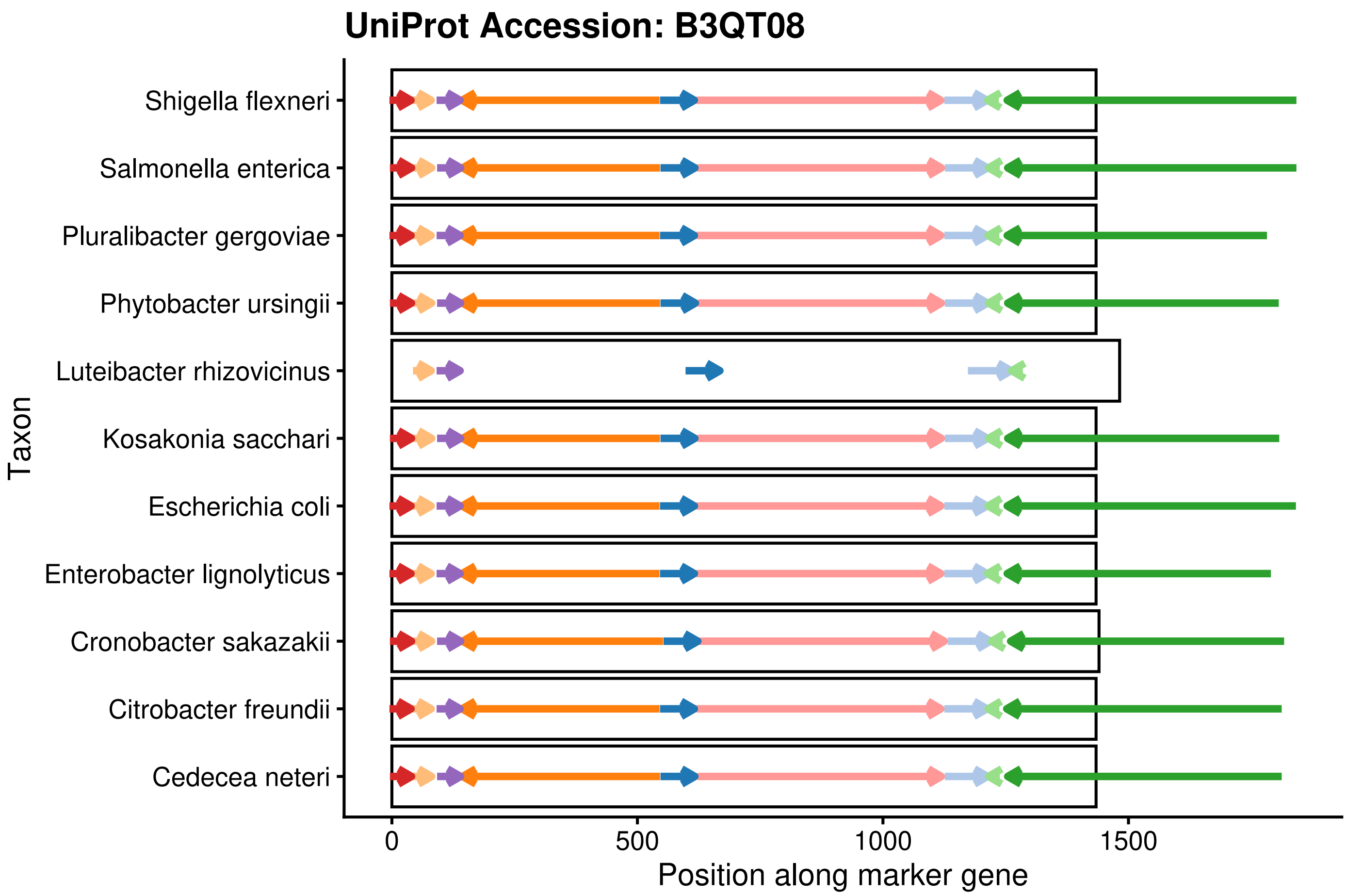
UniProt Accession: B3QT08
Taxon
Shigella flexneri
Salmonella enterica
Pluralibacter gergoviae
Phytobacter ursingii
Luteibacter rhizovicinus
Kosakonia sacchari
Escherichia coli
Enterobacter lignolyticus
Cronobacter sakazakii
Citrobacter freundii
Cedecea neteri
0
500
1000
1500
Position along marker gene

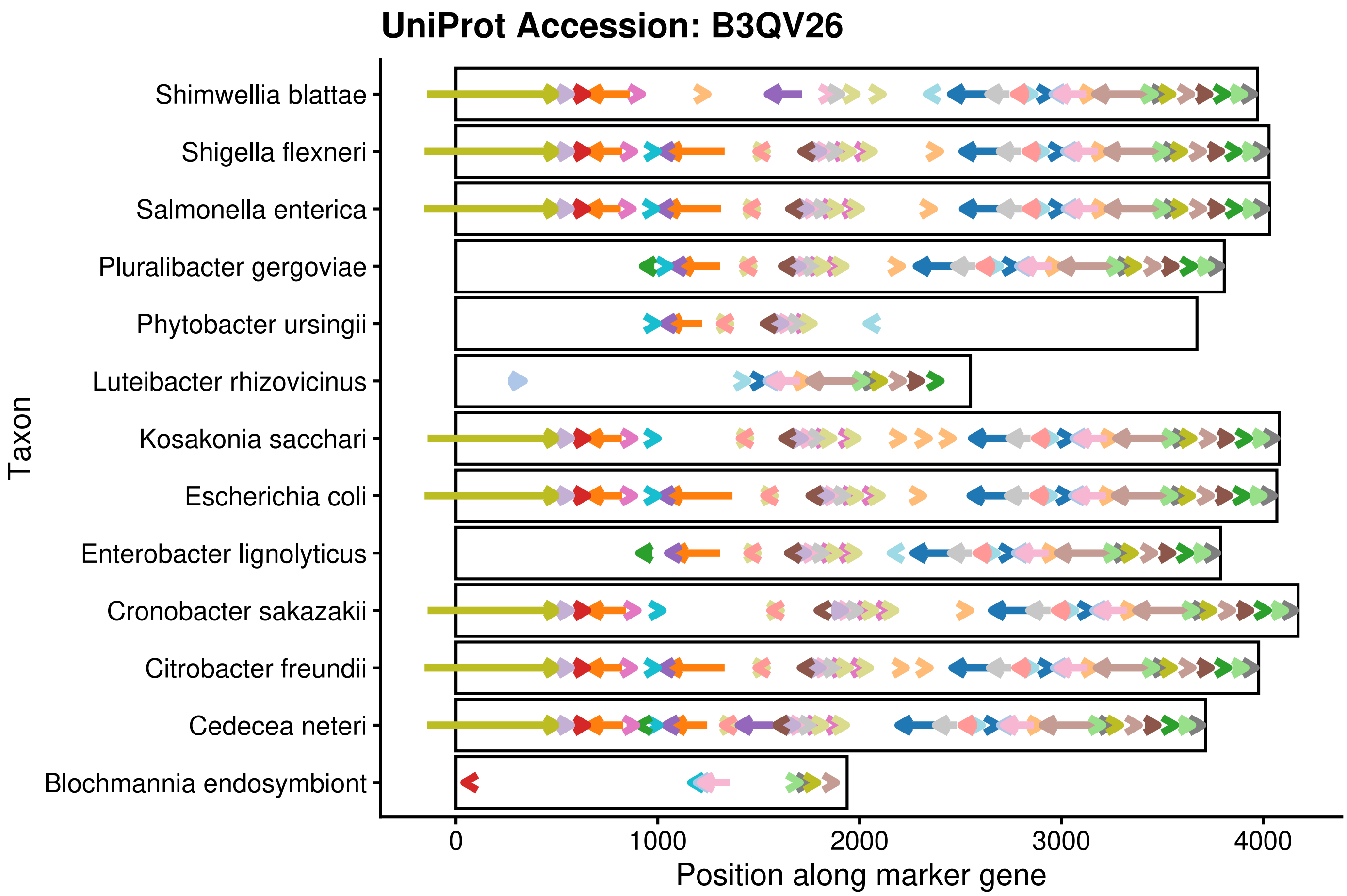

UniProt Accession: B3QV26
Shimwellia blattae
Shigella flexneri
Salmonella enterica
Pluralibacter gergoviae
Phytobacter ursingii
Luteibacter rhizovicinus
Kosakonia sacchari
Escherichia coli
Enterobacter lignolyticus
Cronobacter sakazakii
Citrobacter freundii
Cedecea neteri
Blochmannia endosymbiont
Taxon
0
1000
2000
3000
4000
Position along marker gene

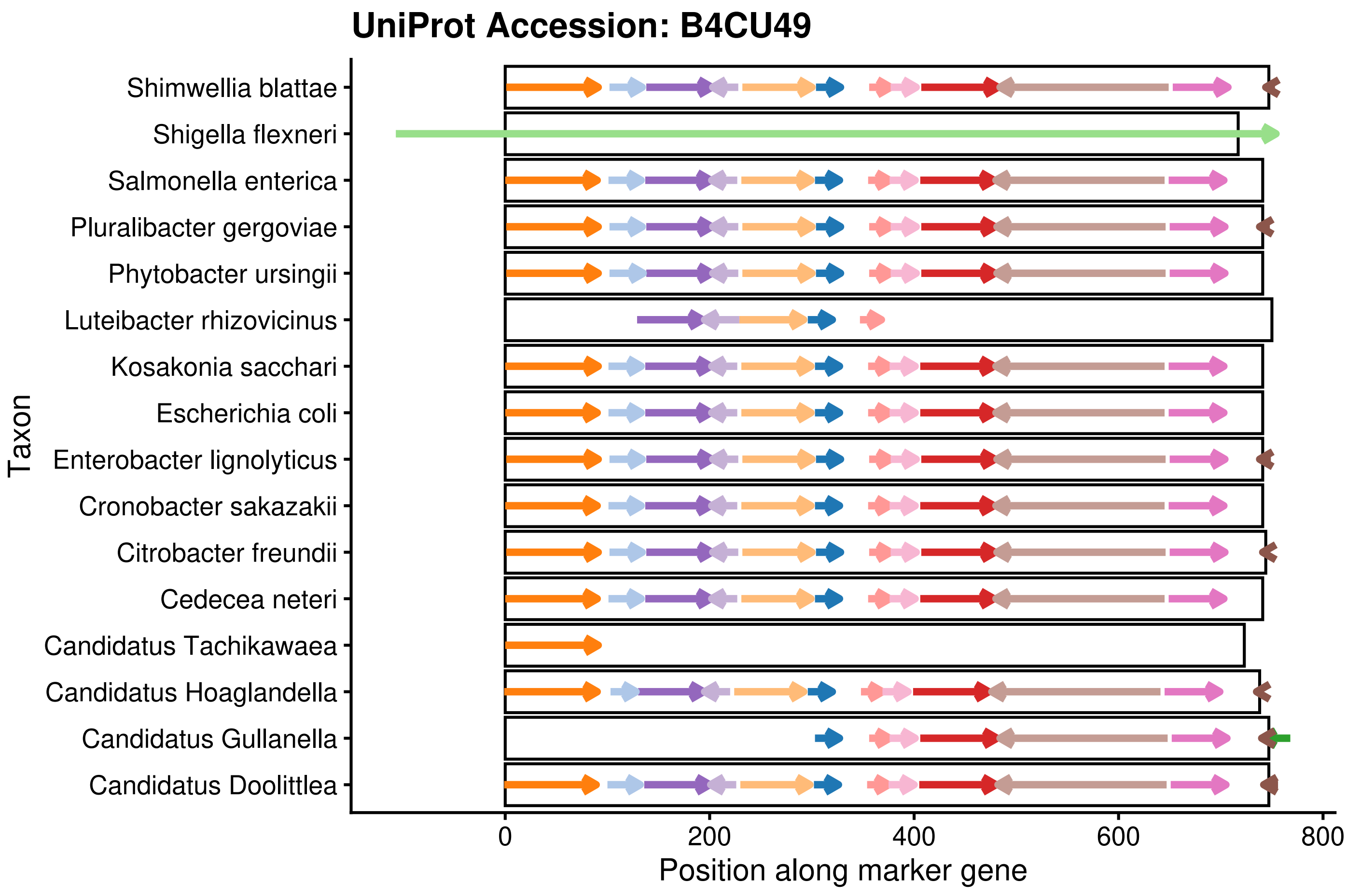
UniProt Accession: B4CU49
Shimwellia blattae
Shigella flexneri
Salmonella enterica
Pluralibacter gergoviae
Phytobacter ursingii
Luteibacter rhizovicinus
Kosakonia sacchari
Escherichia coli
Enterobacter lignolyticus
Cronobacter sakazakii
Citrobacter freundii
Cedecea neteri
Candidatus Tachikawaea
Candidatus Hoaglandella
Candidatus Gullanella
Candidatus Doolittlea
Taxon
0
200
400
600
800
Position along marker gene

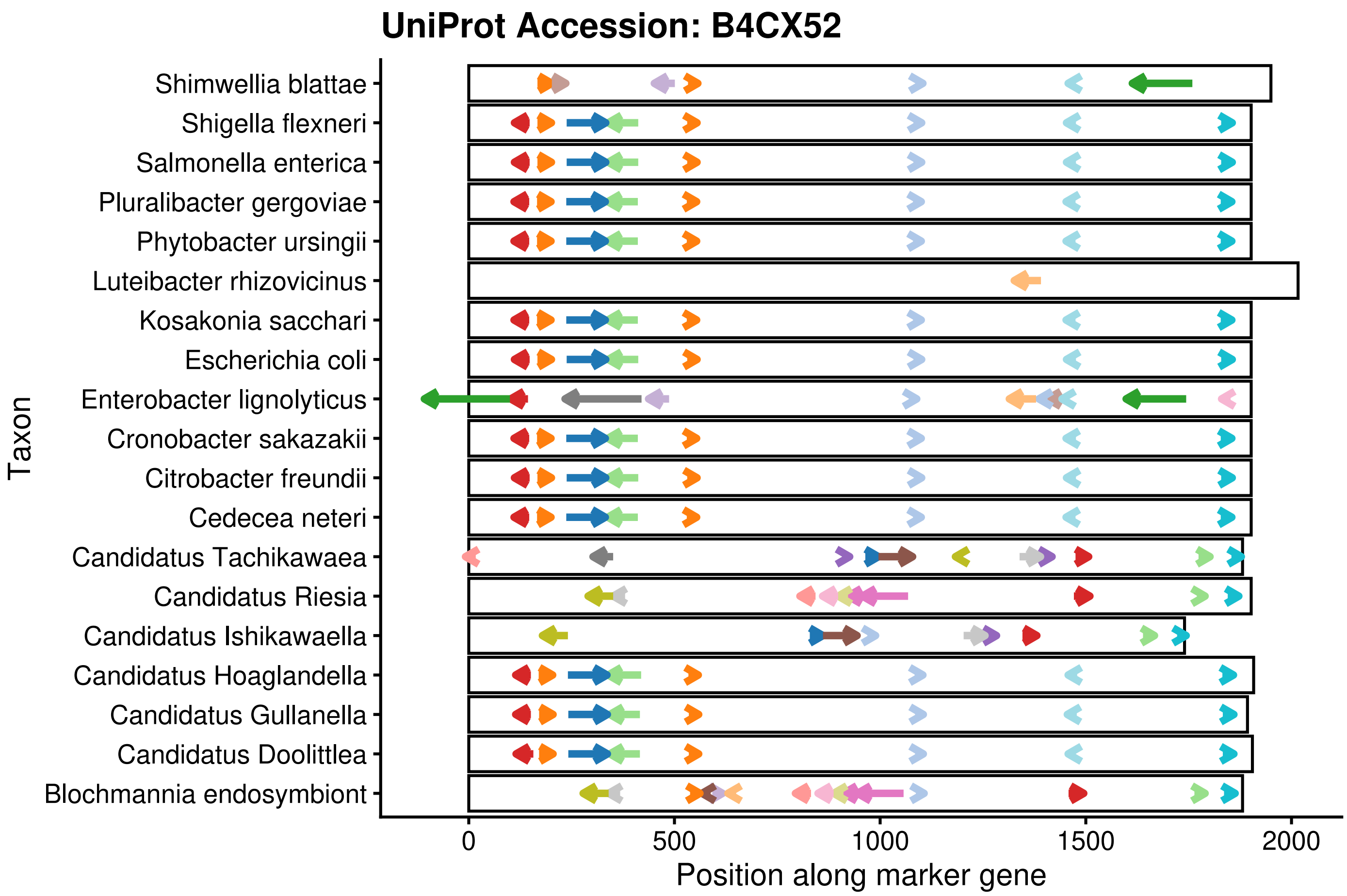

UniProt Accession: B4CX52
Shimwellia blattae
Shigella flexneri
Salmonella enterica
Pluralibacter gergoviae
Phytobacter ursingii
Luteibacter rhizovicinus
Kosakonia sacchari
Escherichia coli
Enterobacter lignolyticus
Cronobacter sakazakii
Citrobacter freundii
Cedecea neteri
Candidatus Tachikawaea
Candidatus Riesia
Candidatus Ishikawaella
Candidatus Hoaglandella
Candidatus Gullanella
Candidatus Doolittlea
Blochmannia endosymbiont
Taxon
0
500
1000
1500
2000
Position along marker gene

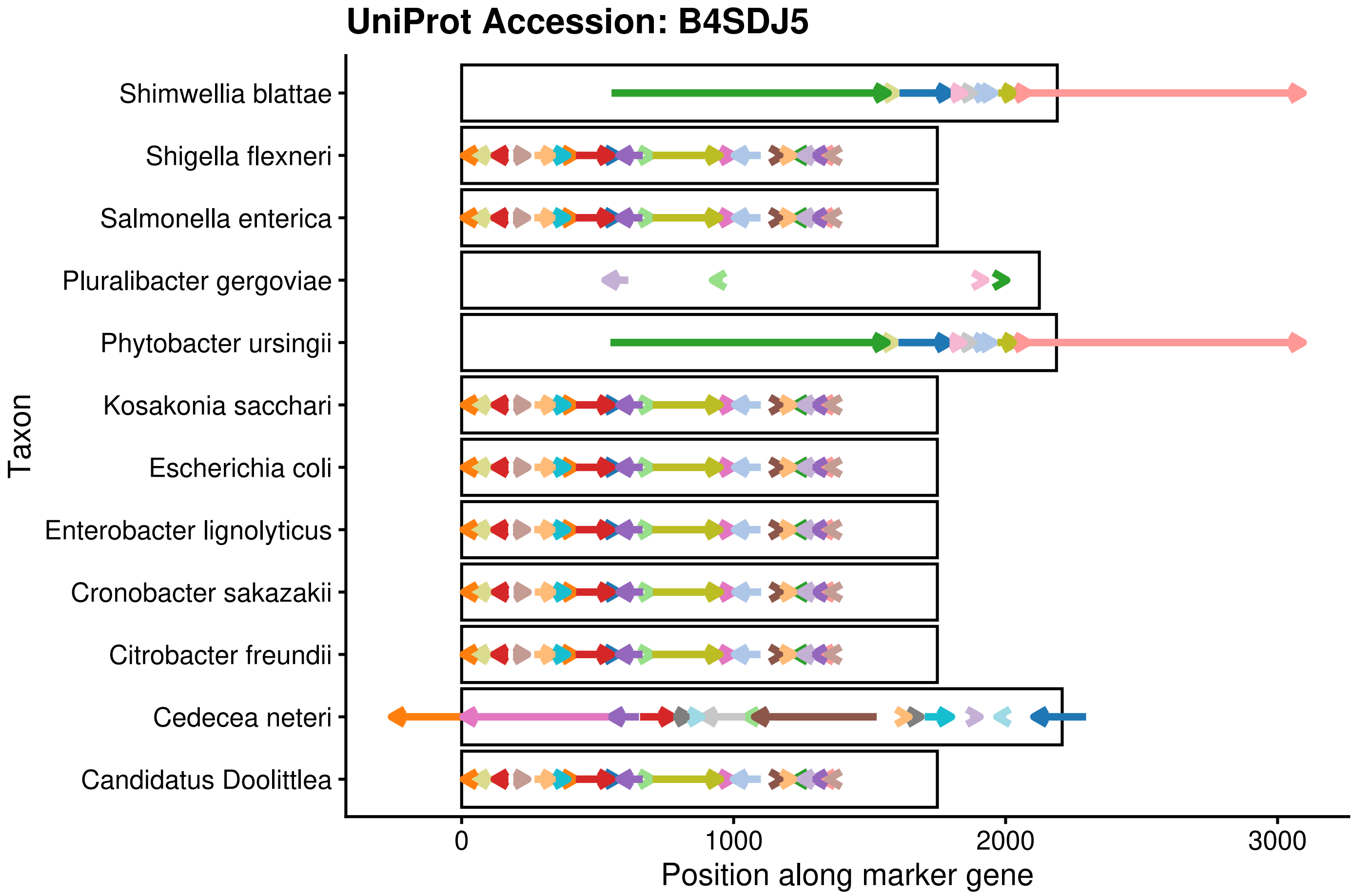

UniProt Accession: B4SDJ5
Taxon
Shimwellia blattae
Shigella flexneri
Salmonella enterica
Pluralibacter gergoviae
Phytobacter ursingii
Kosakonia sacchari
Escherichia coli
Enterobacter lignolyticus
Cronobacter sakazakii
Citrobacter freundii
Cedecea neteri
Candidatus Doolittlea
0
1000
2000
3000
Position along marker gene

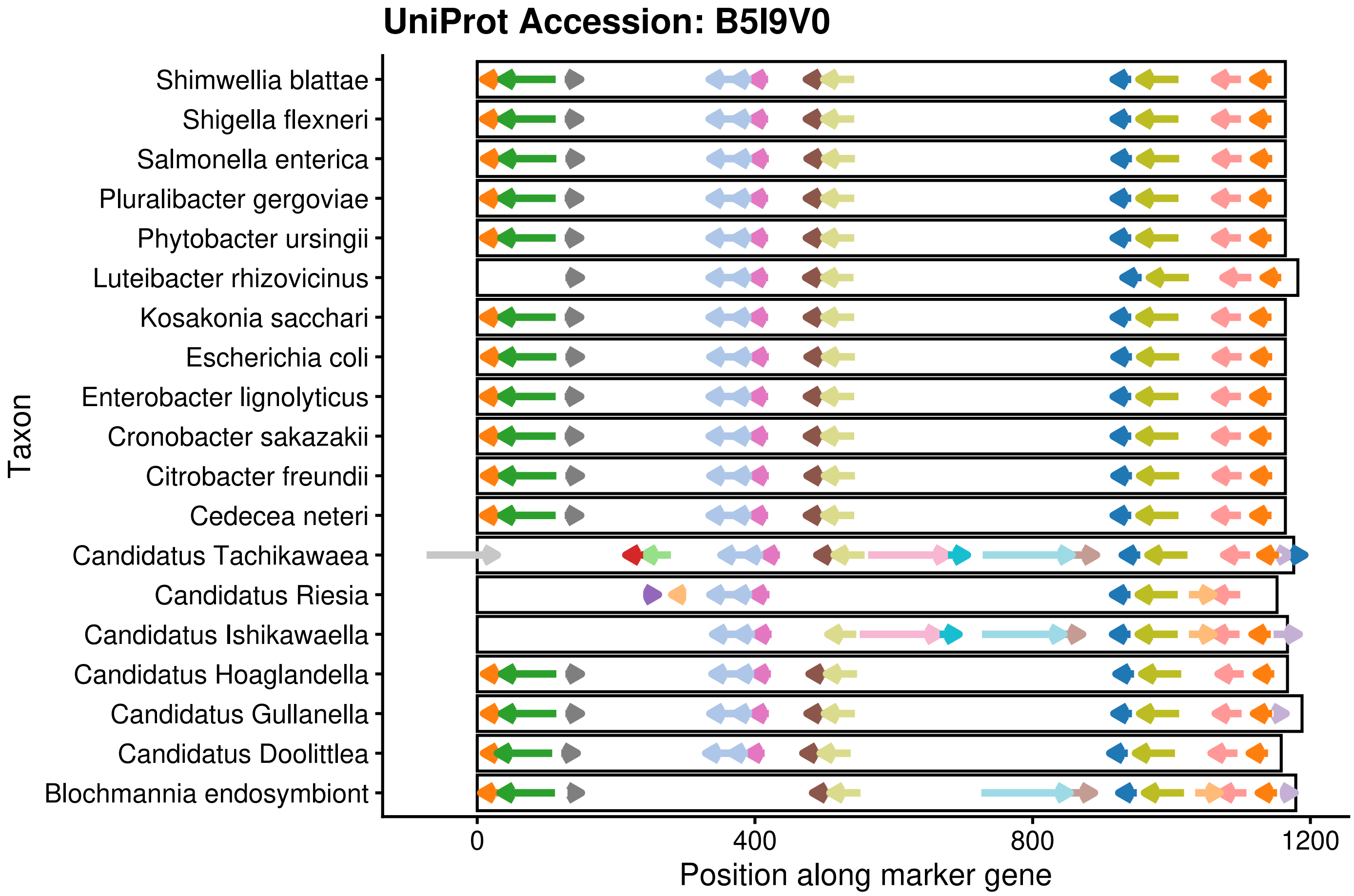
UniProt Accession: B5I9V0
Shimwellia blattae
Shigella flexneri
Salmonella enterica
Pluralibacter gergoviae
Phytobacter ursingii
Luteibacter rhizovicinus
Kosakonia sacchari
Escherichia coli
Enterobacter lignolyticus
Cronobacter sakazakii
Citrobacter freundii
Cedecea neteri
Candidatus Tachikawaea
Candidatus Riesia
Candidatus Ishikawaella
Candidatus Hoaglandella
Candidatus Gullanella
Candidatus Doolittlea
Blochmannia endosymbiont
Taxon
0
400
800
1200
Position along marker gene

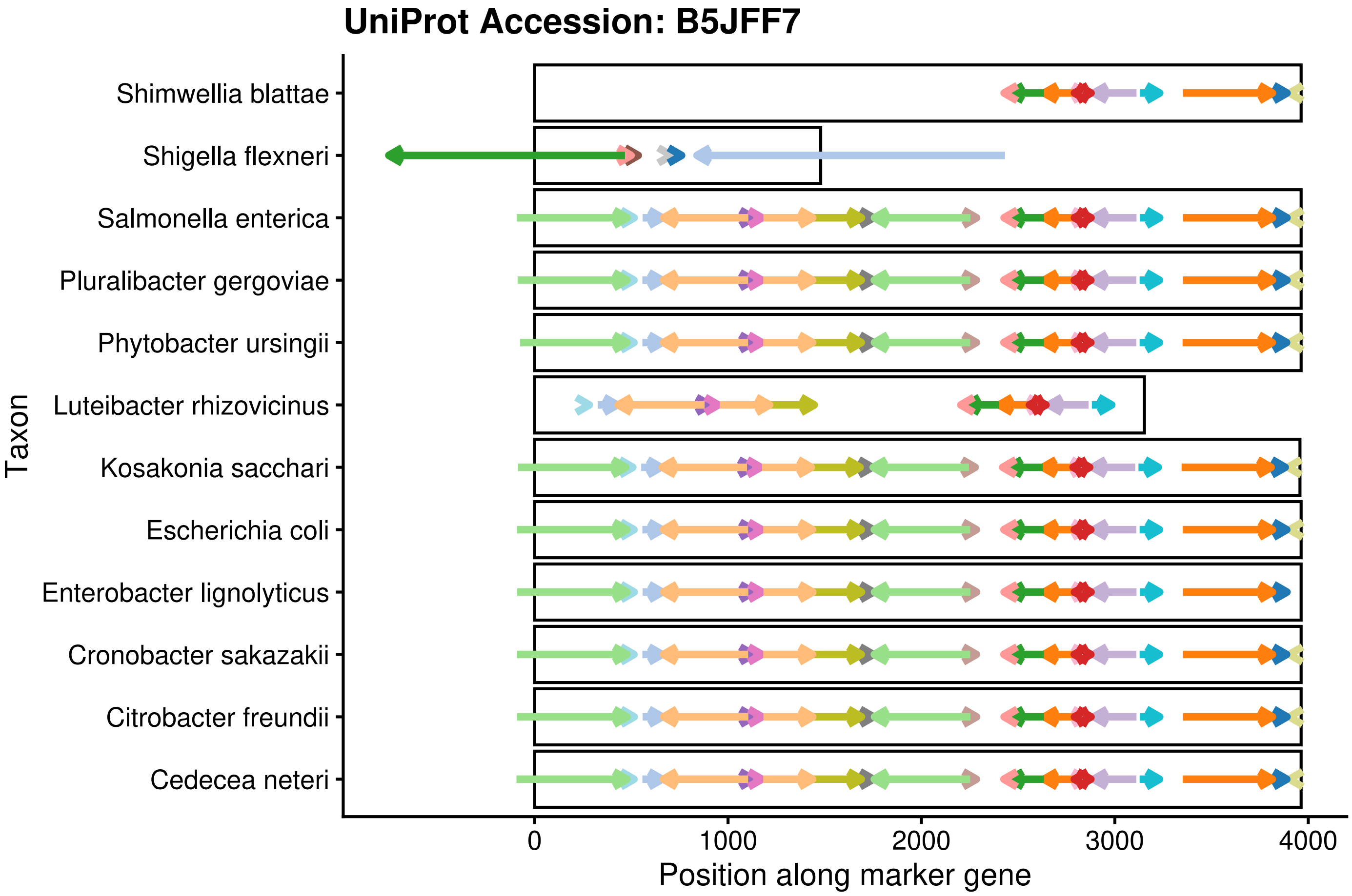

UniProt Accession: B5JFF7
Shimwellia blattae
Shigella flexneri
Salmonella enterica
Pluralibacter gergoviae
Phytobacter ursingii
Luteibacter rhizovicinus
Kosakonia sacchari
Escherichia coli
Enterobacter lignolyticus
Cronobacter sakazakii
Citrobacter freundii
Cedecea neteri
Taxon
0
1000
2000
3000
4000
Position along marker gene

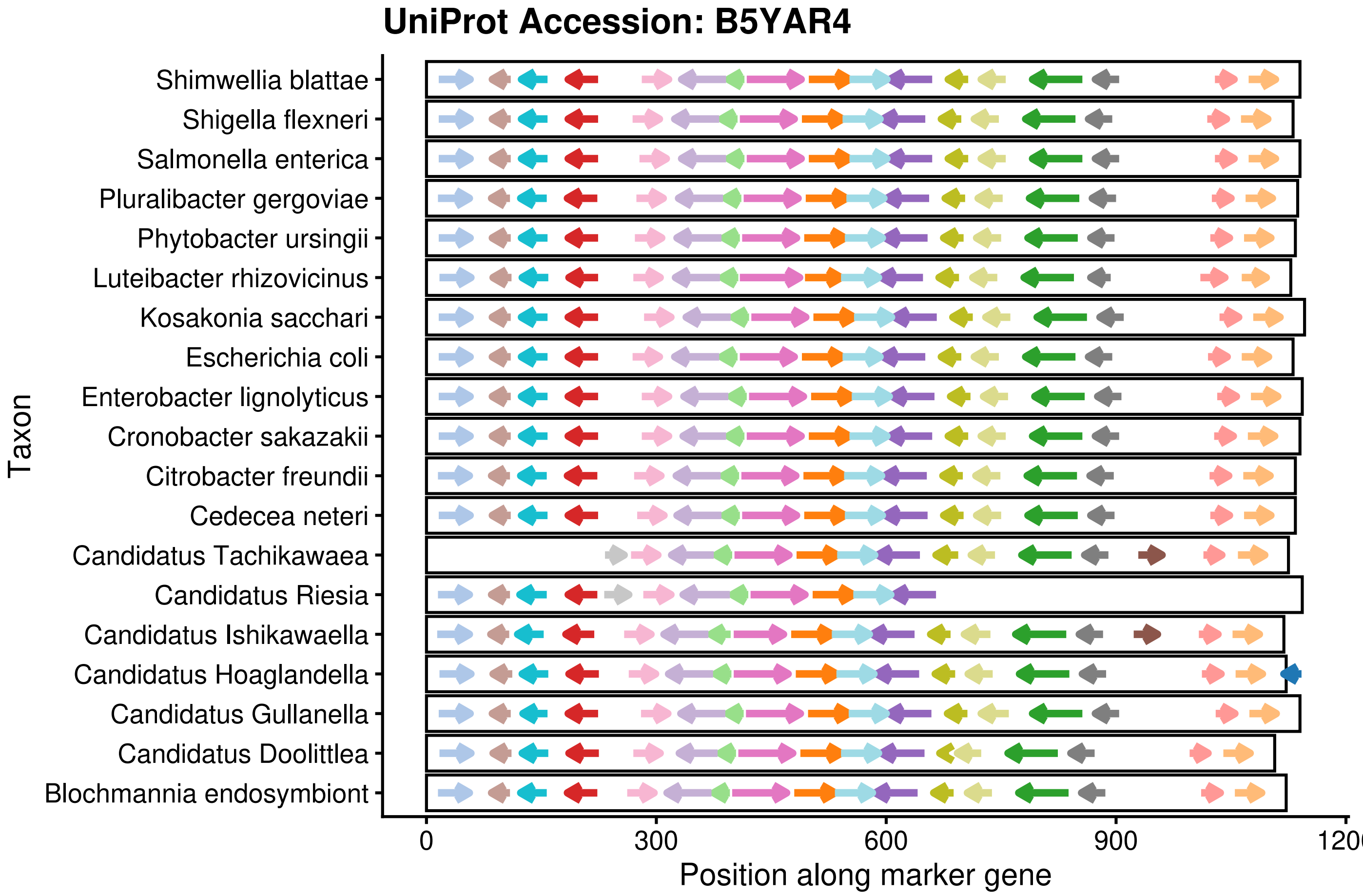
UniProt Accession: B5YAR4
Taxon
Shimwellia blattae
Shigella flexneri
Salmonella enterica
Pluralibacter gergoviae
Phytobacter ursingii
Luteibacter rhizovicinus
Kosakonia sacchari
Escherichia coli
Enterobacter lignolyticus
Cronobacter sakazakii
Citrobacter freundii
Cedecea neteri
Candidatus Tachikawaea
Candidatus Riesia
Candidatus Ishikawaella
Candidatus Hoaglandella
Candidatus Gullanella
Candidatus Doolittlea
Blochmannia endosymbiont
0
300
600
900
Position along marker gene

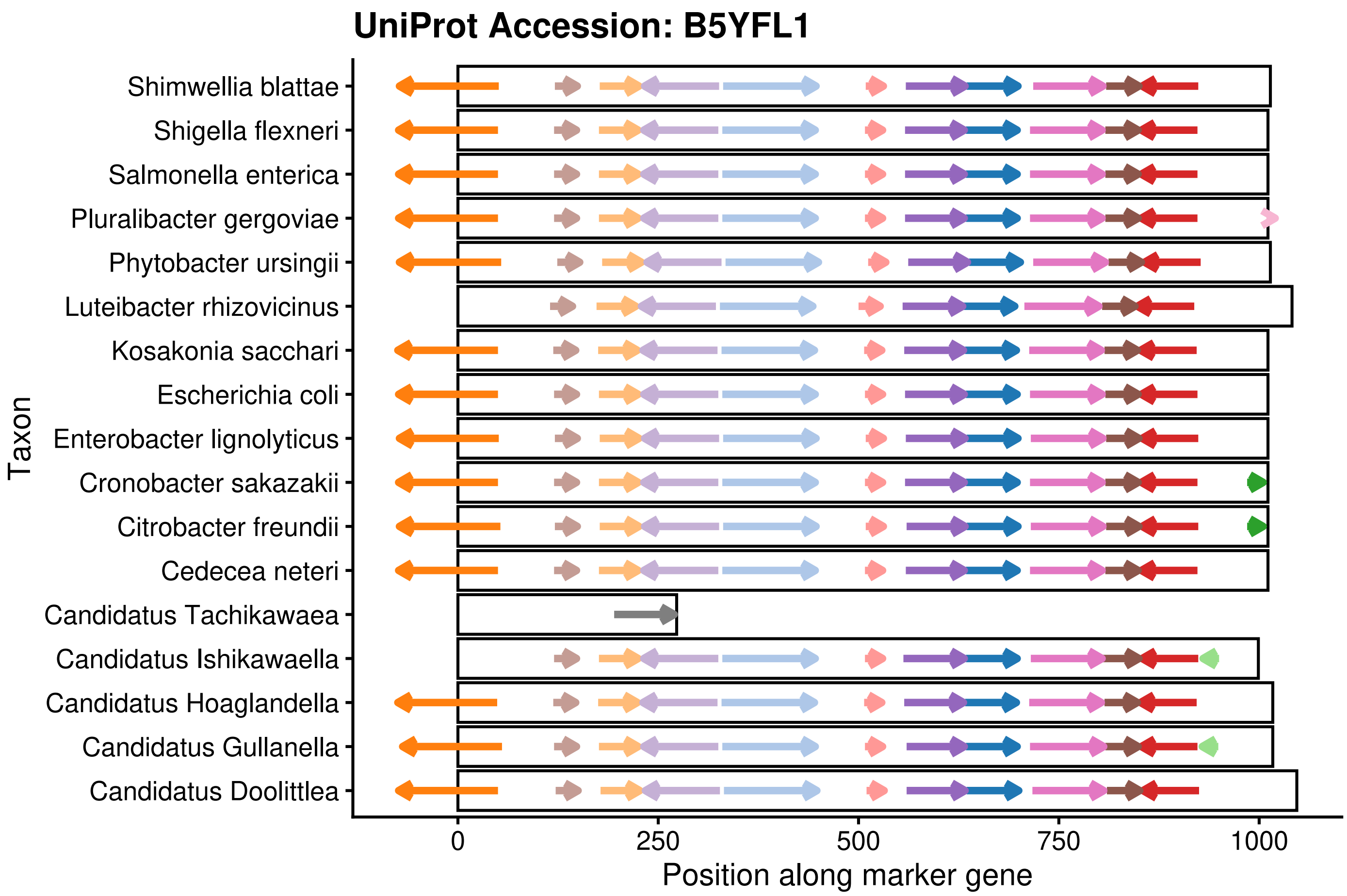

UniProt Accession: B5YFL1
Shimwellia blattae
Shigella flexneri
Salmonella enterica
Pluralibacter gergoviae
Phytobacter ursingii
Luteibacter rhizovicinus
Kosakonia sacchari
Escherichia coli
Enterobacter lignolyticus
Cronobacter sakazakii
Citrobacter freundii
Cedecea neteri
Candidatus Tachikawaea
Candidatus Ishikawaella
Candidatus Hoaglandella
Candidatus Gullanella
Candidatus Doolittlea
Taxon
0
250
500
750
1000
Position along marker gene

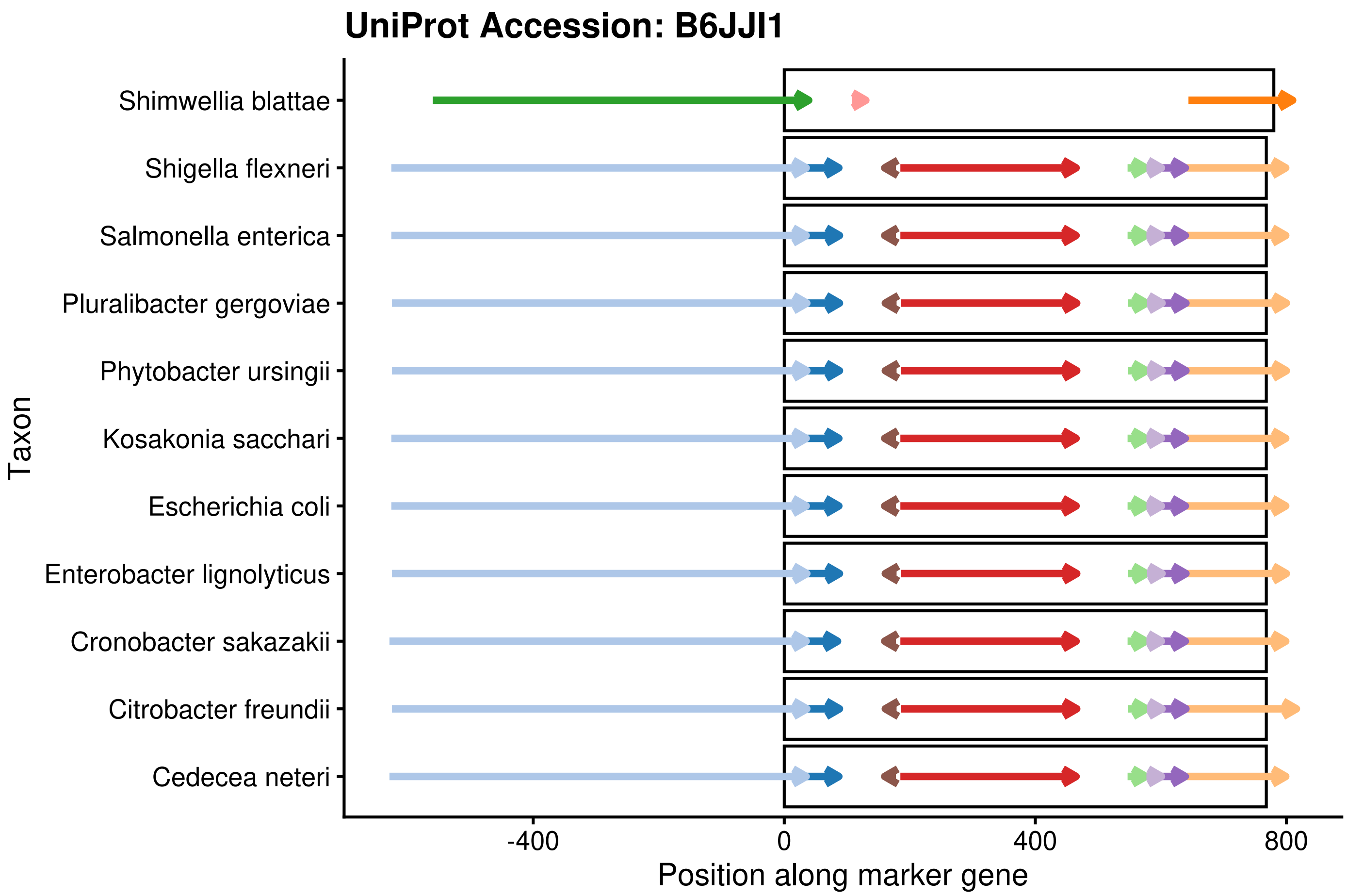
UniProt Accession: B6JJI1
Taxon
Shimwellia blattae
Shigella flexneri
Salmonella enterica
Pluralibacter gergoviae
Phytobacter ursingii
Kosakonia sacchari
Escherichia coli
Enterobacter lignolyticus
Cronobacter sakazakii
Citrobacter freundii
Cedecea neteri
-400
0
400
800
Position along marker gene

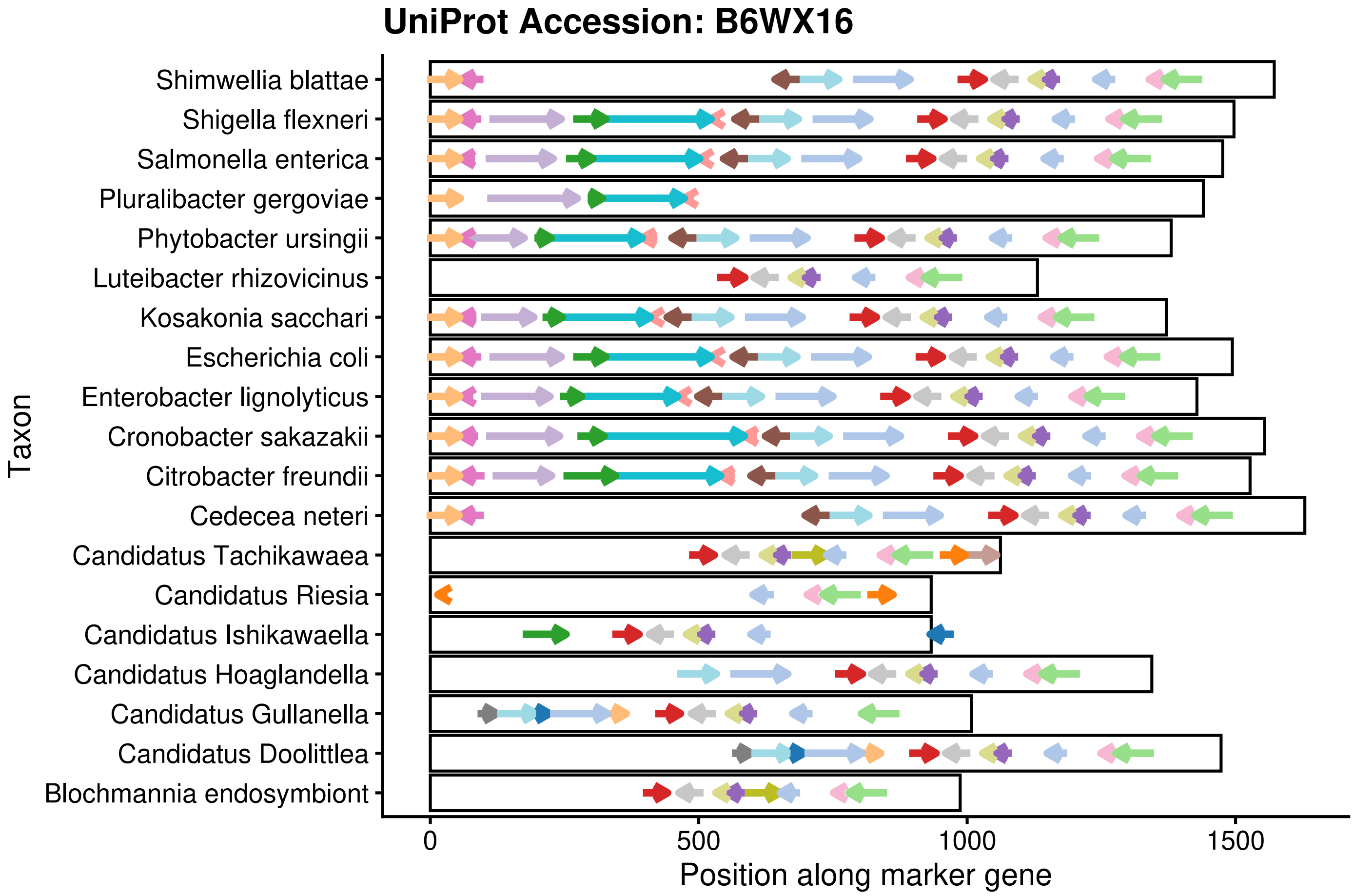

UniProt Accession: B6WX16
Taxon
Shimwellia blattae
Shigella flexneri
Salmonella enterica
Pluralibacter gergoviae
Phytobacter ursingii
Luteibacter rhizovicinus
Kosakonia sacchari
Escherichia coli
Enterobacter lignolyticus
Cronobacter sakazakii
Citrobacter freundii
Cedecea neteri
Candidatus Tachikawaea
Candidatus Riesia
Candidatus Ishikawaella
Candidatus Hoaglandella
Candidatus Gullanella
Candidatus Doolittlea
Blochmannia endosymbiont
0
500
1000
1500
Position along marker gene

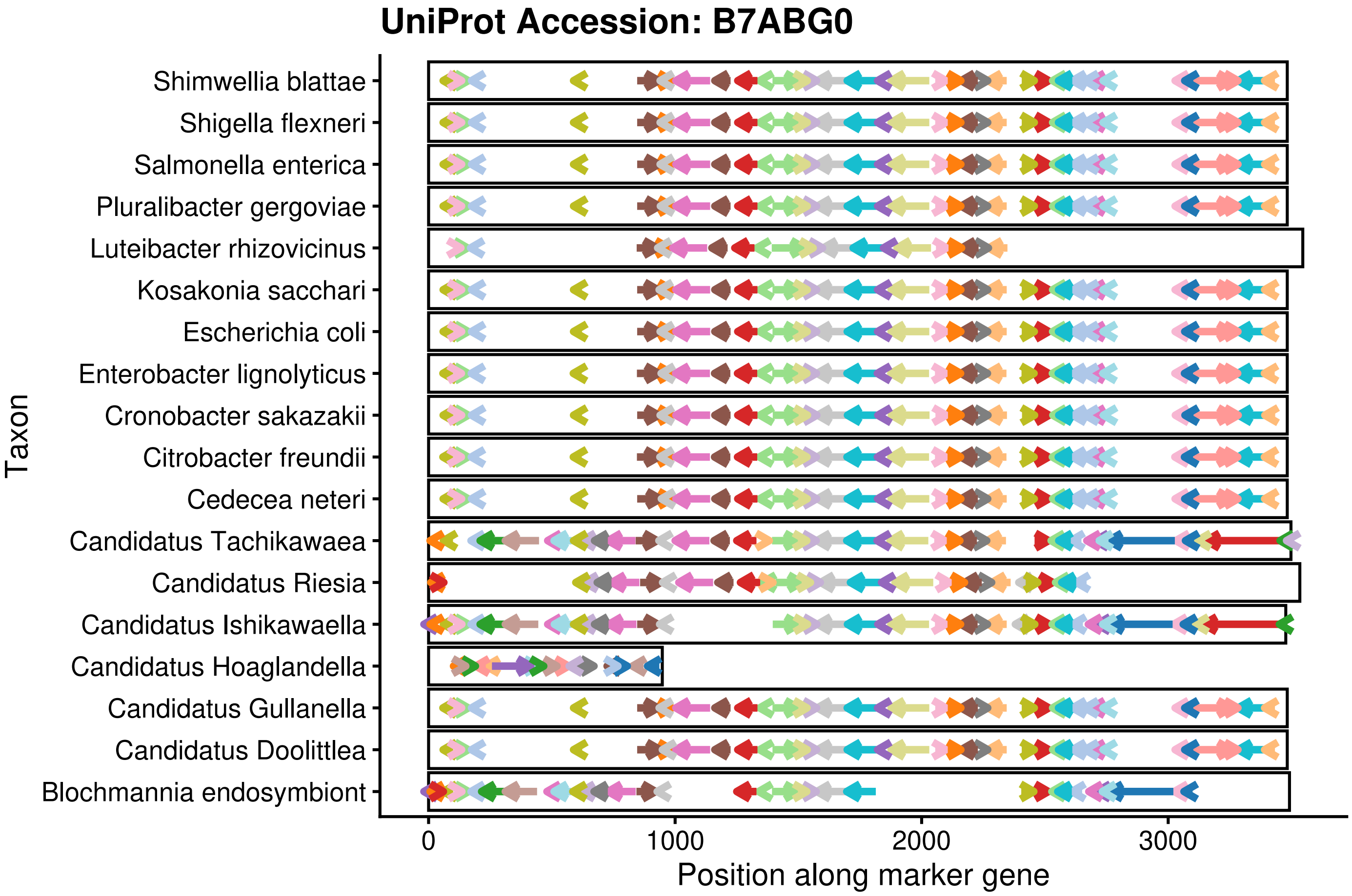
UniProt Accession: B7ABG0
Taxon
Shimwellia blattae
Shigella flexneri
Salmonella enterica
Pluralibacter gergoviae
Luteibacter rhizovicinus
Kosakonia sacchari
Escherichia coli
Enterobacter lignolyticus
Cronobacter sakazakii
Citrobacter freundii
Cedecea neteri
Candidatus Tachikawaea
Candidatus Riesia
Candidatus Ishikawaella
Candidatus Hoaglandella
Candidatus Gullanella
Candidatus Doolittlea
Blochmannia endosymbiont
0
1000
2000
3000
Position along marker gene

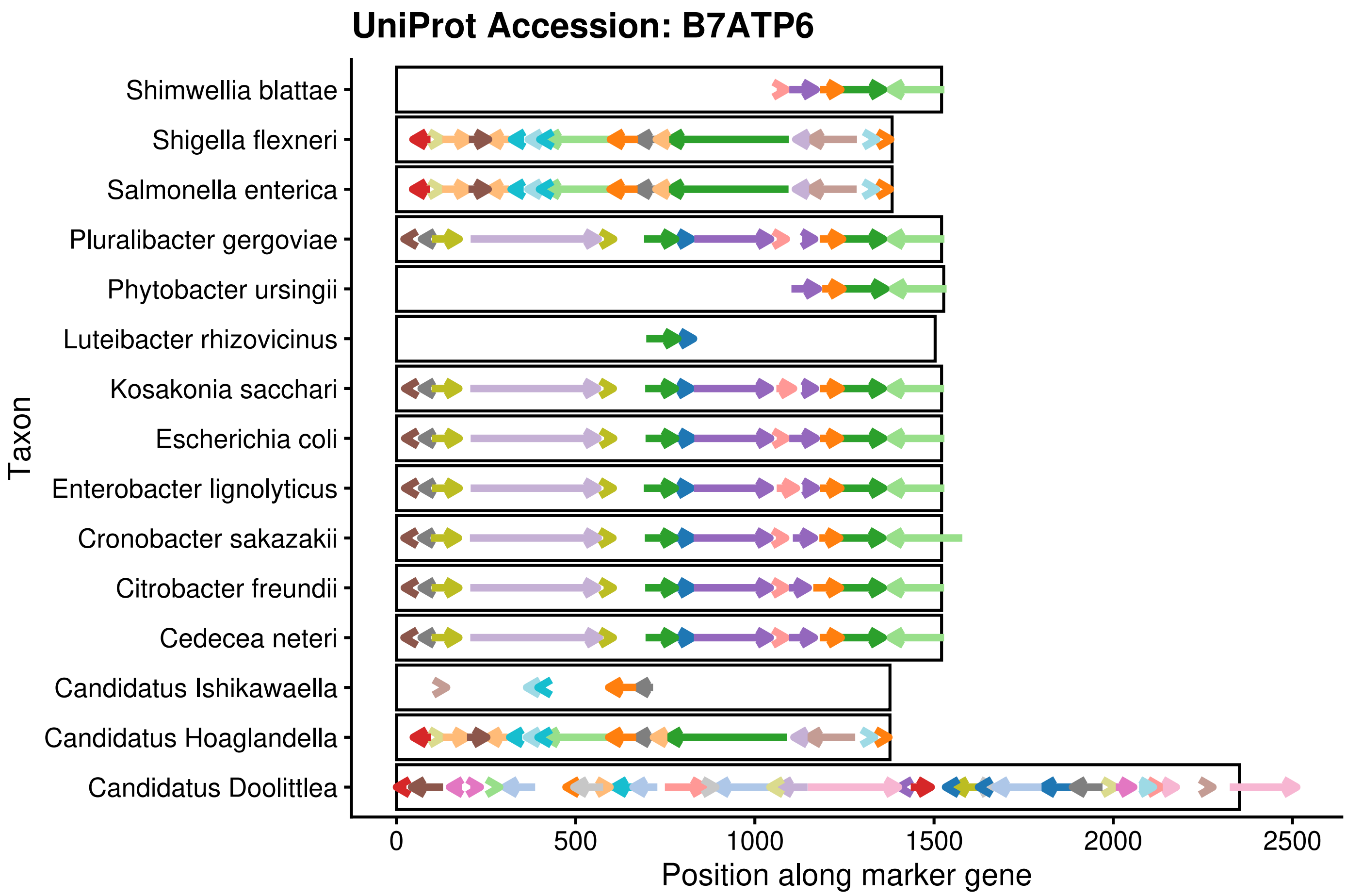
UniProt Accession: B7ATP6
Shimwellia blattae
Shigella flexneri
Salmonella enterica
Pluralibacter gergoviae
Phytobacter ursingii
Luteibacter rhizovicinus
Kosakonia sacchari
Escherichia coli
Enterobacter lignolyticus
Cronobacter sakazakii
Citrobacter freundii
Cedecea neteri
Candidatus Ishikawaella
Candidatus Hoaglandella
Candidatus Doolittlea
Taxon
0
500
1000
1500
2000
2500
Position along marker gene

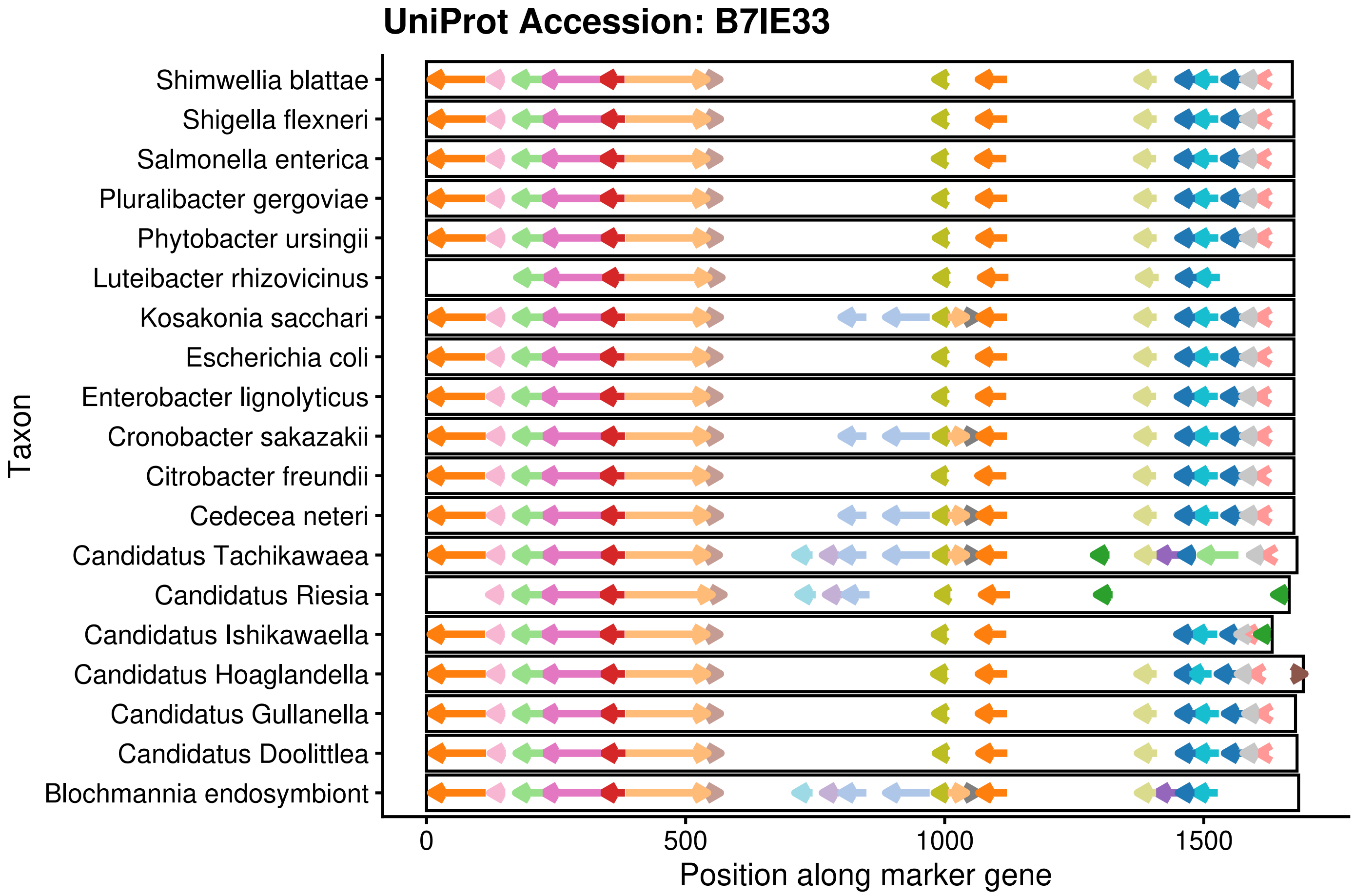
UniProt Accession: B7IE33
Taxon
Shimwellia blattae
Shigella flexneri
Salmonella enterica
Pluralibacter gergoviae
Phytobacter ursingii
Luteibacter rhizovicinus
Kosakonia sacchari
Escherichia coli
Enterobacter lignolyticus
Cronobacter sakazakii
Citrobacter freundii
Cedecea neteri
Candidatus Tachikawaea
Candidatus Riesia
Candidatus Ishikawaella
Candidatus Hoaglandella
Candidatus Gullanella
Candidatus Doolittlea
Blochmannia endosymbiont
0
500
1000
1500
Position along marker gene

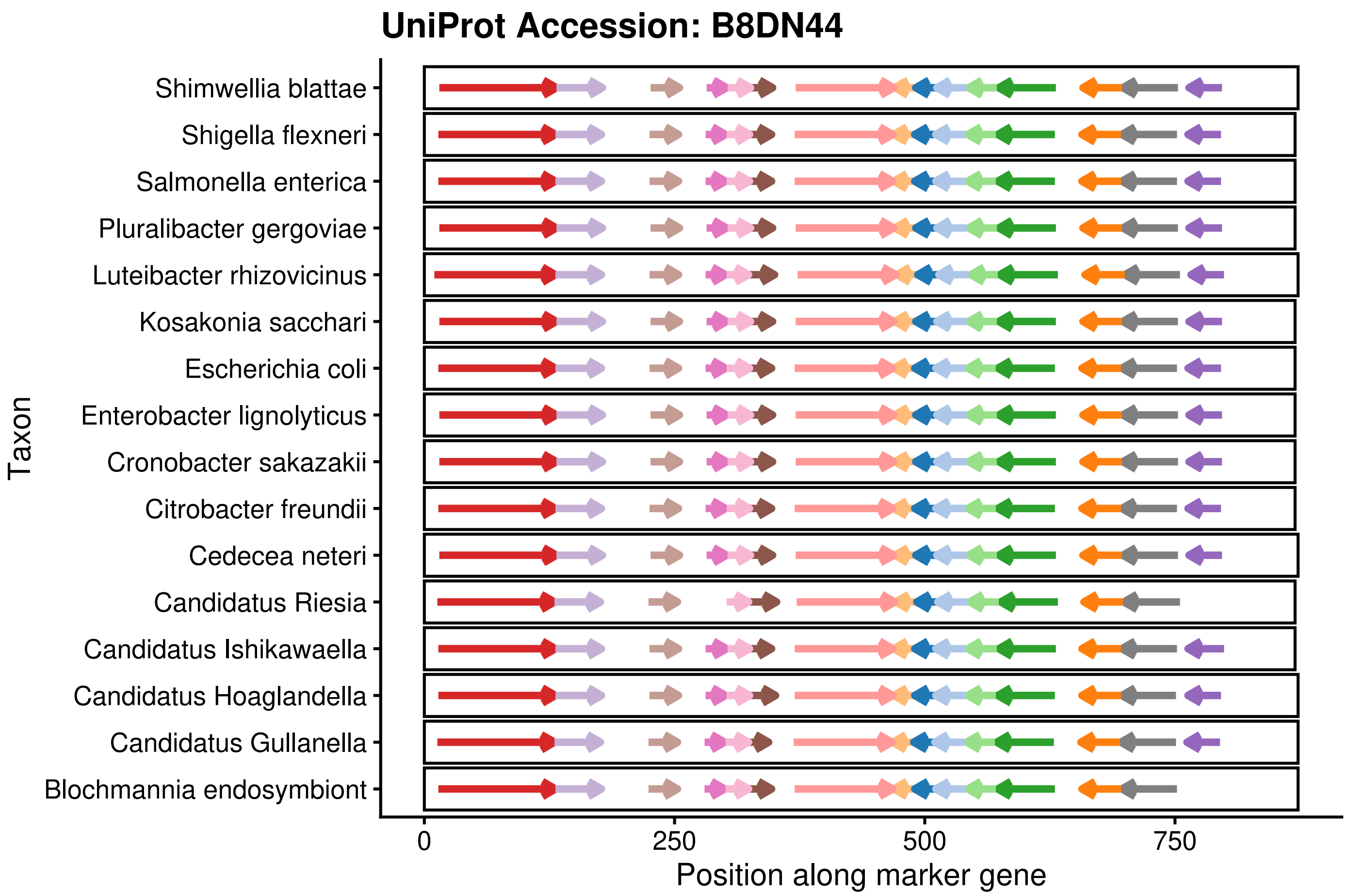

UniProt Accession: B8DN44
Taxon
Shimwellia blattae
Shigella flexneri
Salmonella enterica
Pluralibacter gergoviae
Luteibacter rhizovicinus
Kosakonia sacchari
Escherichia coli
Enterobacter lignolyticus
Cronobacter sakazakii
Citrobacter freundii
Cedecea neteri
Candidatus Riesia
Candidatus Ishikawaella
Candidatus Hoaglandella
Candidatus Gullanella
Blochmannia endosymbiont
0
250
500
750
Position along marker gene

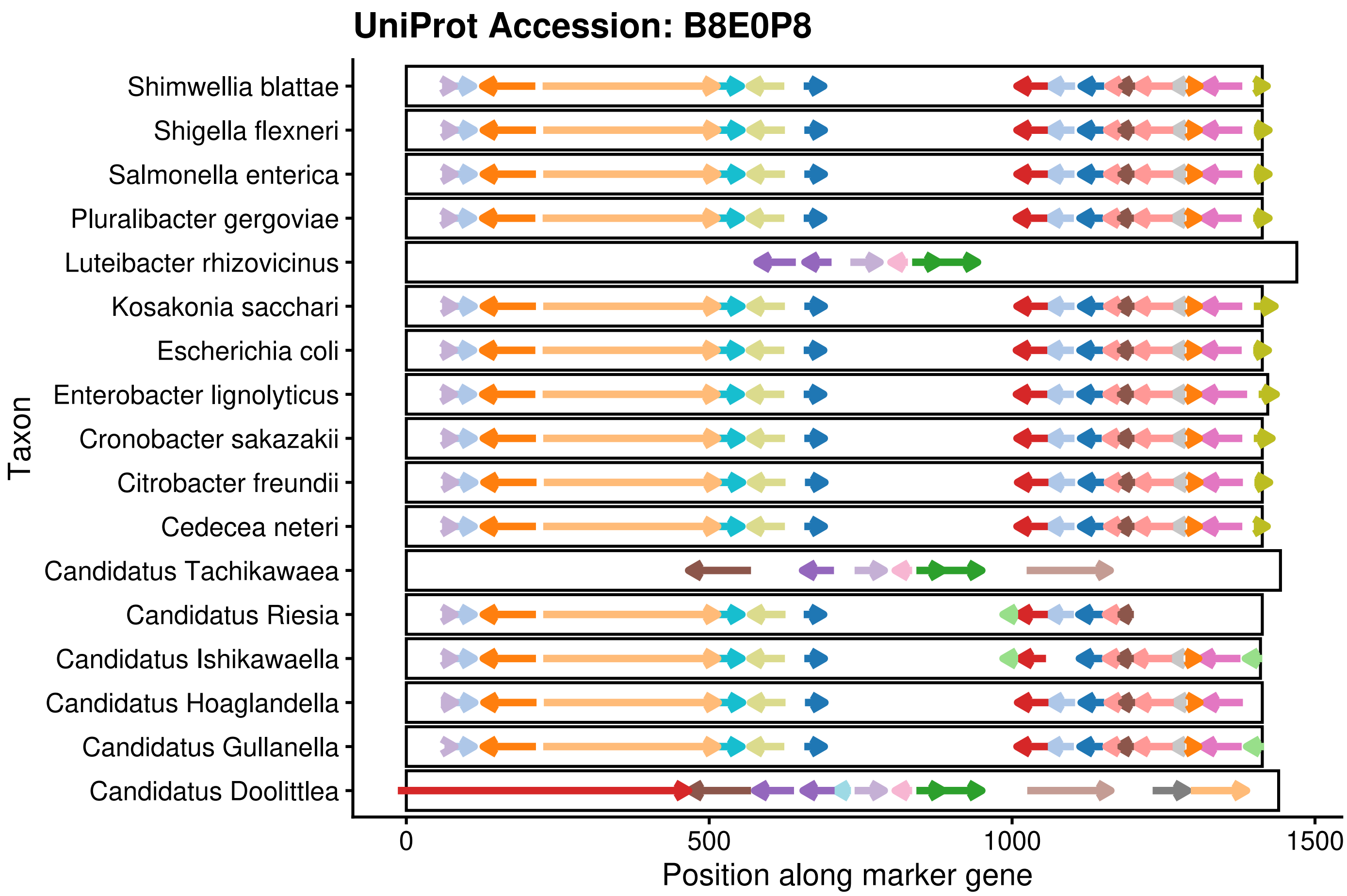
UniProt Accession: B8E0P8
Taxon
Shimwellia blattae
Shigella flexneri
Salmonella enterica
Pluralibacter gergoviae
Luteibacter rhizovicinus
Kosakonia sacchari
Escherichia coli
Enterobacter lignolyticus
Cronobacter sakazakii
Citrobacter freundii
Cedecea neteri
Candidatus Tachikawaea
Candidatus Riesia
Candidatus Ishikawaella
Candidatus Hoaglandella
Candidatus Gullanella
Candidatus Doolittlea
0
500
1000
1500
Position along marker gene

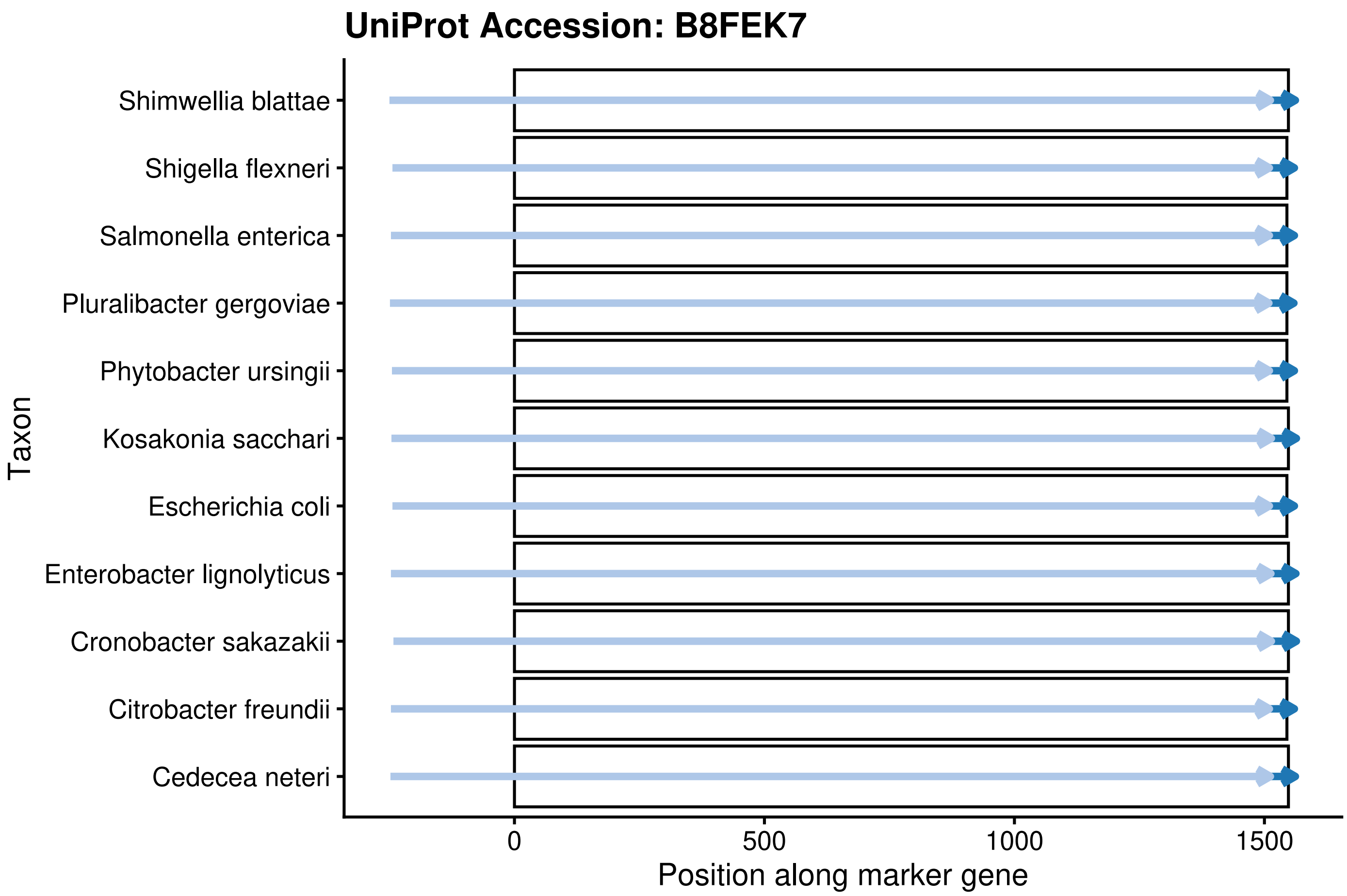
UniProt Accession: B8FEK7
Shimwellia blattae
Shigella flexneri
Salmonella enterica
Pluralibacter gergoviae
Phytobacter ursingii
Kosakonia sacchari
Escherichia coli
Enterobacter lignolyticus
Cronobacter sakazakii
Citrobacter freundii
Cedecea neteri
Taxon
0
500
1000
1500
Position along marker gene

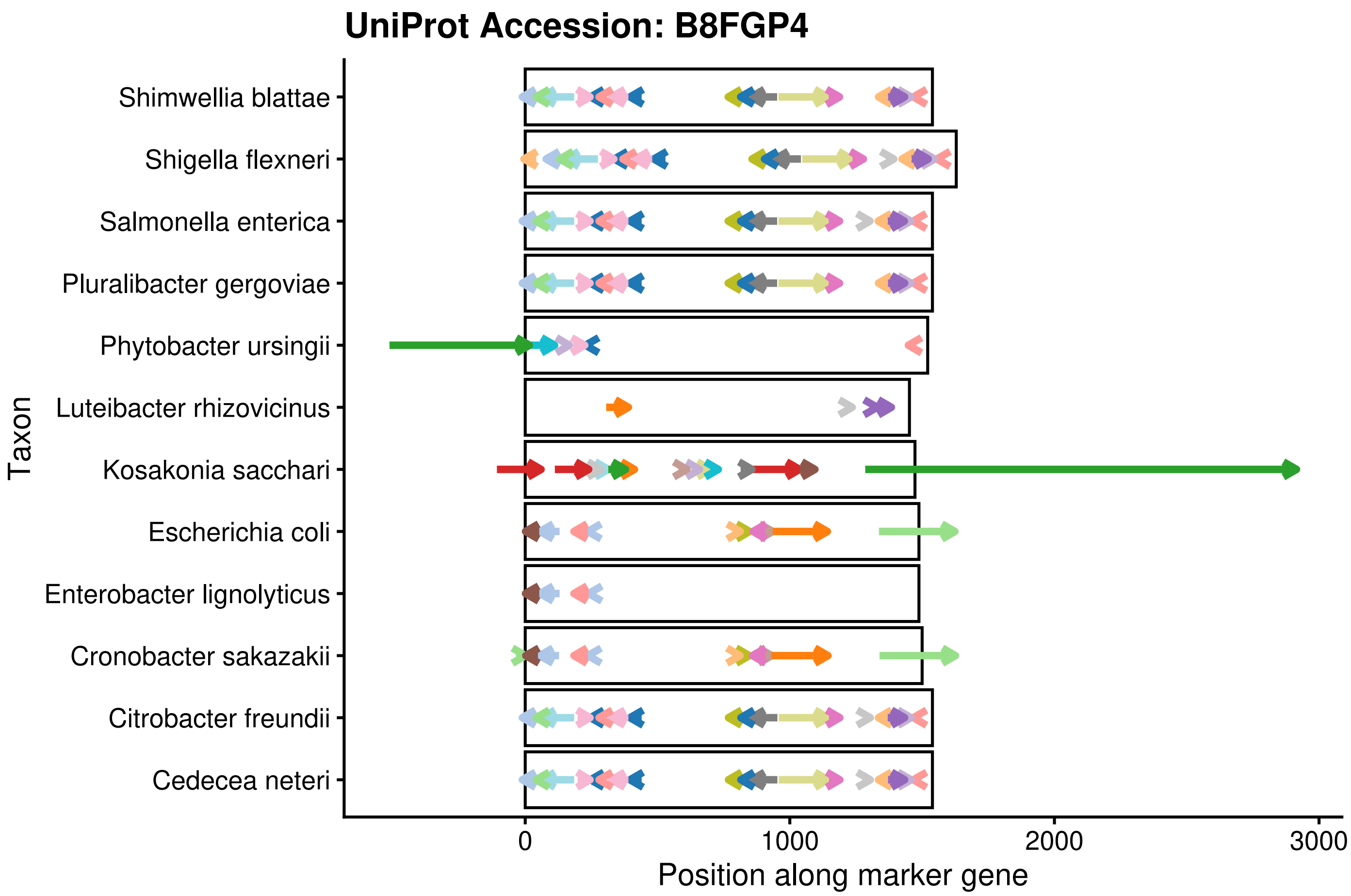
UniProt Accession: B8FGP4
Shimwellia blattae
Shigella flexneri
Salmonella enterica
Pluralibacter gergoviae
Phytobacter ursingii
Luteibacter rhizovicinus
Kosakonia sacchari
Escherichia coli
Enterobacter lignolyticus
Cronobacter sakazakii
Citrobacter freundii
Cedecea neteri
Taxon
0
1000
2000
3000
Position along marker gene

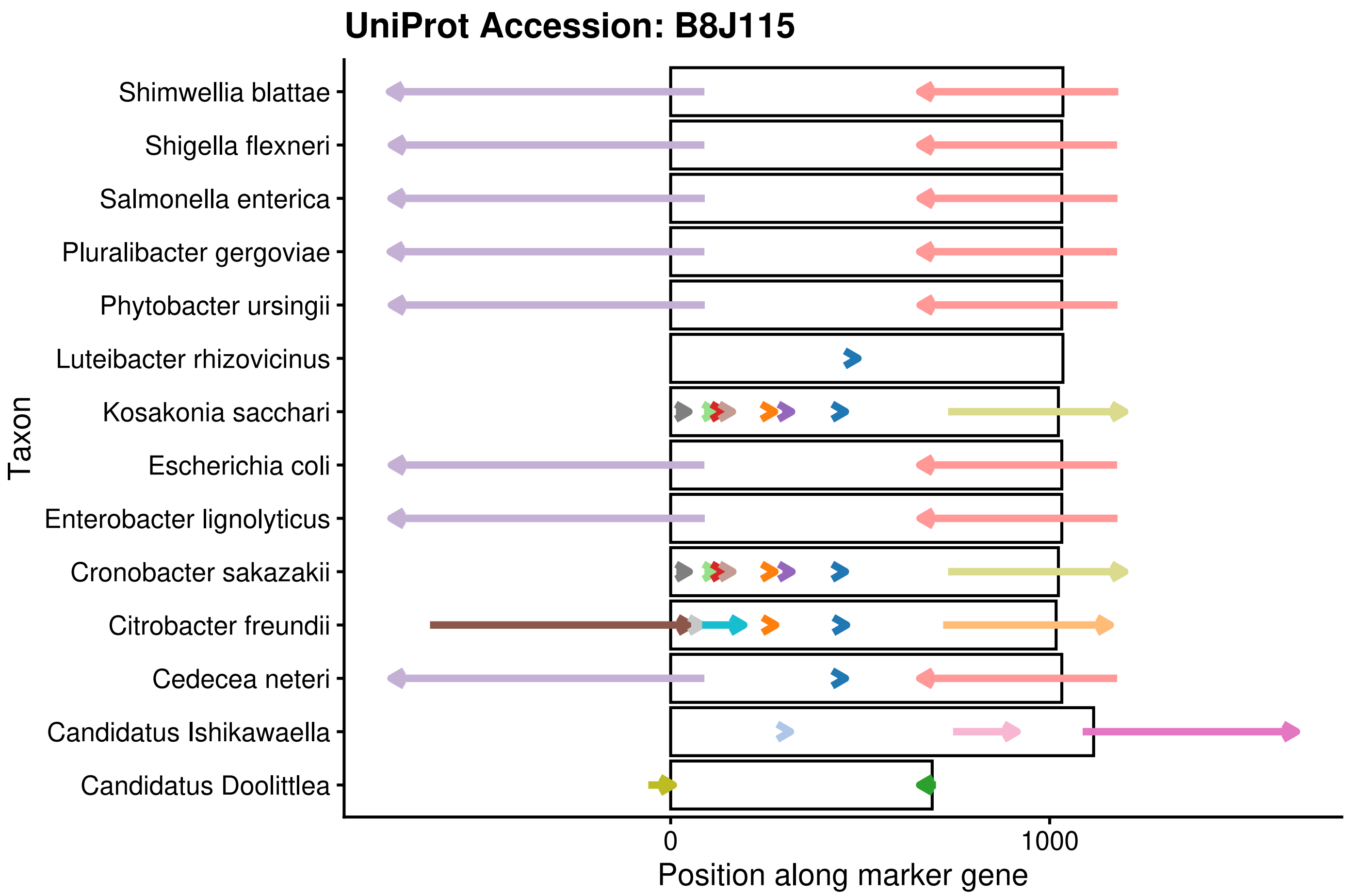
UniProt Accession: B8J115
Shimwellia blattae
Shigella flexneri
Salmonella enterica
Pluralibacter gergoviae
Phytobacter ursingii
Luteibacter rhizovicinus
Kosakonia sacchari
Escherichia coli
Enterobacter lignolyticus
Cronobacter sakazakii
Citrobacter freundii
Cedecea neteri
Candidatus Ishikawaella
Candidatus Doolittlea
Taxon
0
1000
Position along marker gene

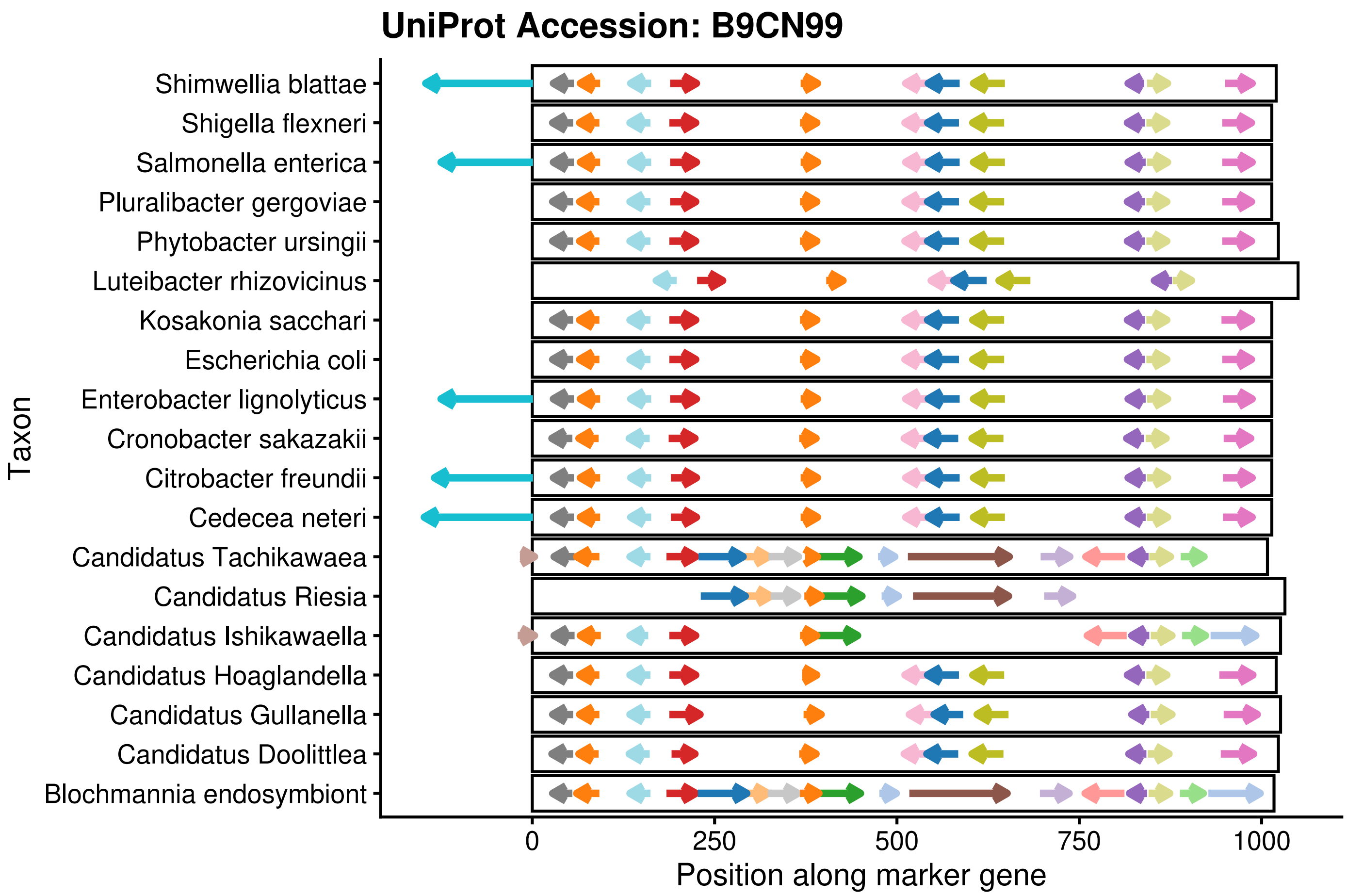
UniProt Accession: B9CN99
Shimwellia blattae
Shigella flexneri
Salmonella enterica
Pluralibacter gergoviae
Phytobacter ursingii
Luteibacter rhizovicinus
Kosakonia sacchari
Escherichia coli
Enterobacter lignolyticus
Cronobacter sakazakii
Citrobacter freundii
Cedecea neteri
Candidatus Tachikawaea
Candidatus Riesia
Candidatus Ishikawaella
Candidatus Hoaglandella
Candidatus Gullanella
Candidatus Doolittlea
Blochmannia endosymbiont
Taxon
0
250
500
750
1000
Position along marker gene

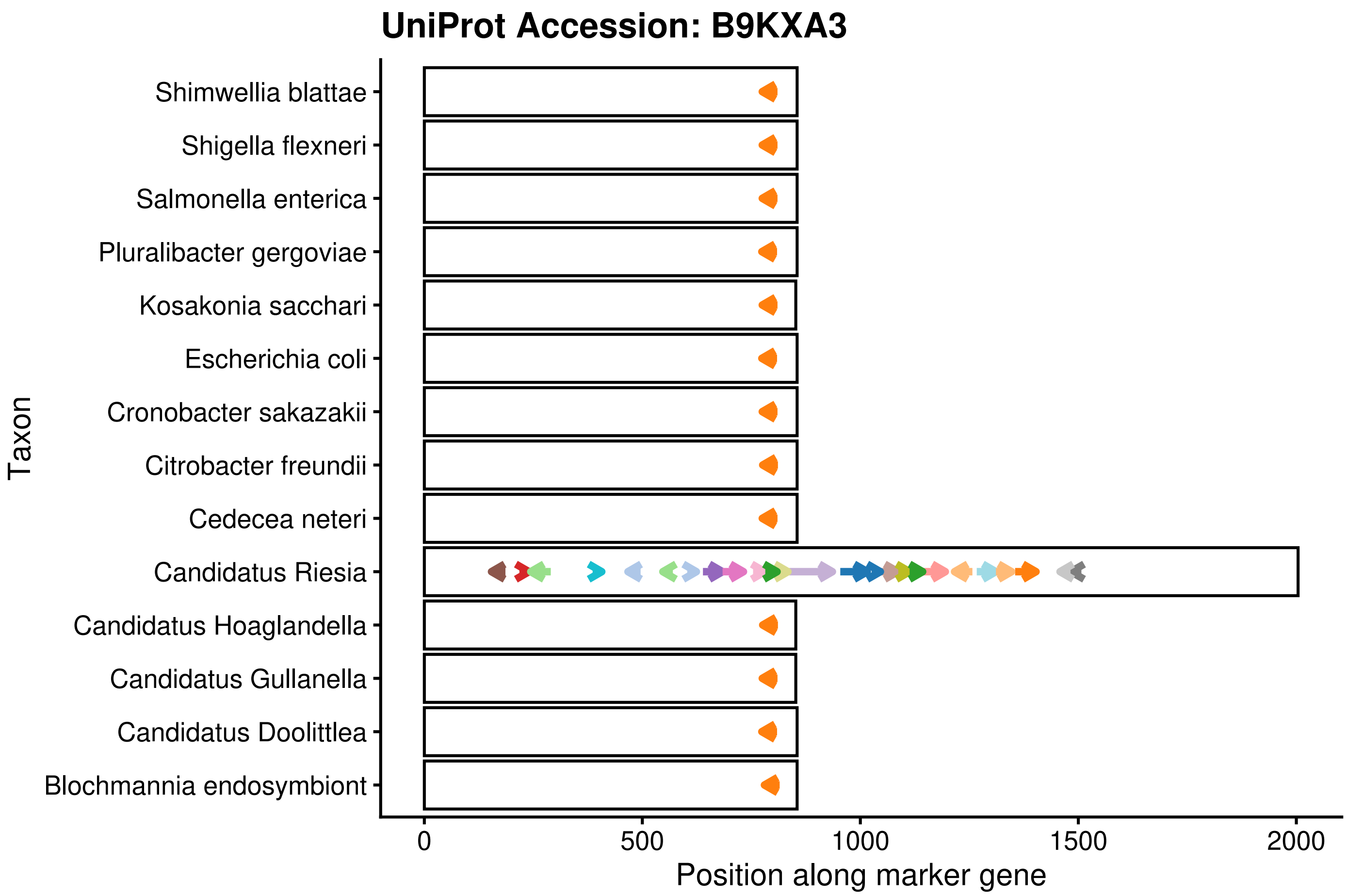
UniProt Accession: B9KXA3
Taxon
Shimwellia blattae
Shigella flexneri
Salmonella enterica
Pluralibacter gergoviae
Kosakonia sacchari
Escherichia coli
Cronobacter sakazakii
Citrobacter freundii
Cedecea neteri
Candidatus Riesia
Candidatus Hoaglandella
Candidatus Gullanella
Candidatus Doolittlea
Blochmannia endosymbiont
0
500
1000
1500
2000
Position along marker gene

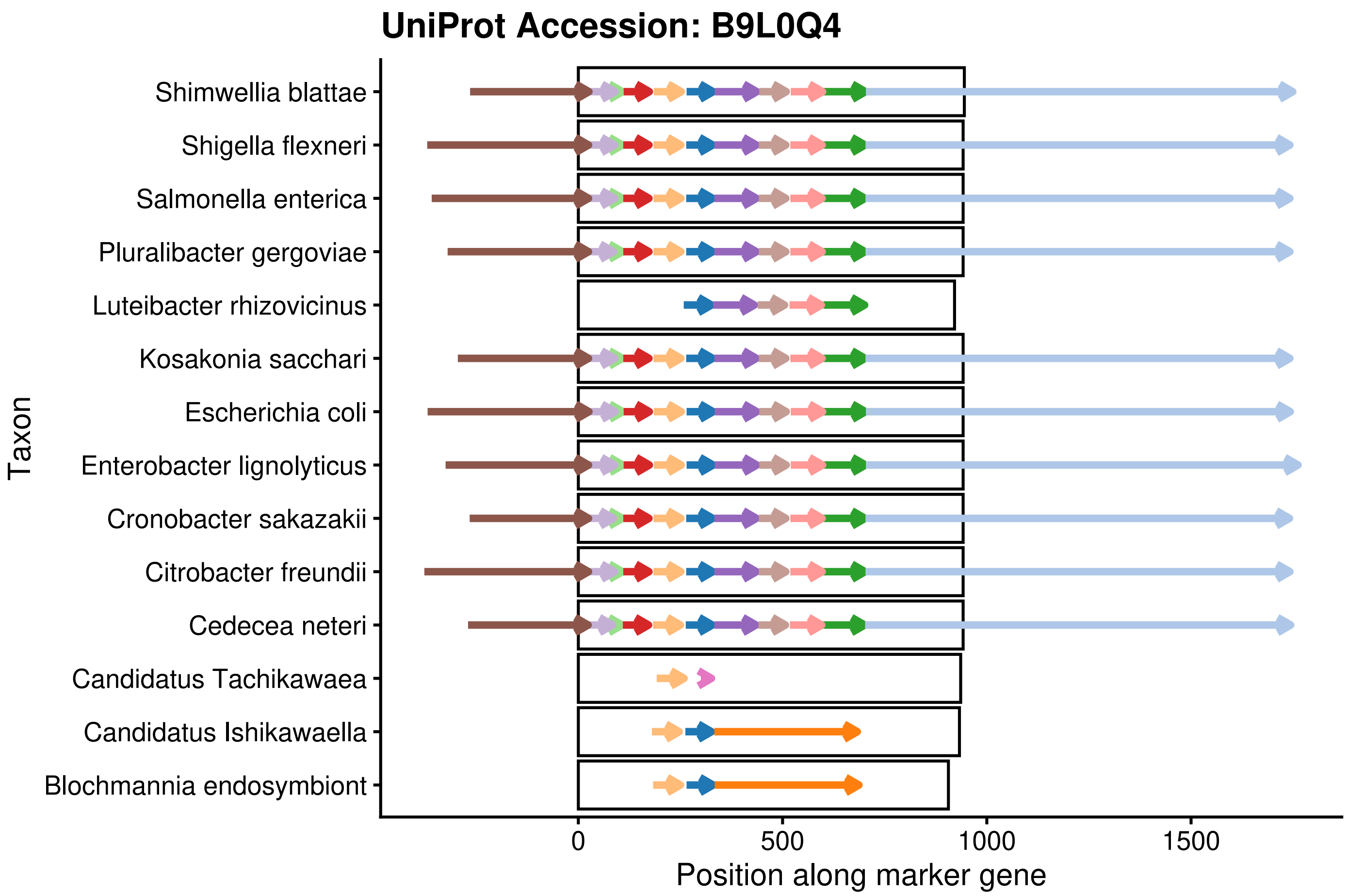
UniProt Accession: B9L0Q4
Taxon
Shimwellia blattae
Shigella flexneri
Salmonella enterica
Pluralibacter gergoviae
Luteibacter rhizovicinus
Kosakonia sacchari
Escherichia coli
Enterobacter lignolyticus
Cronobacter sakazakii
Citrobacter freundii
Cedecea neteri
Candidatus Tachikawaea
Candidatus Ishikawaella
Blochmannia endosymbiont
0
500
1000
1500
Position along marker gene

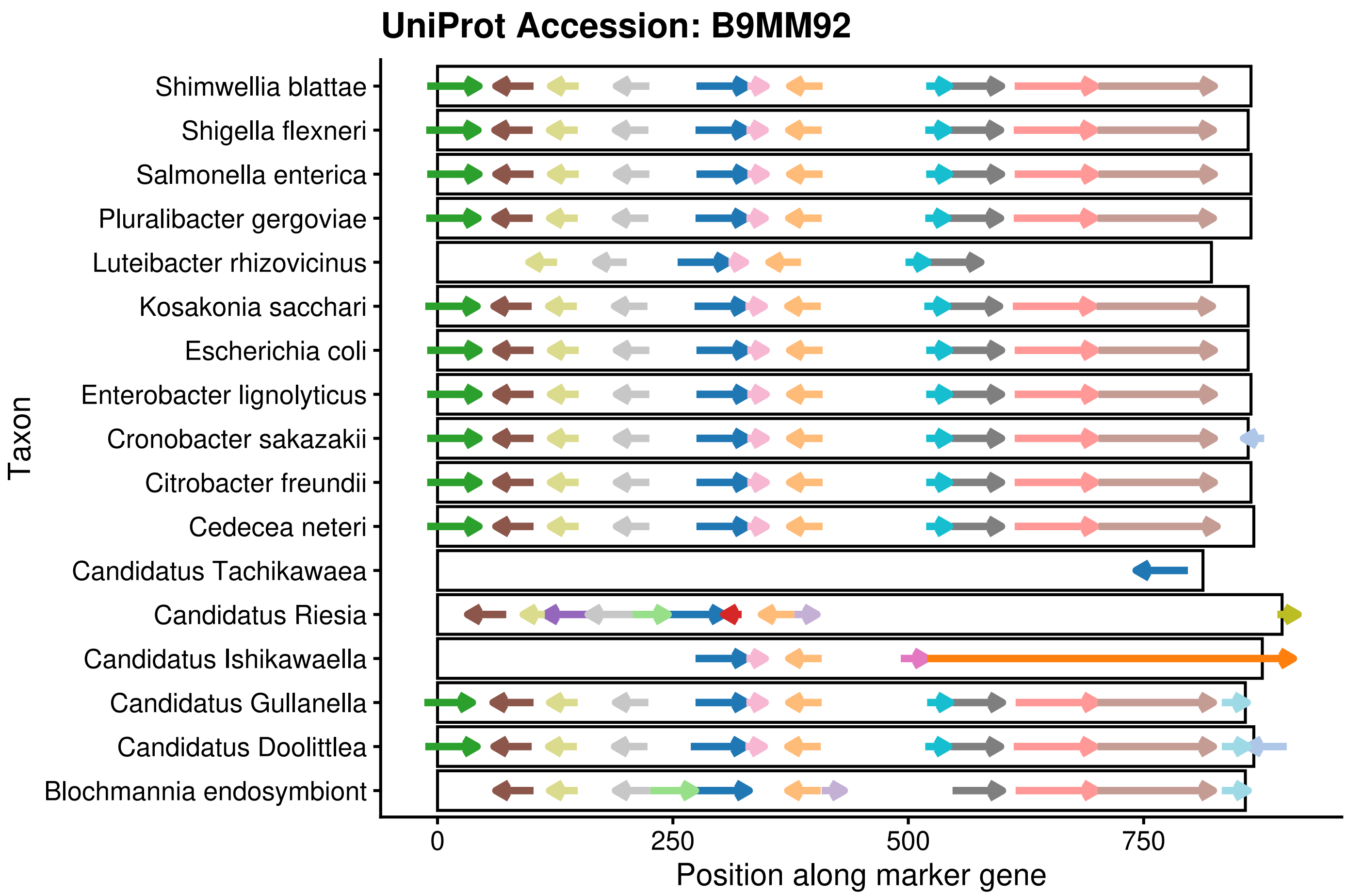
UniProt Accession: B9MM92
Taxon
Shimwellia blattae
Shigella flexneri
Salmonella enterica
Pluralibacter gergoviae
Luteibacter rhizovicinus
Kosakonia sacchari
Escherichia coli
Enterobacter lignolyticus
Cronobacter sakazakii
Citrobacter freundii
Cedecea neteri
Candidatus Tachikawaea
Candidatus Riesia
Candidatus Ishikawaella
Candidatus Gullanella
Candidatus Doolittlea
Blochmannia endosymbiont
0
250
500
750
Position along marker gene

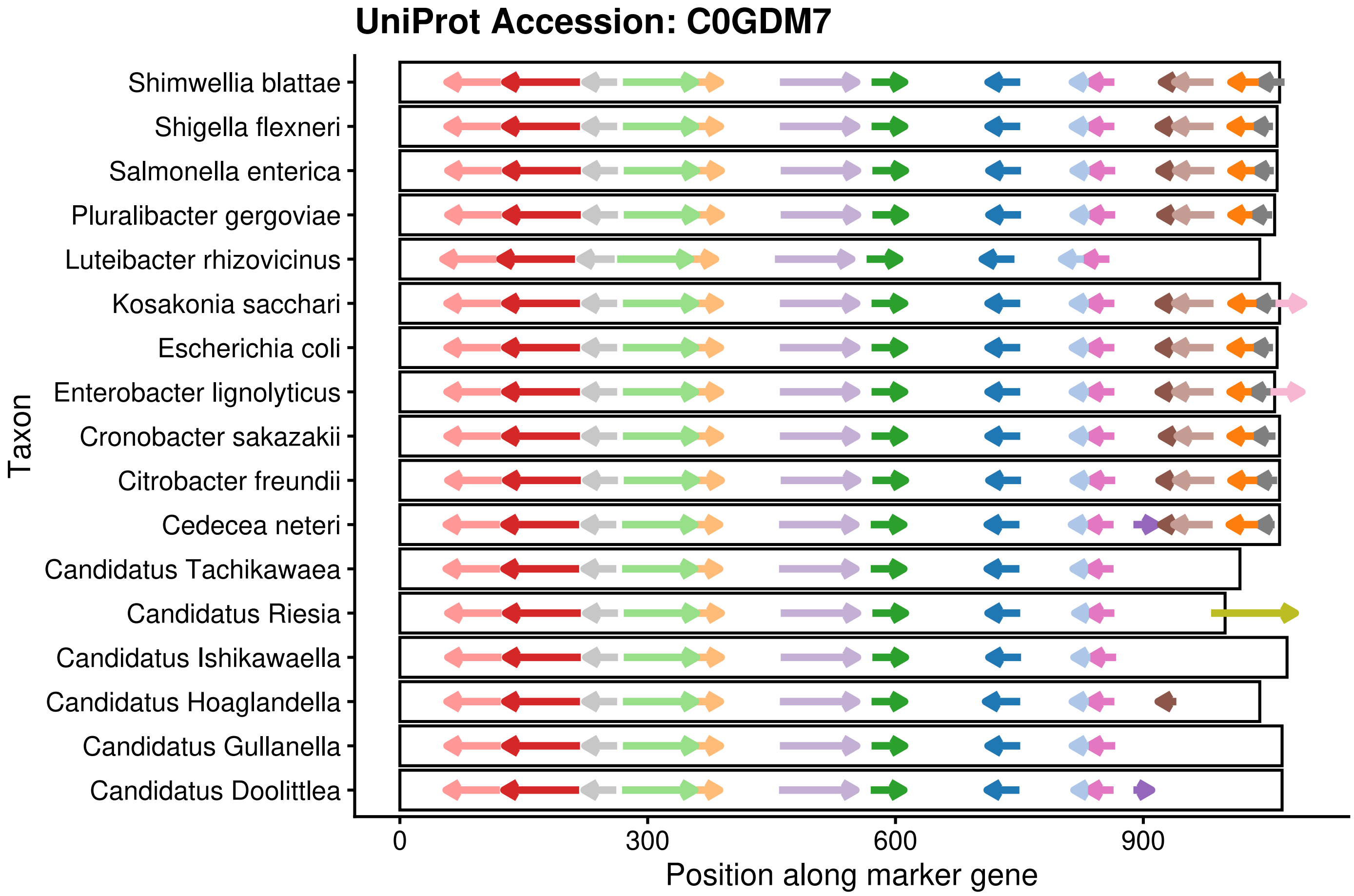

UniProt Accession: C0GDM7
Shimwellia blattae
Shigella flexneri
Salmonella enterica
Pluralibacter gergoviae
Luteibacter rhizovicinus
Kosakonia sacchari
Escherichia coli
Enterobacter lignolyticus
Cronobacter sakazakii
Citrobacter freundii
Cedecea neteri
Candidatus Tachikawaea
Candidatus Riesia
Candidatus Ishikawaella
Candidatus Hoaglandella
Candidatus Gullanella
Candidatus Doolittlea
Taxon
0
300
600
900
Position along marker gene

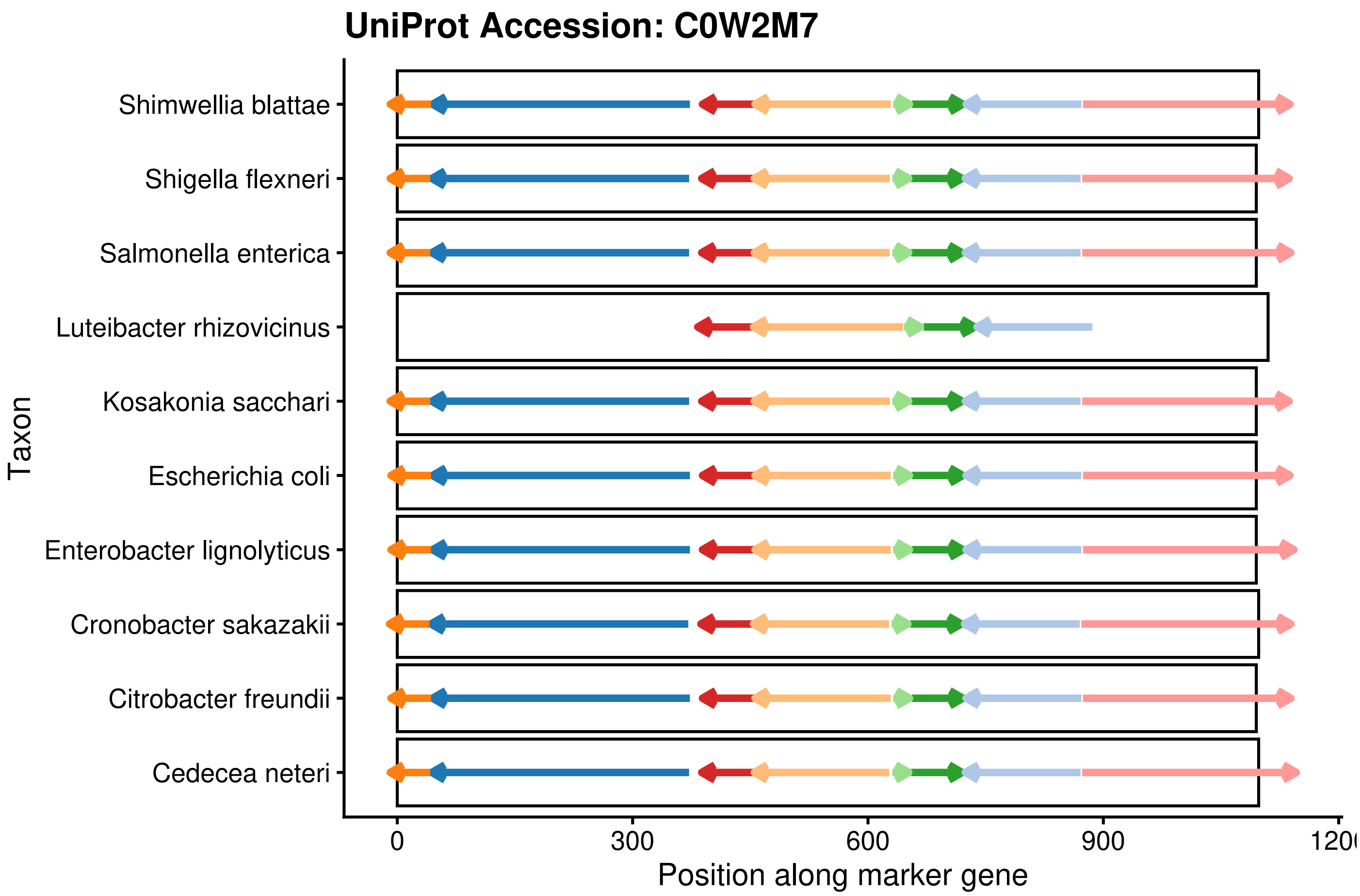

UniProt Accession: C0W2M7
Taxon
Shimwellia blattae
Shigella flexneri
Salmonella enterica
Luteibacter rhizovicinus
Kosakonia sacchari
Escherichia coli
Enterobacter lignolyticus
Cronobacter sakazakii
Citrobacter freundii
Cedecea neteri
0
300
600
900
Position along marker gene

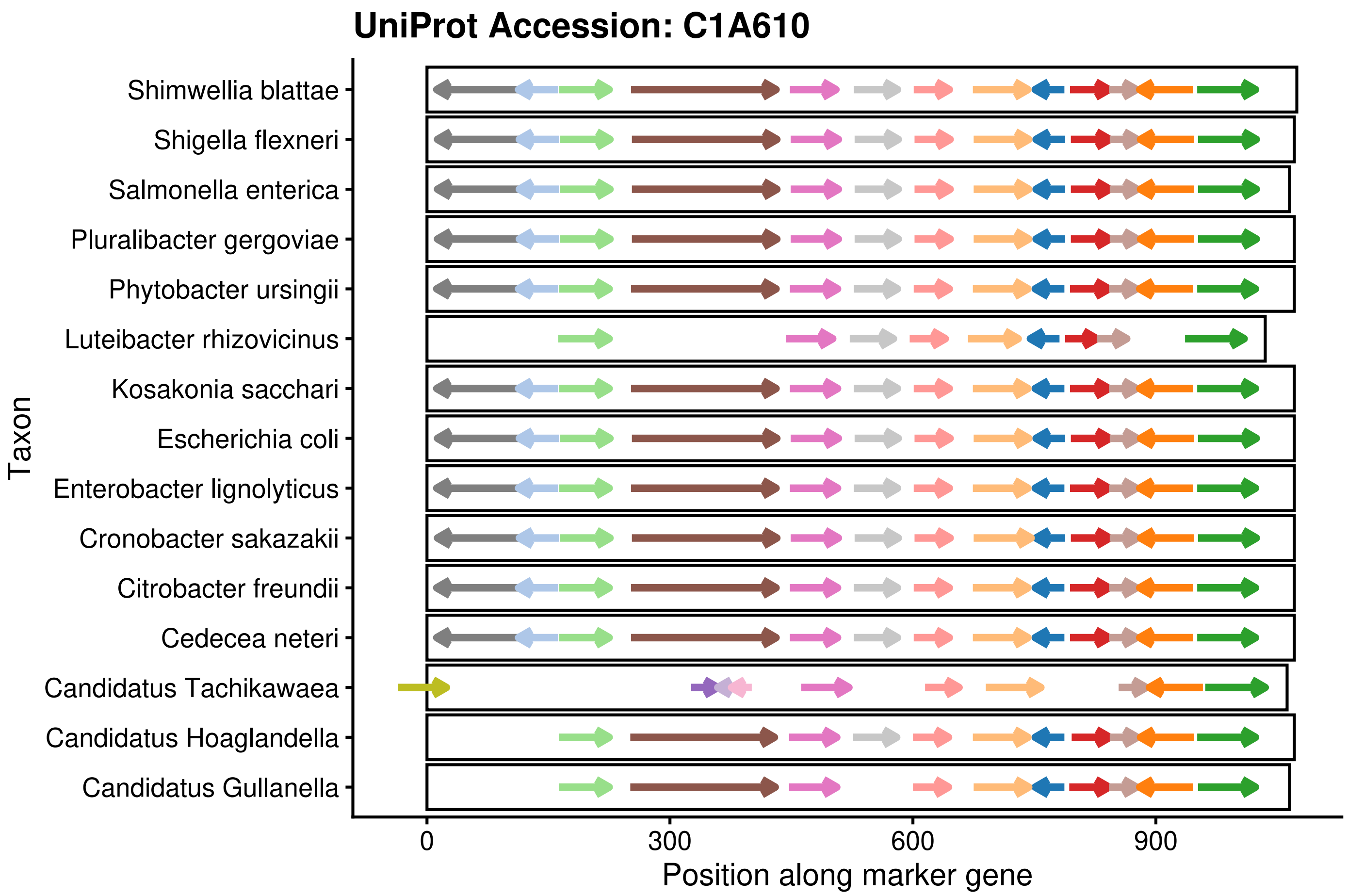
UniProt Accession: C1A610
Shimwellia blattae
Shigella flexneri
Salmonella enterica
Pluralibacter gergoviae
Phytobacter ursingii
Luteibacter rhizovicinus
Kosakonia sacchari
Escherichia coli
Enterobacter lignolyticus
Cronobacter sakazakii
Citrobacter freundii
Cedecea neteri
Candidatus Tachikawaea
Candidatus Hoaglandella
Candidatus Gullanella
Taxon
0
300
600
900
Position along marker gene

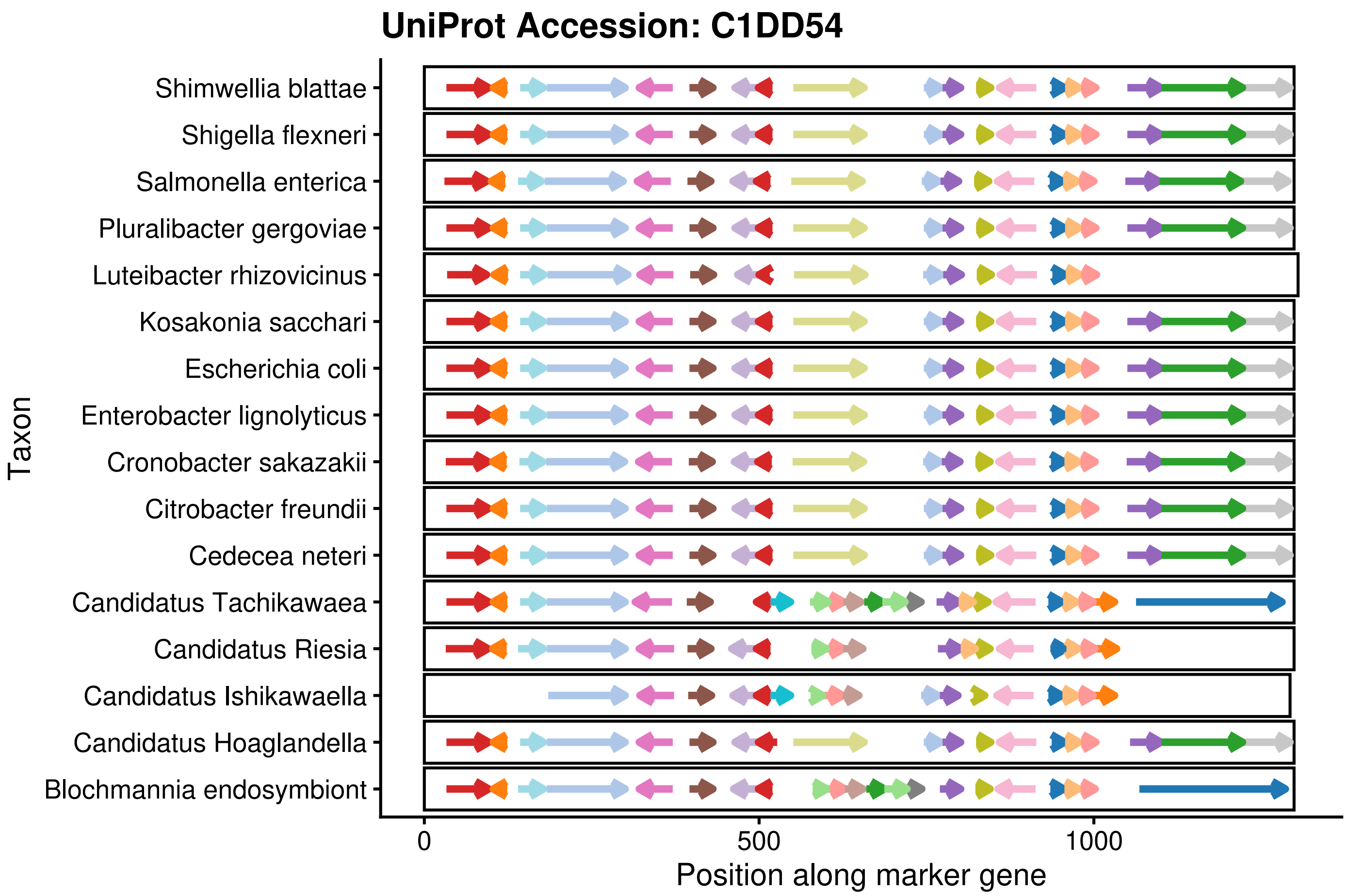
UniProt Accession: C1DD54
Taxon
Shimwellia blattae
Shigella flexneri
Salmonella enterica
Pluralibacter gergoviae
Luteibacter rhizovicinus
Kosakonia sacchari
Escherichia coli
Enterobacter lignolyticus
Cronobacter sakazakii
Citrobacter freundii
Cedecea neteri
Candidatus Tachikawaea
Candidatus Riesia
Candidatus Ishikawaella
Candidatus Hoaglandella
Blochmannia endosymbiont
0
500
1000
Position along marker gene

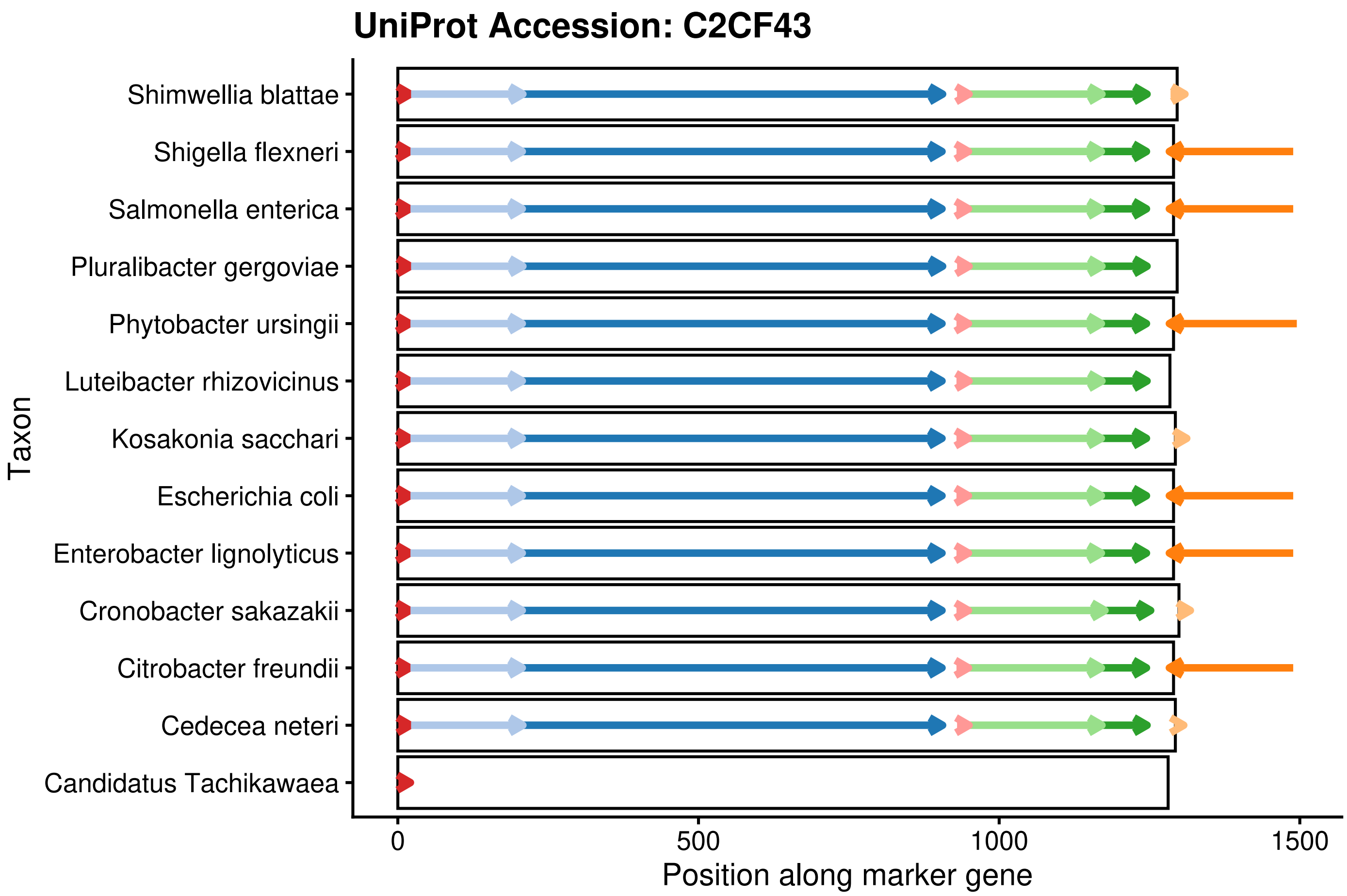
UniProt Accession: C2CF43
Taxon
Shimwellia blattae
Shigella flexneri
Salmonella enterica
Pluralibacter gergoviae
Phytobacter ursingii
Luteibacter rhizovicinus
Kosakonia sacchari
Escherichia coli
Enterobacter lignolyticus
Cronobacter sakazakii
Citrobacter freundii
Cedecea neteri
Candidatus Tachikawaea
0
500
1000
1500
Position along marker gene

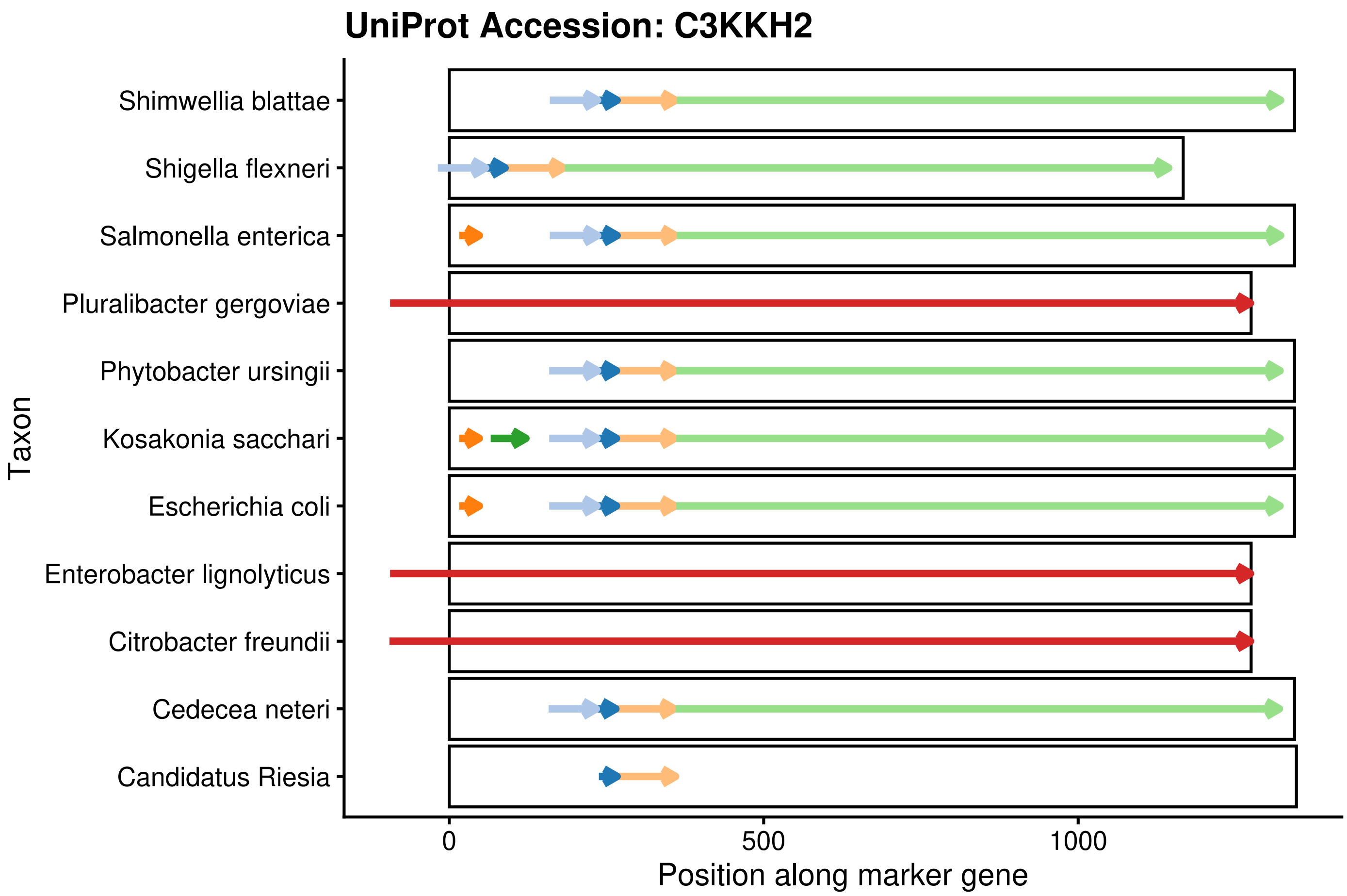
UniProt Accession: C3KKH2
Shimwellia blattae
Shigella flexneri
Salmonella enterica
Pluralibacter gergoviae
Phytobacter ursingii
Kosakonia sacchari
Escherichia coli
Enterobacter lignolyticus
Citrobacter freundii
Cedecea neteri
Candidatus Riesia
Taxon
0
500
1000
Position along marker gene

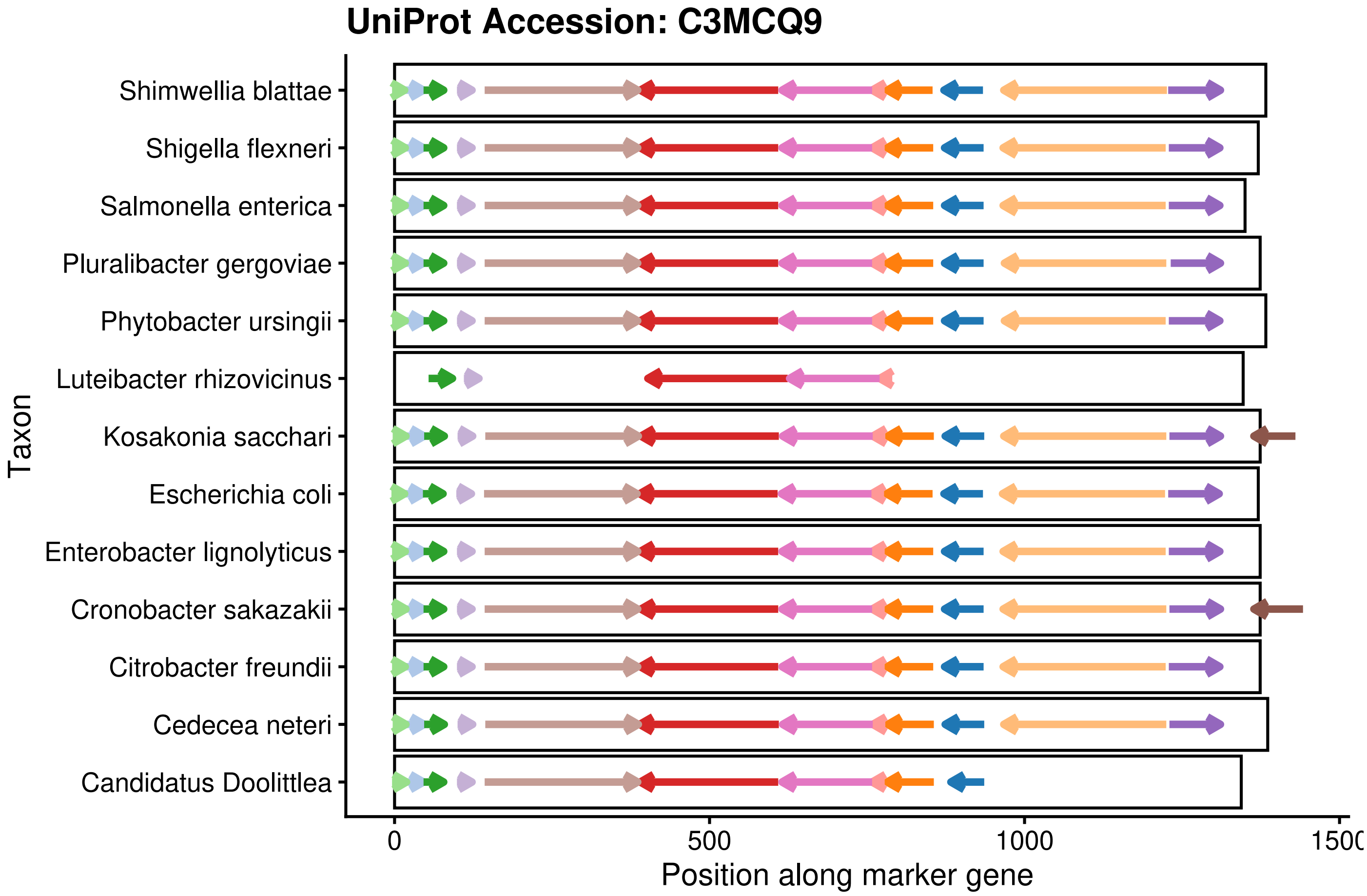

UniProt Accession: C3MCQ9
Taxon
Shimwellia blattae
Shigella flexneri
Salmonella enterica
Pluralibacter gergoviae
Phytobacter ursingii
Luteibacter rhizovicinus
Kosakonia sacchari
Escherichia coli
Enterobacter lignolyticus
Cronobacter sakazakii
Citrobacter freundii
Cedecea neteri
Candidatus Doolittlea
0
500
1000
Position along marker gene

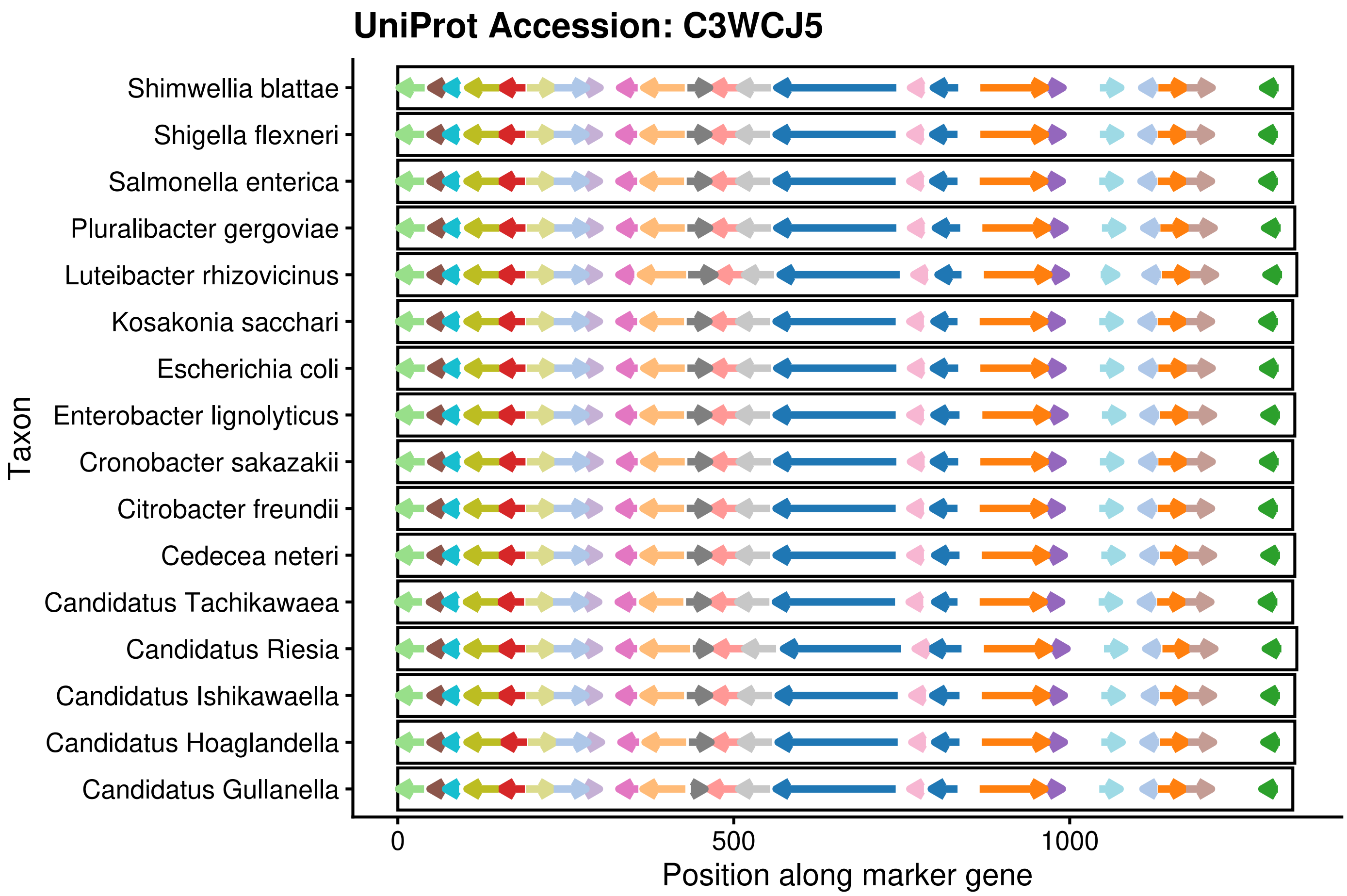
UniProt Accession: C3WCJ5
Taxon
Shimwellia blattae
Shigella flexneri
Salmonella enterica
Pluralibacter gergoviae
Luteibacter rhizovicinus
Kosakonia sacchari
Escherichia coli
Enterobacter lignolyticus
Cronobacter sakazakii
Citrobacter freundii
Cedecea neteri
Candidatus Tachikawaea
Candidatus Riesia
Candidatus Ishikawaella
Candidatus Hoaglandella
Candidatus Gullanella
0
500
1000
Position along marker gene

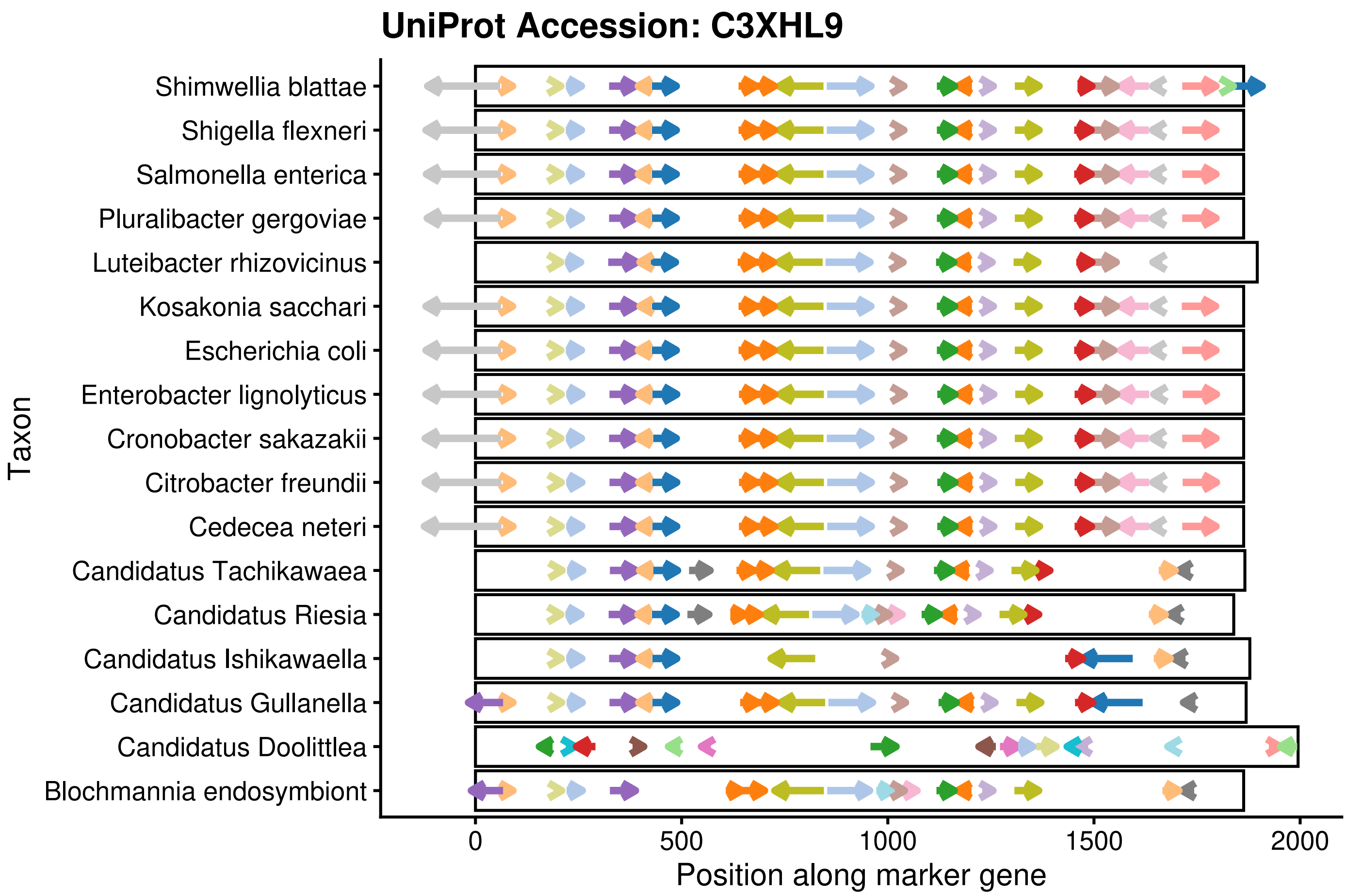

UniProt Accession: C3XHL9
Shimwellia blattae
Shigella flexneri
Salmonella enterica
Pluralibacter gergoviae
Luteibacter rhizovicinus
Kosakonia sacchari
Escherichia coli
Enterobacter lignolyticus
Cronobacter sakazakii
Citrobacter freundii
Cedecea neteri
Candidatus Tachikawaea
Candidatus Riesia
Candidatus Ishikawaella
Candidatus Gullanella
Candidatus Doolittlea
Blochmannia endosymbiont
Taxon
0
500
1000
1500
2000
Position along marker gene

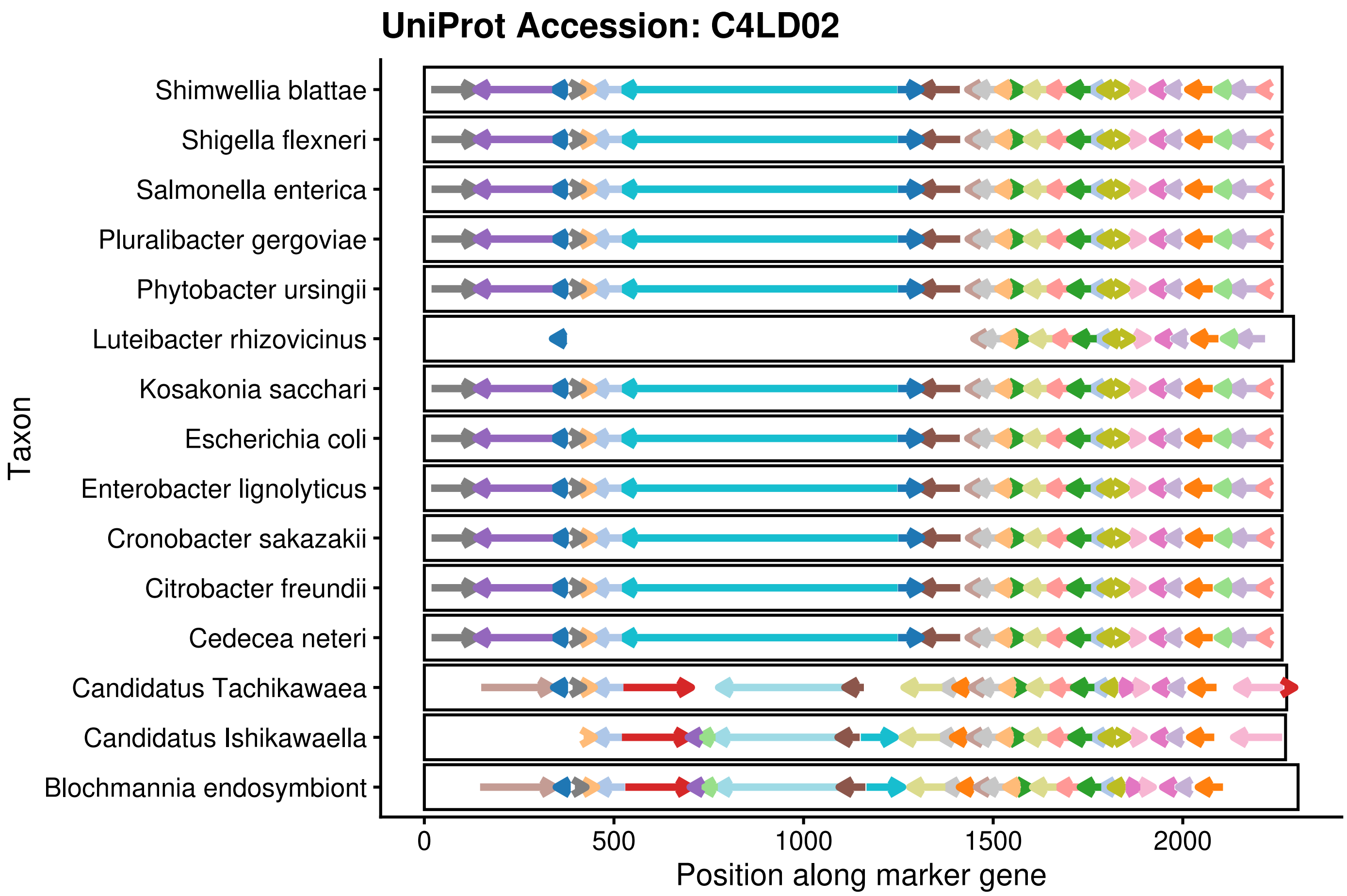
UniProt Accession: C4LD02
Taxon
Shimwellia blattae
Shigella flexneri
Salmonella enterica
Pluralibacter gergoviae
Phytobacter ursingii
Luteibacter rhizovicinus
Kosakonia sacchari
Escherichia coli
Enterobacter lignolyticus
Cronobacter sakazakii
Citrobacter freundii
Cedecea neteri
Candidatus Tachikawaea
Candidatus Ishikawaella
Blochmannia endosymbiont
0
500
1000
1500
2000
Position along marker gene

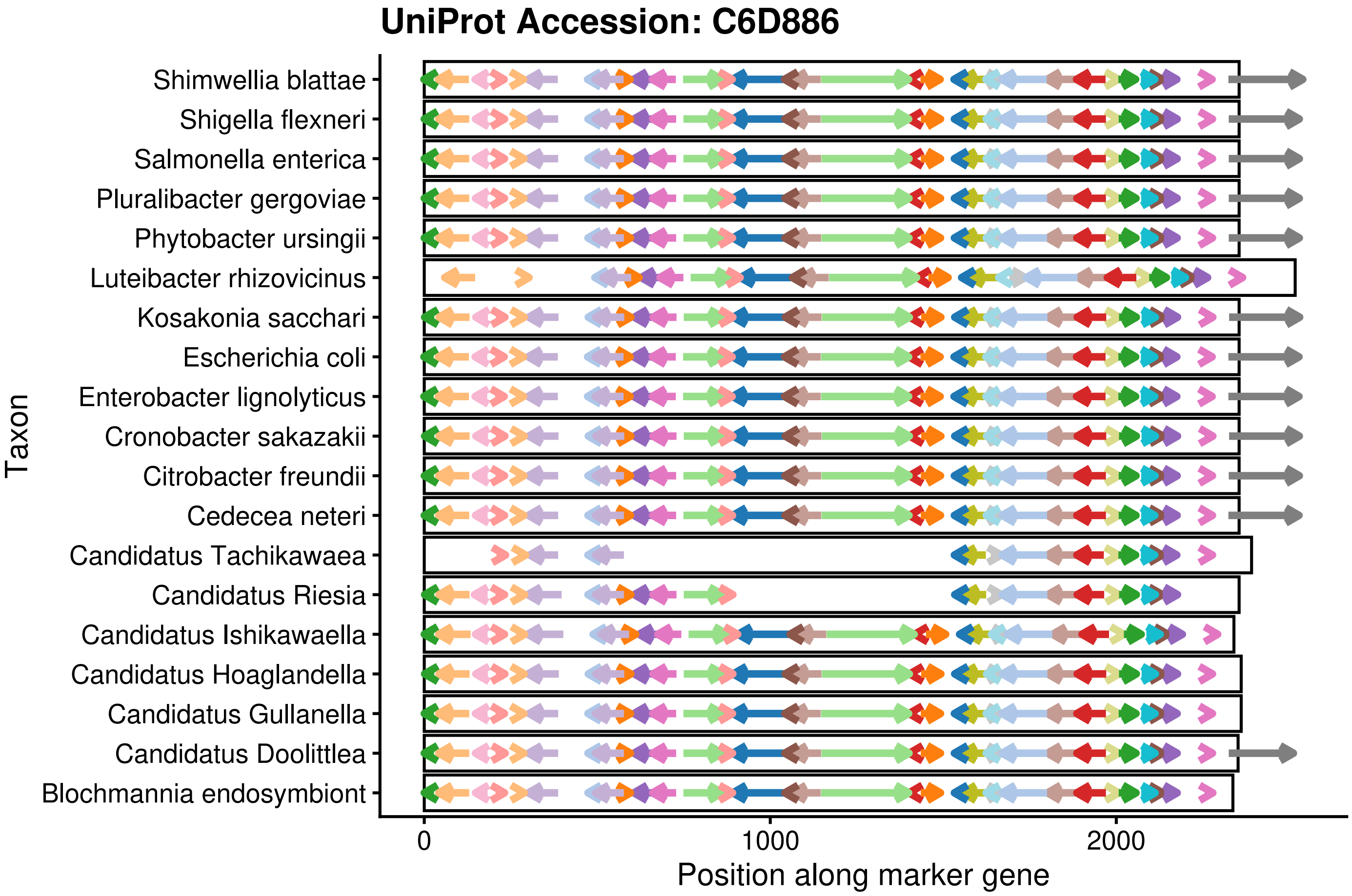
UniProt Accession: C6D886
Shimwellia blattae
Shigella flexneri
Salmonella enterica
Pluralibacter gergoviae
Phytobacter ursingii
Luteibacter rhizovicinus
Kosakonia sacchari
Escherichia coli
Enterobacter lignolyticus
Cronobacter sakazakii
Citrobacter freundii
Cedecea neteri
Candidatus Tachikawaea
Candidatus Riesia
Candidatus Ishikawaella
Candidatus Hoaglandella
Candidatus Gullanella
Candidatus Doolittlea
Blochmannia endosymbiont
Taxon
0
1000
2000
Position along marker gene

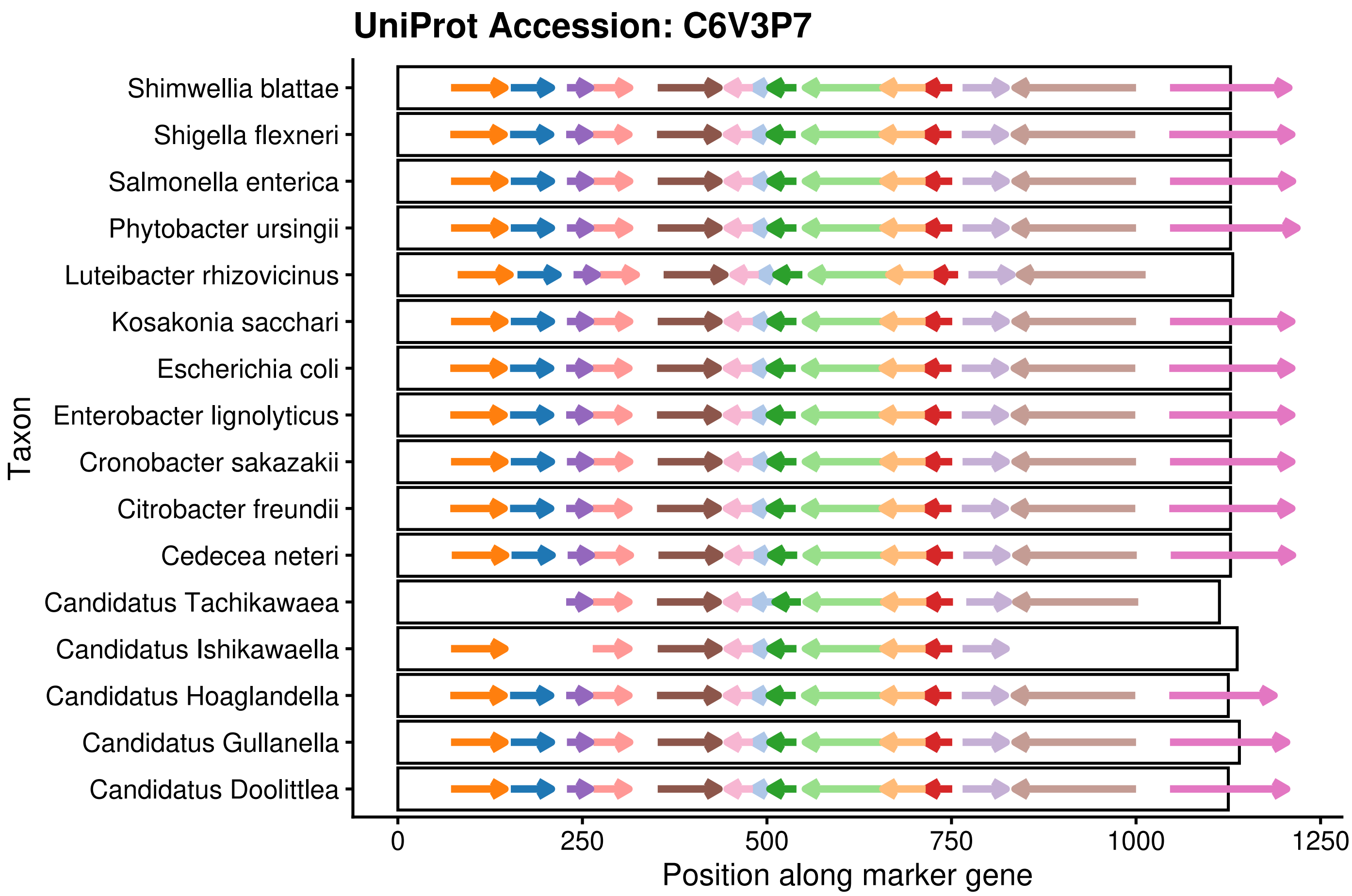
UniProt Accession: C6V3P7
Shimwellia blattae
Shigella flexneri
Salmonella enterica
Phytobacter ursingii
Luteibacter rhizovicinus
Kosakonia sacchari
Escherichia coli
Enterobacter lignolyticus
Cronobacter sakazakii
Citrobacter freundii
Cedecea neteri
Candidatus Tachikawaea
Candidatus Ishikawaella
Candidatus Hoaglandella
Candidatus Gullanella
Candidatus Doolittlea
Taxon
0
250
500
750
1000
1250
Position along marker gene

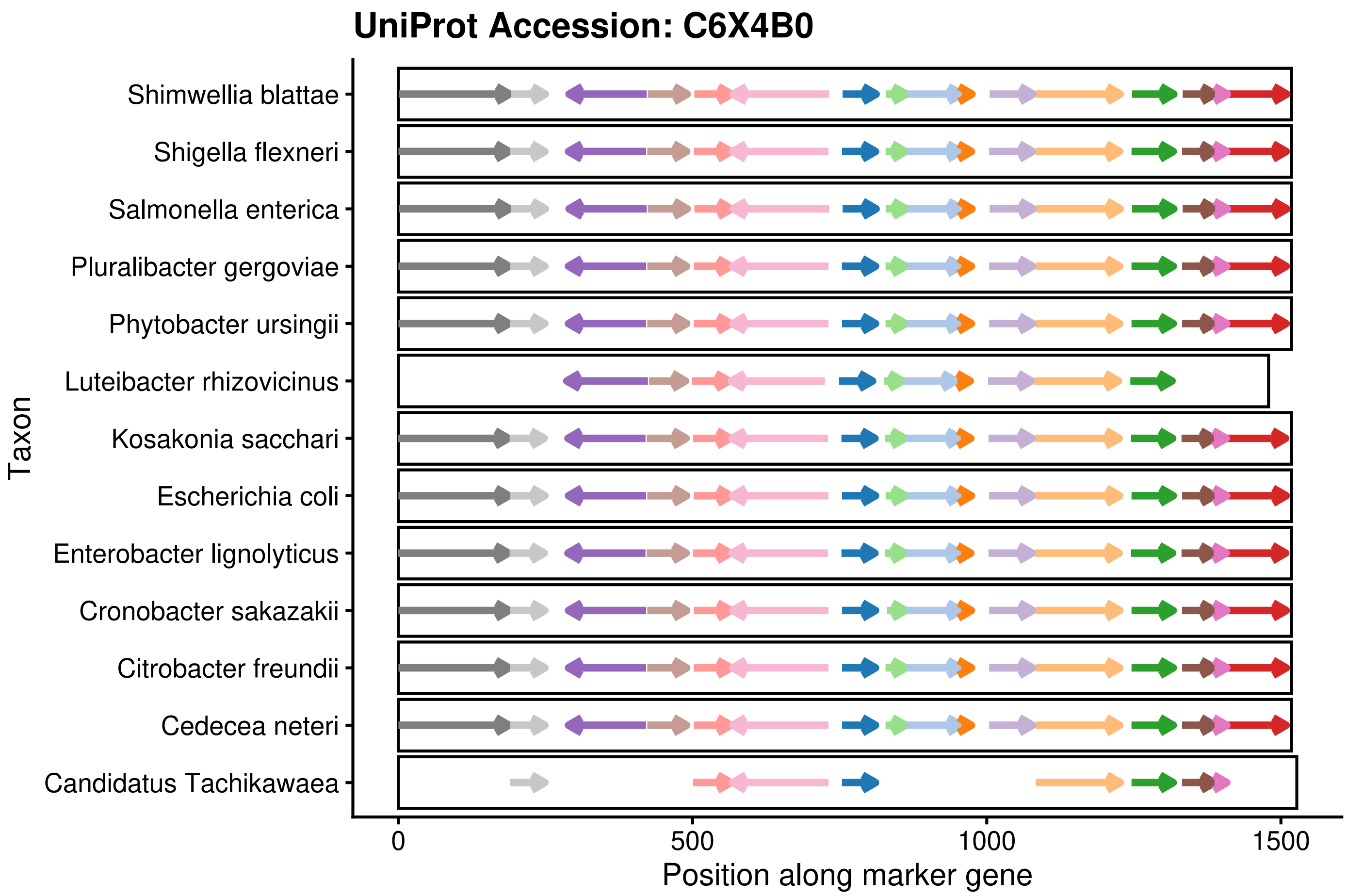

UniProt Accession: C6X4B0
Shimwellia blattae
Shigella flexneri
Salmonella enterica
Pluralibacter gergoviae
Phytobacter ursingii
Luteibacter rhizovicinus
Kosakonia sacchari
Escherichia coli
Enterobacter lignolyticus
Cronobacter sakazakii
Citrobacter freundii
Cedecea neteri
Candidatus Tachikawaea
Taxon
0
500
1000
1500
Position along marker gene

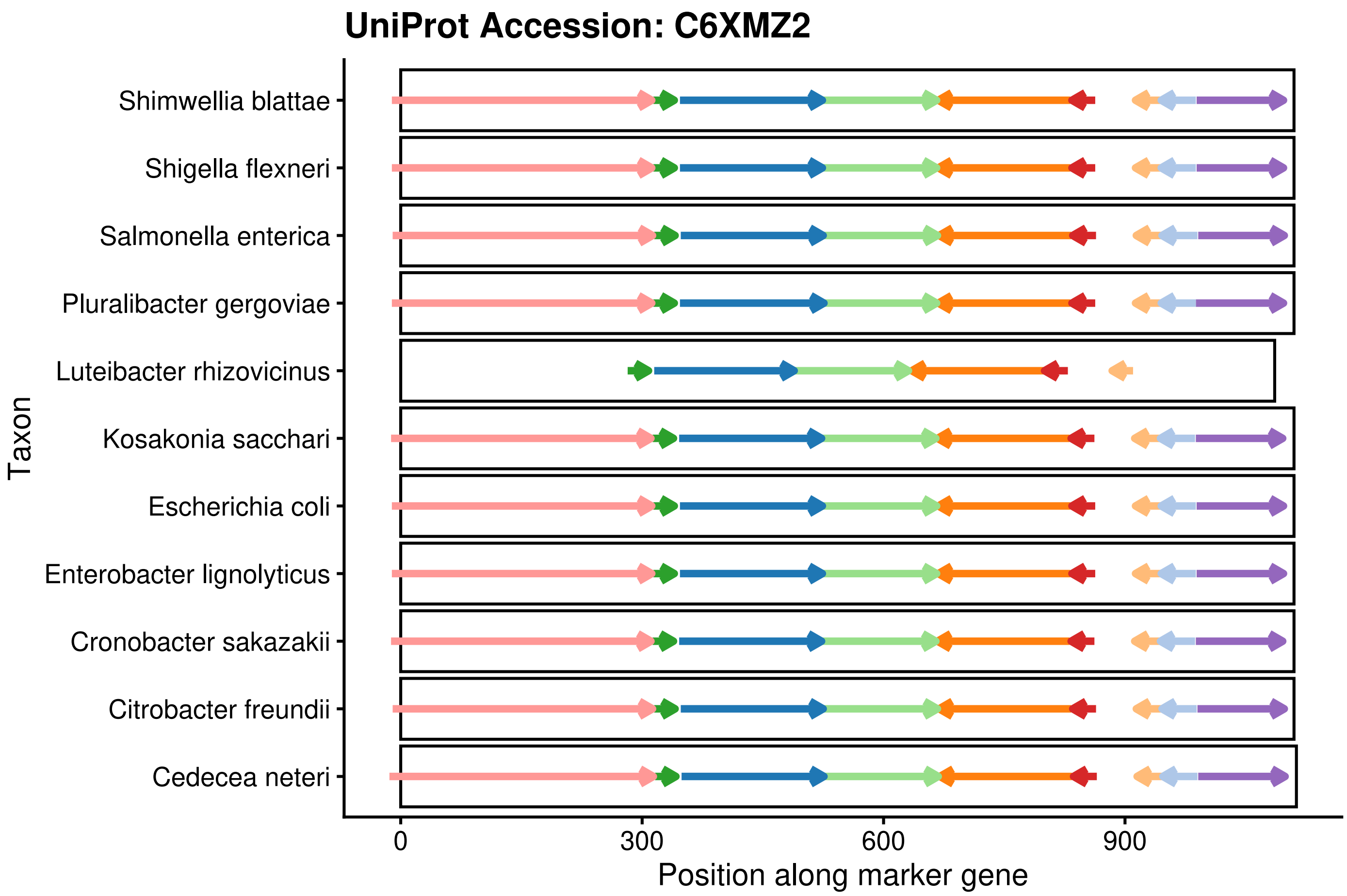
UniProt Accession: C6XMZ2
Taxon
Shimwellia blattae
Shigella flexneri
Salmonella enterica
Pluralibacter gergoviae
Luteibacter rhizovicinus
Kosakonia sacchari
Escherichia coli
Enterobacter lignolyticus
Cronobacter sakazakii
Citrobacter freundii
Cedecea neteri
0
300
600
900
Position along marker gene

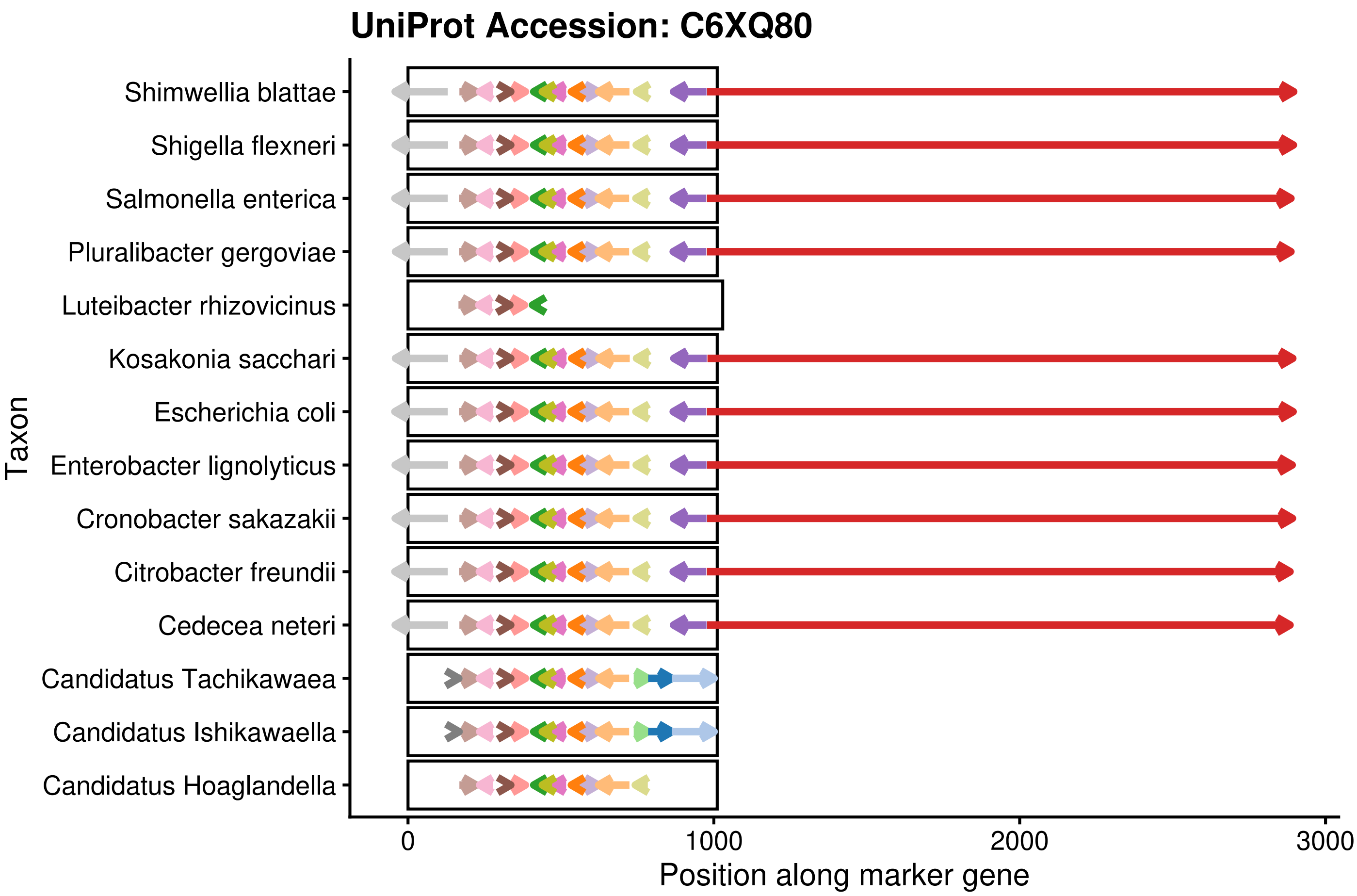

UniProt Accession: C6XQ80
Taxon
Shimwellia blattae
Shigella flexneri
Salmonella enterica
Pluralibacter gergoviae
Luteibacter rhizovicinus
Kosakonia sacchari
Escherichia coli
Enterobacter lignolyticus
Cronobacter sakazakii
Citrobacter freundii
Cedecea neteri
Candidatus Tachikawaea
Candidatus Ishikawaella
Candidatus Hoaglandella
0
1000
2000
3000
Position along marker gene

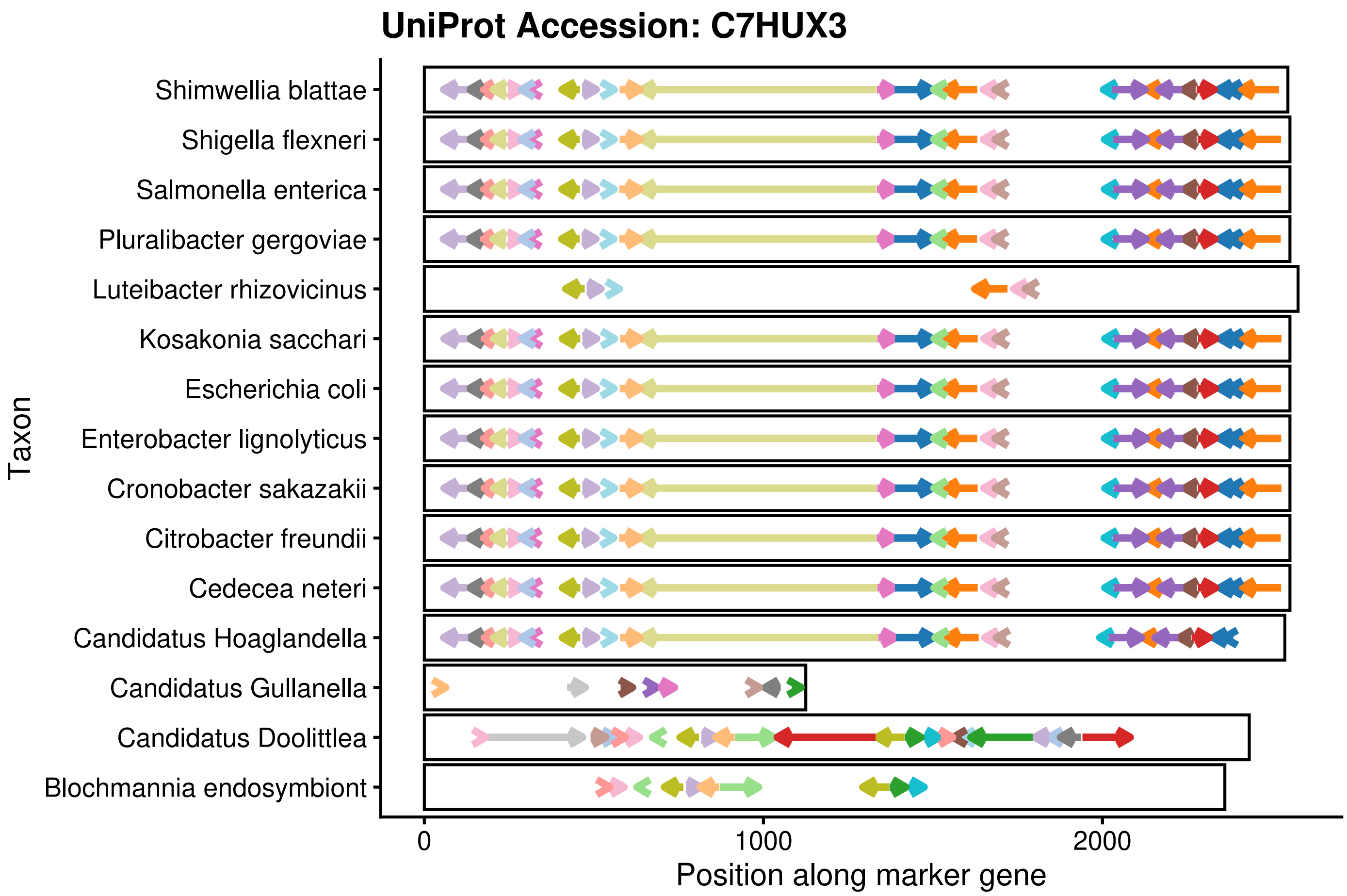
UniProt Accession: C7HUX3
Taxon
Shimwellia blattae
Shigella flexneri
Salmonella enterica
Pluralibacter gergoviae
Luteibacter rhizovicinus
Kosakonia sacchari
Escherichia coli
Enterobacter lignolyticus
Cronobacter sakazakii
Citrobacter freundii
Cedecea neteri
Candidatus Hoaglandella
Candidatus Gullanella
Candidatus Doolittlea
Blochmannia endosymbiont
0
1000
2000
Position along marker gene

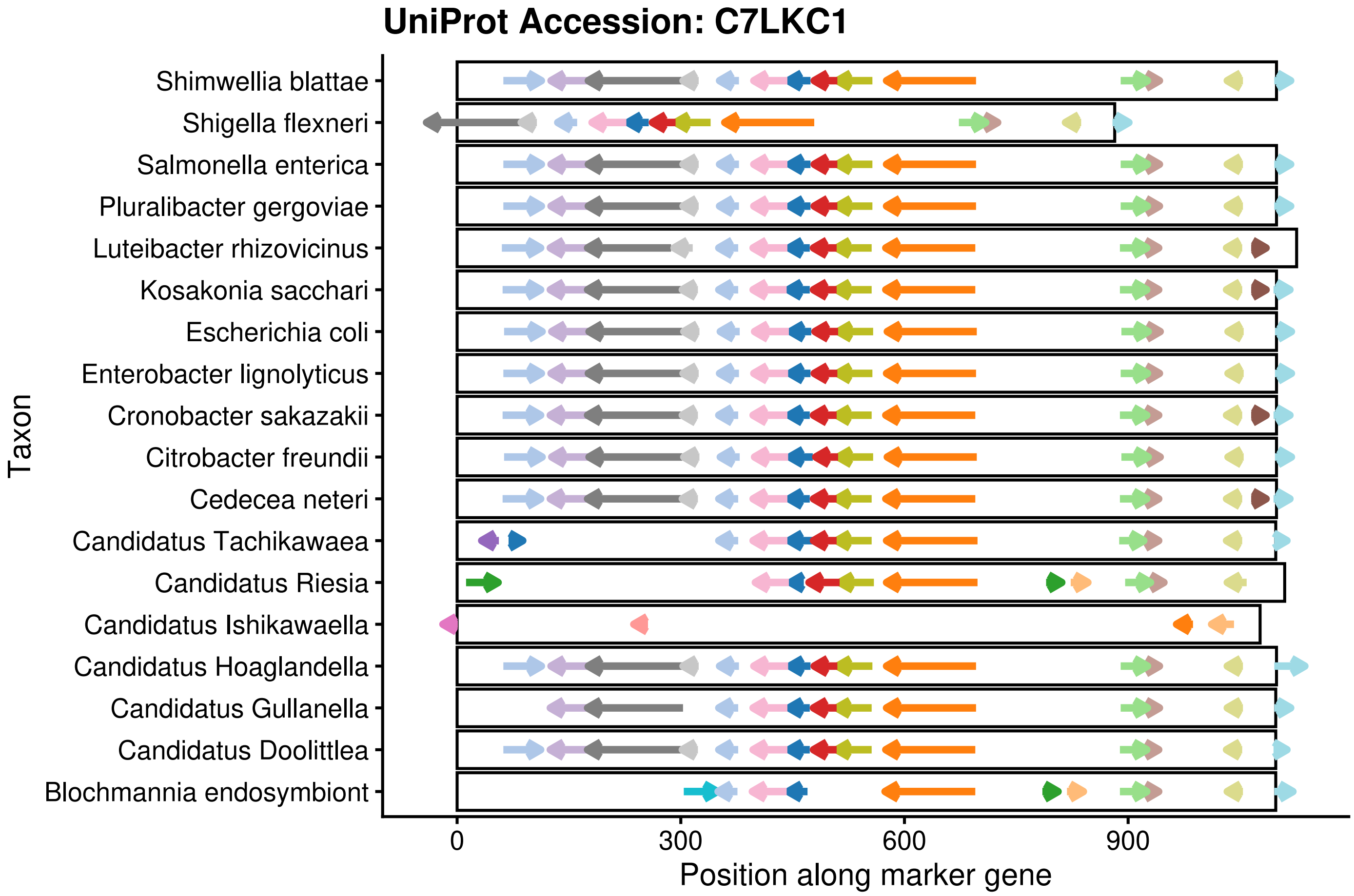
UniProt Accession: C7LKC1
Shimwellia blattae
Shigella flexneri
Salmonella enterica
Pluralibacter gergoviae
Luteibacter rhizovicinus
Kosakonia sacchari
Escherichia coli
Enterobacter lignolyticus
Cronobacter sakazakii
Citrobacter freundii
Cedecea neteri
Candidatus Tachikawaea
Candidatus Riesia
Candidatus Ishikawaella
Candidatus Hoaglandella
Candidatus Gullanella
Candidatus Doolittlea
Blochmannia endosymbiont
Taxon
0
300
600
900
Position along marker gene

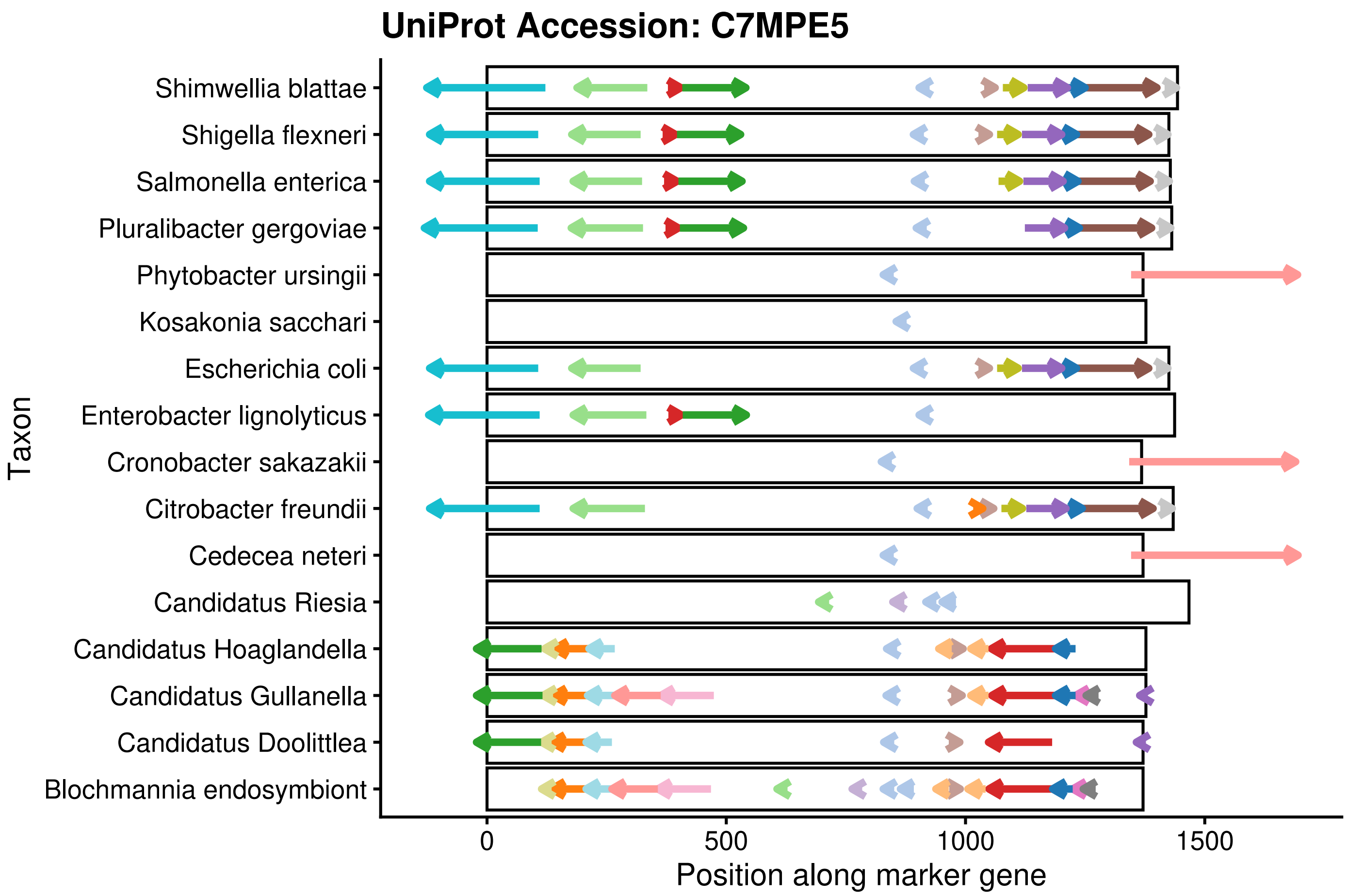
UniProt Accession: C7MPE5
Shimwellia blattae
Shigella flexneri
Salmonella enterica
Pluralibacter gergoviae
Phytobacter ursingii
Kosakonia sacchari
Escherichia coli
Enterobacter lignolyticus
Cronobacter sakazakii
Citrobacter freundii
Cedecea neteri
Candidatus Riesia
Candidatus Hoaglandella
Candidatus Gullanella
Candidatus Doolittlea
Blochmannia endosymbiont
Taxon
0
500
1000
1500
Position along marker gene

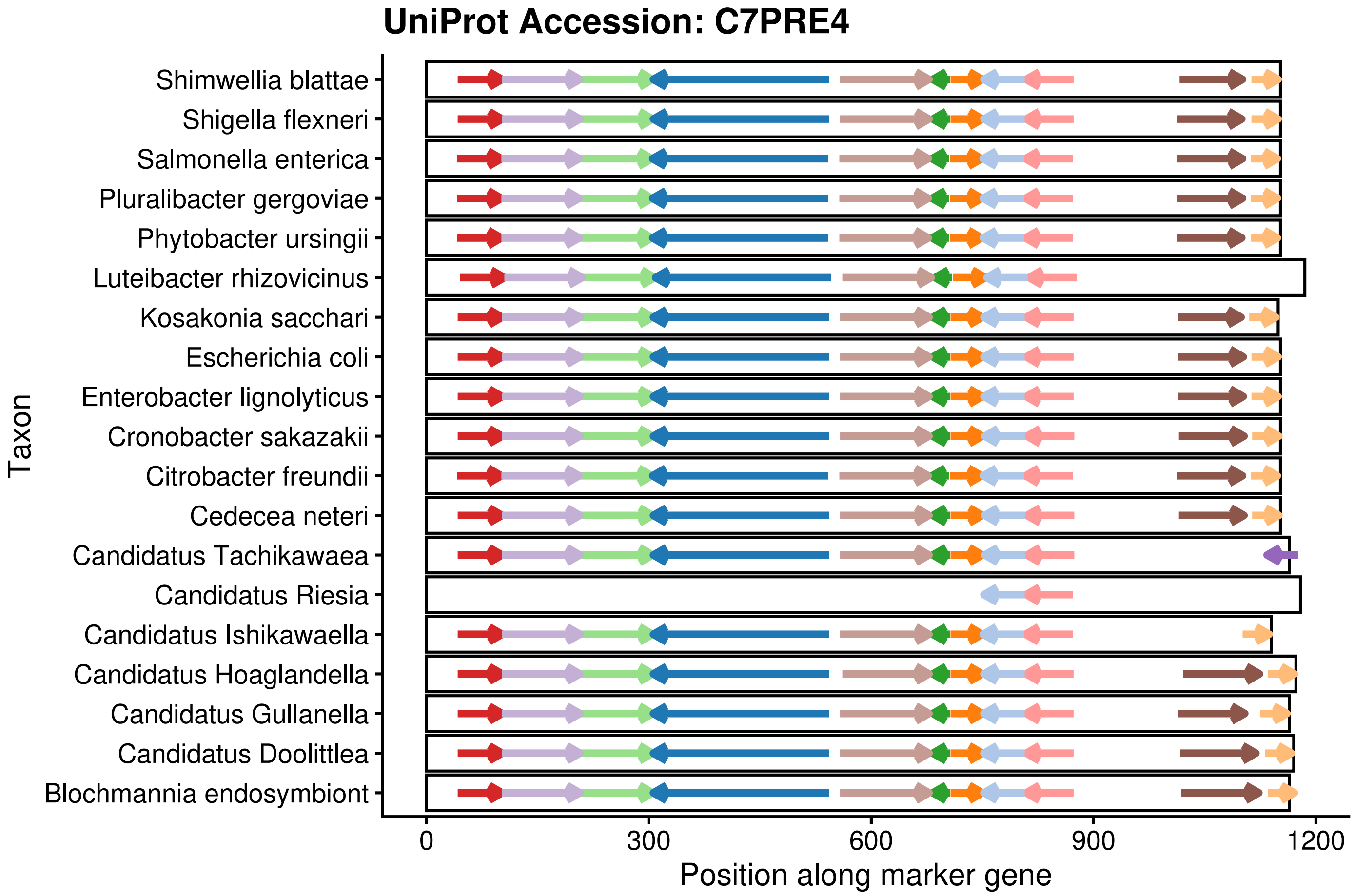
UniProt Accession: C7PRE4
Shimwellia blattae
Shigella flexneri
Salmonella enterica
Pluralibacter gergoviae
Phytobacter ursingii
Luteibacter rhizovicinus
Kosakonia sacchari
Escherichia coli
Enterobacter lignolyticus
Cronobacter sakazakii
Citrobacter freundii
Cedecea neteri
Candidatus Tachikawaea
Candidatus Riesia
Candidatus Ishikawaella
Candidatus Hoaglandella
Candidatus Gullanella
Candidatus Doolittlea
Blochmannia endosymbiont
Taxon
0
300
600
900
1200
Position along marker gene

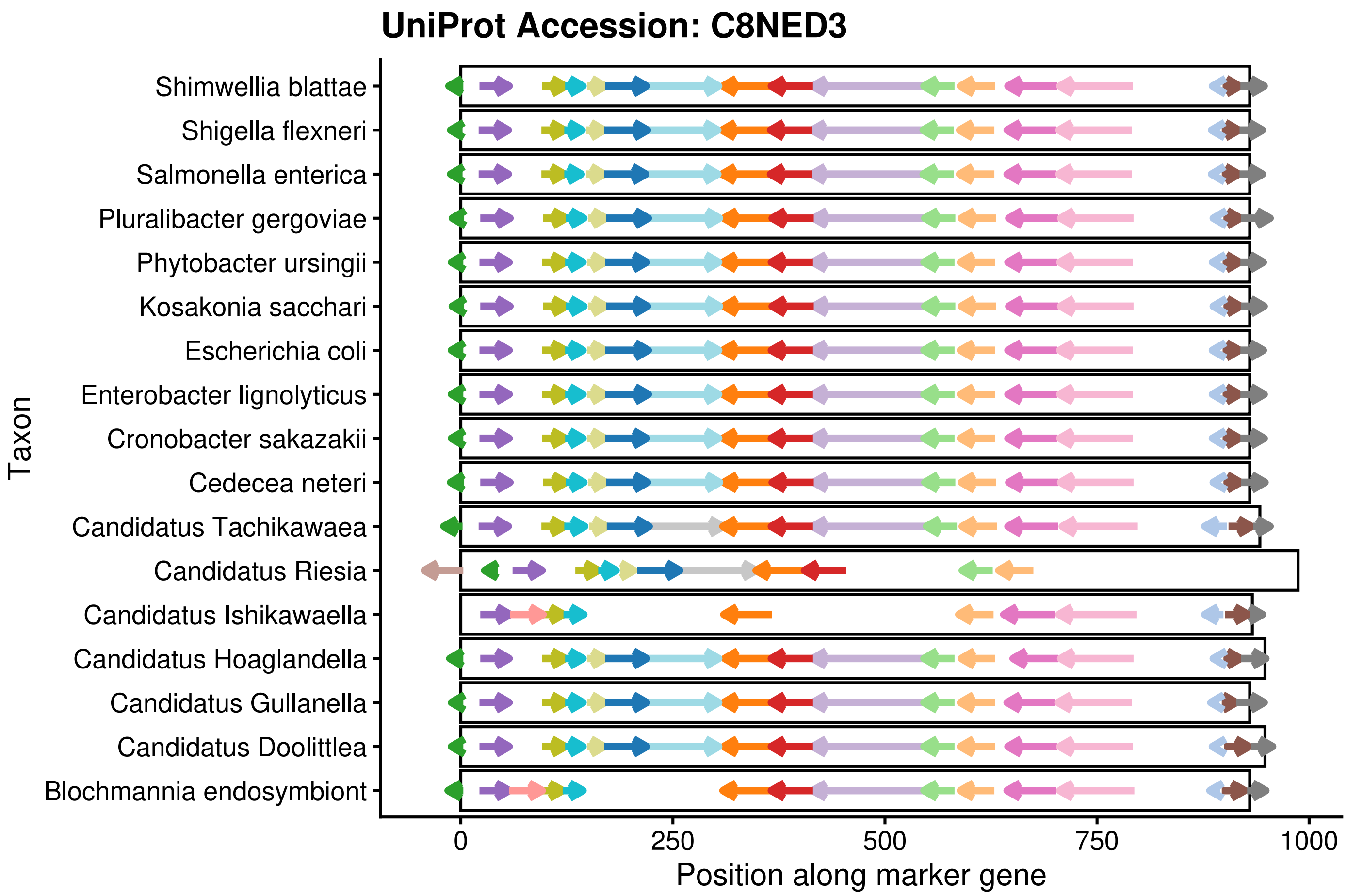

UniProt Accession: C8NED3
Taxon
Shimwellia blattae
Shigella flexneri
Salmonella enterica
Pluralibacter gergoviae
Phytobacter ursingii
Kosakonia sacchari
Escherichia coli
Enterobacter lignolyticus
Cronobacter sakazakii
Cedecea neteri
Candidatus Tachikawaea
Candidatus Riesia
Candidatus Ishikawaella
Candidatus Hoaglandella
Candidatus Gullanella
Candidatus Doolittlea
Blochmannia endosymbiont
0
250
500
750
1000
Position along marker gene

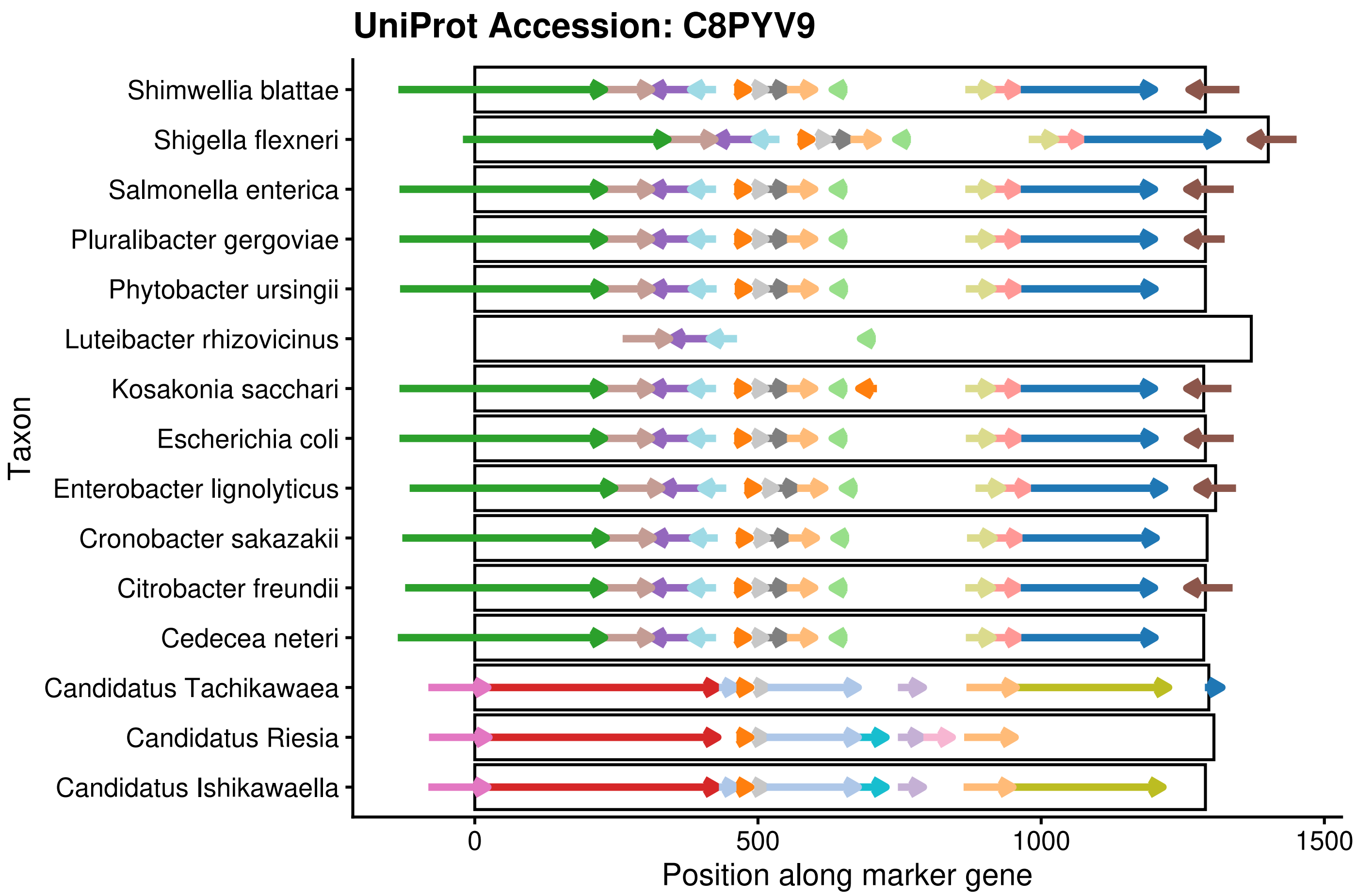
UniProt Accession: C8PYV9
Shimwellia blattae
Shigella flexneri
Salmonella enterica
Pluralibacter gergoviae
Phytobacter ursingii
Luteibacter rhizovicinus
Kosakonia sacchari
Escherichia coli
Enterobacter lignolyticus
Cronobacter sakazakii
Citrobacter freundii
Cedecea neteri
Candidatus Tachikawaea
Candidatus Riesia
Candidatus Ishikawaella
Taxon
0
500
1000
1500
Position along marker gene

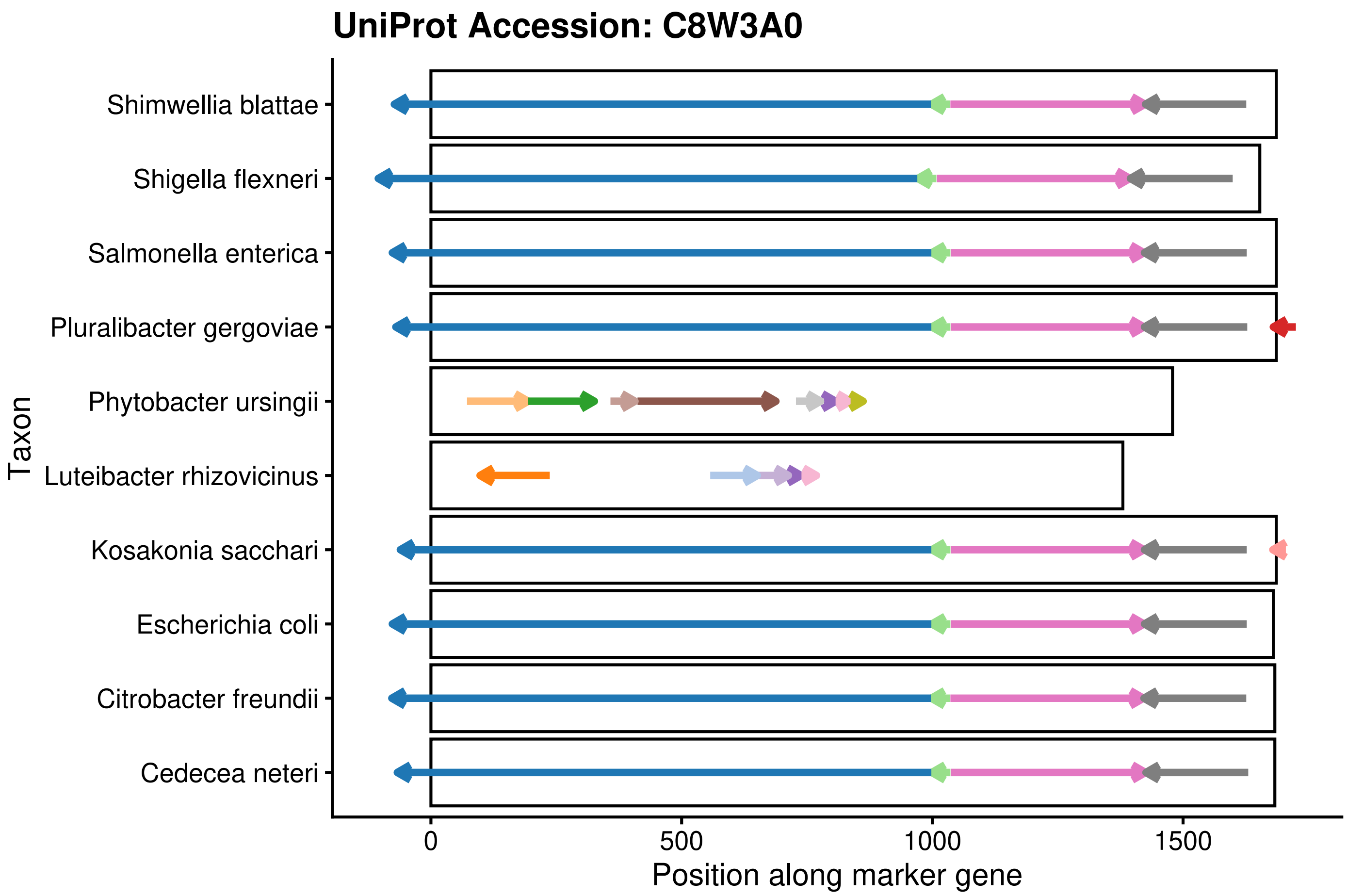

UniProt Accession: C8W3A0
Shimwellia blattae
Shigella flexneri
Salmonella enterica
Pluralibacter gergoviae
Phytobacter ursingii
Luteibacter rhizovicinus
Kosakonia sacchari
Escherichia coli
Citrobacter freundii
Cedecea neteri
Taxon
0
500
1000
1500
Position along marker gene

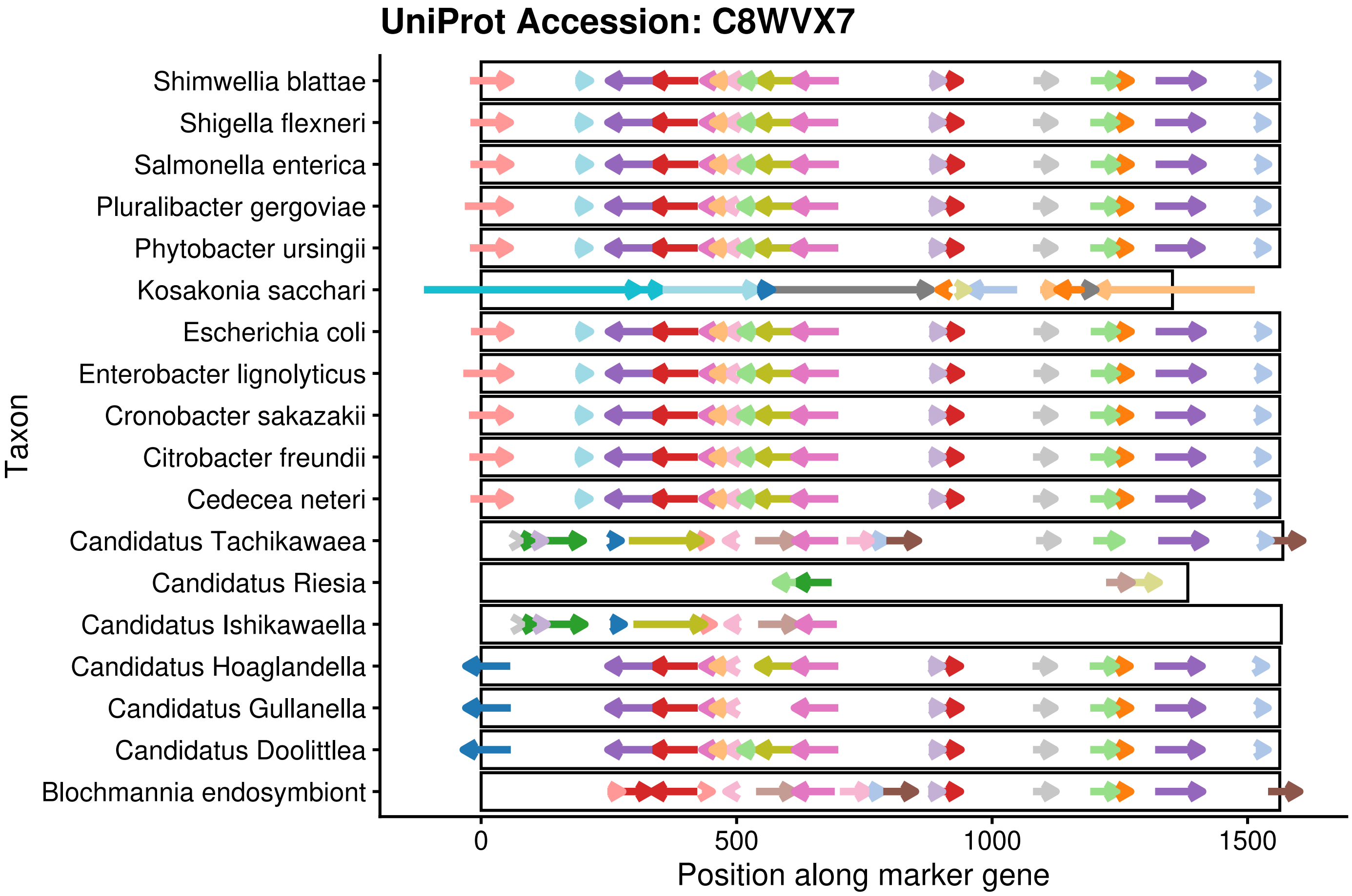
UniProt Accession: C8WVX7
Taxon
Shimwellia blattae
Shigella flexneri
Salmonella enterica
Pluralibacter gergoviae
Phytobacter ursingii
Kosakonia sacchari
Escherichia coli
Enterobacter lignolyticus
Cronobacter sakazakii
Citrobacter freundii
Cedecea neteri
Candidatus Tachikawaea
Candidatus Riesia
Candidatus Ishikawaella
Candidatus Hoaglandella
Candidatus Gullanella
Candidatus Doolittlea
Blochmannia endosymbiont
0
500
1000
1500
Position along marker gene

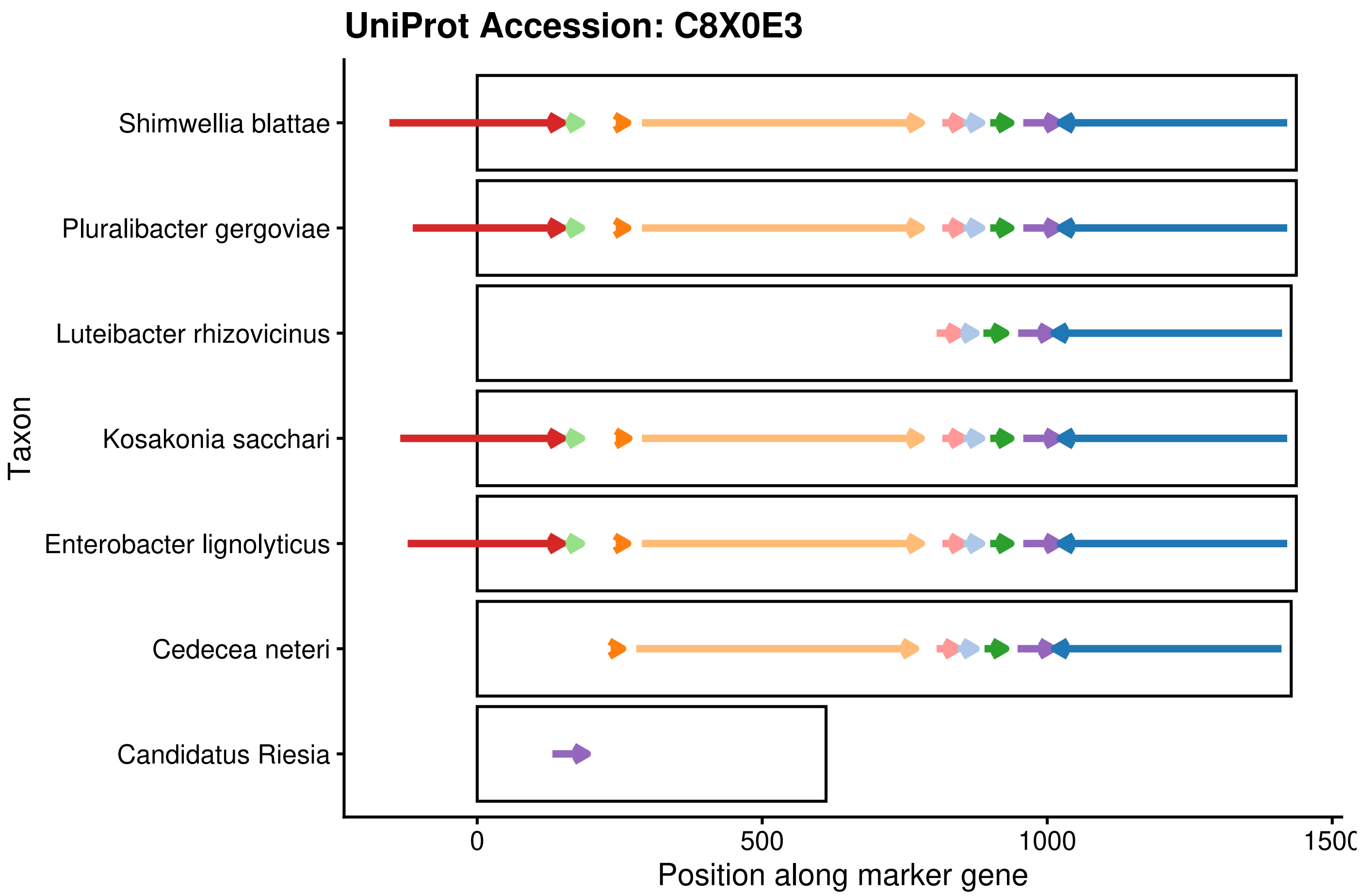
UniProt Accession: C8X0E3
Taxon
Shimwellia blattae
Pluralibacter gergoviae
Luteibacter rhizovicinus
Kosakonia sacchari
Enterobacter lignolyticus
Cedecea neteri
Candidatus Riesia
0
500
1000
1500
Position along marker gene

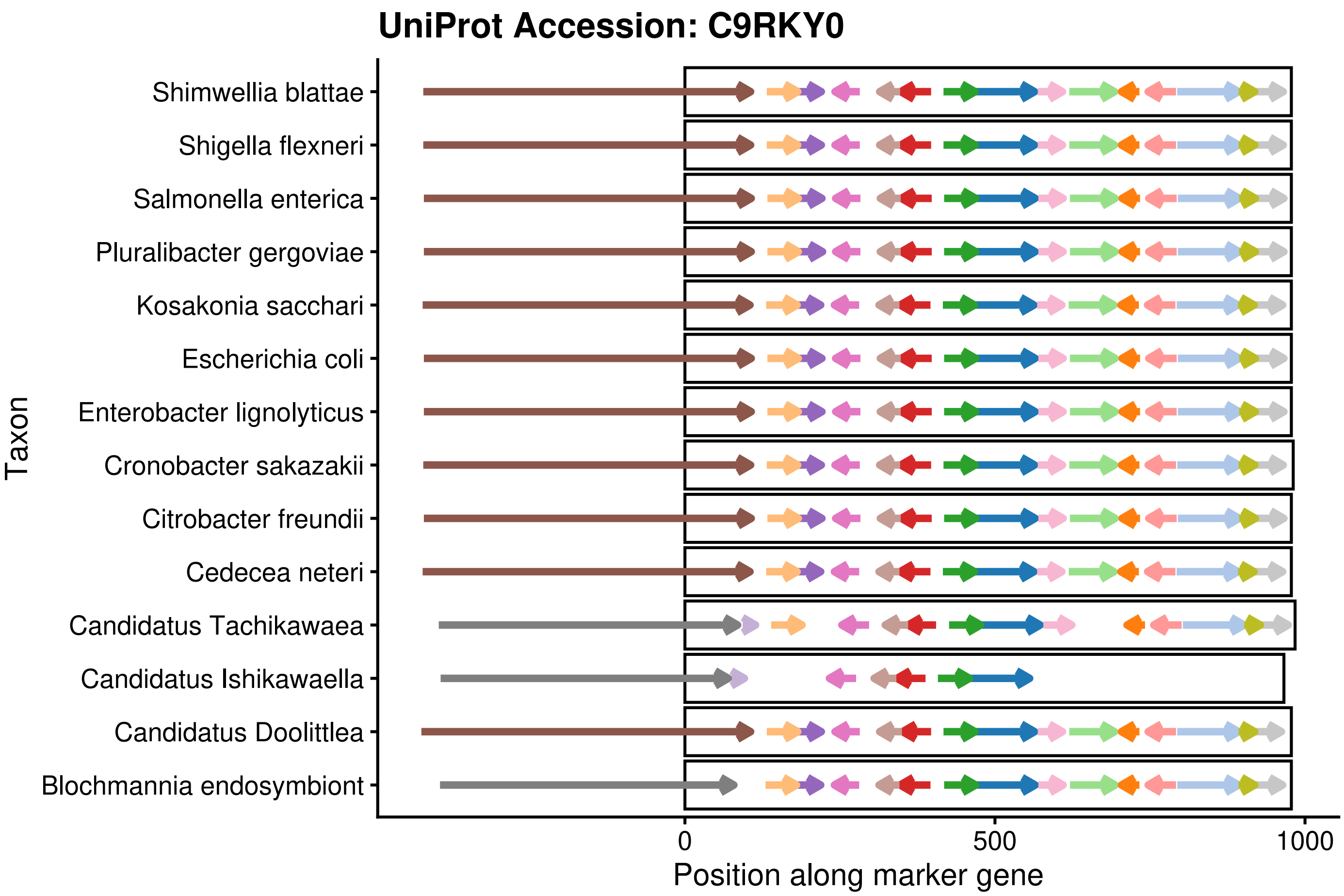

UniProt Accession: C9RKY0
Shimwellia blattae
Shigella flexneri
Salmonella enterica
Pluralibacter gergoviae
Kosakonia sacchari
Escherichia coli
Enterobacter lignolyticus
Cronobacter sakazakii
Citrobacter freundii
Cedecea neteri
Candidatus Tachikawaea
Candidatus Ishikawaella
Candidatus Doolittlea
Blochmannia endosymbiont
Taxon
0
500
1000
Position along marker gene

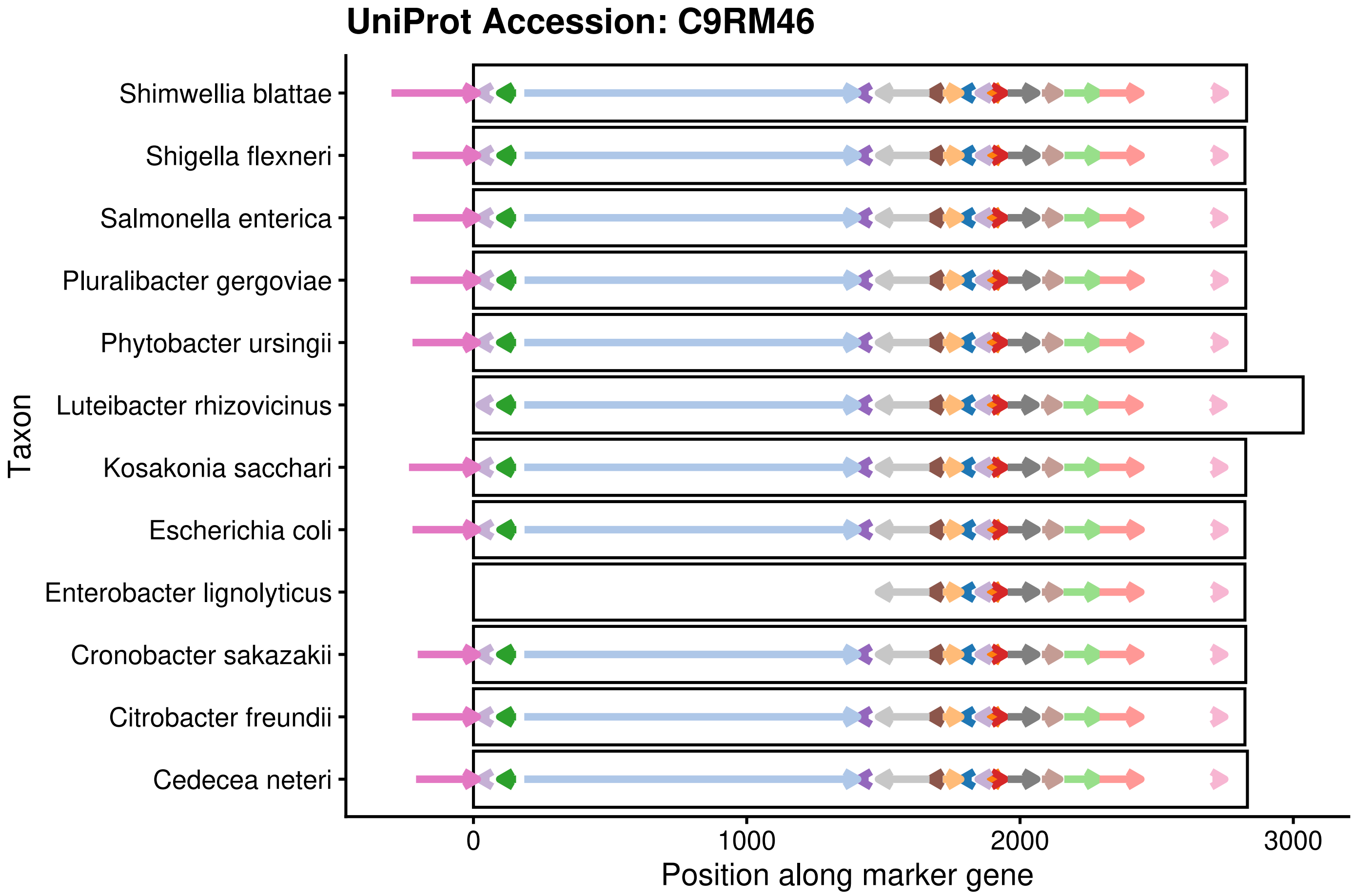

UniProt Accession: C9RM46
Taxon
Shimwellia blattae
Shigella flexneri
Salmonella enterica
Pluralibacter gergoviae
Phytobacter ursingii
Luteibacter rhizovicinus
Kosakonia sacchari
Escherichia coli
Enterobacter lignolyticus
Cronobacter sakazakii
Citrobacter freundii
Cedecea neteri
0
1000
2000
3000
Position along marker gene

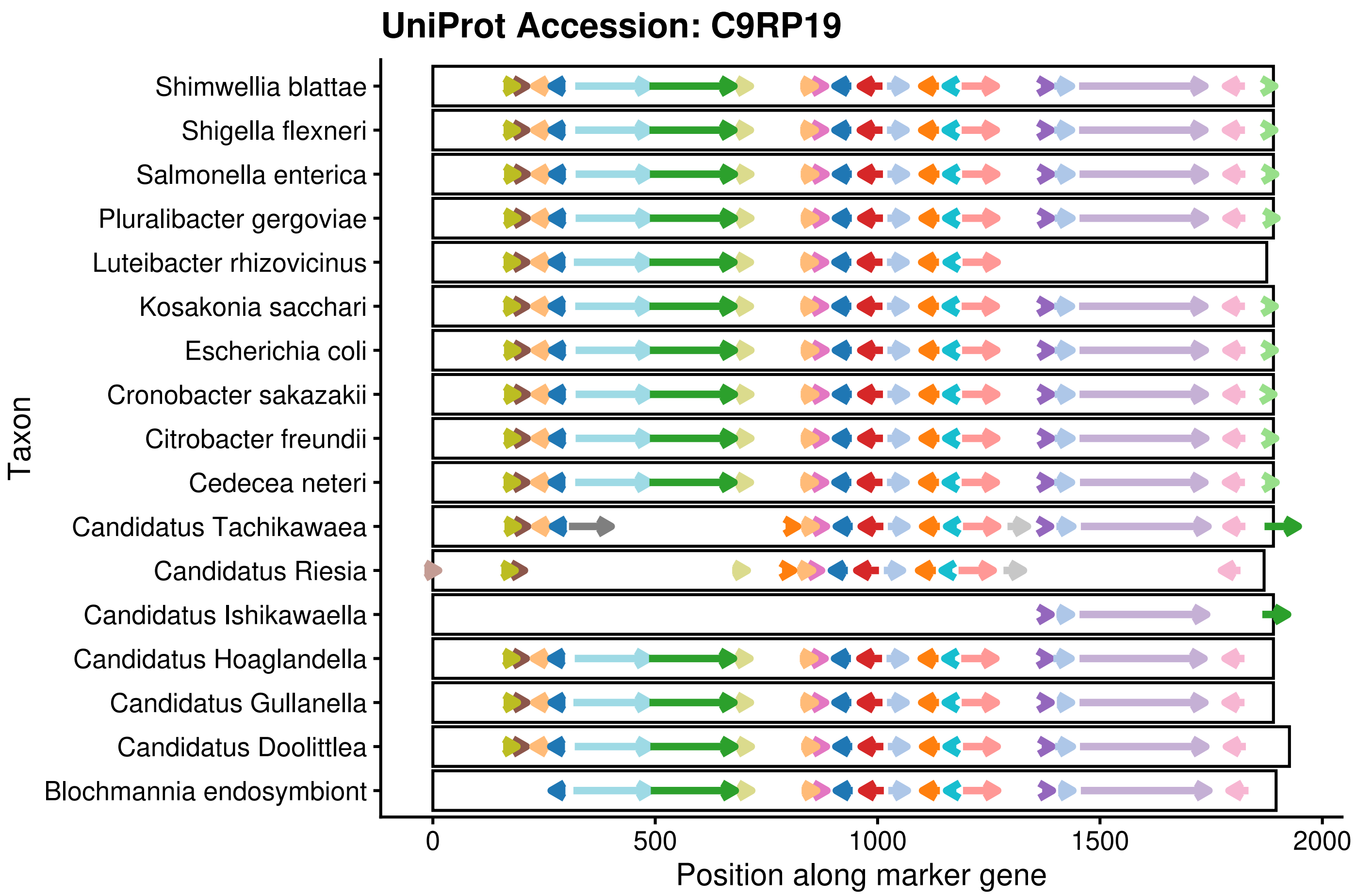
UniProt Accession: C9RP19
Taxon
Shimwellia blattae
Shigella flexneri
Salmonella enterica
Pluralibacter gergoviae
Luteibacter rhizovicinus
Kosakonia sacchari
Escherichia coli
Cronobacter sakazakii
Citrobacter freundii
Cedecea neteri
Candidatus Tachikawaea
Candidatus Riesia
Candidatus Ishikawaella
Candidatus Hoaglandella
Candidatus Gullanella
Candidatus Doolittlea
Blochmannia endosymbiont
0
500
1000
1500
2000
Position along marker gene

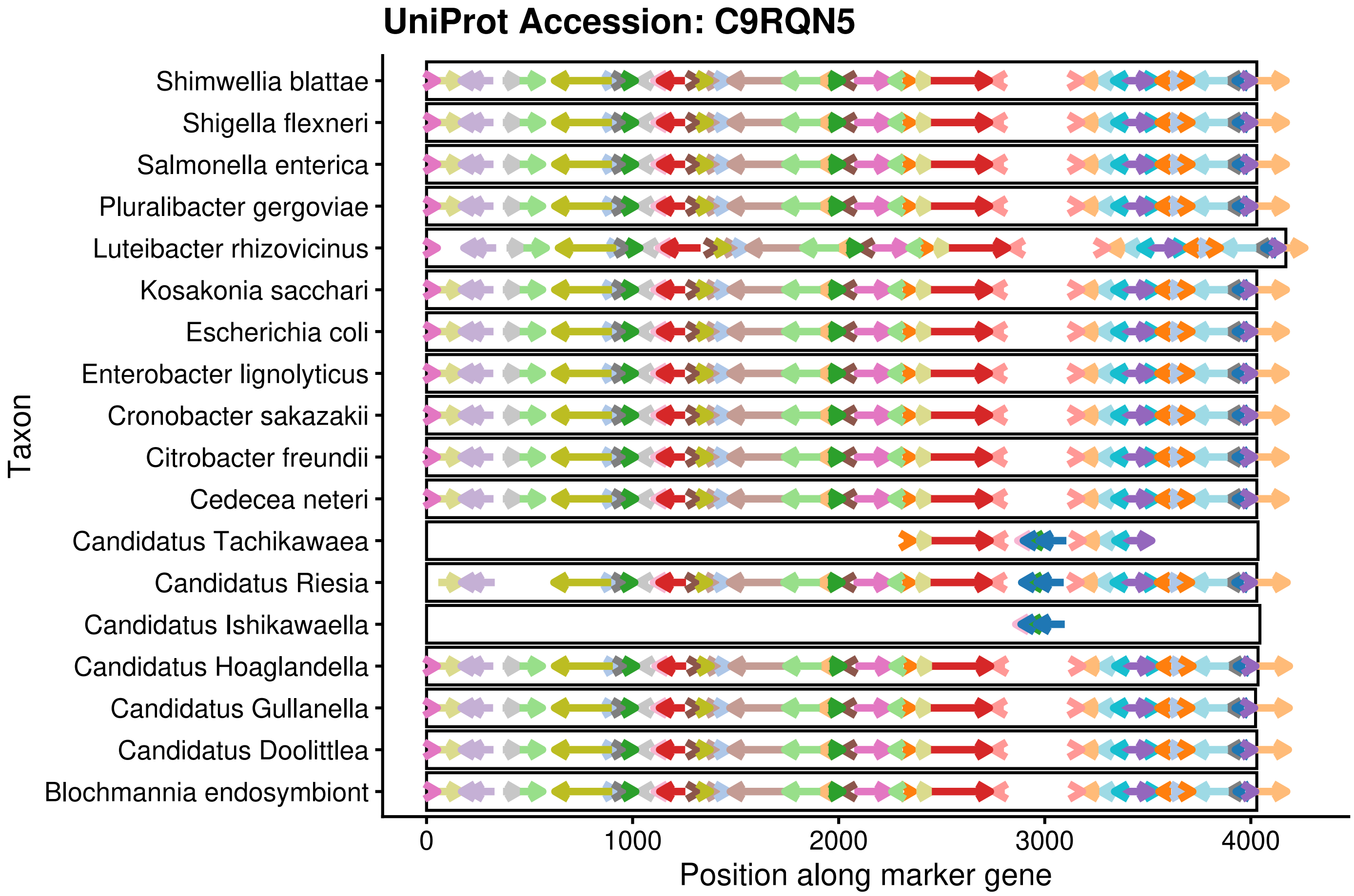
UniProt Accession: C9RQN5
Taxon
Shimwellia blattae
Shigella flexneri
Salmonella enterica
Pluralibacter gergoviae
Luteibacter rhizovicinus
Kosakonia sacchari
Escherichia coli
Enterobacter lignolyticus
Cronobacter sakazakii
Citrobacter freundii
Cedecea neteri
Candidatus Tachikawaea
Candidatus Riesia
Candidatus Ishikawaella
Candidatus Hoaglandella
Candidatus Gullanella
Candidatus Doolittlea
Blochmannia endosymbiont
0
1000
2000
3000
4000
Position along marker gene

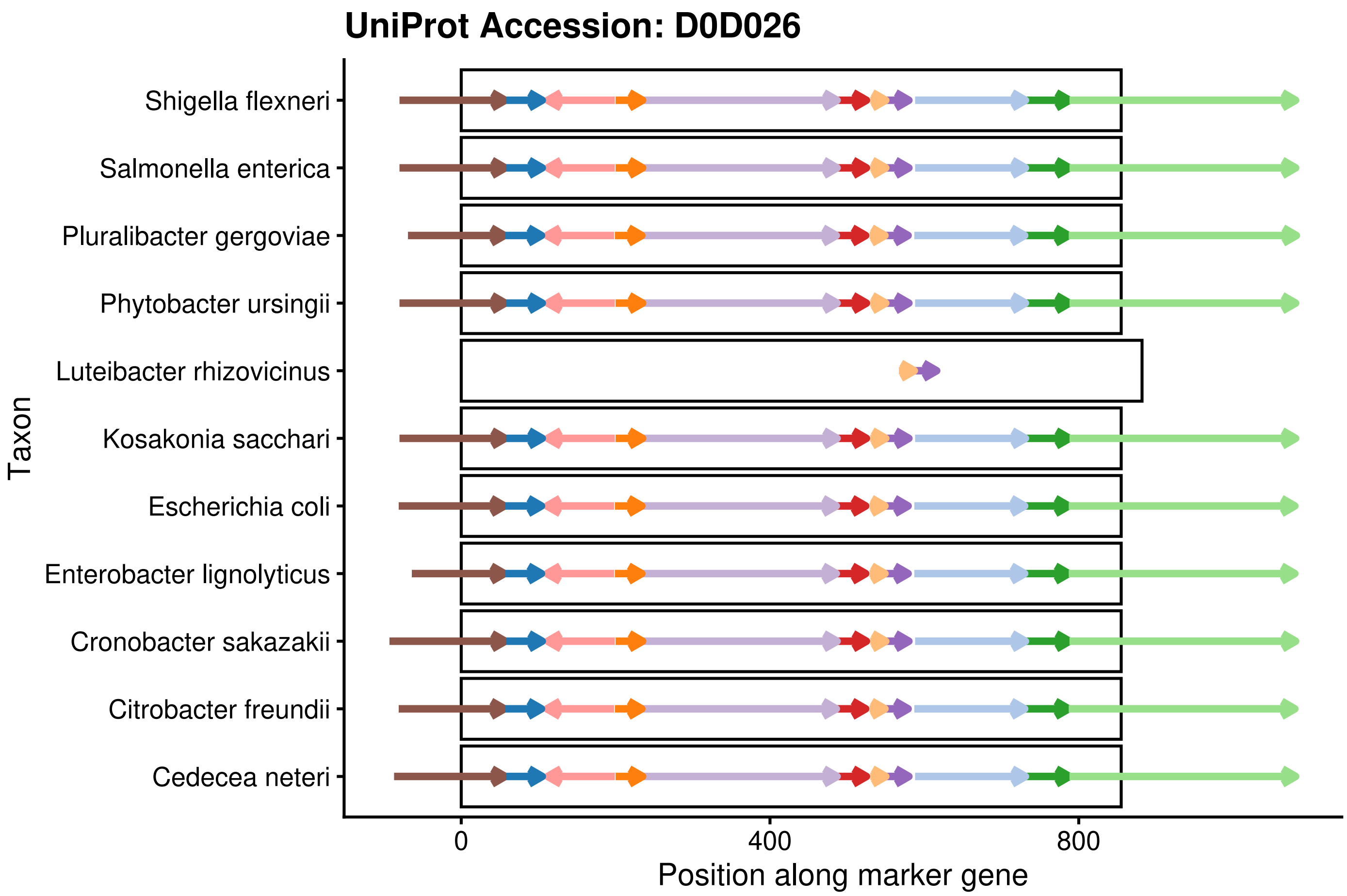

UniProt Accession: D0D026
Shigella flexneri
Salmonella enterica
Pluralibacter gergoviae
Phytobacter ursingii
Luteibacter rhizovicinus
Kosakonia sacchari
Escherichia coli
Enterobacter lignolyticus
Cronobacter sakazakii
Citrobacter freundii
Cedecea neteri
Taxon
0
400
800
Position along marker gene

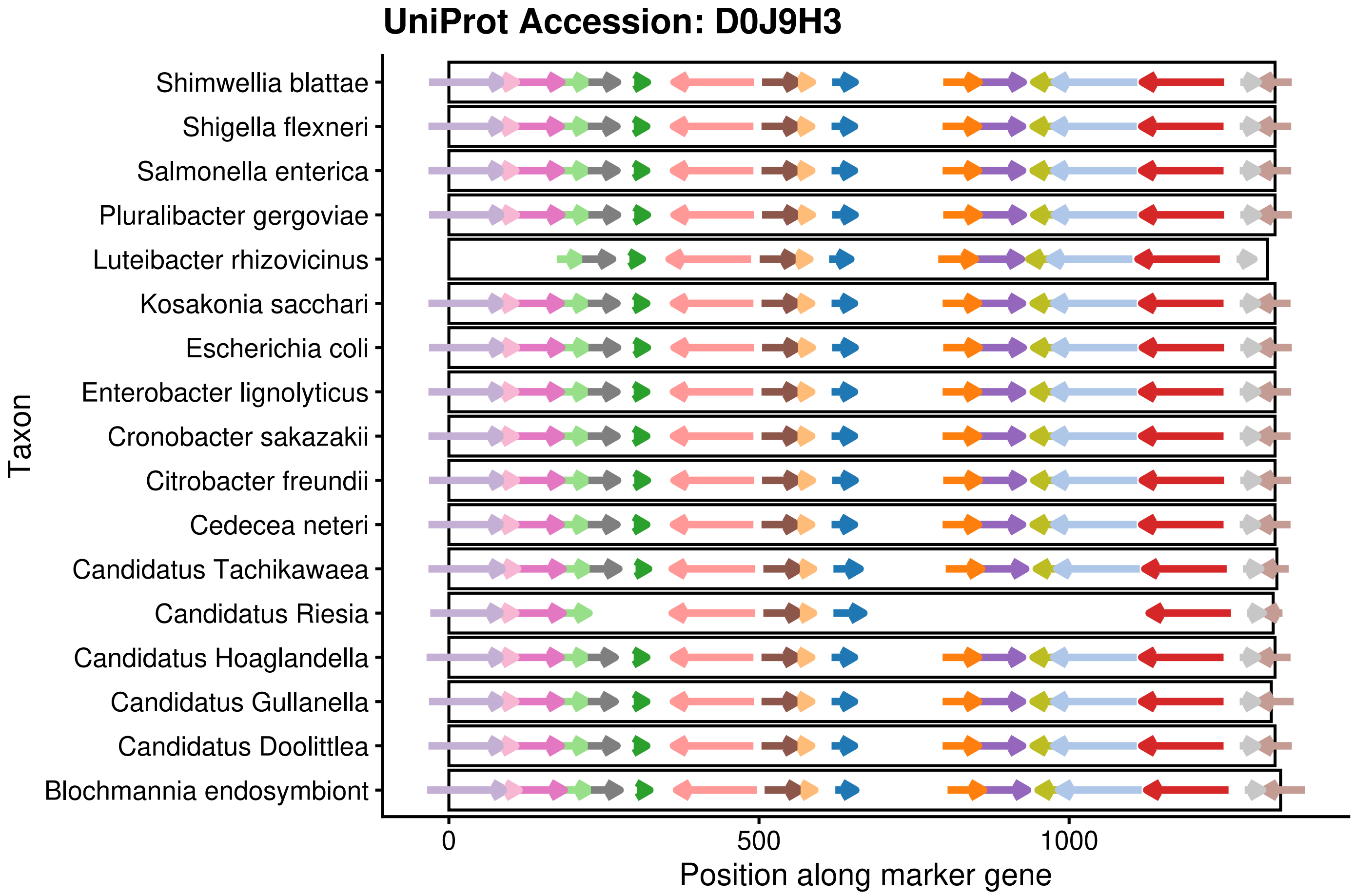

UniProt Accession: D0J9H3
Shimwellia blattae
Shigella flexneri
Salmonella enterica
Pluralibacter gergoviae
Luteibacter rhizovicinus
Kosakonia sacchari
Escherichia coli
Enterobacter lignolyticus
Cronobacter sakazakii
Citrobacter freundii
Cedecea neteri
Candidatus Tachikawaea
Candidatus Riesia
Candidatus Hoaglandella
Candidatus Gullanella
Candidatus Doolittlea
Blochmannia endosymbiont
Taxon
0
500
1000
Position along marker gene

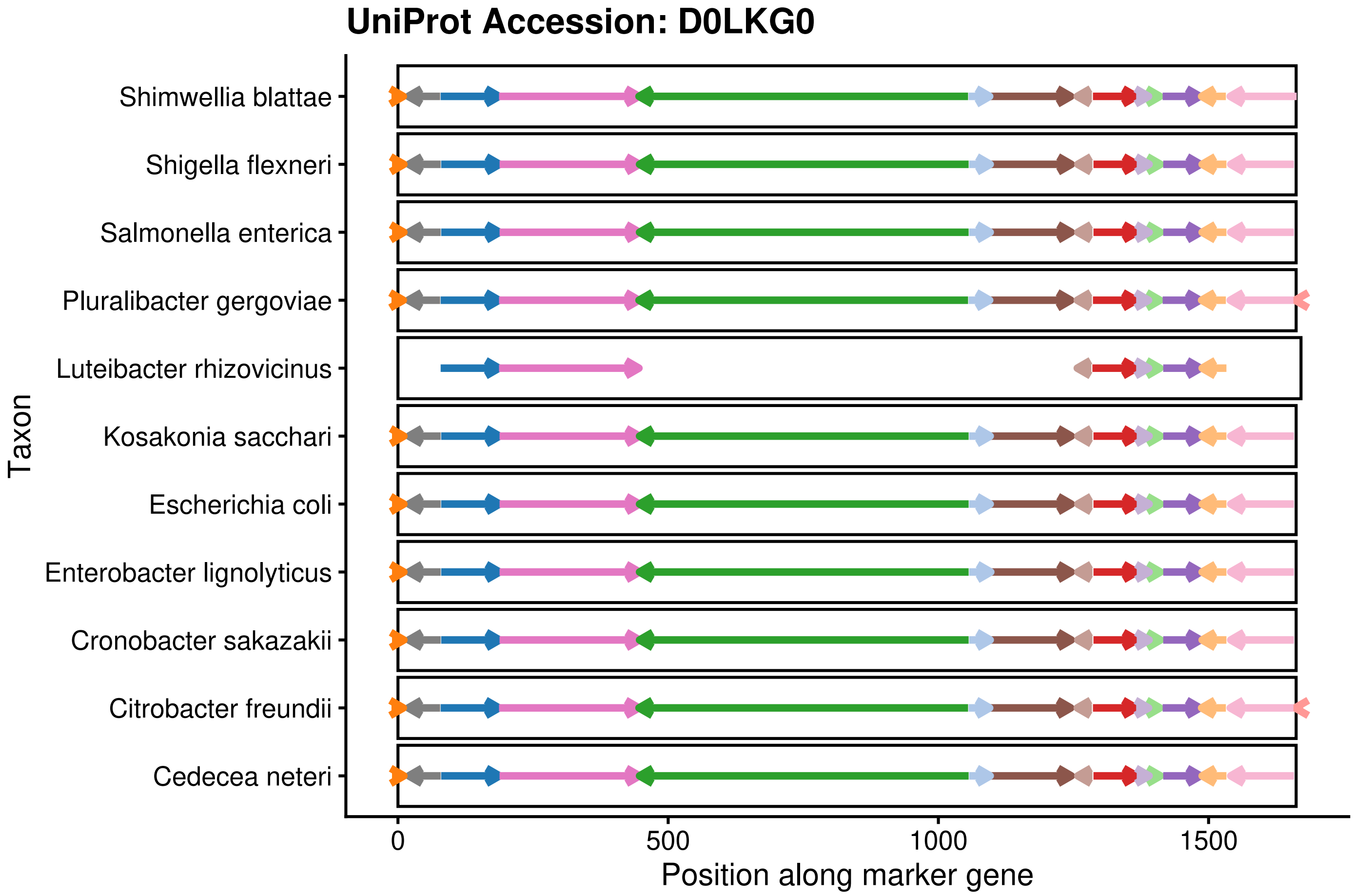
UniProt Accession: D0LKG0
Shimwellia blattae
Shigella flexneri
Salmonella enterica
Pluralibacter gergoviae
Luteibacter rhizovicinus
Kosakonia sacchari
Escherichia coli
Enterobacter lignolyticus
Cronobacter sakazakii
Citrobacter freundii
Cedecea neteri
Taxon
0
500
1000
1500
Position along marker gene

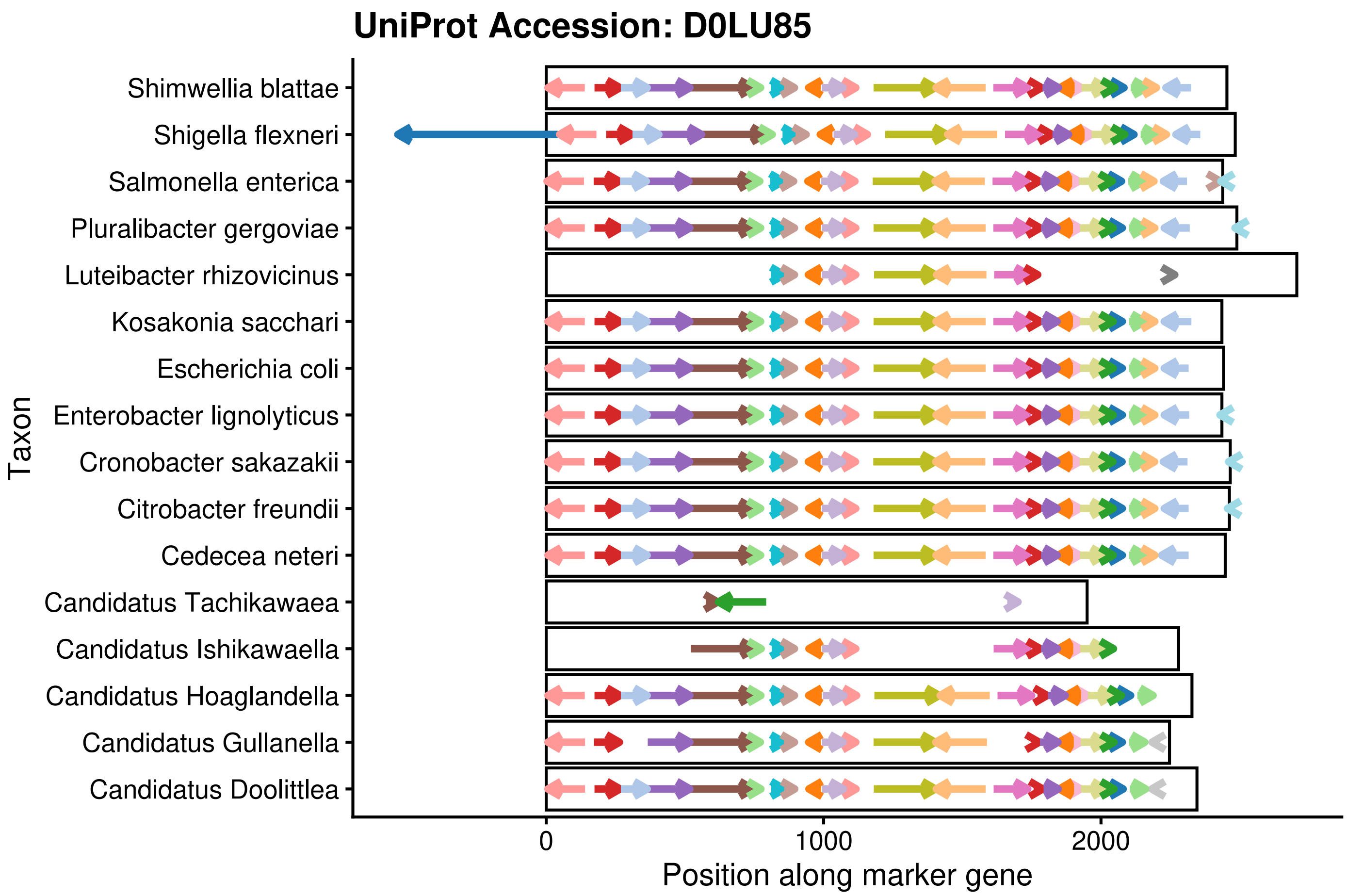
UniProt Accession: D0LU85
Shimwellia blattae
Shigella flexneri
Salmonella enterica
Pluralibacter gergoviae
Luteibacter rhizovicinus
Kosakonia sacchari
Escherichia coli
Enterobacter lignolyticus
Cronobacter sakazakii
Citrobacter freundii
Cedecea neteri
Candidatus Tachikawaea
Candidatus Ishikawaella
Candidatus Hoaglandella
Candidatus Gullanella
Candidatus Doolittlea
Taxon
0
1000
2000
Position along marker gene

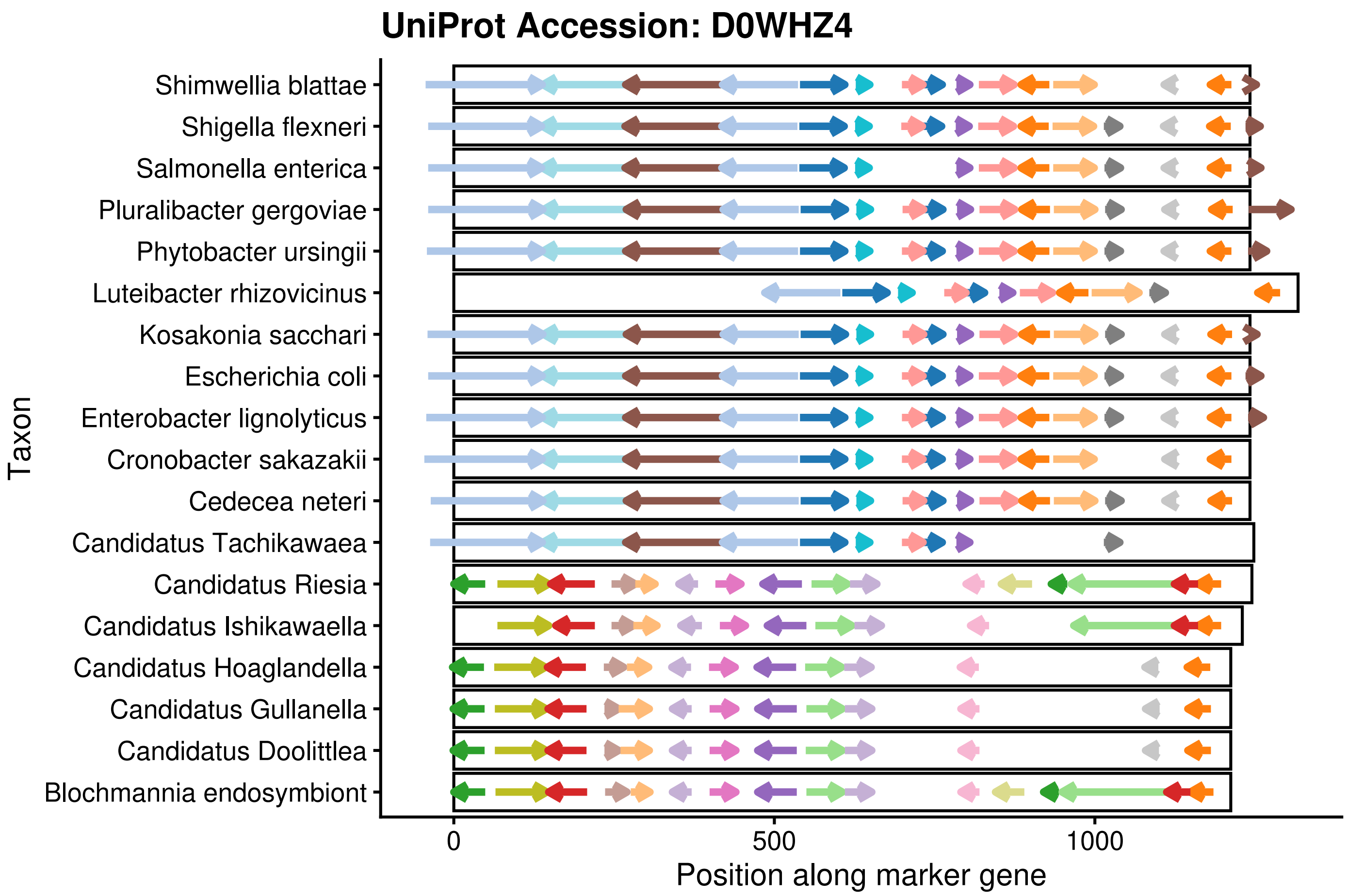
UniProt Accession: D0WHZ4
Shimwellia blattae
Shigella flexneri
Salmonella enterica
Pluralibacter gergoviae
Phytobacter ursingii
Luteibacter rhizovicinus
Kosakonia sacchari
Escherichia coli
Enterobacter lignolyticus
Cronobacter sakazakii
Cedecea neteri
Candidatus Tachikawaea
Candidatus Riesia
Candidatus Ishikawaella
Candidatus Hoaglandella
Candidatus Gullanella
Candidatus Doolittlea
Blochmannia endosymbiont
Taxon
0
500
1000
Position along marker gene

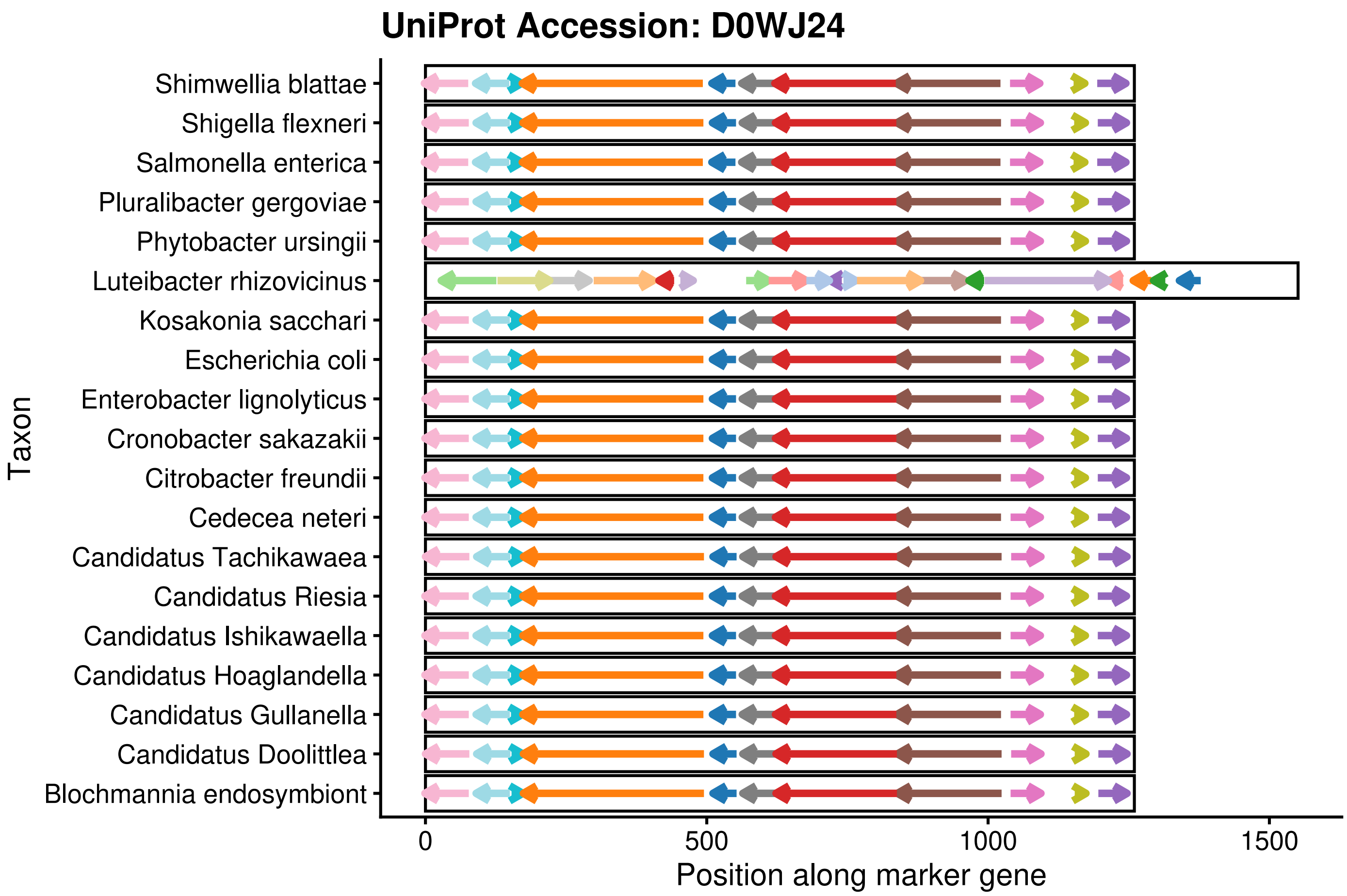
UniProt Accession: D0WJ24
Shimwellia blattae
Shigella flexneri
Salmonella enterica
Pluralibacter gergoviae
Phytobacter ursingii
Luteibacter rhizovicinus
Kosakonia sacchari
Escherichia coli
Enterobacter lignolyticus
Cronobacter sakazakii
Citrobacter freundii
Cedecea neteri
Candidatus Tachikawaea
Candidatus Riesia
Candidatus Ishikawaella
Candidatus Hoaglandella
Candidatus Gullanella
Candidatus Doolittlea
Blochmannia endosymbiont
Taxon
0
500
1000
1500
Position along marker gene

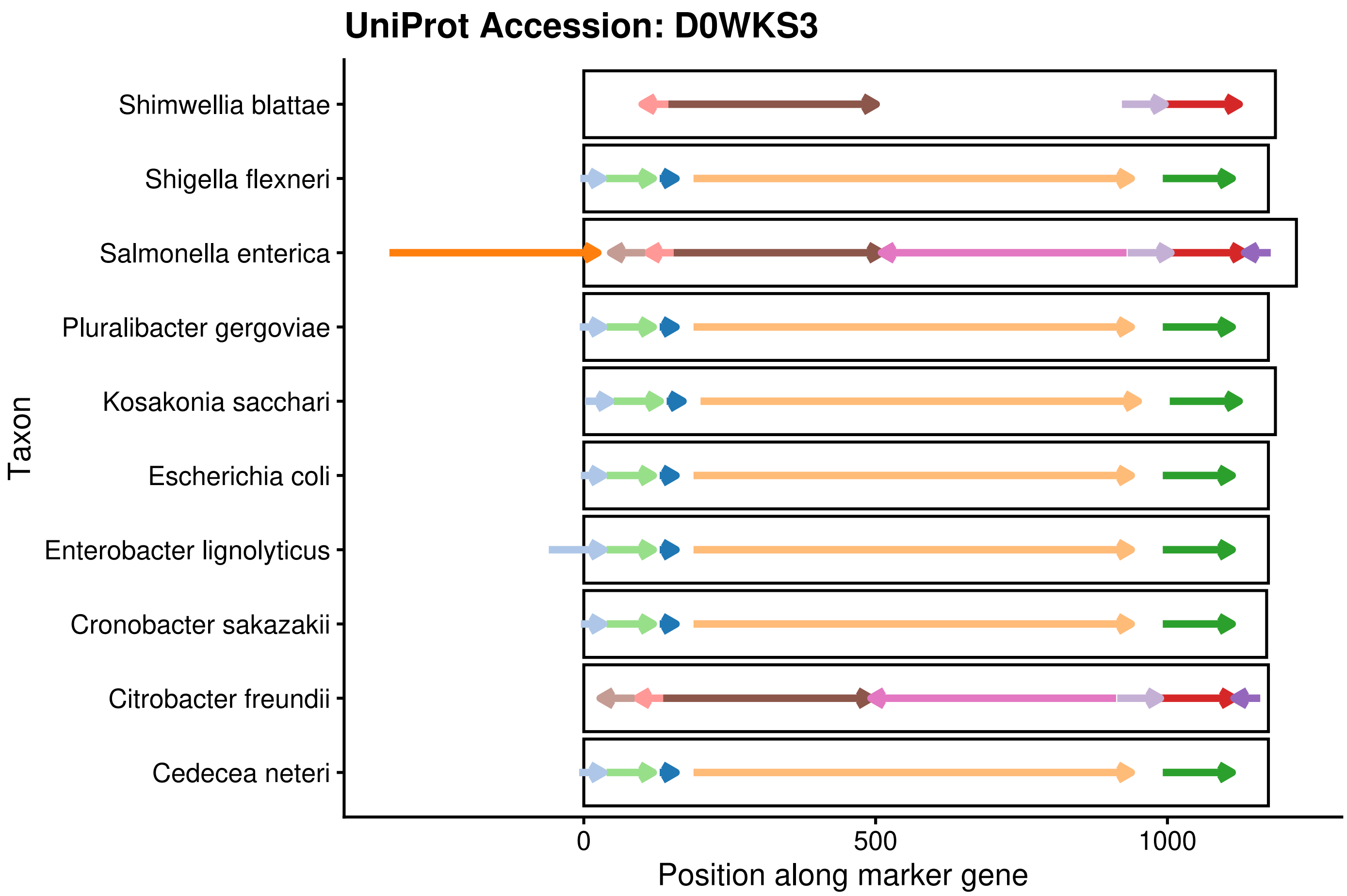
UniProt Accession: D0WKS3
Shimwellia blattae
Shigella flexneri
Salmonella enterica
Pluralibacter gergoviae
Kosakonia sacchari
Escherichia coli
Enterobacter lignolyticus
Cronobacter sakazakii
Citrobacter freundii
Cedecea neteri
Taxon
0
500
1000
Position along marker gene

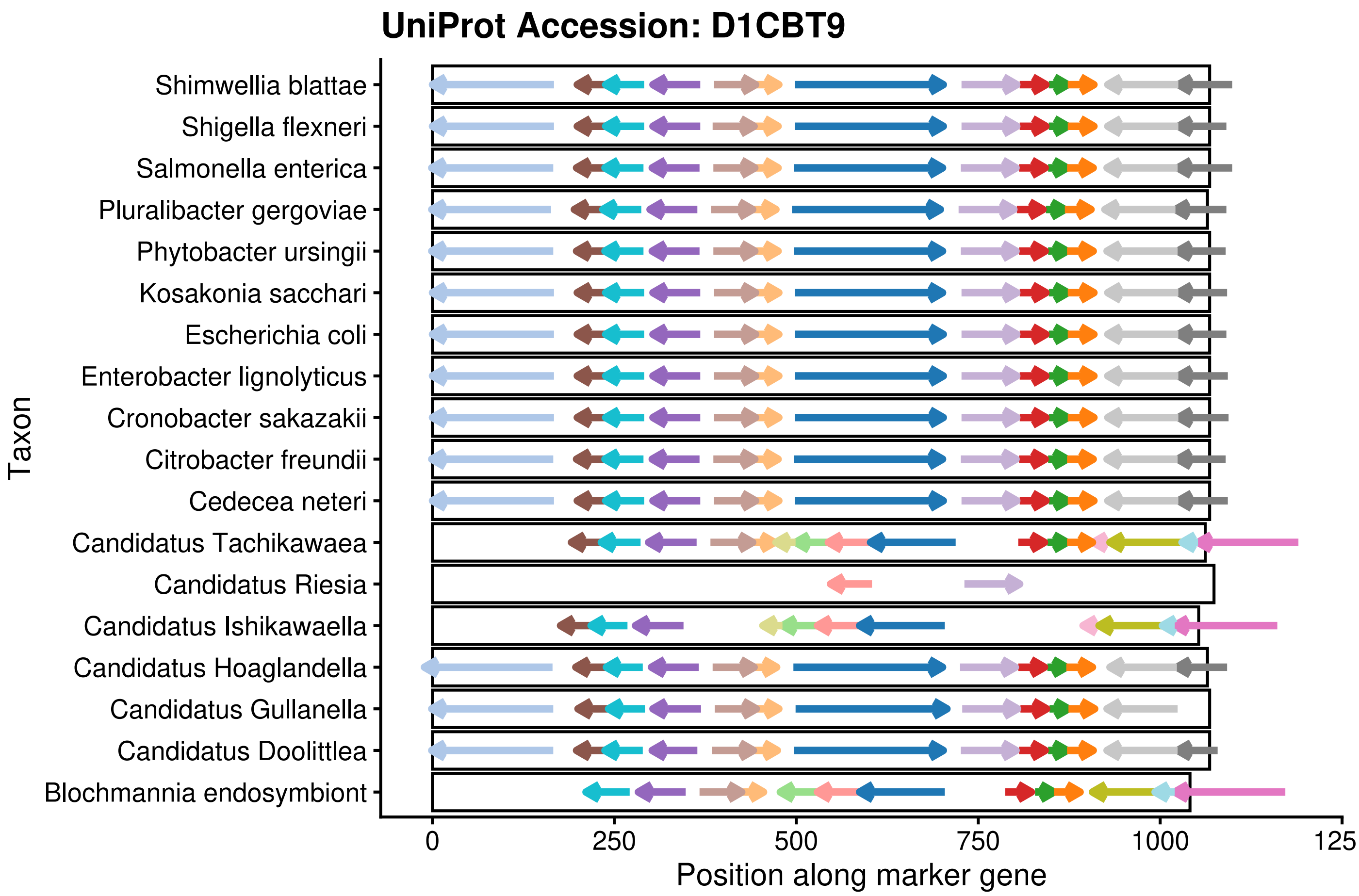
UniProt Accession: D1CBT9
Taxon
Shimwellia blattae
Shigella flexneri
Salmonella enterica
Pluralibacter gergoviae
Phytobacter ursingii
Kosakonia sacchari
Escherichia coli
Enterobacter lignolyticus
Cronobacter sakazakii
Citrobacter freundii
Cedecea neteri
Candidatus Tachikawaea
Candidatus Riesia
Candidatus Ishikawaella
Candidatus Hoaglandella
Candidatus Gullanella
Candidatus Doolittlea
Blochmannia endosymbiont
0
250
500
750
1000
125
Position along marker gene

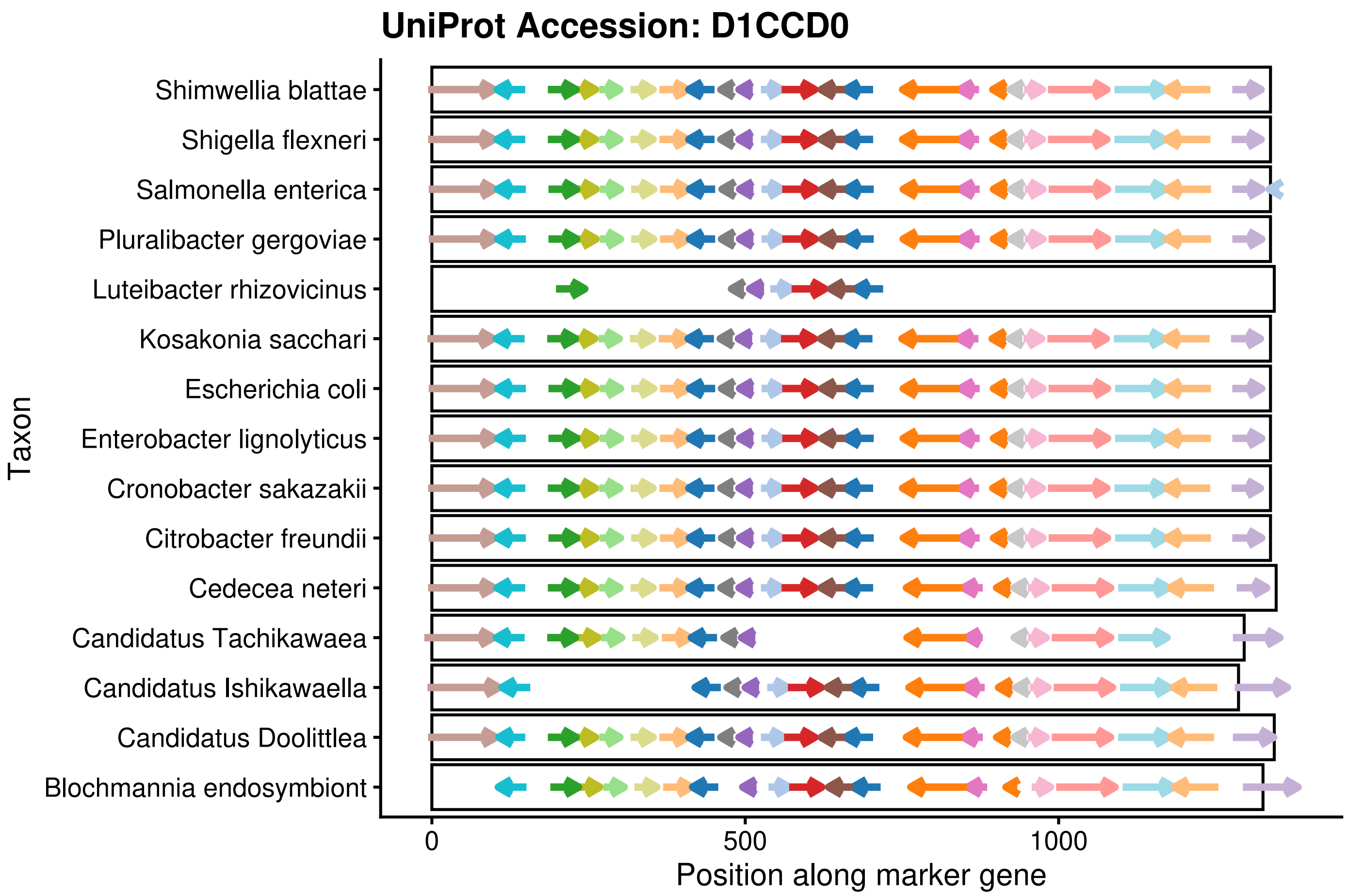
UniProt Accession: D1CCD0
Taxon
Shimwellia blattae
Shigella flexneri
Salmonella enterica
Pluralibacter gergoviae
Luteibacter rhizovicinus
Kosakonia sacchari
Escherichia coli
Enterobacter lignolyticus
Cronobacter sakazakii
Citrobacter freundii
Cedecea neteri
Candidatus Tachikawaea
Candidatus Ishikawaella
Candidatus Doolittlea
Blochmannia endosymbiont
0
500
1000
Position along marker gene

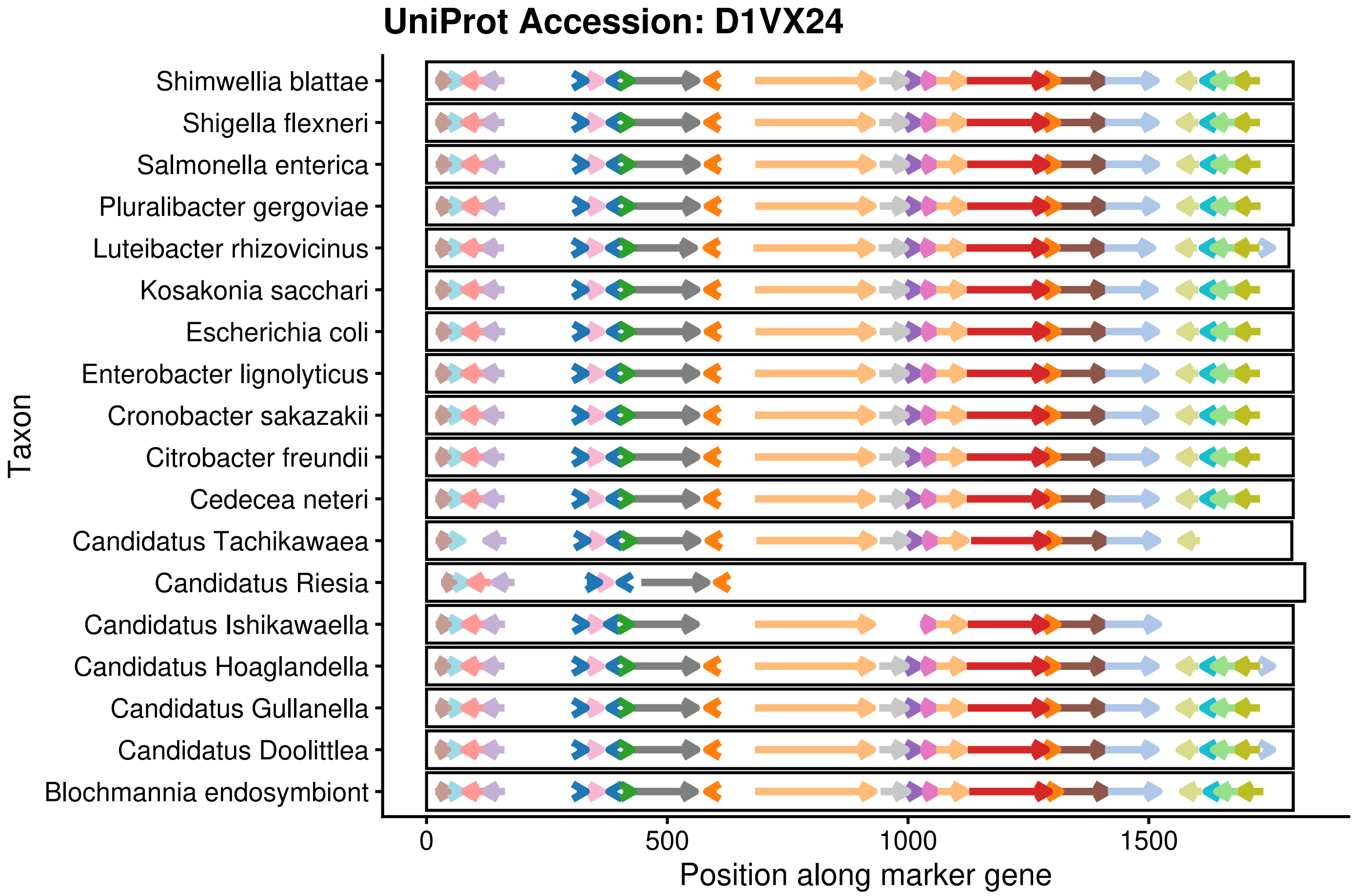

UniProt Accession: D1VX24
Taxon
Shimwellia blattae
Shigella flexneri
Salmonella enterica
Pluralibacter gergoviae
Luteibacter rhizovicinus
Kosakonia sacchari
Escherichia coli
Enterobacter lignolyticus
Cronobacter sakazakii
Citrobacter freundii
Cedecea neteri
Candidatus Tachikawaea
Candidatus Riesia
Candidatus Ishikawaella
Candidatus Hoaglandella
Candidatus Gullanella
Candidatus Doolittlea
Blochmannia endosymbiont
0
500
1000
1500
Position along marker gene

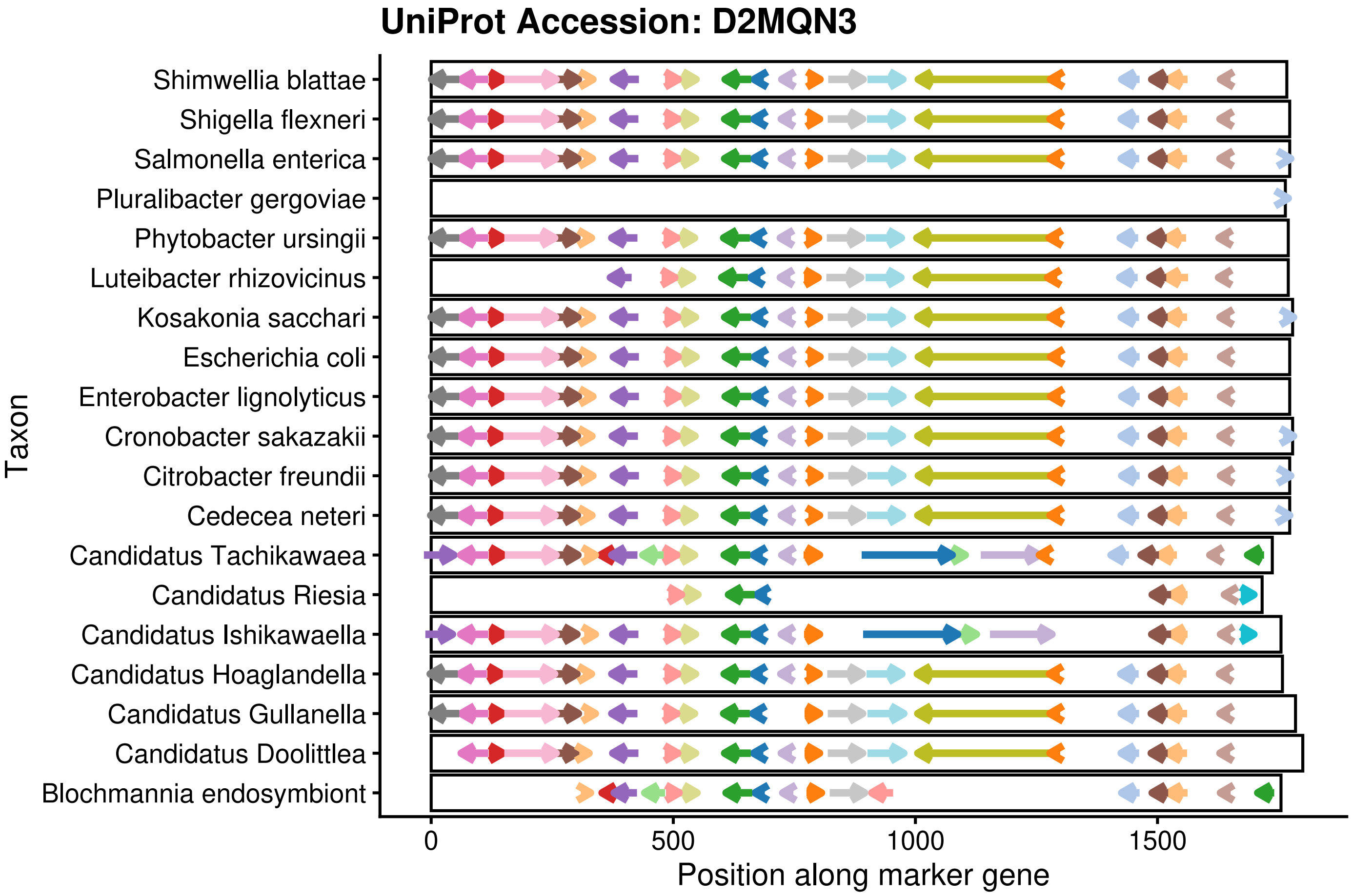
UniProt Accession: D2MQN3
Taxon
Shimwellia blattae
Shigella flexneri
Salmonella enterica
Pluralibacter gergoviae
Phytobacter ursingii
Luteibacter rhizovicinus
Kosakonia sacchari
Escherichia coli
Enterobacter lignolyticus
Cronobacter sakazakii
Citrobacter freundii
Cedecea neteri
Candidatus Tachikawaea
Candidatus Riesia
Candidatus Ishikawaella
Candidatus Hoaglandella
Candidatus Gullanella
Candidatus Doolittlea
Blochmannia endosymbiont
0
500
1000
1500
Position along marker gene

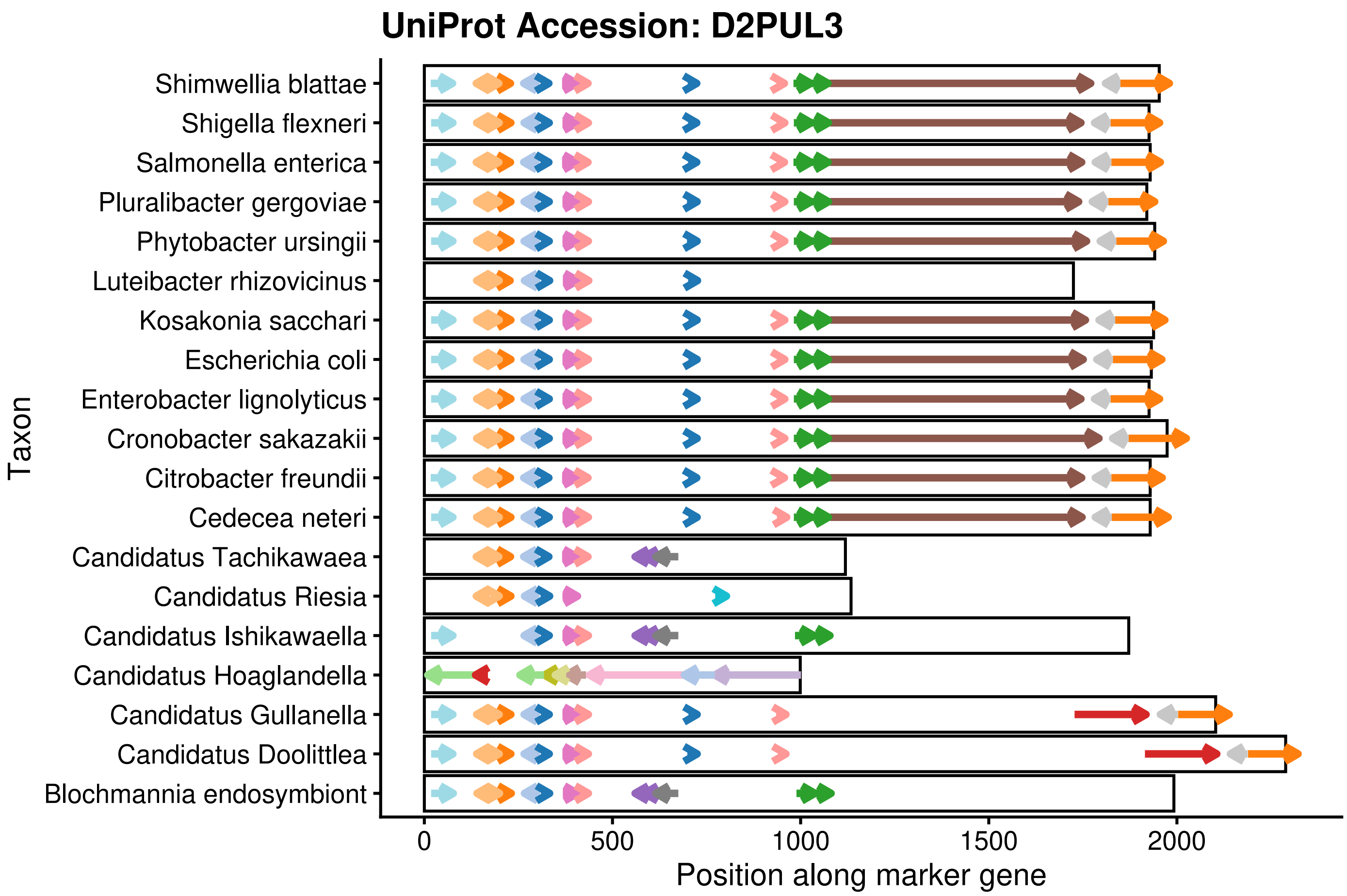

UniProt Accession: D2PUL3
Taxon
Shimwellia blattae
Shigella flexneri
Salmonella enterica
Pluralibacter gergoviae
Phytobacter ursingii
Luteibacter rhizovicinus
Kosakonia sacchari
Escherichia coli
Enterobacter lignolyticus
Cronobacter sakazakii
Citrobacter freundii
Cedecea neteri
Candidatus Tachikawaea
Candidatus Riesia
Candidatus Ishikawaella
Candidatus Hoaglandella
Candidatus Gullanella
Candidatus Doolittlea
Blochmannia endosymbiont
0
500
1000
1500
2000
Position along marker gene

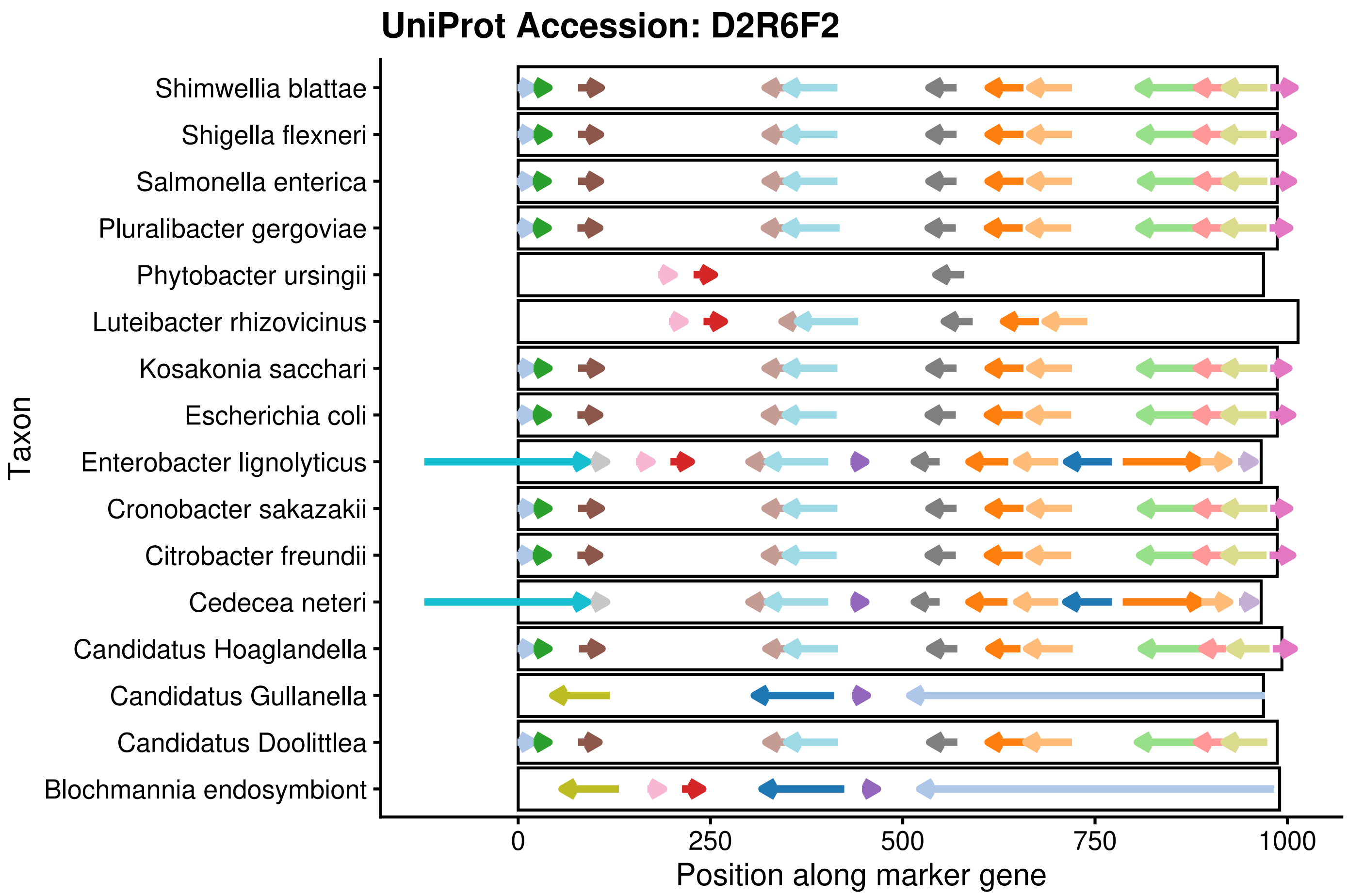
UniProt Accession: D2R6F2
Shimwellia blattae
Shigella flexneri
Salmonella enterica
Pluralibacter gergoviae
Phytobacter ursingii
Luteibacter rhizovicinus
Kosakonia sacchari
Escherichia coli
Enterobacter lignolyticus
Cronobacter sakazakii
Citrobacter freundii
Cedecea neteri
Candidatus Hoaglandella
Candidatus Gullanella
Candidatus Doolittlea
Blochmannia endosymbiont
Taxon
0
250
500
750
1000
Position along marker gene

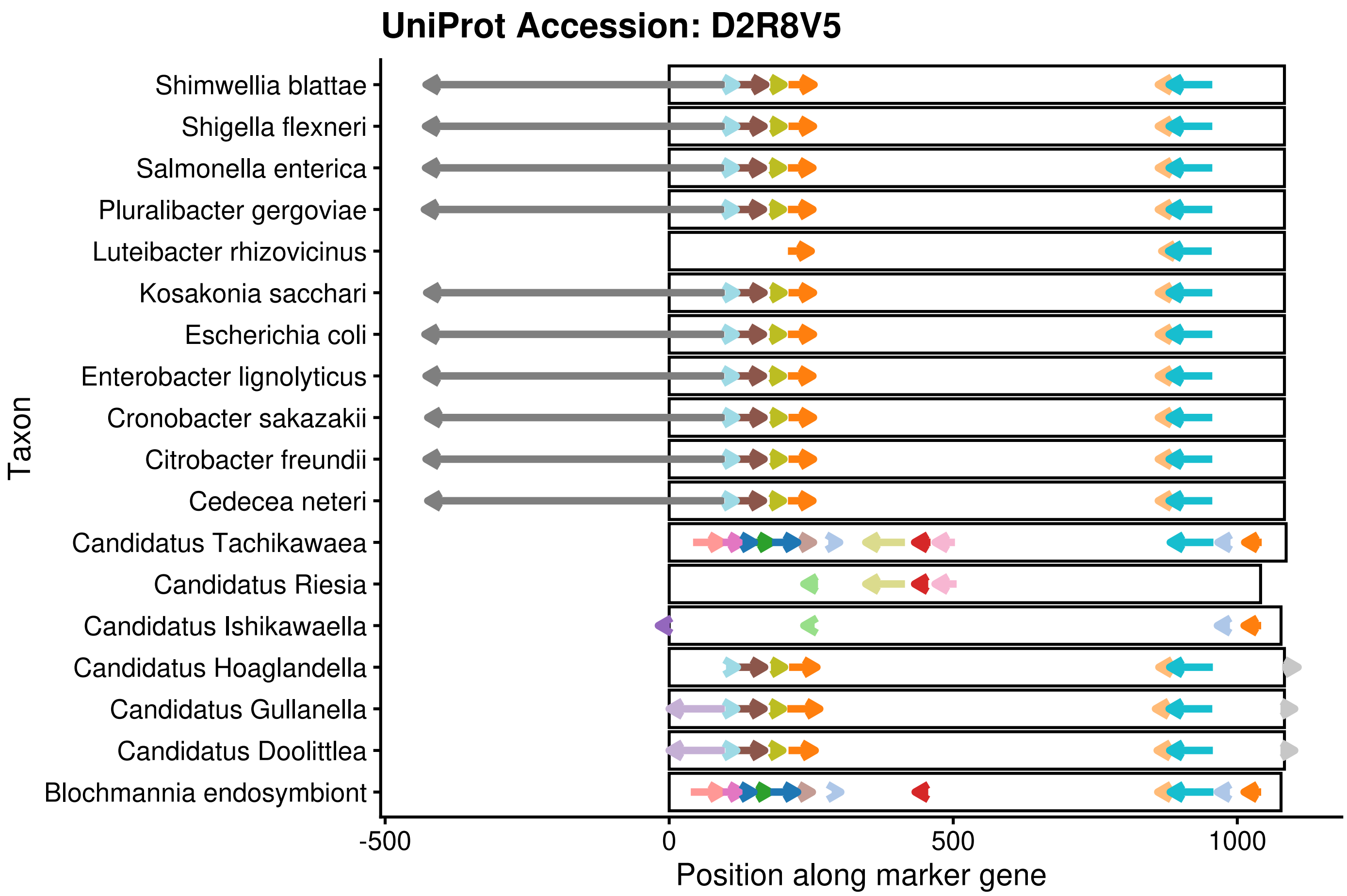
UniProt Accession: D2R8V5
Shimwellia blattae
Shigella flexneri
Salmonella enterica
Pluralibacter gergoviae
Luteibacter rhizovicinus
Kosakonia sacchari
Escherichia coli
Enterobacter lignolyticus
Cronobacter sakazakii
Citrobacter freundii
Cedecea neteri
Candidatus Tachikawaea
Candidatus Riesia
Candidatus Ishikawaella
Candidatus Hoaglandella
Candidatus Gullanella
Candidatus Doolittlea
Blochmannia endosymbiont
Taxon
-500
0
500
1000
Position along marker gene

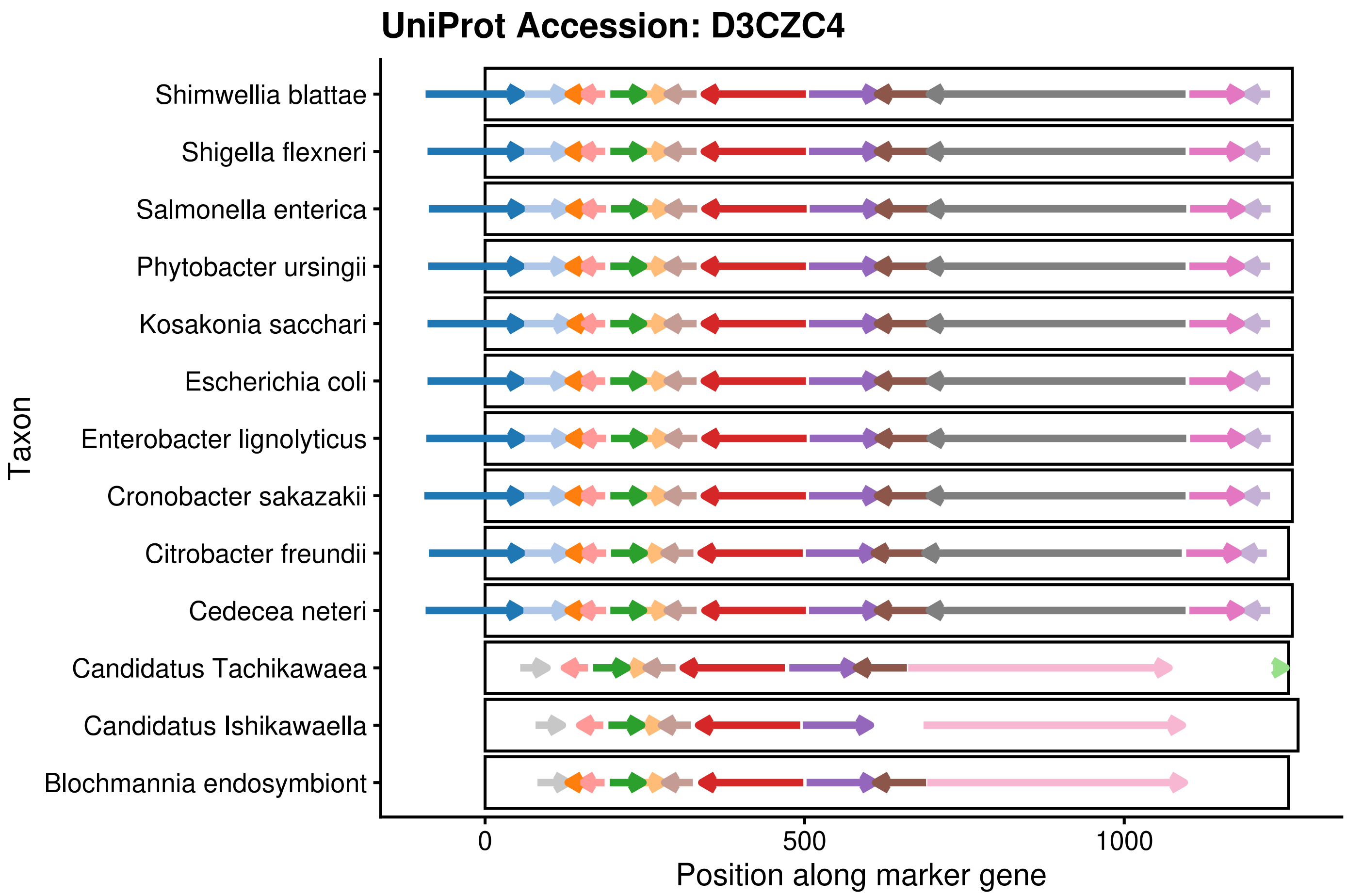
UniProt Accession: D3CZC4
Taxon
Shimwellia blattae
Shigella flexneri
Salmonella enterica
Phytobacter ursingii
Kosakonia sacchari
Escherichia coli
Enterobacter lignolyticus
Cronobacter sakazakii
Citrobacter freundii
Cedecea neteri
Candidatus Tachikawaea
Candidatus Ishikawaella
Blochmannia endosymbiont
0
500
1000
Position along marker gene

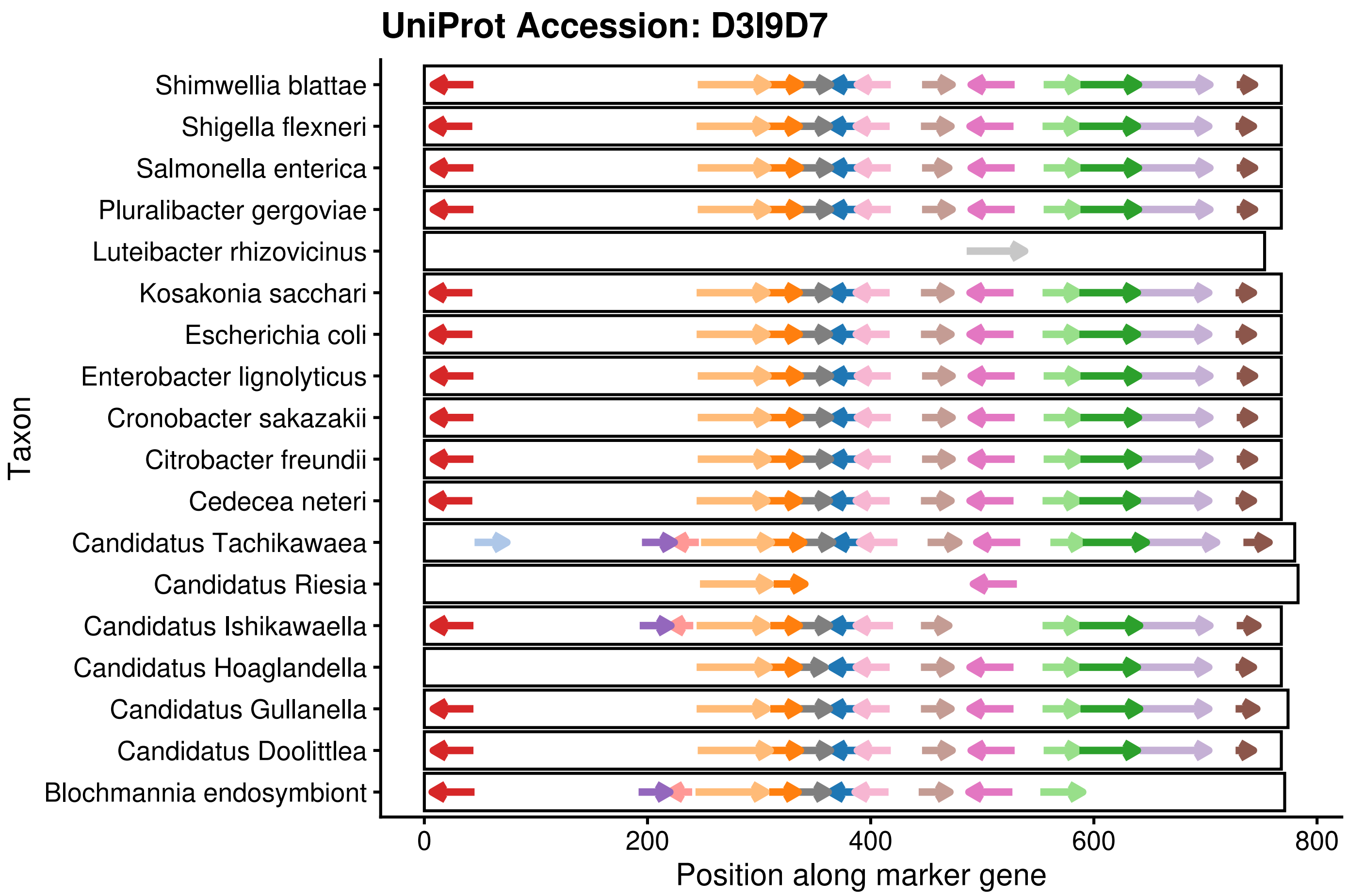
UniProt Accession: D3I9D7
Shimwellia blattae
Shigella flexneri
Salmonella enterica
Pluralibacter gergoviae
Luteibacter rhizovicinus
Kosakonia sacchari
Escherichia coli
Enterobacter lignolyticus
Cronobacter sakazakii
Citrobacter freundii
Cedecea neteri
Candidatus Tachikawaea
Candidatus Riesia
Candidatus Ishikawaella
Candidatus Hoaglandella
Candidatus Gullanella
Candidatus Doolittlea
Blochmannia endosymbiont
Taxon
0
200
400
600
800
Position along marker gene

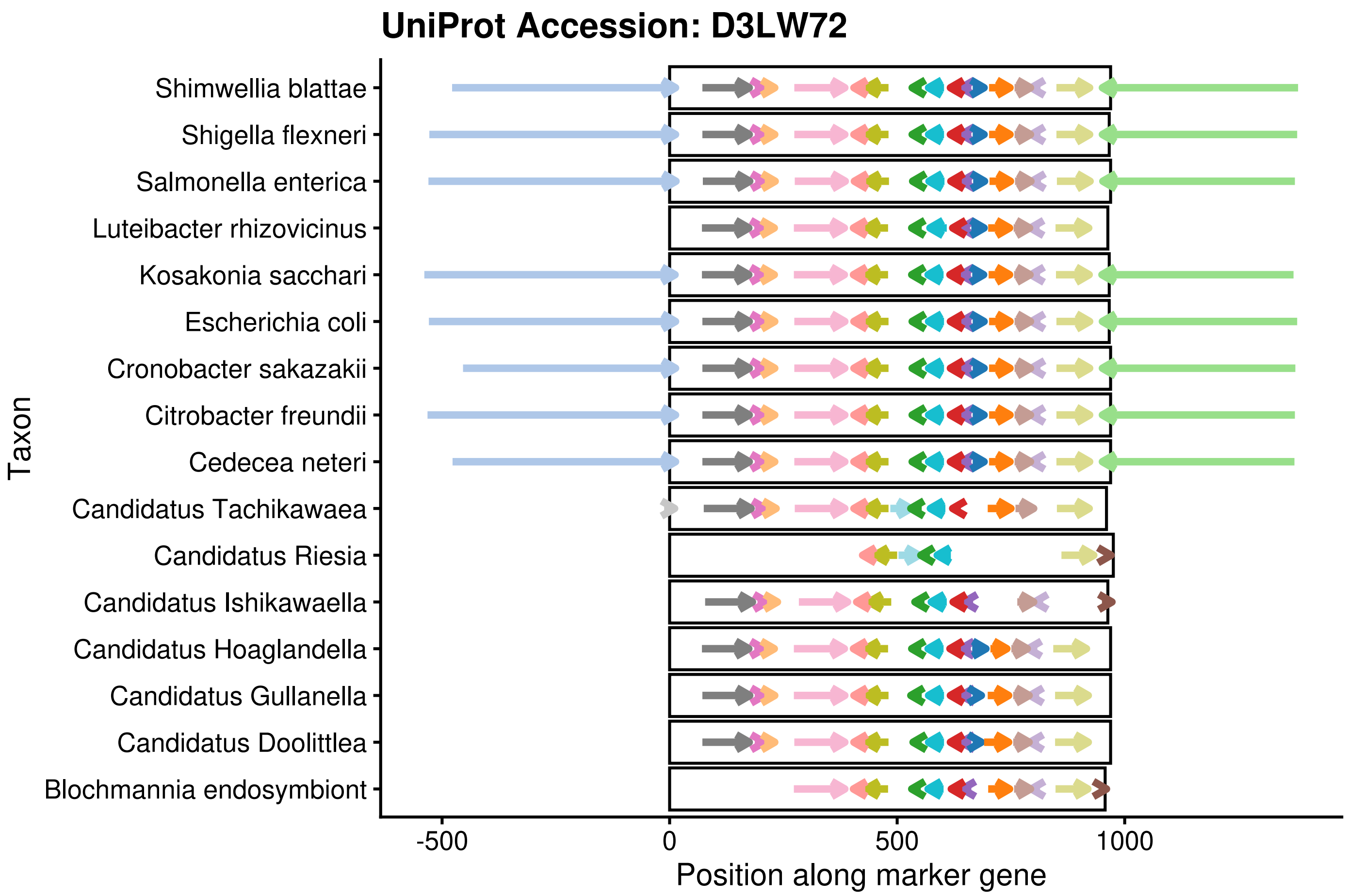
UniProt Accession: D3LW72
Taxon
Shimwellia blattae
Shigella flexneri
Salmonella enterica
Luteibacter rhizovicinus
Kosakonia sacchari
Escherichia coli
Cronobacter sakazakii
Citrobacter freundii
Cedecea neteri
Candidatus Tachikawaea
Candidatus Riesia
Candidatus Ishikawaella
Candidatus Hoaglandella
Candidatus Gullanella
Candidatus Doolittlea
Blochmannia endosymbiont
-500
0
500
1000
Position along marker gene

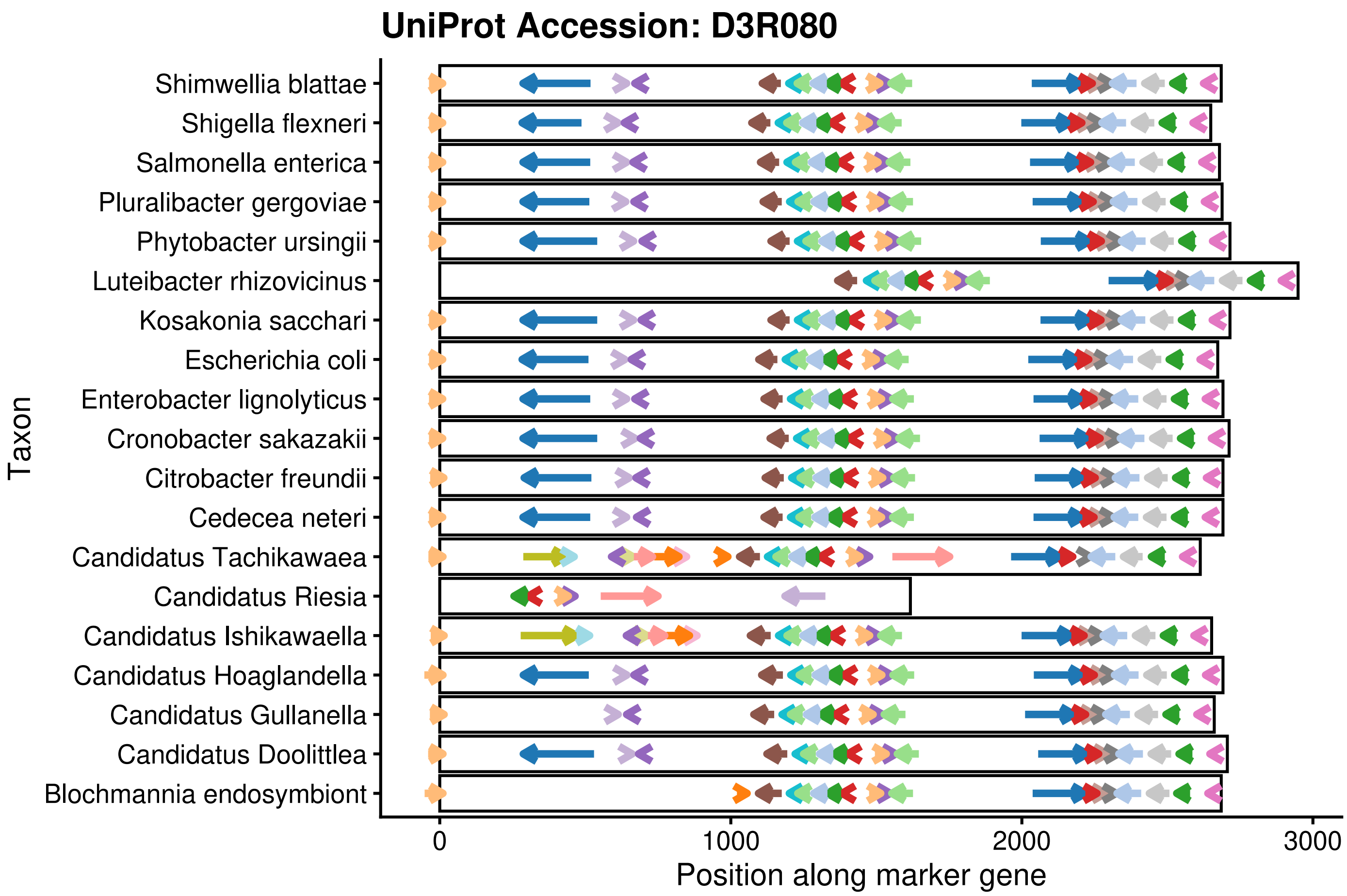
UniProt Accession: D3R080
Taxon
Shimwellia blattae
Shigella flexneri
Salmonella enterica
Pluralibacter gergoviae
Phytobacter ursingii
Luteibacter rhizovicinus
Kosakonia sacchari
Escherichia coli
Enterobacter lignolyticus
Cronobacter sakazakii
Citrobacter freundii
Cedecea neteri
Candidatus Tachikawaea
Candidatus Riesia
Candidatus Ishikawaella
Candidatus Hoaglandella
Candidatus Gullanella
Candidatus Doolittlea
Blochmannia endosymbiont
0
1000
2000
3000
Position along marker gene

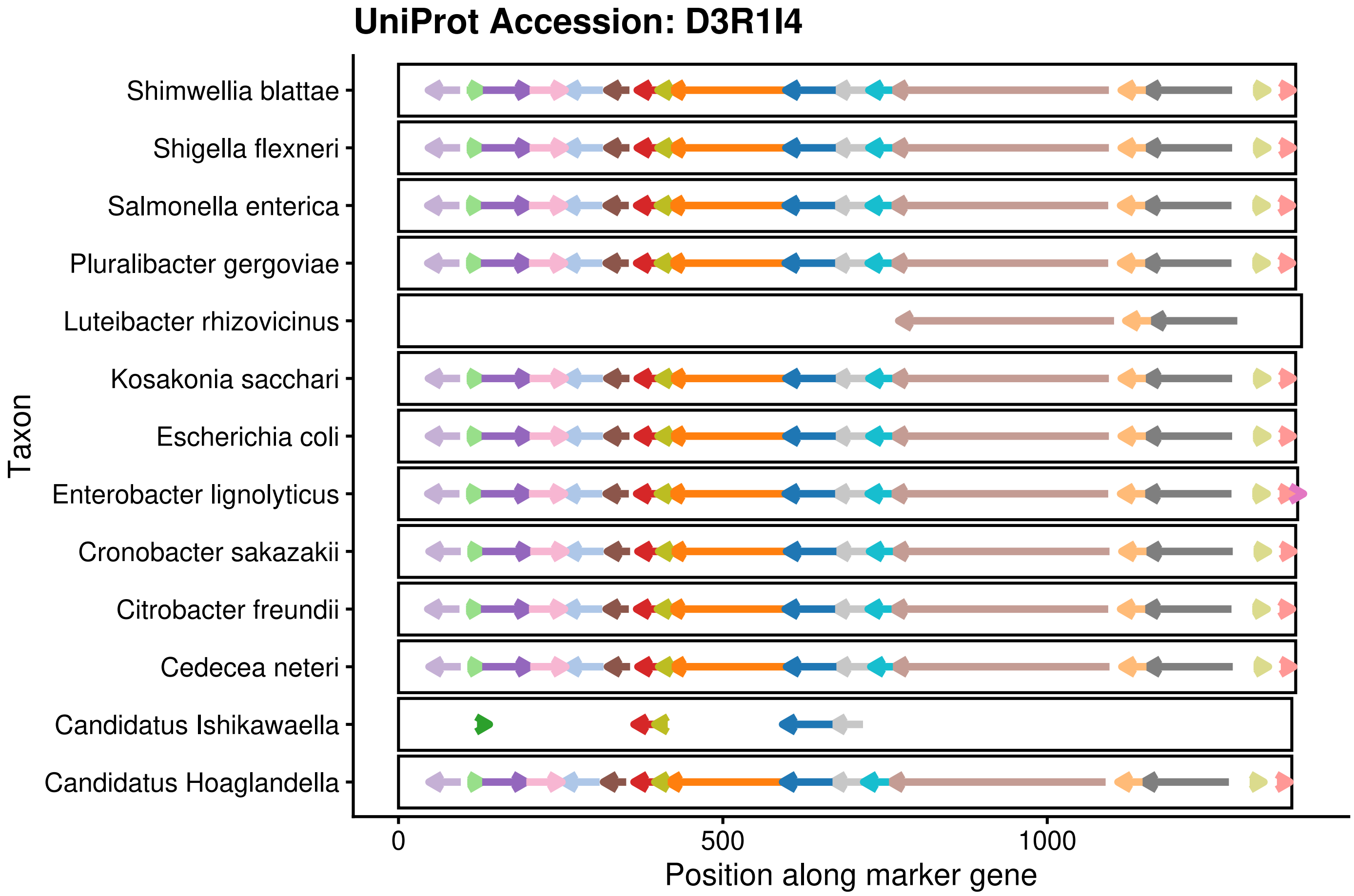
UniProt Accession: D3R1I4
Taxon
Shimwellia blattae
Shigella flexneri
Salmonella enterica
Pluralibacter gergoviae
Luteibacter rhizovicinus
Kosakonia sacchari
Escherichia coli
Enterobacter lignolyticus
Cronobacter sakazakii
Citrobacter freundii
Cedecea neteri
Candidatus Ishikawaella
Candidatus Hoaglandella
0
500
1000
Position along marker gene

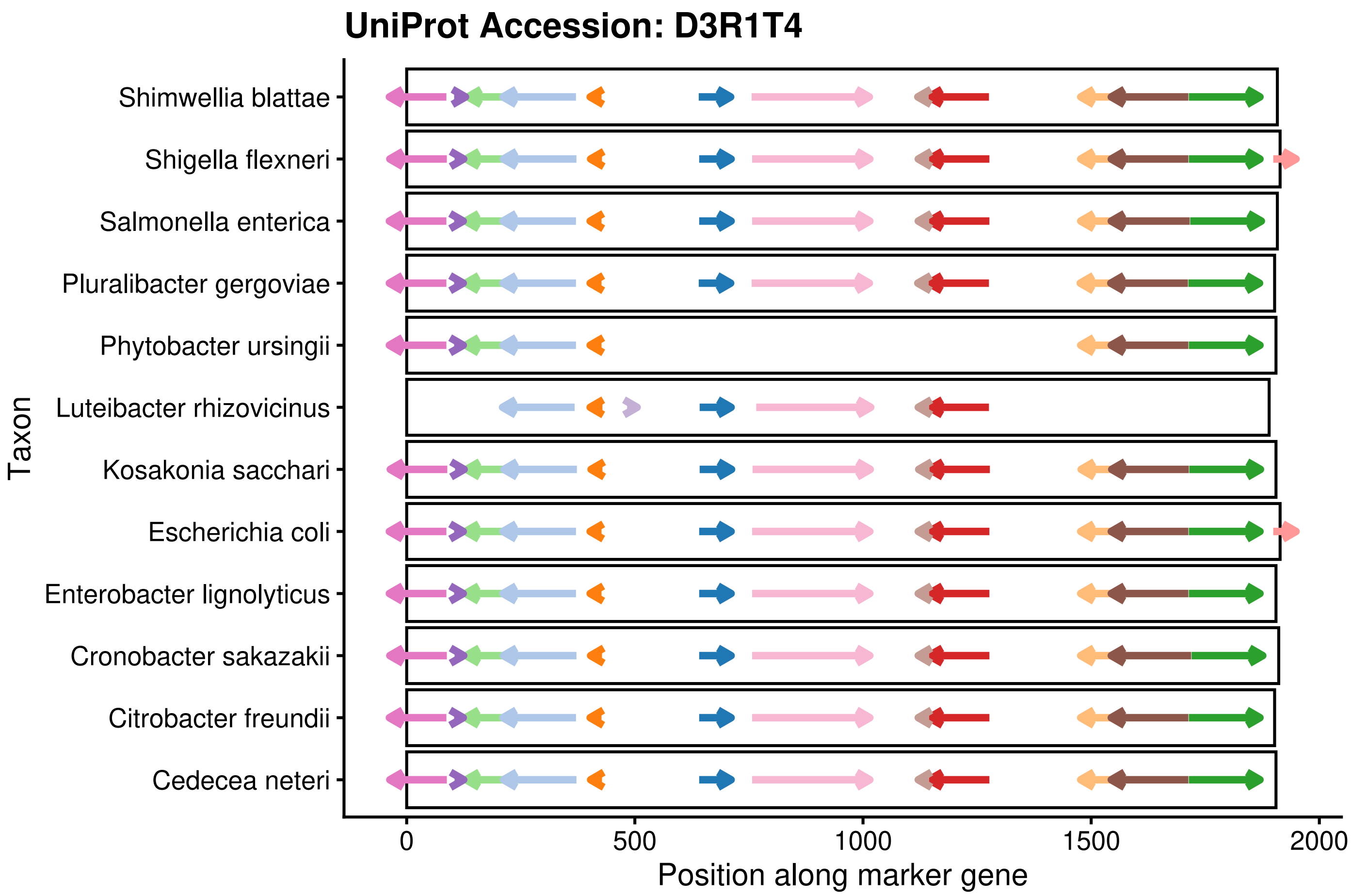

UniProt Accession: D3R1T4
Shimwellia blattae
Shigella flexneri
Salmonella enterica
Pluralibacter gergoviae
Phytobacter ursingii
Luteibacter rhizovicinus
Kosakonia sacchari
Escherichia coli
Enterobacter lignolyticus
Cronobacter sakazakii
Citrobacter freundii
Cedecea neteri
Taxon
0
500
1000
1500
2000
Position along marker gene

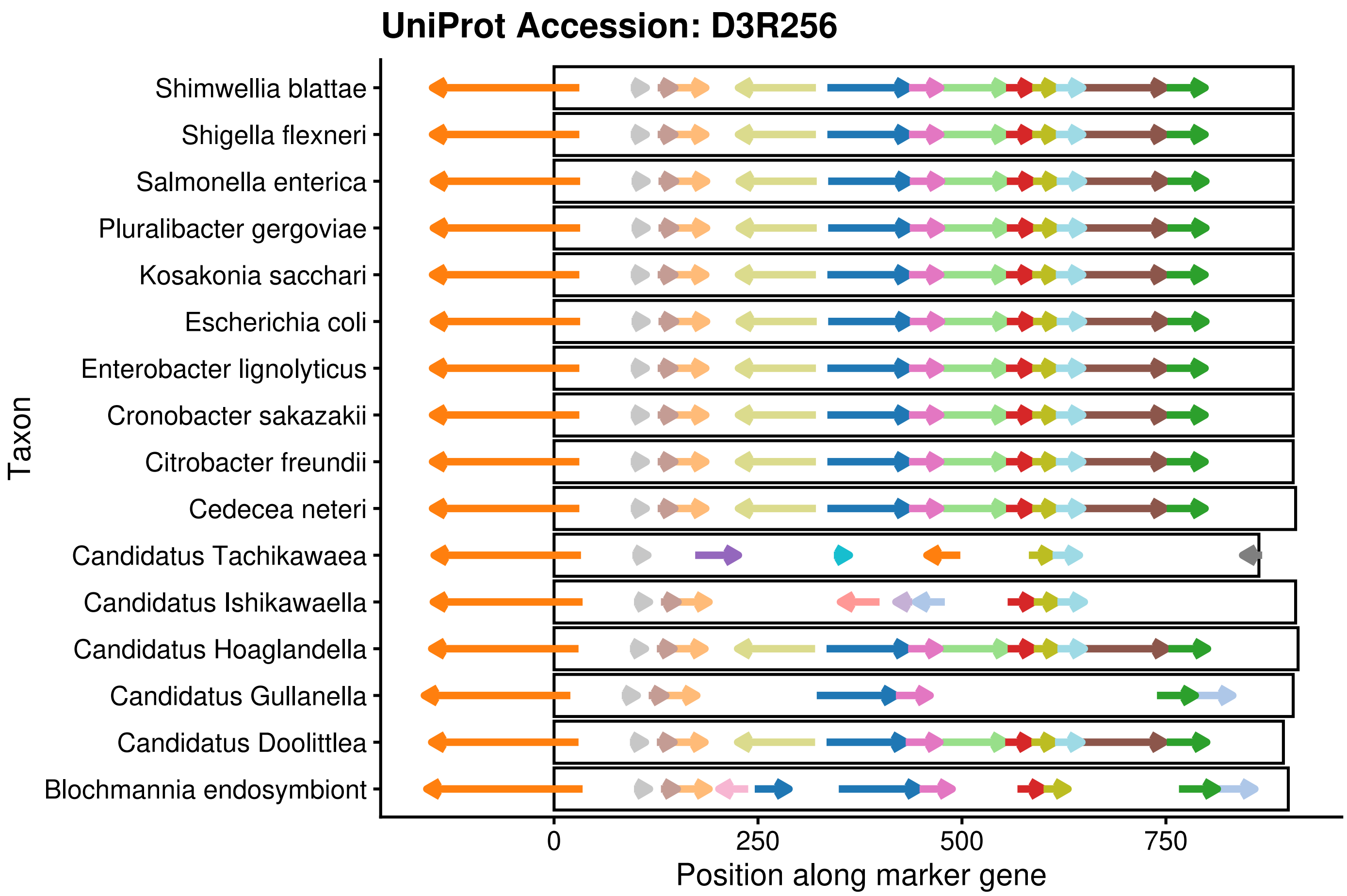

UniProt Accession: D3R256
Shimwellia blattae
Shigella flexneri
Salmonella enterica
Pluralibacter gergoviae
Kosakonia sacchari
Escherichia coli
Enterobacter lignolyticus
Cronobacter sakazakii
Citrobacter freundii
Cedecea neteri
Candidatus Tachikawaea
Candidatus Ishikawaella
Candidatus Hoaglandella
Candidatus Gullanella
Candidatus Doolittlea
Blochmannia endosymbiont
Taxon
0
250
500
750
Position along marker gene

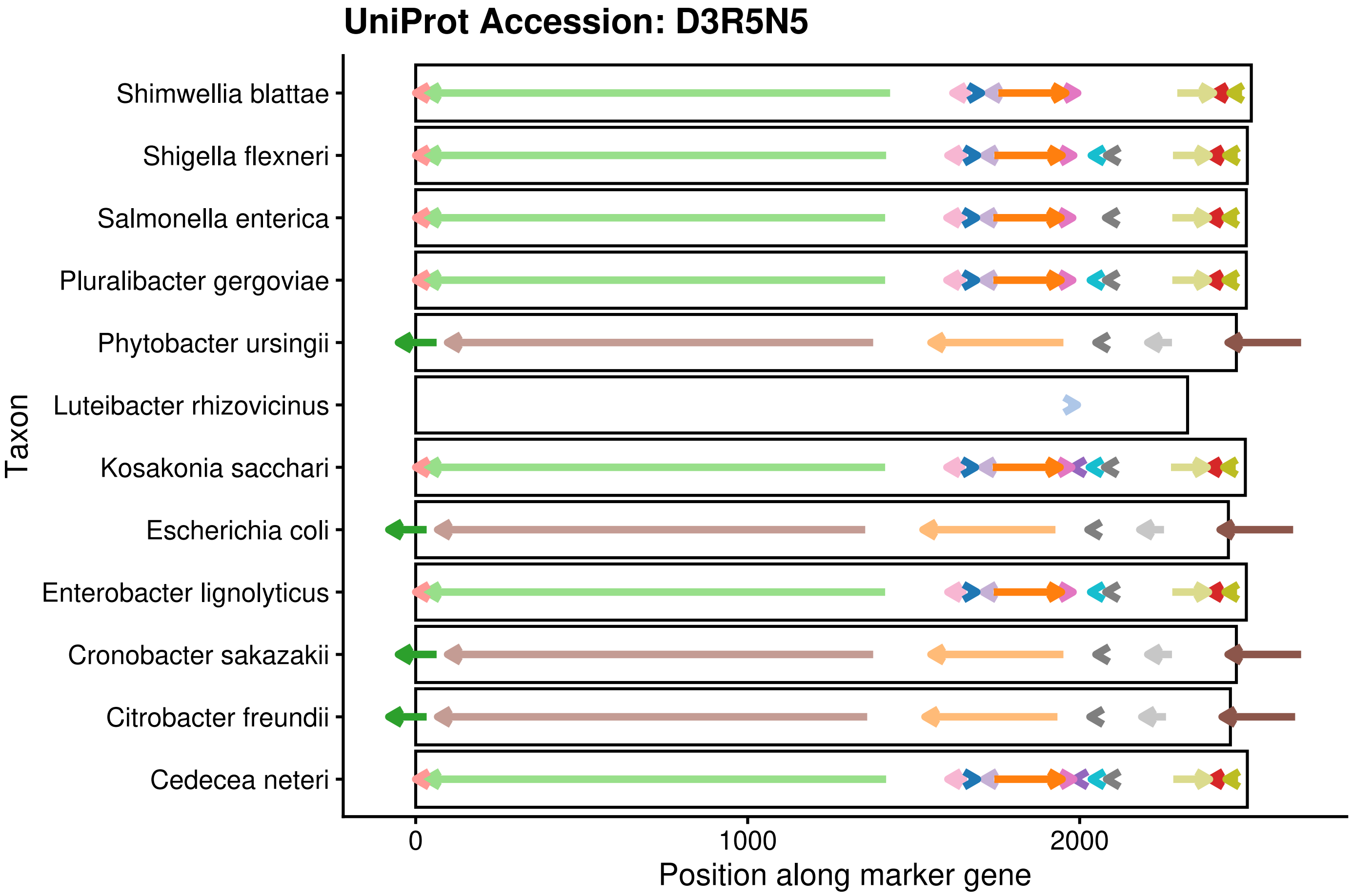
UniProt Accession: D3R5N5
Taxon
Shimwellia blattae
Shigella flexneri
Salmonella enterica
Pluralibacter gergoviae
Phytobacter ursingii
Luteibacter rhizovicinus
Kosakonia sacchari
Escherichia coli
Enterobacter lignolyticus
Cronobacter sakazakii
Citrobacter freundii
Cedecea neteri
0
1000
2000
Position along marker gene

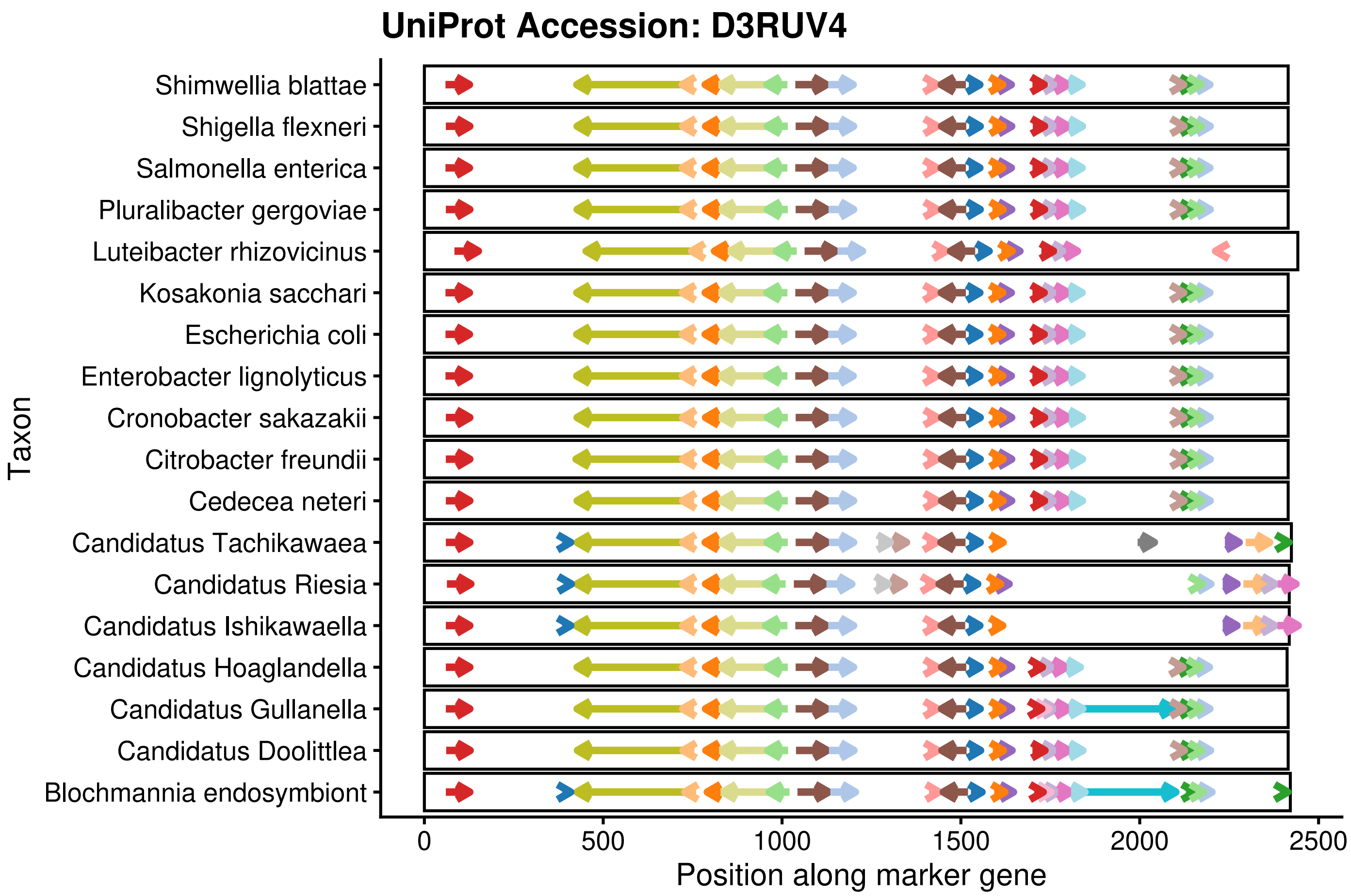
UniProt Accession: D3RUV4
Taxon
Shimwellia blattae
Shigella flexneri
Salmonella enterica
Pluralibacter gergoviae
Luteibacter rhizovicinus
Kosakonia sacchari
Escherichia coli
Enterobacter lignolyticus
Cronobacter sakazakii
Citrobacter freundii
Cedecea neteri
Candidatus Tachikawaea
Candidatus Riesia
Candidatus Ishikawaella
Candidatus Hoaglandella
Candidatus Gullanella
Candidatus Doolittlea
Blochmannia endosymbiont
0
500
1000
1500
2000
2500
Position along marker gene

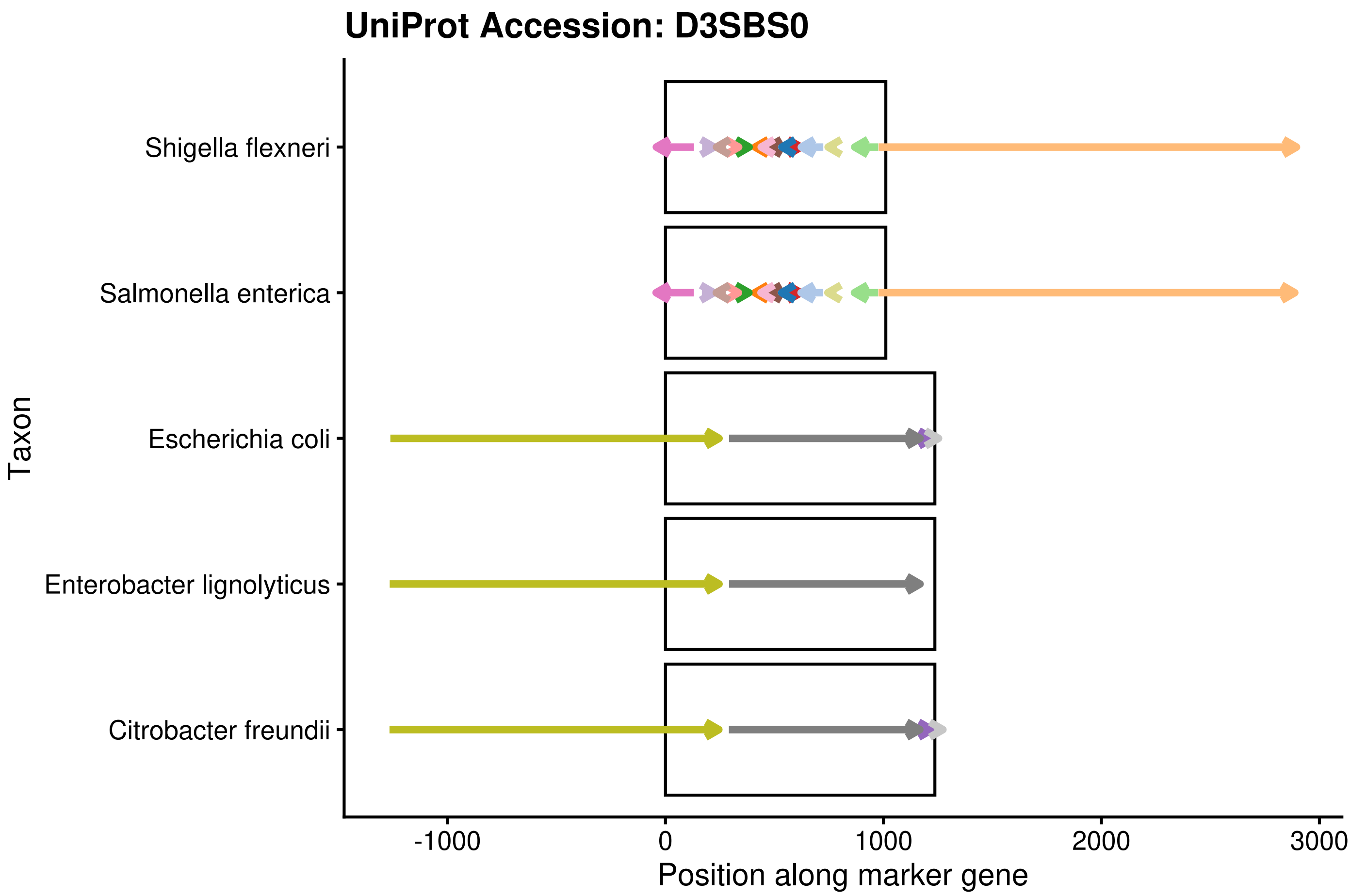
UniProt Accession: D3SBS0
Shigella flexneri
Salmonella enterica
Escherichia coli
Enterobacter lignolyticus
Citrobacter freundii
Taxon
-1000
0
1000
2000
3000
Position along marker gene

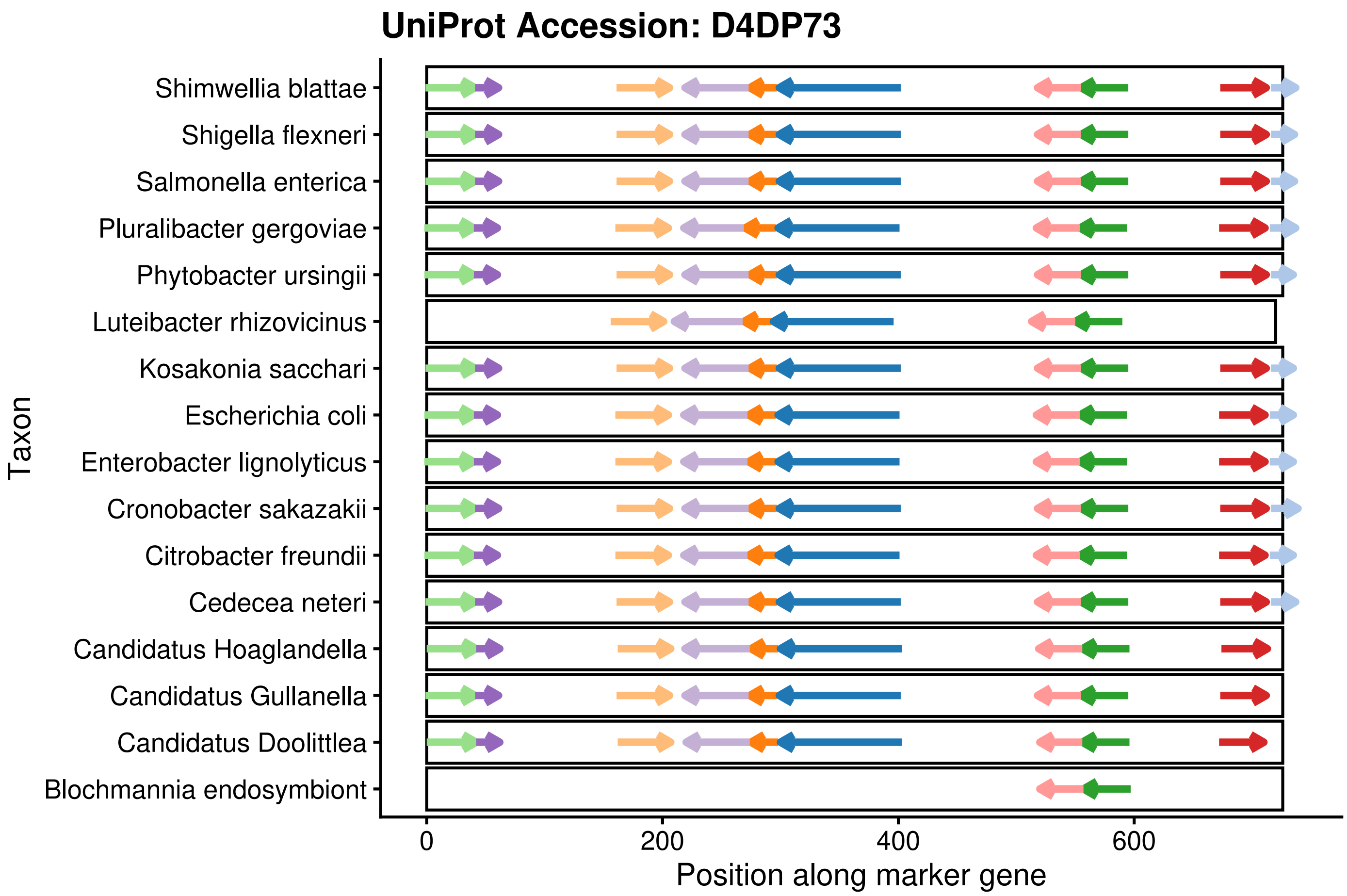
UniProt Accession: D4DP73
Shimwellia blattae
Shigella flexneri
Salmonella enterica
Pluralibacter gergoviae
Phytobacter ursingii
Luteibacter rhizovicinus
Kosakonia sacchari
Escherichia coli
Enterobacter lignolyticus
Cronobacter sakazakii
Citrobacter freundii
Cedecea neteri
Candidatus Hoaglandella
Candidatus Gullanella
Candidatus Doolittlea
Blochmannia endosymbiont
Taxon
0
200
400
600
Position along marker gene

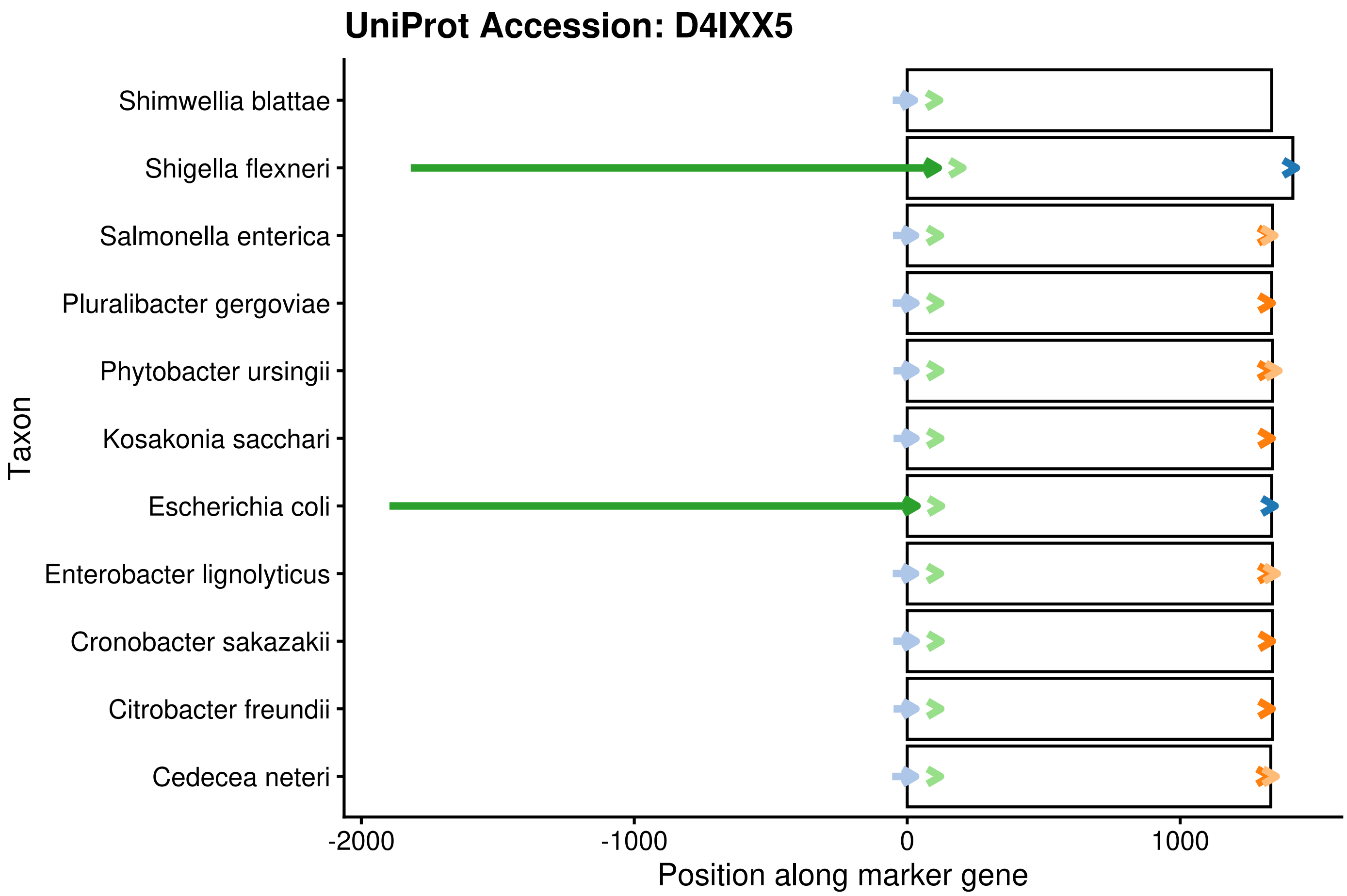

UniProt Accession: D4IXX5
Shimwellia blattae
Shigella flexneri
Salmonella enterica
Pluralibacter gergoviae
Phytobacter ursingii
Kosakonia sacchari
Escherichia coli
Enterobacter lignolyticus
Cronobacter sakazakii
Citrobacter freundii
Cedecea neteri
Taxon
-2000
-1000
0
1000
Position along marker gene

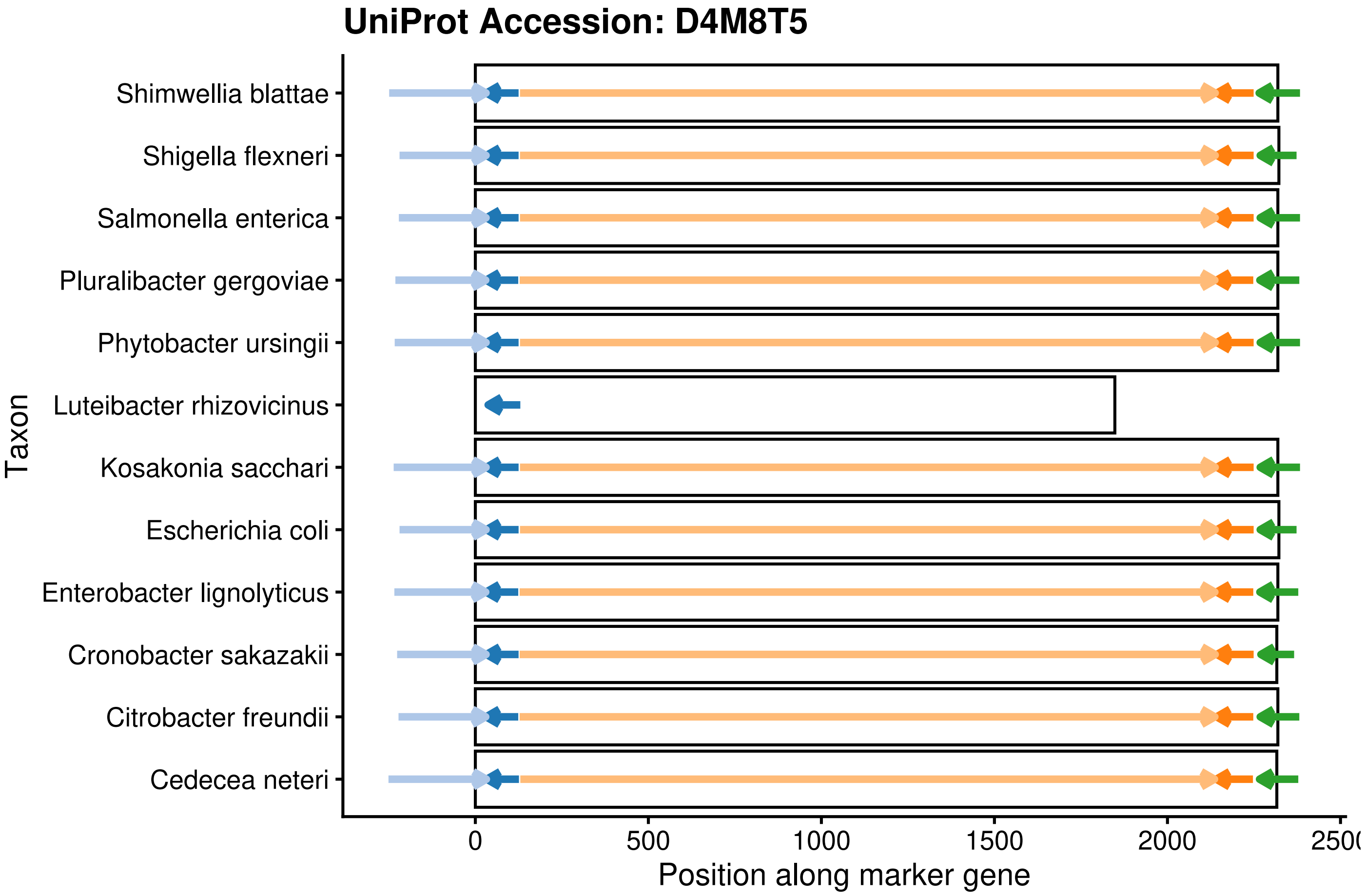

UniProt Accession: D4M8T5
Shimwellia blattae
Shigella flexneri
Salmonella enterica
Pluralibacter gergoviae
Phytobacter ursingii
Luteibacter rhizovicinus
Kosakonia sacchari
Escherichia coli
Enterobacter lignolyticus
Cronobacter sakazakii
Citrobacter freundii
Cedecea neteri
Taxon
0
500
1000
1500
2000
Position along marker gene

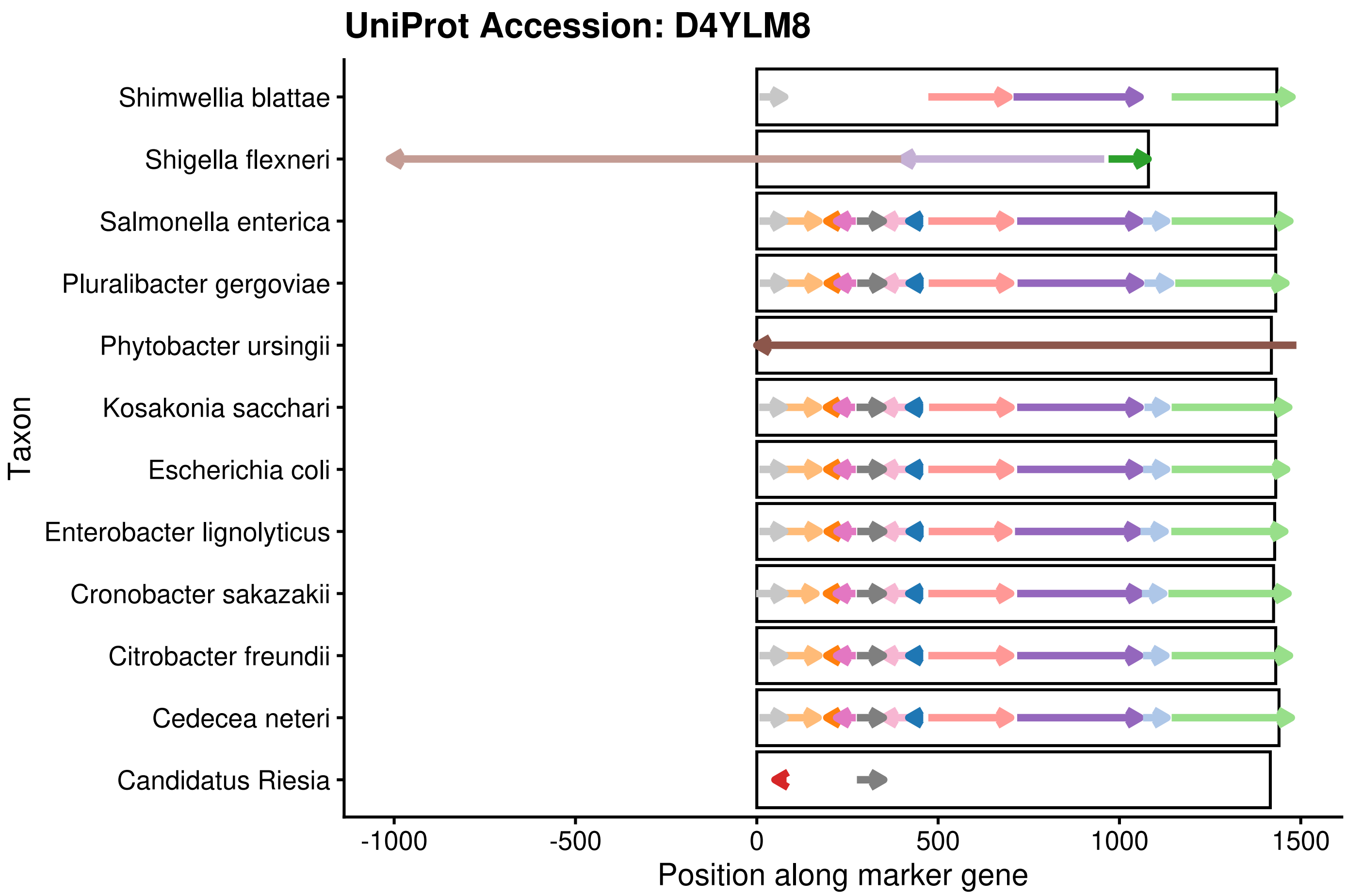
UniProt Accession: D4YLM8
Shimwellia blattae
Shigella flexneri
Salmonella enterica
Pluralibacter gergoviae
Phytobacter ursingii
Kosakonia sacchari
Escherichia coli
Enterobacter lignolyticus
Cronobacter sakazakii
Citrobacter freundii
Cedecea neteri
Candidatus Riesia
Taxon
-1000
-500
0
500
1000
1500
Position along marker gene

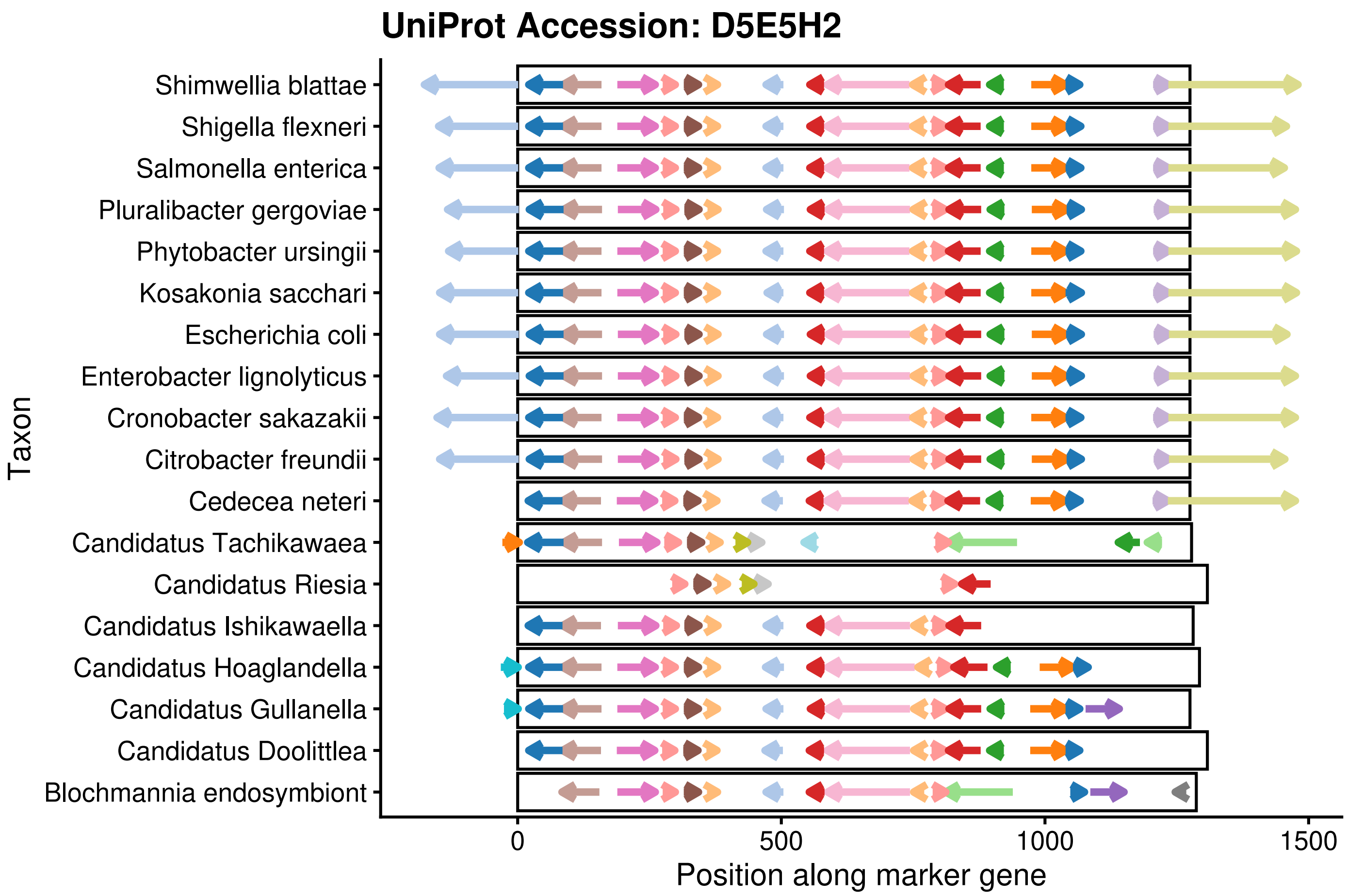
UniProt Accession: D5E5H2
Taxon
Shimwellia blattae
Shigella flexneri
Salmonella enterica
Pluralibacter gergoviae
Phytobacter ursingii
Kosakonia sacchari
Escherichia coli
Enterobacter lignolyticus
Cronobacter sakazakii
Citrobacter freundii
Cedecea neteri
Candidatus Tachikawaea
Candidatus Riesia
Candidatus Ishikawaella
Candidatus Hoaglandella
Candidatus Gullanella
Candidatus Doolittlea
Blochmannia endosymbiont
0
500
1000
1500
Position along marker gene

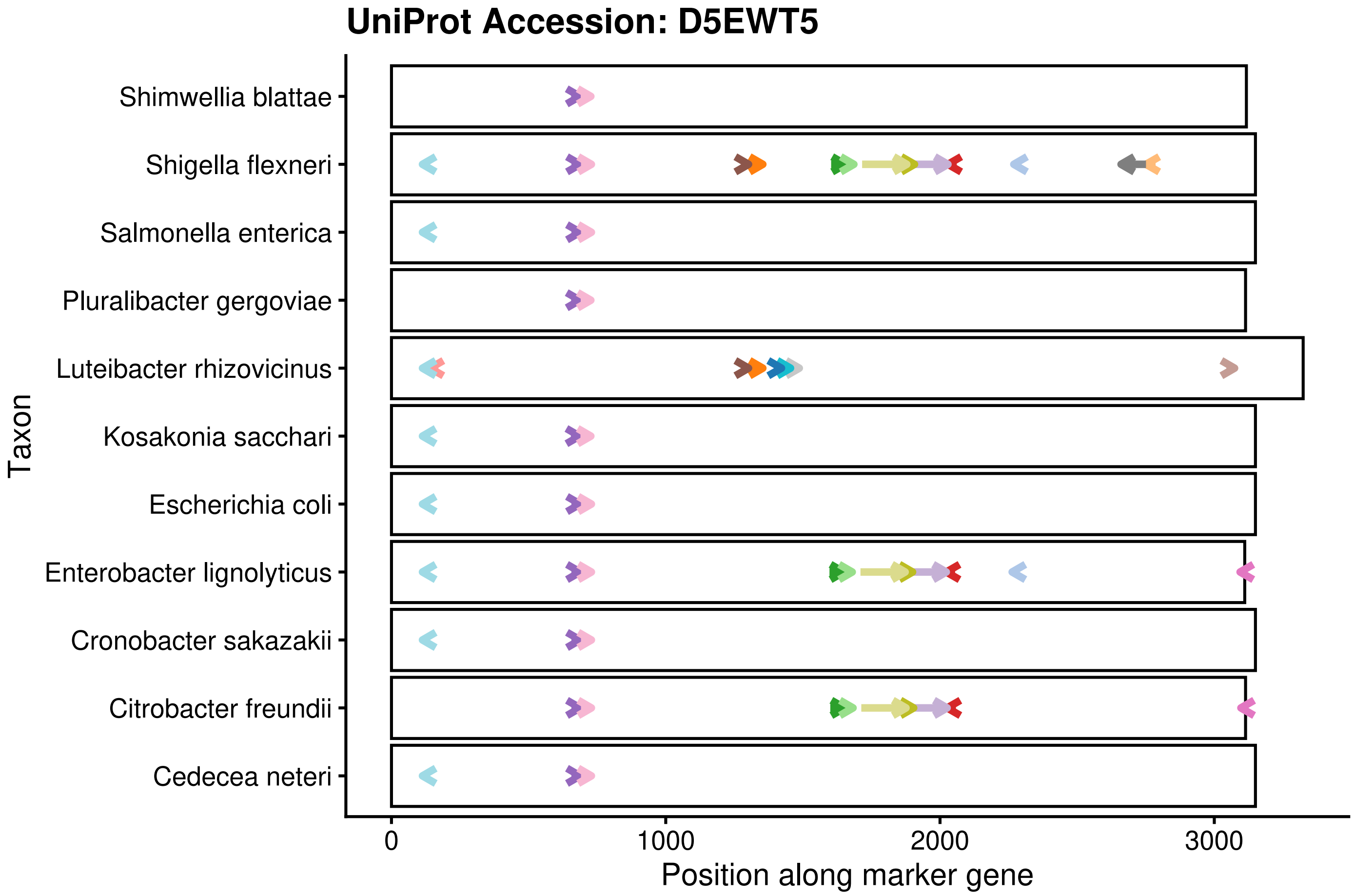
UniProt Accession: D5EWT5
Taxon
Shimwellia blattae
Shigella flexneri
Salmonella enterica
Pluralibacter gergoviae
Luteibacter rhizovicinus
Kosakonia sacchari
Escherichia coli
Enterobacter lignolyticus
Cronobacter sakazakii
Citrobacter freundii
Cedecea neteri
0
1000
2000
3000
Position along marker gene

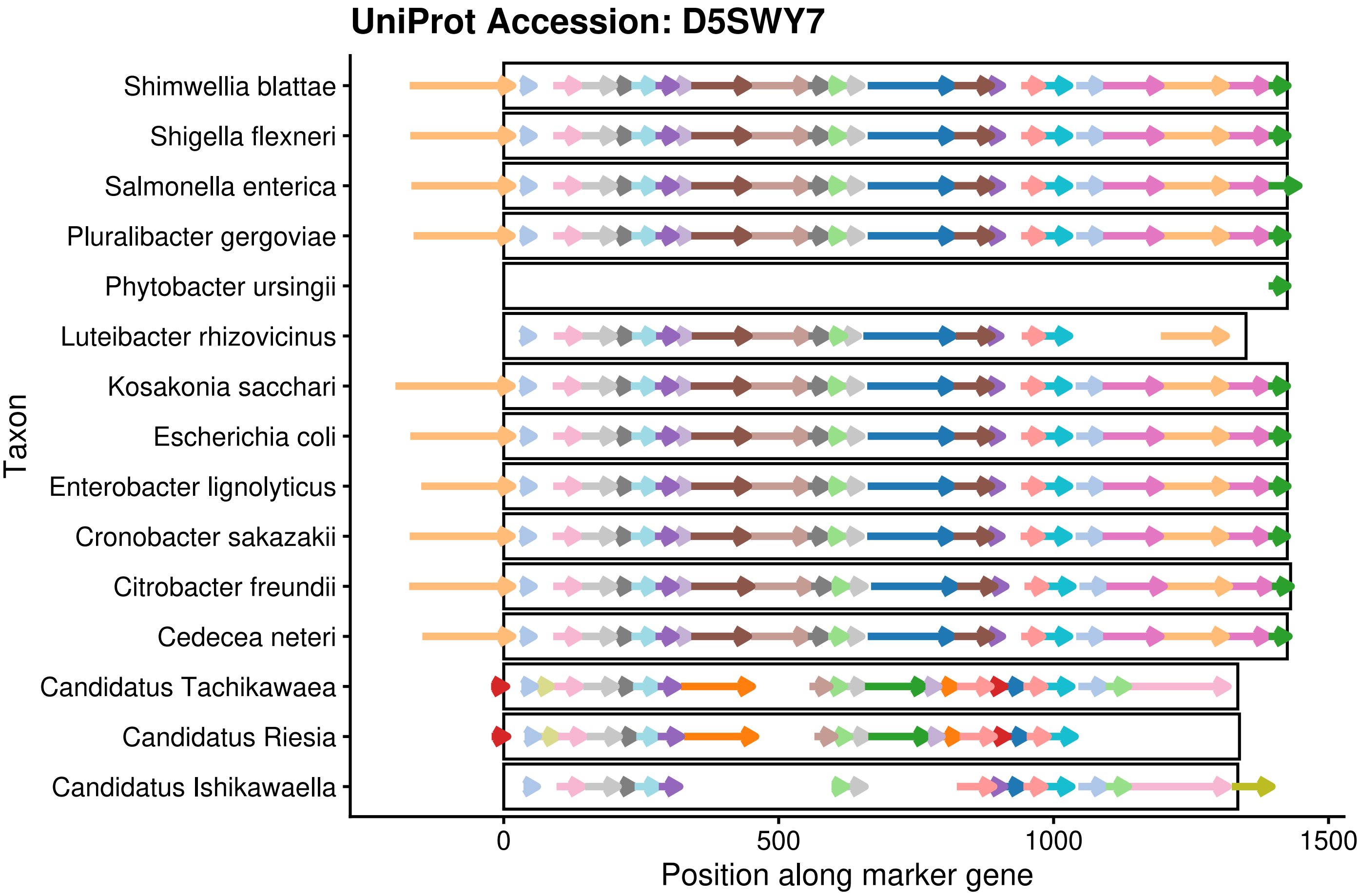
UniProt Accession: D5SWY7
Shimwellia blattae
Shigella flexneri
Salmonella enterica
Pluralibacter gergoviae
Phytobacter ursingii
Luteibacter rhizovicinus
Kosakonia sacchari
Escherichia coli
Enterobacter lignolyticus
Cronobacter sakazakii
Citrobacter freundii
Cedecea neteri
Candidatus Tachikawaea
Candidatus Riesia
Candidatus Ishikawaella
Taxon
0
500
1000
1500
Position along marker gene

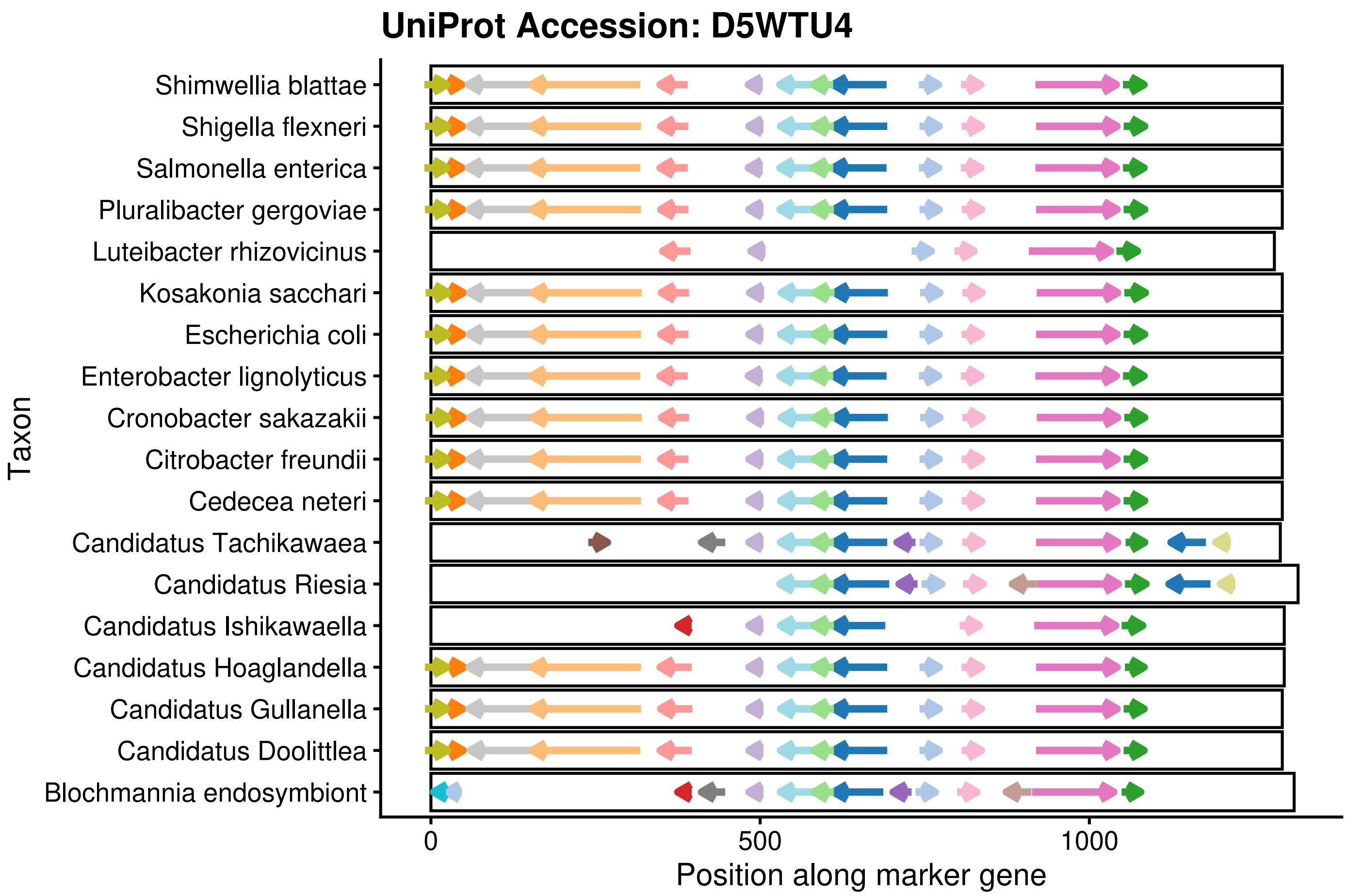

UniProt Accession: D5WTU4
Taxon
Shimwellia blattae
Shigella flexneri
Salmonella enterica
Pluralibacter gergoviae
Luteibacter rhizovicinus
Kosakonia sacchari
Escherichia coli
Enterobacter lignolyticus
Cronobacter sakazakii
Citrobacter freundii
Cedecea neteri
Candidatus Tachikawaea
Candidatus Riesia
Candidatus Ishikawaella
Candidatus Hoaglandella
Candidatus Gullanella
Candidatus Doolittlea
Blochmannia endosymbiont
0
500
1000
Position along marker gene

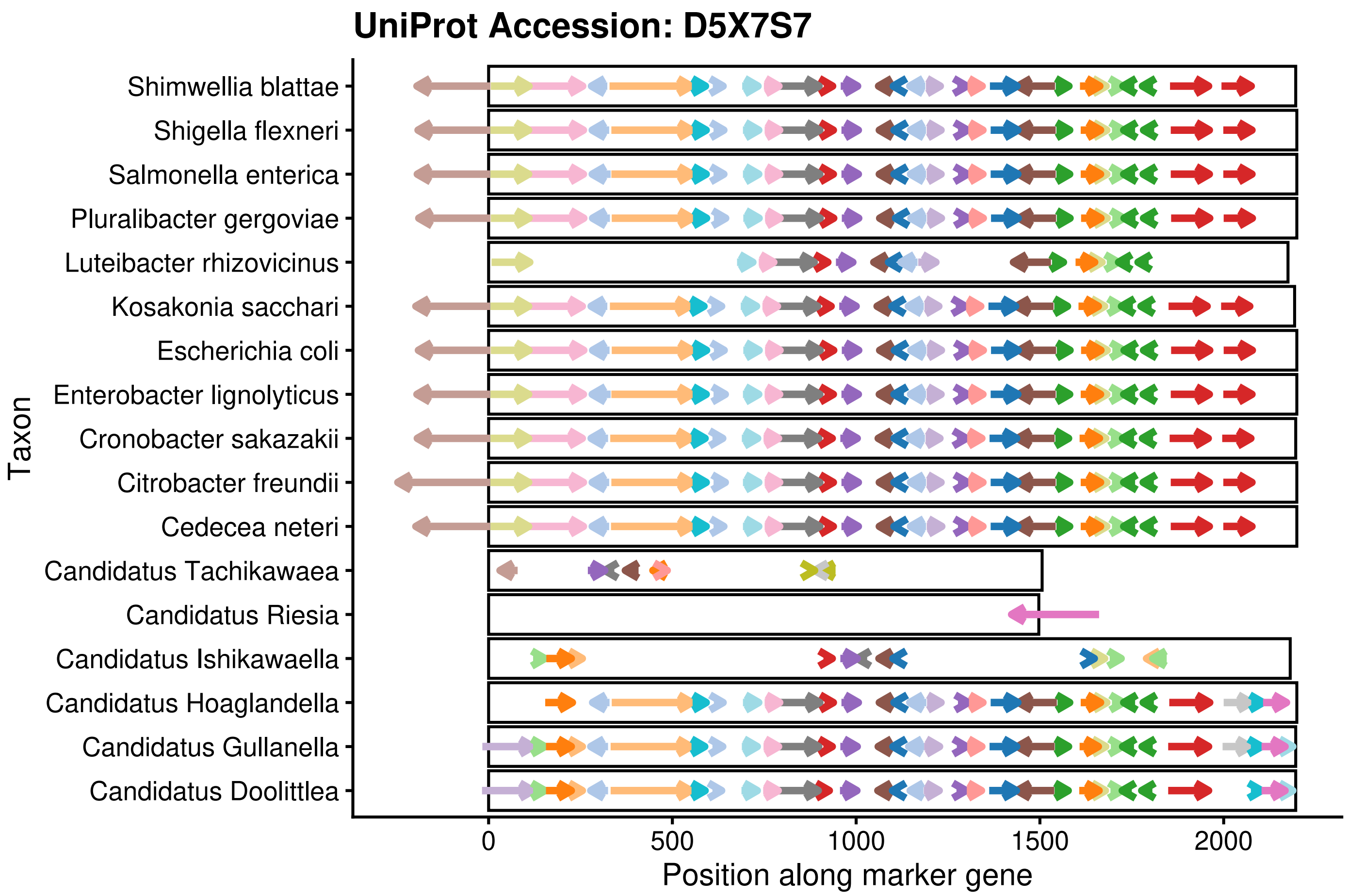

UniProt Accession: D5X7S7
Shimwellia blattae
Shigella flexneri
Salmonella enterica
Pluralibacter gergoviae
Luteibacter rhizovicinus
Kosakonia sacchari
Escherichia coli
Enterobacter lignolyticus
Cronobacter sakazakii
Citrobacter freundii
Cedecea neteri
Candidatus Tachikawaea
Candidatus Riesia
Candidatus Ishikawaella
Candidatus Hoaglandella
Candidatus Gullanella
Candidatus Doolittlea
Taxon
0
500
1000
1500
2000
Position along marker gene

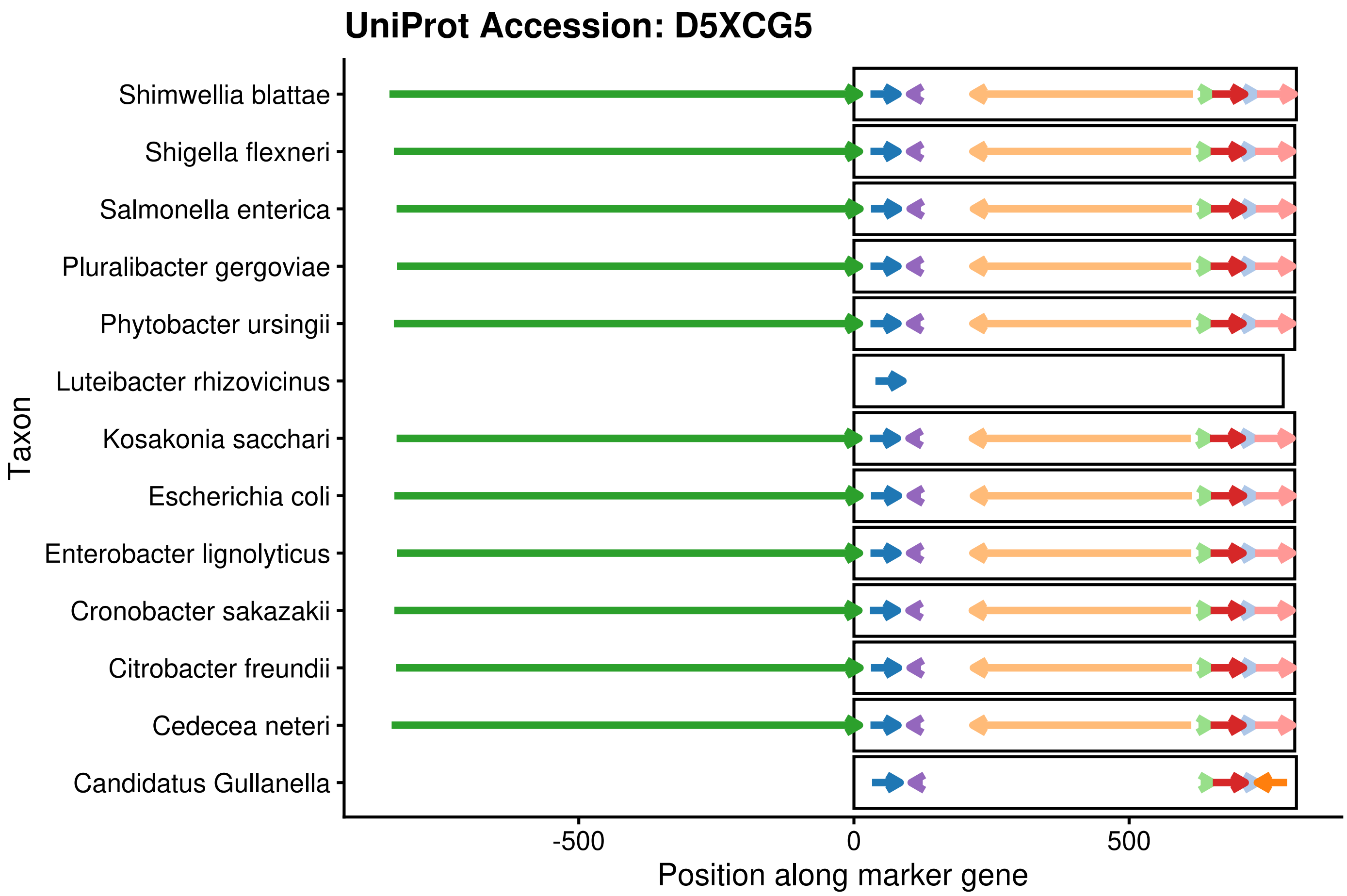

UniProt Accession: D5XCG5
Shimwellia blattae
Shigella flexneri
Salmonella enterica
Pluralibacter gergoviae
Phytobacter ursingii
Luteibacter rhizovicinus
Kosakonia sacchari
Escherichia coli
Enterobacter lignolyticus
Cronobacter sakazakii
Citrobacter freundii
Cedecea neteri
Candidatus Gullanella
Taxon
-500
0
500
Position along marker gene

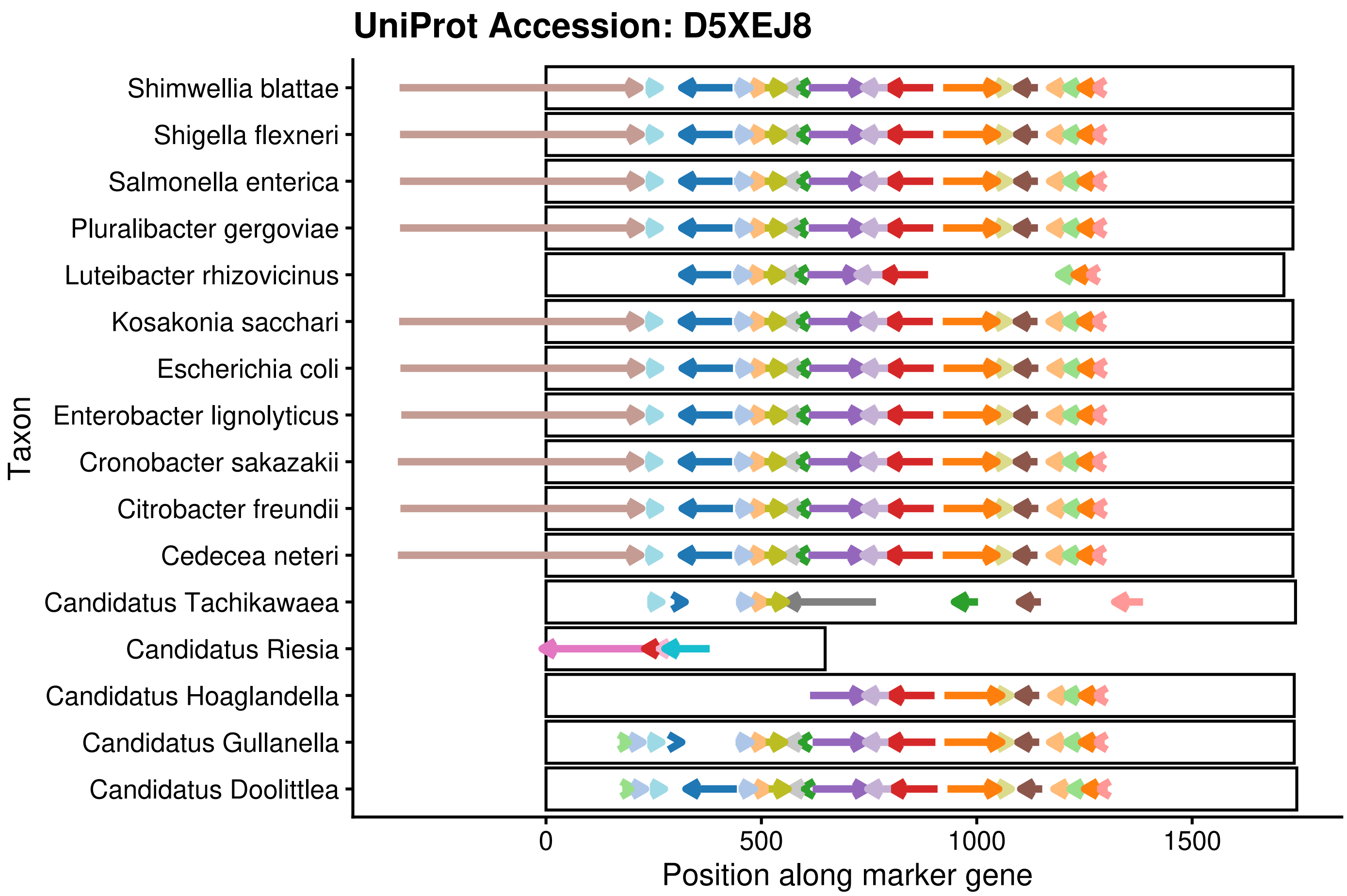

UniProt Accession: D5XEJ8
Taxon
Shimwellia blattae
Shigella flexneri
Salmonella enterica
Pluralibacter gergoviae
Luteibacter rhizovicinus
Kosakonia sacchari
Escherichia coli
Enterobacter lignolyticus
Cronobacter sakazakii
Citrobacter freundii
Cedecea neteri
Candidatus Tachikawaea
Candidatus Riesia
Candidatus Hoaglandella
Candidatus Gullanella
Candidatus Doolittlea
0
500
1000
1500
Position along marker gene

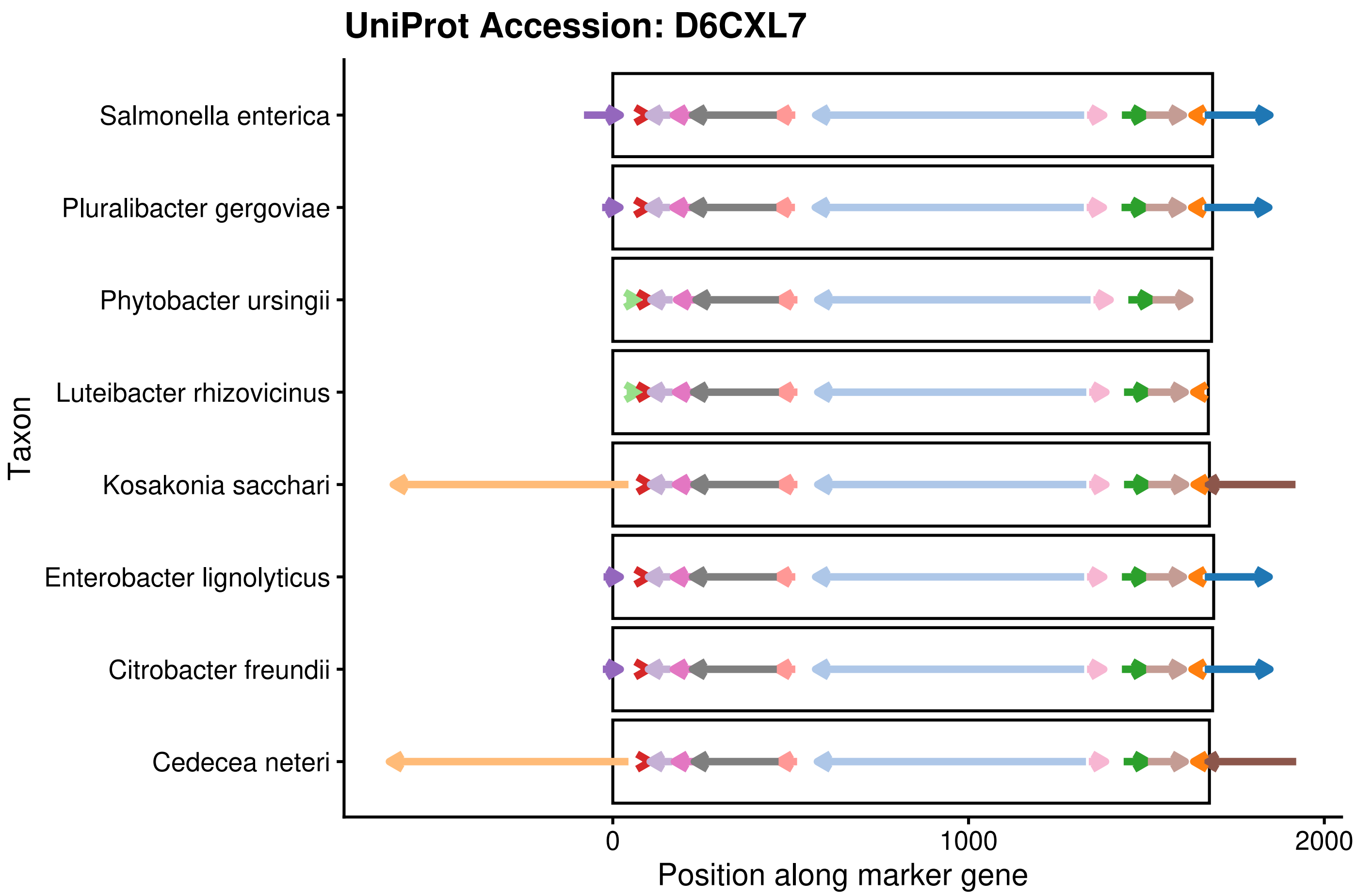
UniProt Accession: D6CXL7
Taxon
Salmonella enterica
Pluralibacter gergoviae
Phytobacter ursingii
Luteibacter rhizovicinus
Kosakonia sacchari
Enterobacter lignolyticus
Citrobacter freundii
Cedecea neteri
0
1000
2000
Position along marker gene

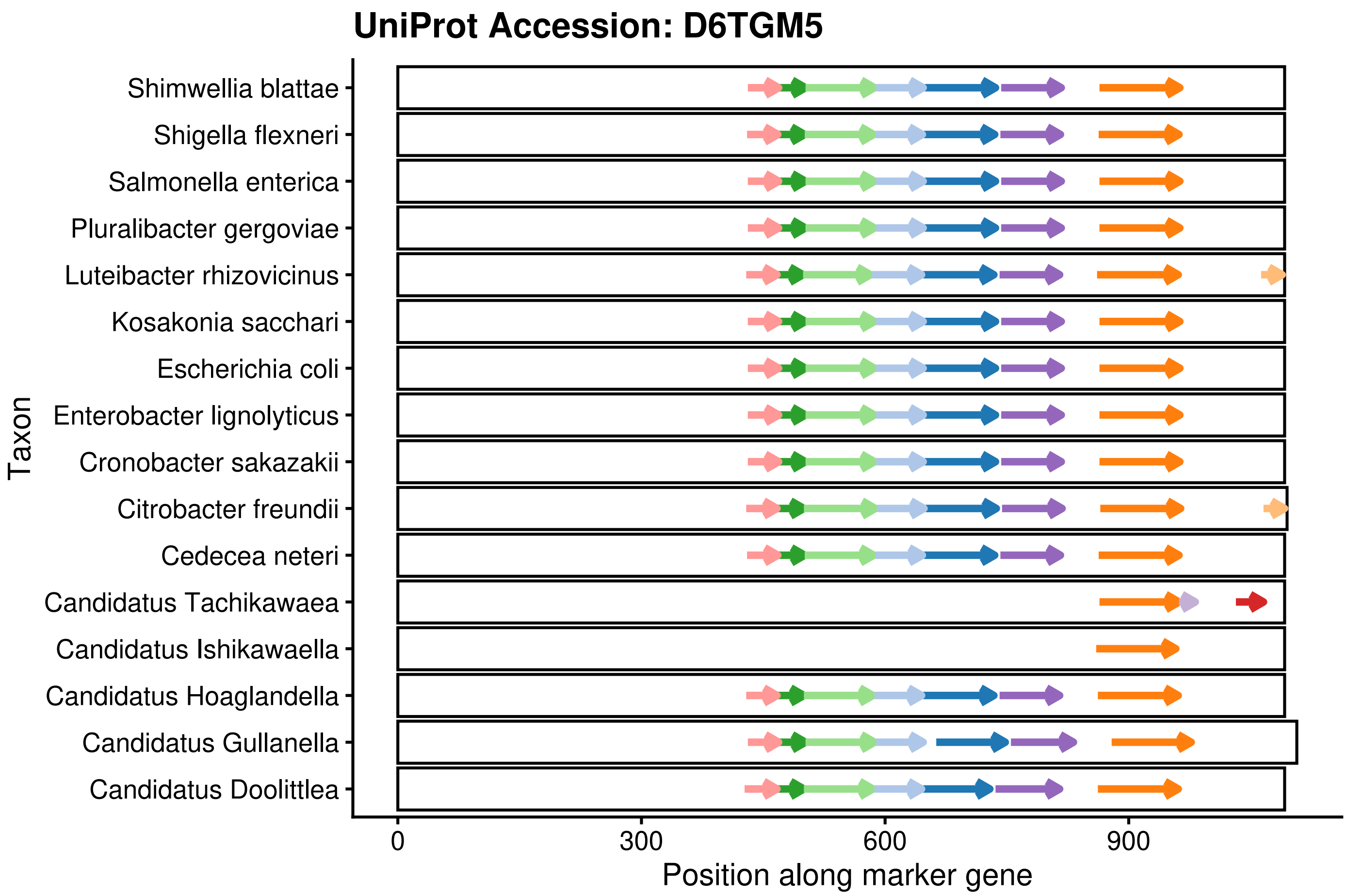
UniProt Accession: D6TGM5
Shimwellia blattae
Shigella flexneri
Salmonella enterica
Pluralibacter gergoviae
Luteibacter rhizovicinus
Kosakonia sacchari
Escherichia coli
Enterobacter lignolyticus
Cronobacter sakazakii
Citrobacter freundii
Cedecea neteri
Candidatus Tachikawaea
Candidatus Ishikawaella
Candidatus Hoaglandella
Candidatus Gullanella
Candidatus Doolittlea
Taxon
0
300
600
900
Position along marker gene

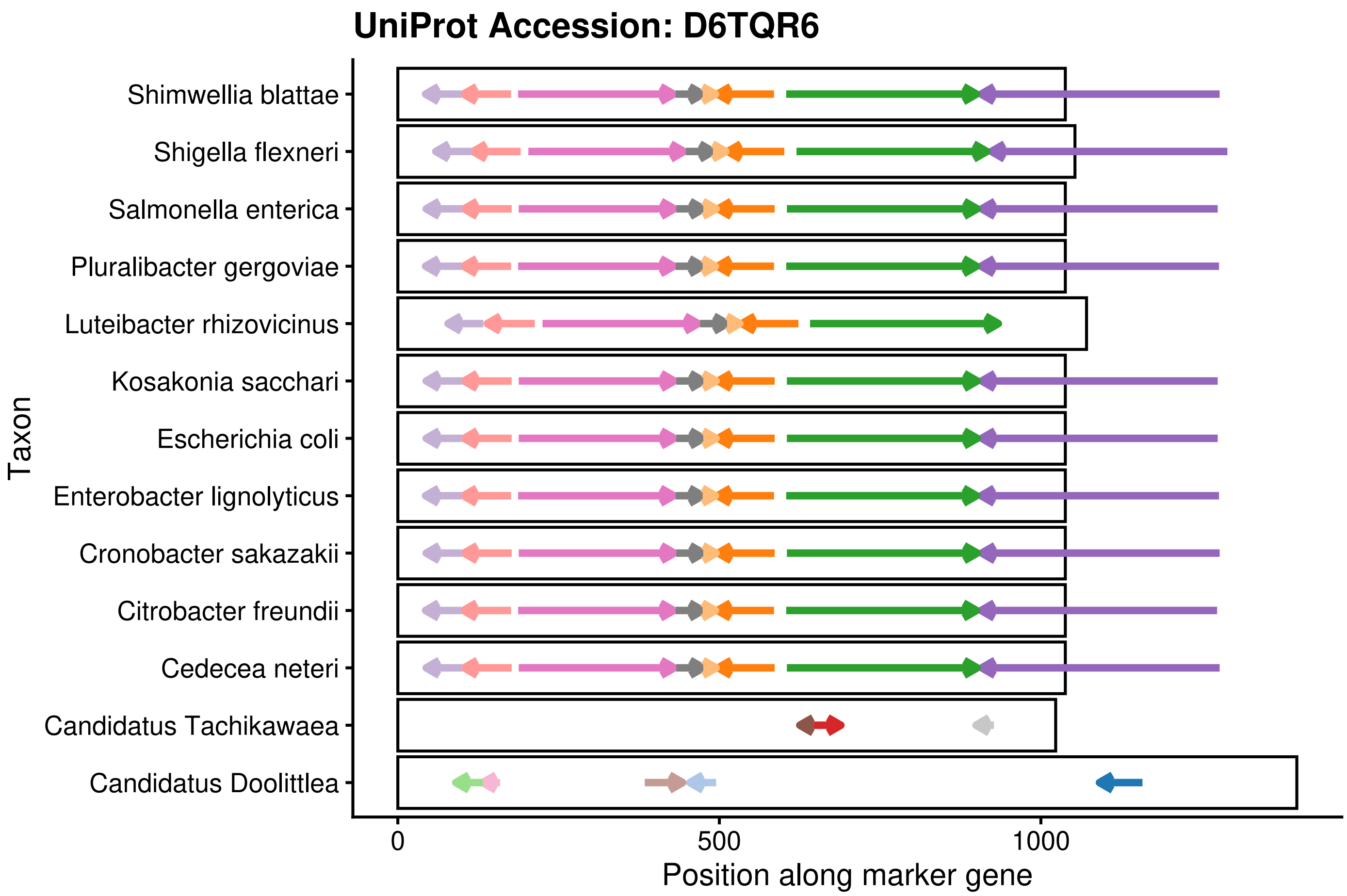

UniProt Accession: D6TQR6
Taxon
Shimwellia blattae
Shigella flexneri
Salmonella enterica
Pluralibacter gergoviae
Luteibacter rhizovicinus
Kosakonia sacchari
Escherichia coli
Enterobacter lignolyticus
Cronobacter sakazakii
Citrobacter freundii
Cedecea neteri
Candidatus Tachikawaea
Candidatus Doolittlea
0
500
1000
Position along marker gene

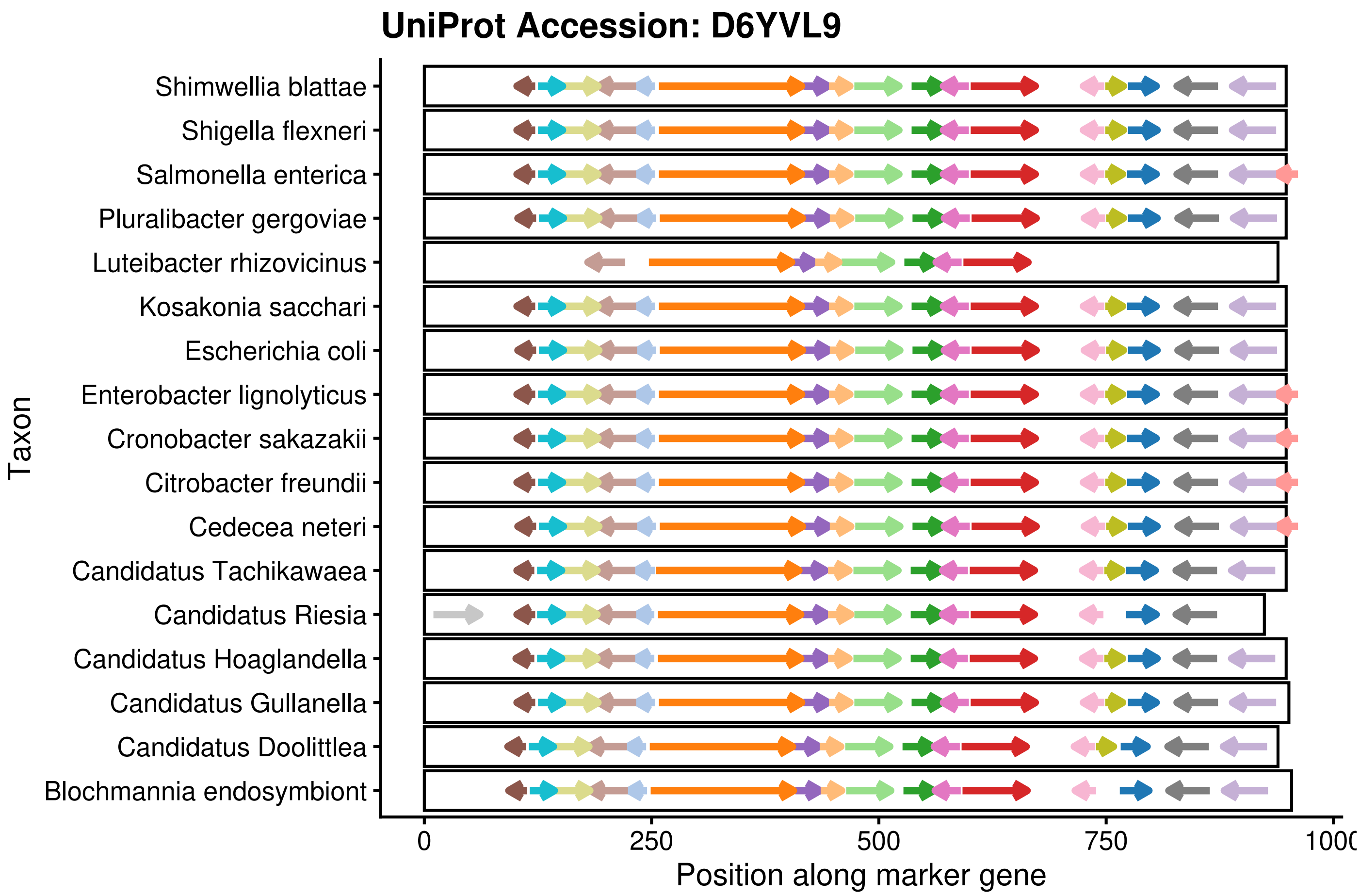

UniProt Accession: D6YVL9
Shimwellia blattae
Shigella flexneri
Salmonella enterica
Pluralibacter gergoviae
Luteibacter rhizovicinus
Kosakonia sacchari
Escherichia coli
Enterobacter lignolyticus
Cronobacter sakazakii
Citrobacter freundii
Cedecea neteri
Candidatus Tachikawaea
Candidatus Riesia
Candidatus Hoaglandella
Candidatus Gullanella
Candidatus Doolittlea
Blochmannia endosymbiont
Taxon
0
250
500
750
1000
Position along marker gene

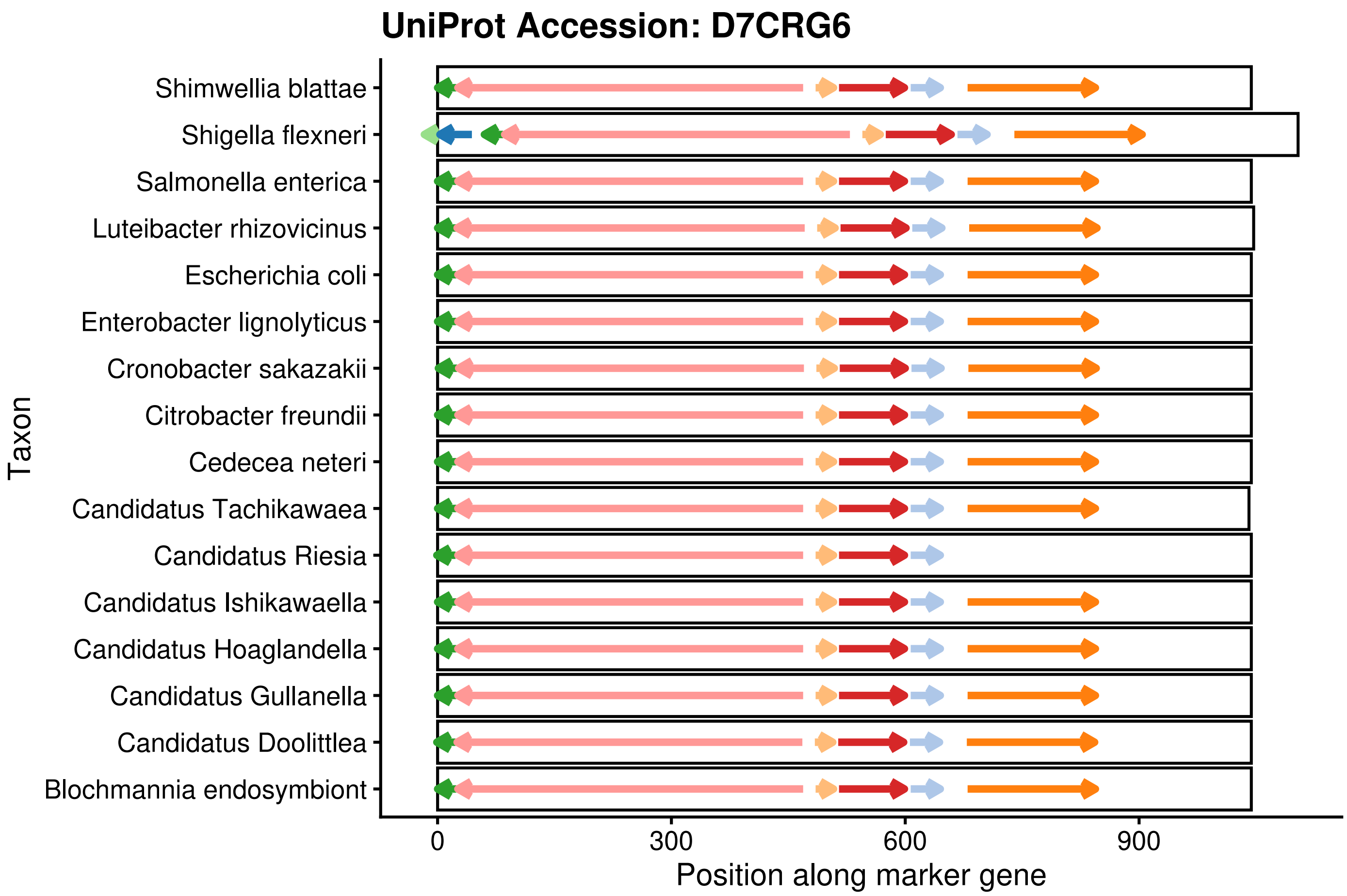

UniProt Accession: D7CRG6
Shimwellia blattae
Shigella flexneri
Salmonella enterica
Luteibacter rhizovicinus
Escherichia coli
Enterobacter lignolyticus
Cronobacter sakazakii
Citrobacter freundii
Cedecea neteri
Candidatus Tachikawaea
Candidatus Riesia
Candidatus Ishikawaella
Candidatus Hoaglandella
Candidatus Gullanella
Candidatus Doolittlea
Blochmannia endosymbiont
Taxon
0
300
600
900
Position along marker gene

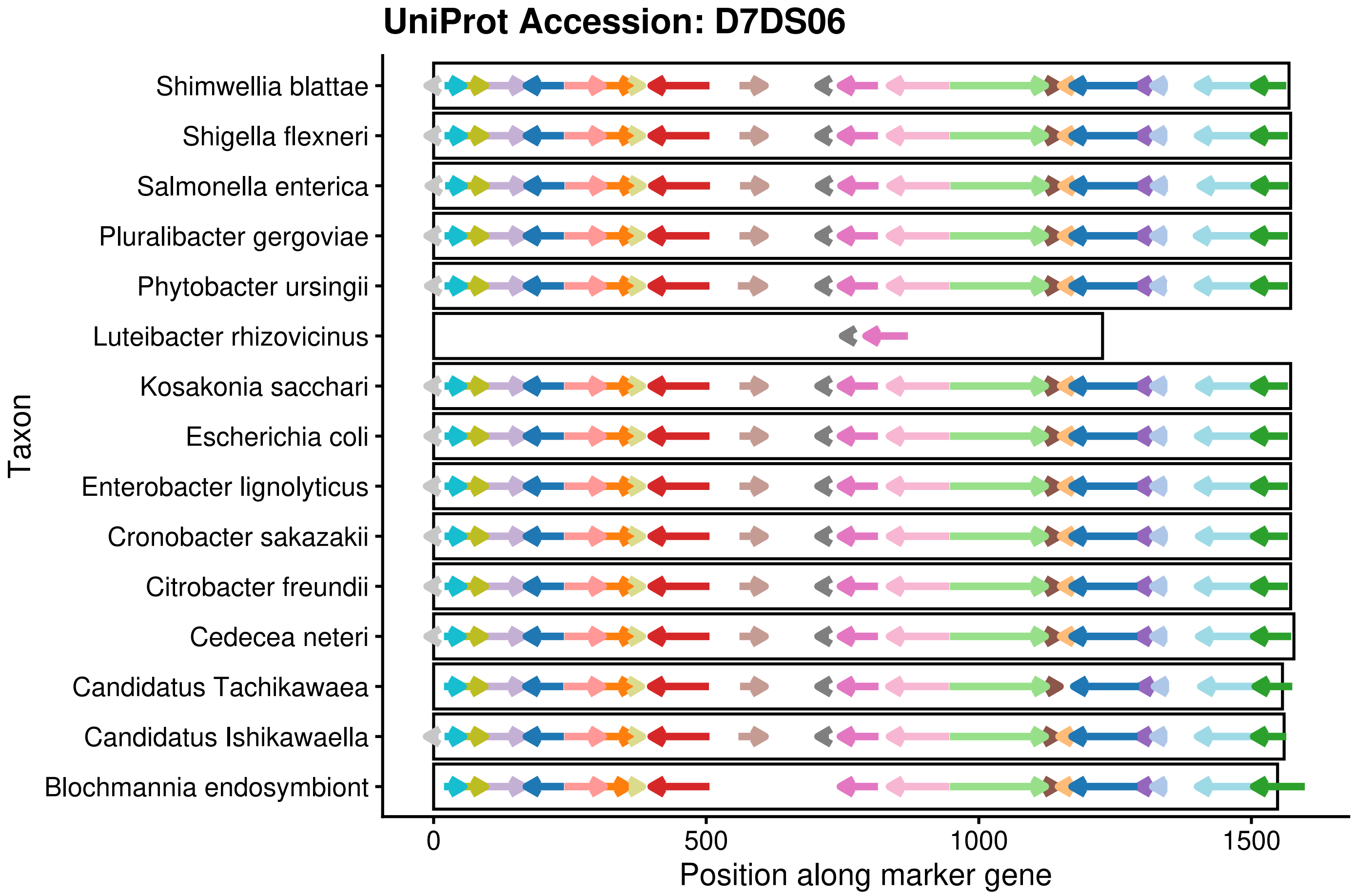
UniProt Accession: D7DS06
Taxon
Shimwellia blattae
Shigella flexneri
Salmonella enterica
Pluralibacter gergoviae
Phytobacter ursingii
Luteibacter rhizovicinus
Kosakonia sacchari
Escherichia coli
Enterobacter lignolyticus
Cronobacter sakazakii
Citrobacter freundii
Cedecea neteri
Candidatus Tachikawaea
Candidatus Ishikawaella
Blochmannia endosymbiont
0
500
1000
1500
Position along marker gene

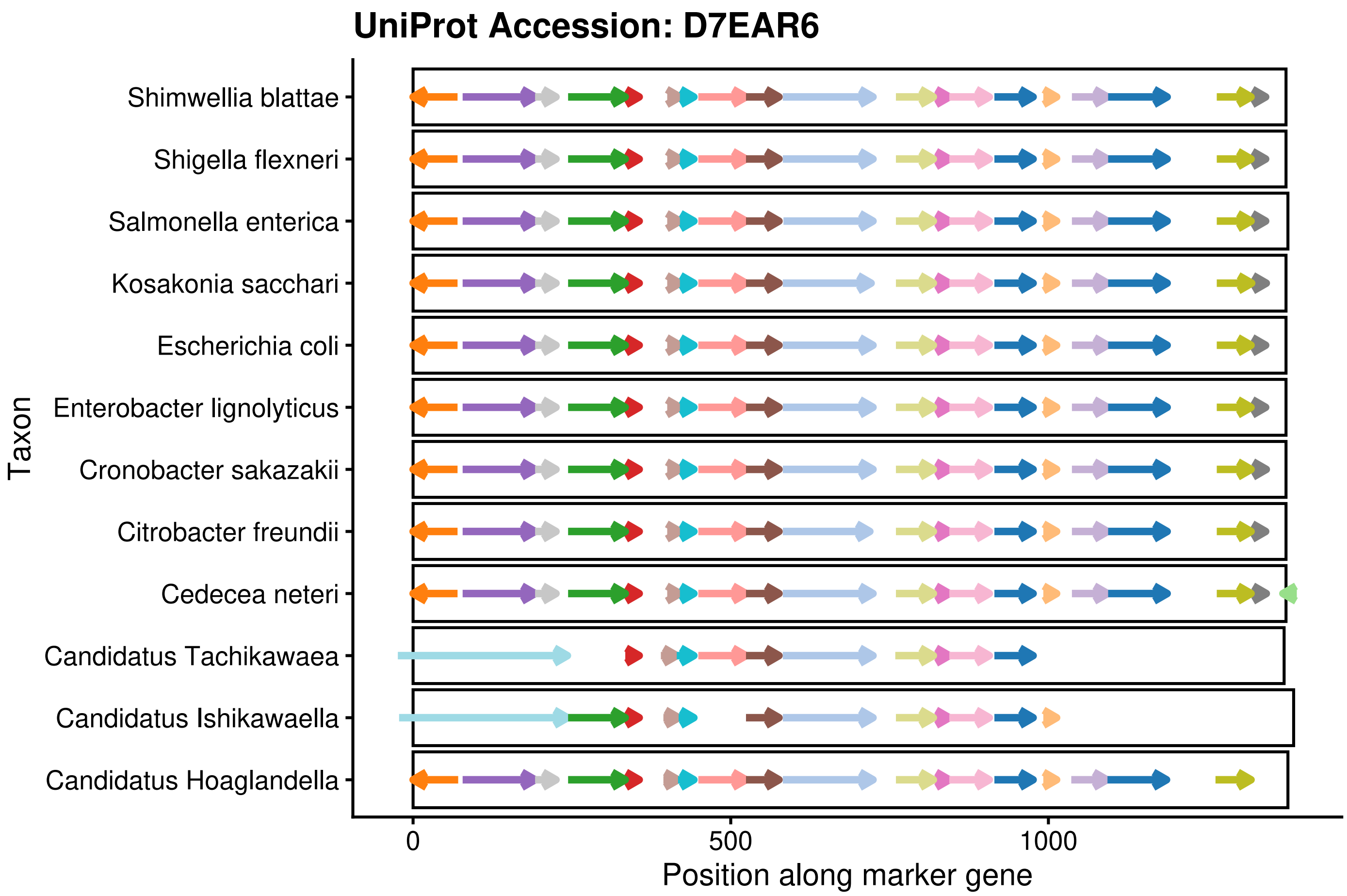
UniProt Accession: D7EAR6
Shimwellia blattae
Shigella flexneri
Salmonella enterica
Kosakonia sacchari
Escherichia coli
Enterobacter lignolyticus
Cronobacter sakazakii
Citrobacter freundii
Cedecea neteri
Candidatus Tachikawaea
Candidatus Ishikawaella
Candidatus Hoaglandella
Taxon
0
500
1000
Position along marker gene

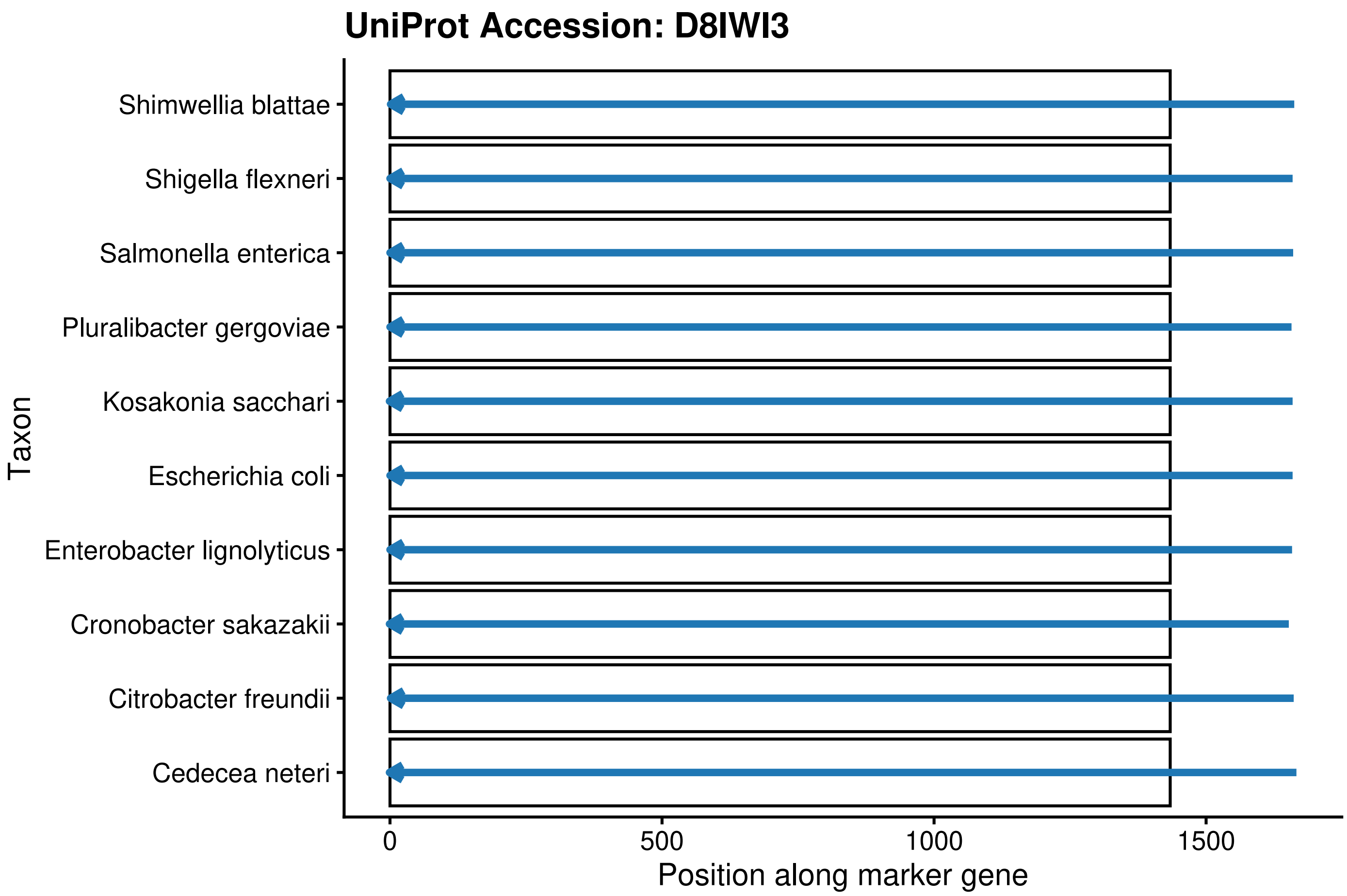
UniProt Accession: D8IWI3
Shimwellia blattae
Shigella flexneri
Salmonella enterica
Pluralibacter gergoviae
Kosakonia sacchari
Escherichia coli
Enterobacter lignolyticus
Cronobacter sakazakii
Citrobacter freundii
Cedecea neteri
Taxon
0
500
1000
1500
Position along marker gene

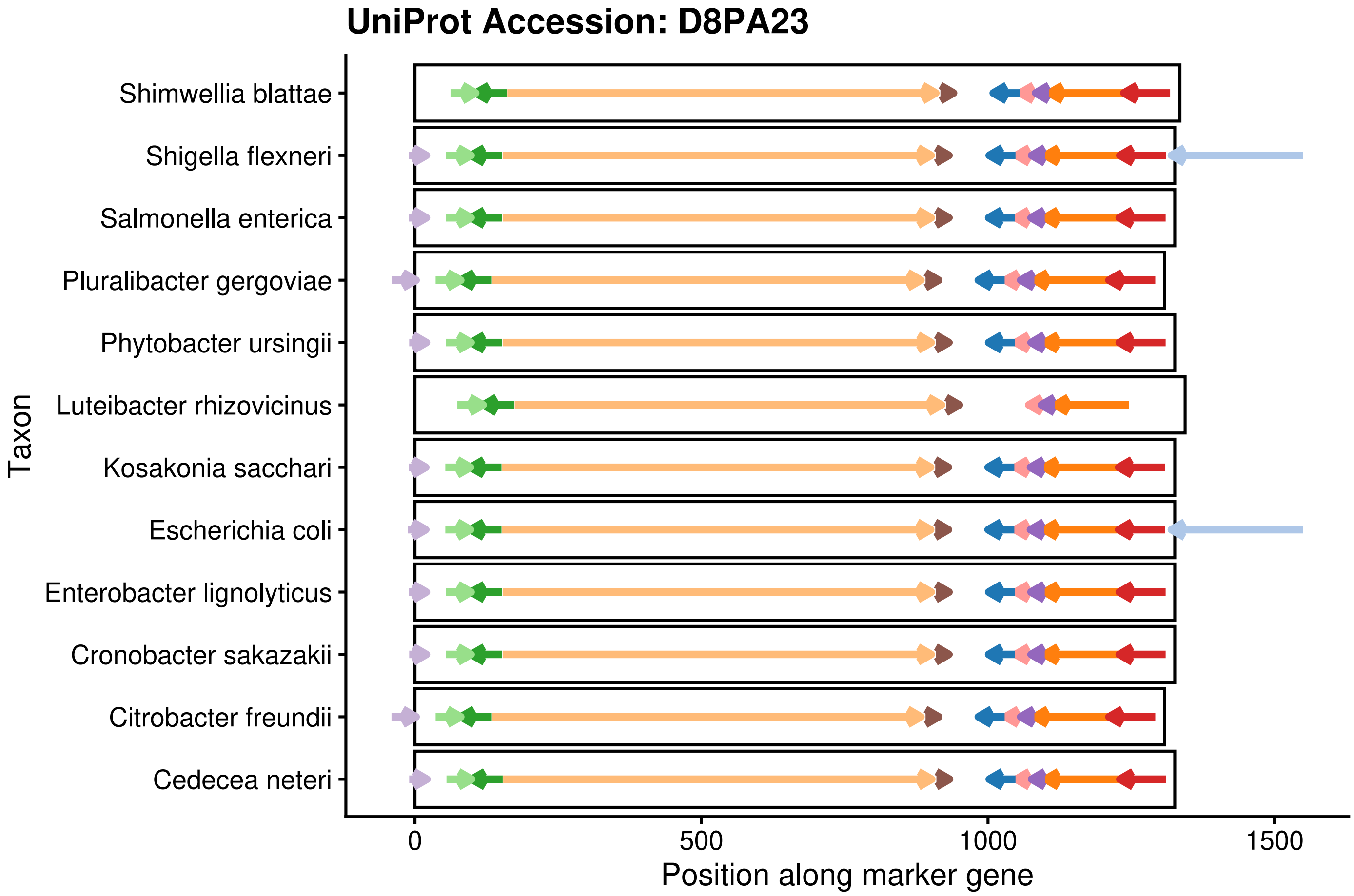

UniProt Accession: D8PA23
Shimwellia blattae
Shigella flexneri
Salmonella enterica
Pluralibacter gergoviae
Phytobacter ursingii
Luteibacter rhizovicinus
Kosakonia sacchari
Escherichia coli
Enterobacter lignolyticus
Cronobacter sakazakii
Citrobacter freundii
Cedecea neteri
Taxon
0
500
1000
1500
Position along marker gene

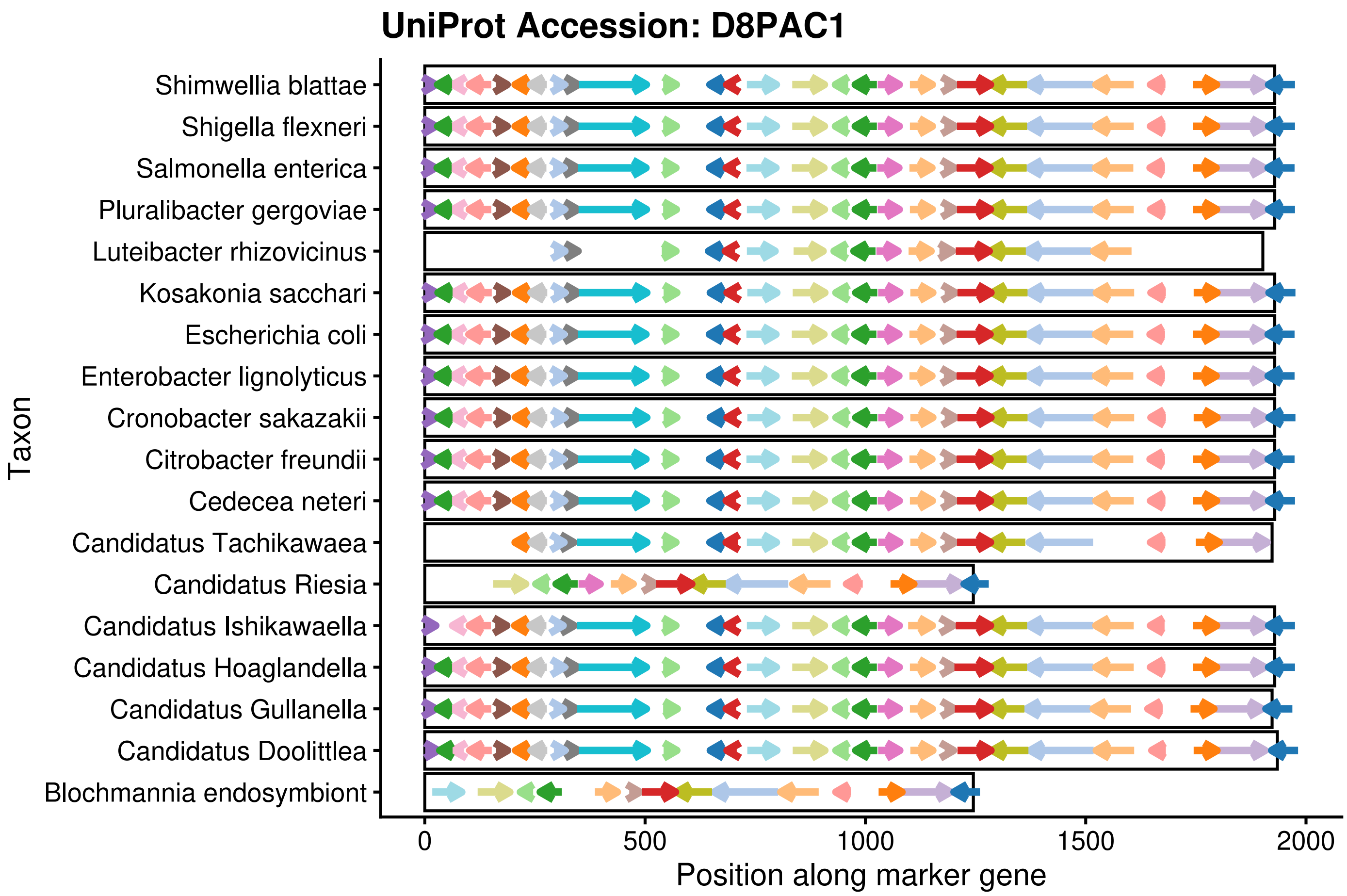
UniProt Accession: D8PAC1
Taxon
Shimwellia blattae
Shigella flexneri
Salmonella enterica
Pluralibacter gergoviae
Luteibacter rhizovicinus
Kosakonia sacchari
Escherichia coli
Enterobacter lignolyticus
Cronobacter sakazakii
Citrobacter freundii
Cedecea neteri
Candidatus Tachikawaea
Candidatus Riesia
Candidatus Ishikawaella
Candidatus Hoaglandella
Candidatus Gullanella
Candidatus Doolittlea
Blochmannia endosymbiont
0
500
1000
1500
2000
Position along marker gene

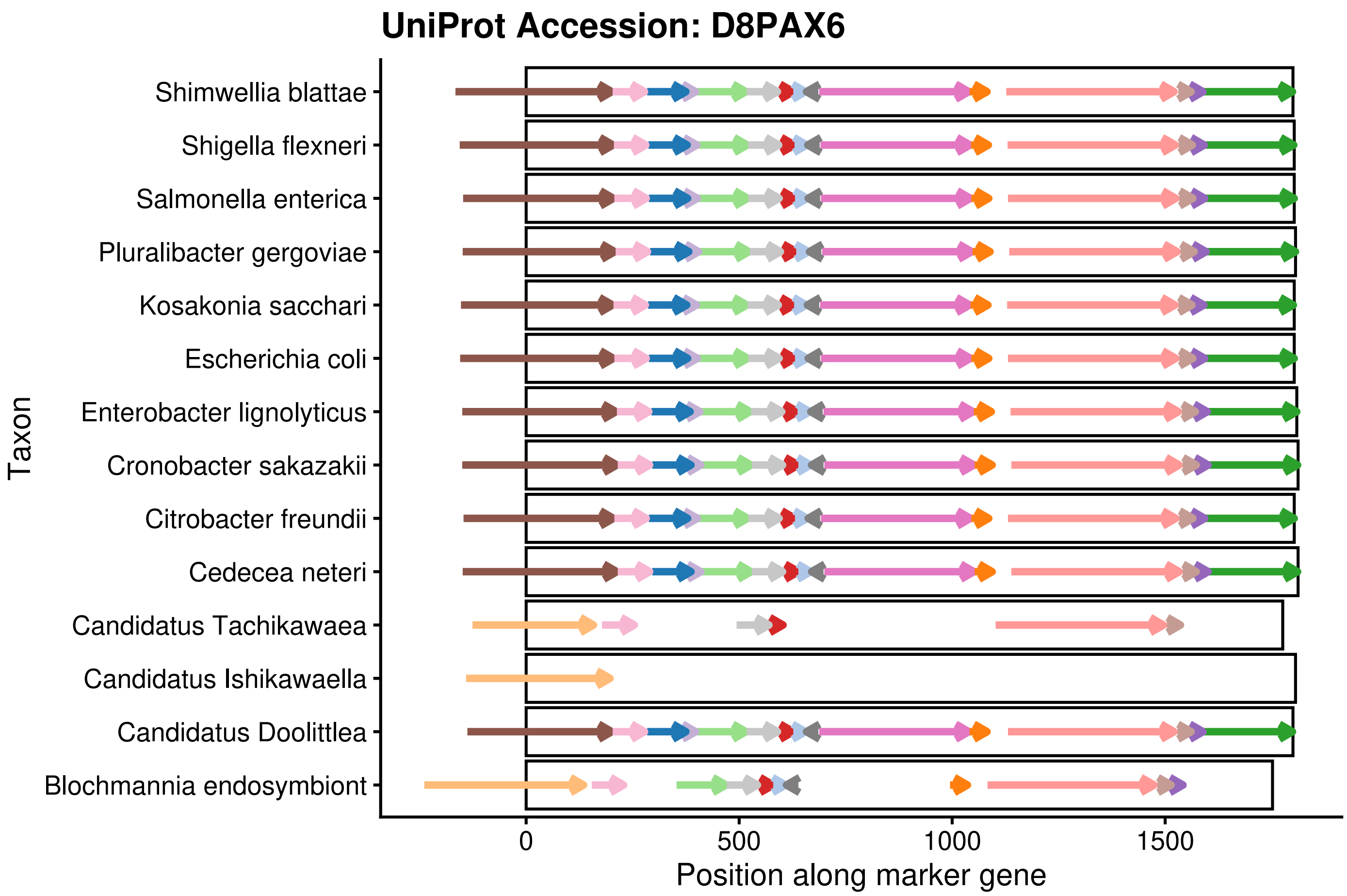
UniProt Accession: D8PAX6
Taxon
Shimwellia blattae
Shigella flexneri
Salmonella enterica
Pluralibacter gergoviae
Kosakonia sacchari
Escherichia coli
Enterobacter lignolyticus
Cronobacter sakazakii
Citrobacter freundii
Cedecea neteri
Candidatus Tachikawaea
Candidatus Ishikawaella
Candidatus Doolittlea
Blochmannia endosymbiont
0
500
1000
1500
Position along marker gene

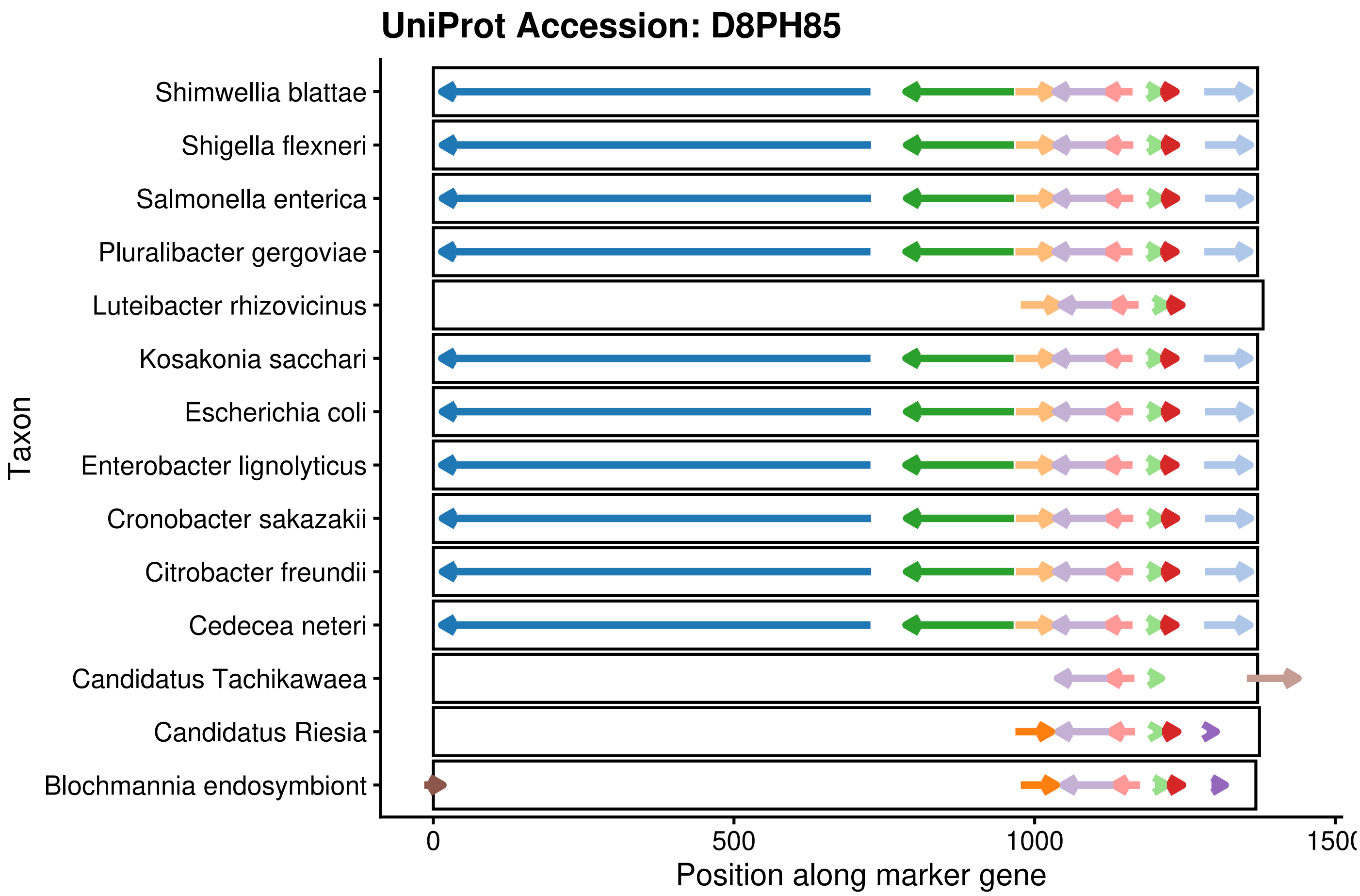

UniProt Accession: D8PH85
Taxon
Shimwellia blattae
Shigella flexneri
Salmonella enterica
Pluralibacter gergoviae
Luteibacter rhizovicinus
Kosakonia sacchari
Escherichia coli
Enterobacter lignolyticus
Cronobacter sakazakii
Citrobacter freundii
Cedecea neteri
Candidatus Tachikawaea
Candidatus Riesia
Blochmannia endosymbiont
0
500
1000
Position along marker gene

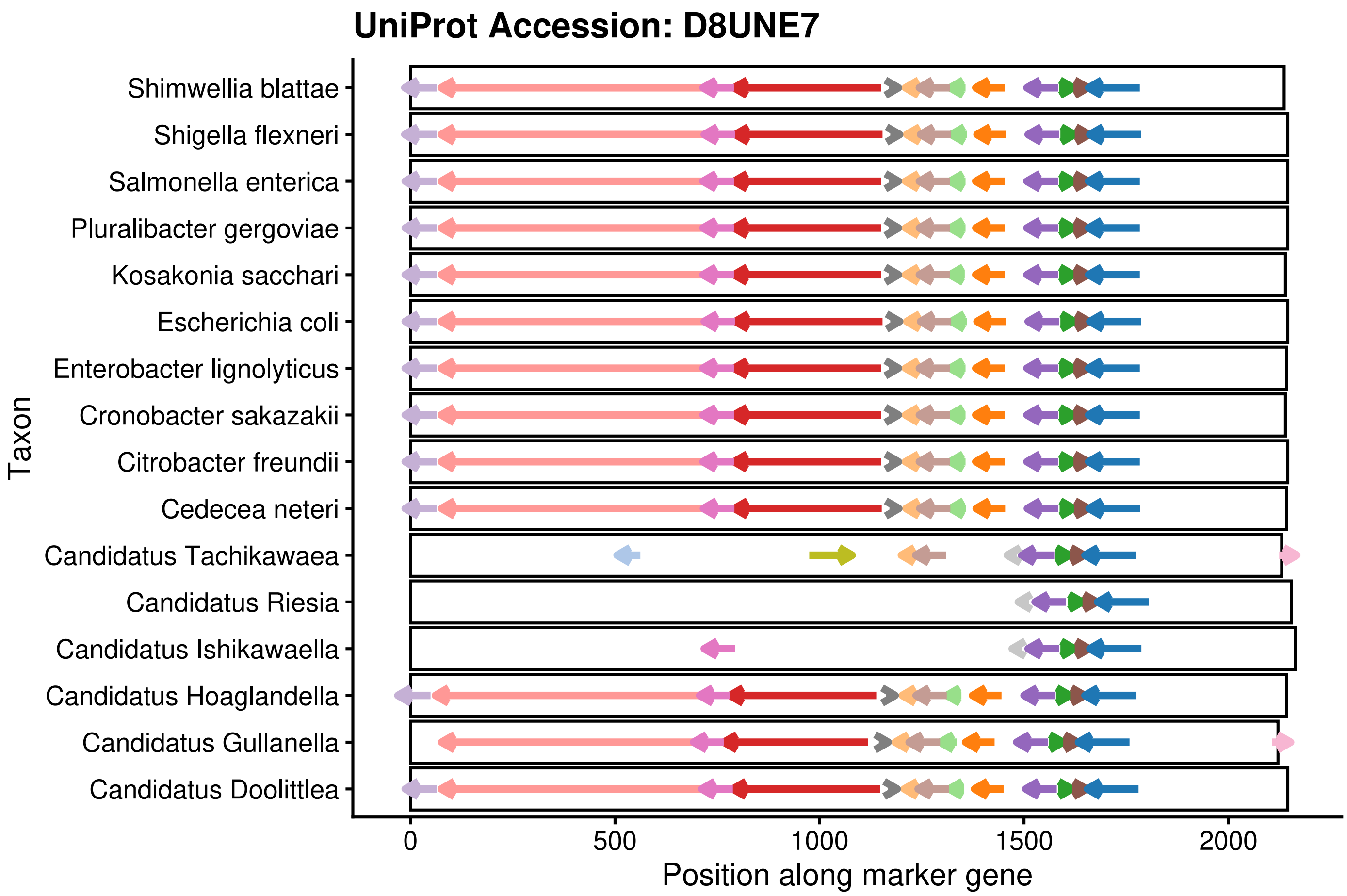
UniProt Accession: D8UNE7
Taxon
Shimwellia blattae
Shigella flexneri
Salmonella enterica
Pluralibacter gergoviae
Kosakonia sacchari
Escherichia coli
Enterobacter lignolyticus
Cronobacter sakazakii
Citrobacter freundii
Cedecea neteri
Candidatus Tachikawaea
Candidatus Riesia
Candidatus Ishikawaella
Candidatus Hoaglandella
Candidatus Gullanella
Candidatus Doolittlea
0
500
1000
1500
2000
Position along marker gene

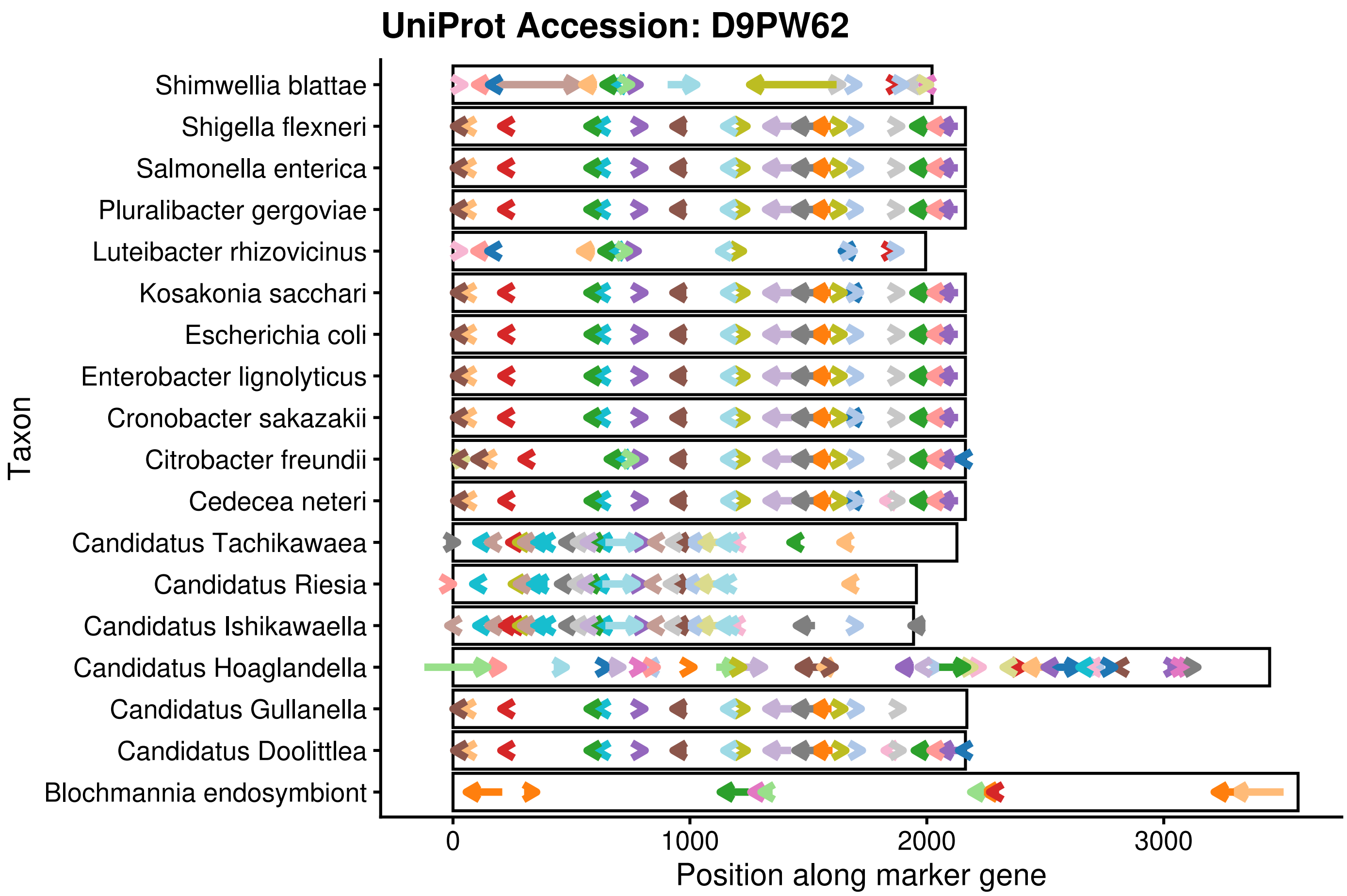
UniProt Accession: D9PW62
Taxon
Shimwellia blattae
Shigella flexneri
Salmonella enterica
Pluralibacter gergoviae
Luteibacter rhizovicinus
Kosakonia sacchari
Escherichia coli
Enterobacter lignolyticus
Cronobacter sakazakii
Citrobacter freundii
Cedecea neteri
Candidatus Tachikawaea
Candidatus Riesia
Candidatus Ishikawaella
Candidatus Hoaglandella
Candidatus Gullanella
Candidatus Doolittlea
Blochmannia endosymbiont
0
1000
2000
3000
Position along marker gene

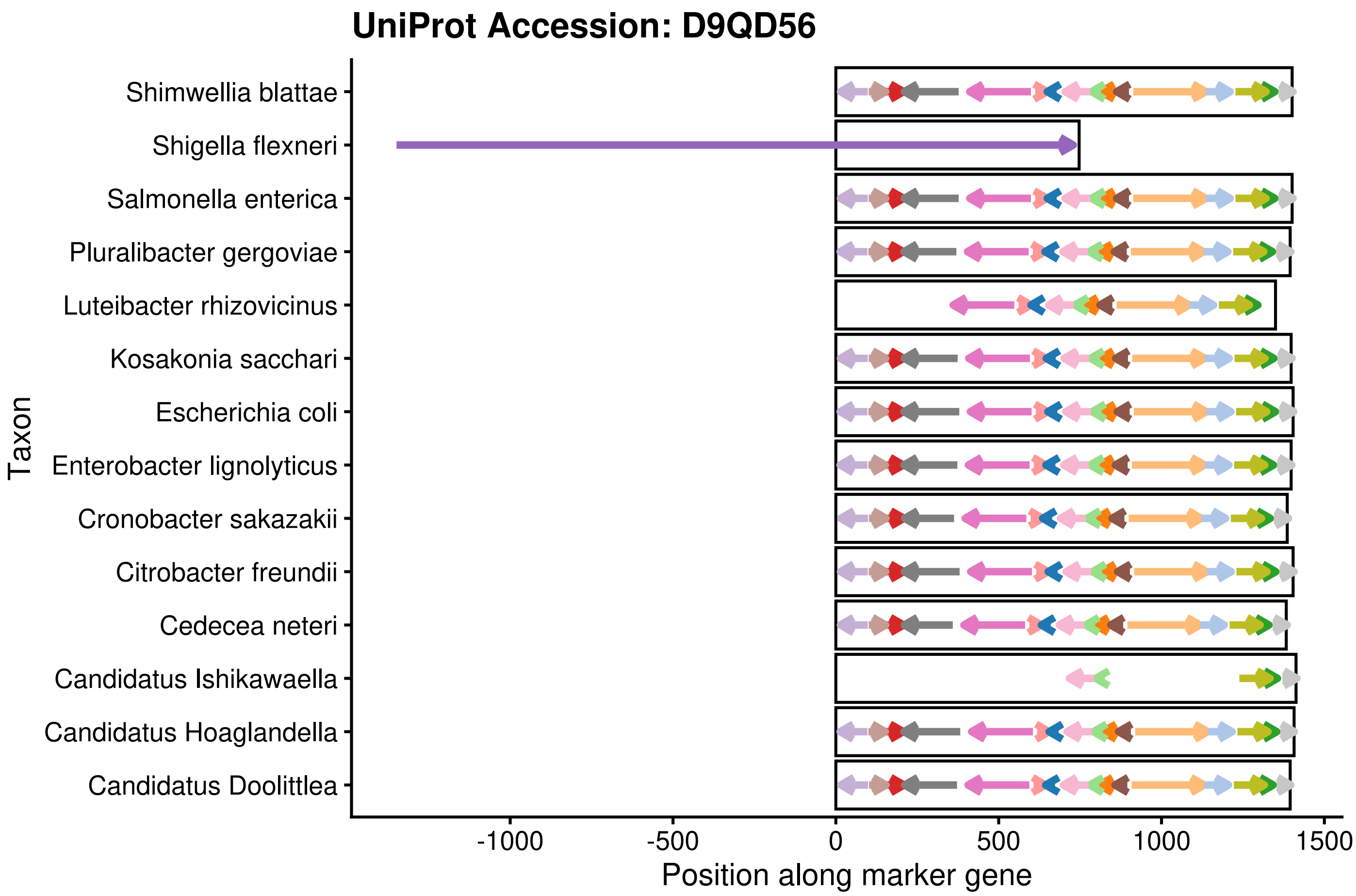
UniProt Accession: D9QD56
Taxon
Shimwellia blattae
Shigella flexneri
Salmonella enterica
Pluralibacter gergoviae
Luteibacter rhizovicinus
Kosakonia sacchari
Escherichia coli
Enterobacter lignolyticus
Cronobacter sakazakii
Citrobacter freundii
Cedecea neteri
Candidatus Ishikawaella
Candidatus Hoaglandella
Candidatus Doolittlea
-1000
-500
0
500
1000
1500
Position along marker gene

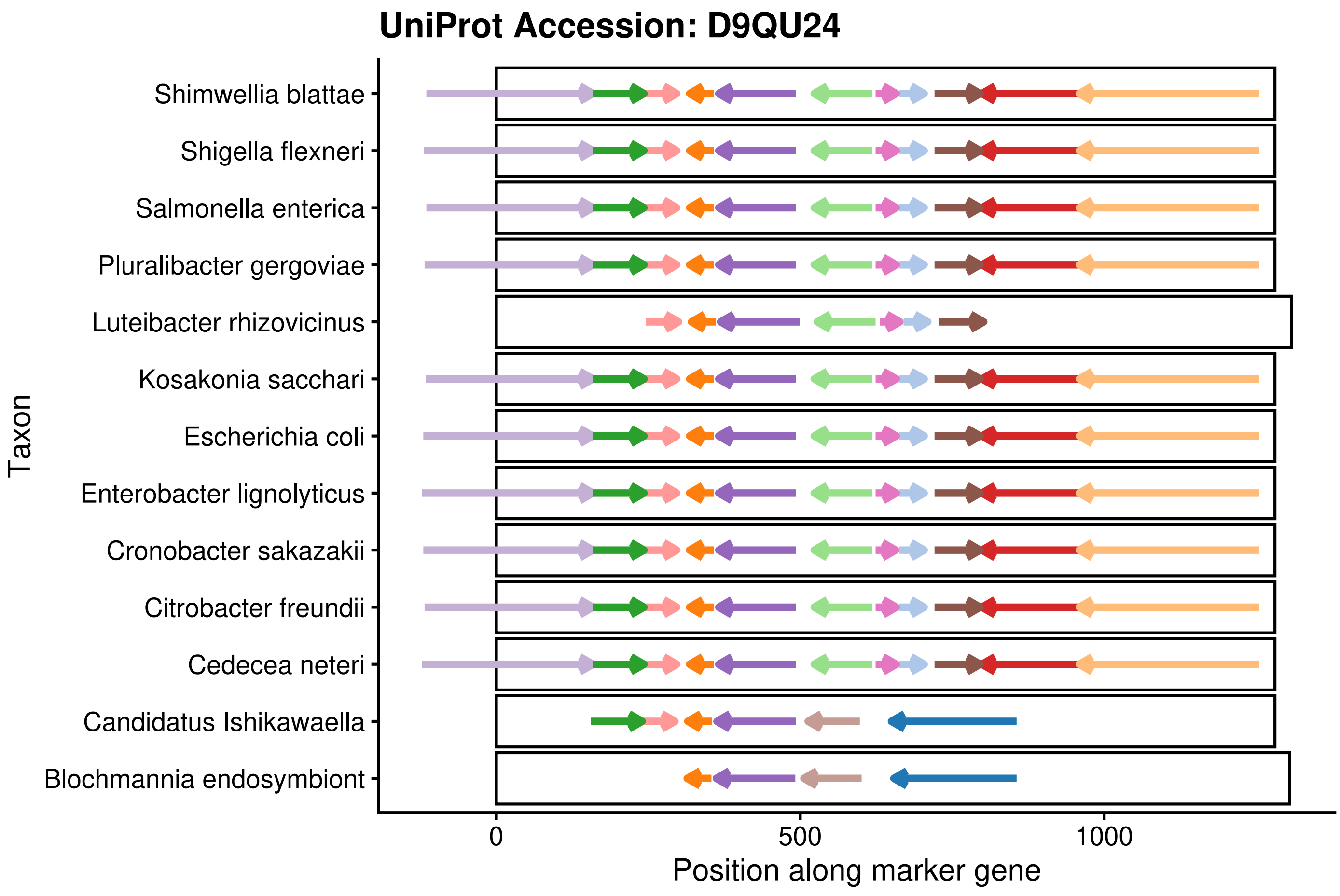
UniProt Accession: D9QU24
Taxon
Shimwellia blattae
Shigella flexneri
Salmonella enterica
Pluralibacter gergoviae
Luteibacter rhizovicinus
Kosakonia sacchari
Escherichia coli
Enterobacter lignolyticus
Cronobacter sakazakii
Citrobacter freundii
Cedecea neteri
Candidatus Ishikawaella
Blochmannia endosymbiont
0
500
1000
Position along marker gene

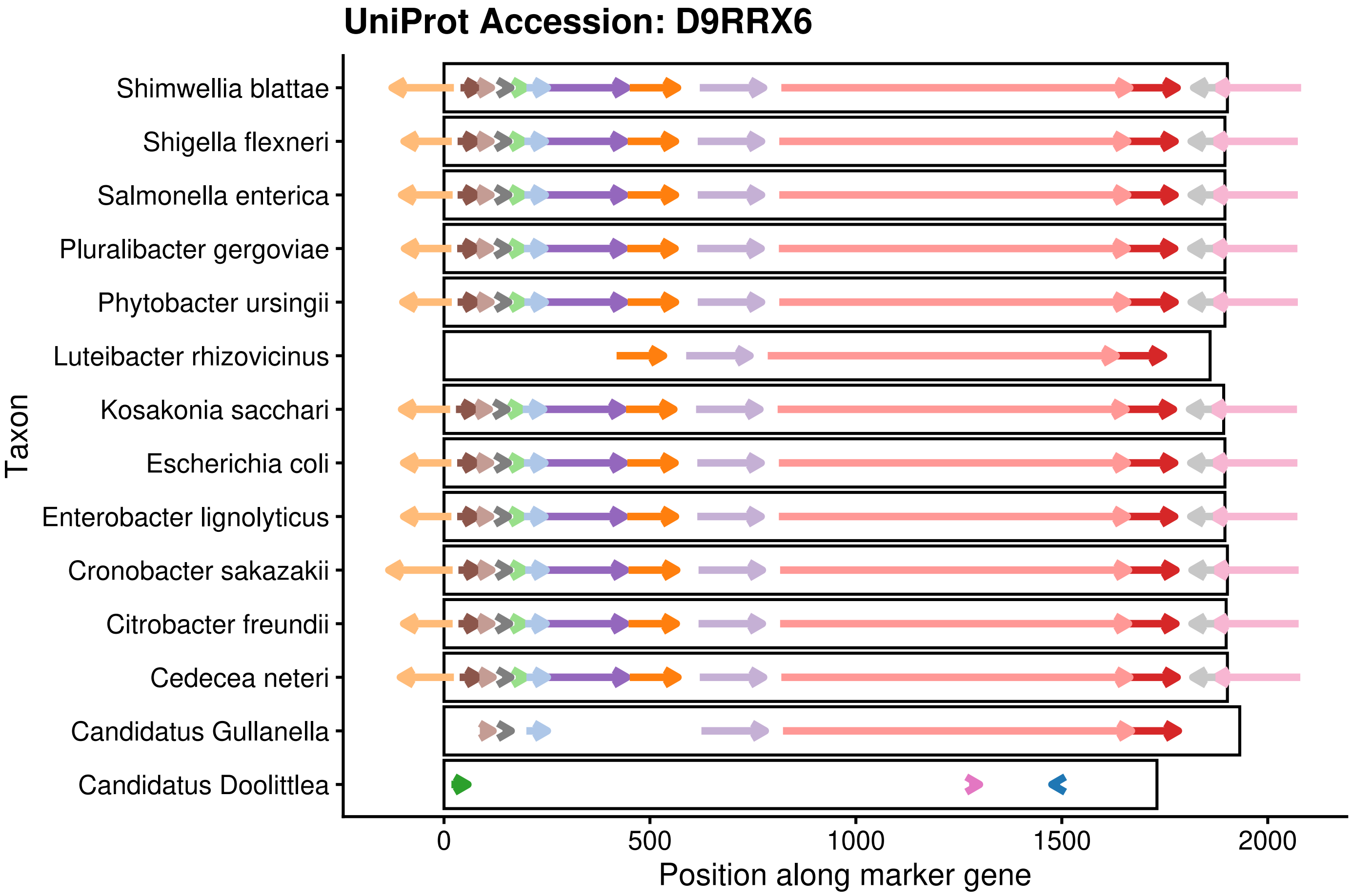

UniProt Accession: D9RRX6
Shimwellia blattae
Shigella flexneri
Salmonella enterica
Pluralibacter gergoviae
Phytobacter ursingii
Luteibacter rhizovicinus
Kosakonia sacchari
Escherichia coli
Enterobacter lignolyticus
Cronobacter sakazakii
Citrobacter freundii
Cedecea neteri
Candidatus Gullanella
Candidatus Doolittlea
Taxon
0
500
1000
1500
2000
Position along marker gene

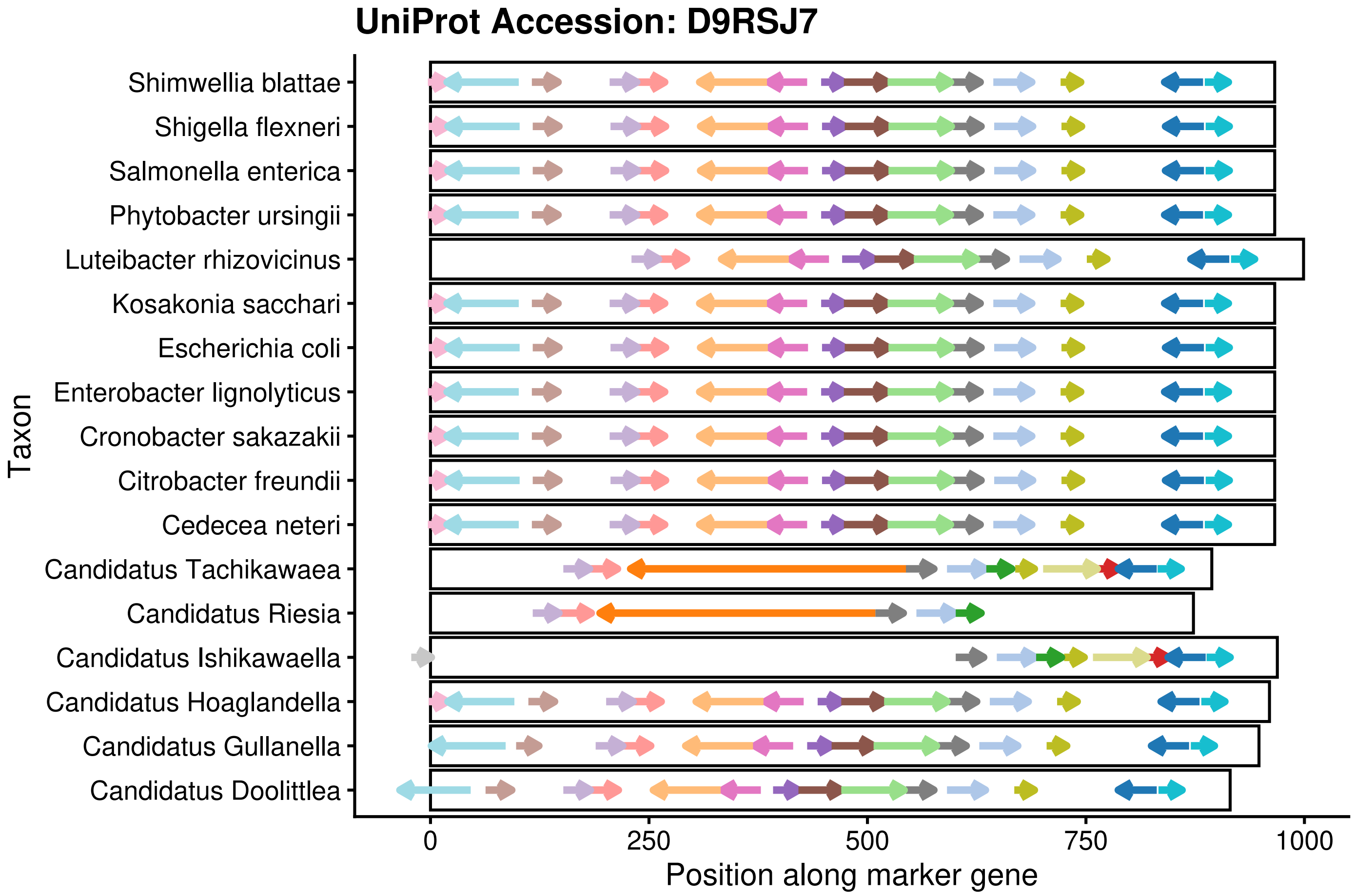

UniProt Accession: D9RSJ7
Shimwellia blattae
Shigella flexneri
Salmonella enterica
Phytobacter ursingii
Luteibacter rhizovicinus
Kosakonia sacchari
Escherichia coli
Enterobacter lignolyticus
Cronobacter sakazakii
Citrobacter freundii
Cedecea neteri
Candidatus Tachikawaea
Candidatus Riesia
Candidatus Ishikawaella
Candidatus Hoaglandella
Candidatus Gullanella
Candidatus Doolittlea
Taxon
0
250
500
750
1000
Position along marker gene

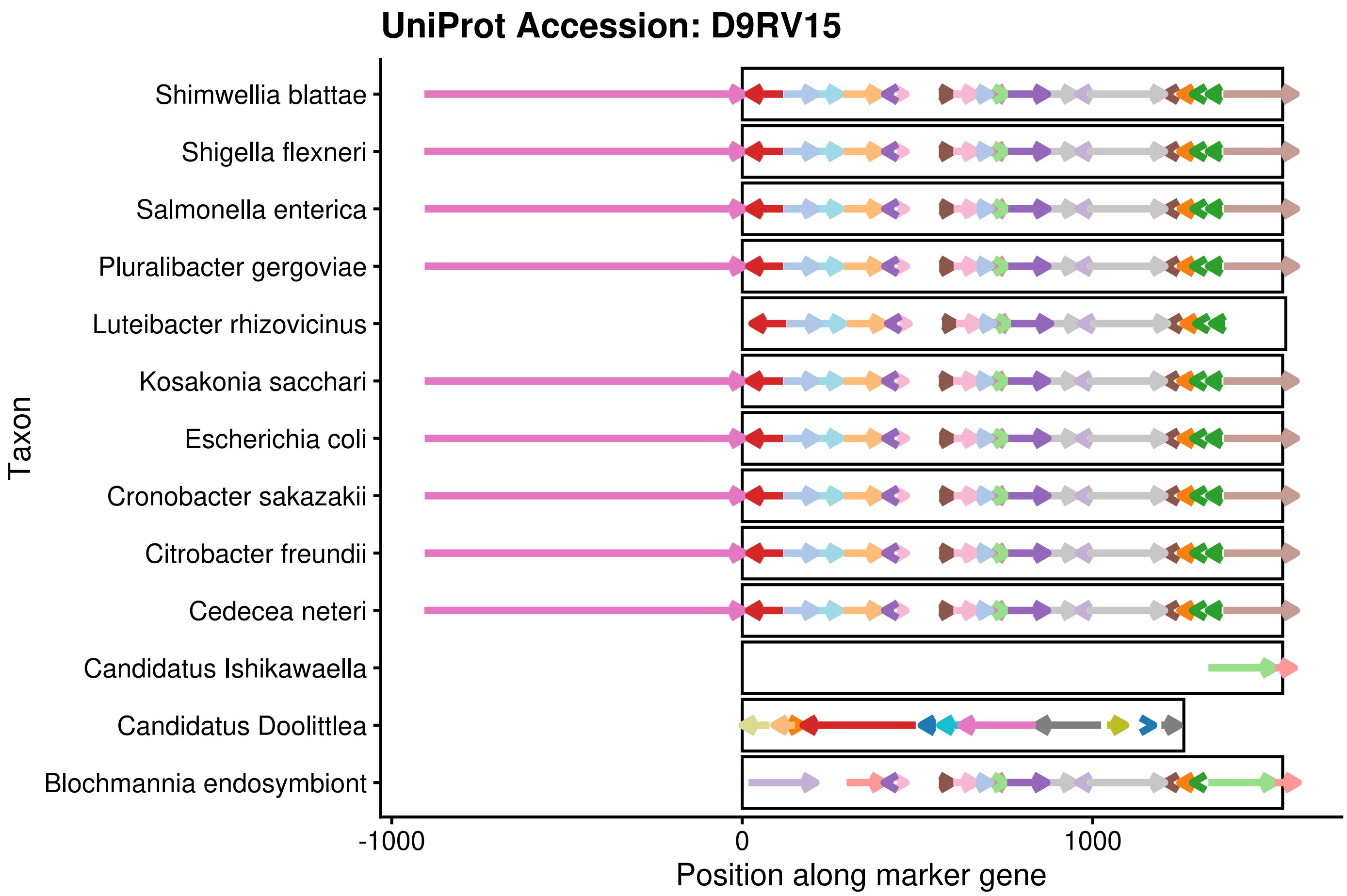
UniProt Accession: D9RV15
Shimwellia blattae
Shigella flexneri
Salmonella enterica
Pluralibacter gergoviae
Luteibacter rhizovicinus
Kosakonia sacchari
Escherichia coli
Cronobacter sakazakii
Citrobacter freundii
Cedecea neteri
Candidatus Ishikawaella
Candidatus Doolittlea
Blochmannia endosymbiont
Taxon
-1000
0
1000
Position along marker gene

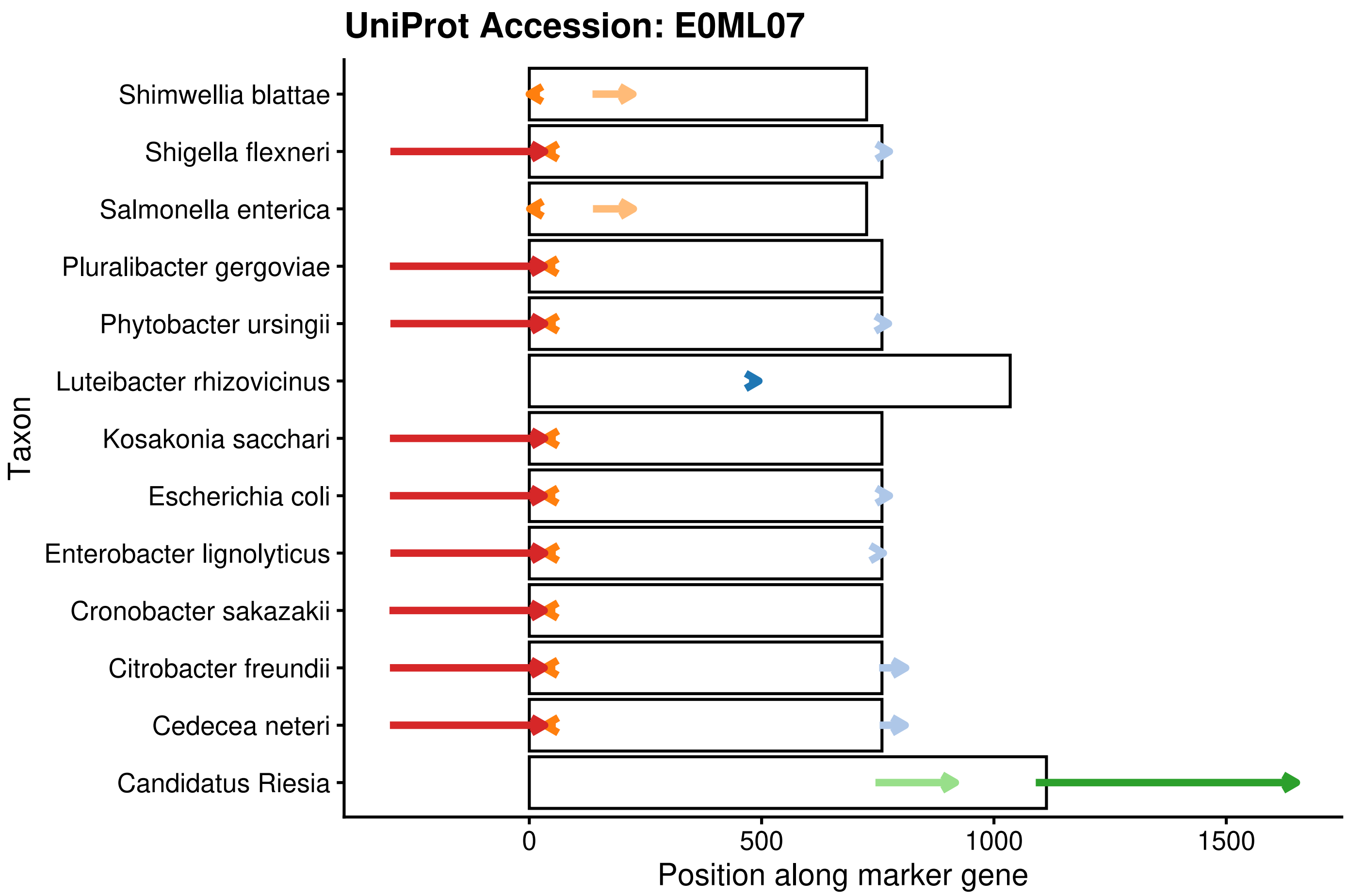
UniProt Accession: E0ML07
Taxon
Shimwellia blattae
Shigella flexneri
Salmonella enterica
Pluralibacter gergoviae
Phytobacter ursingii
Luteibacter rhizovicinus
Kosakonia sacchari
Escherichia coli
Enterobacter lignolyticus
Cronobacter sakazakii
Citrobacter freundii
Cedecea neteri
Candidatus Riesia
0
500
1000
1500
Position along marker gene

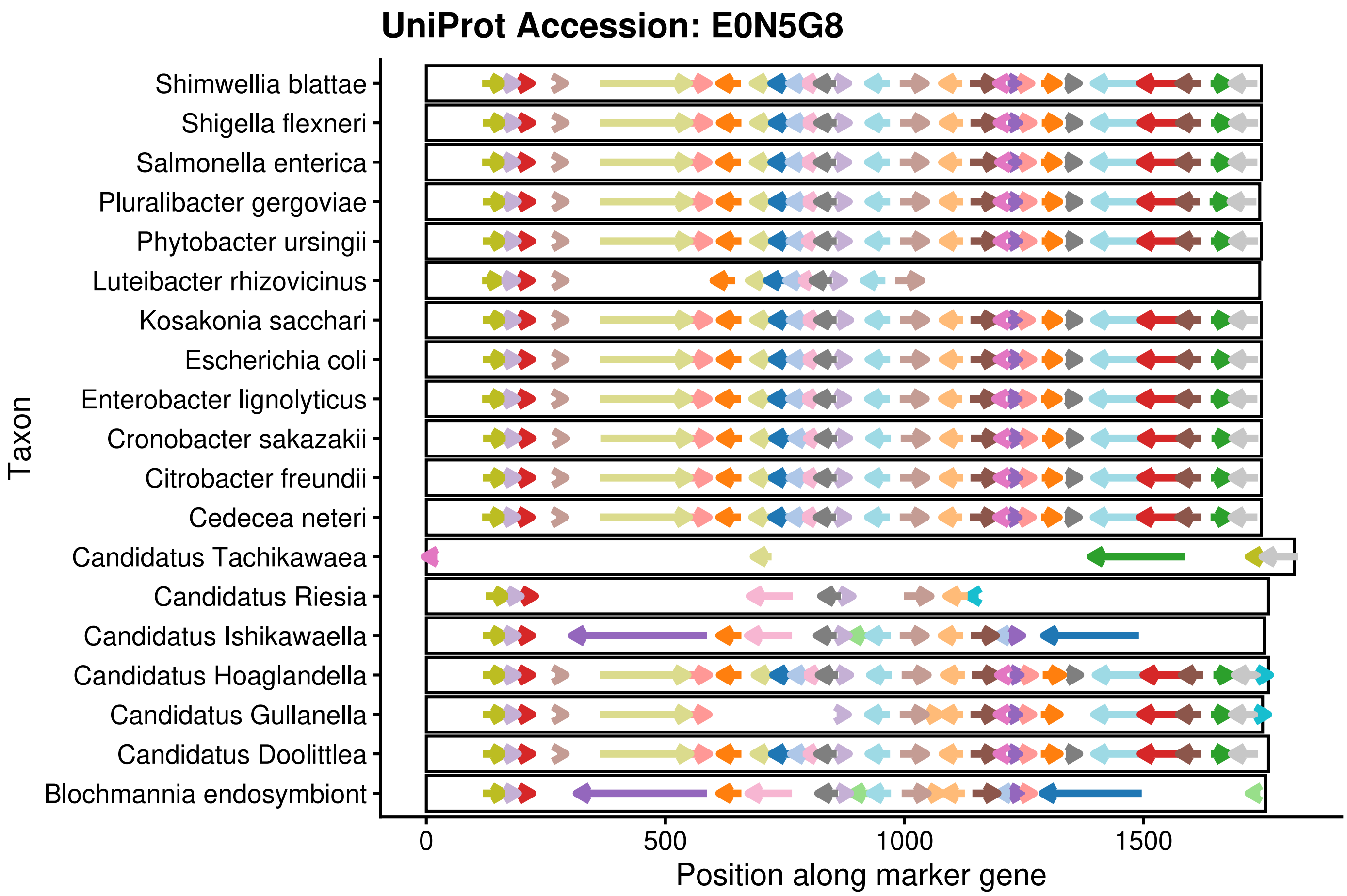
UniProt Accession: E0N5G8
Shimwellia blattae
Shigella flexneri
Salmonella enterica
Pluralibacter gergoviae
Phytobacter ursingii
Luteibacter rhizovicinus
Kosakonia sacchari
Escherichia coli
Enterobacter lignolyticus
Cronobacter sakazakii
Citrobacter freundii
Cedecea neteri
Candidatus Tachikawaea
Candidatus Riesia
Candidatus Ishikawaella
Candidatus Hoaglandella
Candidatus Gullanella
Candidatus Doolittlea
Blochmannia endosymbiont
Taxon
0
500
1000
1500
Position along marker gene

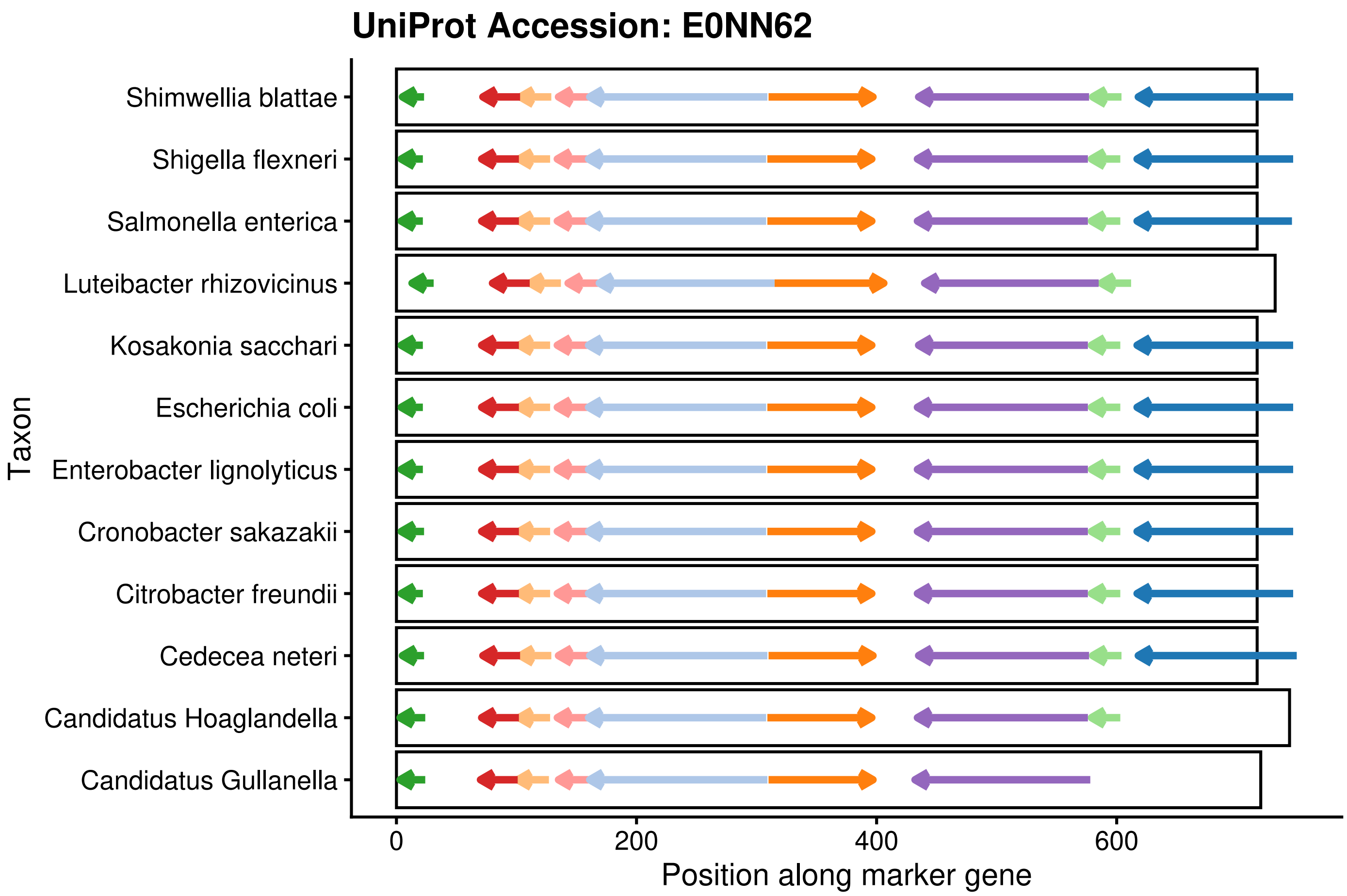
UniProt Accession: E0NN62
Shimwellia blattae
Shigella flexneri
Salmonella enterica
Luteibacter rhizovicinus
Kosakonia sacchari
Escherichia coli
Enterobacter lignolyticus
Cronobacter sakazakii
Citrobacter freundii
Cedecea neteri
Candidatus Hoaglandella
Candidatus Gullanella
Taxon
0
200
400
600
Position along marker gene

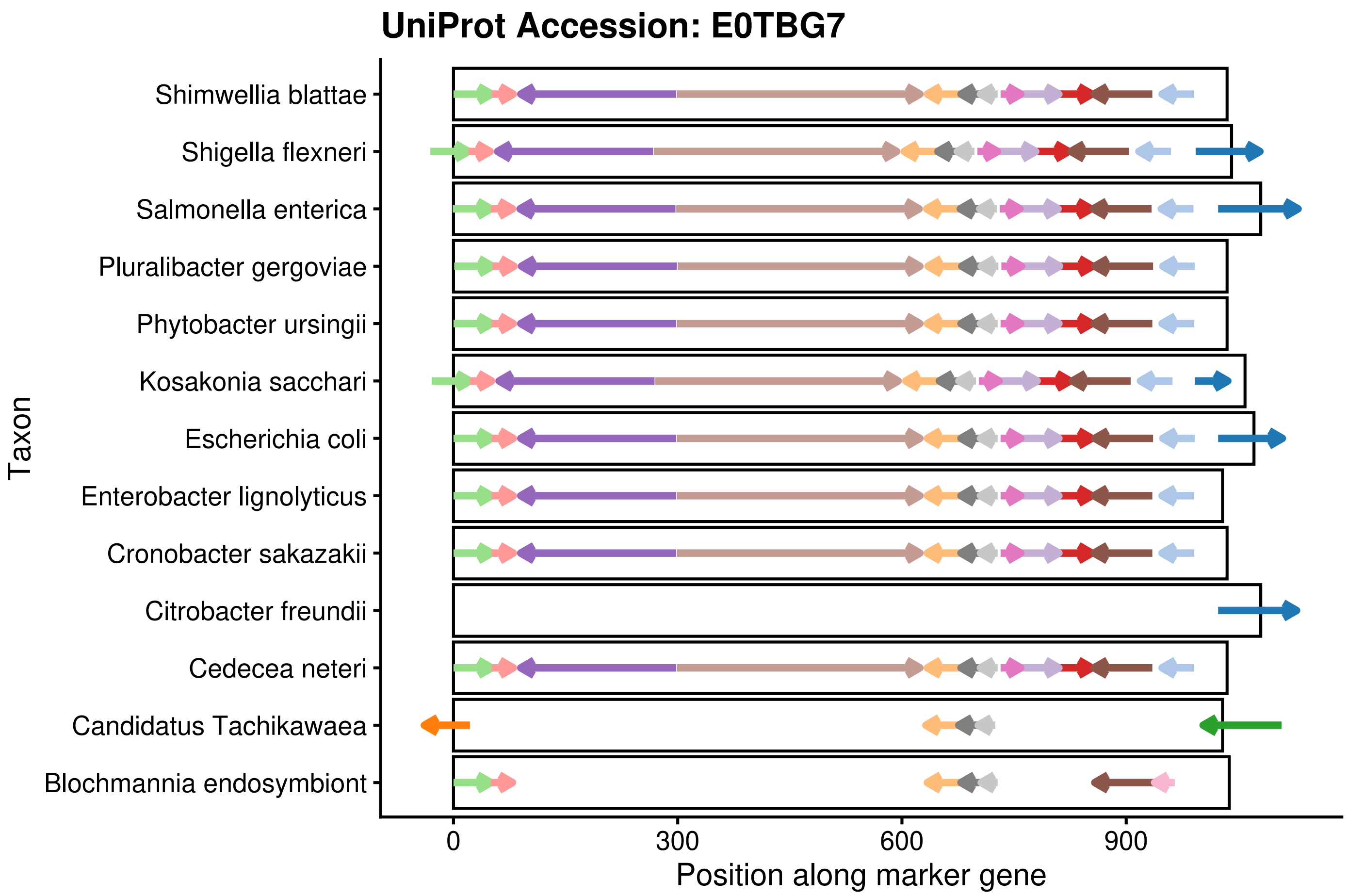

UniProt Accession: E0TBG7
Shimwellia blattae
Shigella flexneri
Salmonella enterica
Pluralibacter gergoviae
Phytobacter ursingii
Kosakonia sacchari
Escherichia coli
Enterobacter lignolyticus
Cronobacter sakazakii
Citrobacter freundii
Cedecea neteri
Candidatus Tachikawaea
Blochmannia endosymbiont
Taxon
0
300
600
900
Position along marker gene

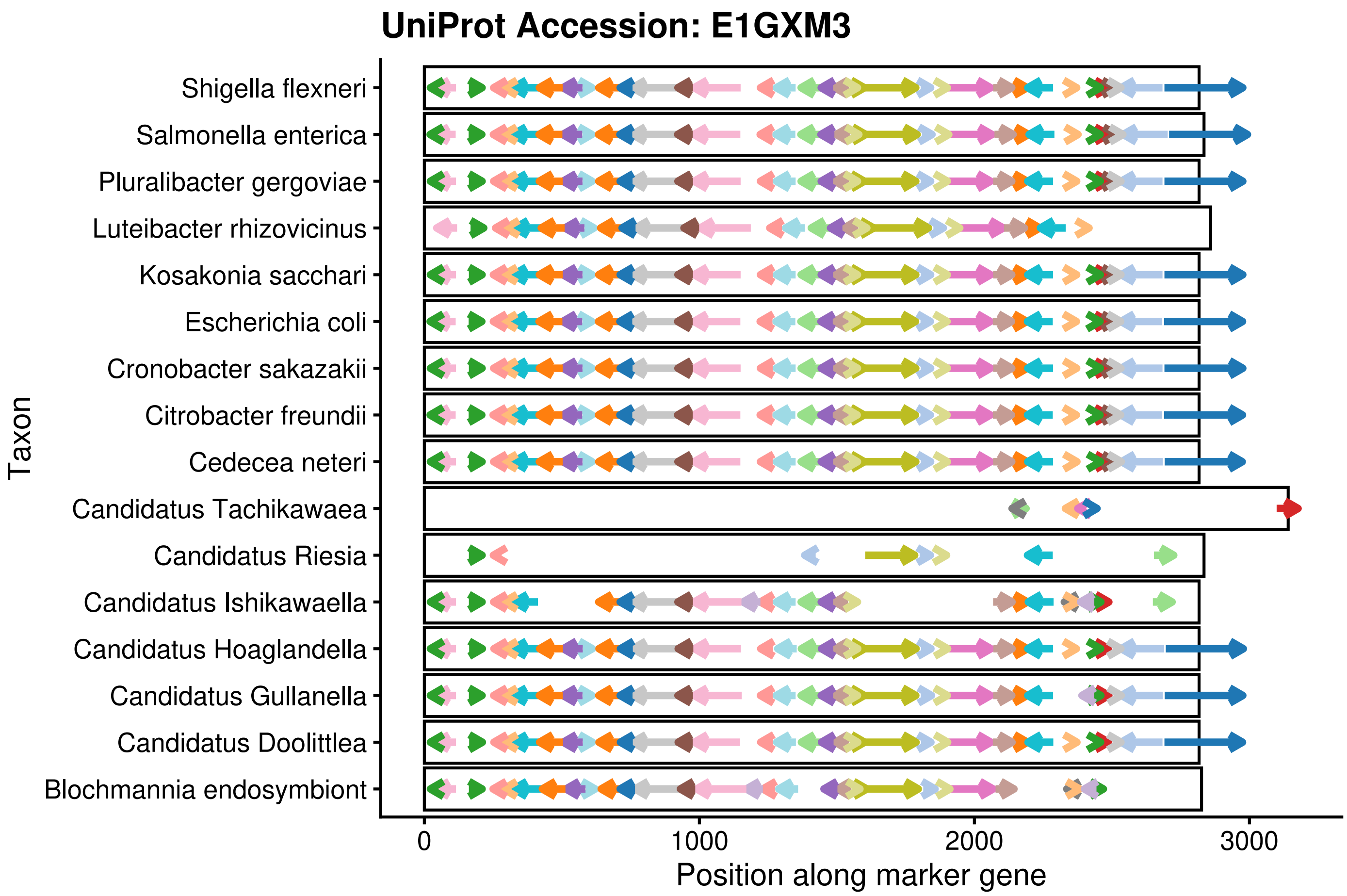

UniProt Accession: E1GXM3
Taxon
Shigella flexneri
Salmonella enterica
Pluralibacter gergoviae
Luteibacter rhizovicinus
Kosakonia sacchari
Escherichia coli
Cronobacter sakazakii
Citrobacter freundii
Cedecea neteri
Candidatus Tachikawaea
Candidatus Riesia
Candidatus Ishikawaella
Candidatus Hoaglandella
Candidatus Gullanella
Candidatus Doolittlea
Blochmannia endosymbiont
0
1000
2000
3000
Position along marker gene

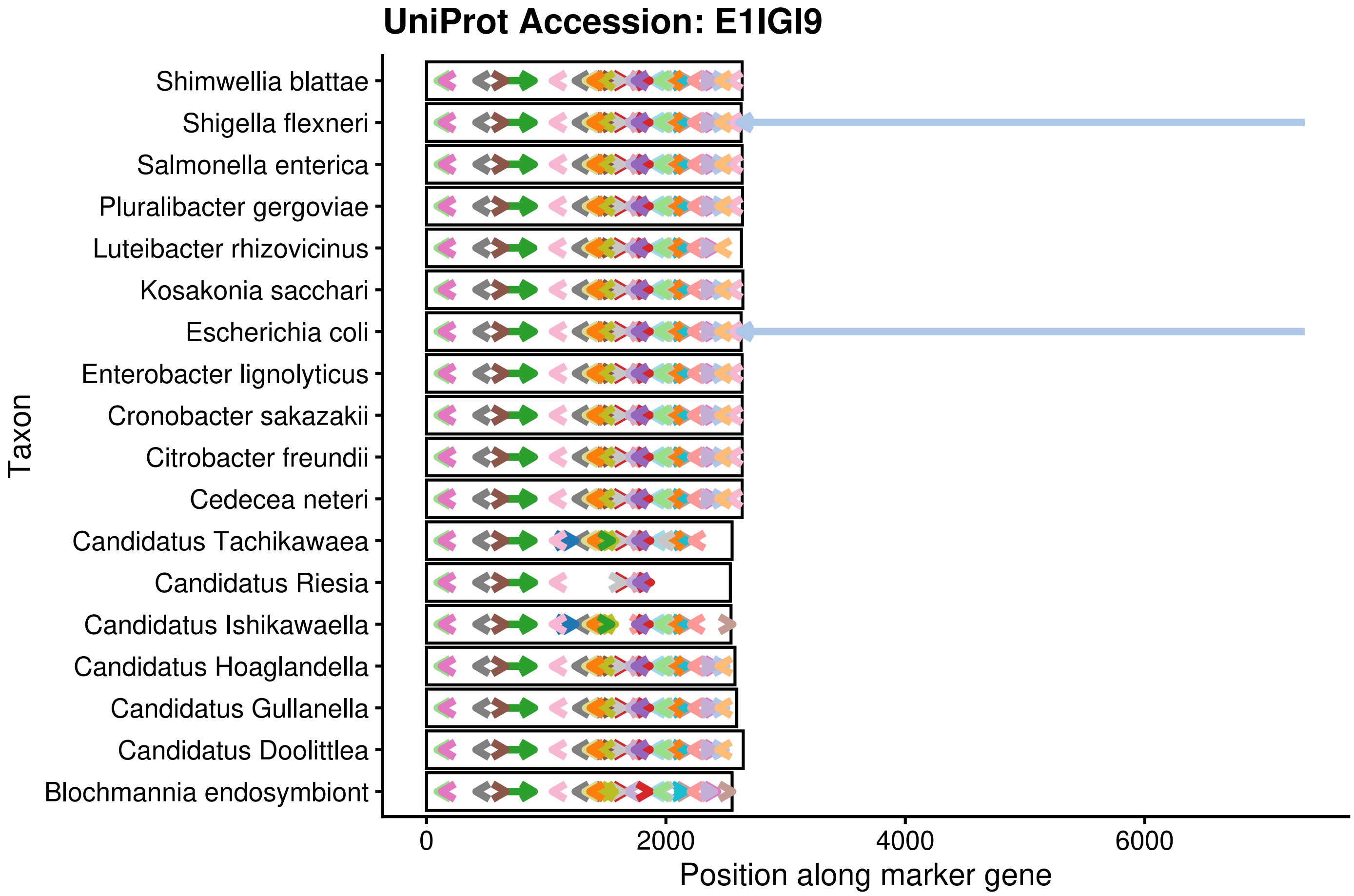

UniProt Accession: E1IGI9
Shimwellia blattae
Shigella flexneri
Salmonella enterica
Pluralibacter gergoviae
Luteibacter rhizovicinus
Kosakonia sacchari
Escherichia coli
Enterobacter lignolyticus
Cronobacter sakazakii
Citrobacter freundii
Cedecea neteri
Candidatus Tachikawaea
Candidatus Riesia
Candidatus Ishikawaella
Candidatus Hoaglandella
Candidatus Gullanella
Candidatus Doolittlea
Blochmannia endosymbiont
Taxon
0
2000
4000
6000
Position along marker gene

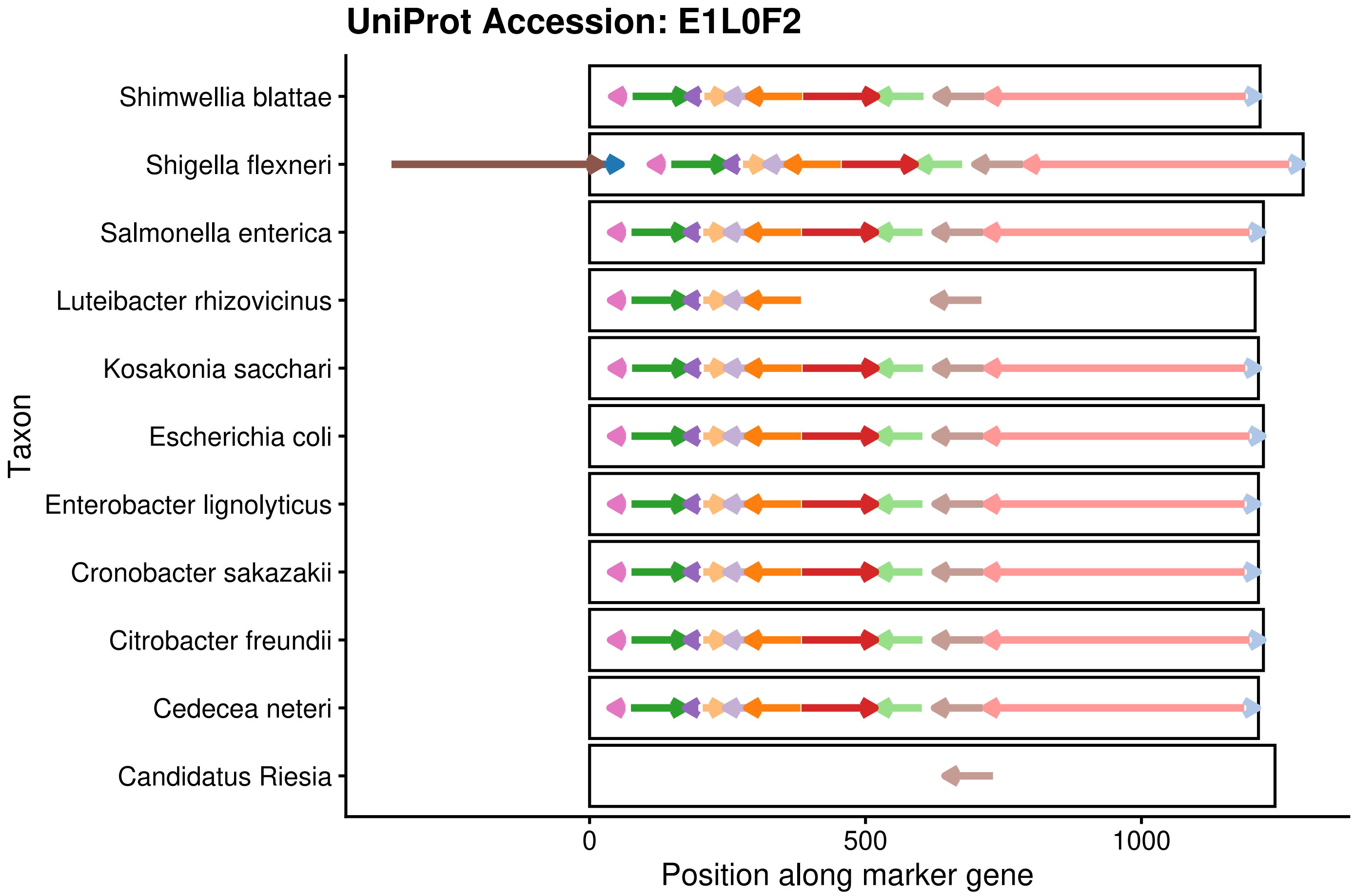

UniProt Accession: E1L0F2
Shimwellia blattae
Shigella flexneri
Salmonella enterica
Luteibacter rhizovicinus
Kosakonia sacchari
Escherichia coli
Enterobacter lignolyticus
Cronobacter sakazakii
Citrobacter freundii
Cedecea neteri
Candidatus Riesia
Taxon
0
500
1000
Position along marker gene

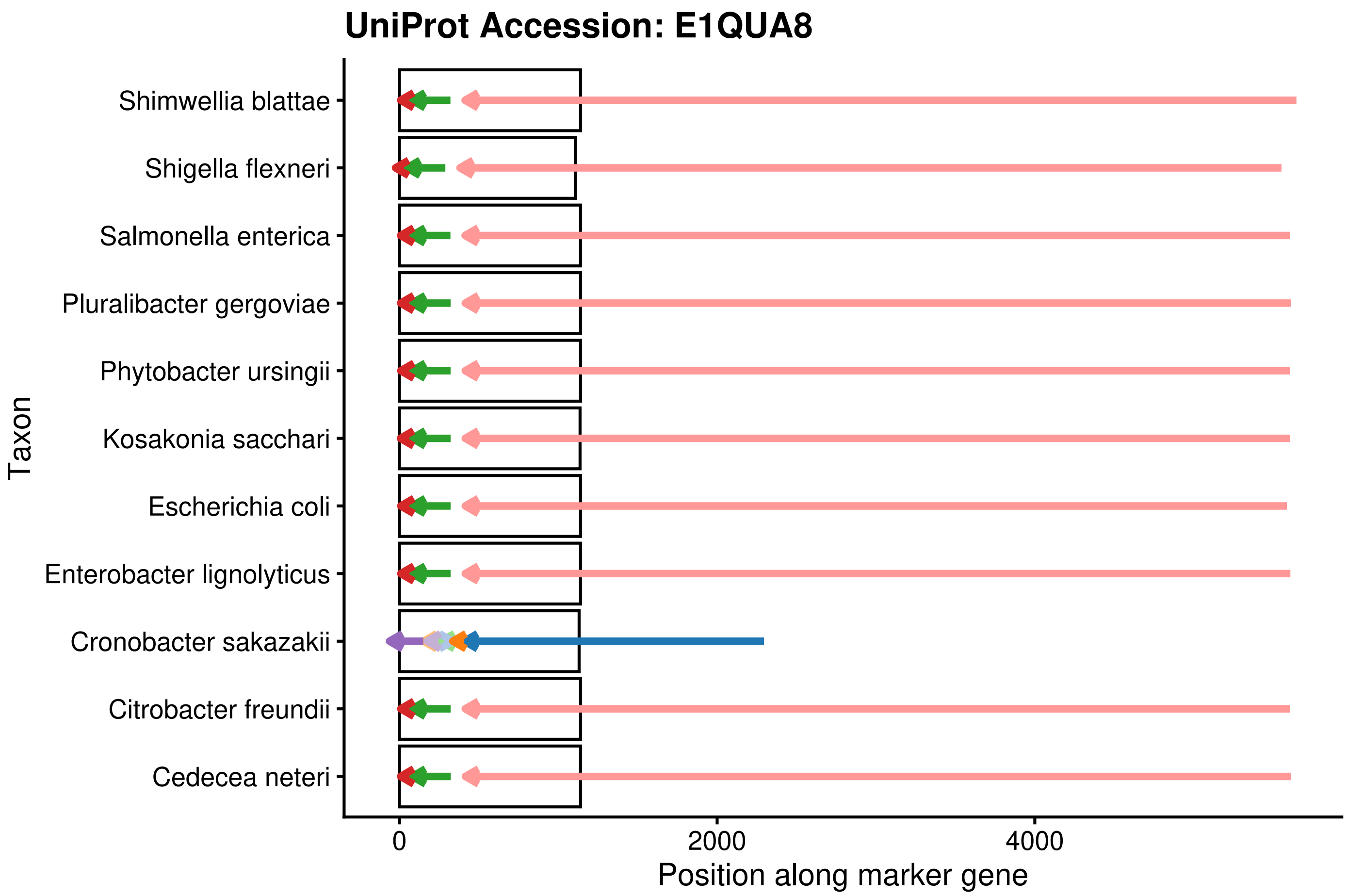

UniProt Accession: E1QUA8
Shimwellia blattae
Shigella flexneri
Salmonella enterica
Pluralibacter gergoviae
Phytobacter ursingii
Kosakonia sacchari
Escherichia coli
Enterobacter lignolyticus
Cronobacter sakazakii
Citrobacter freundii
Cedecea neteri
Taxon
0
2000
4000
Position along marker gene

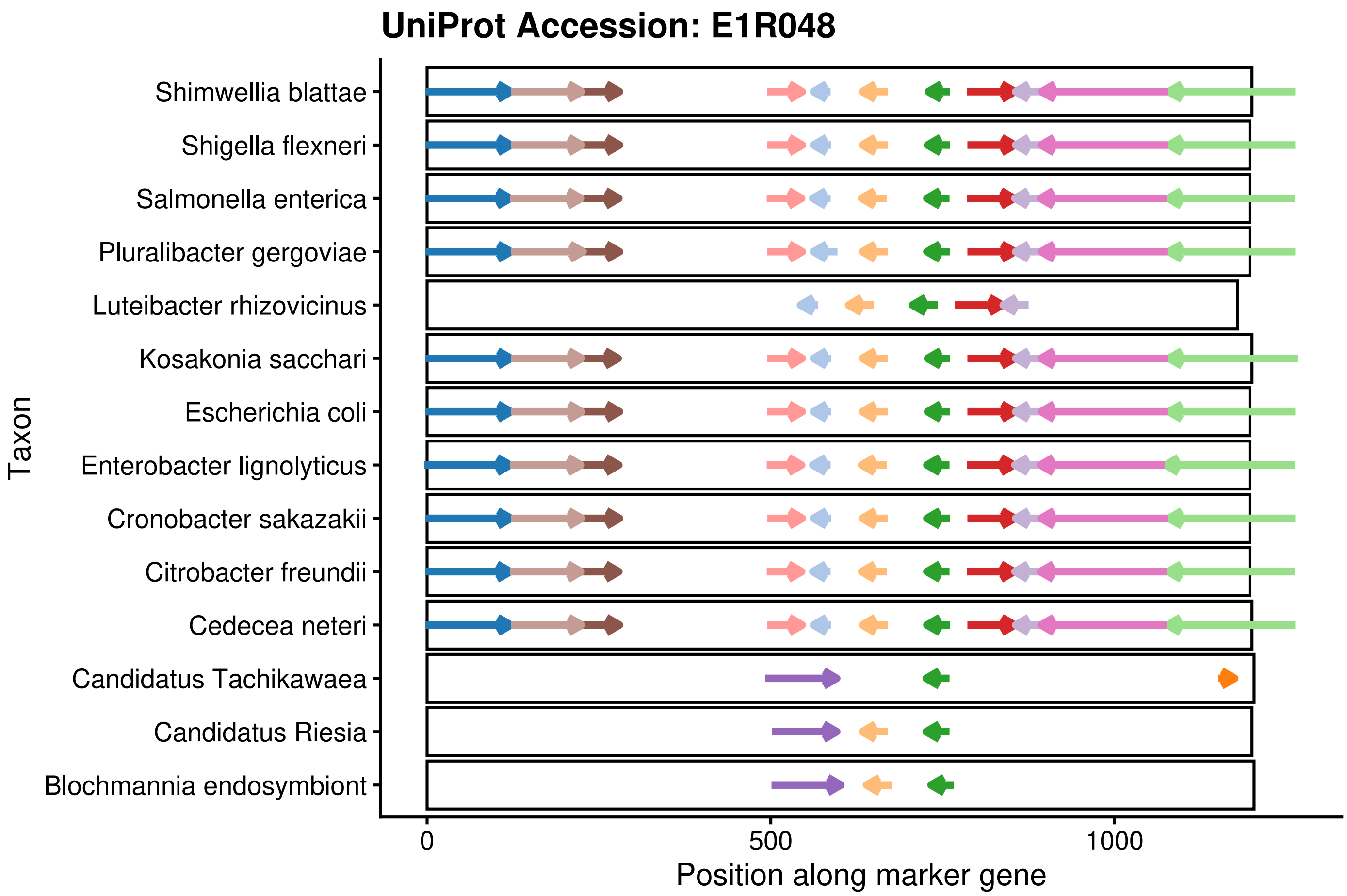
UniProt Accession: E1R048
Taxon
Shimwellia blattae
Shigella flexneri
Salmonella enterica
Pluralibacter gergoviae
Luteibacter rhizovicinus
Kosakonia sacchari
Escherichia coli
Enterobacter lignolyticus
Cronobacter sakazakii
Citrobacter freundii
Cedecea neteri
Candidatus Tachikawaea
Candidatus Riesia
Blochmannia endosymbiont
0
500
1000
Position along marker gene

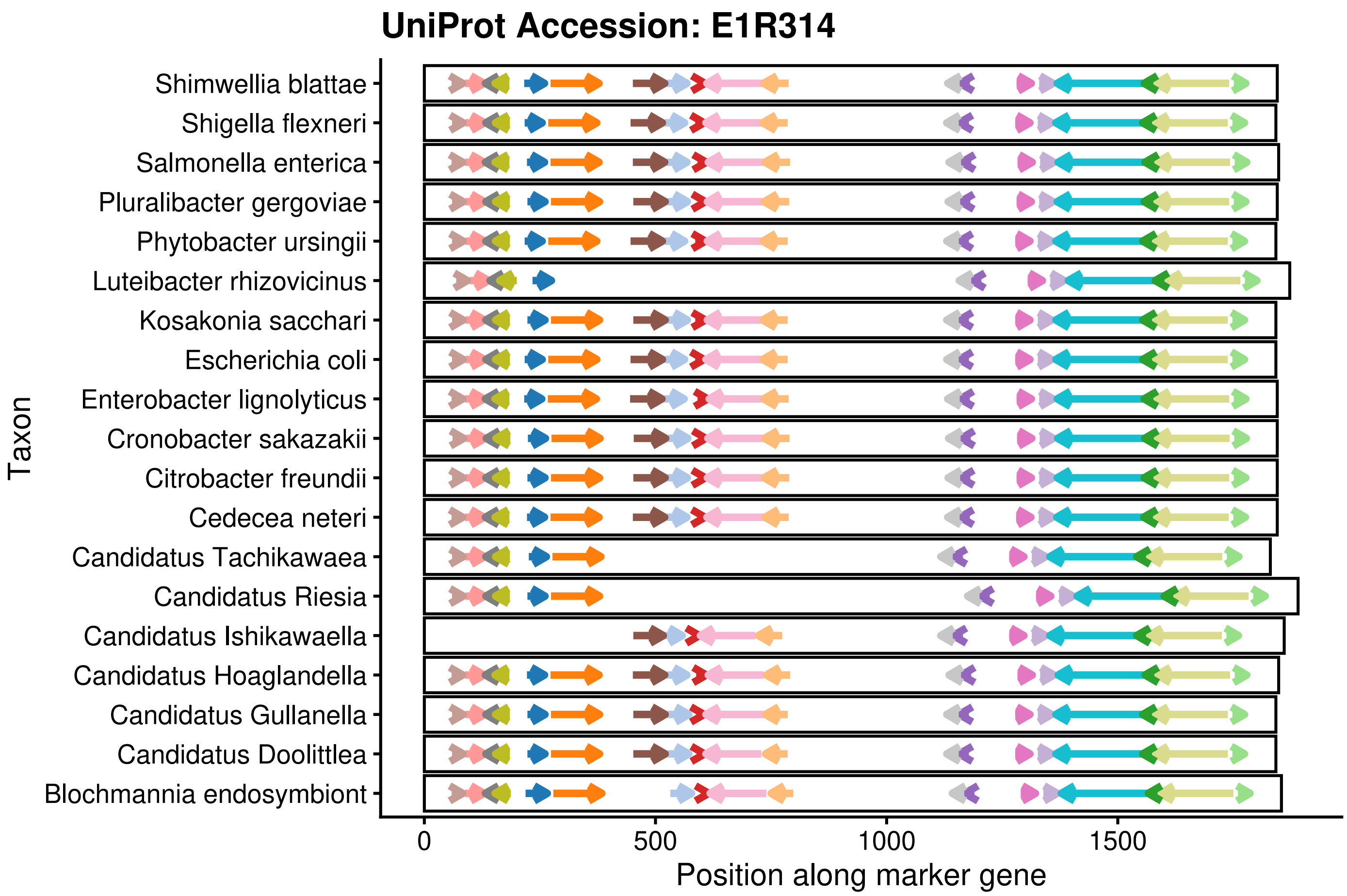

UniProt Accession: E1R314
Shimwellia blattae
Shigella flexneri
Salmonella enterica
Pluralibacter gergoviae
Phytobacter ursingii
Luteibacter rhizovicinus
Kosakonia sacchari
Escherichia coli
Enterobacter lignolyticus
Cronobacter sakazakii
Citrobacter freundii
Cedecea neteri
Candidatus Tachikawaea
Candidatus Riesia
Candidatus Ishikawaella
Candidatus Hoaglandella
Candidatus Gullanella
Candidatus Doolittlea
Blochmannia endosymbiont
Taxon
0
500
1000
1500
Position along marker gene

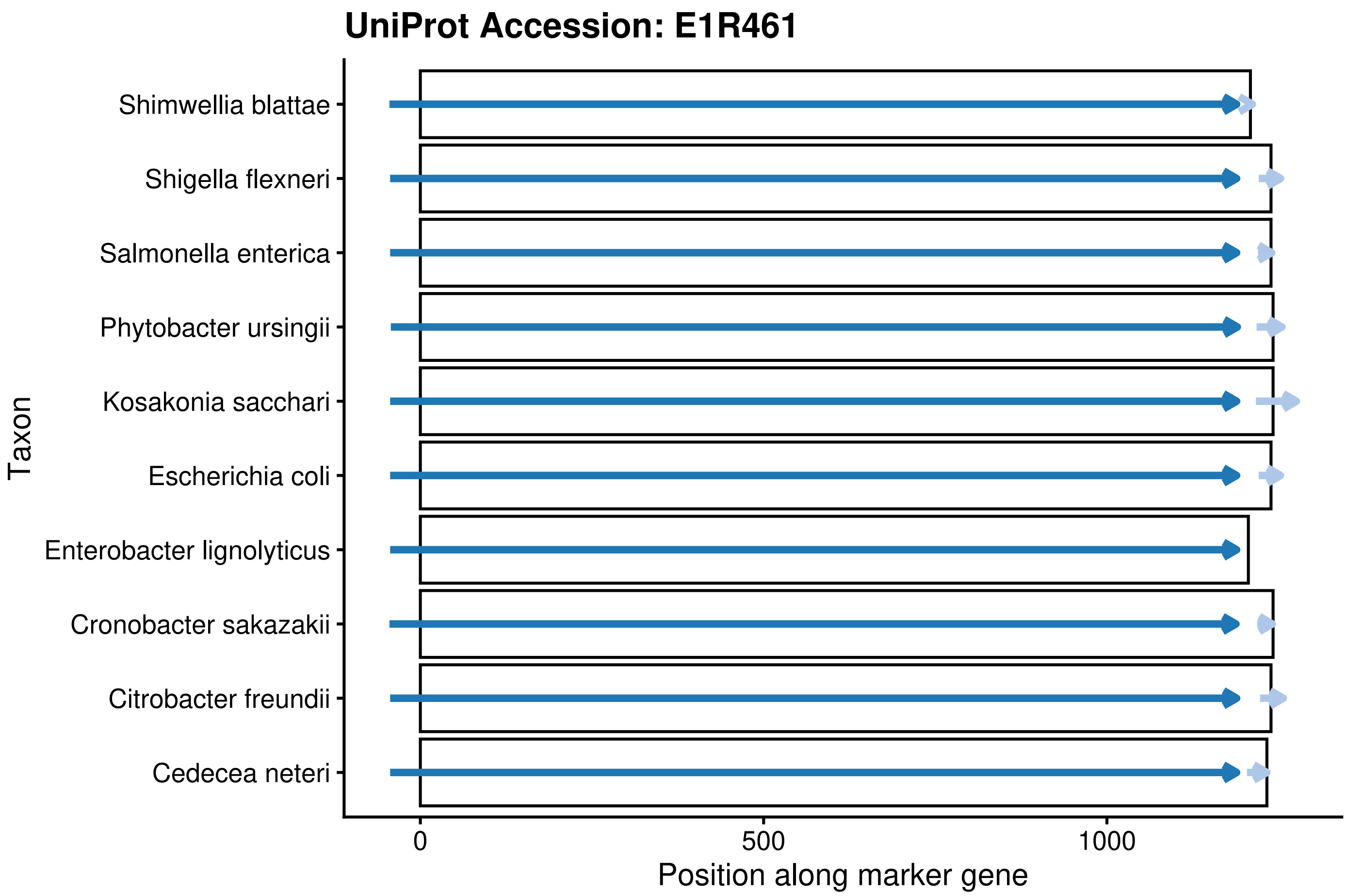

UniProt Accession: E1R461
Shimwellia blattae
Shigella flexneri
Salmonella enterica
Phytobacter ursingii
Kosakonia sacchari
Escherichia coli
Enterobacter lignolyticus
Cronobacter sakazakii
Citrobacter freundii
Cedecea neteri
Taxon
0
500
1000
Position along marker gene

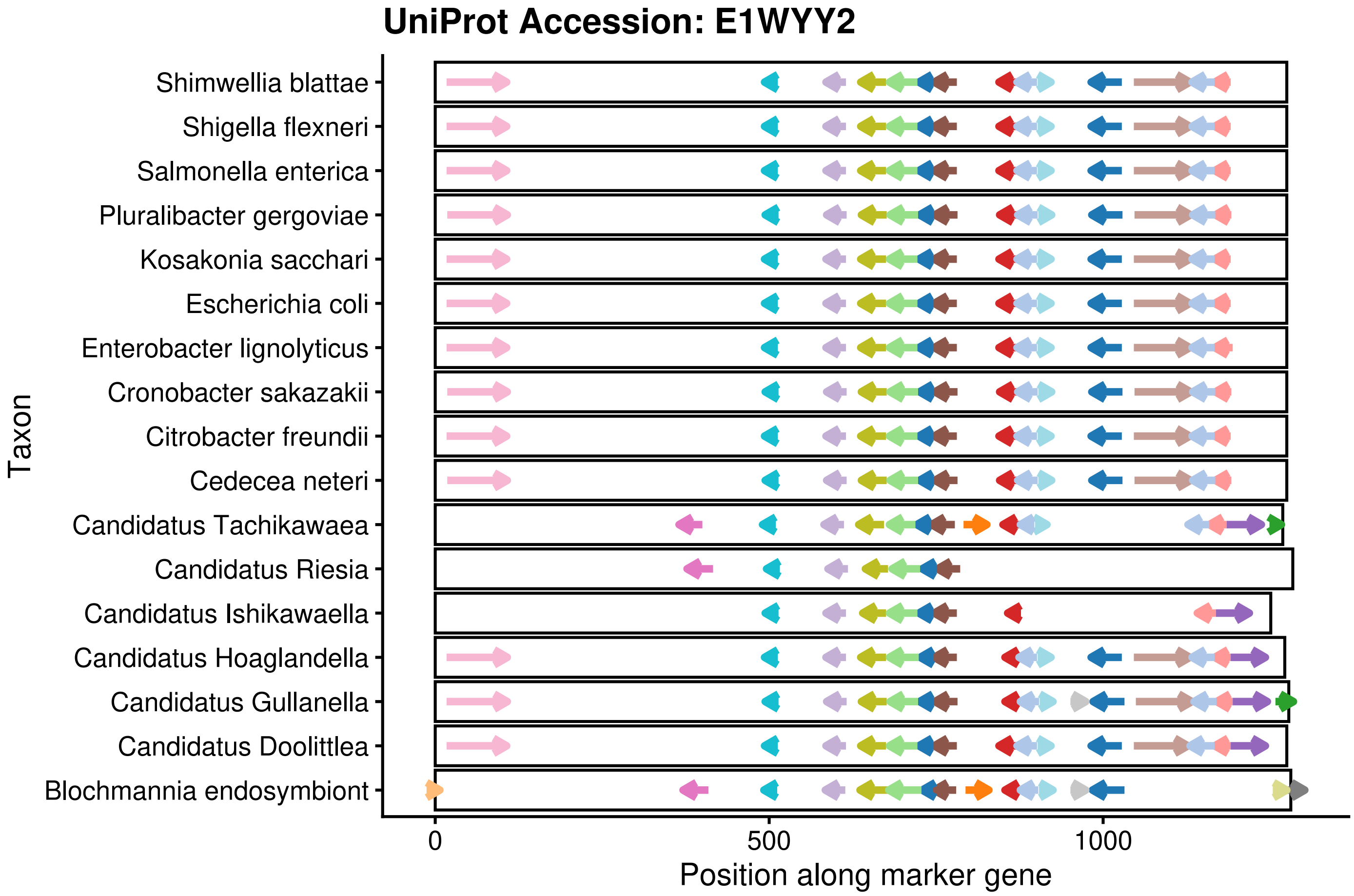

UniProt Accession: E1WYY2
Shimwellia blattae
Shigella flexneri
Salmonella enterica
Pluralibacter gergoviae
Kosakonia sacchari
Escherichia coli
Enterobacter lignolyticus
Cronobacter sakazakii
Citrobacter freundii
Cedecea neteri
Candidatus Tachikawaea
Candidatus Riesia
Candidatus Ishikawaella
Candidatus Hoaglandella
Candidatus Gullanella
Candidatus Doolittlea
Blochmannia endosymbiont
Taxon
0
500
1000
Position along marker gene

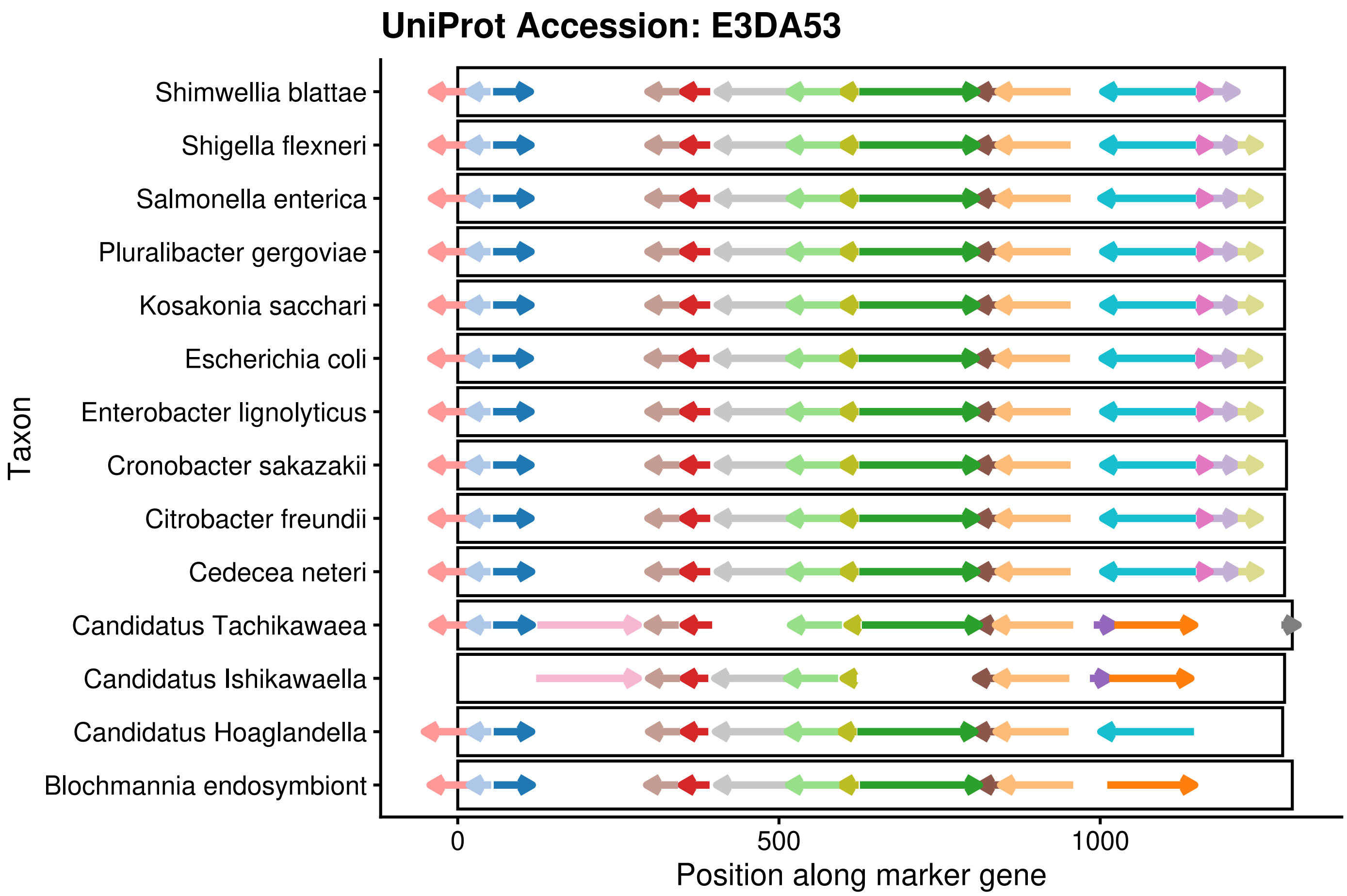
UniProt Accession: E3DA53
Shimwellia blattae
Shigella flexneri
Salmonella enterica
Pluralibacter gergoviae
Kosakonia sacchari
Escherichia coli
Enterobacter lignolyticus
Cronobacter sakazakii
Citrobacter freundii
Cedecea neteri
Candidatus Tachikawaea
Candidatus Ishikawaella
Candidatus Hoaglandella
Blochmannia endosymbiont
Taxon
0
500
1000
Position along marker gene

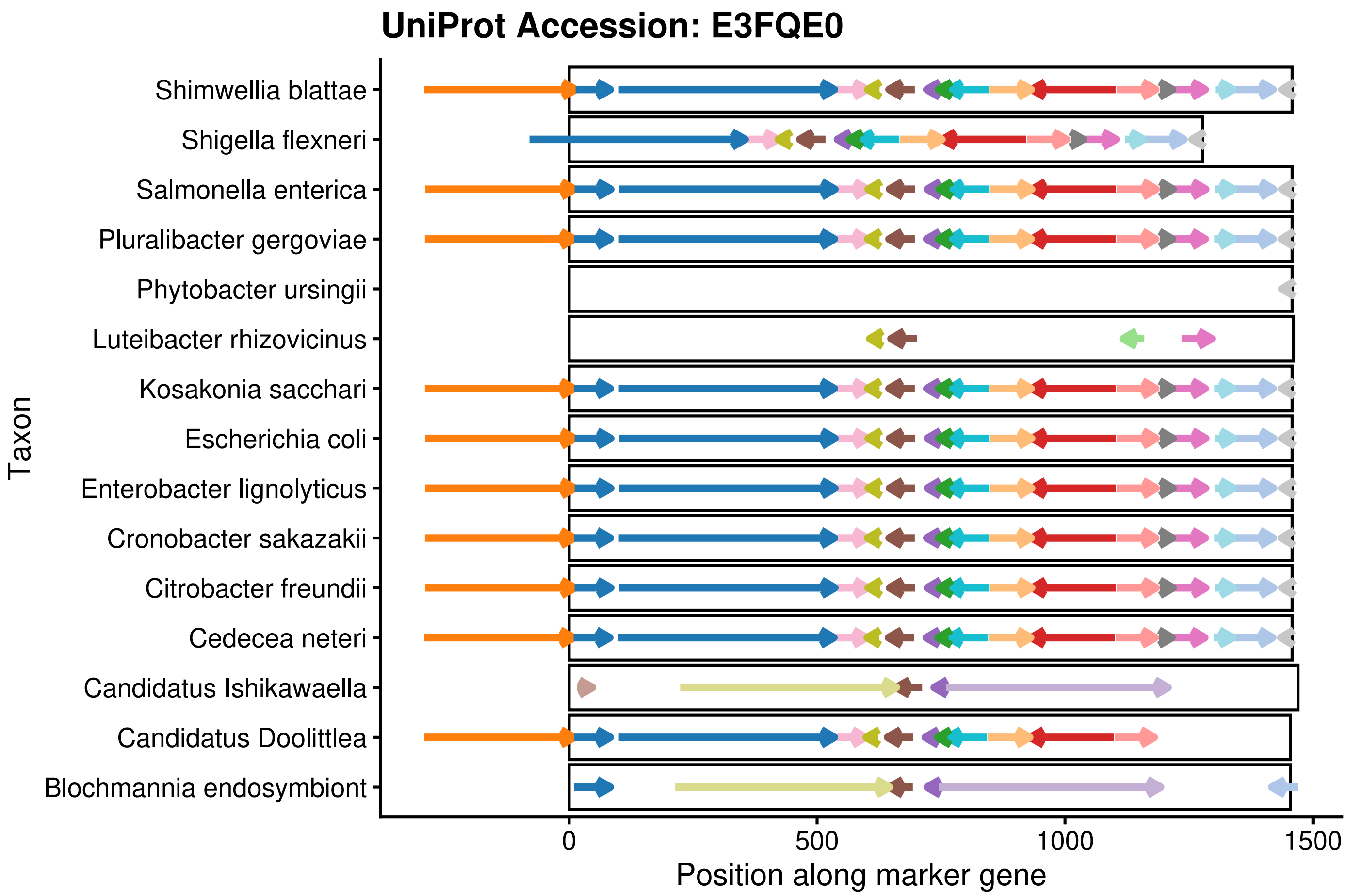
UniProt Accession: E3FQE0
Taxon
Shimwellia blattae
Shigella flexneri
Salmonella enterica
Pluralibacter gergoviae
Phytobacter ursingii
Luteibacter rhizovicinus
Kosakonia sacchari
Escherichia coli
Enterobacter lignolyticus
Cronobacter sakazakii
Citrobacter freundii
Cedecea neteri
Candidatus Ishikawaella
Candidatus Doolittlea
Blochmannia endosymbiont
0
500
1000
1500
Position along marker gene

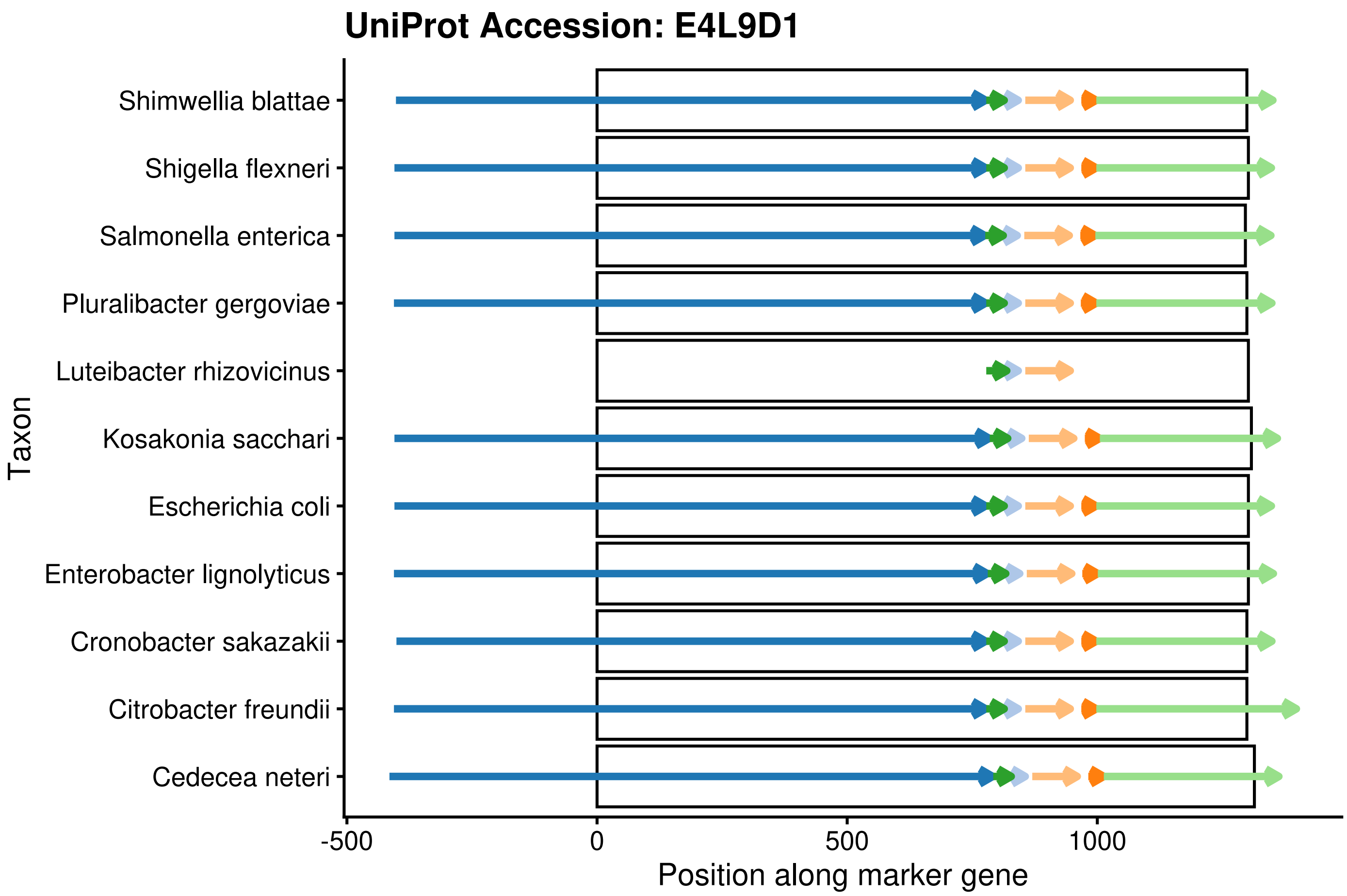
UniProt Accession: E4L9D1
Taxon
Shimwellia blattae
Shigella flexneri
Salmonella enterica
Pluralibacter gergoviae
Luteibacter rhizovicinus
Kosakonia sacchari
Escherichia coli
Enterobacter lignolyticus
Cronobacter sakazakii
Citrobacter freundii
Cedecea neteri
-500
0
500
1000
Position along marker gene

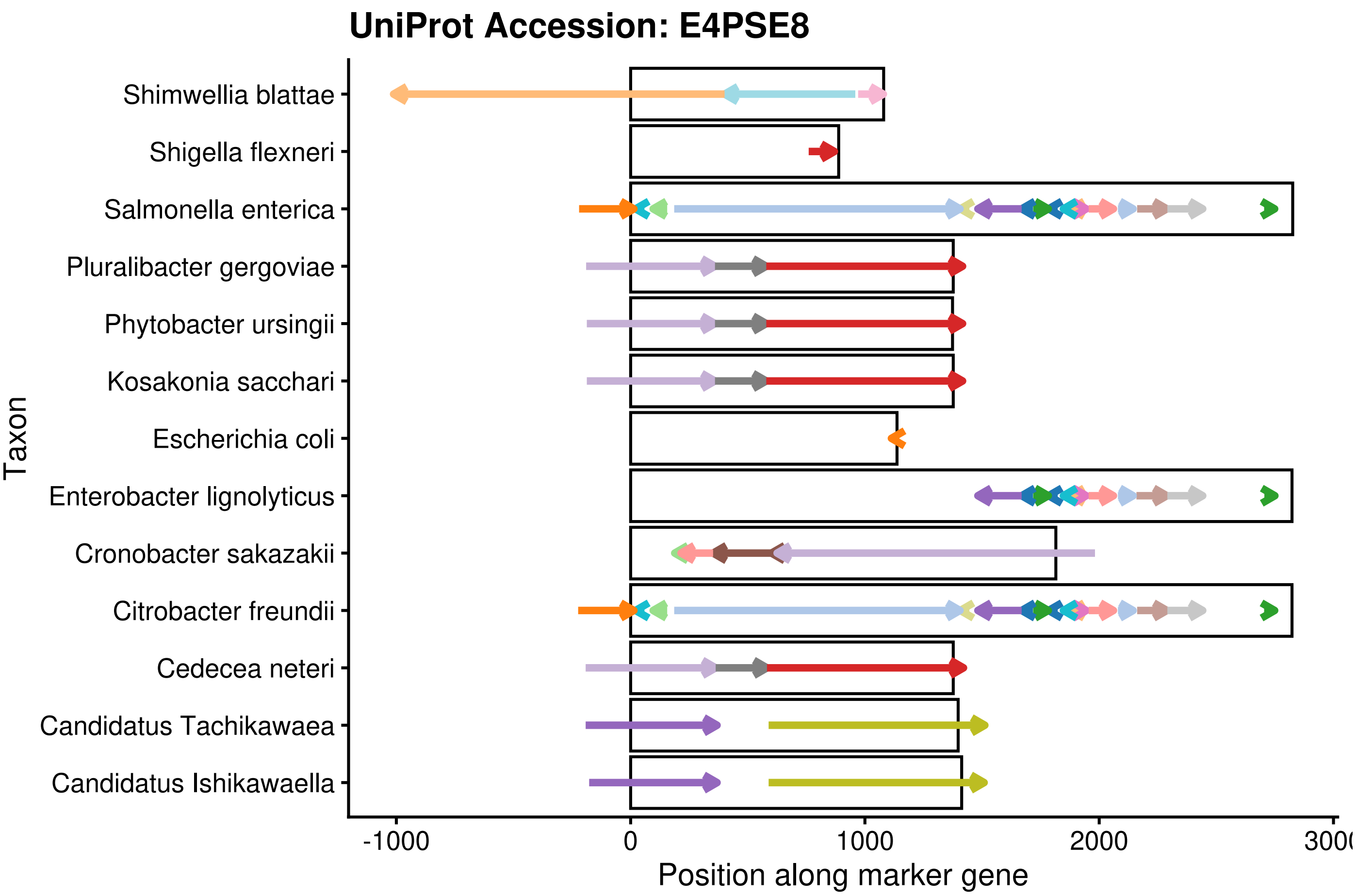
UniProt Accession: E4PSE8
Taxon
Shimwellia blattae
Shigella flexneri
Salmonella enterica
Pluralibacter gergoviae
Phytobacter ursingii
Kosakonia sacchari
Escherichia coli
Enterobacter lignolyticus
Cronobacter sakazakii
Citrobacter freundii
Cedecea neteri
Candidatus Tachikawaea
Candidatus Ishikawaella
-1000
0
1000
2000
Position along marker gene

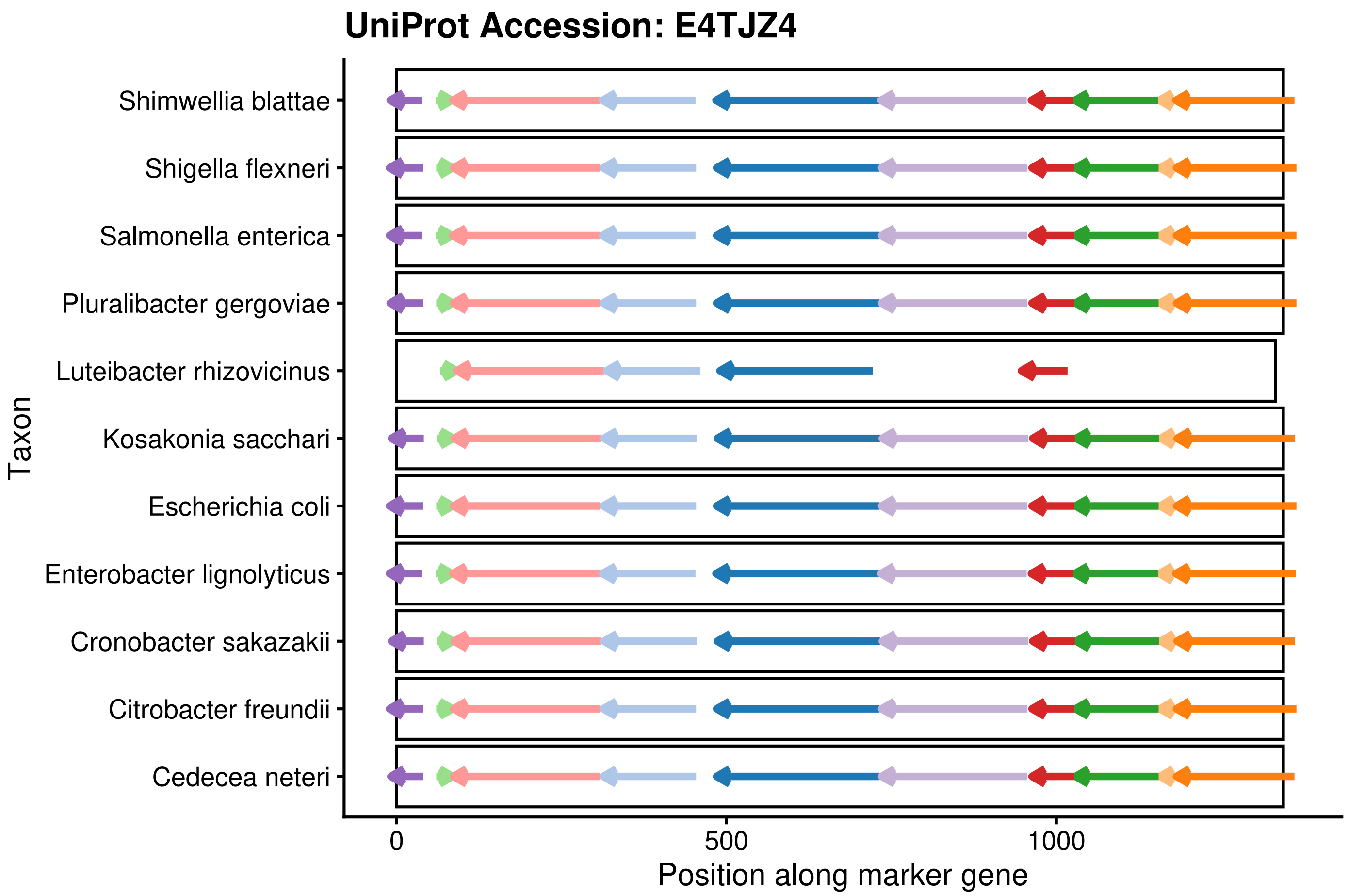
UniProt Accession: E4TJZ4
Shimwellia blattae
Shigella flexneri
Salmonella enterica
Pluralibacter gergoviae
Luteibacter rhizovicinus
Kosakonia sacchari
Escherichia coli
Enterobacter lignolyticus
Cronobacter sakazakii
Citrobacter freundii
Cedecea neteri
Taxon
0
500
1000
Position along marker gene

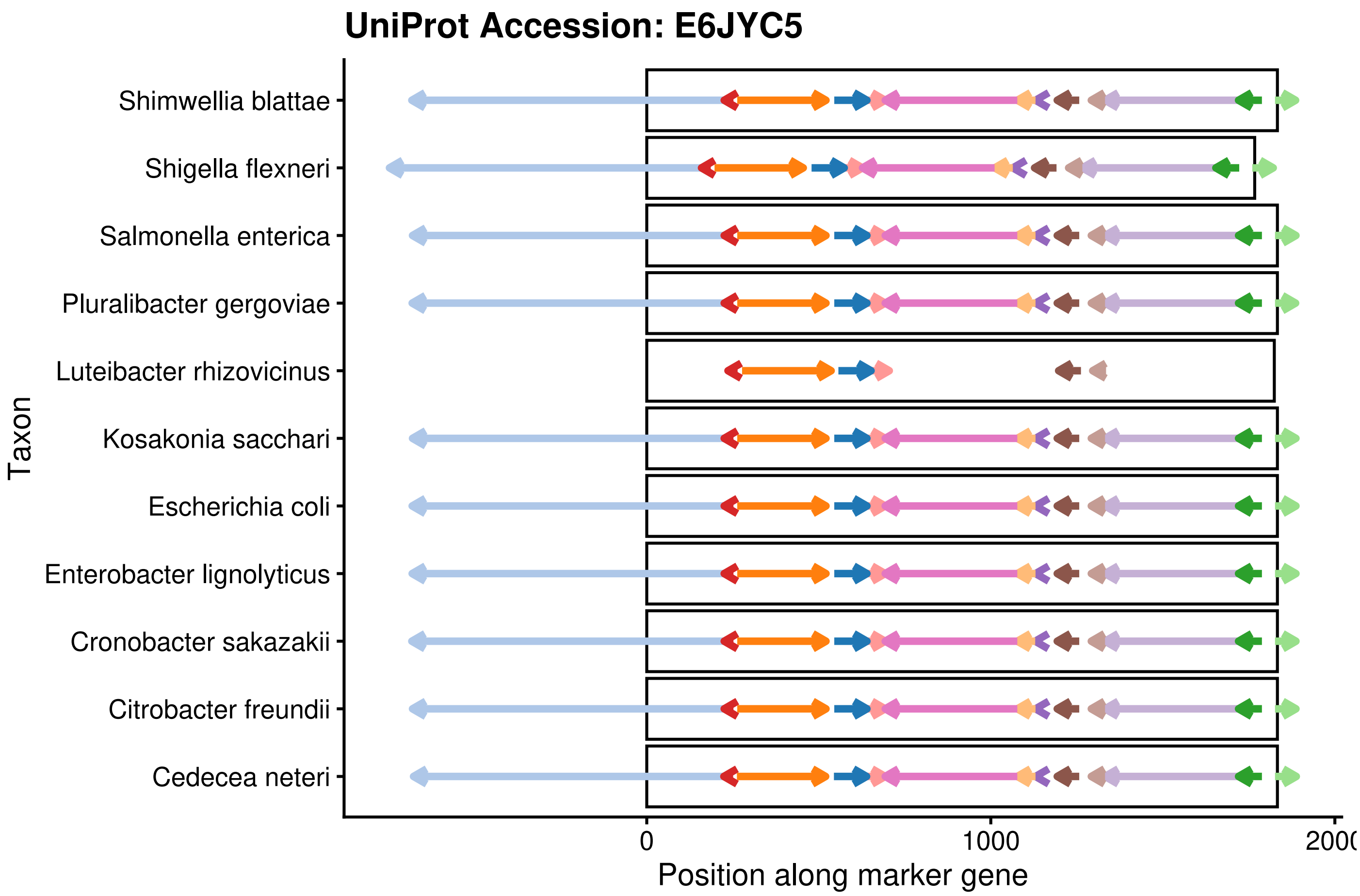

UniProt Accession: E6JYC5
Shimwellia blattae
Shigella flexneri
Salmonella enterica
Pluralibacter gergoviae
Luteibacter rhizovicinus
Kosakonia sacchari
Escherichia coli
Enterobacter lignolyticus
Cronobacter sakazakii
Citrobacter freundii
Cedecea neteri
Taxon
0
1000
2000
Position along marker gene

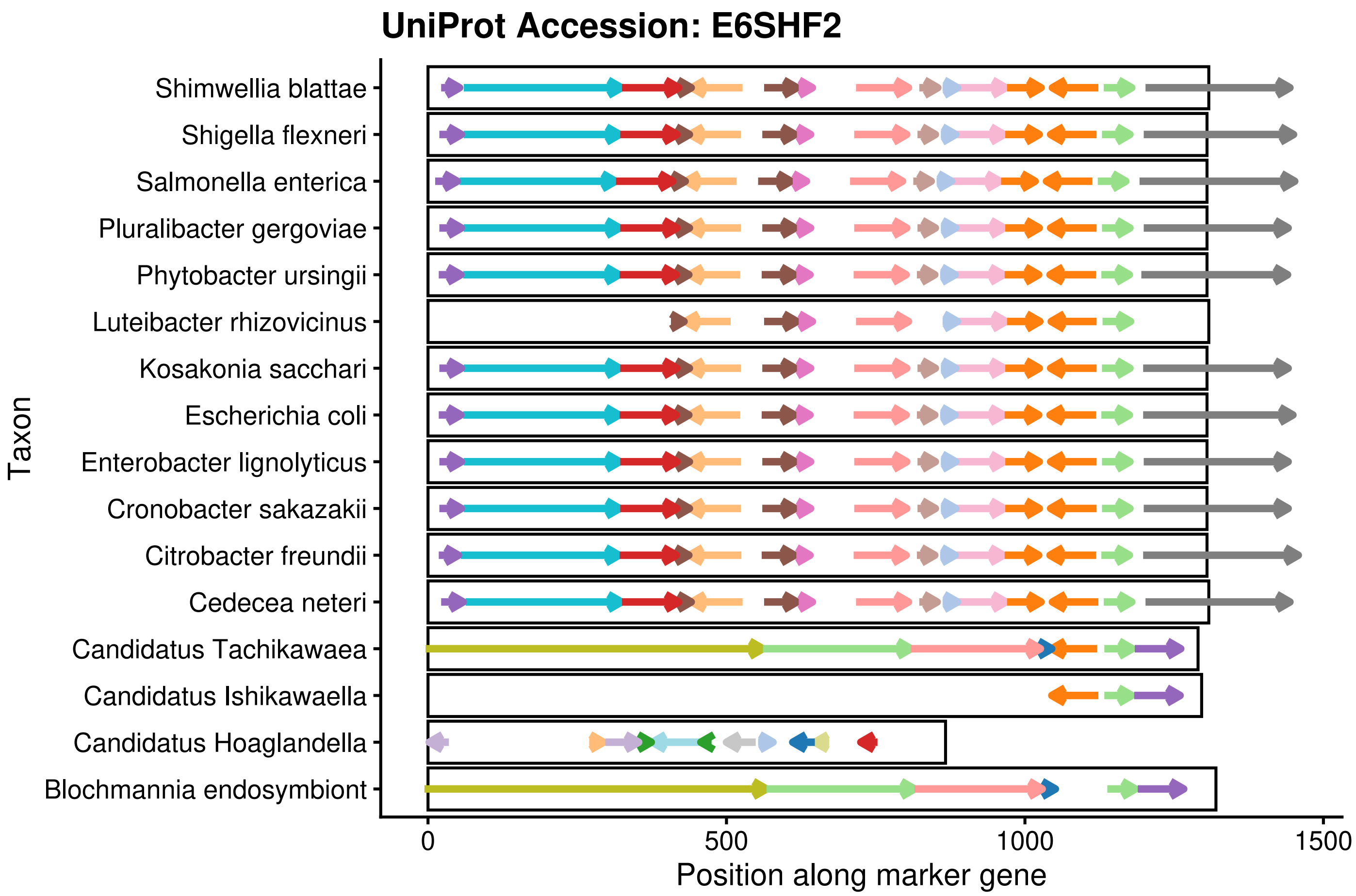
UniProt Accession: E6SHF2
Taxon
Shimwellia blattae
Shigella flexneri
Salmonella enterica
Pluralibacter gergoviae
Phytobacter ursingii
Luteibacter rhizovicinus
Kosakonia sacchari
Escherichia coli
Enterobacter lignolyticus
Cronobacter sakazakii
Citrobacter freundii
Cedecea neteri
Candidatus Tachikawaea
Candidatus Ishikawaella
Candidatus Hoaglandella
Blochmannia endosymbiont
0
500
1000
1500
Position along marker gene

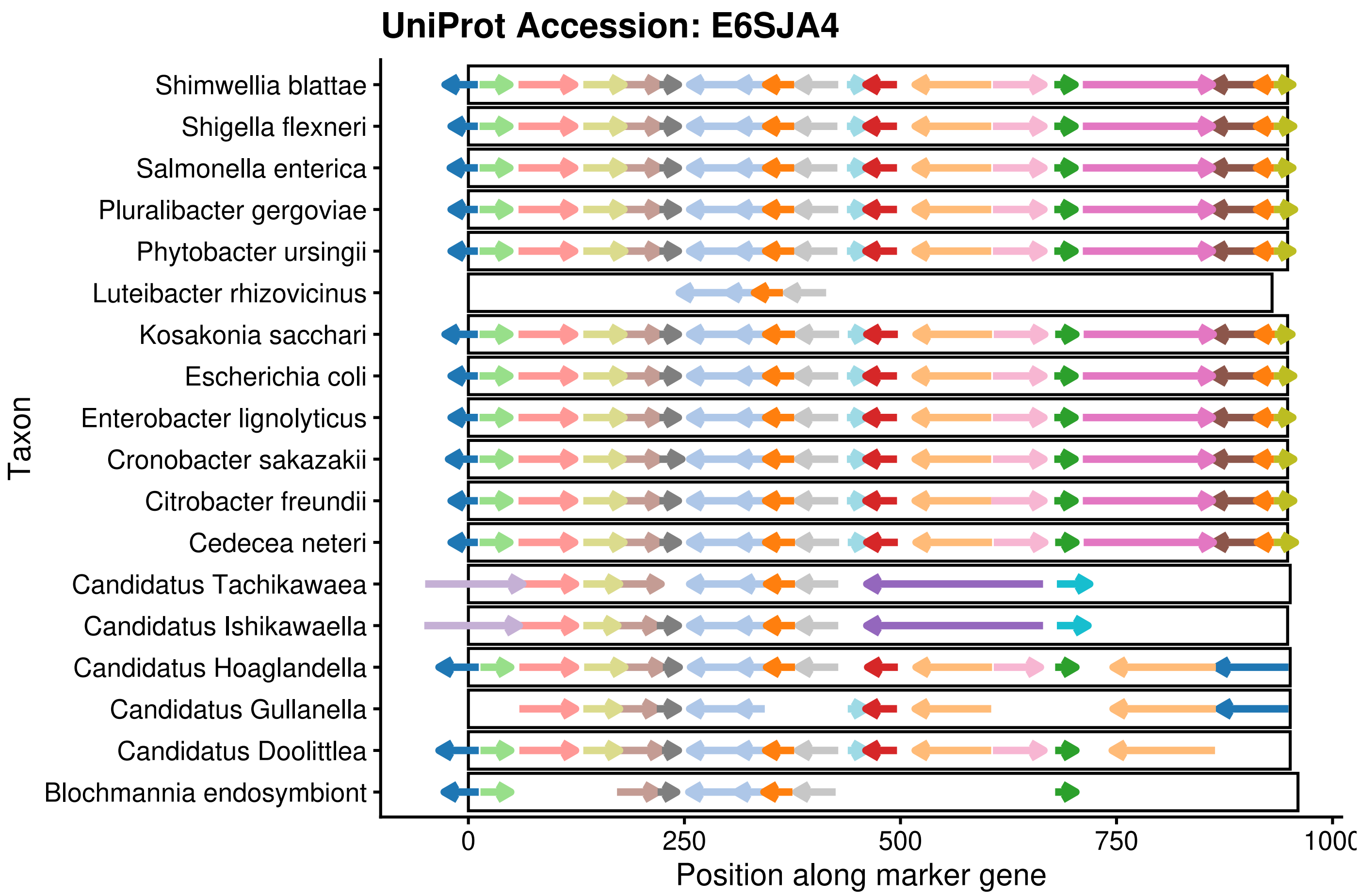

UniProt Accession: E6SJA4
Taxon
Shimwellia blattae
Shigella flexneri
Salmonella enterica
Pluralibacter gergoviae
Phytobacter ursingii
Luteibacter rhizovicinus
Kosakonia sacchari
Escherichia coli
Enterobacter lignolyticus
Cronobacter sakazakii
Citrobacter freundii
Cedecea neteri
Candidatus Tachikawaea
Candidatus Ishikawaella
Candidatus Hoaglandella
Candidatus Gullanella
Candidatus Doolittlea
Blochmannia endosymbiont
0
250
500
750
1000
Position along marker gene

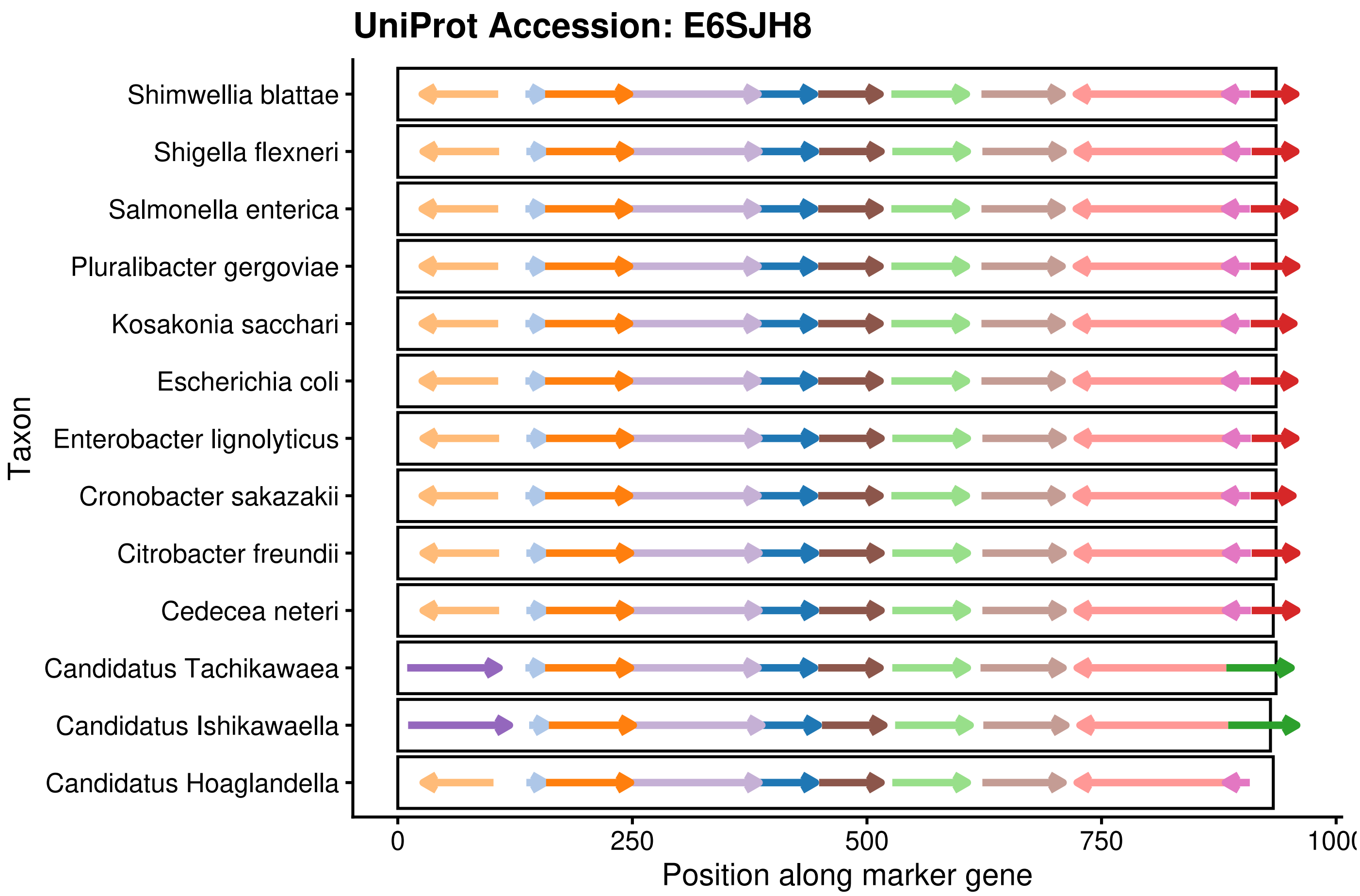
UniProt Accession: E6SJH8
Taxon
Shimwellia blattae
Shigella flexneri
Salmonella enterica
Pluralibacter gergoviae
Kosakonia sacchari
Escherichia coli
Enterobacter lignolyticus
Cronobacter sakazakii
Citrobacter freundii
Cedecea neteri
Candidatus Tachikawaea
Candidatus Ishikawaella
Candidatus Hoaglandella
0
250
500
750
1000
Position along marker gene

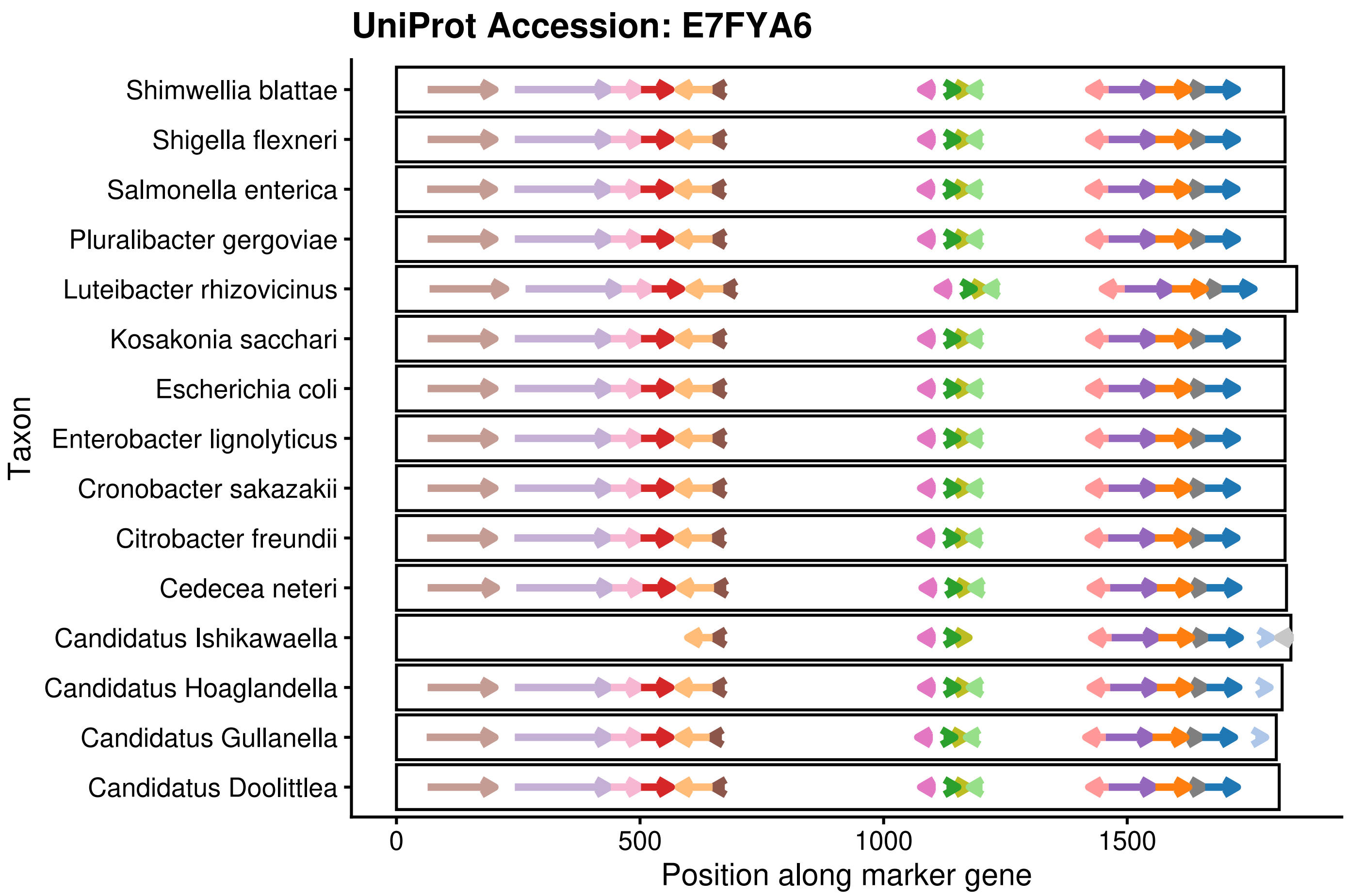

UniProt Accession: E7FYA6
Taxon
Shimwellia blattae
Shigella flexneri
Salmonella enterica
Pluralibacter gergoviae
Luteibacter rhizovicinus
Kosakonia sacchari
Escherichia coli
Enterobacter lignolyticus
Cronobacter sakazakii
Citrobacter freundii
Cedecea neteri
Candidatus Ishikawaella
Candidatus Hoaglandella
Candidatus Gullanella
Candidatus Doolittlea
0
500
1000
1500
Position along marker gene

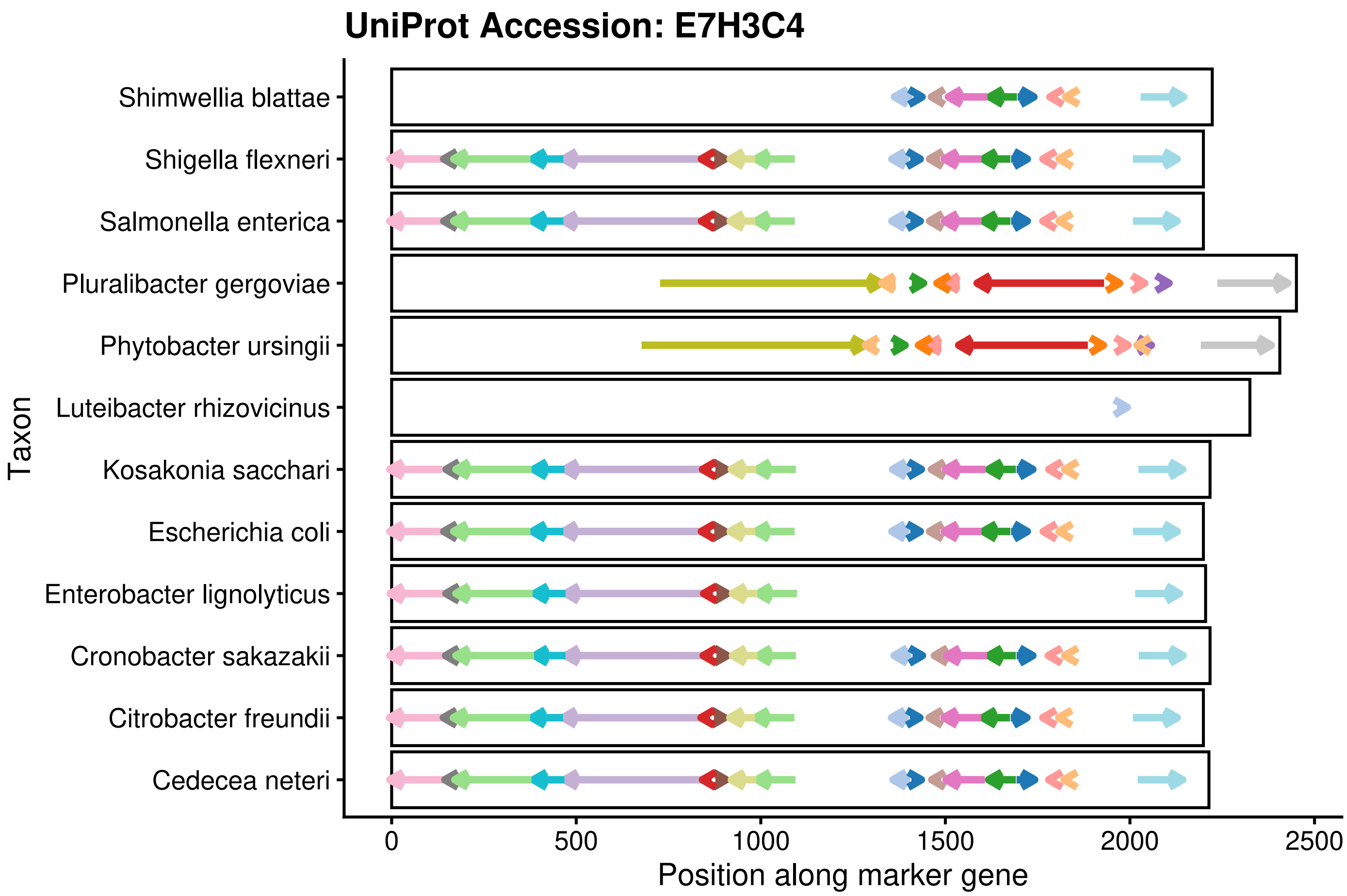
UniProt Accession: E7H3C4
Shimwellia blattae
Shigella flexneri
Salmonella enterica
Pluralibacter gergoviae
Phytobacter ursingii
Luteibacter rhizovicinus
Kosakonia sacchari
Escherichia coli
Enterobacter lignolyticus
Cronobacter sakazakii
Citrobacter freundii
Cedecea neteri
Taxon
0
500
1000
1500
2000
2500
Position along marker gene

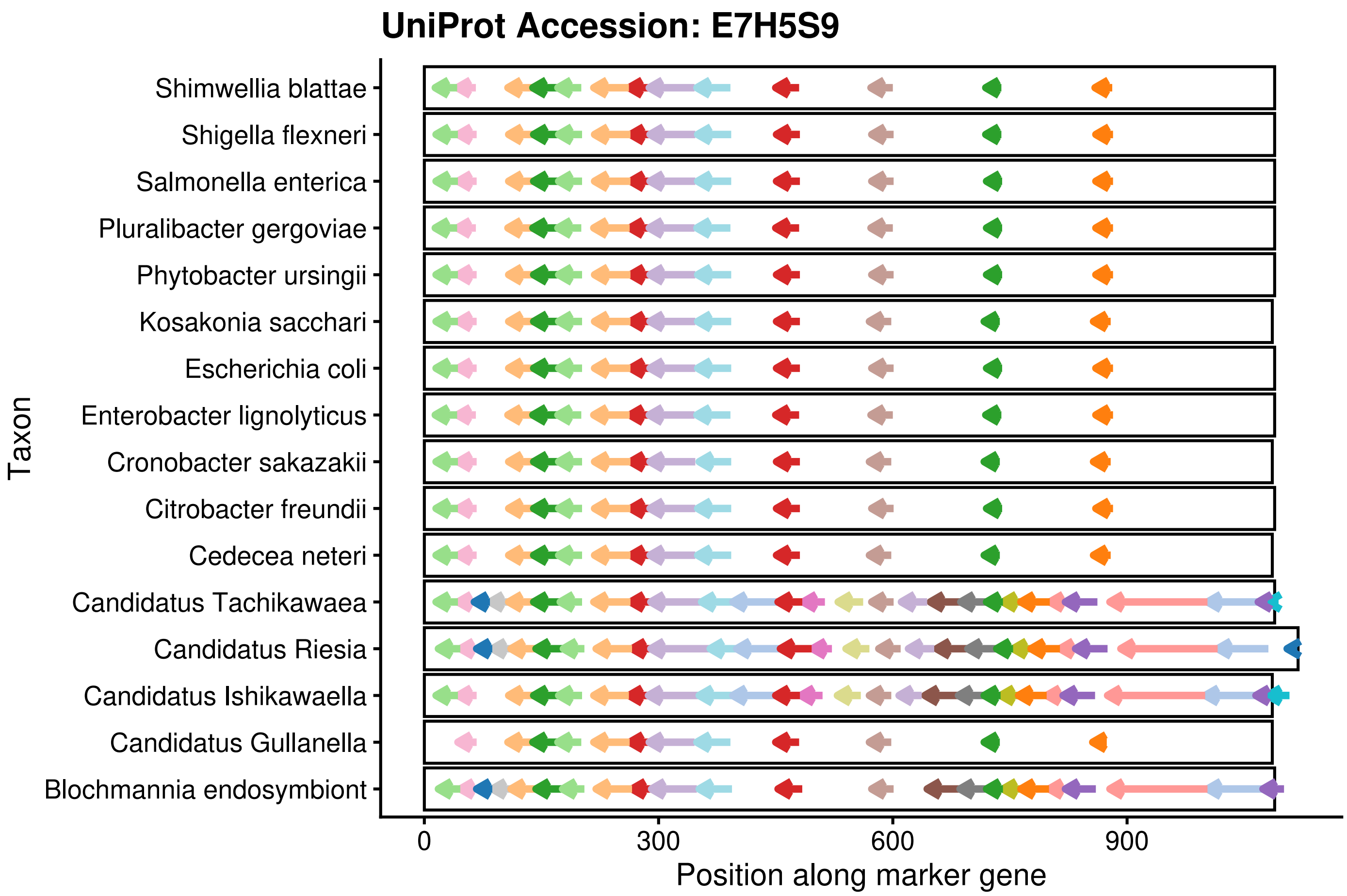

UniProt Accession: E7H5S9
Taxon
Shimwellia blattae
Shigella flexneri
Salmonella enterica
Pluralibacter gergoviae
Phytobacter ursingii
Kosakonia sacchari
Escherichia coli
Enterobacter lignolyticus
Cronobacter sakazakii
Citrobacter freundii
Cedecea neteri
Candidatus Tachikawaea
Candidatus Riesia
Candidatus Ishikawaella
Candidatus Gullanella
Blochmannia endosymbiont
0
300
600
900
Position along marker gene

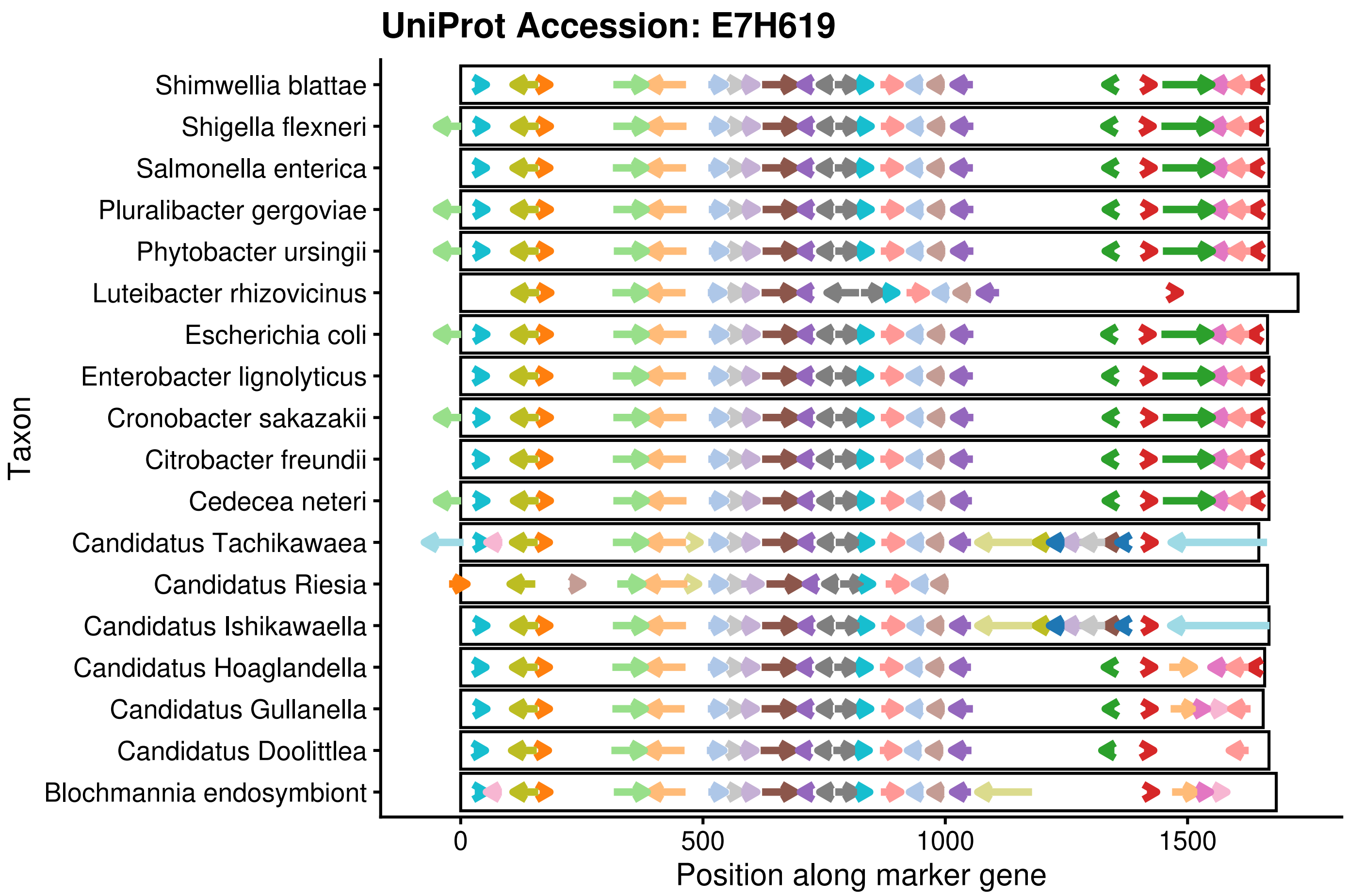

UniProt Accession: E7H619
Shimwellia blattae
Shigella flexneri
Salmonella enterica
Pluralibacter gergoviae
Phytobacter ursingii
Luteibacter rhizovicinus
Escherichia coli
Enterobacter lignolyticus
Cronobacter sakazakii
Citrobacter freundii
Cedecea neteri
Candidatus Tachikawaea
Candidatus Riesia
Candidatus Ishikawaella
Candidatus Hoaglandella
Candidatus Gullanella
Candidatus Doolittlea
Blochmannia endosymbiont
Taxon
0
500
1000
1500
Position along marker gene

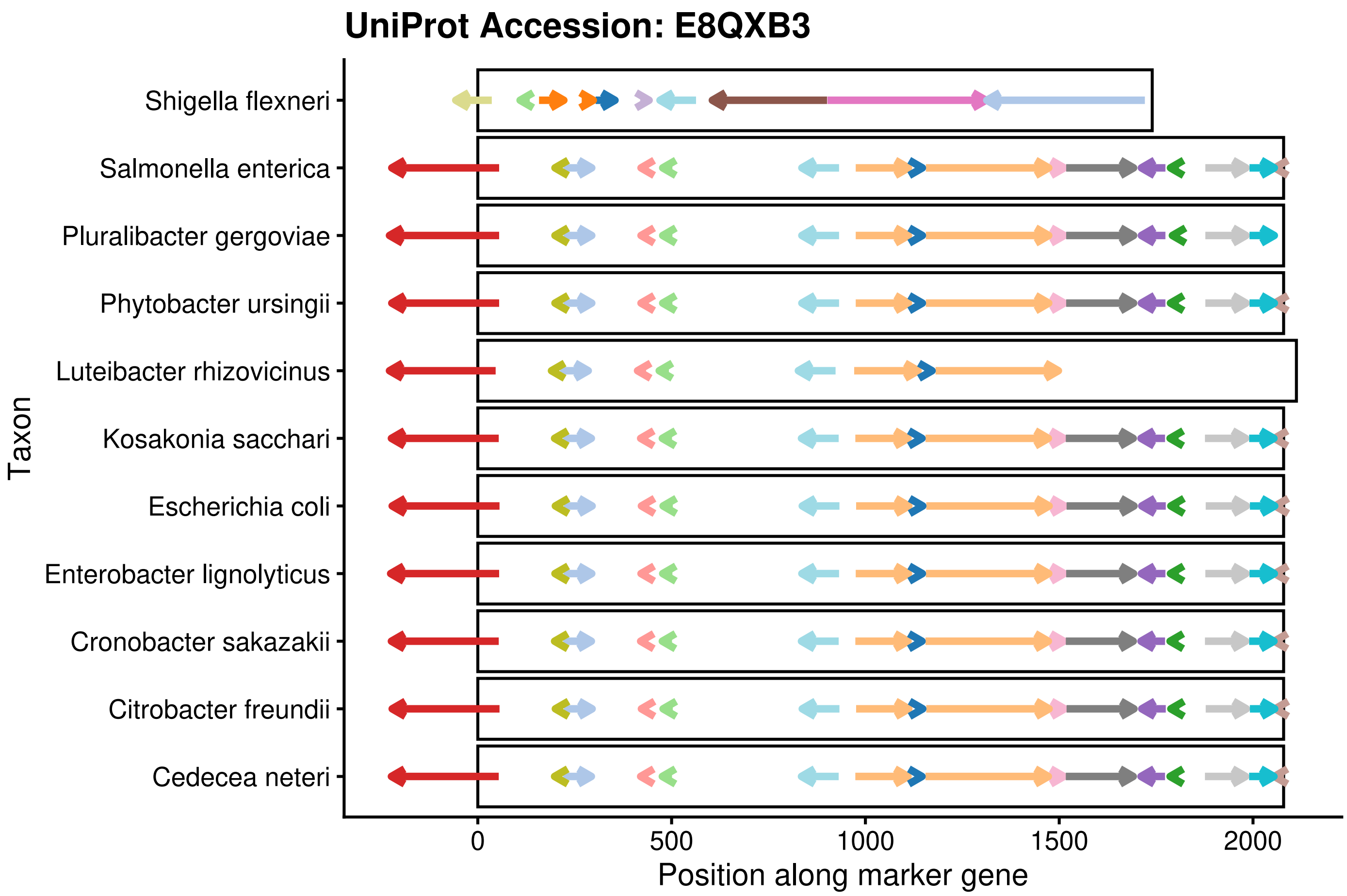

UniProt Accession: E8QXB3
Taxon
Shigella flexneri
Salmonella enterica
Pluralibacter gergoviae
Phytobacter ursingii
Luteibacter rhizovicinus
Kosakonia sacchari
Escherichia coli
Enterobacter lignolyticus
Cronobacter sakazakii
Citrobacter freundii
Cedecea neteri
0
500
1000
1500
2000
Position along marker gene

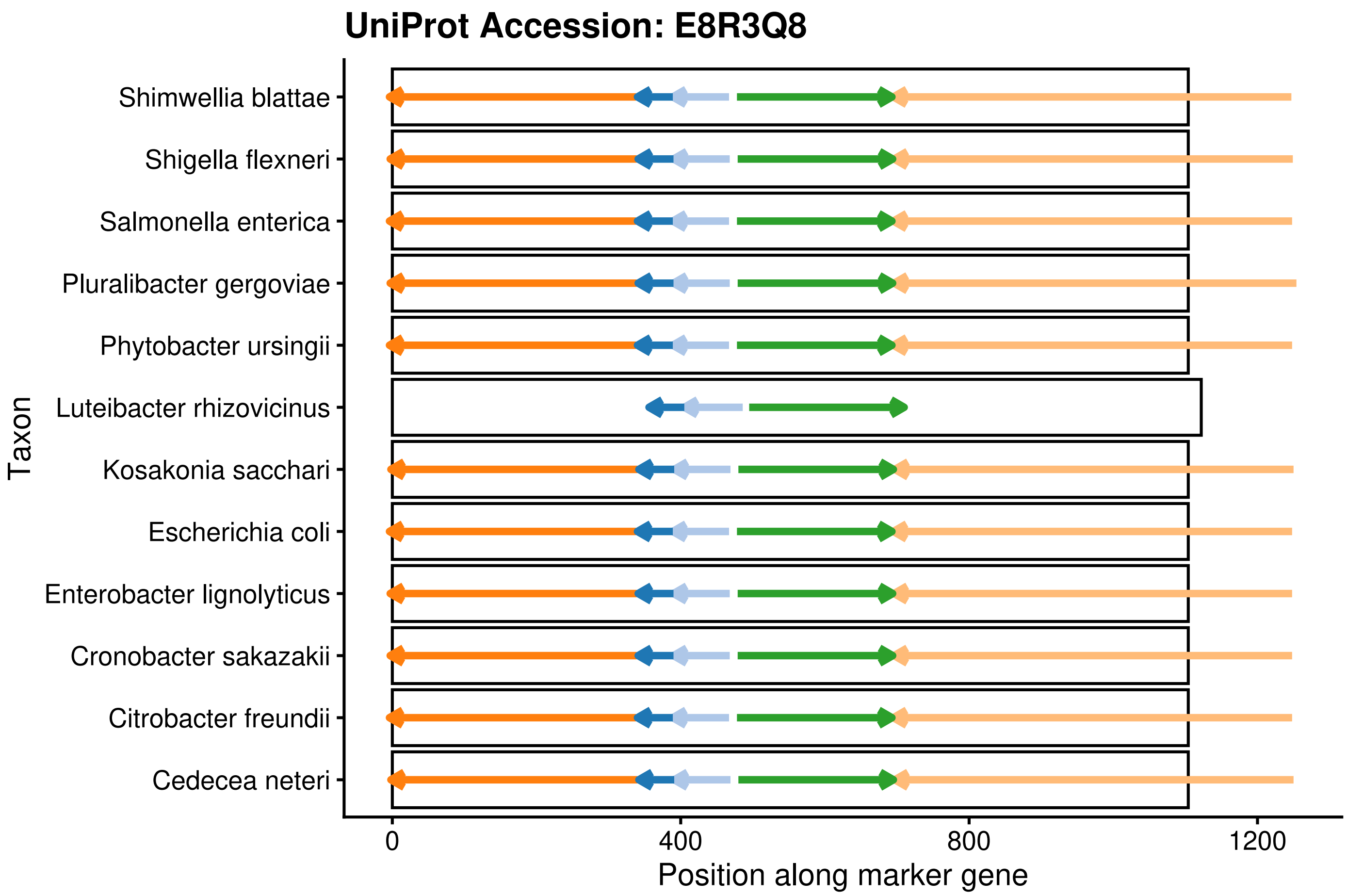

UniProt Accession: E8R3Q8
Taxon
Shimwellia blattae
Shigella flexneri
Salmonella enterica
Pluralibacter gergoviae
Phytobacter ursingii
Luteibacter rhizovicinus
Kosakonia sacchari
Escherichia coli
Enterobacter lignolyticus
Cronobacter sakazakii
Citrobacter freundii
Cedecea neteri
0
400
800
1200
Position along marker gene

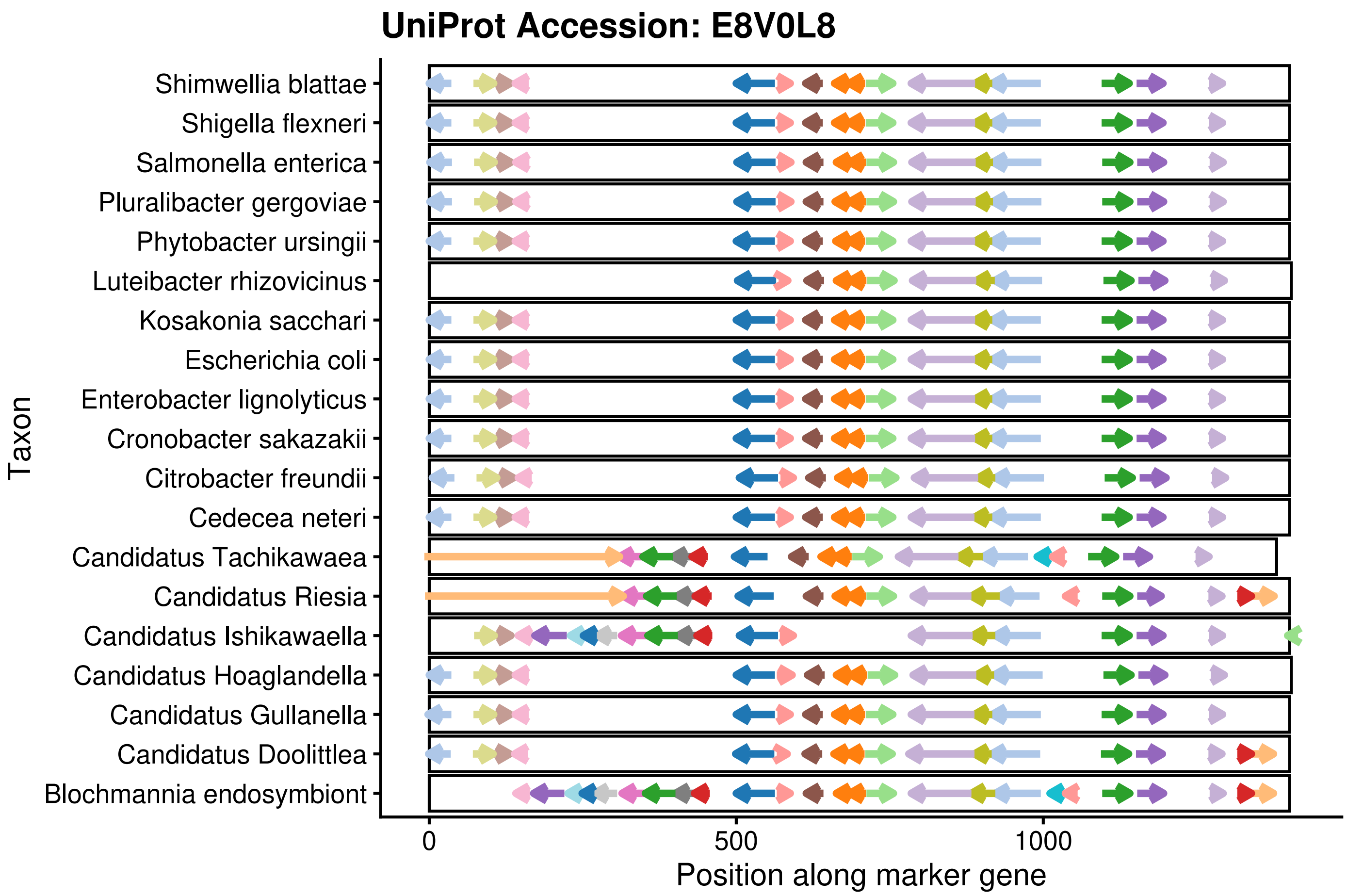
UniProt Accession: E8V0L8
Taxon
Shimwellia blattae
Shigella flexneri
Salmonella enterica
Pluralibacter gergoviae
Phytobacter ursingii
Luteibacter rhizovicinus
Kosakonia sacchari
Escherichia coli
Enterobacter lignolyticus
Cronobacter sakazakii
Citrobacter freundii
Cedecea neteri
Candidatus Tachikawaea
Candidatus Riesia
Candidatus Ishikawaella
Candidatus Hoaglandella
Candidatus Gullanella
Candidatus Doolittlea
Blochmannia endosymbiont
0
500
1000
Position along marker gene

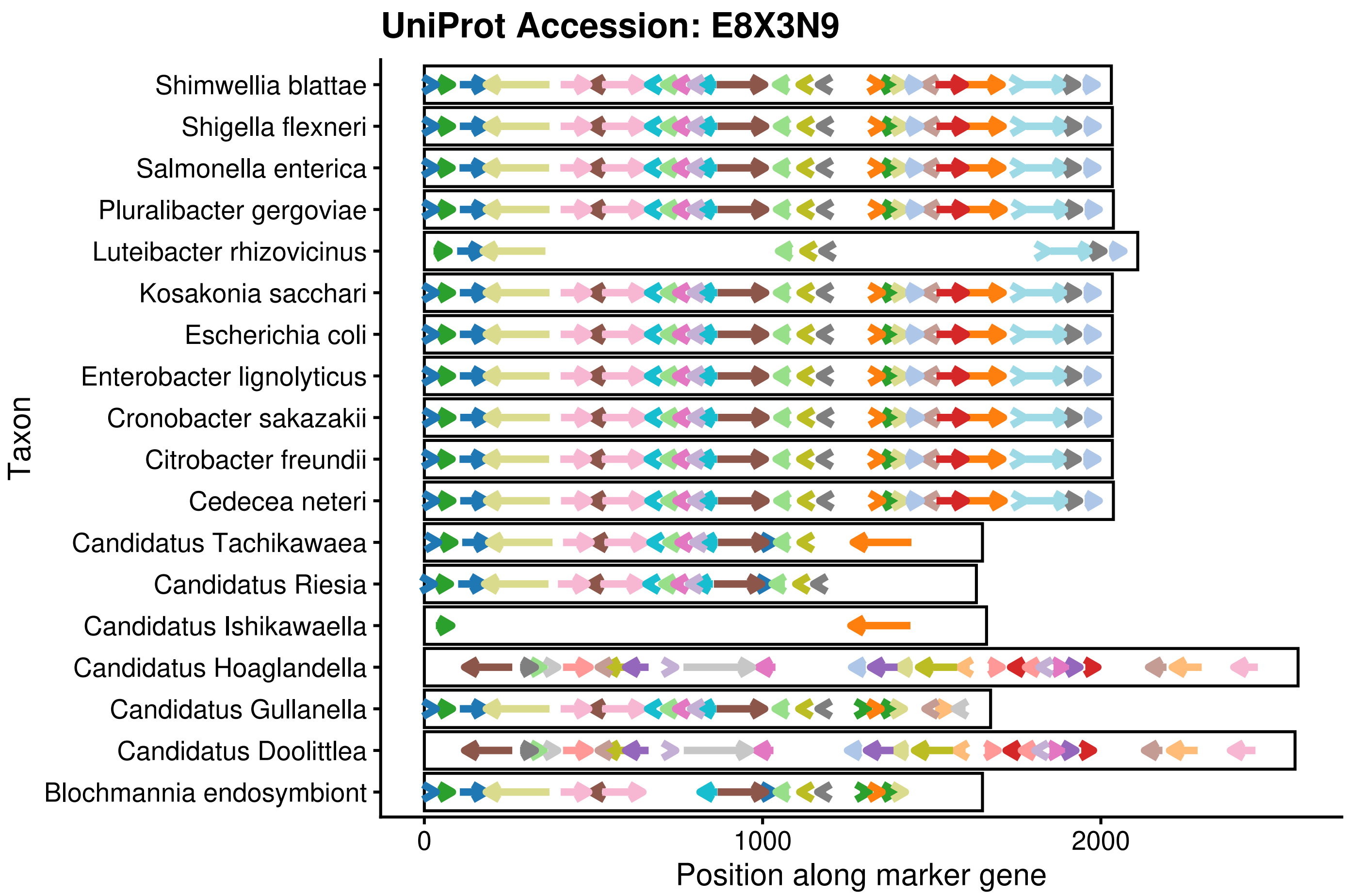
UniProt Accession: E8X3N9
Taxon
Shimwellia blattae
Shigella flexneri
Salmonella enterica
Pluralibacter gergoviae
Luteibacter rhizovicinus
Kosakonia sacchari
Escherichia coli
Enterobacter lignolyticus
Cronobacter sakazakii
Citrobacter freundii
Cedecea neteri
Candidatus Tachikawaea
Candidatus Riesia
Candidatus Ishikawaella
Candidatus Hoaglandella
Candidatus Gullanella
Candidatus Doolittlea
Blochmannia endosymbiont
0
1000
2000
Position along marker gene

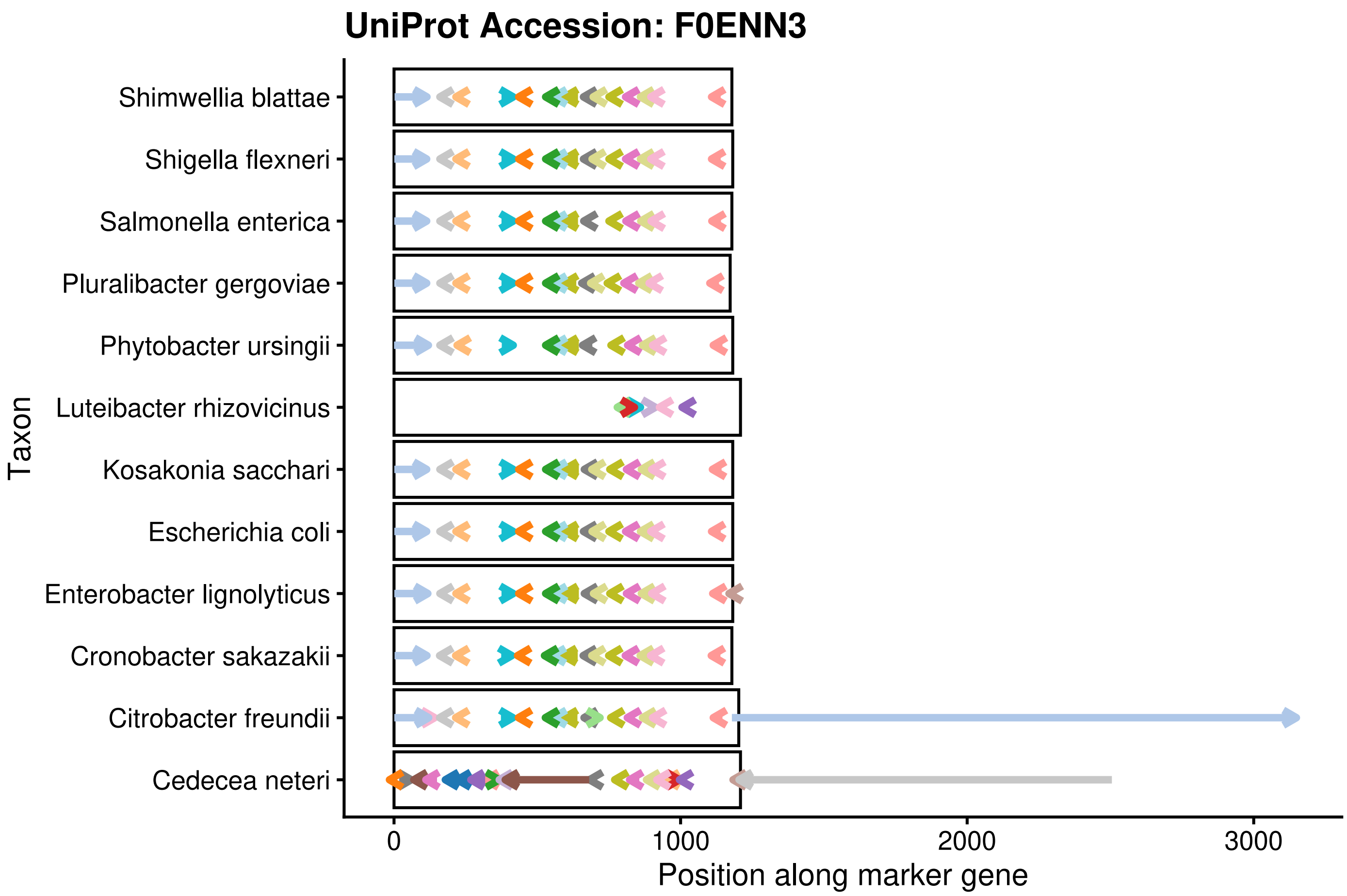

UniProt Accession: F0ENN3
Shimwellia blattae
Shigella flexneri
Salmonella enterica
Pluralibacter gergoviae
Phytobacter ursingii
Luteibacter rhizovicinus
Kosakonia sacchari
Escherichia coli
Enterobacter lignolyticus
Cronobacter sakazakii
Citrobacter freundii
Cedecea neteri
Taxon
0
1000
2000
3000
Position along marker gene

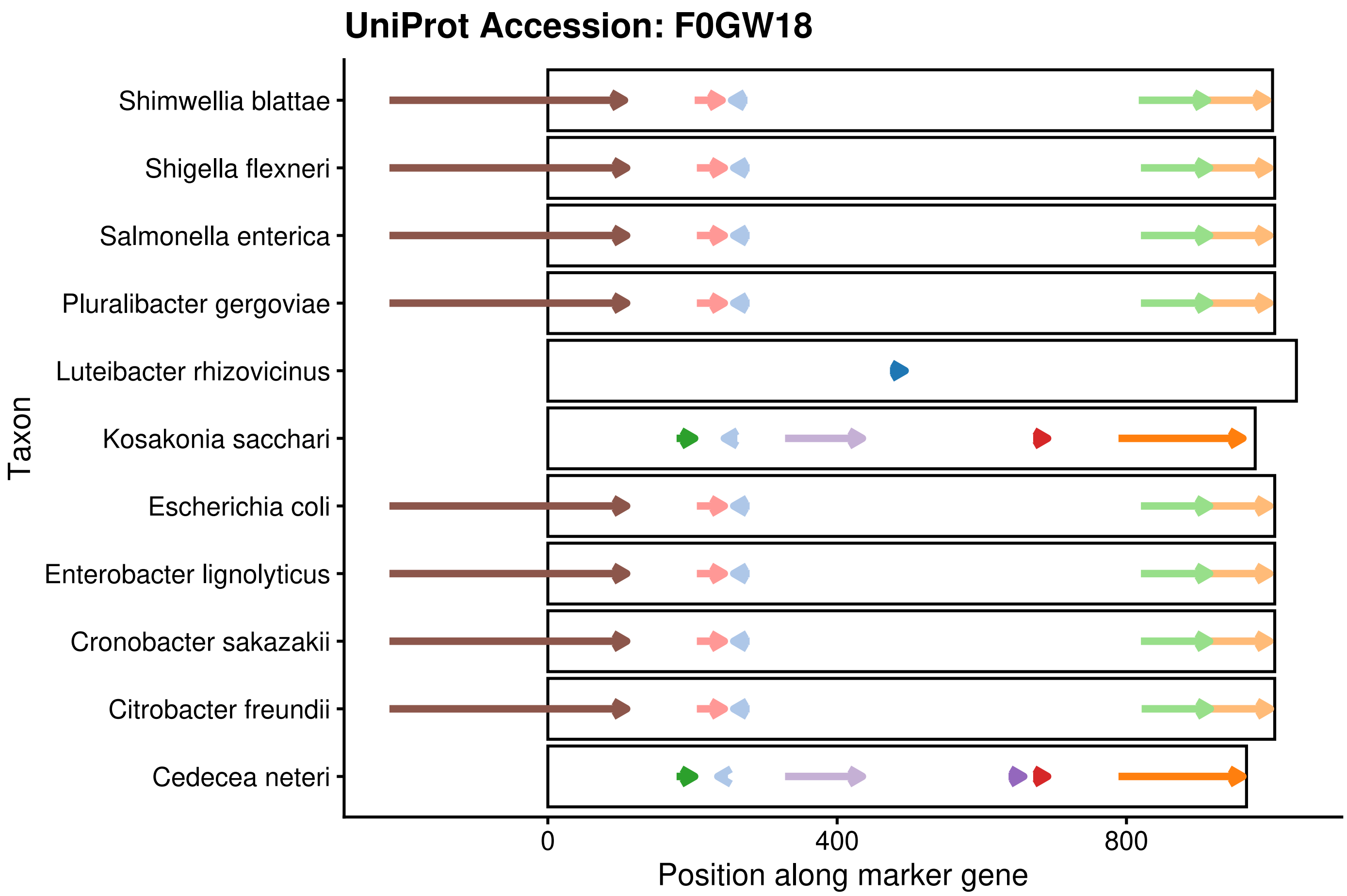
UniProt Accession: F0GW18
Shimwellia blattae
Shigella flexneri
Salmonella enterica
Pluralibacter gergoviae
Luteibacter rhizovicinus
Kosakonia sacchari
Escherichia coli
Enterobacter lignolyticus
Cronobacter sakazakii
Citrobacter freundii
Cedecea neteri
Taxon
0
400
800
Position along marker gene

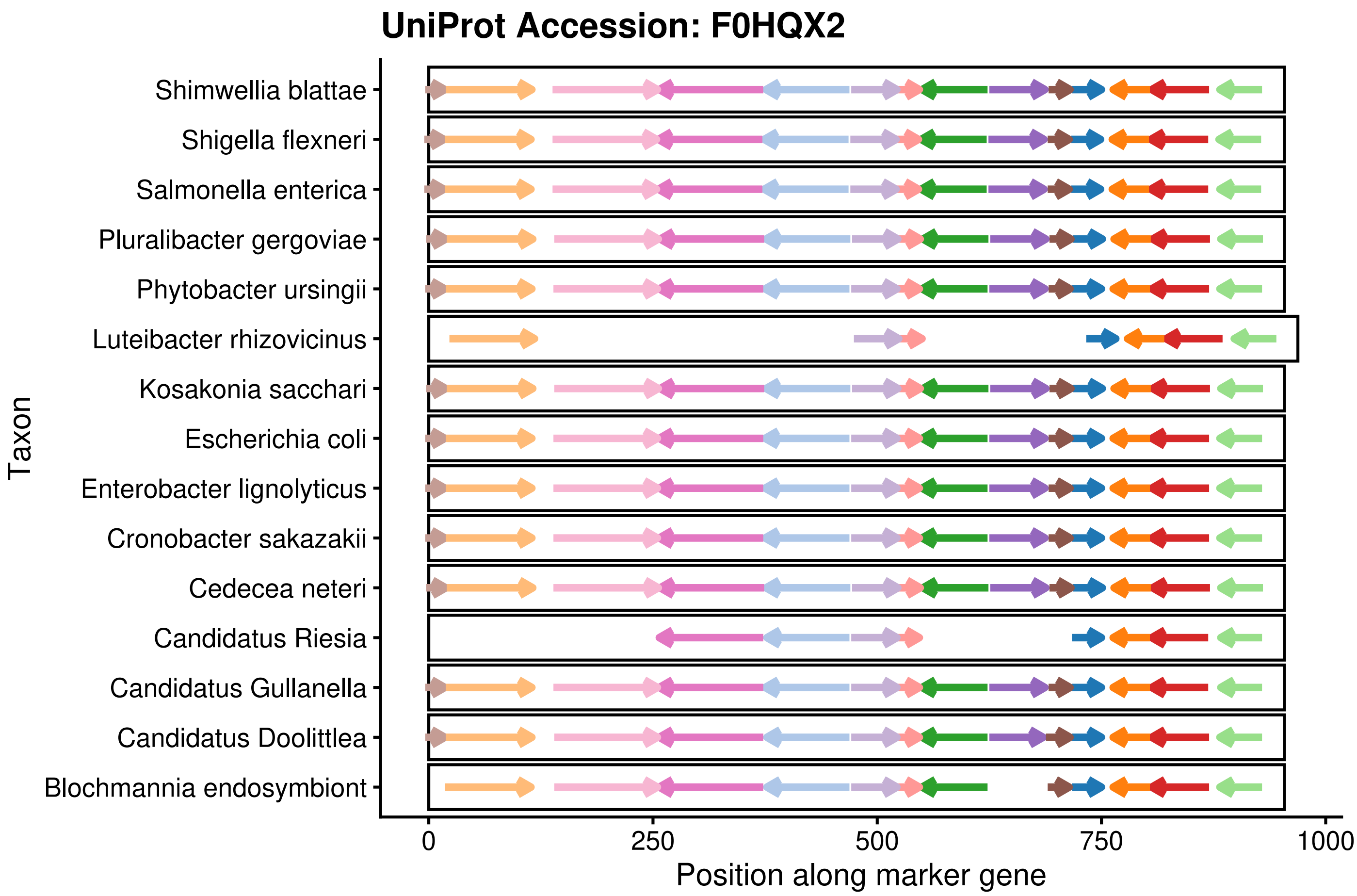

UniProt Accession: F0HQX2
Shimwellia blattae
Shigella flexneri
Salmonella enterica
Pluralibacter gergoviae
Phytobacter ursingii
Luteibacter rhizovicinus
Kosakonia sacchari
Escherichia coli
Enterobacter lignolyticus
Cronobacter sakazakii
Cedecea neteri
Candidatus Riesia
Candidatus Gullanella
Candidatus Doolittlea
Blochmannia endosymbiont
Taxon
0
250
500
750
1000
Position along marker gene

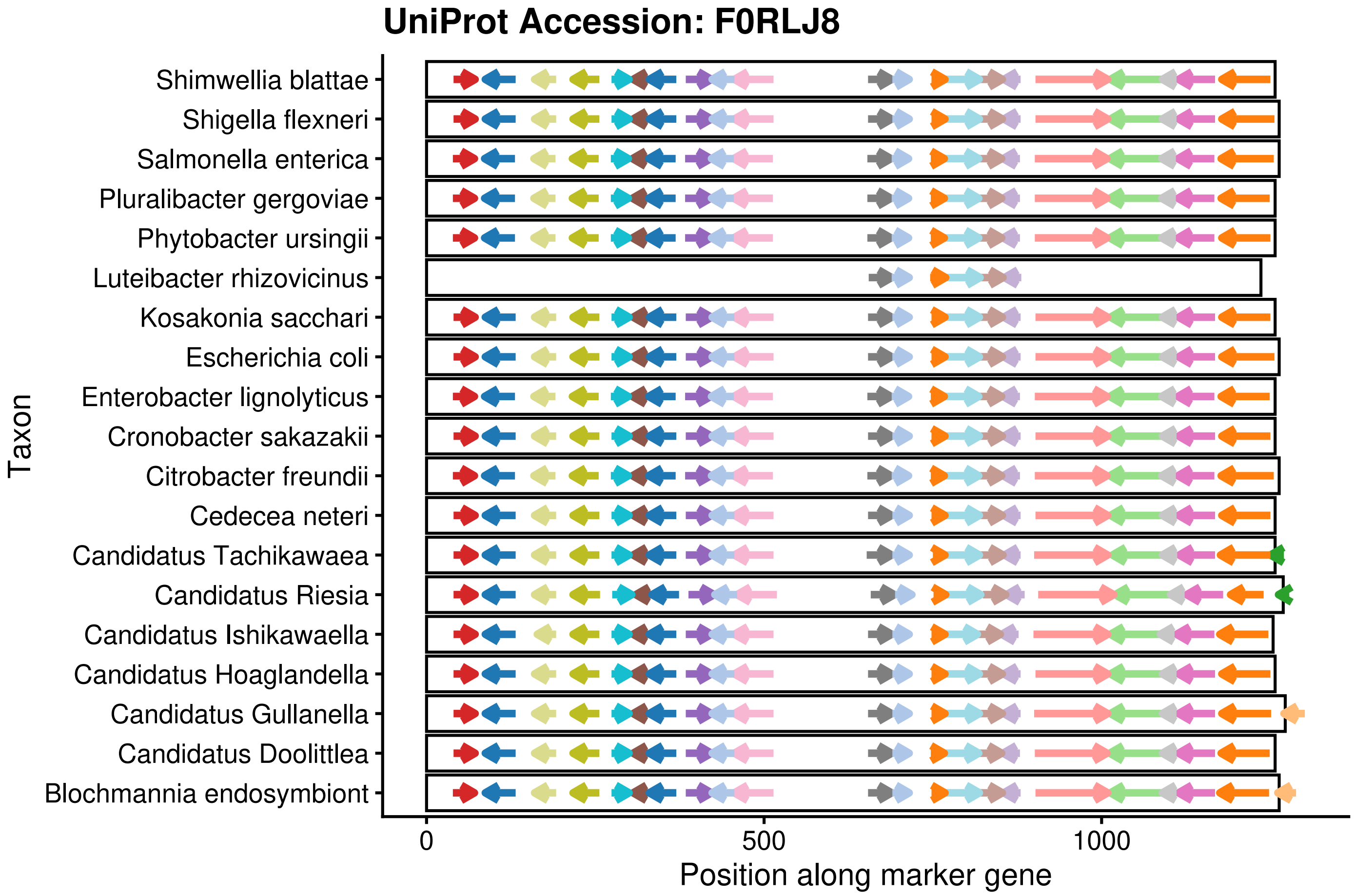
UniProt Accession: F0RLJ8
Taxon
Shimwellia blattae
Shigella flexneri
Salmonella enterica
Pluralibacter gergoviae
Phytobacter ursingii
Luteibacter rhizovicinus
Kosakonia sacchari
Escherichia coli
Enterobacter lignolyticus
Cronobacter sakazakii
Citrobacter freundii
Cedecea neteri
Candidatus Tachikawaea
Candidatus Riesia
Candidatus Ishikawaella
Candidatus Hoaglandella
Candidatus Gullanella
Candidatus Doolittlea
Blochmannia endosymbiont
0
500
1000
Position along marker gene

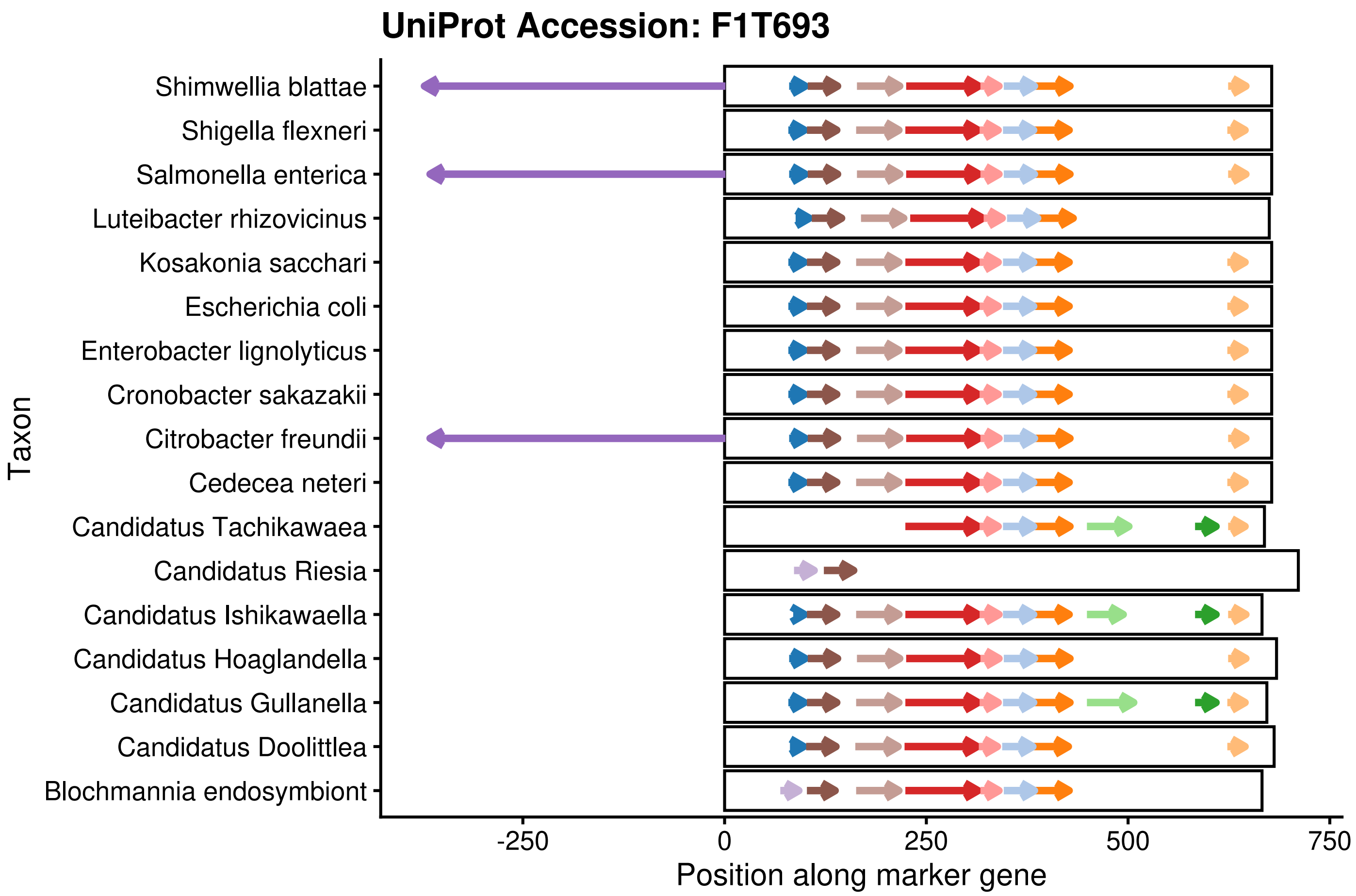

UniProt Accession: F1T693
Shimwellia blattae
Shigella flexneri
Salmonella enterica
Luteibacter rhizovicinus
Kosakonia sacchari
Escherichia coli
Enterobacter lignolyticus
Cronobacter sakazakii
Citrobacter freundii
Cedecea neteri
Candidatus Tachikawaea
Candidatus Riesia
Candidatus Ishikawaella
Candidatus Hoaglandella
Candidatus Gullanella
Candidatus Doolittlea
Blochmannia endosymbiont
Taxon
-250
0
250
500
750
Position along marker gene

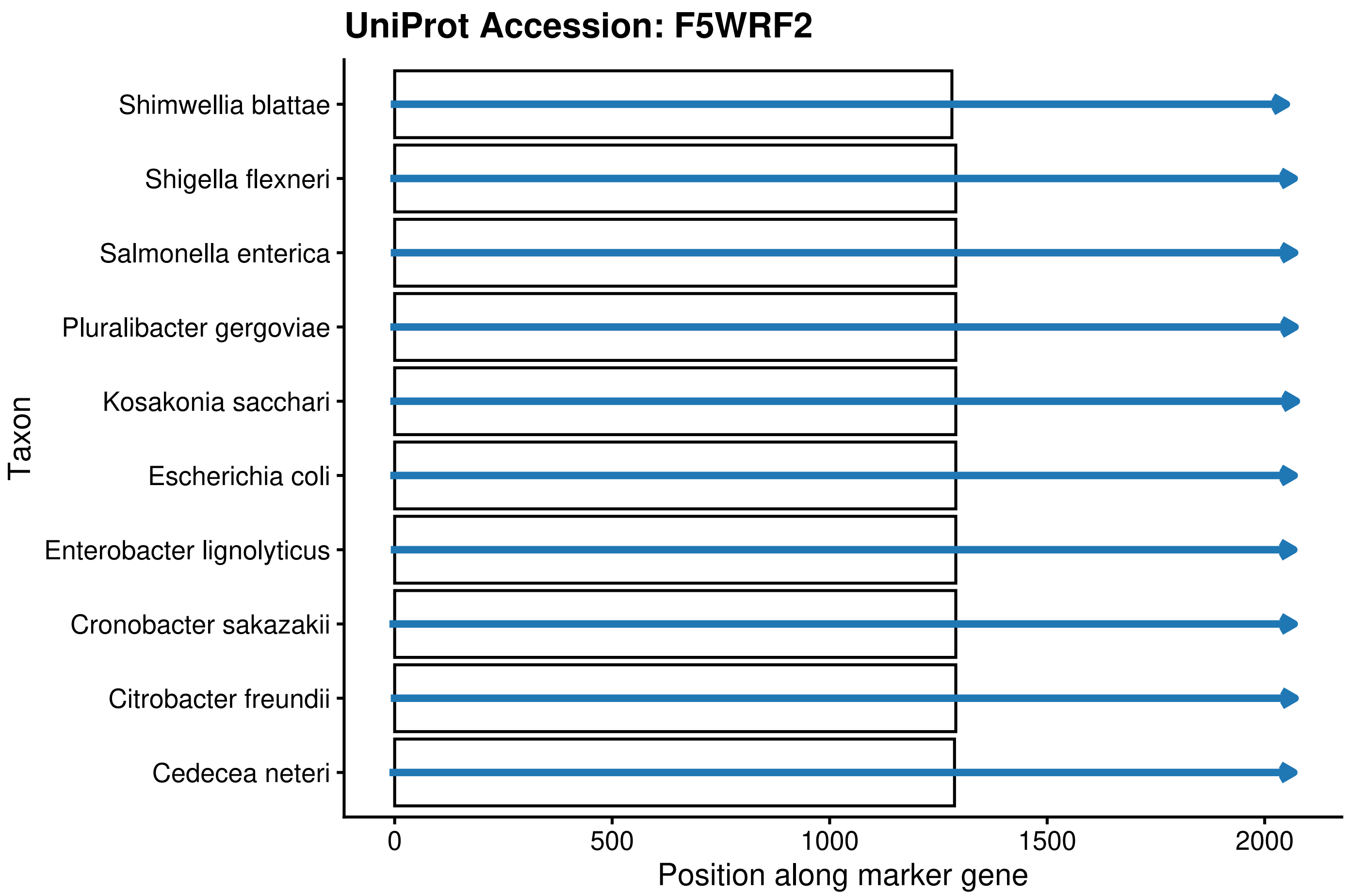
UniProt Accession: F5WRF2
Shimwellia blattae
Shigella flexneri
Salmonella enterica
Pluralibacter gergoviae
Kosakonia sacchari
Escherichia coli
Enterobacter lignolyticus
Cronobacter sakazakii
Citrobacter freundii
Cedecea neteri
Taxon
0
500
1000
1500
2000
Position along marker gene

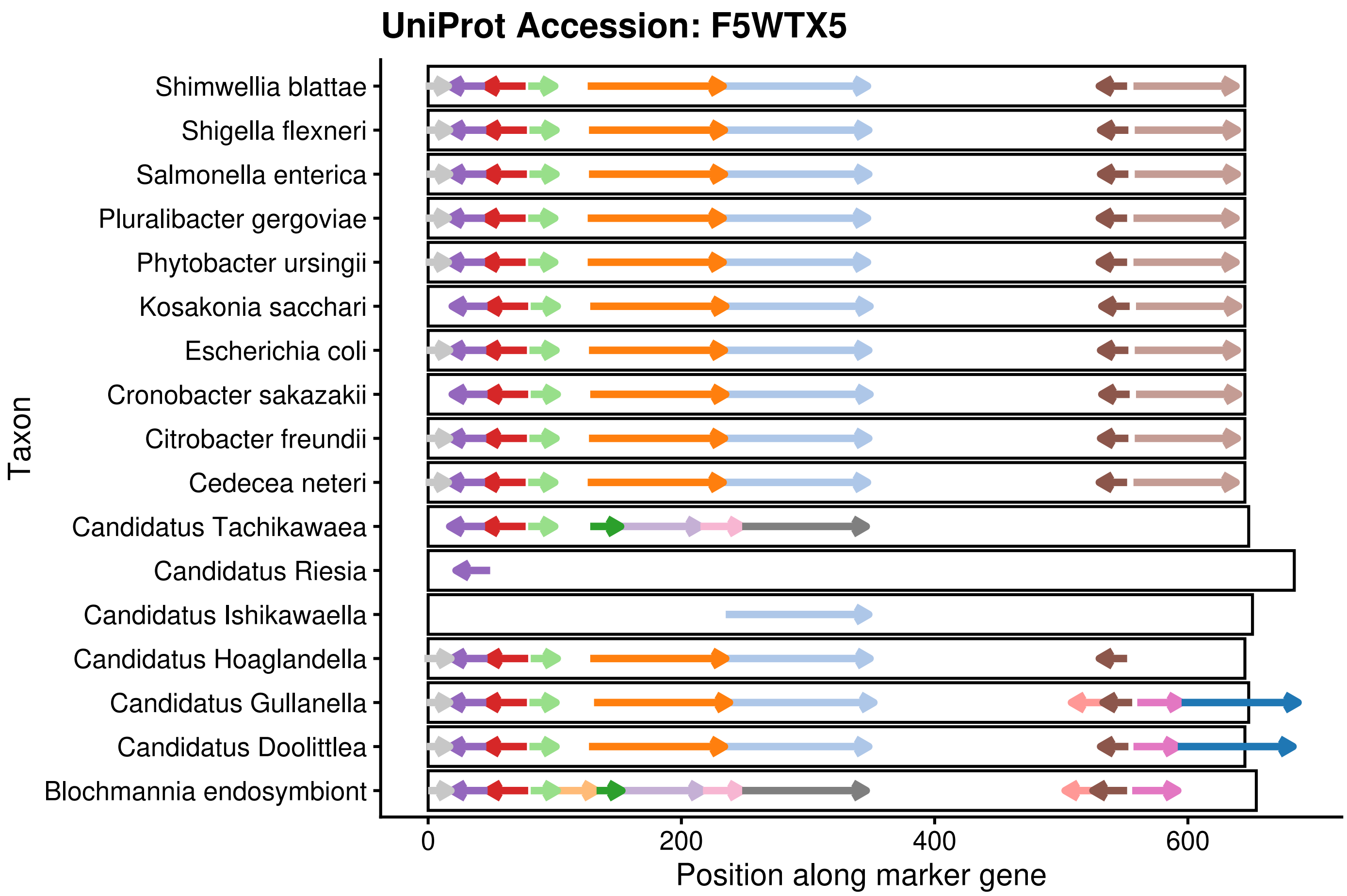

UniProt Accession: F5WTX5
Taxon
Shimwellia blattae
Shigella flexneri
Salmonella enterica
Pluralibacter gergoviae
Phytobacter ursingii
Kosakonia sacchari
Escherichia coli
Cronobacter sakazakii
Citrobacter freundii
Cedecea neteri
Candidatus Tachikawaea
Candidatus Riesia
Candidatus Ishikawaella
Candidatus Hoaglandella
Candidatus Gullanella
Candidatus Doolittlea
Blochmannia endosymbiont
0
200
400
600
Position along marker gene

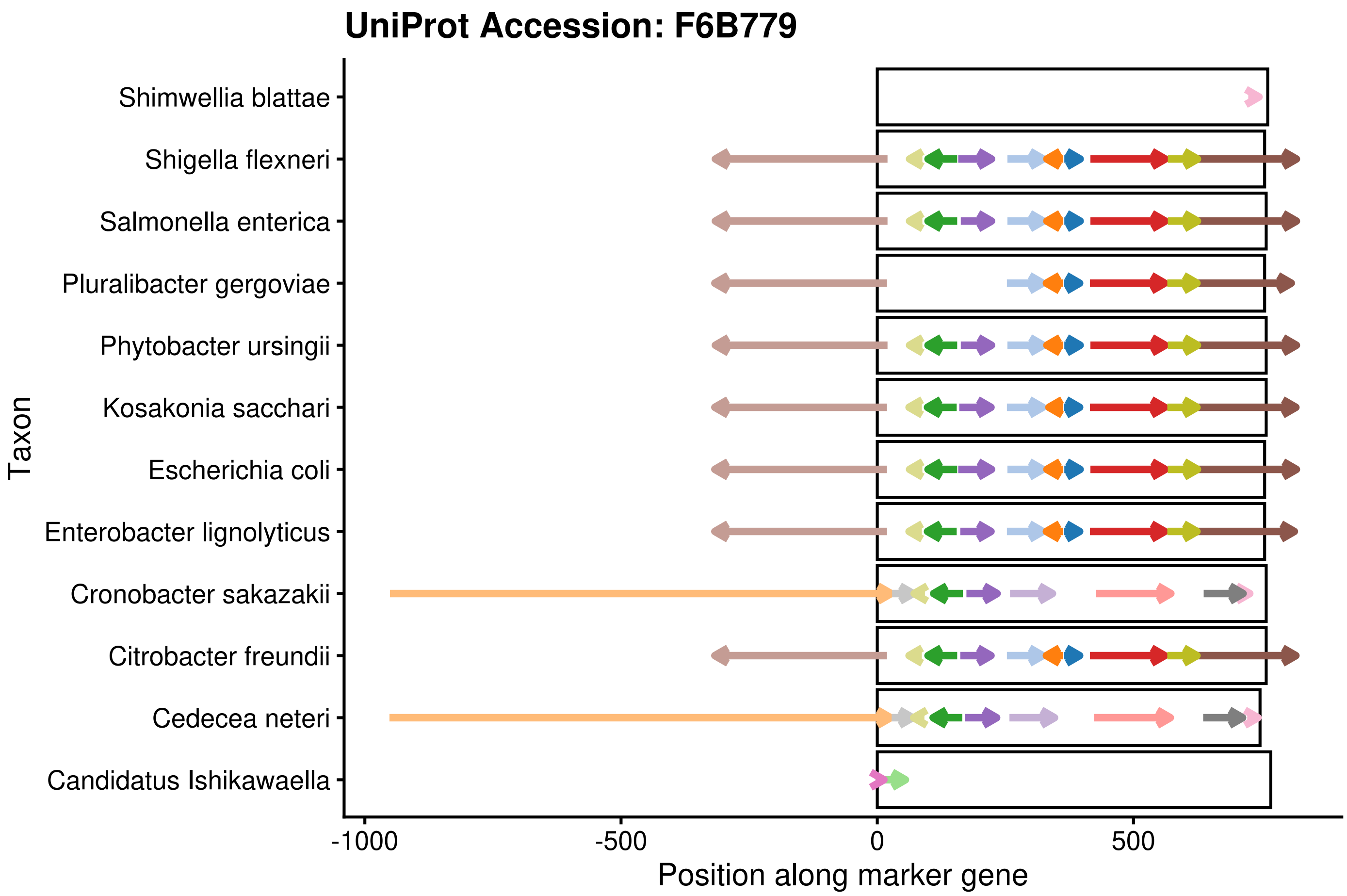
UniProt Accession: F6B779
Shimwellia blattae
Shigella flexneri
Salmonella enterica
Pluralibacter gergoviae
Phytobacter ursingii
Kosakonia sacchari
Escherichia coli
Enterobacter lignolyticus
Cronobacter sakazakii
Citrobacter freundii
Cedecea neteri
Candidatus Ishikawaella
Taxon
-1000
-500
0
500
Position along marker gene

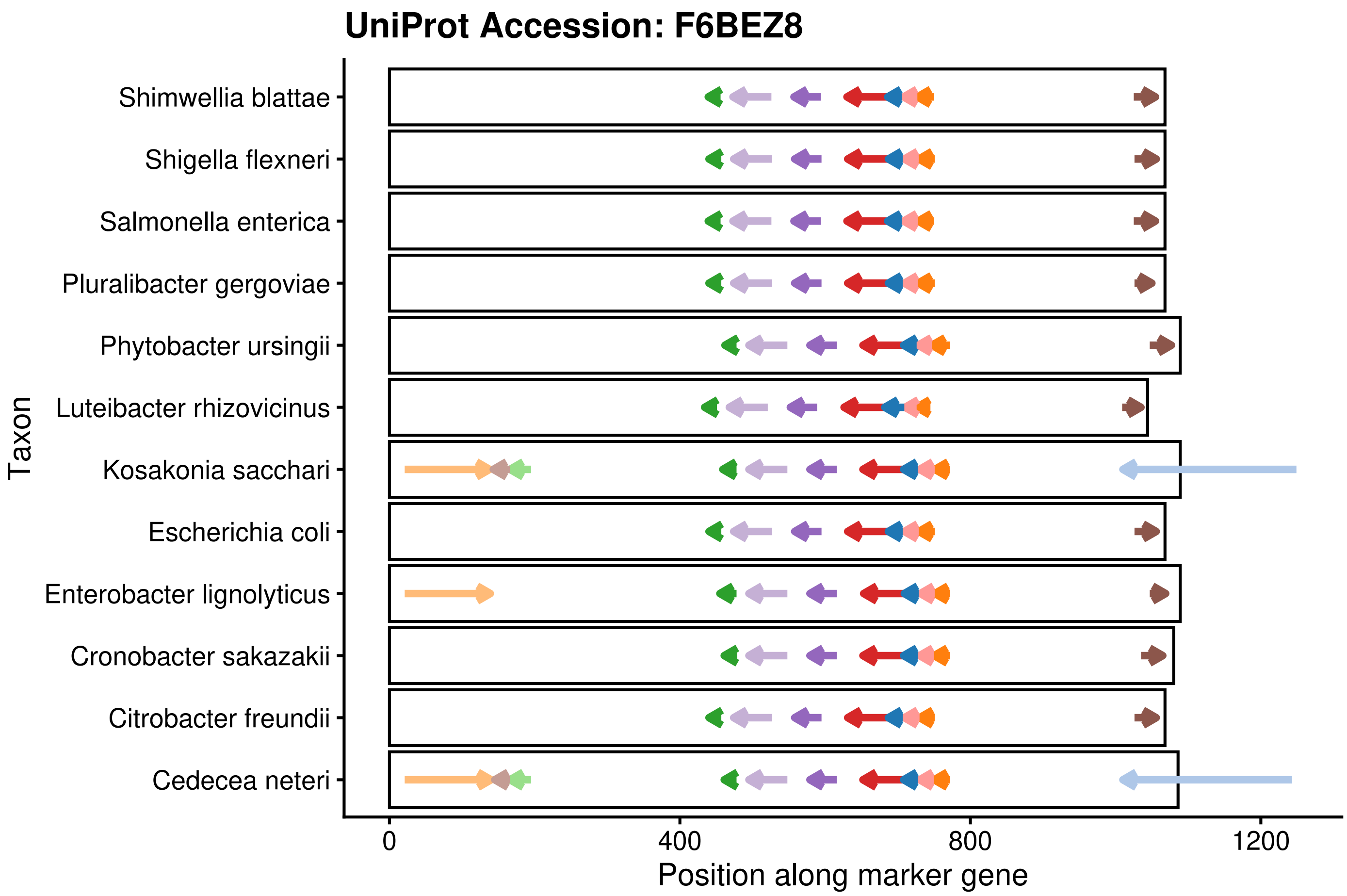

UniProt Accession: F6BEZ8
Taxon
Shimwellia blattae
Shigella flexneri
Salmonella enterica
Pluralibacter gergoviae
Phytobacter ursingii
Luteibacter rhizovicinus
Kosakonia sacchari
Escherichia coli
Enterobacter lignolyticus
Cronobacter sakazakii
Citrobacter freundii
Cedecea neteri
0
400
800
1200
Position along marker gene

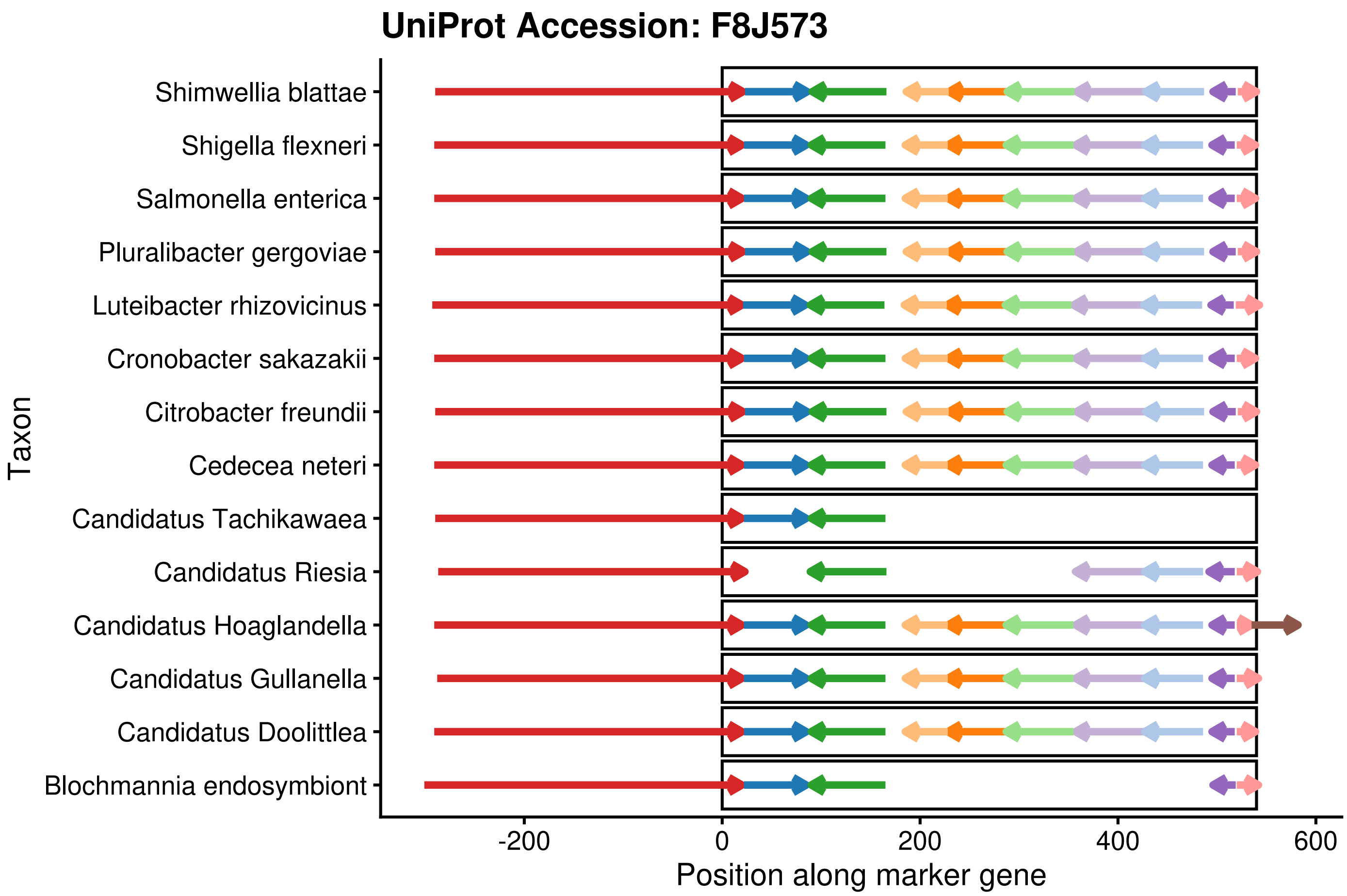
UniProt Accession: F8J573
Taxon
Shimwellia blattae
Shigella flexneri
Salmonella enterica
Pluralibacter gergoviae
Luteibacter rhizovicinus
Cronobacter sakazakii
Citrobacter freundii
Cedecea neteri
Candidatus Tachikawaea
Candidatus Riesia
Candidatus Hoaglandella
Candidatus Gullanella
Candidatus Doolittlea
Blochmannia endosymbiont
-200
0
200
400
600
Position along marker gene

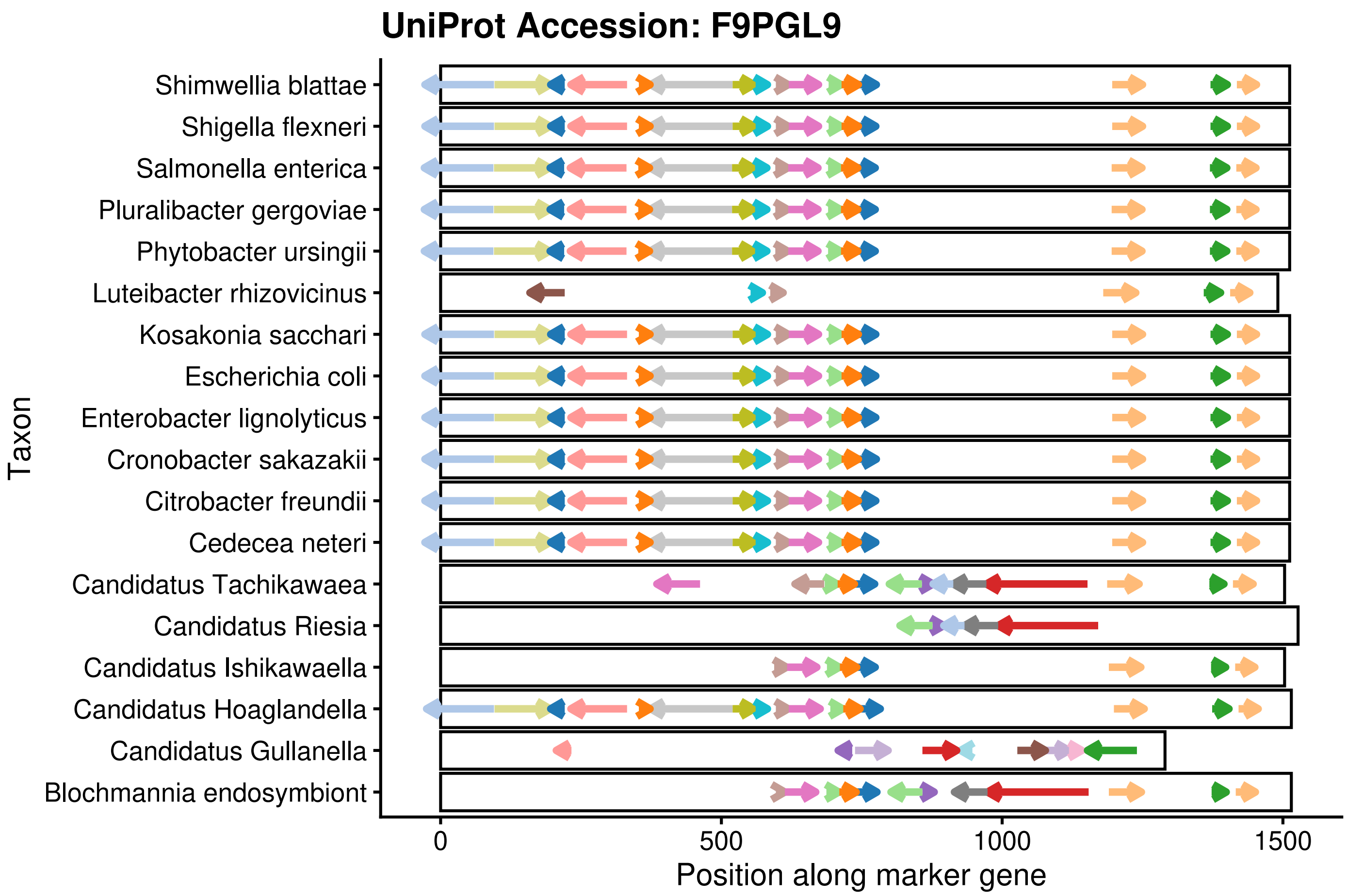
UniProt Accession: F9PGL9
Shimwellia blattae
Shigella flexneri
Salmonella enterica
Pluralibacter gergoviae
Phytobacter ursingii
Luteibacter rhizovicinus
Kosakonia sacchari
Escherichia coli
Enterobacter lignolyticus
Cronobacter sakazakii
Citrobacter freundii
Cedecea neteri
Candidatus Tachikawaea
Candidatus Riesia
Candidatus Ishikawaella
Candidatus Hoaglandella
Candidatus Gullanella
Blochmannia endosymbiont
Taxon
0
500
1000
1500
Position along marker gene

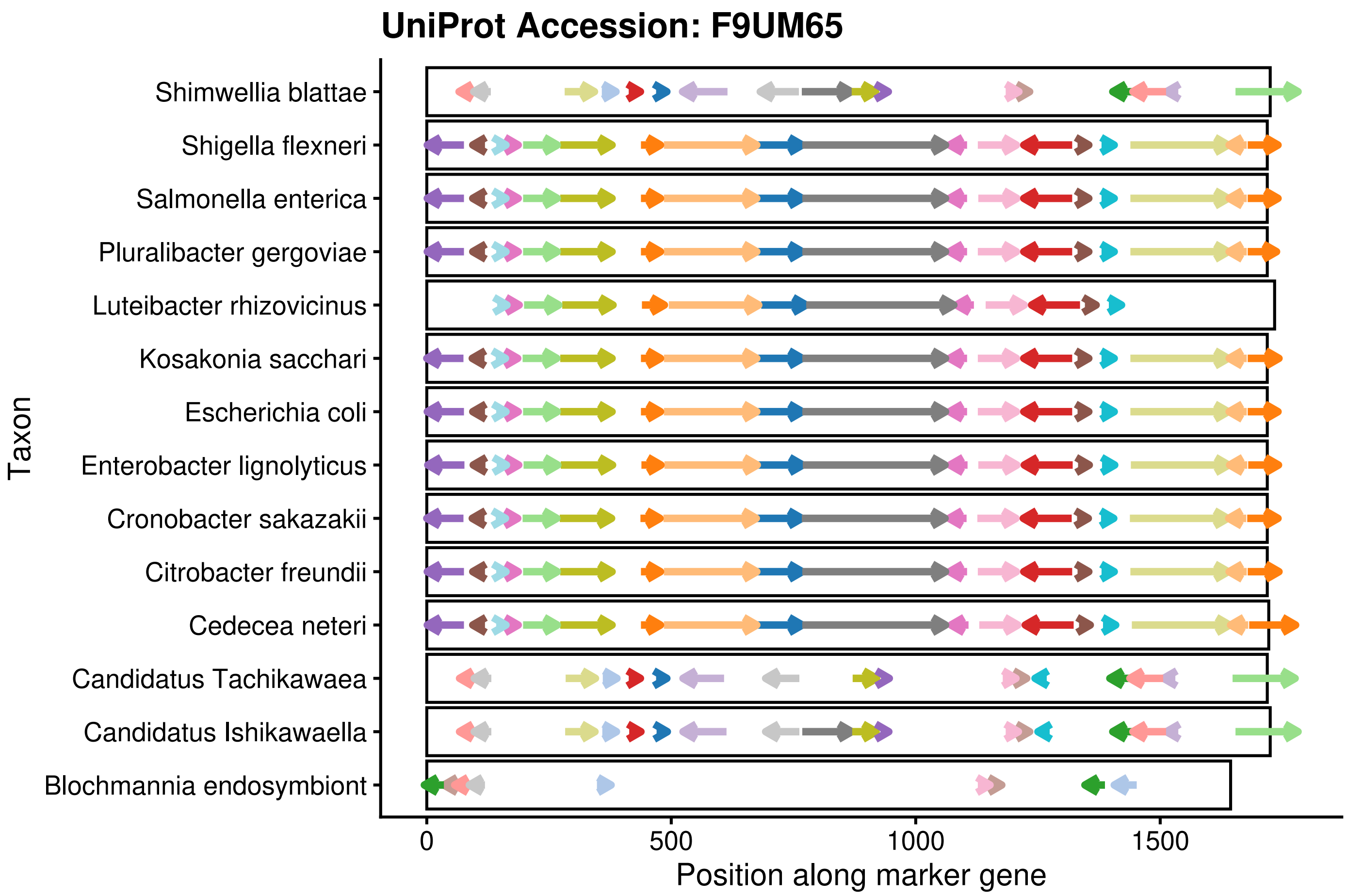
UniProt Accession: F9UM65
Shimwellia blattae
Shigella flexneri
Salmonella enterica
Pluralibacter gergoviae
Luteibacter rhizovicinus
Kosakonia sacchari
Escherichia coli
Enterobacter lignolyticus
Cronobacter sakazakii
Citrobacter freundii
Cedecea neteri
Candidatus Tachikawaea
Candidatus Ishikawaella
Blochmannia endosymbiont
Taxon
0
500
1000
1500
Position along marker gene

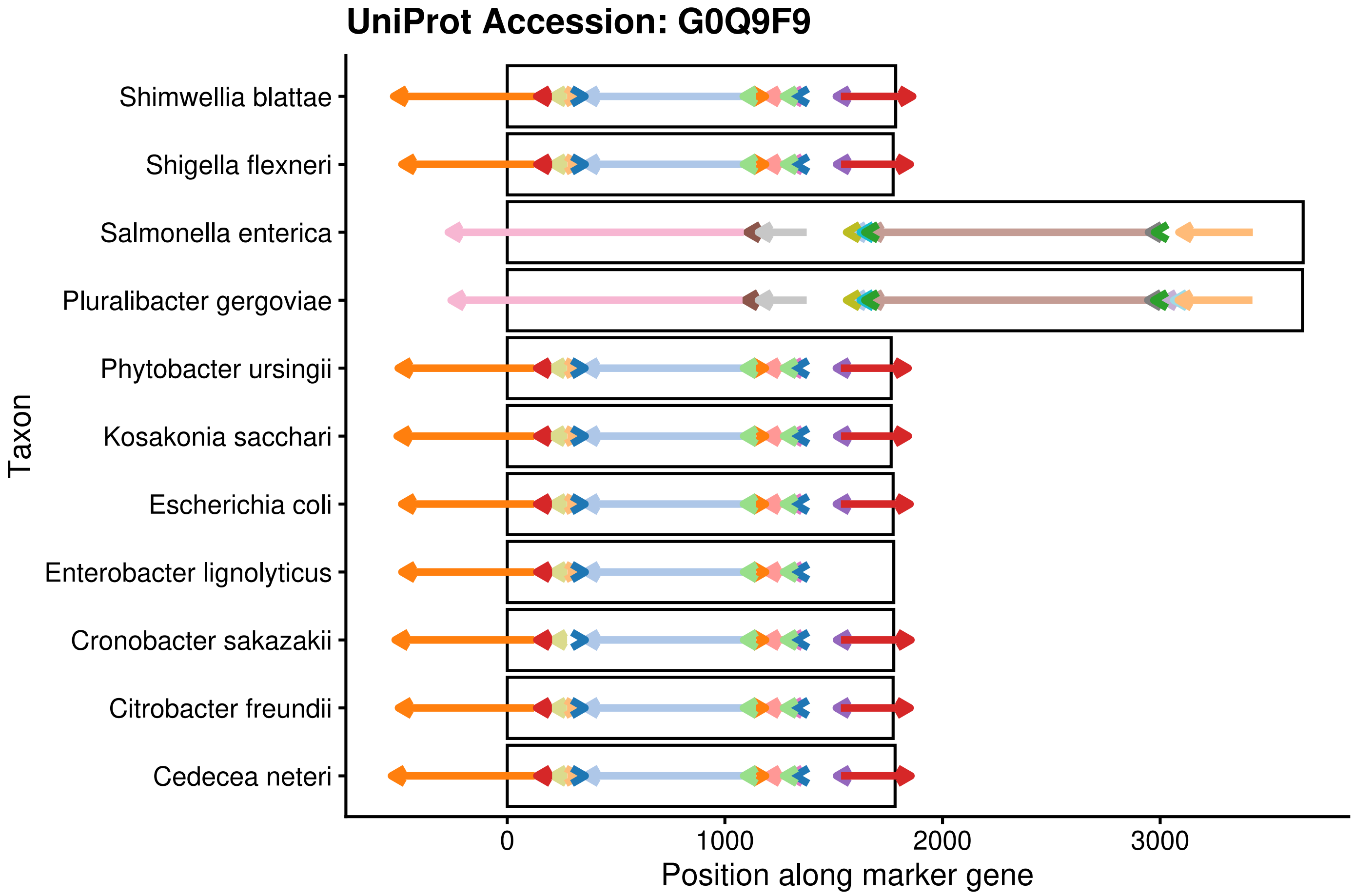

UniProt Accession: G0Q9F9
Shimwellia blattae
Shigella flexneri
Salmonella enterica
Pluralibacter gergoviae
Phytobacter ursingii
Kosakonia sacchari
Escherichia coli
Enterobacter lignolyticus
Cronobacter sakazakii
Citrobacter freundii
Cedecea neteri
Taxon
0
1000
2000
3000
Position along marker gene

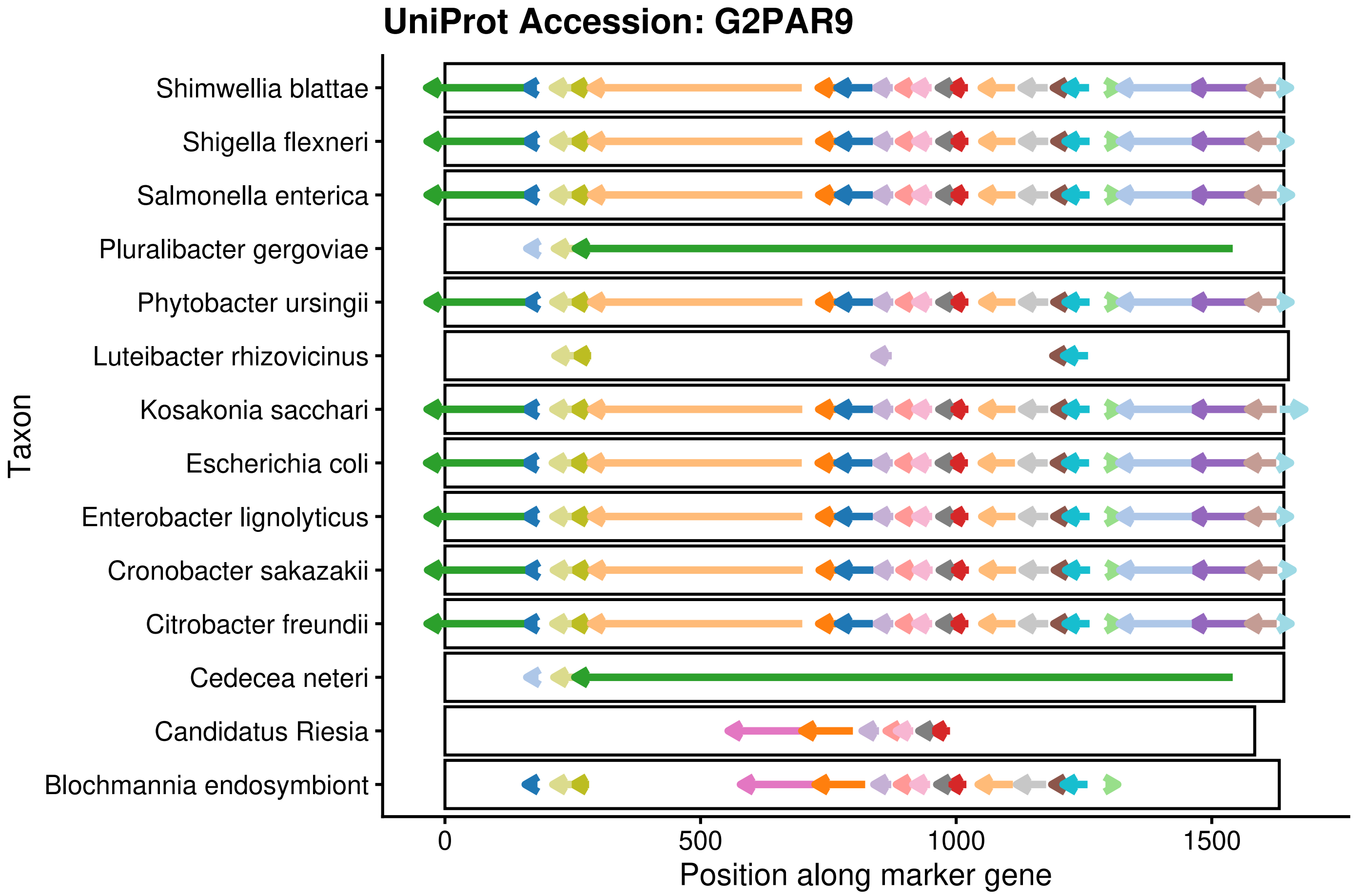

UniProt Accession: G2PAR9
Taxon
Shimwellia blattae
Shigella flexneri
Salmonella enterica
Pluralibacter gergoviae
Phytobacter ursingii
Luteibacter rhizovicinus
Kosakonia sacchari
Escherichia coli
Enterobacter lignolyticus
Cronobacter sakazakii
Citrobacter freundii
Cedecea neteri
Candidatus Riesia
Blochmannia endosymbiont
0
500
1000
1500
Position along marker gene

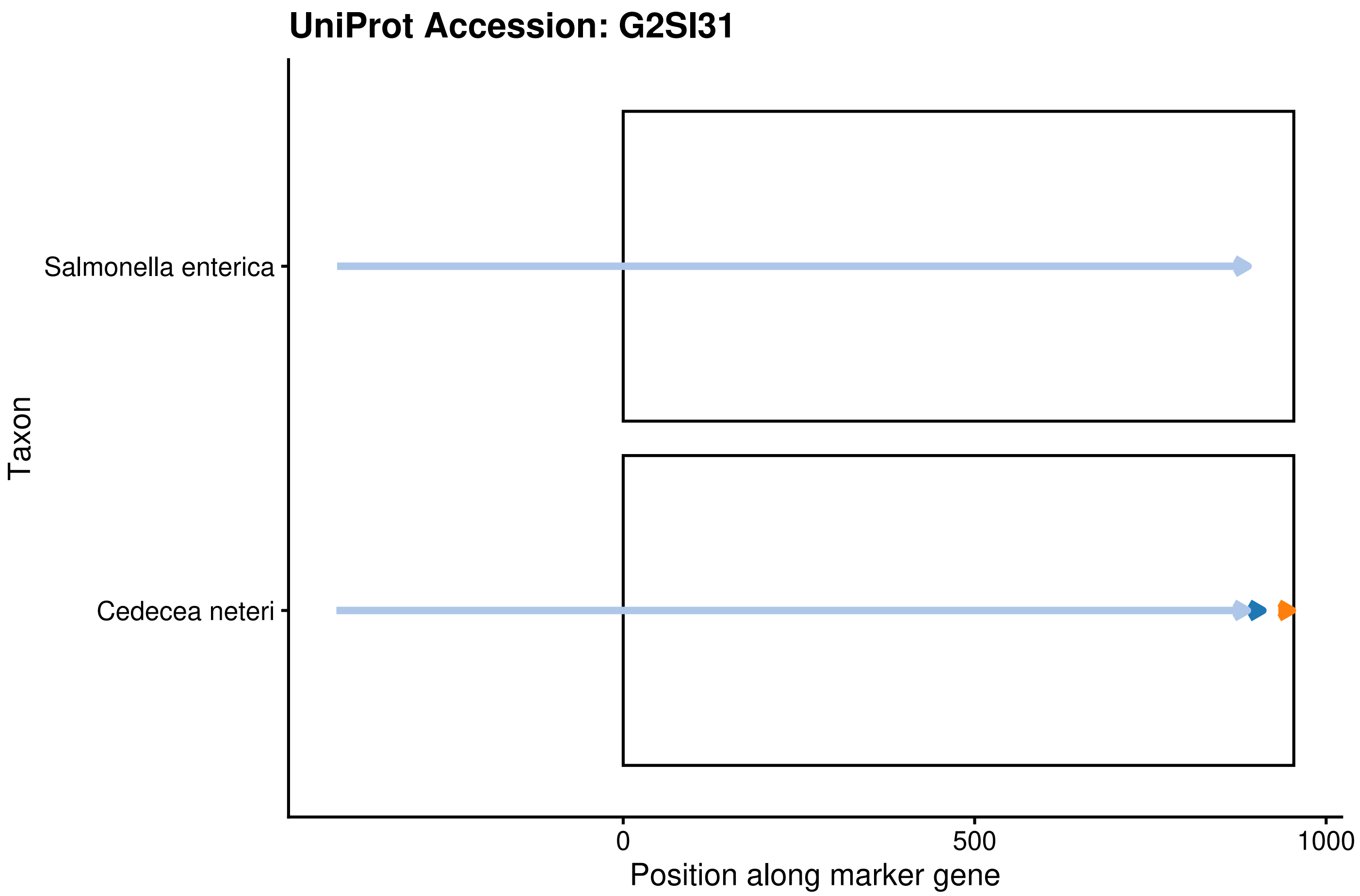

UniProt Accession: G2SI31
Salmonella enterica
Cedecea neteri
Taxon
0
500
1000
Position along marker gene

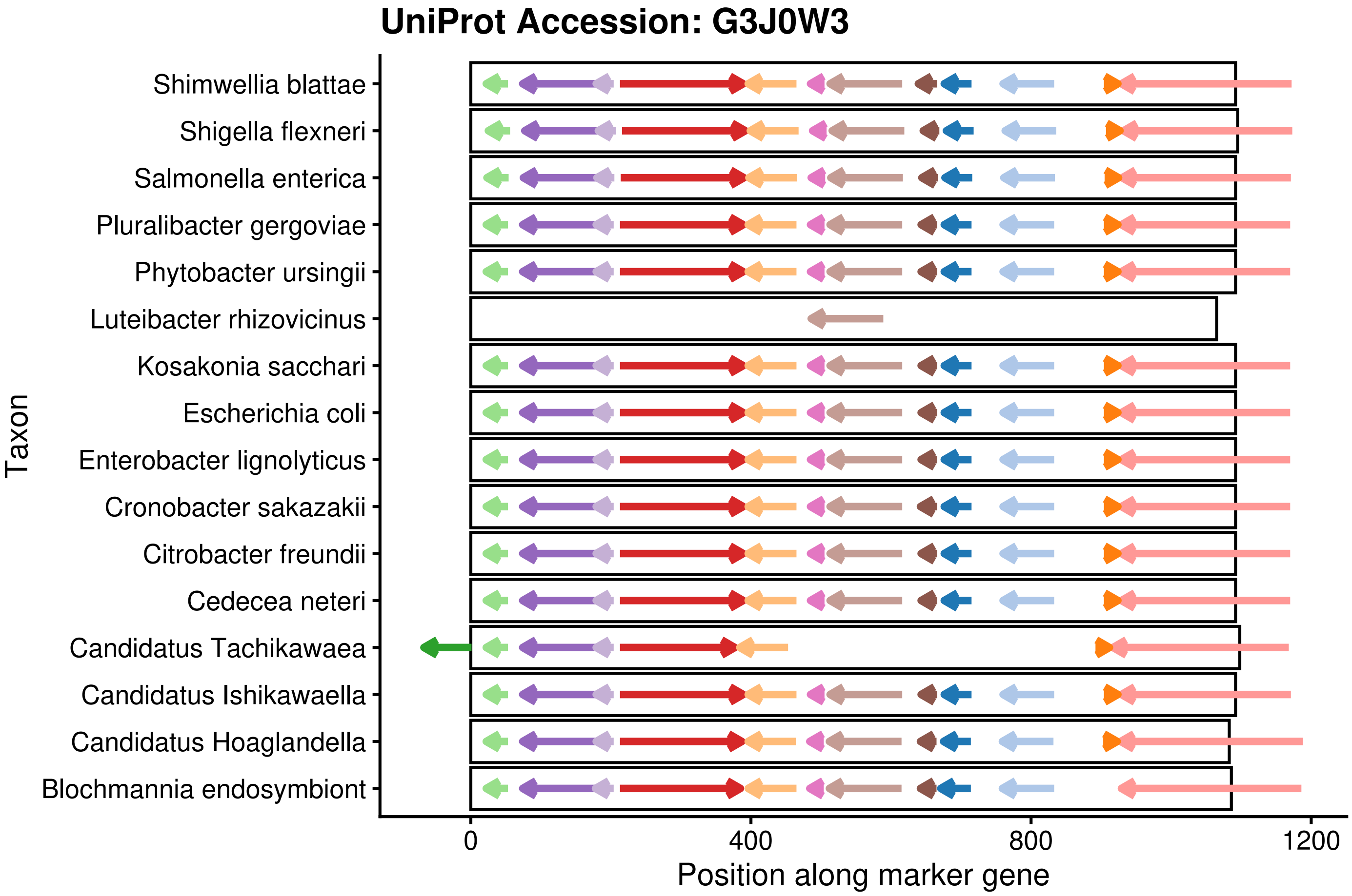
UniProt Accession: G3J0W3
Taxon
Shimwellia blattae
Shigella flexneri
Salmonella enterica
Pluralibacter gergoviae
Phytobacter ursingii
Luteibacter rhizovicinus
Kosakonia sacchari
Escherichia coli
Enterobacter lignolyticus
Cronobacter sakazakii
Citrobacter freundii
Cedecea neteri
Candidatus Tachikawaea
Candidatus Ishikawaella
Candidatus Hoaglandella
Blochmannia endosymbiont
0
400
800
1200
Position along marker gene

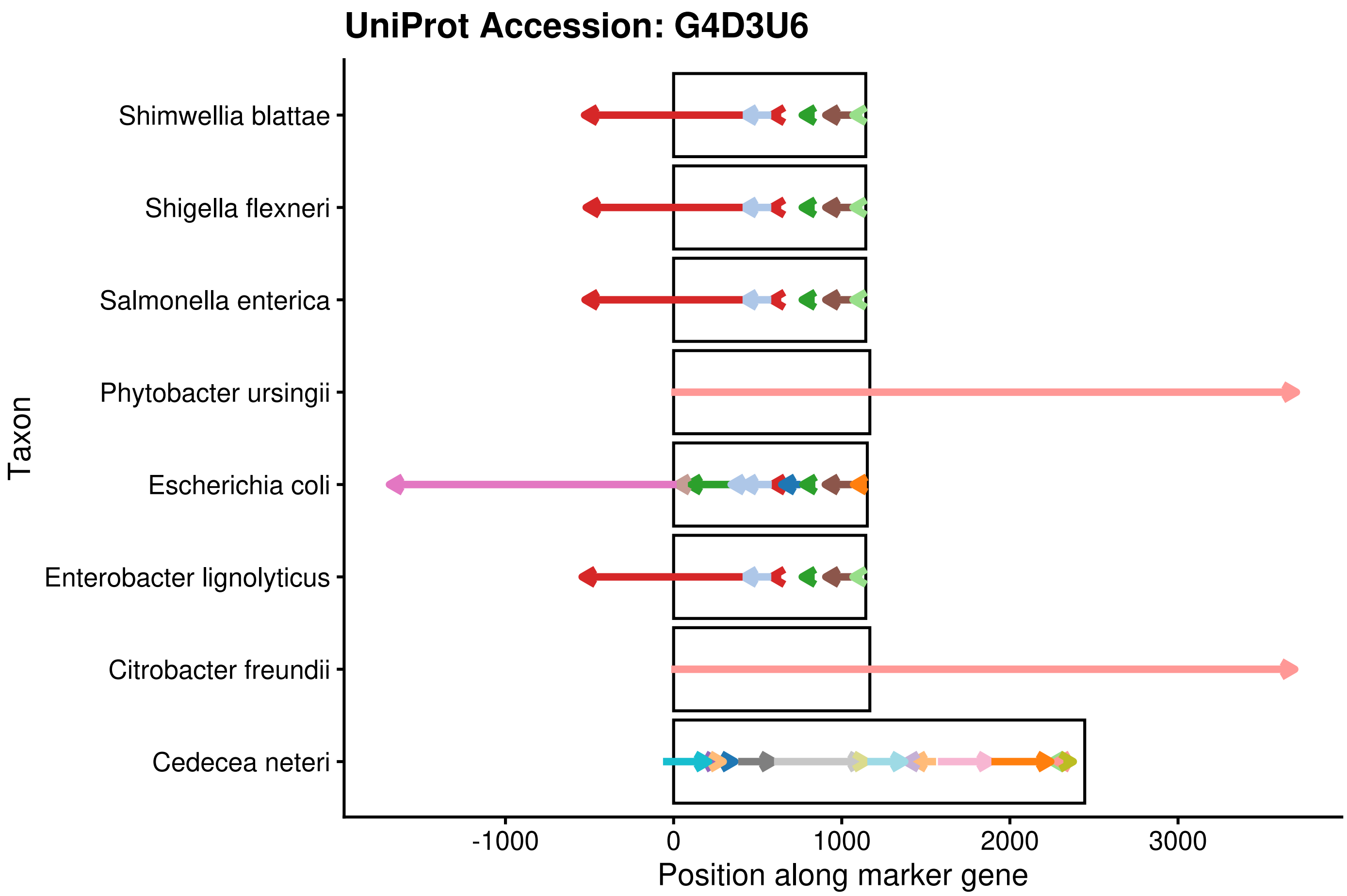
UniProt Accession: G4D3U6
Taxon
Shimwellia blattae
Shigella flexneri
Salmonella enterica
Phytobacter ursingii
Escherichia coli
Enterobacter lignolyticus
Citrobacter freundii
Cedecea neteri
-1000
0
1000
2000
3000
Position along marker gene

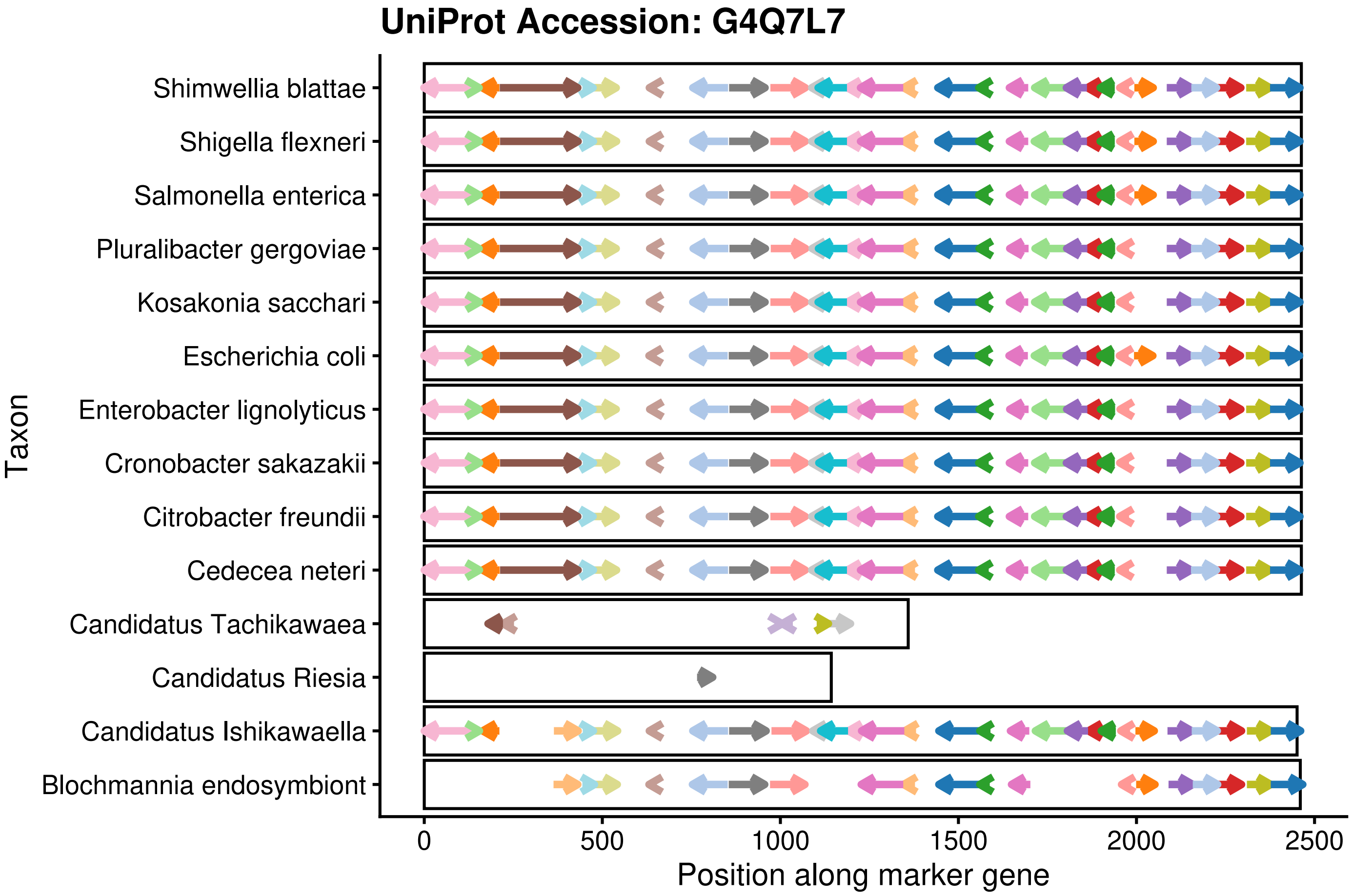
UniProt Accession: G4Q7L7
Taxon
Shimwellia blattae
Shigella flexneri
Salmonella enterica
Pluralibacter gergoviae
Kosakonia sacchari
Escherichia coli
Enterobacter lignolyticus
Cronobacter sakazakii
Citrobacter freundii
Cedecea neteri
Candidatus Tachikawaea
Candidatus Riesia
Candidatus Ishikawaella
Blochmannia endosymbiont
0
500
1000
1500
2000
2500
Position along marker gene

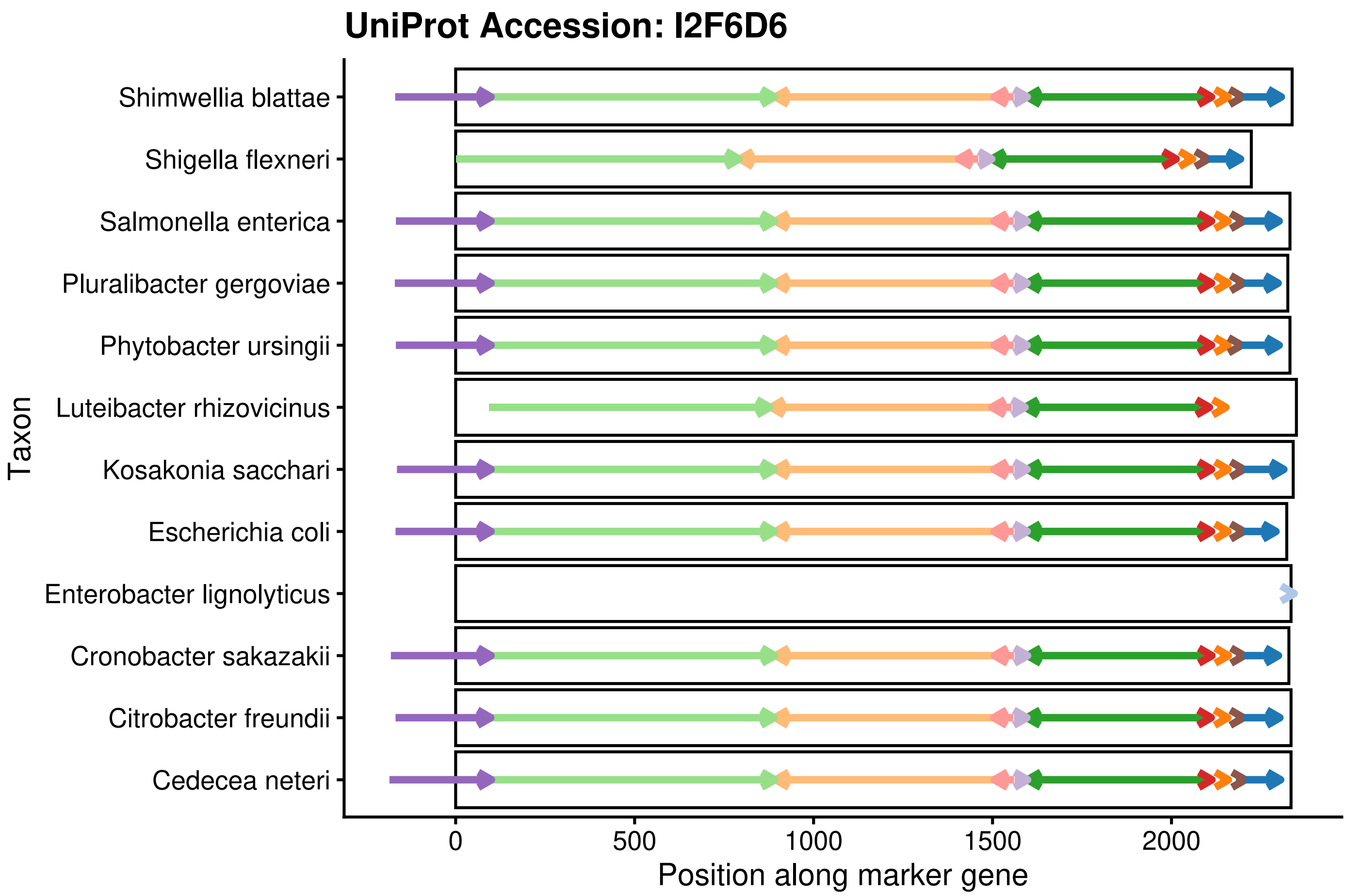
UniProt Accession: I2F6D6
Taxon
Shimwellia blattae
Shigella flexneri
Salmonella enterica
Pluralibacter gergoviae
Phytobacter ursingii
Luteibacter rhizovicinus
Kosakonia sacchari
Escherichia coli
Enterobacter lignolyticus
Cronobacter sakazakii
Citrobacter freundii
Cedecea neteri
0
500
1000
1500
2000
Position along marker gene

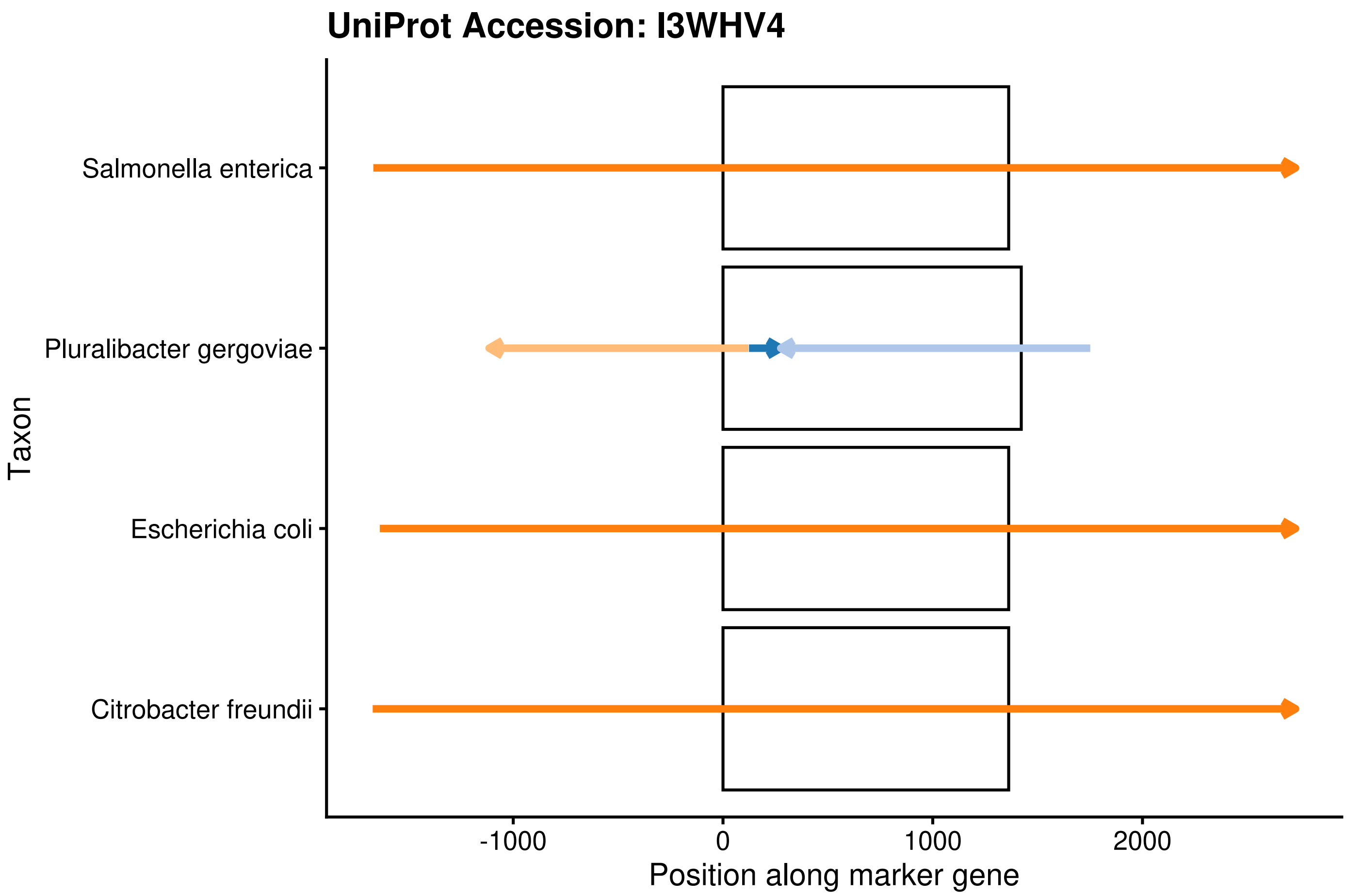
UniProt Accession: I3WHV4
Salmonella enterica
Pluralibacter gergoviae
Escherichia coli
Citrobacter freundii
Taxon
-1000
0
1000
2000
Position along marker gene

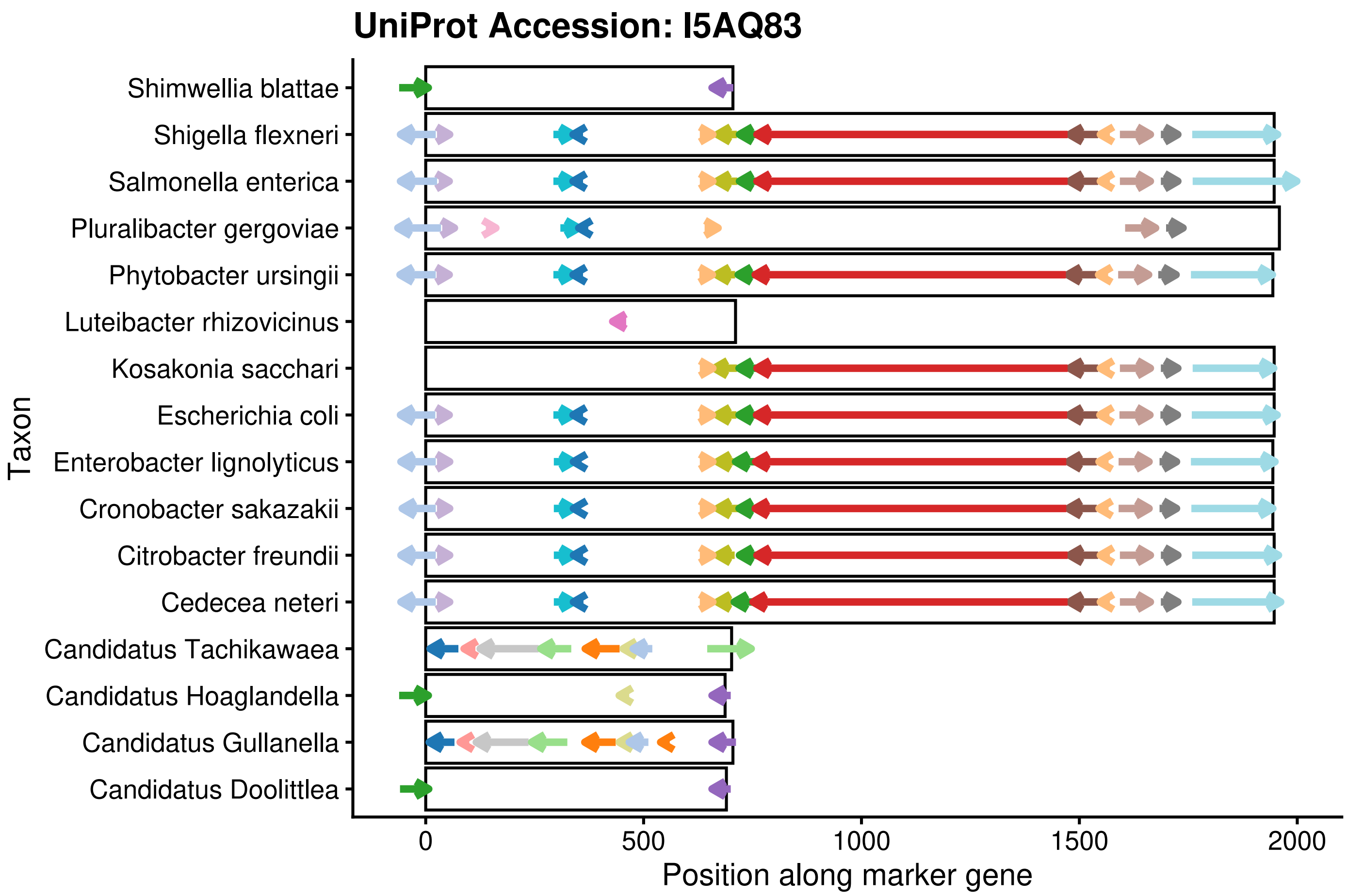
UniProt Accession: I5AQ83
Taxon
Shimwellia blattae
Shigella flexneri
Salmonella enterica
Pluralibacter gergoviae
Phytobacter ursingii
Luteibacter rhizovicinus
Kosakonia sacchari
Escherichia coli
Enterobacter lignolyticus
Cronobacter sakazakii
Citrobacter freundii
Cedecea neteri
Candidatus Tachikawaea
Candidatus Hoaglandella
Candidatus Gullanella
Candidatus Doolittlea
0
500
1000
1500
2000
Position along marker gene

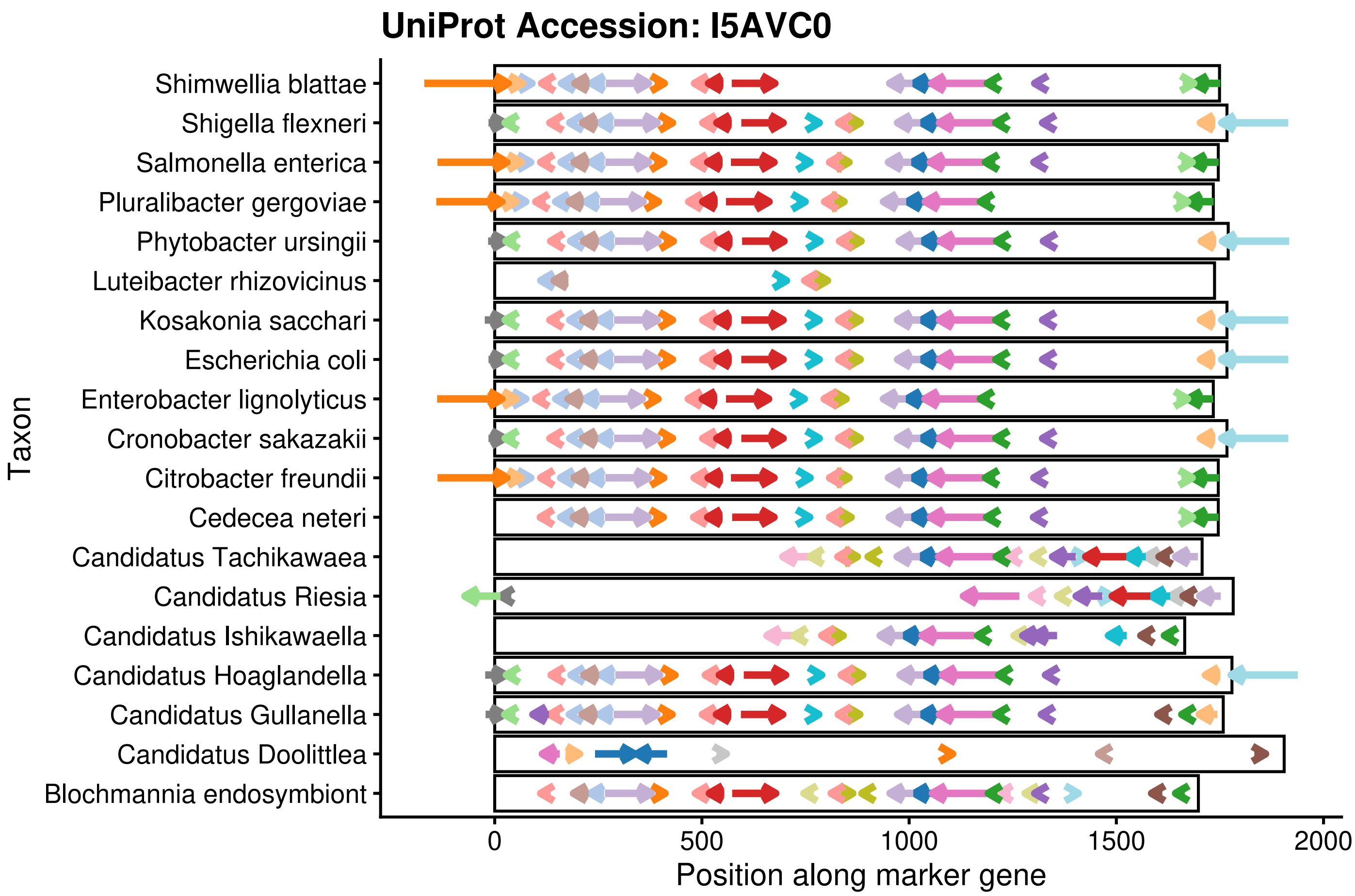

UniProt Accession: I5AVC0
Taxon
Shimwellia blattae
Shigella flexneri
Salmonella enterica
Pluralibacter gergoviae
Phytobacter ursingii
Luteibacter rhizovicinus
Kosakonia sacchari
Escherichia coli
Enterobacter lignolyticus
Cronobacter sakazakii
Citrobacter freundii
Cedecea neteri
Candidatus Tachikawaea
Candidatus Riesia
Candidatus Ishikawaella
Candidatus Hoaglandella
Candidatus Gullanella
Candidatus Doolittlea
Blochmannia endosymbiont
0
500
1000
1500
2000
Position along marker gene

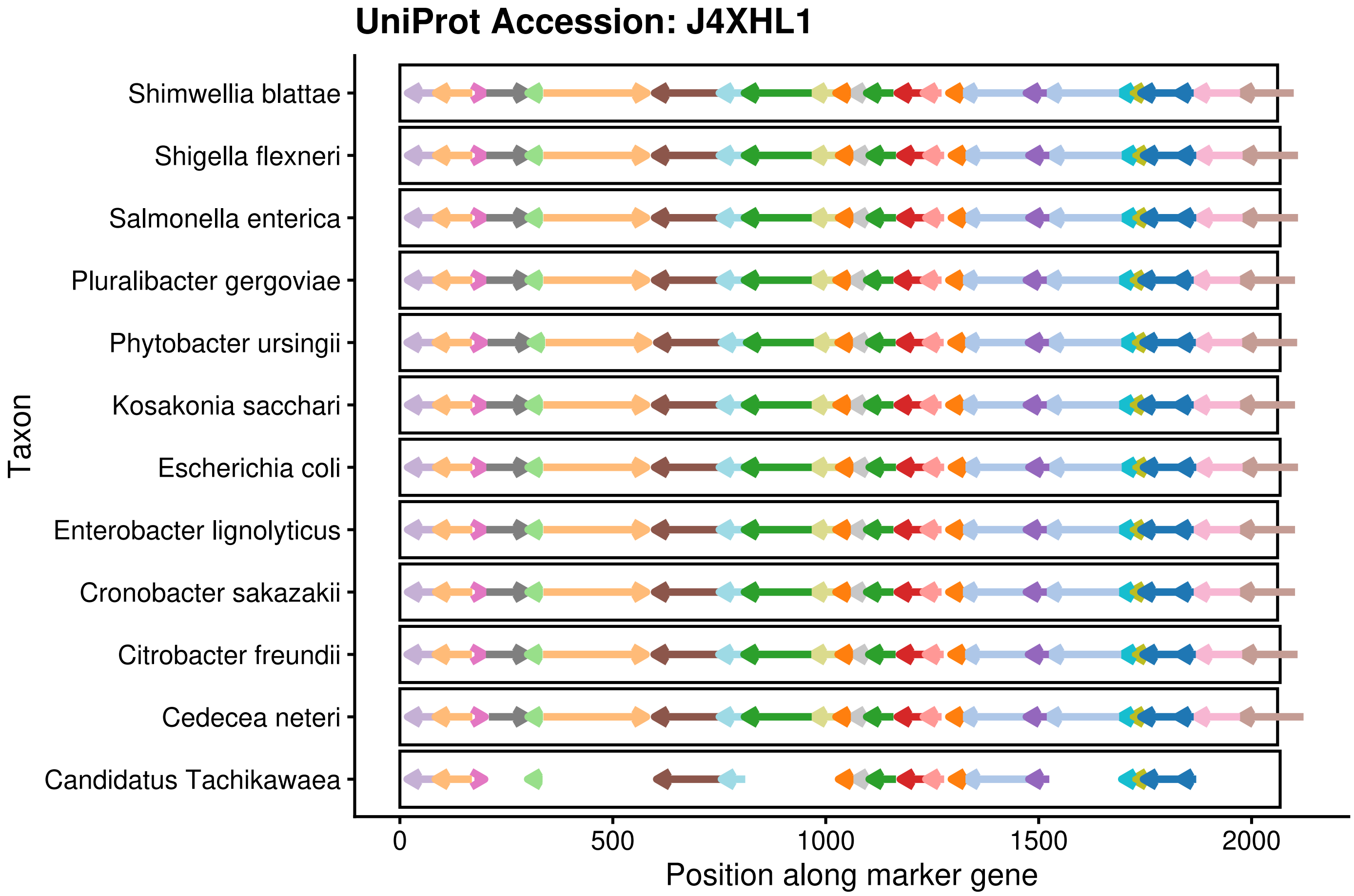

UniProt Accession: J4XHL1
Shimwellia blattae
Shigella flexneri
Salmonella enterica
Pluralibacter gergoviae
Phytobacter ursingii
Kosakonia sacchari
Escherichia coli
Enterobacter lignolyticus
Cronobacter sakazakii
Citrobacter freundii
Cedecea neteri
Candidatus Tachikawaea
Taxon
0
500
1000
1500
2000
Position along marker gene

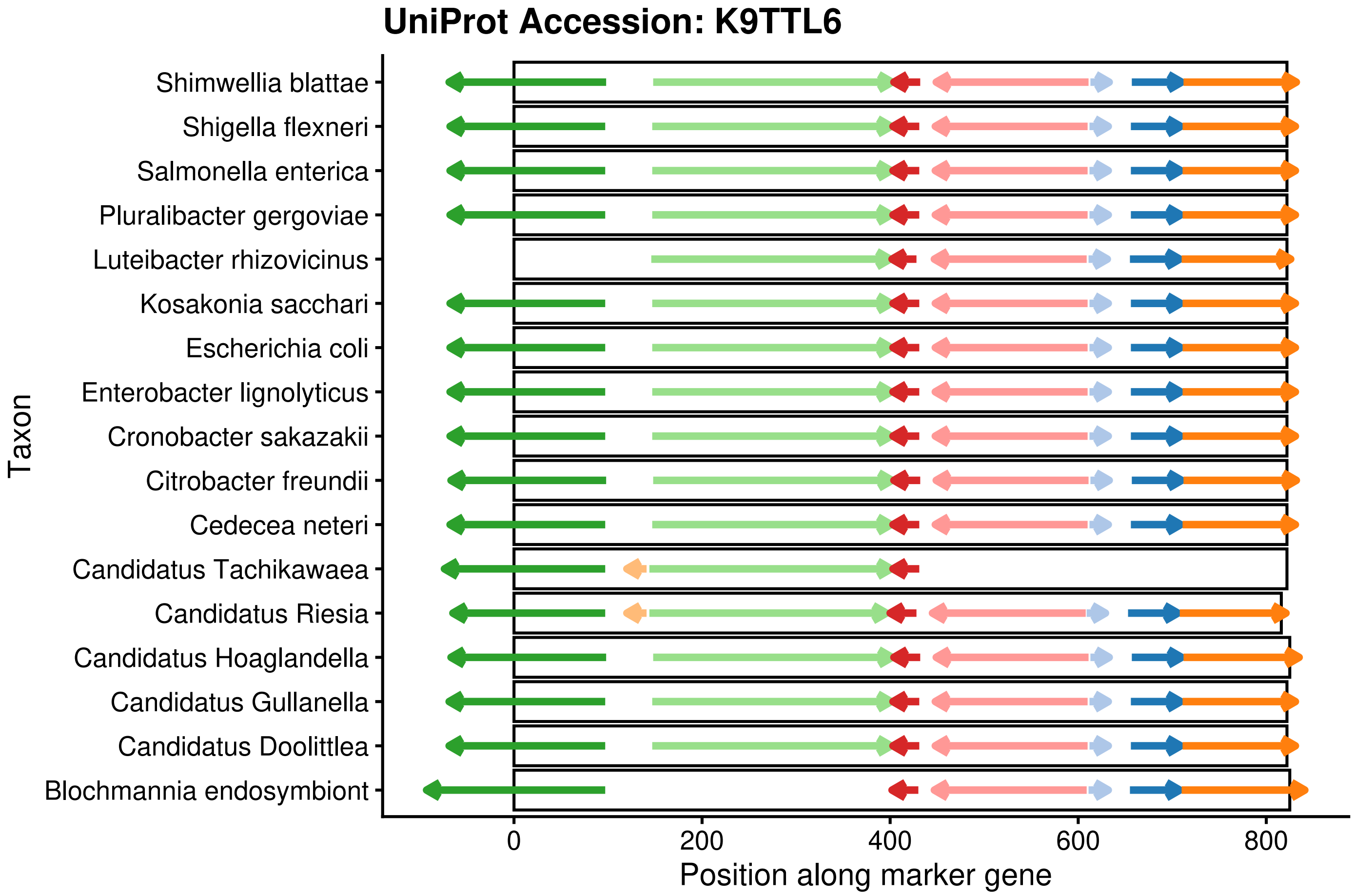
UniProt Accession: K9TTL6
Shimwellia blattae
Shigella flexneri
Salmonella enterica
Pluralibacter gergoviae
Luteibacter rhizovicinus
Kosakonia sacchari
Escherichia coli
Enterobacter lignolyticus
Cronobacter sakazakii
Citrobacter freundii
Cedecea neteri
Candidatus Tachikawaea
Candidatus Riesia
Candidatus Hoaglandella
Candidatus Gullanella
Candidatus Doolittlea
Blochmannia endosymbiont
Taxon
0
200
400
600
800
Position along marker gene

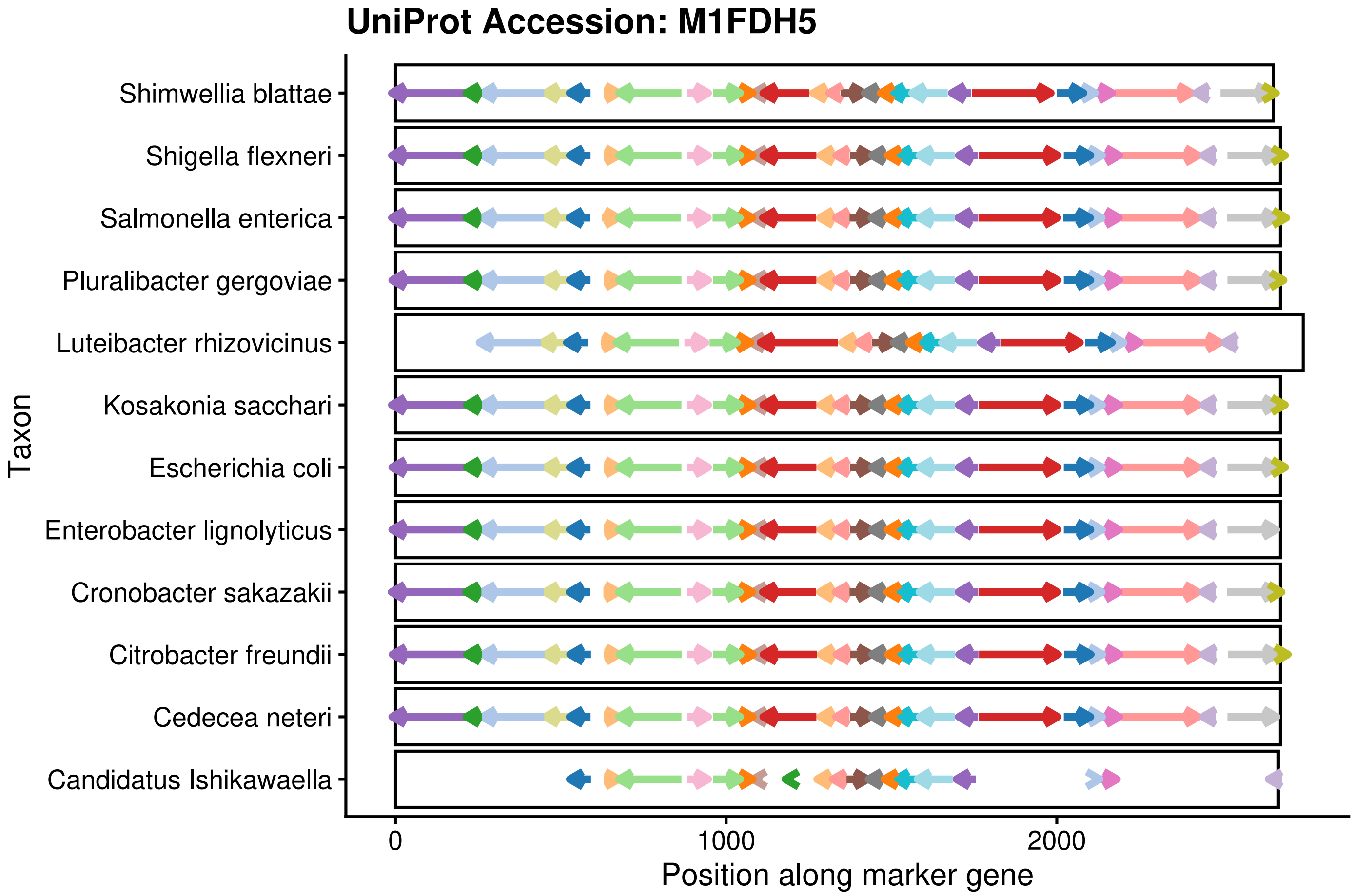
UniProt Accession: M1FDH5
Shimwellia blattae
Shigella flexneri
Salmonella enterica
Pluralibacter gergoviae
Luteibacter rhizovicinus
Kosakonia sacchari
Escherichia coli
Enterobacter lignolyticus
Cronobacter sakazakii
Citrobacter freundii
Cedecea neteri
Candidatus Ishikawaella
Taxon
0
1000
2000
Position along marker gene

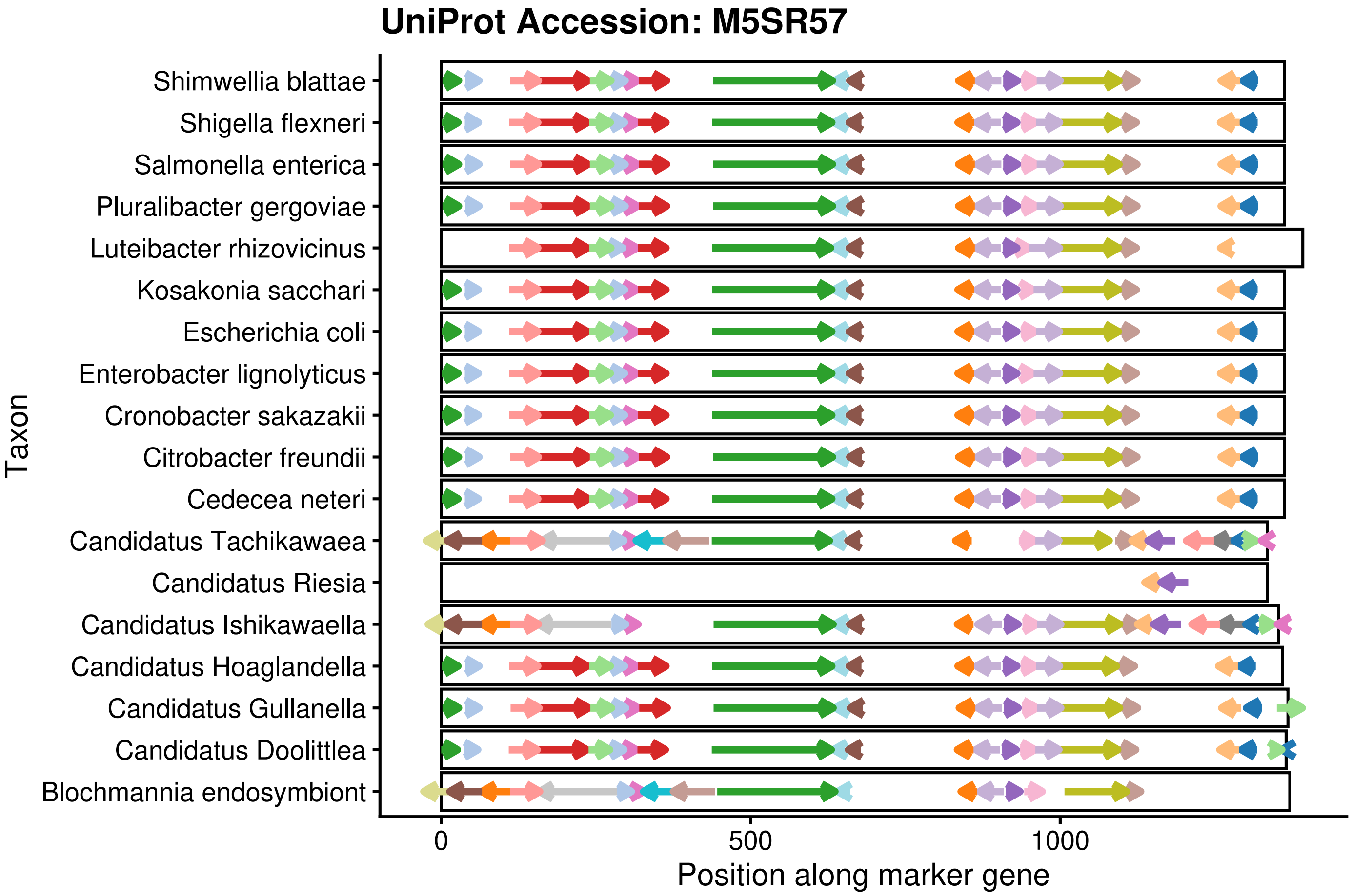

UniProt Accession: M5SR57
Taxon
Shimwellia blattae
Shigella flexneri
Salmonella enterica
Pluralibacter gergoviae
Luteibacter rhizovicinus
Kosakonia sacchari
Escherichia coli
Enterobacter lignolyticus
Cronobacter sakazakii
Citrobacter freundii
Cedecea neteri
Candidatus Tachikawaea
Candidatus Riesia
Candidatus Ishikawaella
Candidatus Hoaglandella
Candidatus Gullanella
Candidatus Doolittlea
Blochmannia endosymbiont
0
500
1000
Position along marker gene

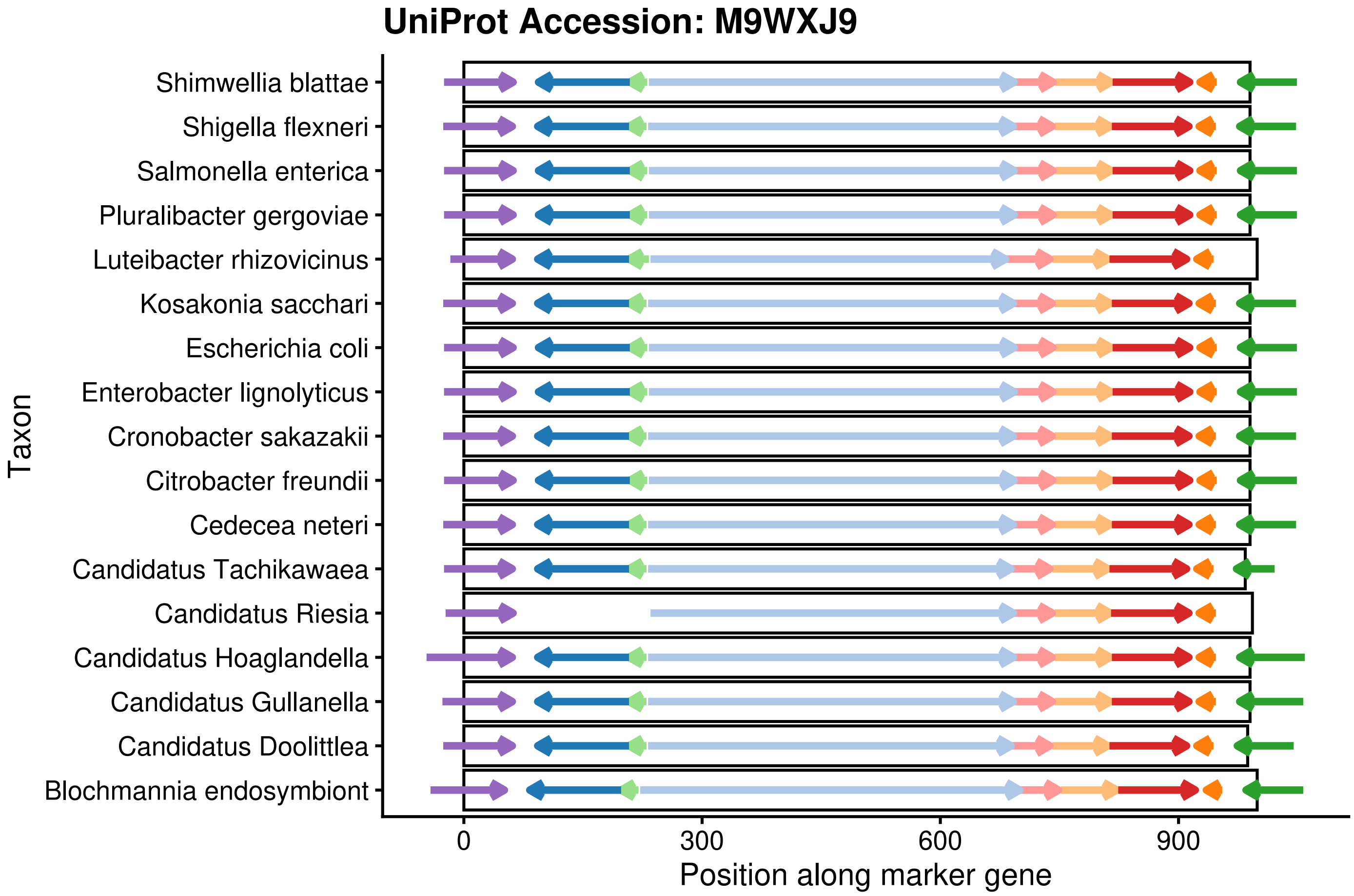
UniProt Accession: M9WXJ9
Shimwellia blattae
Shigella flexneri
Salmonella enterica
Pluralibacter gergoviae
Luteibacter rhizovicinus
Kosakonia sacchari
Escherichia coli
Enterobacter lignolyticus
Cronobacter sakazakii
Citrobacter freundii
Cedecea neteri
Candidatus Tachikawaea
Candidatus Riesia
Candidatus Hoaglandella
Candidatus Gullanella
Candidatus Doolittlea
Blochmannia endosymbiont
Taxon
0
300
600
900
Position along marker gene

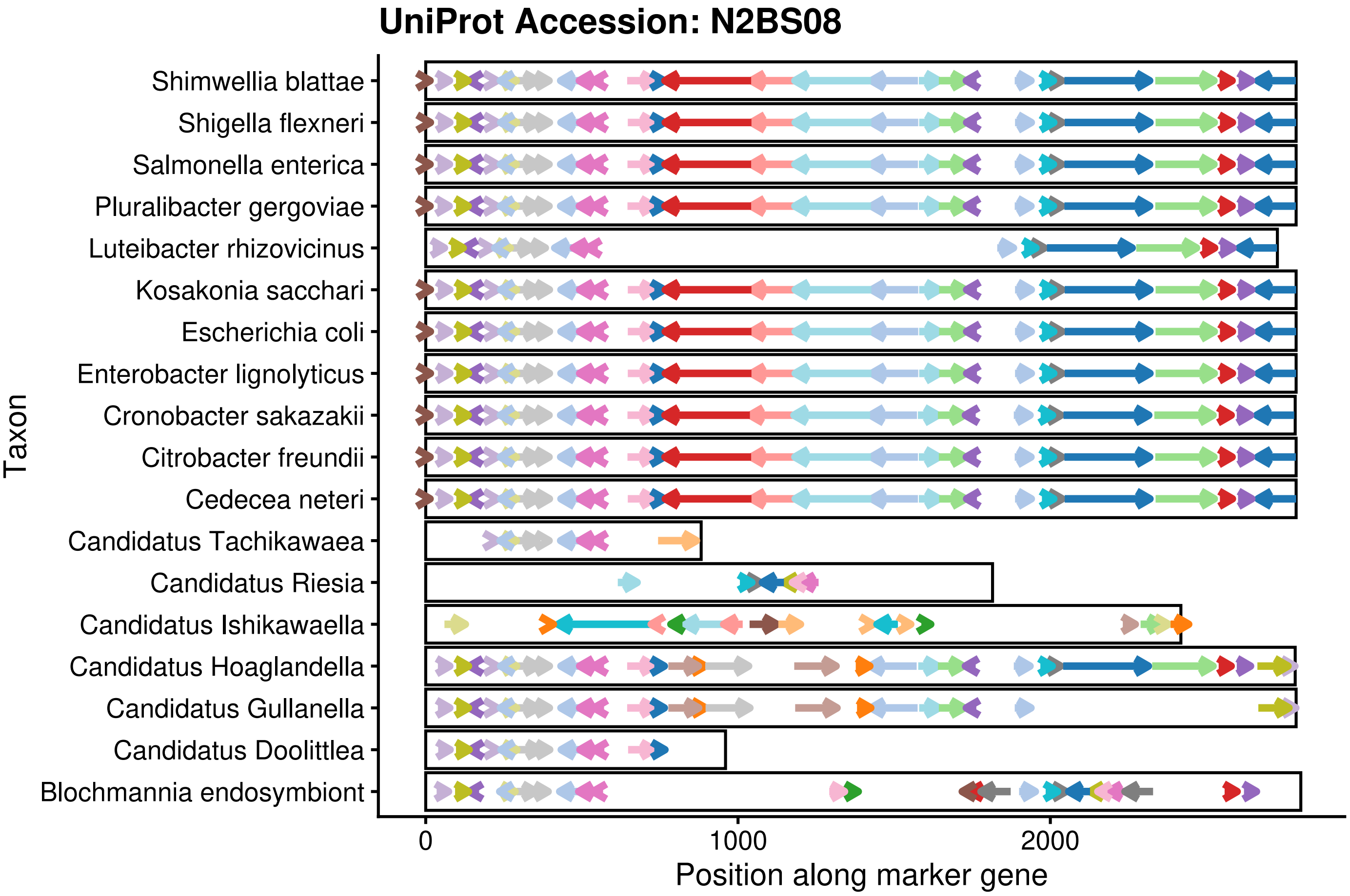
UniProt Accession: N2BS08
Taxon
Shimwellia blattae
Shigella flexneri
Salmonella enterica
Pluralibacter gergoviae
Luteibacter rhizovicinus
Kosakonia sacchari
Escherichia coli
Enterobacter lignolyticus
Cronobacter sakazakii
Citrobacter freundii
Cedecea neteri
Candidatus Tachikawaea
Candidatus Riesia
Candidatus Ishikawaella
Candidatus Hoaglandella
Candidatus Gullanella
Candidatus Doolittlea
Blochmannia endosymbiont
0
1000
2000
Position along marker gene

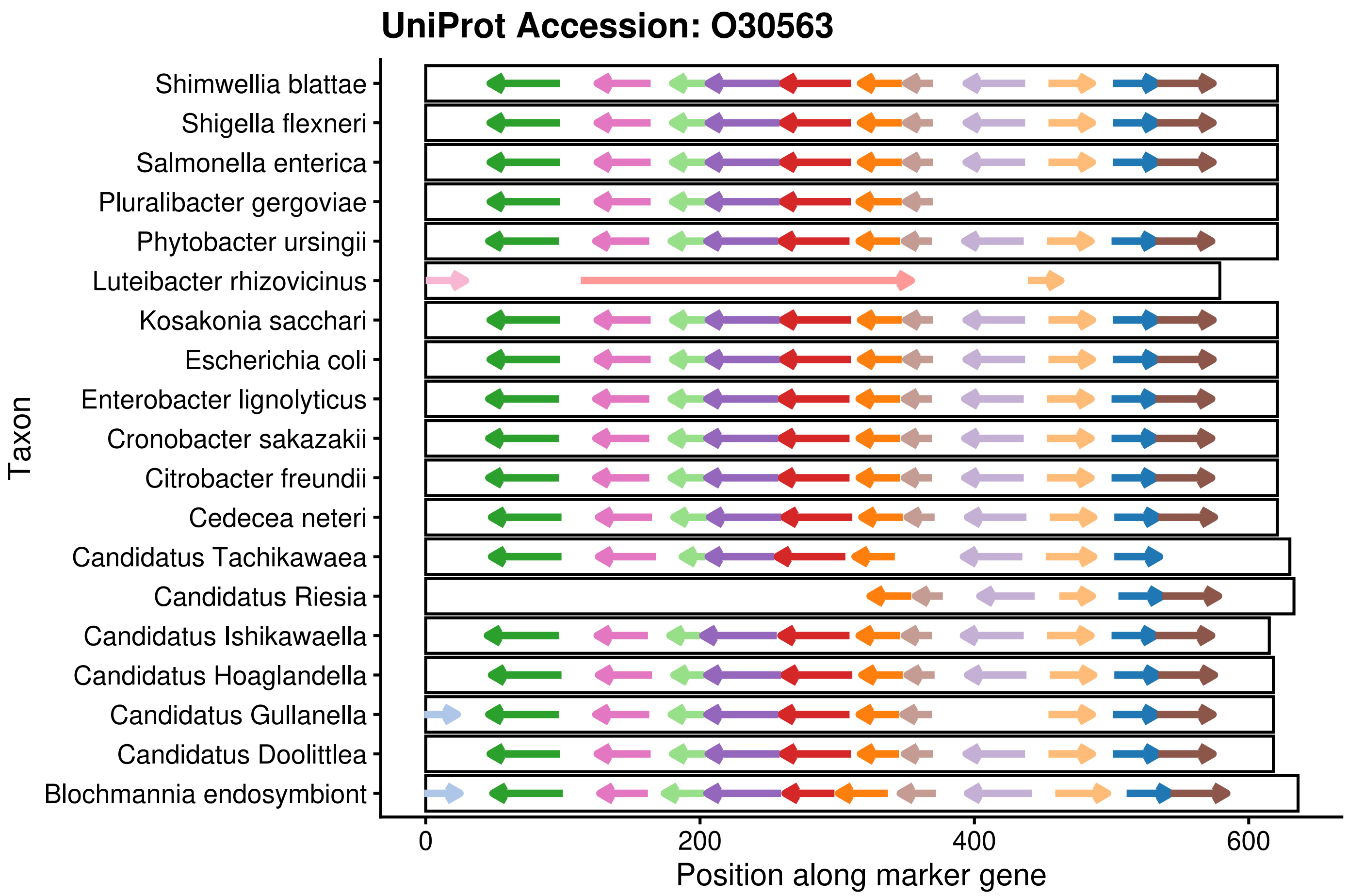

UniProt Accession: O30563
Shimwellia blattae
Shigella flexneri
Salmonella enterica
Pluralibacter gergoviae
Phytobacter ursingii
Luteibacter rhizovicinus
Kosakonia sacchari
Escherichia coli
Enterobacter lignolyticus
Cronobacter sakazakii
Citrobacter freundii
Cedecea neteri
Candidatus Tachikawaea
Candidatus Riesia
Candidatus Ishikawaella
Candidatus Hoaglandella
Candidatus Gullanella
Candidatus Doolittlea
Blochmannia endosymbiont
Taxon
0
200
400
600
Position along marker gene

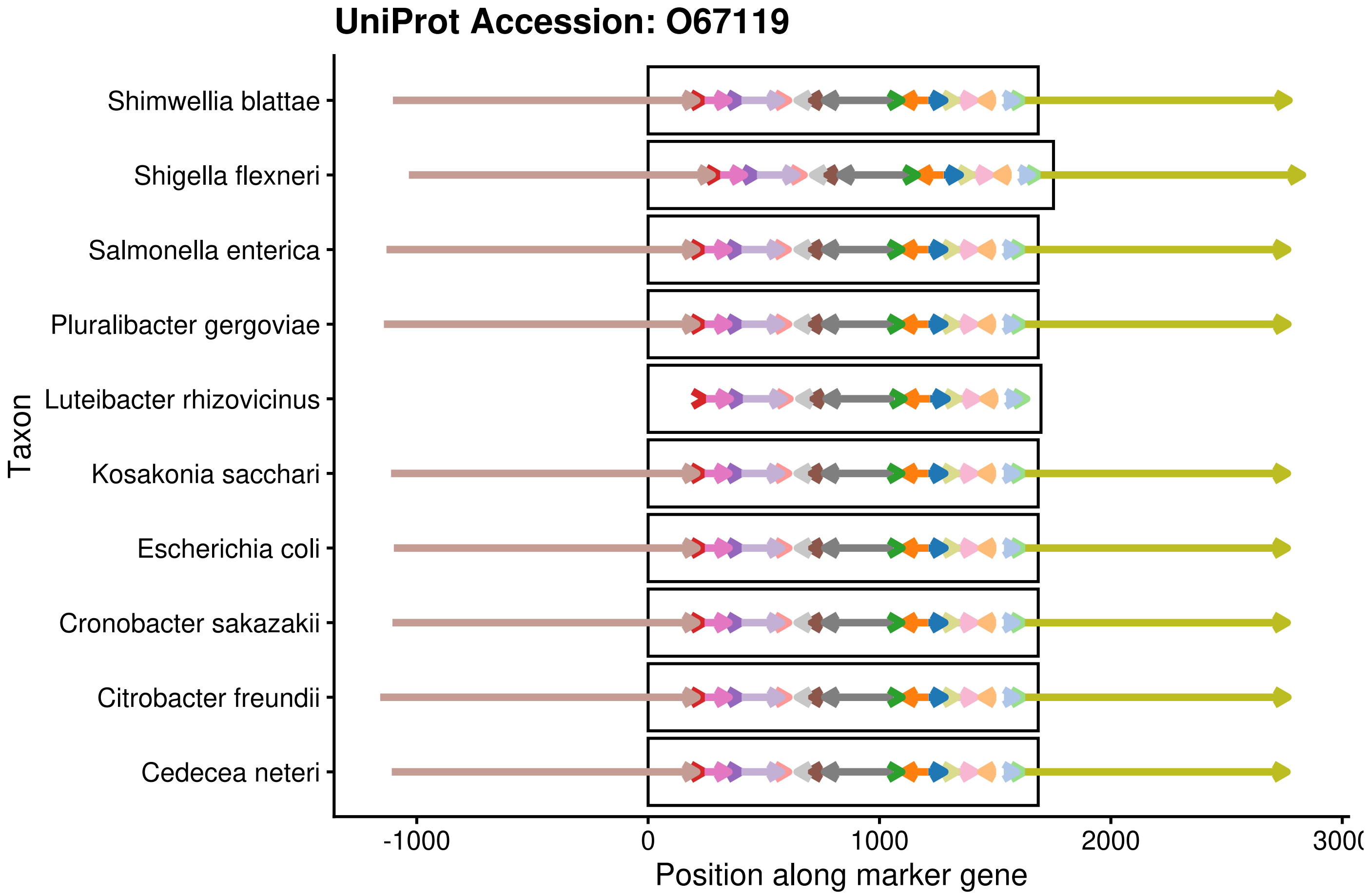
UniProt Accession: O67119
Shimwellia blattae
Shigella flexneri
Salmonella enterica
Pluralibacter gergoviae
Luteibacter rhizovicinus
Kosakonia sacchari
Escherichia coli
Cronobacter sakazakii
Citrobacter freundii
Cedecea neteri
Taxon
-1000
0
1000
2000
Position along marker gene

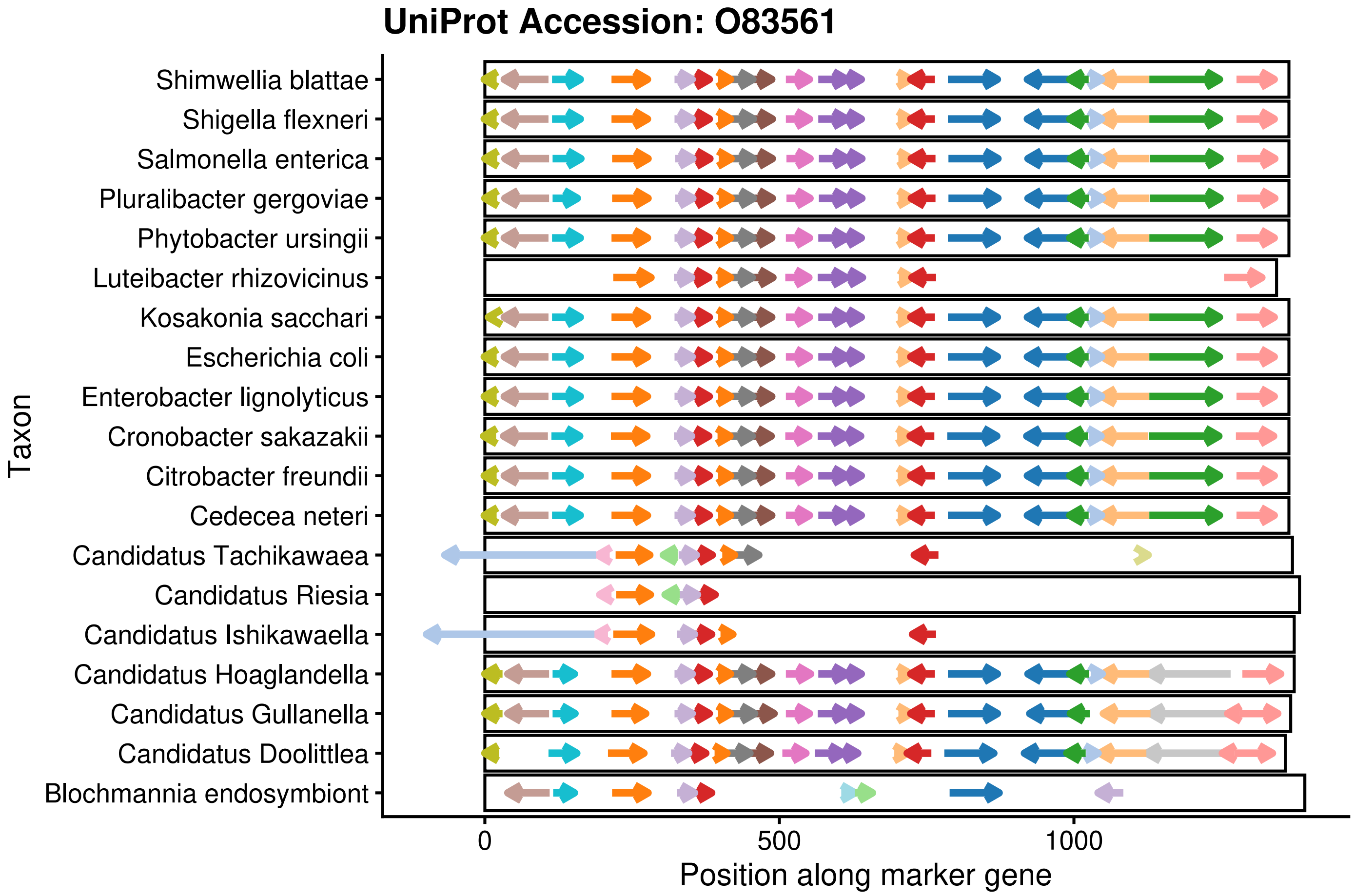

UniProt Accession: O83561
Shimwellia blattae
Shigella flexneri
Salmonella enterica
Pluralibacter gergoviae
Phytobacter ursingii
Luteibacter rhizovicinus
Kosakonia sacchari
Escherichia coli
Enterobacter lignolyticus
Cronobacter sakazakii
Citrobacter freundii
Cedecea neteri
Candidatus Tachikawaea
Candidatus Riesia
Candidatus Ishikawaella
Candidatus Hoaglandella
Candidatus Gullanella
Candidatus Doolittlea
Blochmannia endosymbiont
Taxon
0
500
1000
Position along marker gene

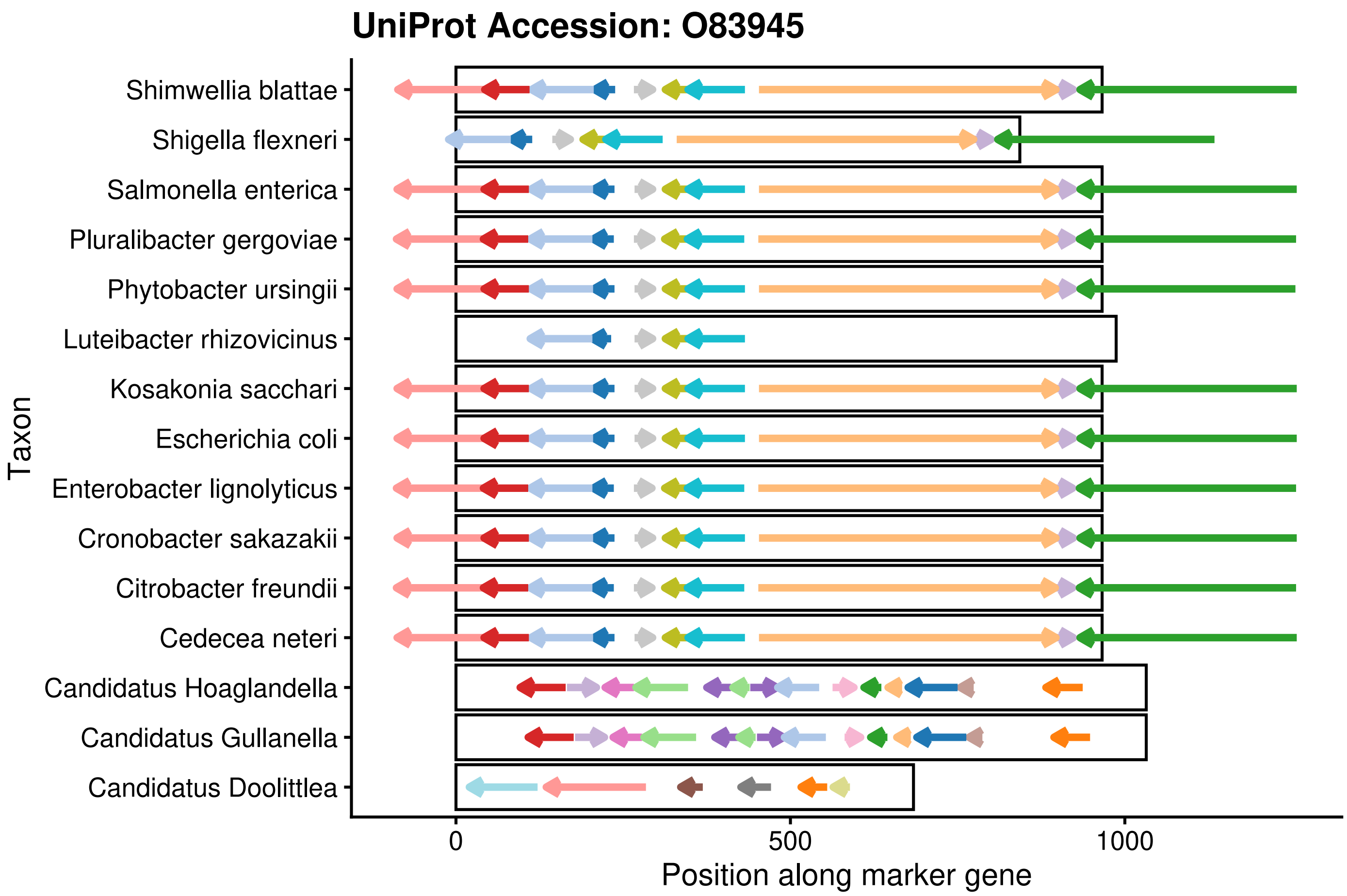

UniProt Accession: O83945
Shimwellia blattae
Shigella flexneri
Salmonella enterica
Pluralibacter gergoviae
Phytobacter ursingii
Luteibacter rhizovicinus
Kosakonia sacchari
Escherichia coli
Enterobacter lignolyticus
Cronobacter sakazakii
Citrobacter freundii
Cedecea neteri
Candidatus Hoaglandella
Candidatus Gullanella
Candidatus Doolittlea
Taxon
0
500
1000
Position along marker gene

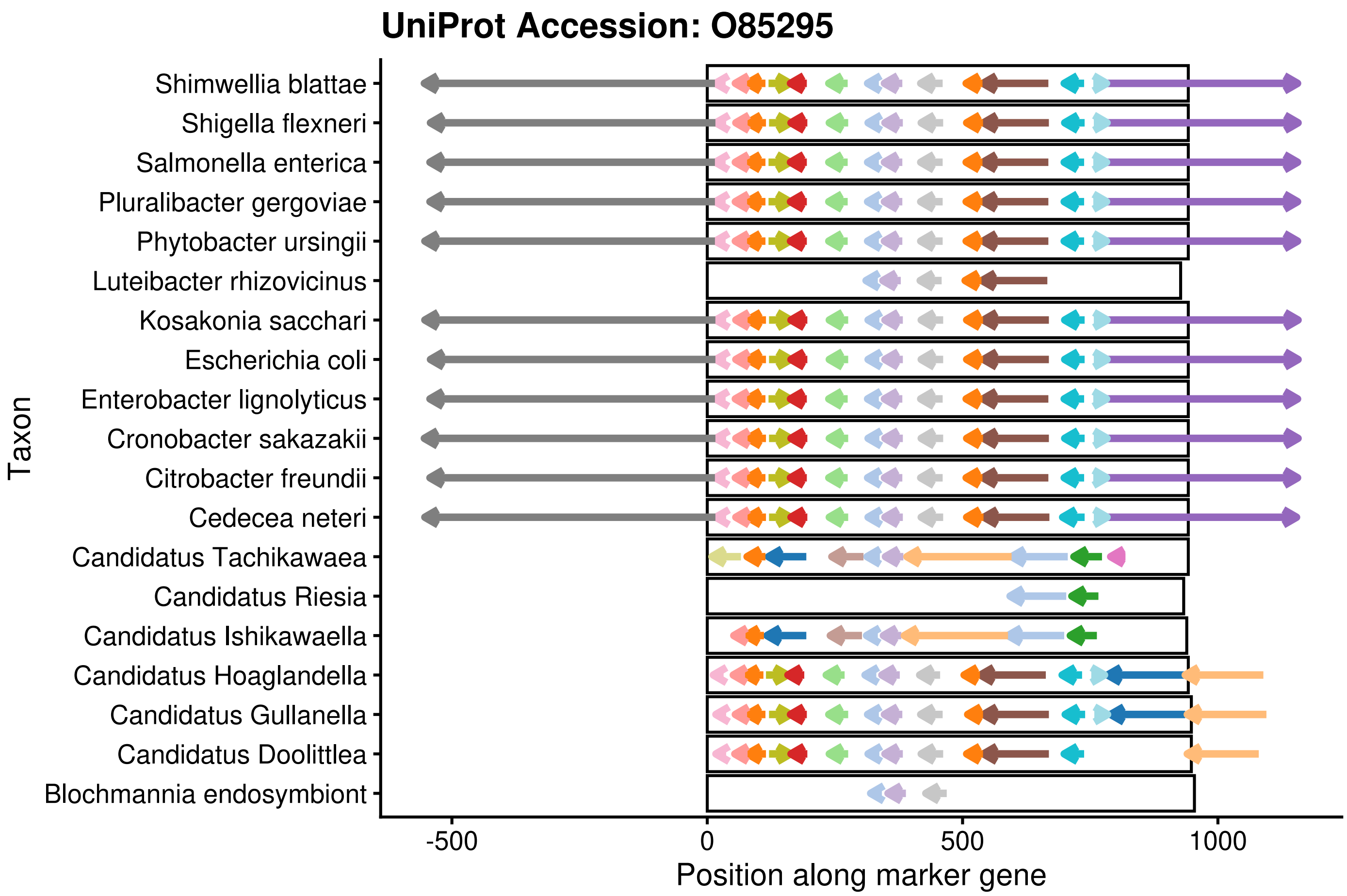
UniProt Accession: O85295
Shimwellia blattae
Shigella flexneri
Salmonella enterica
Pluralibacter gergoviae
Phytobacter ursingii
Luteibacter rhizovicinus
Kosakonia sacchari
Escherichia coli
Enterobacter lignolyticus
Cronobacter sakazakii
Citrobacter freundii
Cedecea neteri
Candidatus Tachikawaea
Candidatus Riesia
Candidatus Ishikawaella
Candidatus Hoaglandella
Candidatus Gullanella
Candidatus Doolittlea
Blochmannia endosymbiont
Taxon
-500
0
500
1000
Position along marker gene

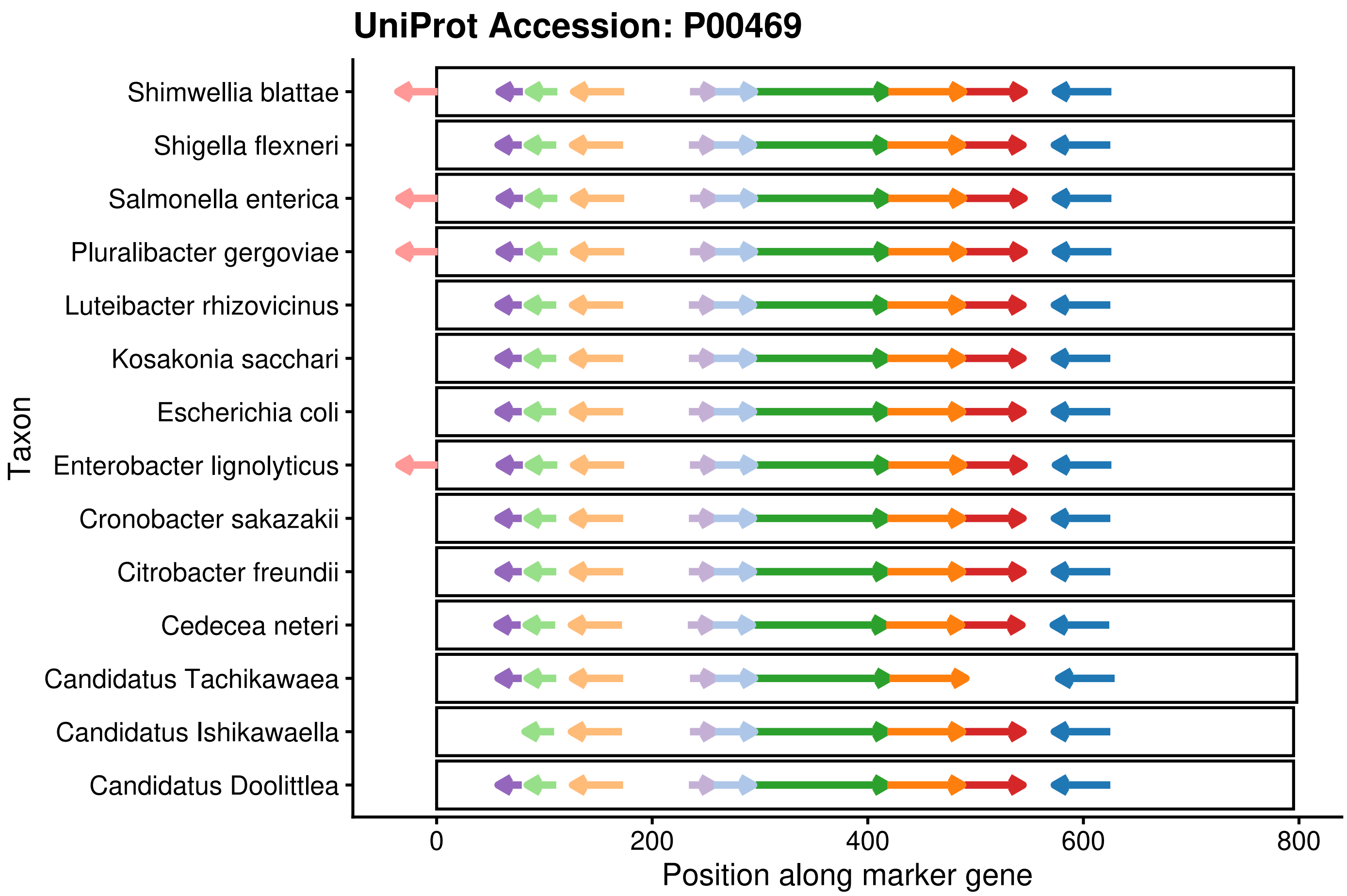
UniProt Accession: P00469
Shimwellia blattae
Shigella flexneri
Salmonella enterica
Pluralibacter gergoviae
Luteibacter rhizovicinus
Kosakonia sacchari
Escherichia coli
Enterobacter lignolyticus
Cronobacter sakazakii
Citrobacter freundii
Cedecea neteri
Candidatus Tachikawaea
Candidatus Ishikawaella
Candidatus Doolittlea
Taxon
0
200
400
600
800
Position along marker gene

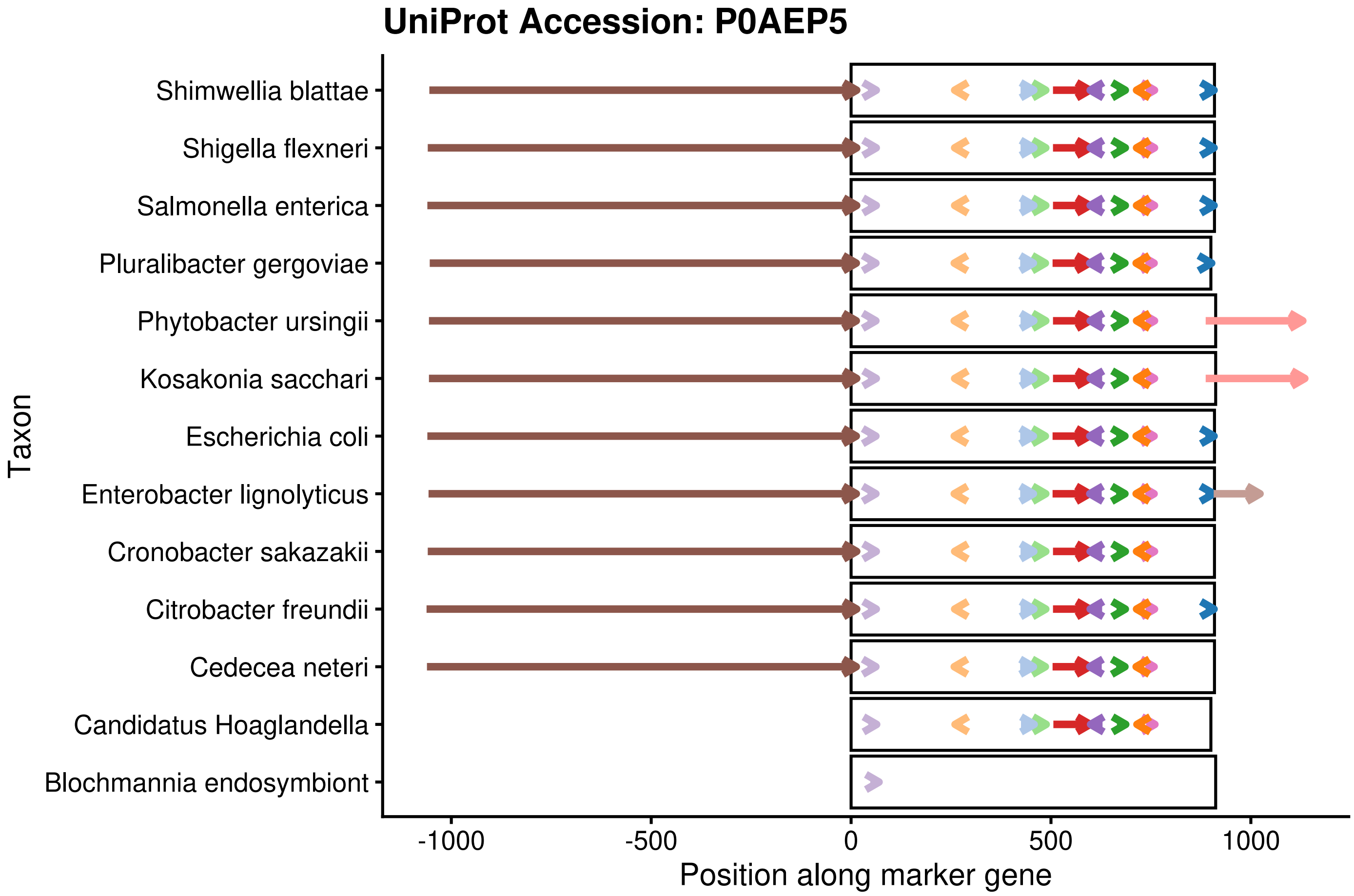

UniProt Accession: P0AEP5
Shimwellia blattae
Shigella flexneri
Salmonella enterica
Pluralibacter gergoviae
Phytobacter ursingii
Kosakonia sacchari
Escherichia coli
Enterobacter lignolyticus
Cronobacter sakazakii
Citrobacter freundii
Cedecea neteri
Candidatus Hoaglandella
Blochmannia endosymbiont
Taxon
-1000
-500
0
500
1000
Position along marker gene

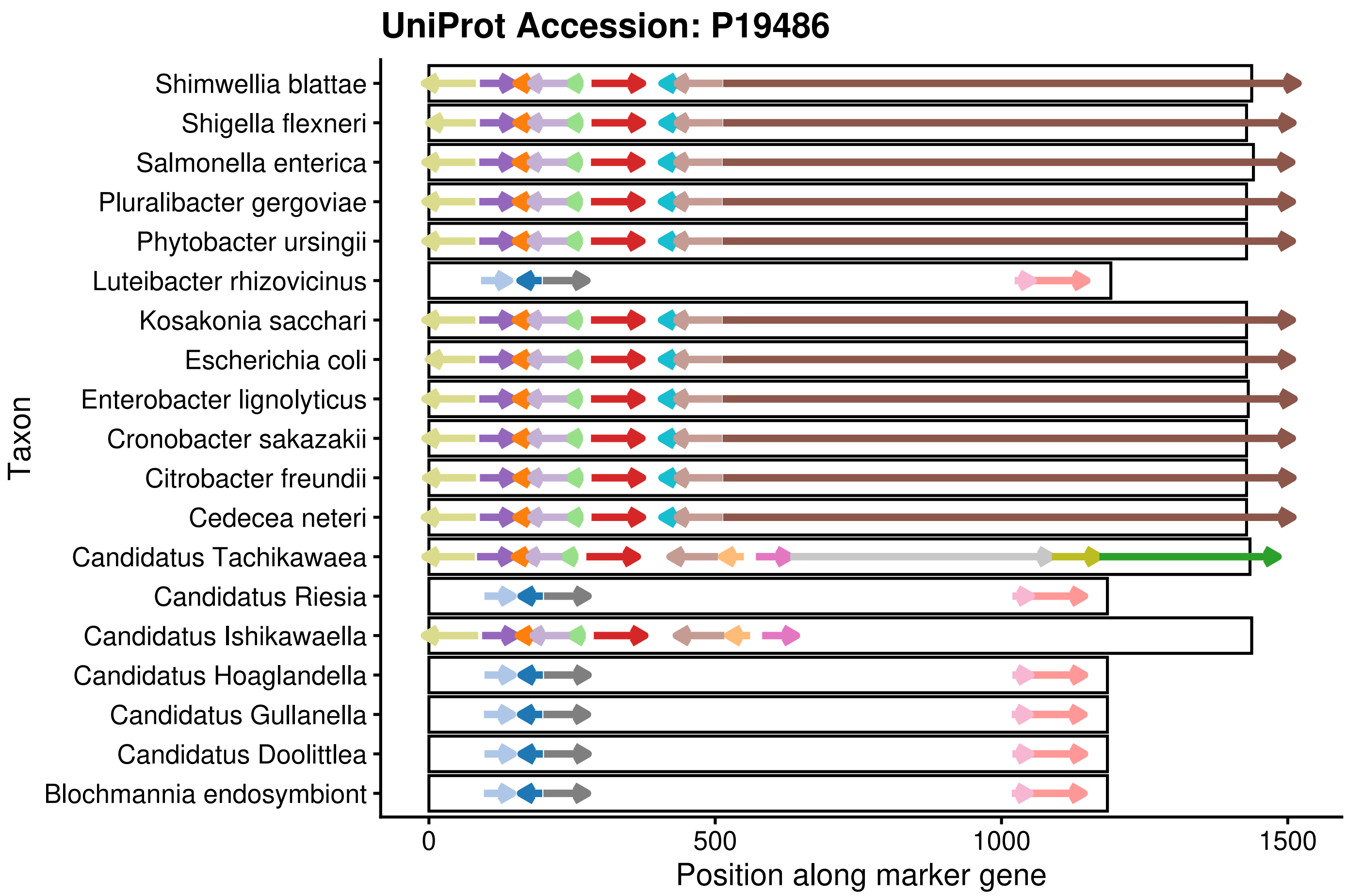

UniProt Accession: P19486
Taxon
Shimwellia blattae
Shigella flexneri
Salmonella enterica
Pluralibacter gergoviae
Phytobacter ursingii
Luteibacter rhizovicinus
Kosakonia sacchari
Escherichia coli
Enterobacter lignolyticus
Cronobacter sakazakii
Citrobacter freundii
Cedecea neteri
Candidatus Tachikawaea
Candidatus Riesia
Candidatus Ishikawaella
Candidatus Hoaglandella
Candidatus Gullanella
Candidatus Doolittlea
Blochmannia endosymbiont
0
500
1000
1500
Position along marker gene

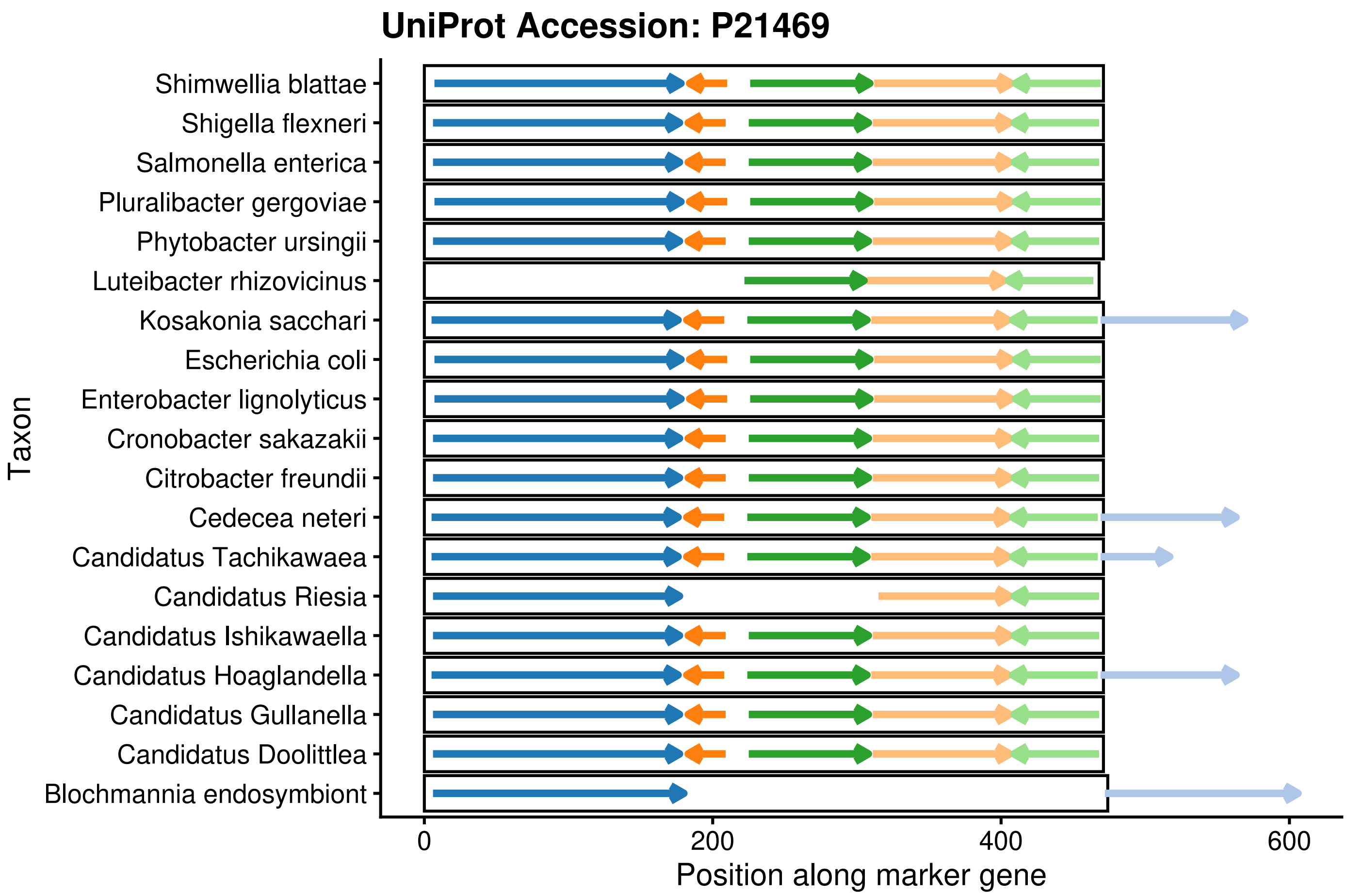

UniProt Accession: P21469
Shimwellia blattae
Shigella flexneri
Salmonella enterica
Pluralibacter gergoviae
Phytobacter ursingii
Luteibacter rhizovicinus
Kosakonia sacchari
Escherichia coli
Enterobacter lignolyticus
Cronobacter sakazakii
Citrobacter freundii
Cedecea neteri
Candidatus Tachikawaea
Candidatus Riesia
Candidatus Ishikawaella
Candidatus Hoaglandella
Candidatus Gullanella
Candidatus Doolittlea
Blochmannia endosymbiont
Taxon
0
200
400
600
Position along marker gene

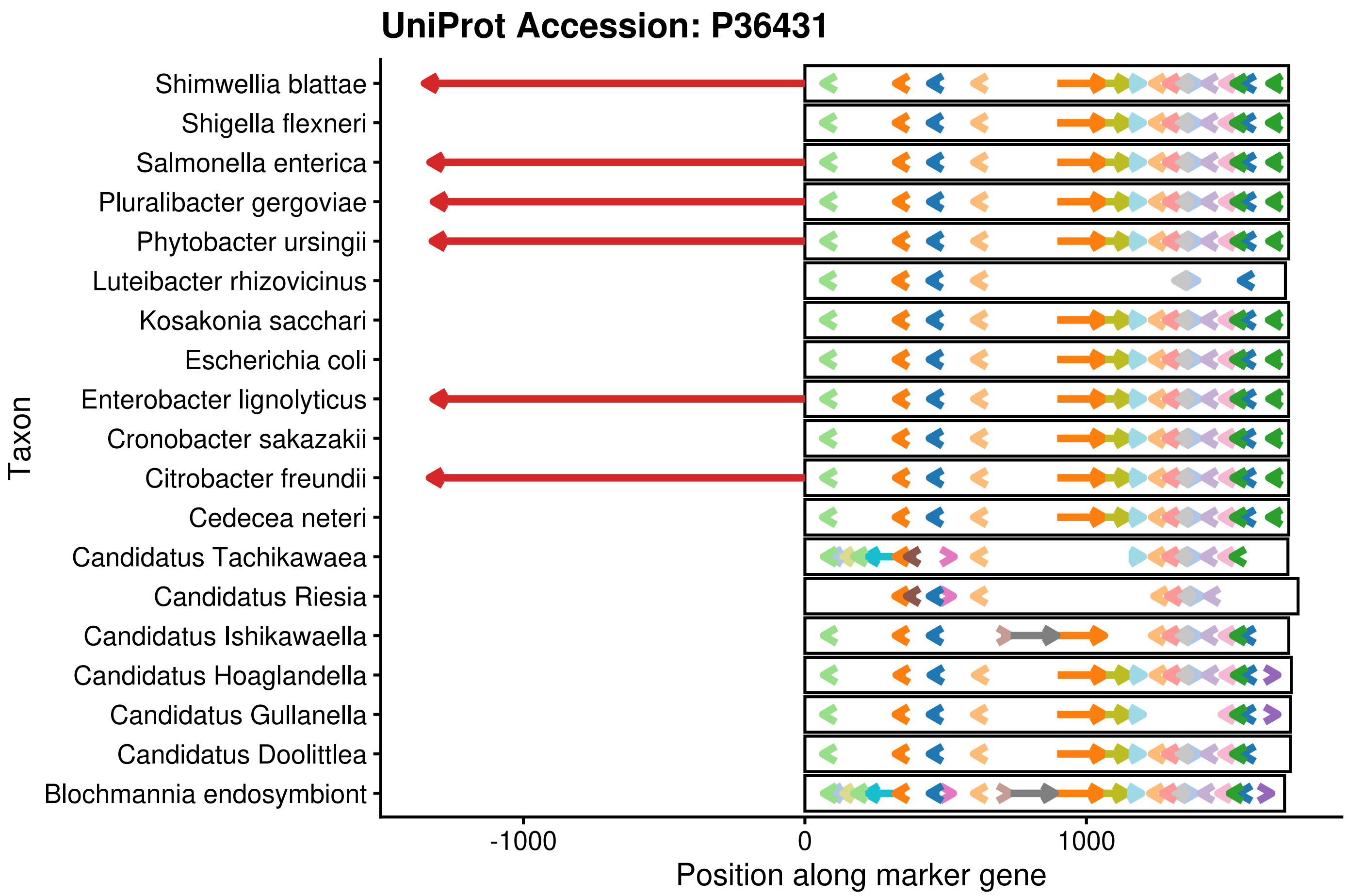
UniProt Accession: P36431
Taxon
Shimwellia blattae
Shigella flexneri
Salmonella enterica
Pluralibacter gergoviae
Phytobacter ursingii
Luteibacter rhizovicinus
Kosakonia sacchari
Escherichia coli
Enterobacter lignolyticus
Cronobacter sakazakii
Citrobacter freundii
Cedecea neteri
Candidatus Tachikawaea
Candidatus Riesia
Candidatus Ishikawaella
Candidatus Hoaglandella
Candidatus Gullanella
Candidatus Doolittlea
Blochmannia endosymbiont
-1000
0
1000
Position along marker gene

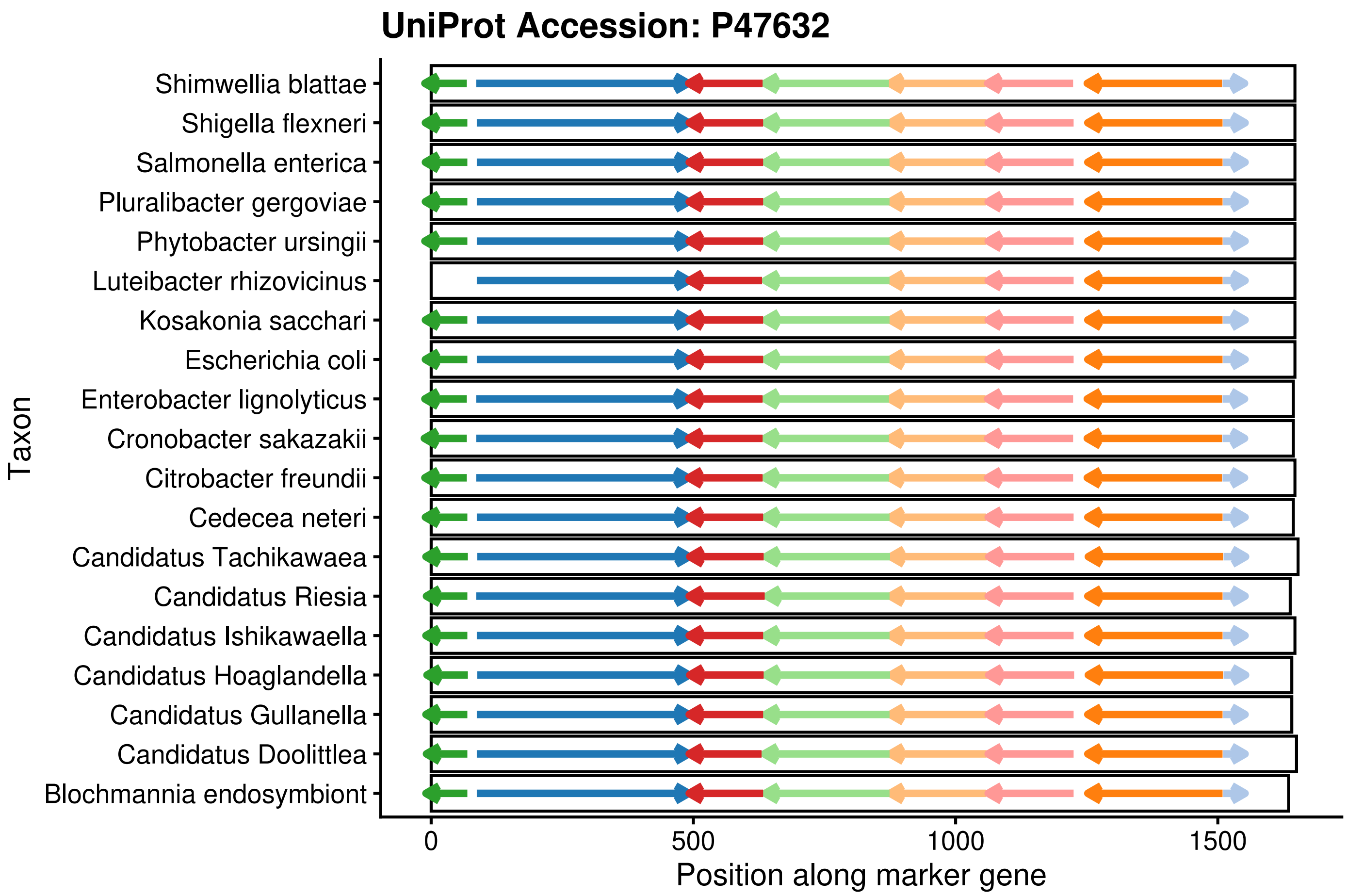
UniProt Accession: P47632
Shimwellia blattae
Shigella flexneri
Salmonella enterica
Pluralibacter gergoviae
Phytobacter ursingii
Luteibacter rhizovicinus
Kosakonia sacchari
Escherichia coli
Enterobacter lignolyticus
Cronobacter sakazakii
Citrobacter freundii
Cedecea neteri
Candidatus Tachikawaea
Candidatus Riesia
Candidatus Ishikawaella
Candidatus Hoaglandella
Candidatus Gullanella
Candidatus Doolittlea
Blochmannia endosymbiont
Taxon
0
500
1000
1500
Position along marker gene

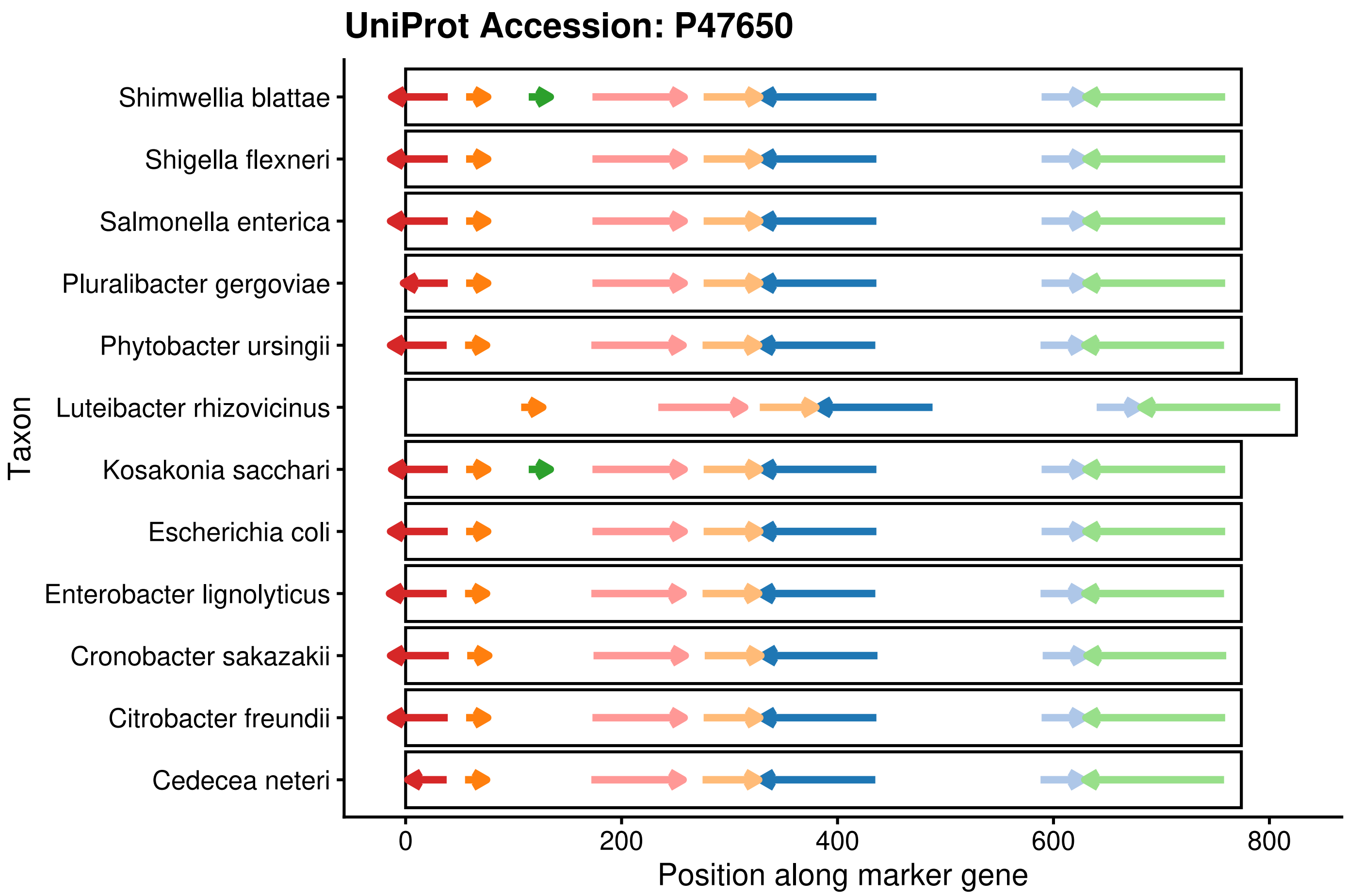
UniProt Accession: P47650
Shimwellia blattae
Shigella flexneri
Salmonella enterica
Pluralibacter gergoviae
Phytobacter ursingii
Luteibacter rhizovicinus
Kosakonia sacchari
Escherichia coli
Enterobacter lignolyticus
Cronobacter sakazakii
Citrobacter freundii
Cedecea neteri
Taxon
0
200
400
600
800
Position along marker gene

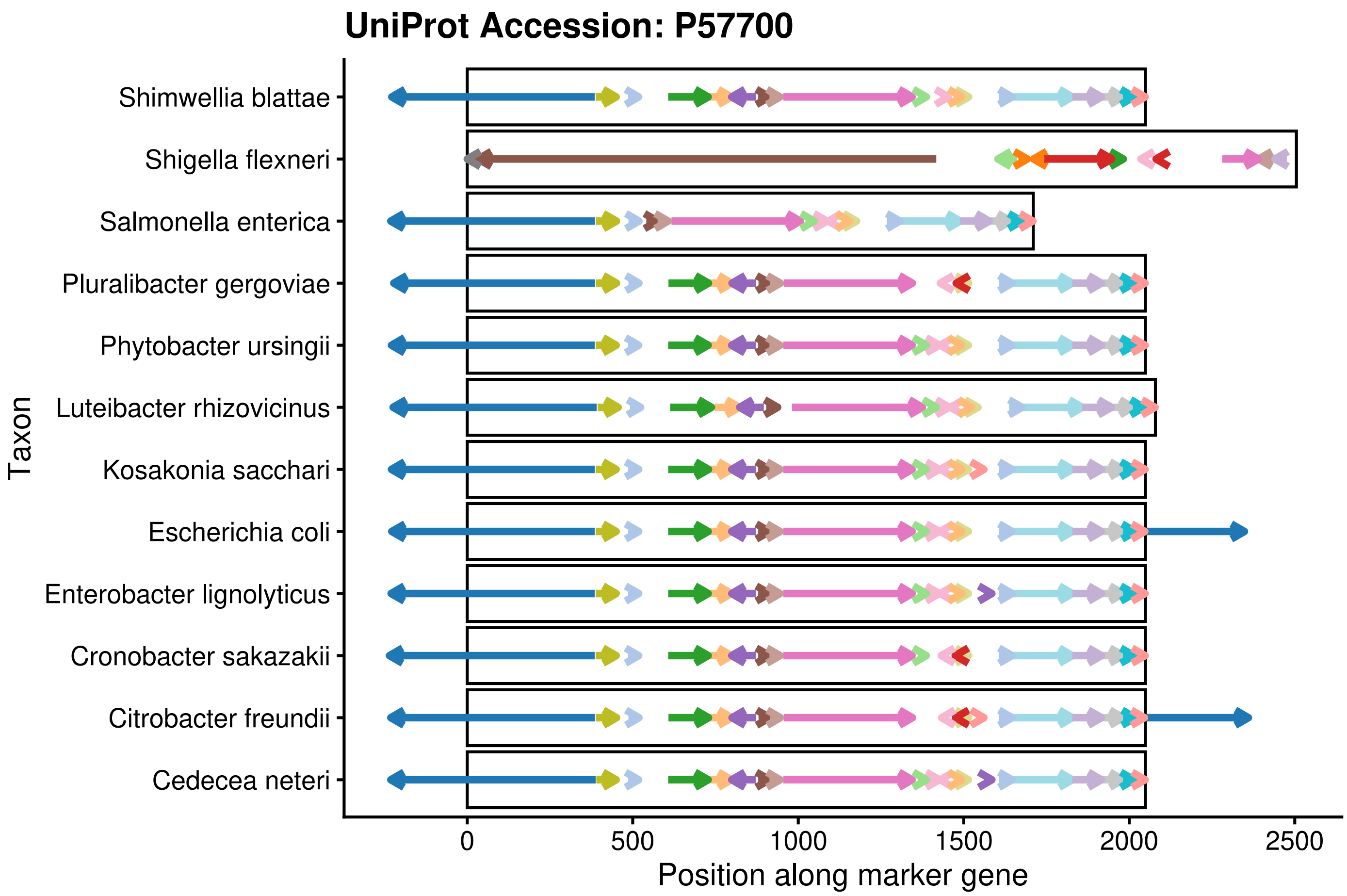

UniProt Accession: P57700
Taxon
Shimwellia blattae
Shigella flexneri
Salmonella enterica
Pluralibacter gergoviae
Phytobacter ursingii
Luteibacter rhizovicinus
Kosakonia sacchari
Escherichia coli
Enterobacter lignolyticus
Cronobacter sakazakii
Citrobacter freundii
Cedecea neteri
0
500
1000
1500
2000
2500
Position along marker gene

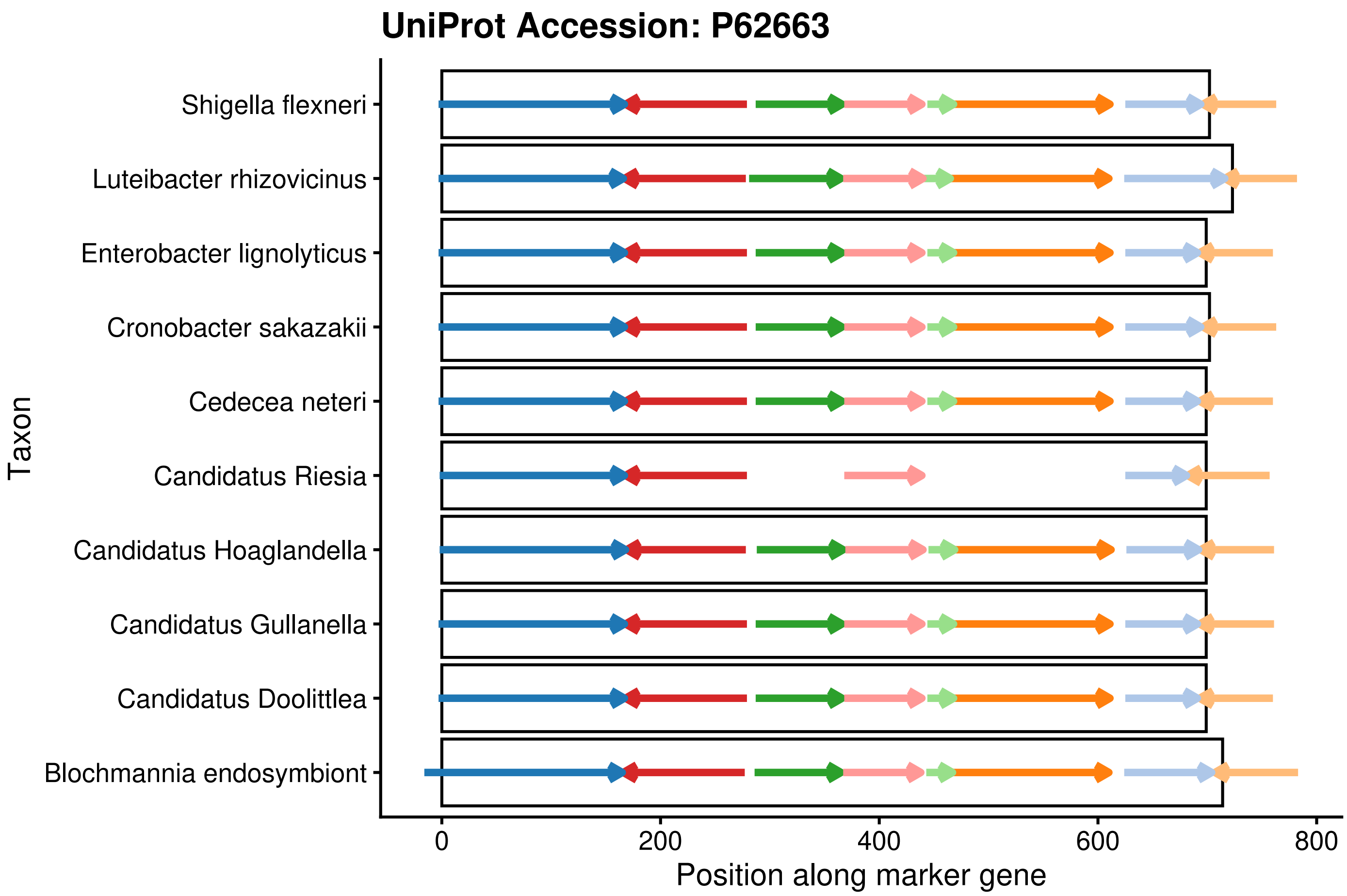
UniProt Accession: P62663
Taxon
Shigella flexneri
Luteibacter rhizovicinus
Enterobacter lignolyticus
Cronobacter sakazakii
Cedecea neteri
Candidatus Riesia
Candidatus Hoaglandella
Candidatus Gullanella
Candidatus Doolittlea
Blochmannia endosymbiont
0
200
400
600
800
Position along marker gene

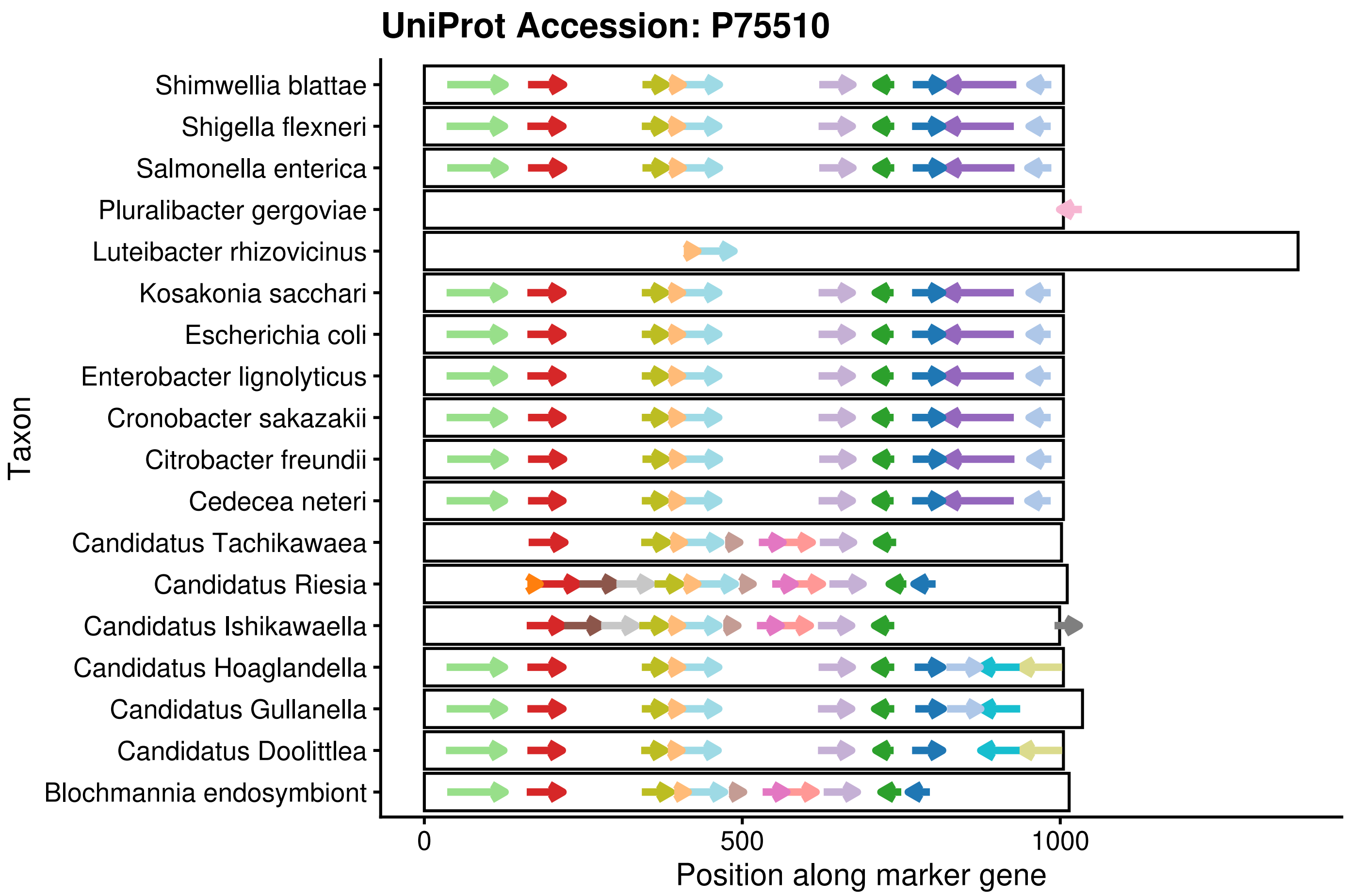

UniProt Accession: P75510
Shimwellia blattae
Shigella flexneri
Salmonella enterica
Pluralibacter gergoviae
Luteibacter rhizovicinus
Kosakonia sacchari
Escherichia coli
Enterobacter lignolyticus
Cronobacter sakazakii
Citrobacter freundii
Cedecea neteri
Candidatus Tachikawaea
Candidatus Riesia
Candidatus Ishikawaella
Candidatus Hoaglandella
Candidatus Gullanella
Candidatus Doolittlea
Blochmannia endosymbiont
Taxon
0
500
1000
Position along marker gene

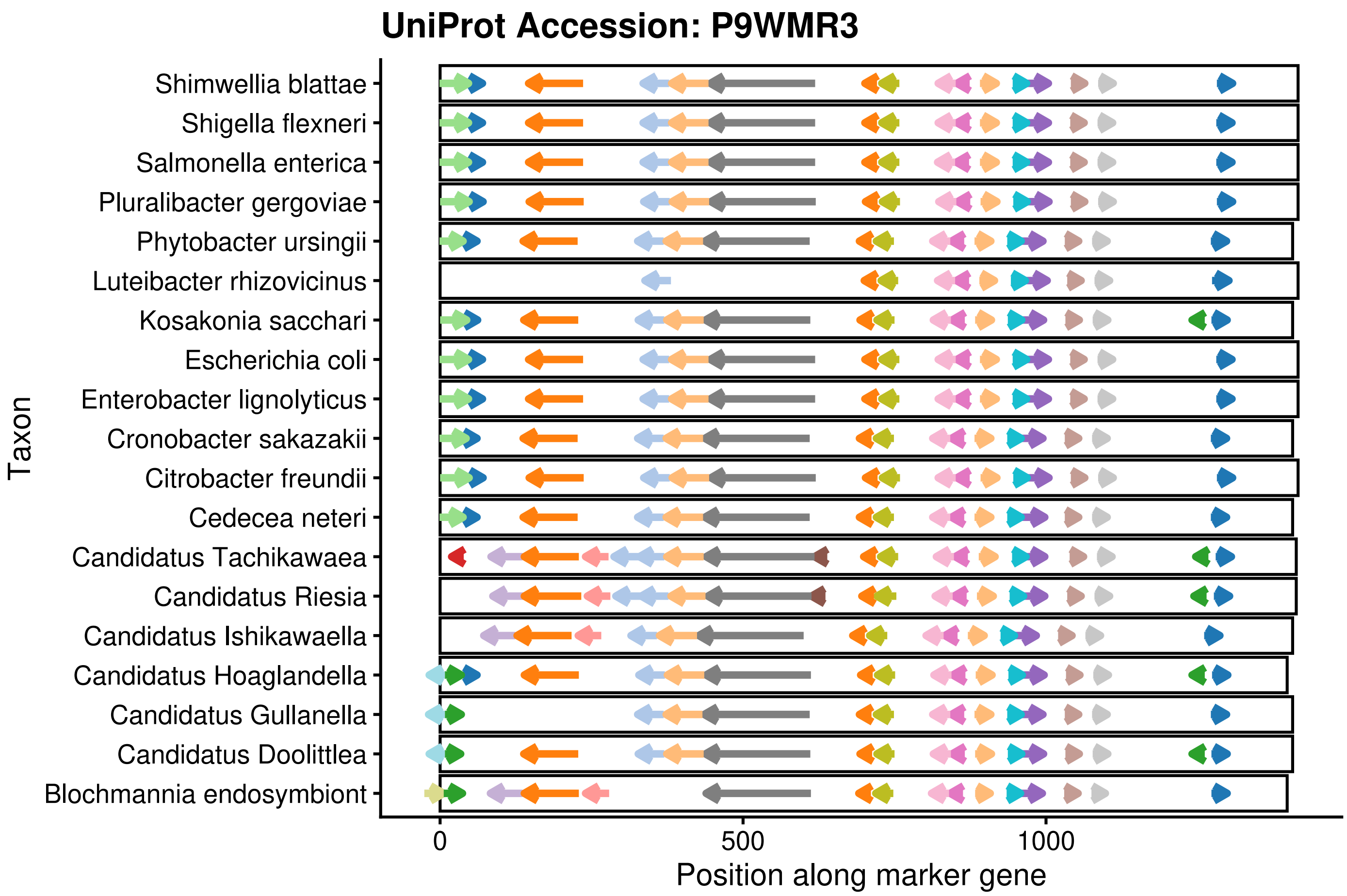
UniProt Accession: P9WMR3
Taxon
Shimwellia blattae
Shigella flexneri
Salmonella enterica
Pluralibacter gergoviae
Phytobacter ursingii
Luteibacter rhizovicinus
Kosakonia sacchari
Escherichia coli
Enterobacter lignolyticus
Cronobacter sakazakii
Citrobacter freundii
Cedecea neteri
Candidatus Tachikawaea
Candidatus Riesia
Candidatus Ishikawaella
Candidatus Hoaglandella
Candidatus Gullanella
Candidatus Doolittlea
Blochmannia endosymbiont
0
500
1000
Position along marker gene

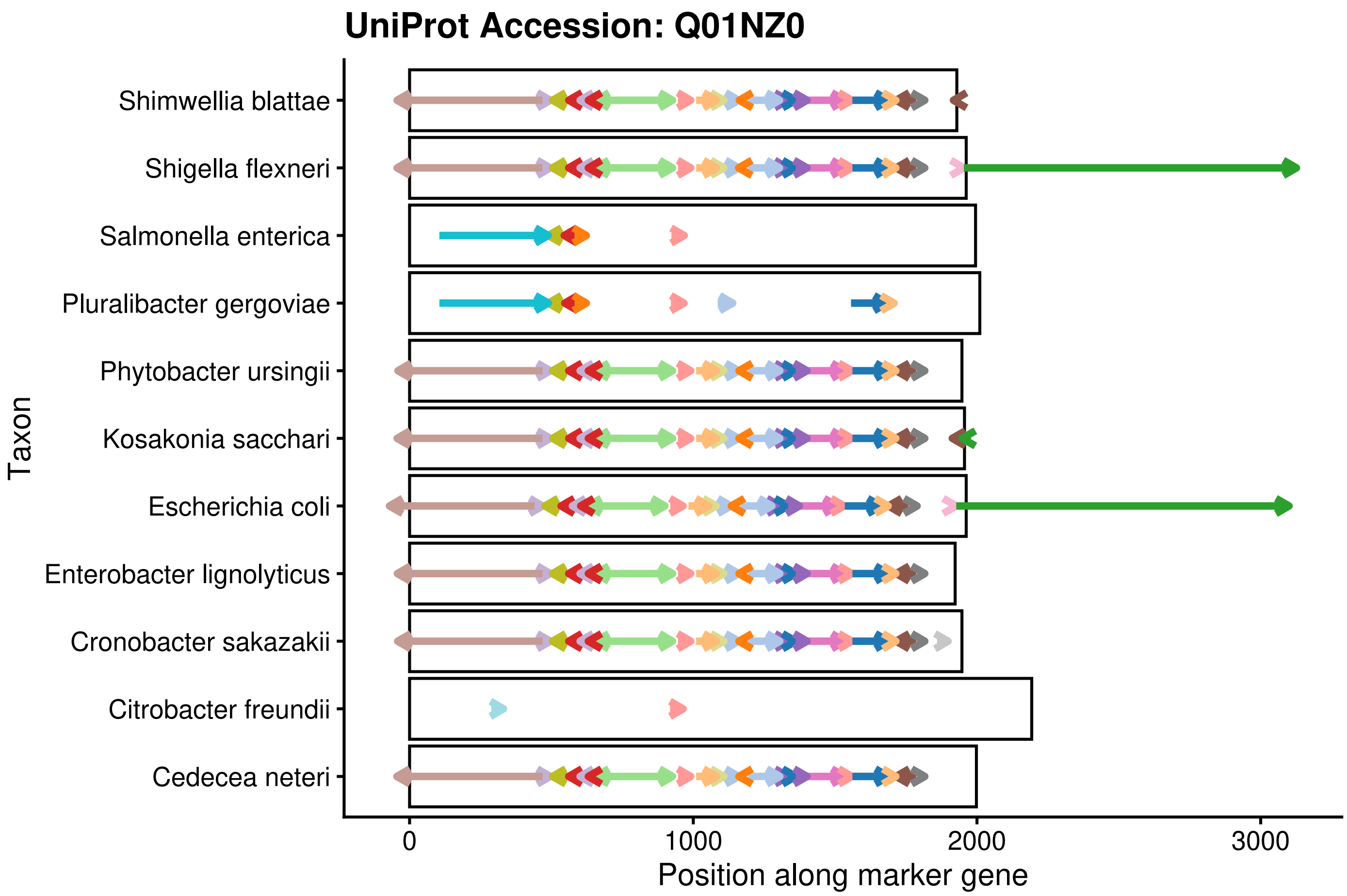
UniProt Accession: Q01NZ0
Taxon
Shimwellia blattae
Shigella flexneri
Salmonella enterica
Pluralibacter gergoviae
Phytobacter ursingii
Kosakonia sacchari
Escherichia coli
Enterobacter lignolyticus
Cronobacter sakazakii
Citrobacter freundii
Cedecea neteri
0
1000
2000
3000
Position along marker gene

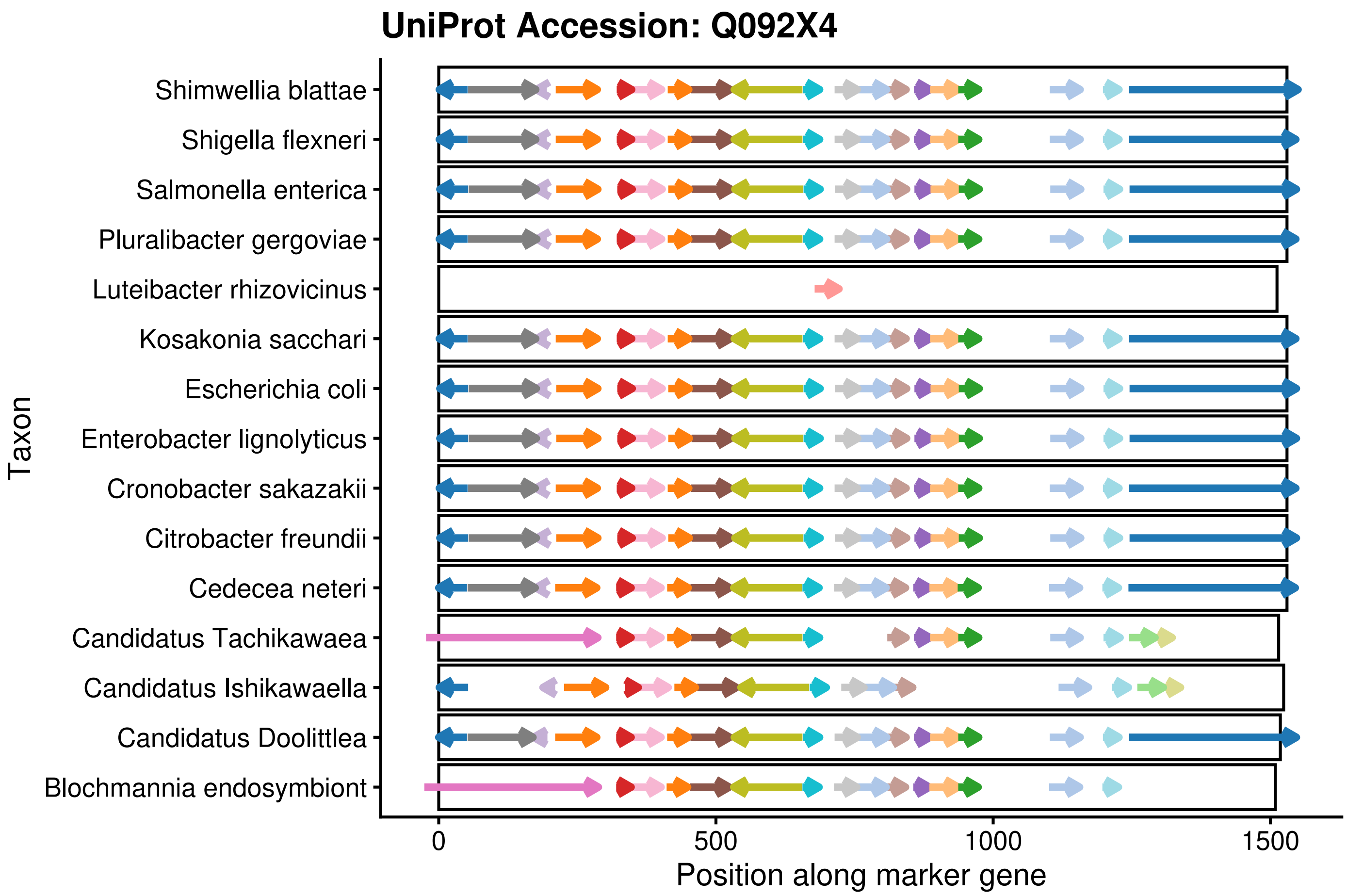
UniProt Accession: Q092X4
Shimwellia blattae
Shigella flexneri
Salmonella enterica
Pluralibacter gergoviae
Luteibacter rhizovicinus
Kosakonia sacchari
Escherichia coli
Enterobacter lignolyticus
Cronobacter sakazakii
Citrobacter freundii
Cedecea neteri
Candidatus Tachikawaea
Candidatus Ishikawaella
Candidatus Doolittlea
Blochmannia endosymbiont
Taxon
0
500
1000
1500
Position along marker gene

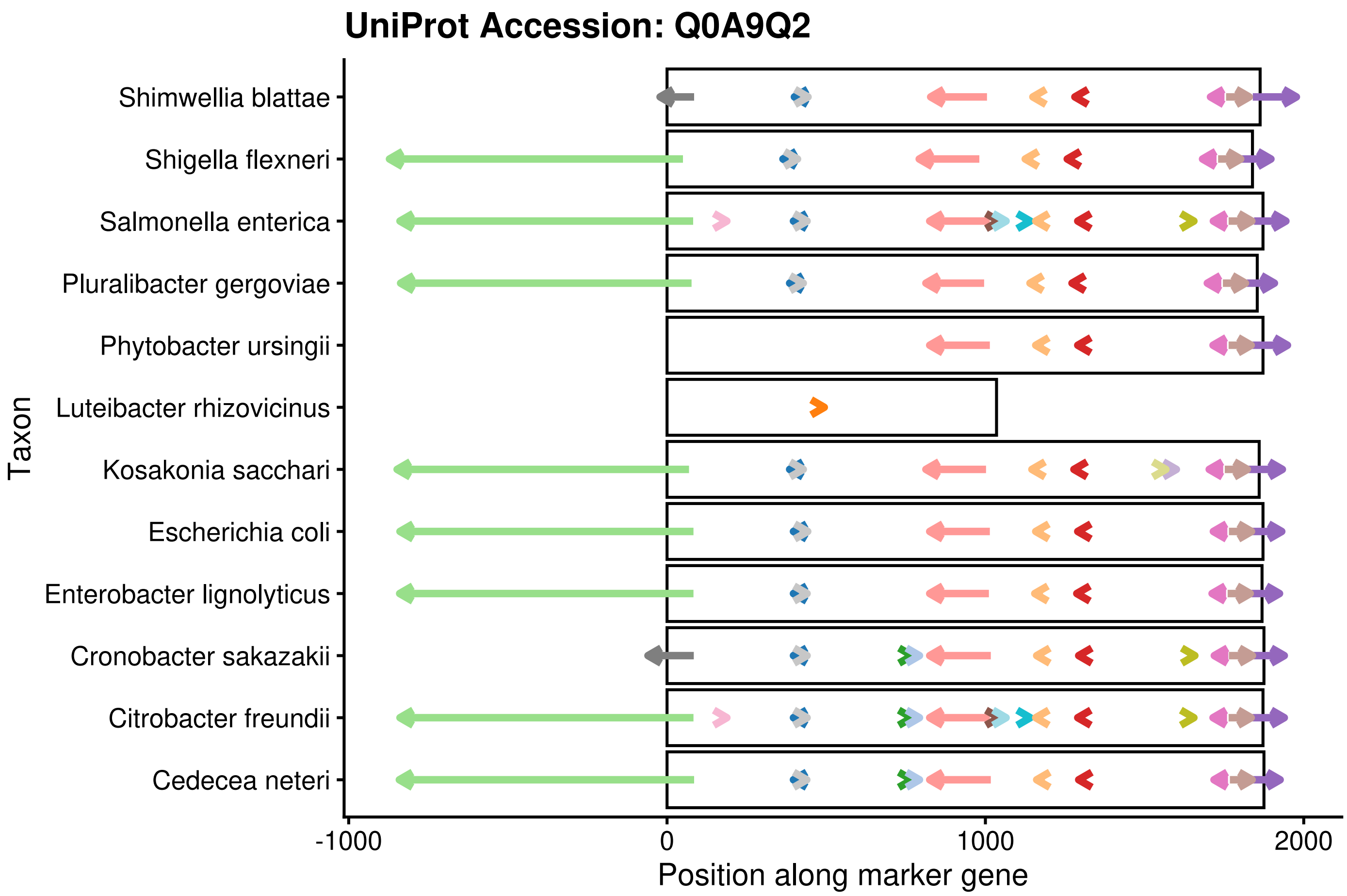

UniProt Accession: Q0A9Q2
Shimwellia blattae
Shigella flexneri
Salmonella enterica
Pluralibacter gergoviae
Phytobacter ursingii
Luteibacter rhizovicinus
Kosakonia sacchari
Escherichia coli
Enterobacter lignolyticus
Cronobacter sakazakii
Citrobacter freundii
Cedecea neteri
Taxon
-1000
0
1000
2000
Position along marker gene

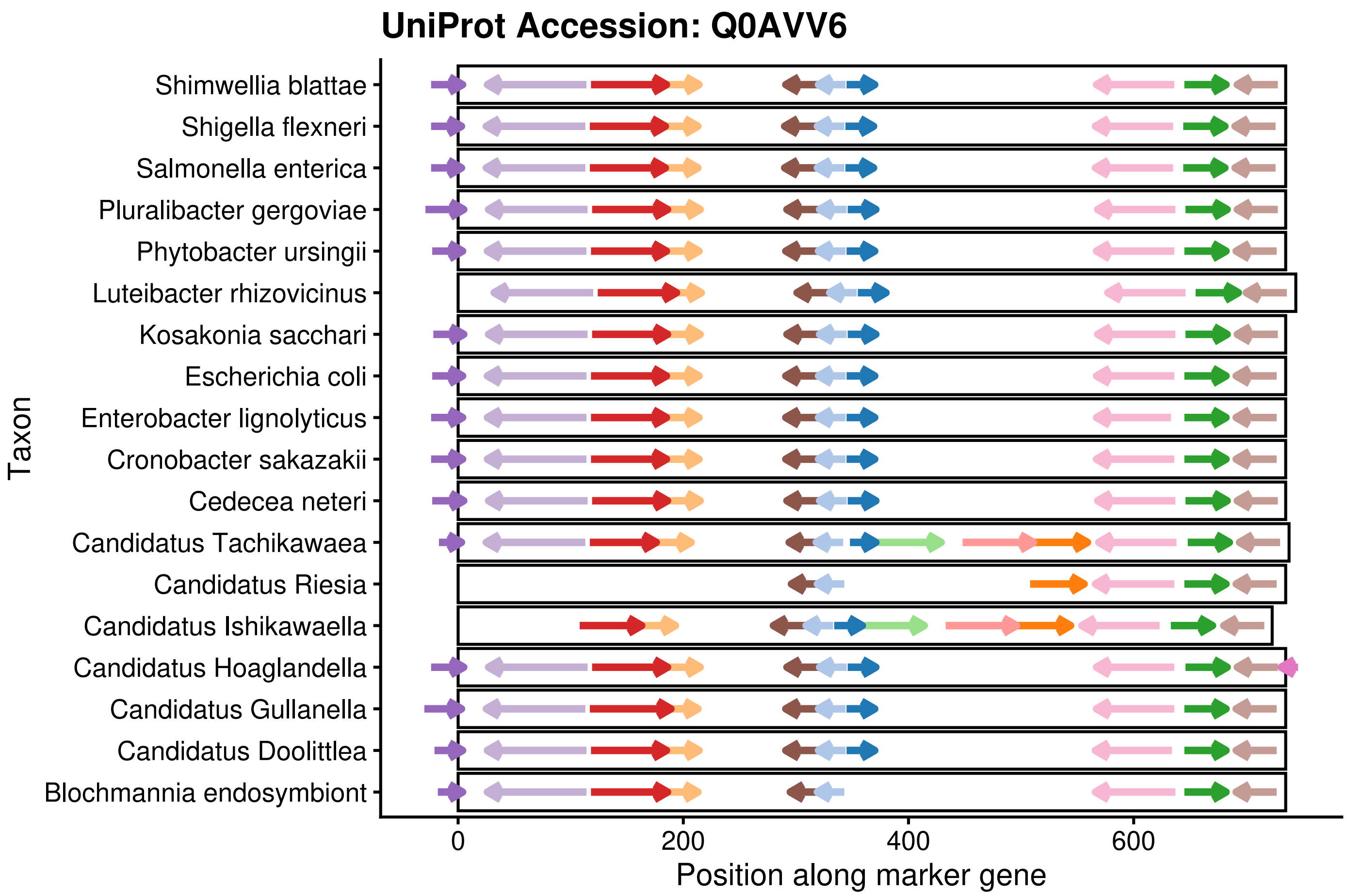

UniProt Accession: Q0AVV6
Shimwellia blattae
Shigella flexneri
Salmonella enterica
Pluralibacter gergoviae
Phytobacter ursingii
Luteibacter rhizovicinus
Kosakonia sacchari
Escherichia coli
Enterobacter lignolyticus
Cronobacter sakazakii
Cedecea neteri
Candidatus Tachikawaea
Candidatus Riesia
Candidatus Ishikawaella
Candidatus Hoaglandella
Candidatus Gullanella
Candidatus Doolittlea
Blochmannia endosymbiont
Taxon
0
200
400
600
Position along marker gene

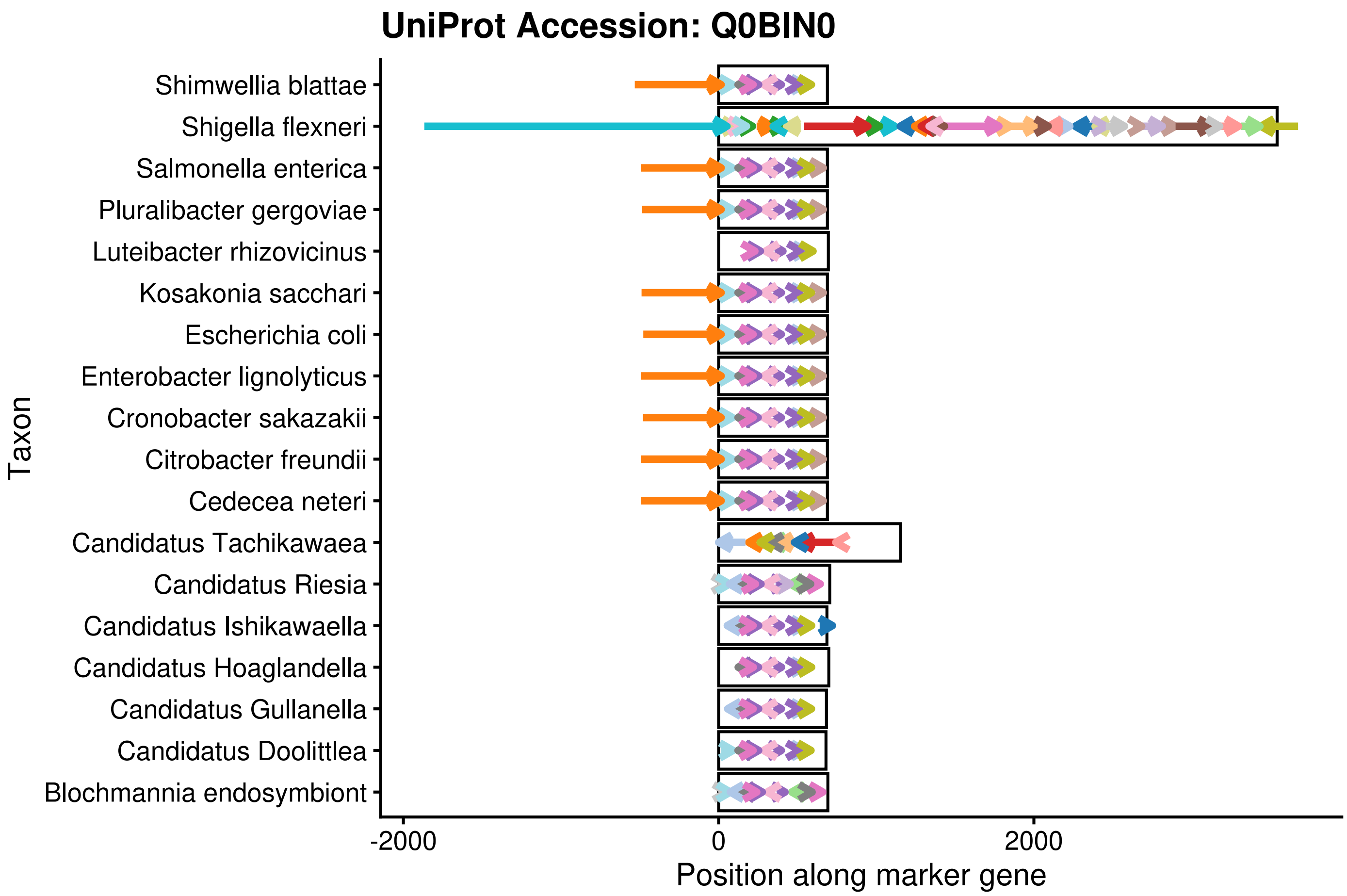
UniProt Accession: Q0BIN0
Taxon
Shimwellia blattae
Shigella flexneri
Salmonella enterica
Pluralibacter gergoviae
Luteibacter rhizovicinus
Kosakonia sacchari
Escherichia coli
Enterobacter lignolyticus
Cronobacter sakazakii
Citrobacter freundii
Cedecea neteri
Candidatus Tachikawaea
Candidatus Riesia
Candidatus Ishikawaella
Candidatus Hoaglandella
Candidatus Gullanella
Candidatus Doolittlea
Blochmannia endosymbiont
-2000
0
2000
Position along marker gene

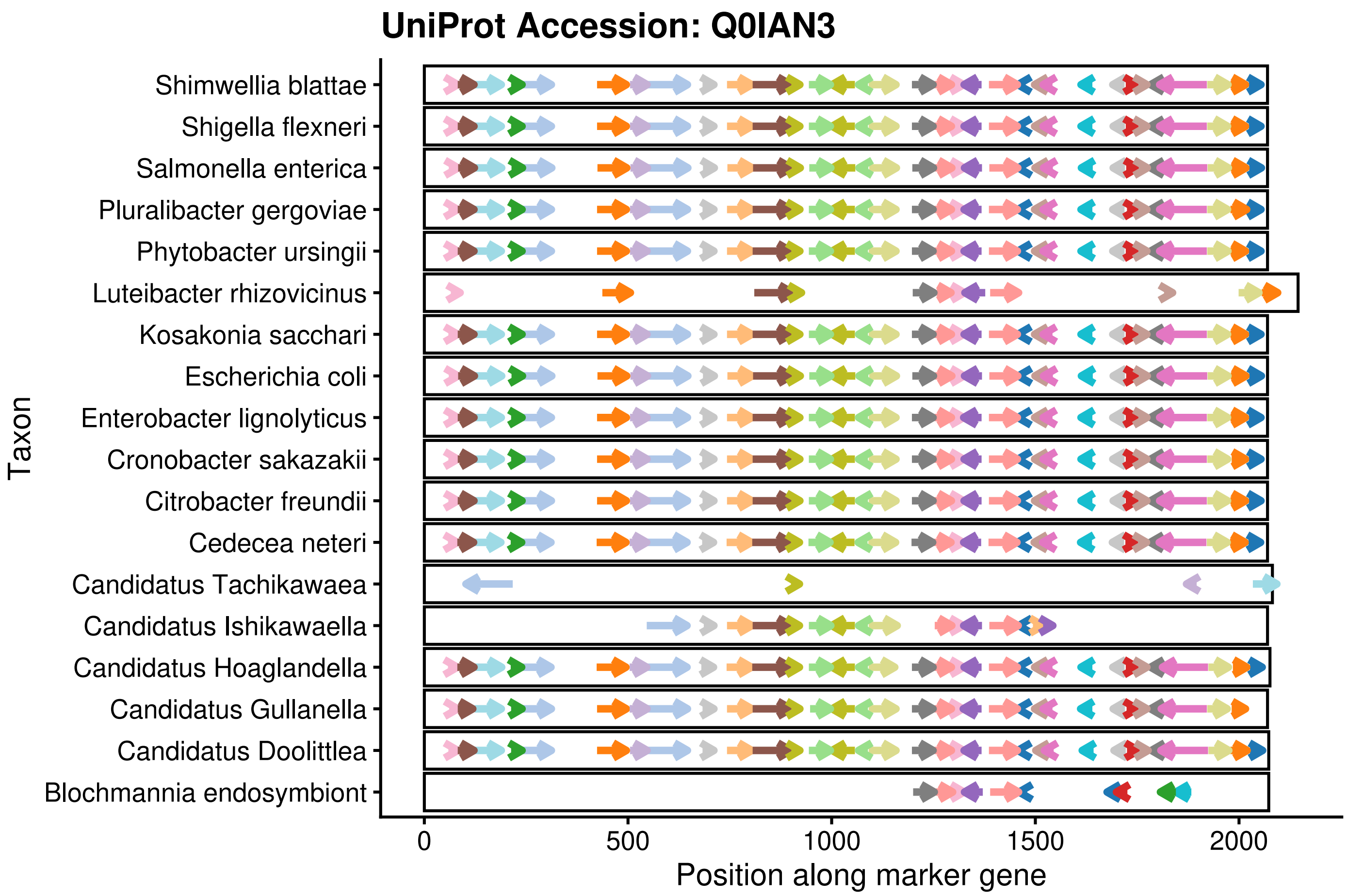

UniProt Accession: Q0IAN3
Taxon
Shimwellia blattae
Shigella flexneri
Salmonella enterica
Pluralibacter gergoviae
Phytobacter ursingii
Luteibacter rhizovicinus
Kosakonia sacchari
Escherichia coli
Enterobacter lignolyticus
Cronobacter sakazakii
Citrobacter freundii
Cedecea neteri
Candidatus Tachikawaea
Candidatus Ishikawaella
Candidatus Hoaglandella
Candidatus Gullanella
Candidatus Doolittlea
Blochmannia endosymbiont
0
500
1000
1500
2000
Position along marker gene

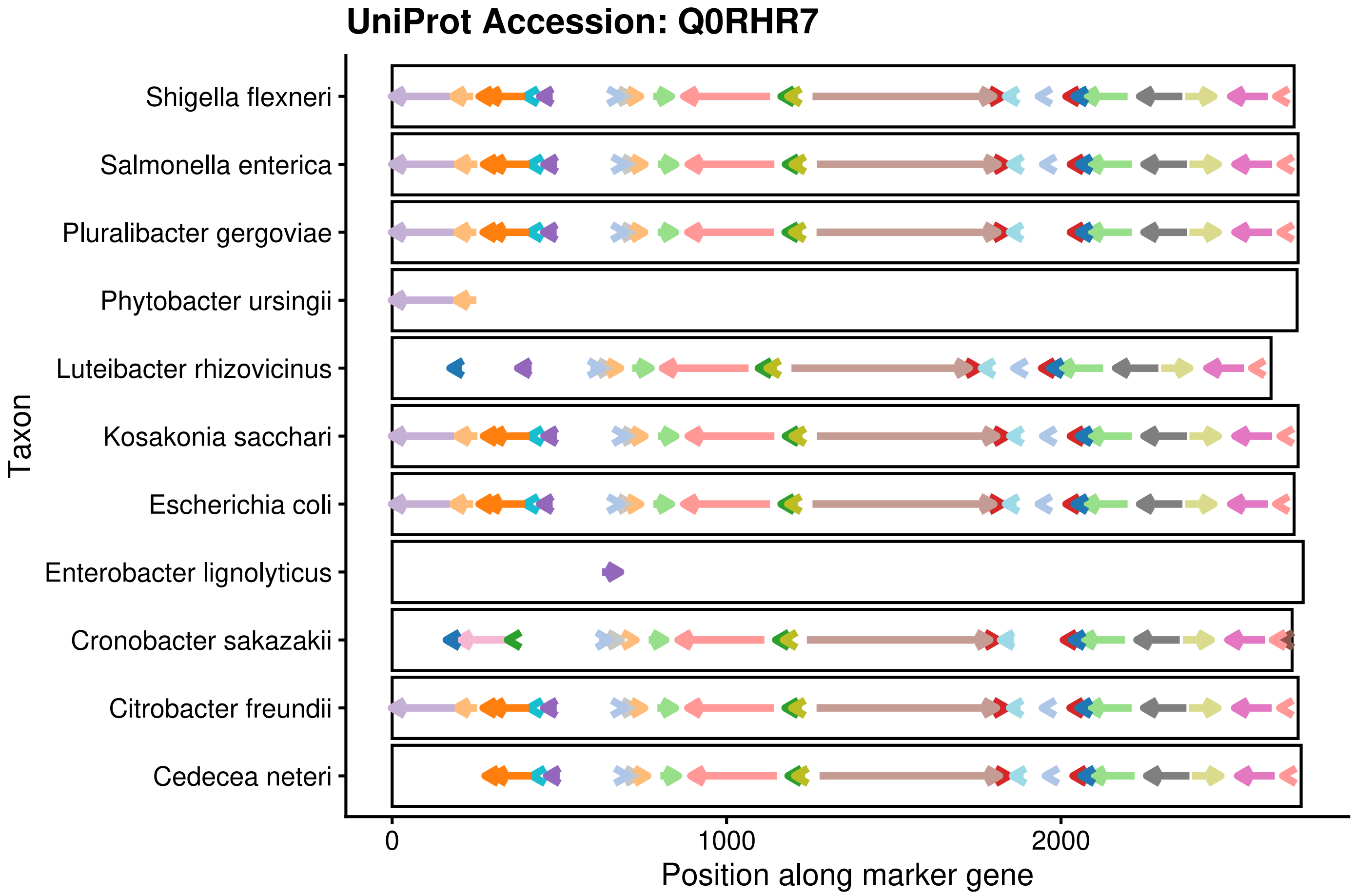

UniProt Accession: Q0RHR7
Taxon
Shigella flexneri
Salmonella enterica
Pluralibacter gergoviae
Phytobacter ursingii
Luteibacter rhizovicinus
Kosakonia sacchari
Escherichia coli
Enterobacter lignolyticus
Cronobacter sakazakii
Citrobacter freundii
Cedecea neteri
0
1000
2000
Position along marker gene

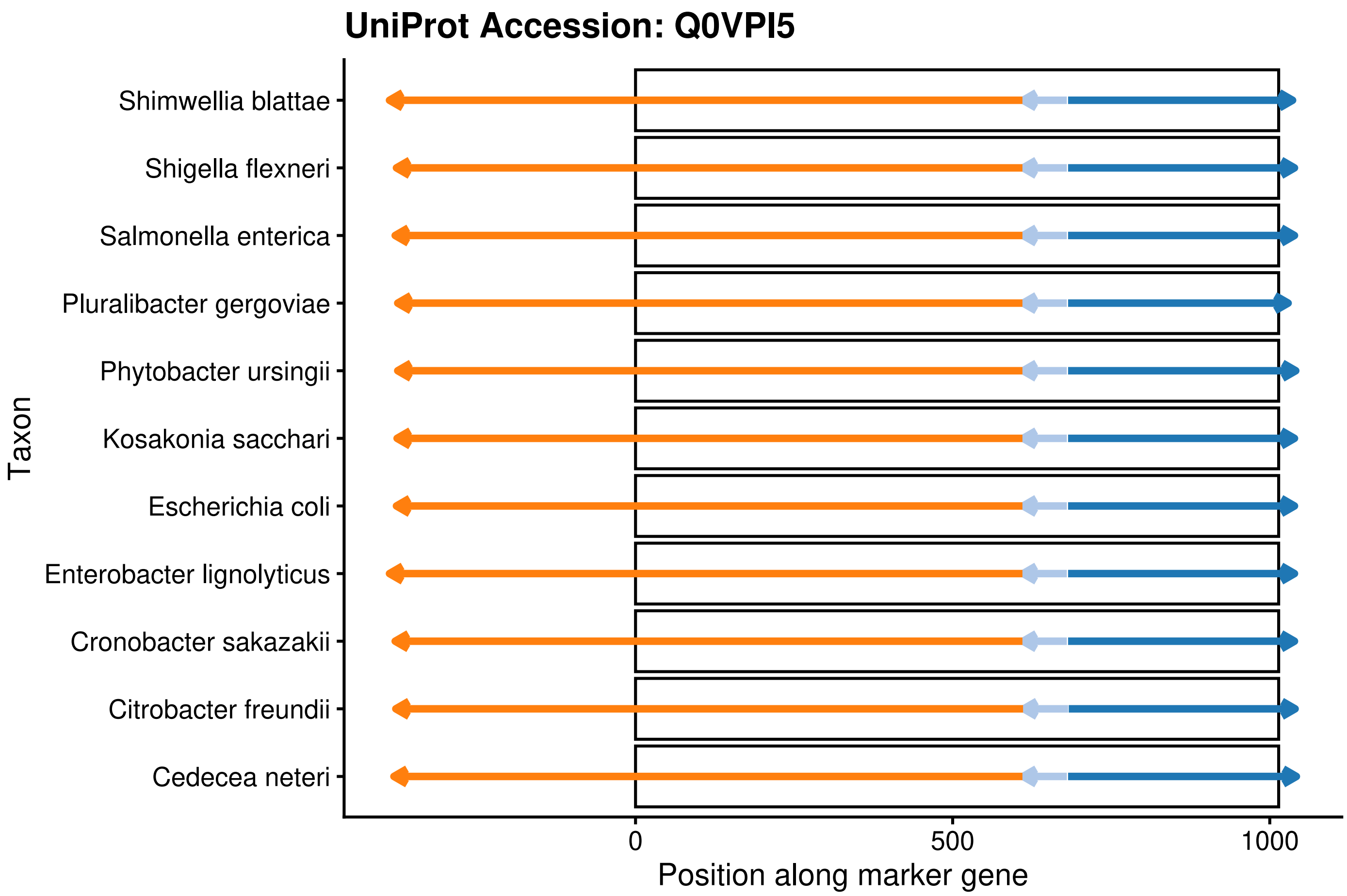
UniProt Accession: Q0VPI5
Taxon
Shimwellia blattae
Shigella flexneri
Salmonella enterica
Pluralibacter gergoviae
Phytobacter ursingii
Kosakonia sacchari
Escherichia coli
Enterobacter lignolyticus
Cronobacter sakazakii
Citrobacter freundii
Cedecea neteri
0
500
1000
Position along marker gene

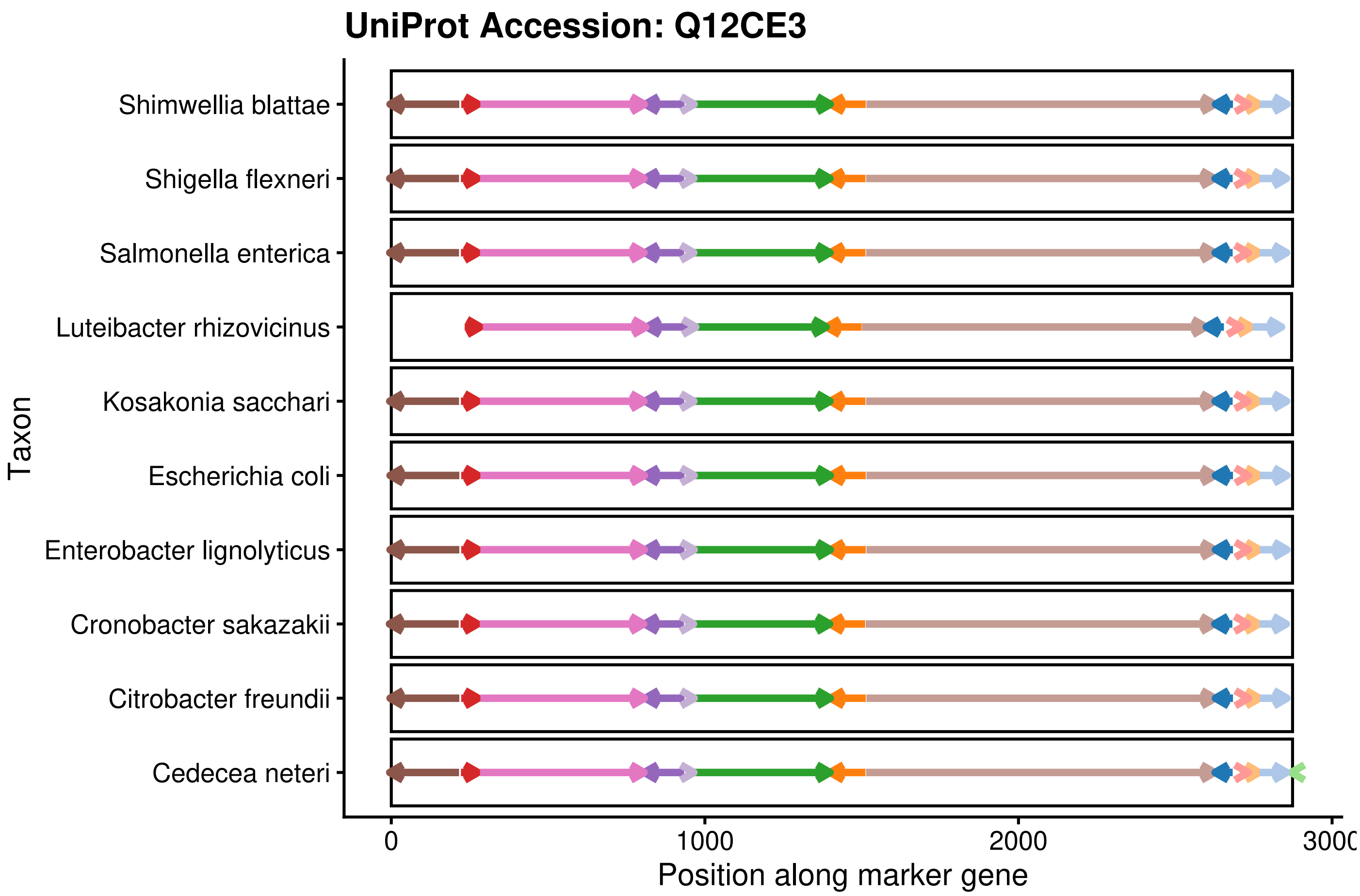
UniProt Accession: Q12CE3
Shimwellia blattae
Shigella flexneri
Salmonella enterica
Luteibacter rhizovicinus
Kosakonia sacchari
Escherichia coli
Enterobacter lignolyticus
Cronobacter sakazakii
Citrobacter freundii
Cedecea neteri
Taxon
0
1000
2000
3000
Position along marker gene

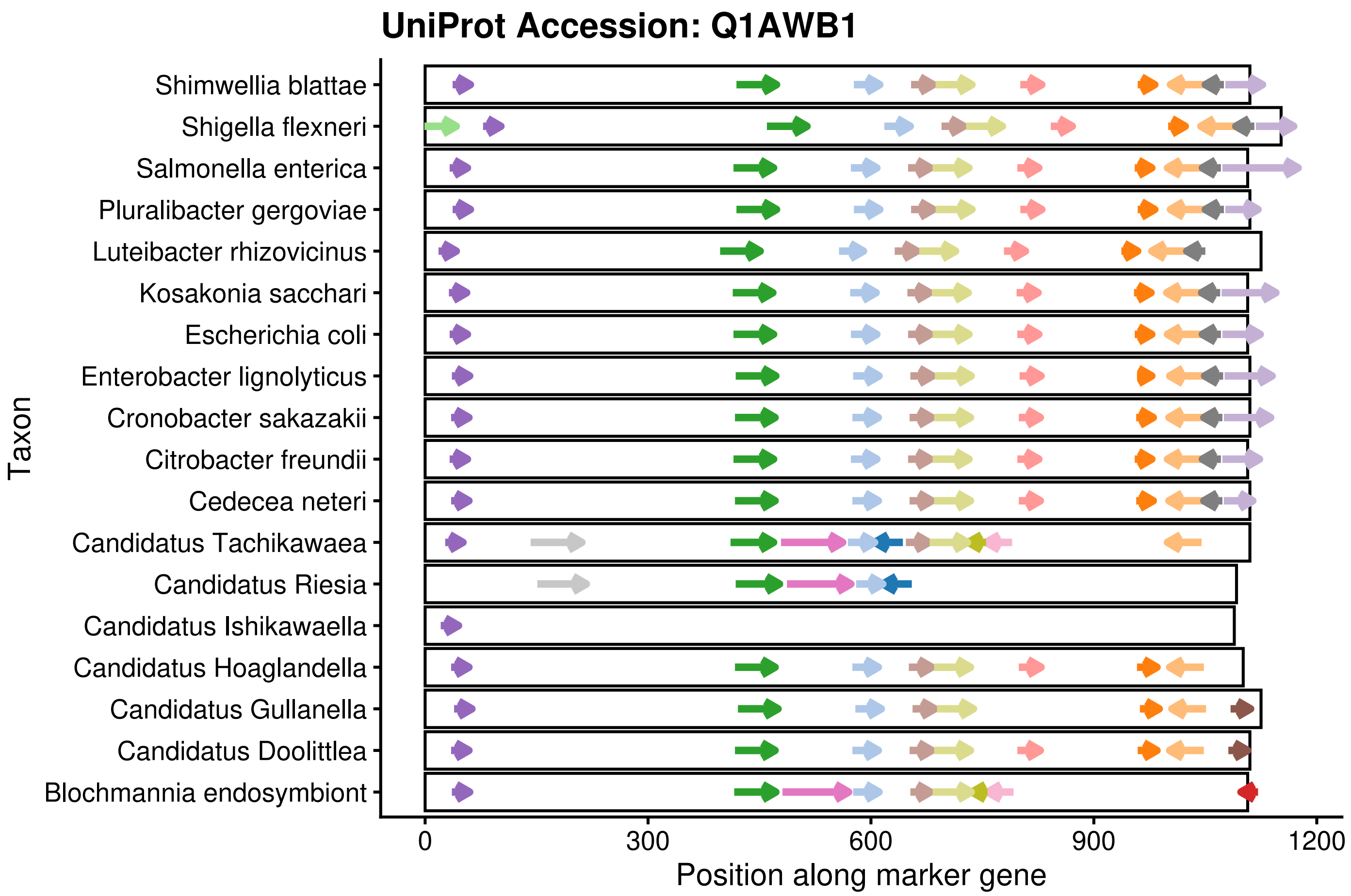

UniProt Accession: Q1AWB1
Taxon
Shimwellia blattae
Shigella flexneri
Salmonella enterica
Pluralibacter gergoviae
Luteibacter rhizovicinus
Kosakonia sacchari
Escherichia coli
Enterobacter lignolyticus
Cronobacter sakazakii
Citrobacter freundii
Cedecea neteri
Candidatus Tachikawaea
Candidatus Riesia
Candidatus Ishikawaella
Candidatus Hoaglandella
Candidatus Gullanella
Candidatus Doolittlea
Blochmannia endosymbiont
0
300
600
900
1200
Position along marker gene

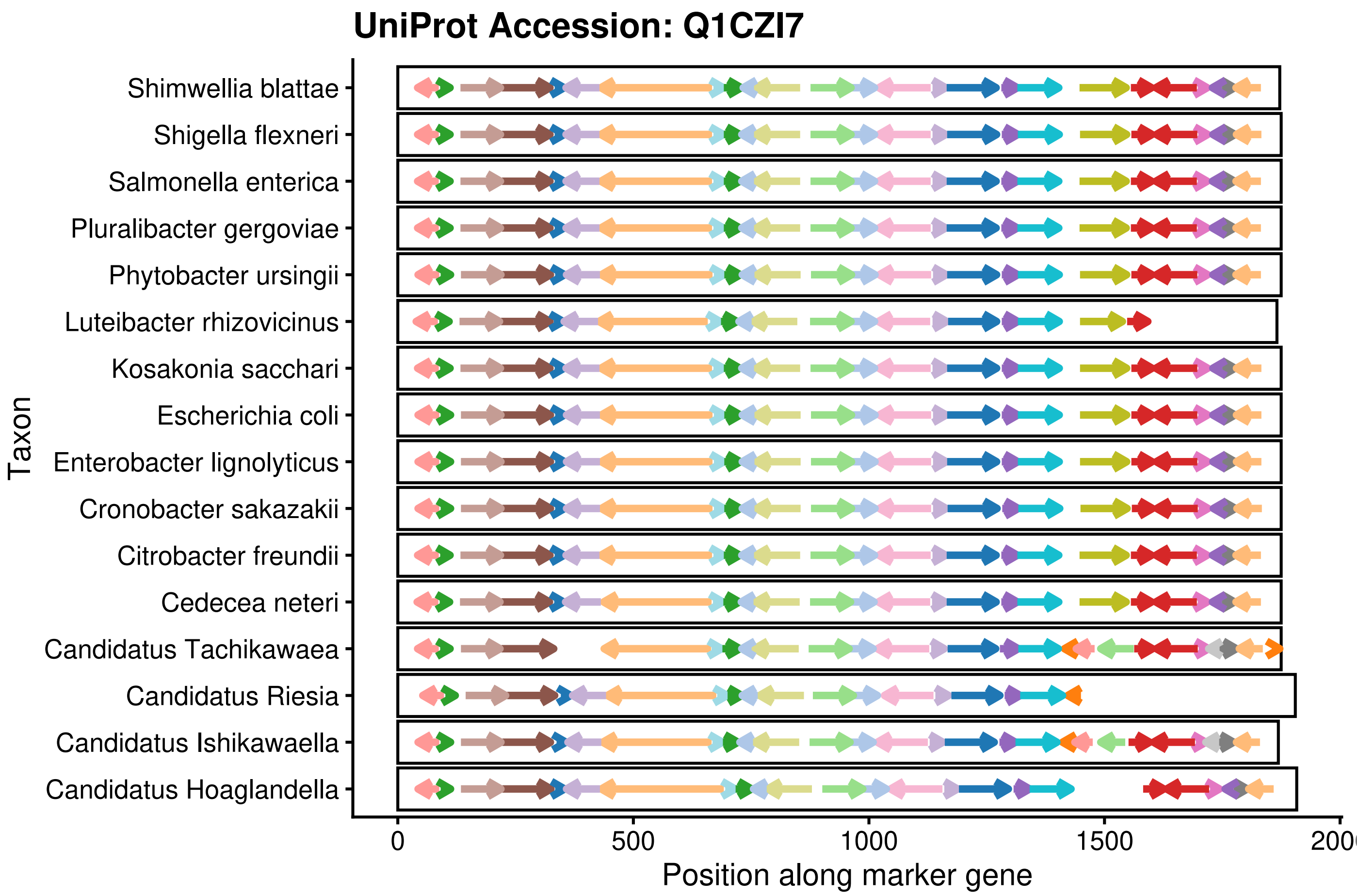

UniProt Accession: Q1CZI7
Taxon
Shimwellia blattae
Shigella flexneri
Salmonella enterica
Pluralibacter gergoviae
Phytobacter ursingii
Luteibacter rhizovicinus
Kosakonia sacchari
Escherichia coli
Enterobacter lignolyticus
Cronobacter sakazakii
Citrobacter freundii
Cedecea neteri
Candidatus Tachikawaea
Candidatus Riesia
Candidatus Ishikawaella
Candidatus Hoaglandella
0
500
1000
1500
Position along marker gene

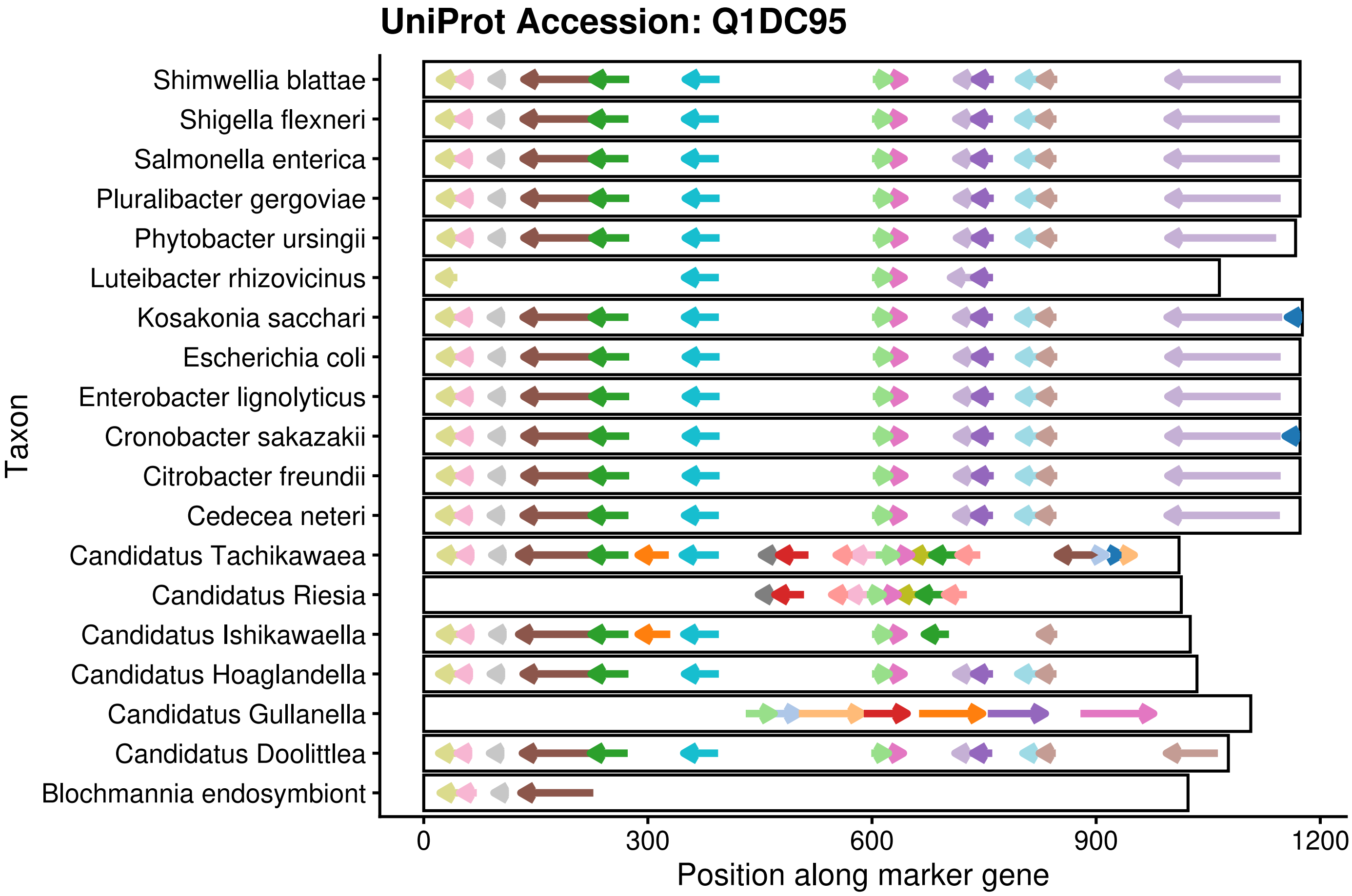
UniProt Accession: Q1DC95
Taxon
Shimwellia blattae
Shigella flexneri
Salmonella enterica
Pluralibacter gergoviae
Phytobacter ursingii
Luteibacter rhizovicinus
Kosakonia sacchari
Escherichia coli
Enterobacter lignolyticus
Cronobacter sakazakii
Citrobacter freundii
Cedecea neteri
Candidatus Tachikawaea
Candidatus Riesia
Candidatus Ishikawaella
Candidatus Hoaglandella
Candidatus Gullanella
Candidatus Doolittlea
Blochmannia endosymbiont
0
300
600
900
1200
Position along marker gene

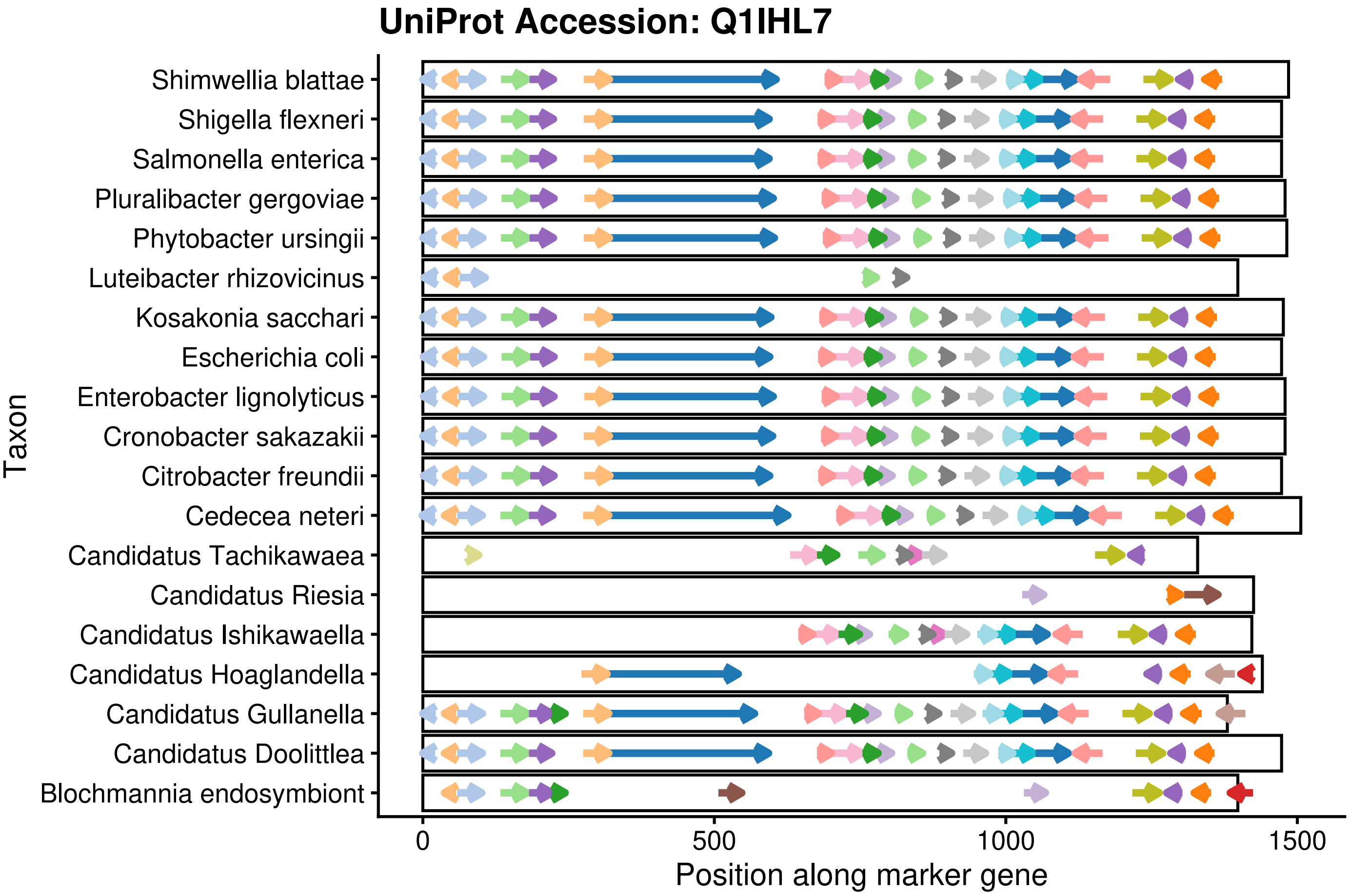
UniProt Accession: Q1IHL7
Shimwellia blattae
Shigella flexneri
Salmonella enterica
Pluralibacter gergoviae
Phytobacter ursingii
Luteibacter rhizovicinus
Kosakonia sacchari
Escherichia coli
Enterobacter lignolyticus
Cronobacter sakazakii
Citrobacter freundii
Cedecea neteri
Candidatus Tachikawaea
Candidatus Riesia
Candidatus Ishikawaella
Candidatus Hoaglandella
Candidatus Gullanella
Candidatus Doolittlea
Blochmannia endosymbiont
Taxon
0
500
1000
1500
Position along marker gene

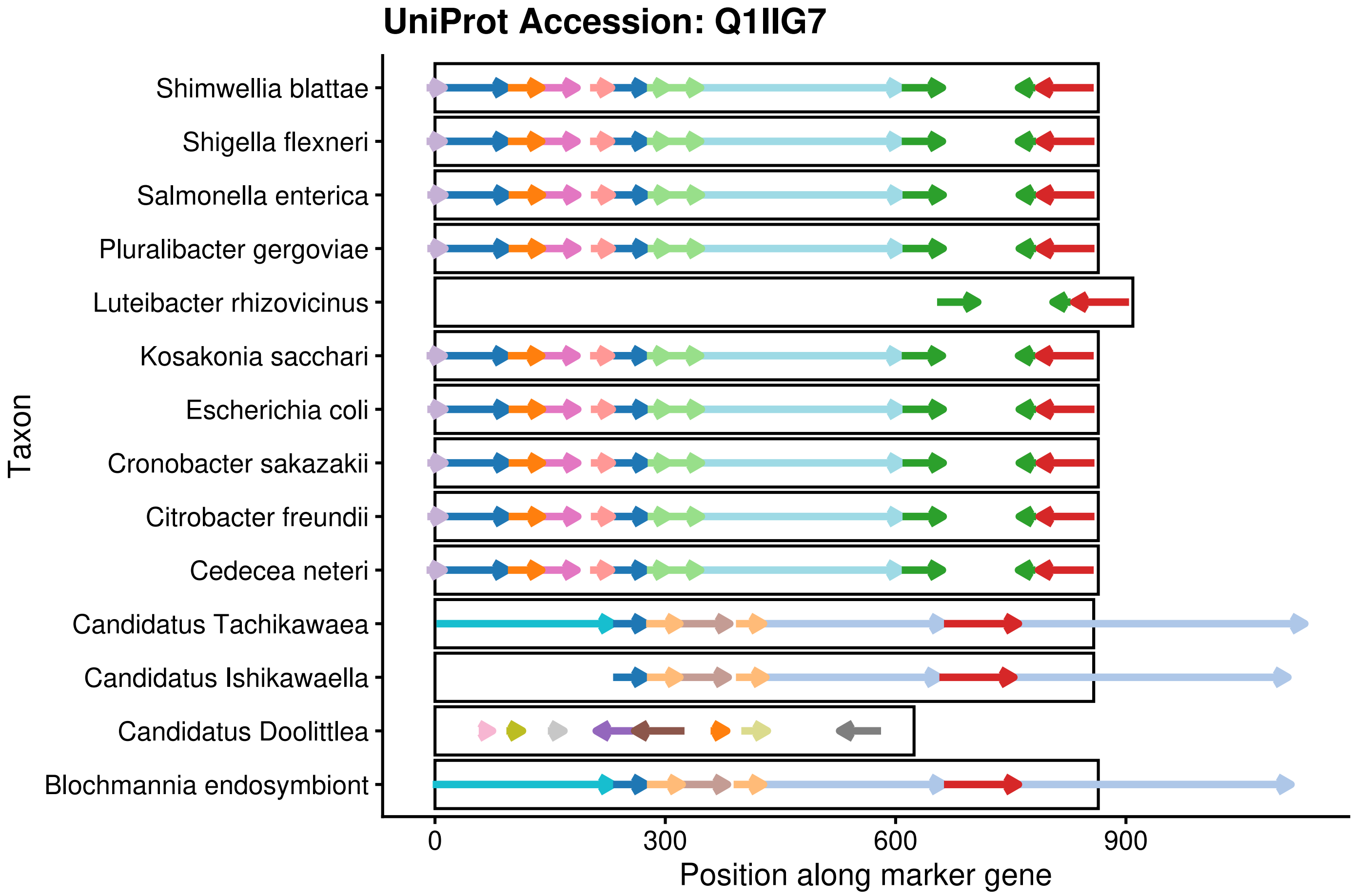
UniProt Accession: Q1IIG7
Shimwellia blattae
Shigella flexneri
Salmonella enterica
Pluralibacter gergoviae
Luteibacter rhizovicinus
Kosakonia sacchari
Escherichia coli
Cronobacter sakazakii
Citrobacter freundii
Cedecea neteri
Candidatus Tachikawaea
Candidatus Ishikawaella
Candidatus Doolittlea
Blochmannia endosymbiont
Taxon
0
300
600
900
Position along marker gene

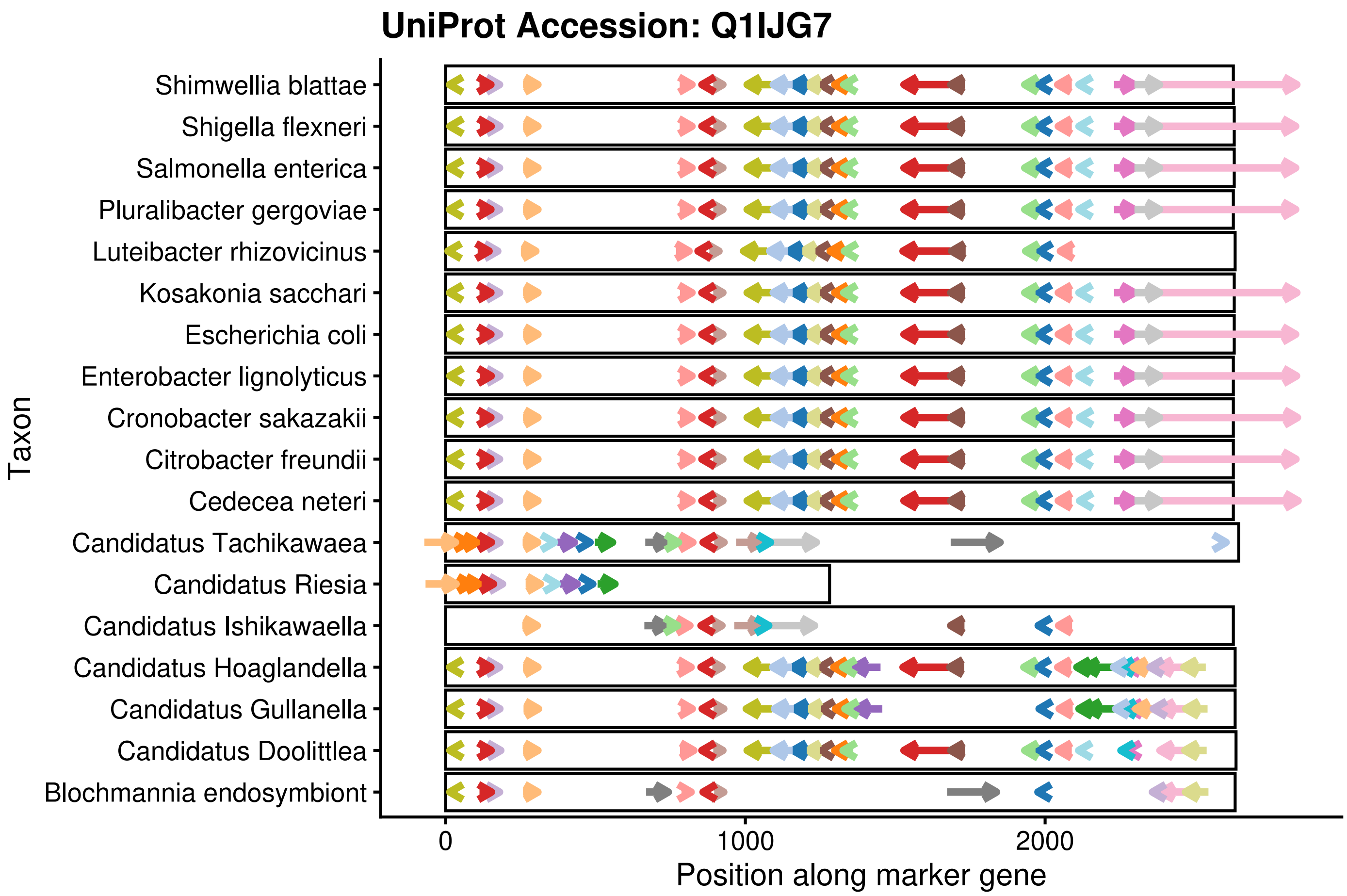
UniProt Accession: Q1IJG7
Shimwellia blattae
Shigella flexneri
Salmonella enterica
Pluralibacter gergoviae
Luteibacter rhizovicinus
Kosakonia sacchari
Escherichia coli
Enterobacter lignolyticus
Cronobacter sakazakii
Citrobacter freundii
Cedecea neteri
Candidatus Tachikawaea
Candidatus Riesia
Candidatus Ishikawaella
Candidatus Hoaglandella
Candidatus Gullanella
Candidatus Doolittlea
Blochmannia endosymbiont
Taxon
0
1000
2000
Position along marker gene

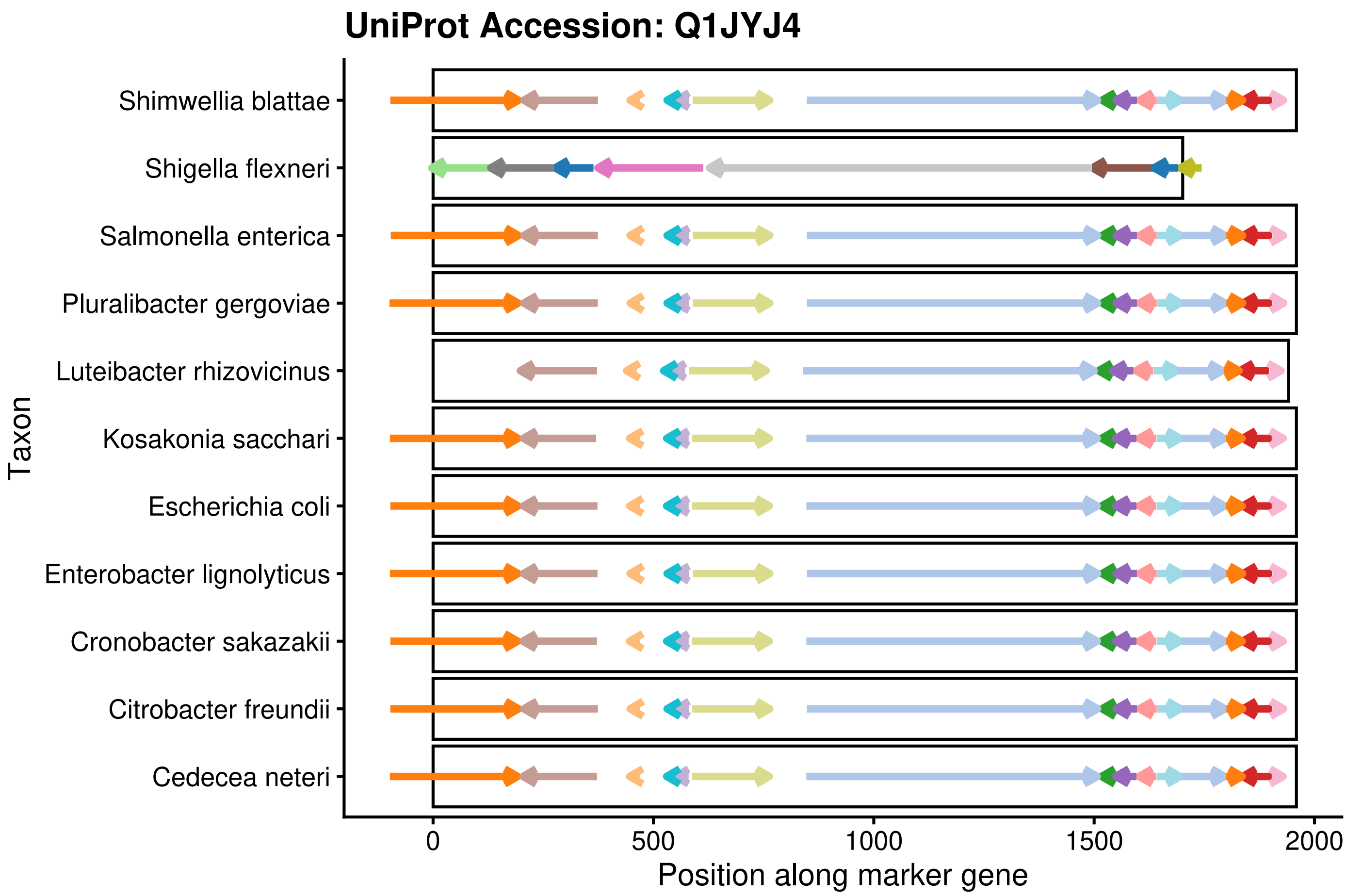

UniProt Accession: Q1JYJ4
Taxon
Shimwellia blattae
Shigella flexneri
Salmonella enterica
Pluralibacter gergoviae
Luteibacter rhizovicinus
Kosakonia sacchari
Escherichia coli
Enterobacter lignolyticus
Cronobacter sakazakii
Citrobacter freundii
Cedecea neteri
0
500
1000
1500
2000
Position along marker gene

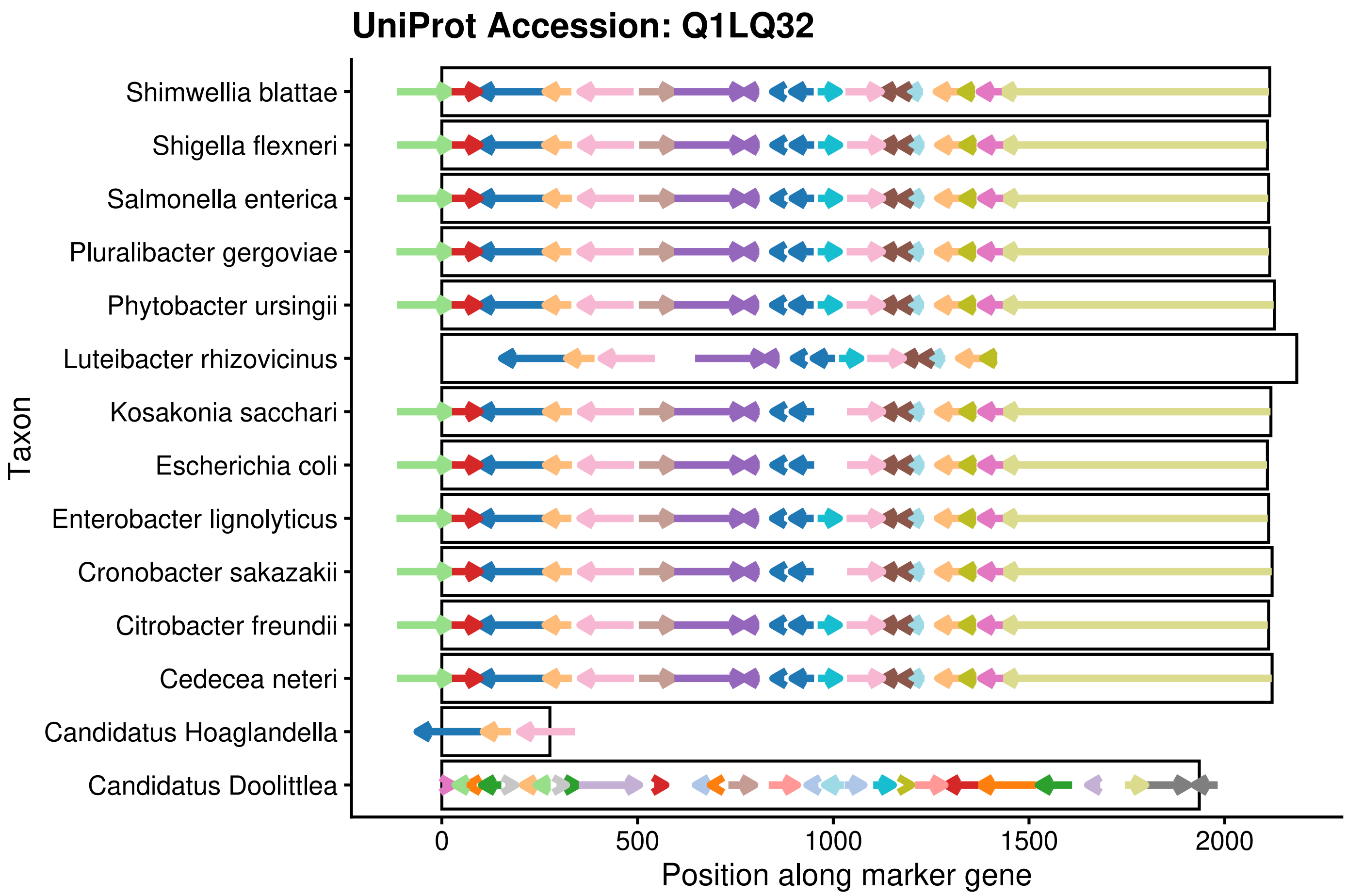
UniProt Accession: Q1LQ32
Shimwellia blattae
Shigella flexneri
Salmonella enterica
Pluralibacter gergoviae
Phytobacter ursingii
Luteibacter rhizovicinus
Kosakonia sacchari
Escherichia coli
Enterobacter lignolyticus
Cronobacter sakazakii
Citrobacter freundii
Cedecea neteri
Candidatus Hoaglandella
Candidatus Doolittlea
Taxon
0
500
1000
1500
2000
Position along marker gene

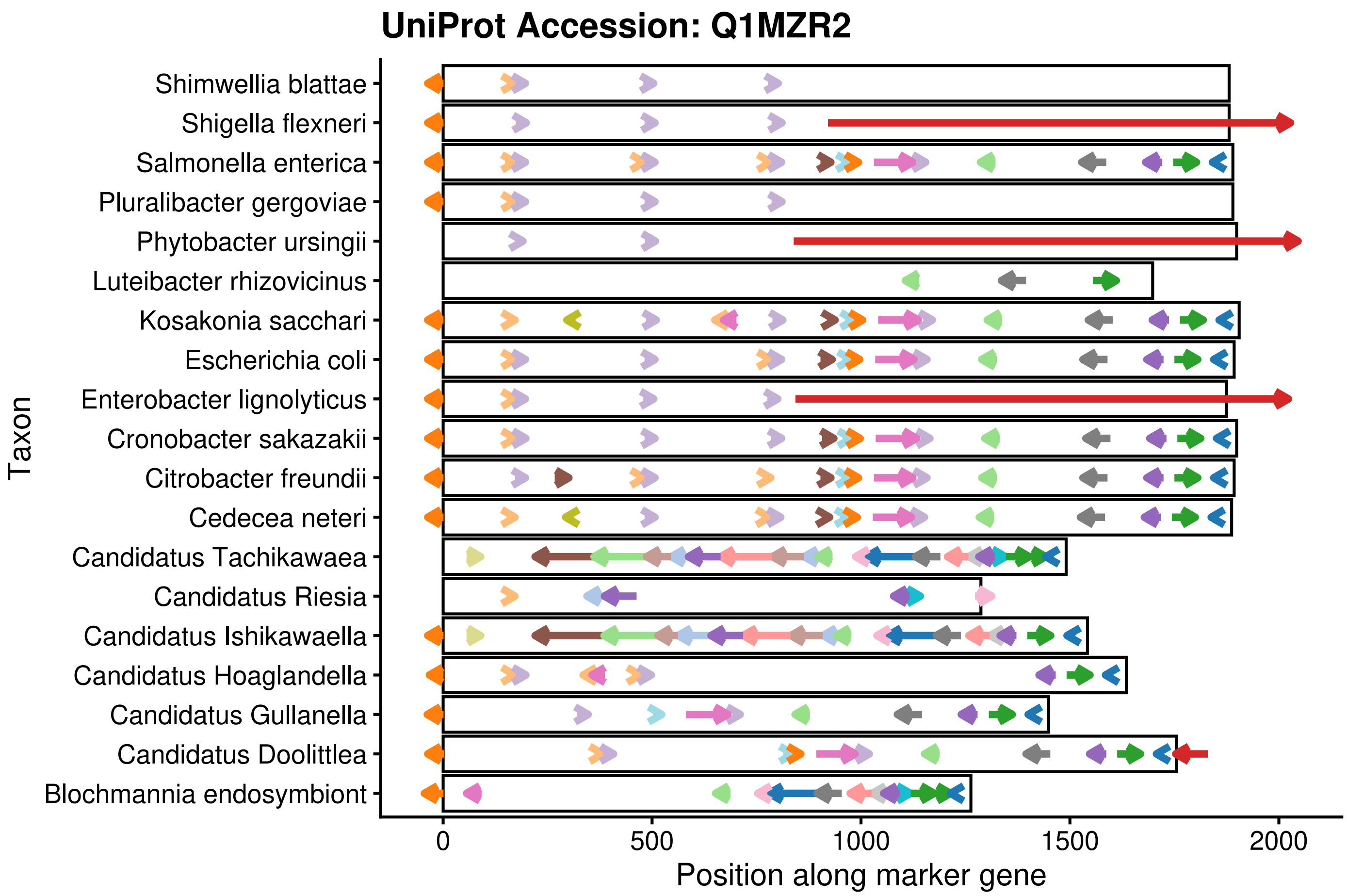
UniProt Accession: Q1MZR2
Taxon
Shimwellia blattae
Shigella flexneri
Salmonella enterica
Pluralibacter gergoviae
Phytobacter ursingii
Luteibacter rhizovicinus
Kosakonia sacchari
Escherichia coli
Enterobacter lignolyticus
Cronobacter sakazakii
Citrobacter freundii
Cedecea neteri
Candidatus Tachikawaea
Candidatus Riesia
Candidatus Ishikawaella
Candidatus Hoaglandella
Candidatus Gullanella
Candidatus Doolittlea
Blochmannia endosymbiont
0
500
1000
1500
2000
Position along marker gene

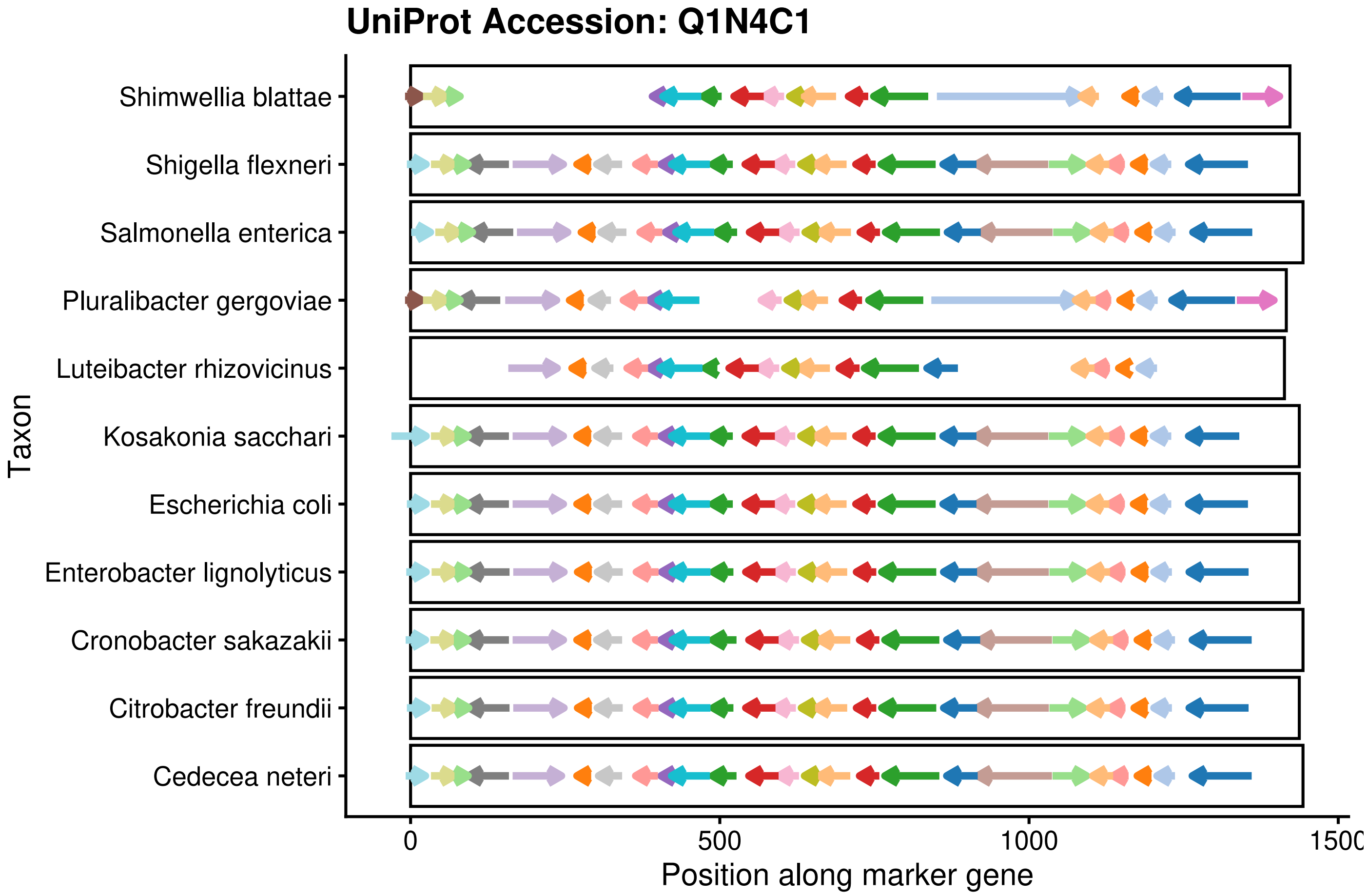

UniProt Accession: Q1N4C1
Taxon
Shimwellia blattae
Shigella flexneri
Salmonella enterica
Pluralibacter gergoviae
Luteibacter rhizovicinus
Kosakonia sacchari
Escherichia coli
Enterobacter lignolyticus
Cronobacter sakazakii
Citrobacter freundii
Cedecea neteri
0
500
1000
1500
Position along marker gene

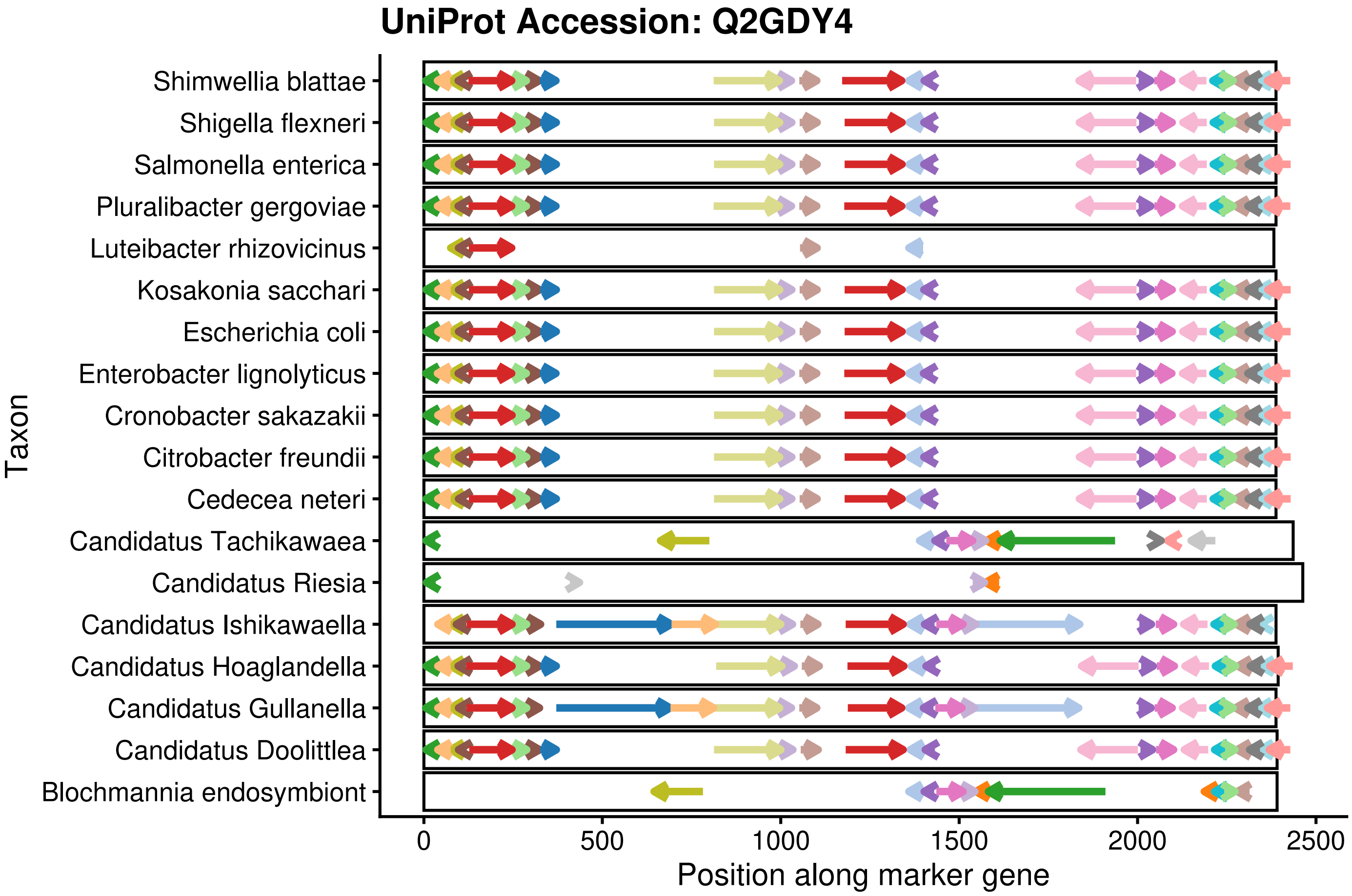
UniProt Accession: Q2GDY4
Taxon
Shimwellia blattae
Shigella flexneri
Salmonella enterica
Pluralibacter gergoviae
Luteibacter rhizovicinus
Kosakonia sacchari
Escherichia coli
Enterobacter lignolyticus
Cronobacter sakazakii
Citrobacter freundii
Cedecea neteri
Candidatus Tachikawaea
Candidatus Riesia
Candidatus Ishikawaella
Candidatus Hoaglandella
Candidatus Gullanella
Candidatus Doolittlea
Blochmannia endosymbiont
0
500
1000
1500
2000
2500
Position along marker gene

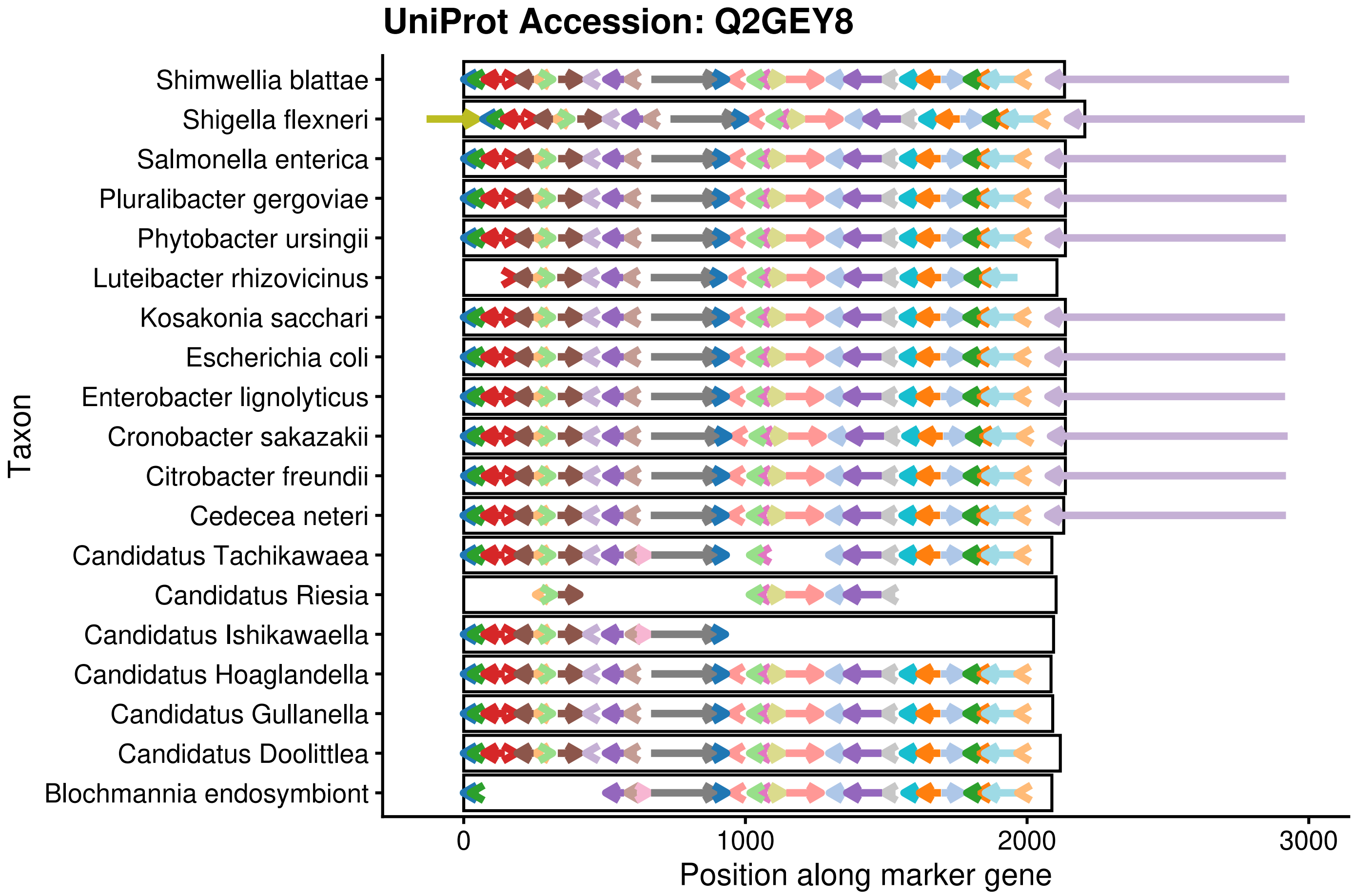
UniProt Accession: Q2GEY8
Taxon
Shimwellia blattae
Shigella flexneri
Salmonella enterica
Pluralibacter gergoviae
Phytobacter ursingii
Luteibacter rhizovicinus
Kosakonia sacchari
Escherichia coli
Enterobacter lignolyticus
Cronobacter sakazakii
Citrobacter freundii
Cedecea neteri
Candidatus Tachikawaea
Candidatus Riesia
Candidatus Ishikawaella
Candidatus Hoaglandella
Candidatus Gullanella
Candidatus Doolittlea
Blochmannia endosymbiont
0
1000
2000
3000
Position along marker gene

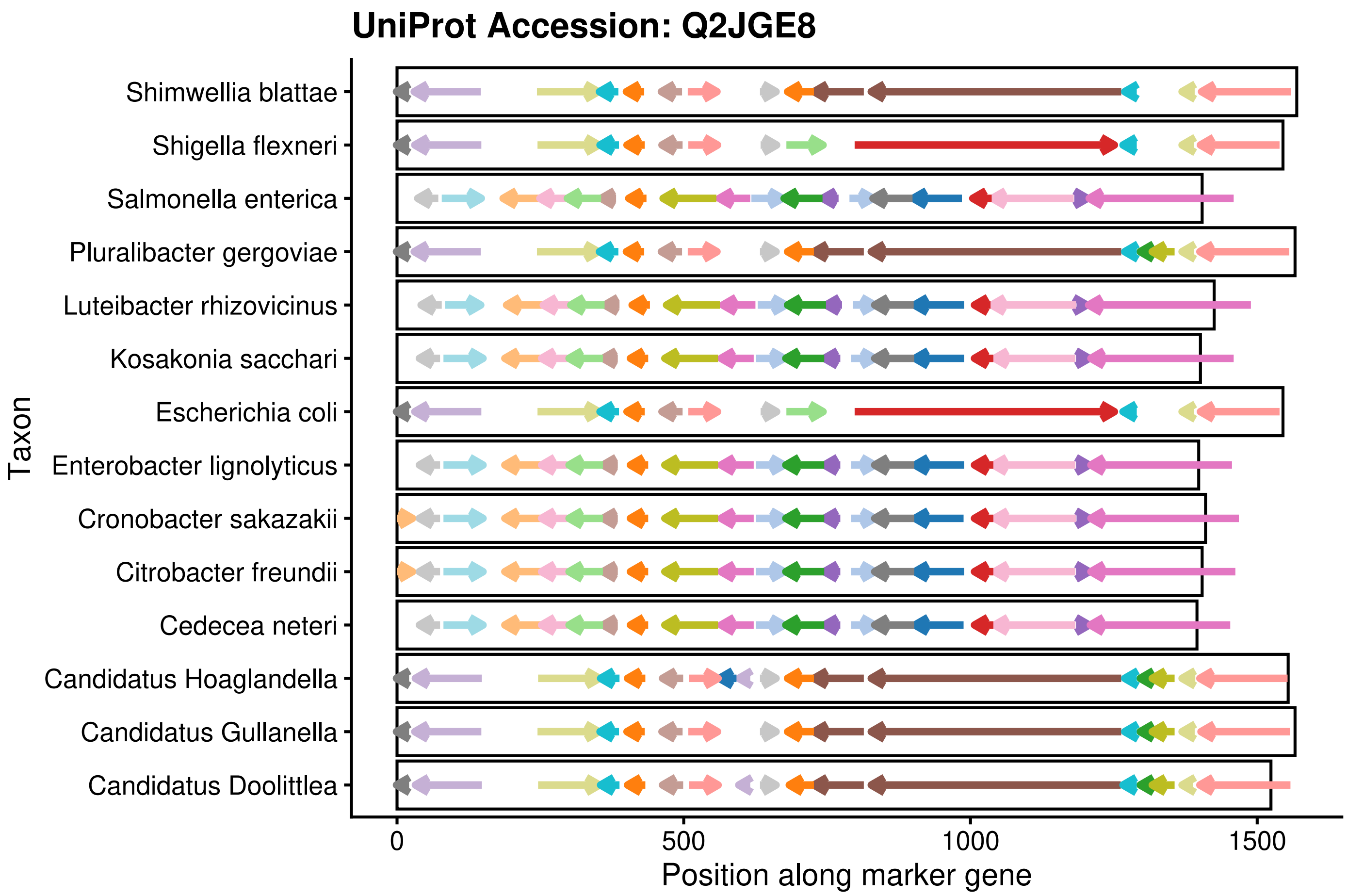

UniProt Accession: Q2JGE8
Shimwellia blattae
Shigella flexneri
Salmonella enterica
Pluralibacter gergoviae
Luteibacter rhizovicinus
Kosakonia sacchari
Escherichia coli
Enterobacter lignolyticus
Cronobacter sakazakii
Citrobacter freundii
Cedecea neteri
Candidatus Hoaglandella
Candidatus Gullanella
Candidatus Doolittlea
Taxon
0
500
1000
1500
Position along marker gene

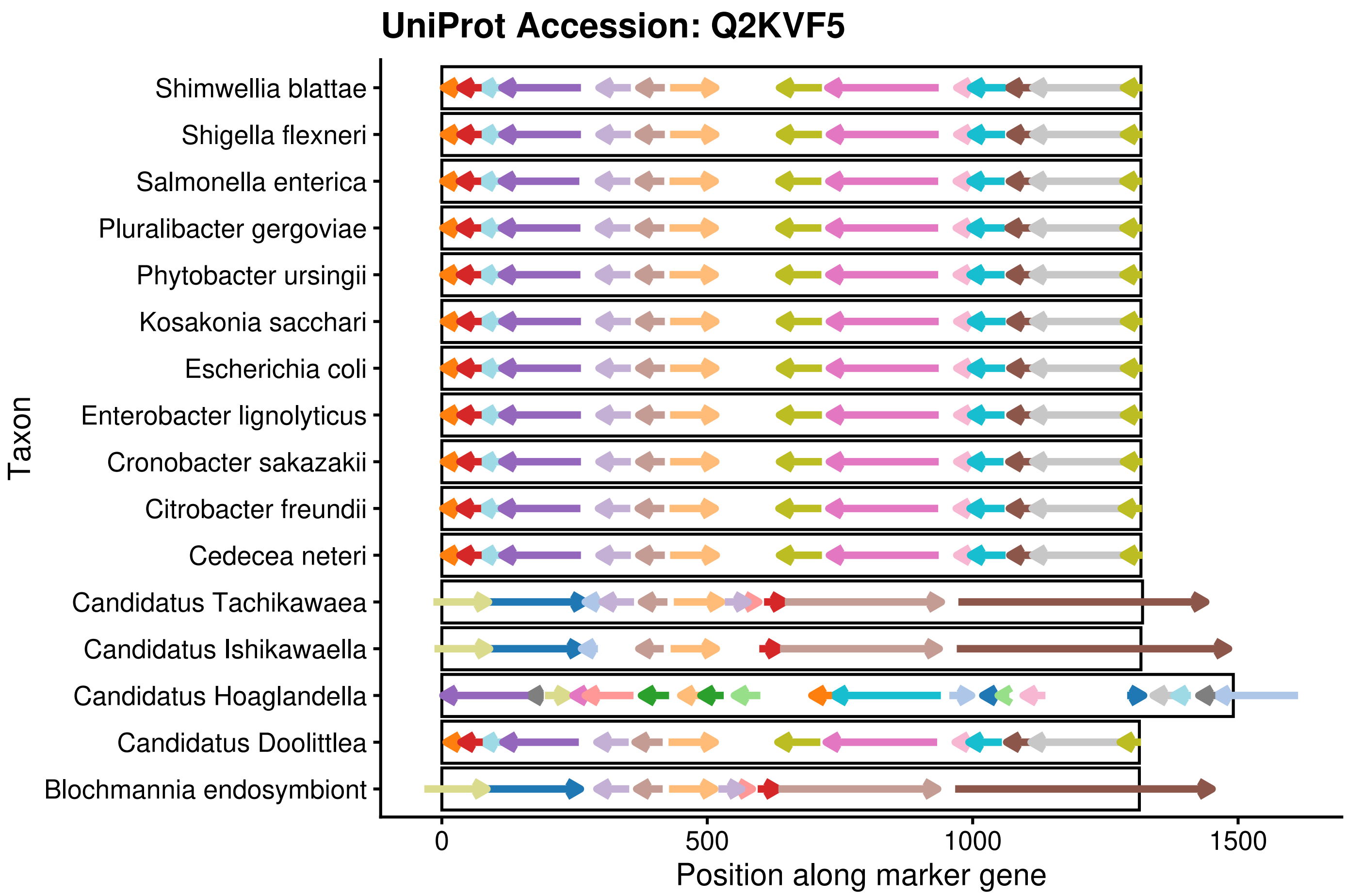
UniProt Accession: Q2KVF5
Taxon
Shimwellia blattae
Shigella flexneri
Salmonella enterica
Pluralibacter gergoviae
Phytobacter ursingii
Kosakonia sacchari
Escherichia coli
Enterobacter lignolyticus
Cronobacter sakazakii
Citrobacter freundii
Cedecea neteri
Candidatus Tachikawaea
Candidatus Ishikawaella
Candidatus Hoaglandella
Candidatus Doolittlea
Blochmannia endosymbiont
0
500
1000
1500
Position along marker gene

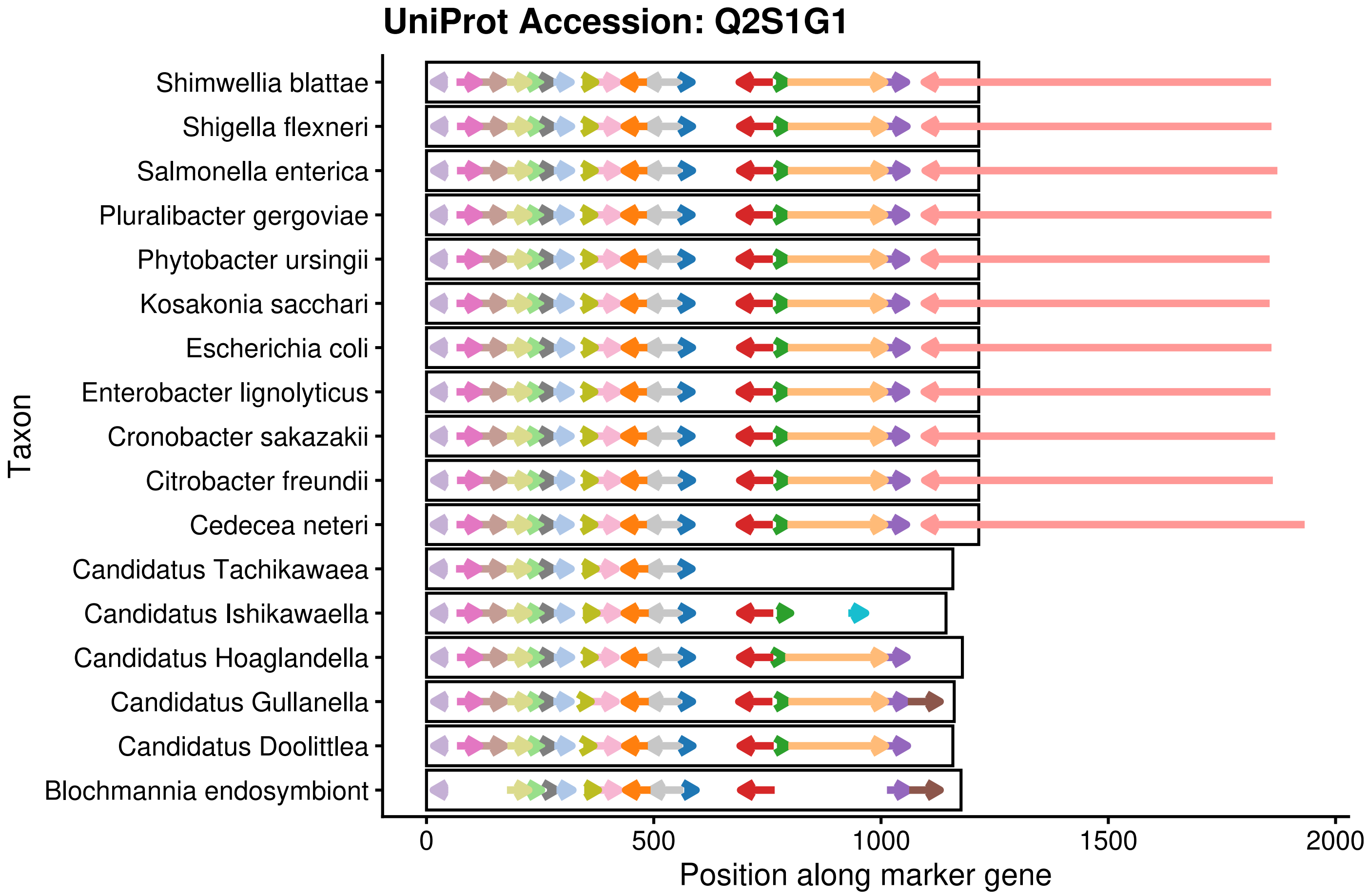
UniProt Accession: Q2S1G1
Taxon
Shimwellia blattae
Shigella flexneri
Salmonella enterica
Pluralibacter gergoviae
Phytobacter ursingii
Kosakonia sacchari
Escherichia coli
Enterobacter lignolyticus
Cronobacter sakazakii
Citrobacter freundii
Cedecea neteri
Candidatus Tachikawaea
Candidatus Ishikawaella
Candidatus Hoaglandella
Candidatus Gullanella
Candidatus Doolittlea
Blochmannia endosymbiont
0
500
1000
1500
2000
Position along marker gene

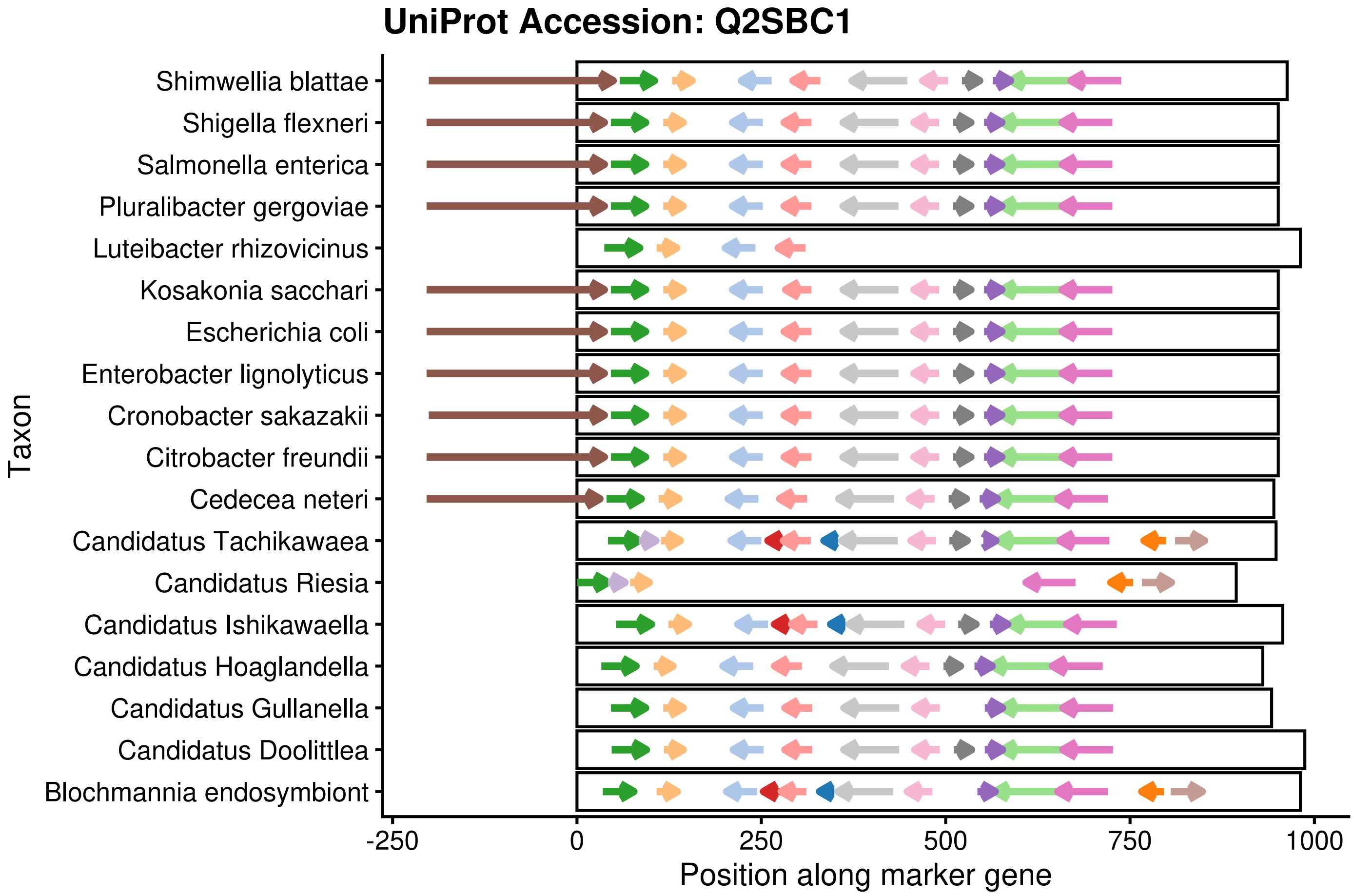

UniProt Accession: Q2SBC1
Shimwellia blattae
Shigella flexneri
Salmonella enterica
Pluralibacter gergoviae
Luteibacter rhizovicinus
Kosakonia sacchari
Escherichia coli
Enterobacter lignolyticus
Cronobacter sakazakii
Citrobacter freundii
Cedecea neteri
Candidatus Tachikawaea
Candidatus Riesia
Candidatus Ishikawaella
Candidatus Hoaglandella
Candidatus Gullanella
Candidatus Doolittlea
Blochmannia endosymbiont
Taxon
-250
0
250
500
750
1000
Position along marker gene

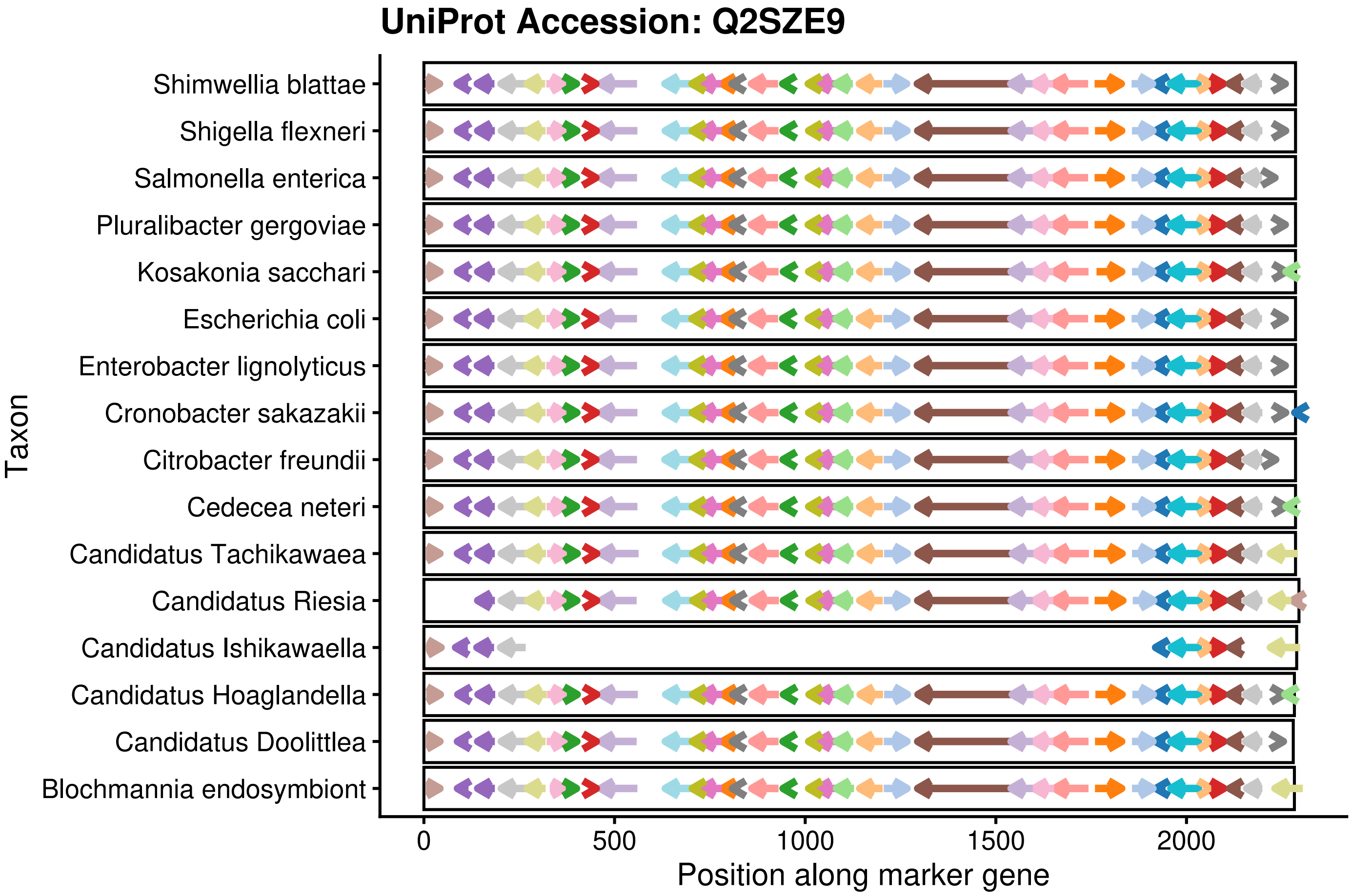

UniProt Accession: Q2SZE9
Taxon
Shimwellia blattae
Shigella flexneri
Salmonella enterica
Pluralibacter gergoviae
Kosakonia sacchari
Escherichia coli
Enterobacter lignolyticus
Cronobacter sakazakii
Citrobacter freundii
Cedecea neteri
Candidatus Tachikawaea
Candidatus Riesia
Candidatus Ishikawaella
Candidatus Hoaglandella
Candidatus Doolittlea
Blochmannia endosymbiont
0
500
1000
1500
2000
Position along marker gene

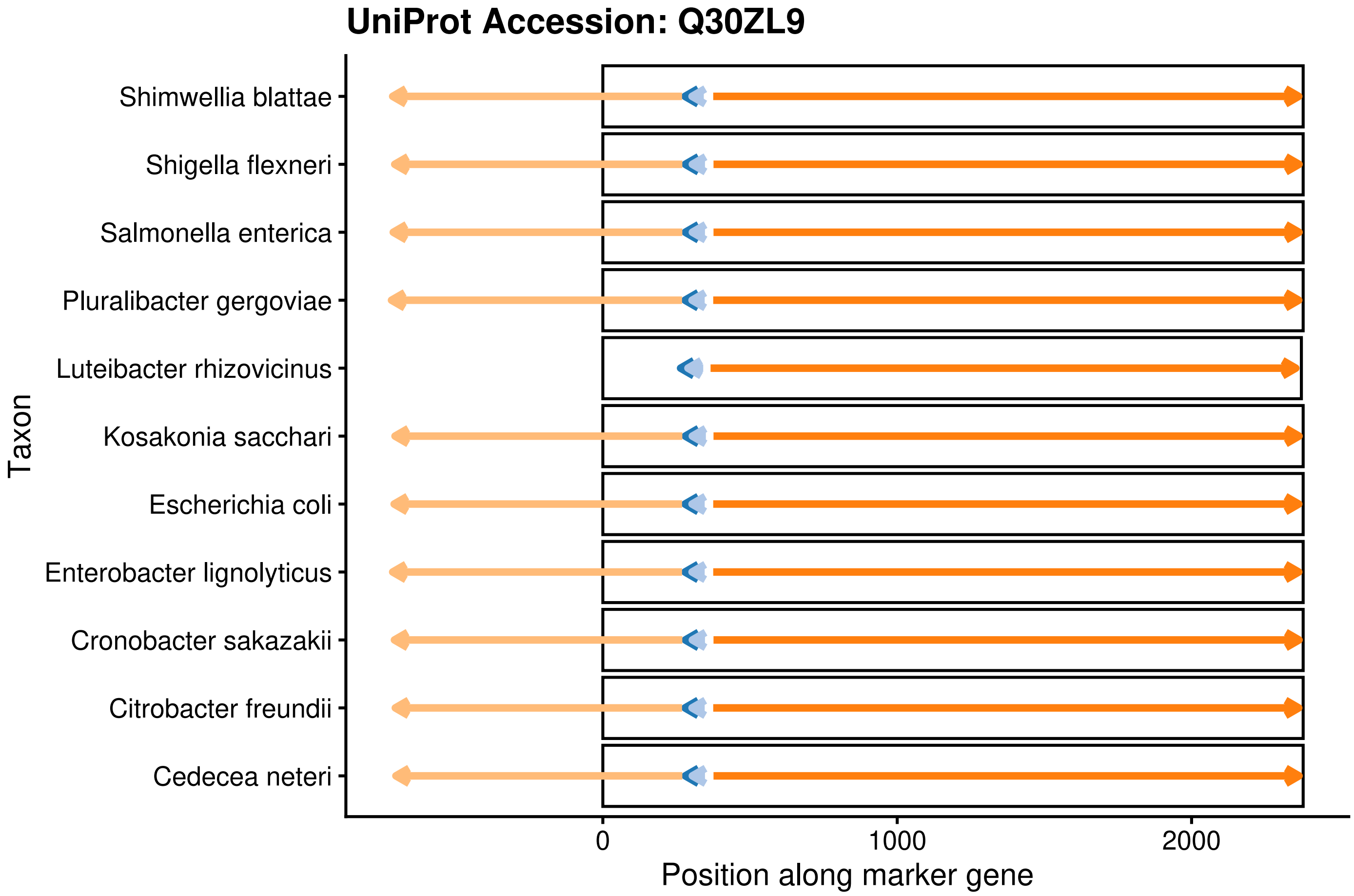

UniProt Accession: Q30ZL9
Shimwellia blattae
Shigella flexneri
Salmonella enterica
Pluralibacter gergoviae
Luteibacter rhizovicinus
Kosakonia sacchari
Escherichia coli
Enterobacter lignolyticus
Cronobacter sakazakii
Citrobacter freundii
Cedecea neteri
Taxon
0
1000
2000
Position along marker gene

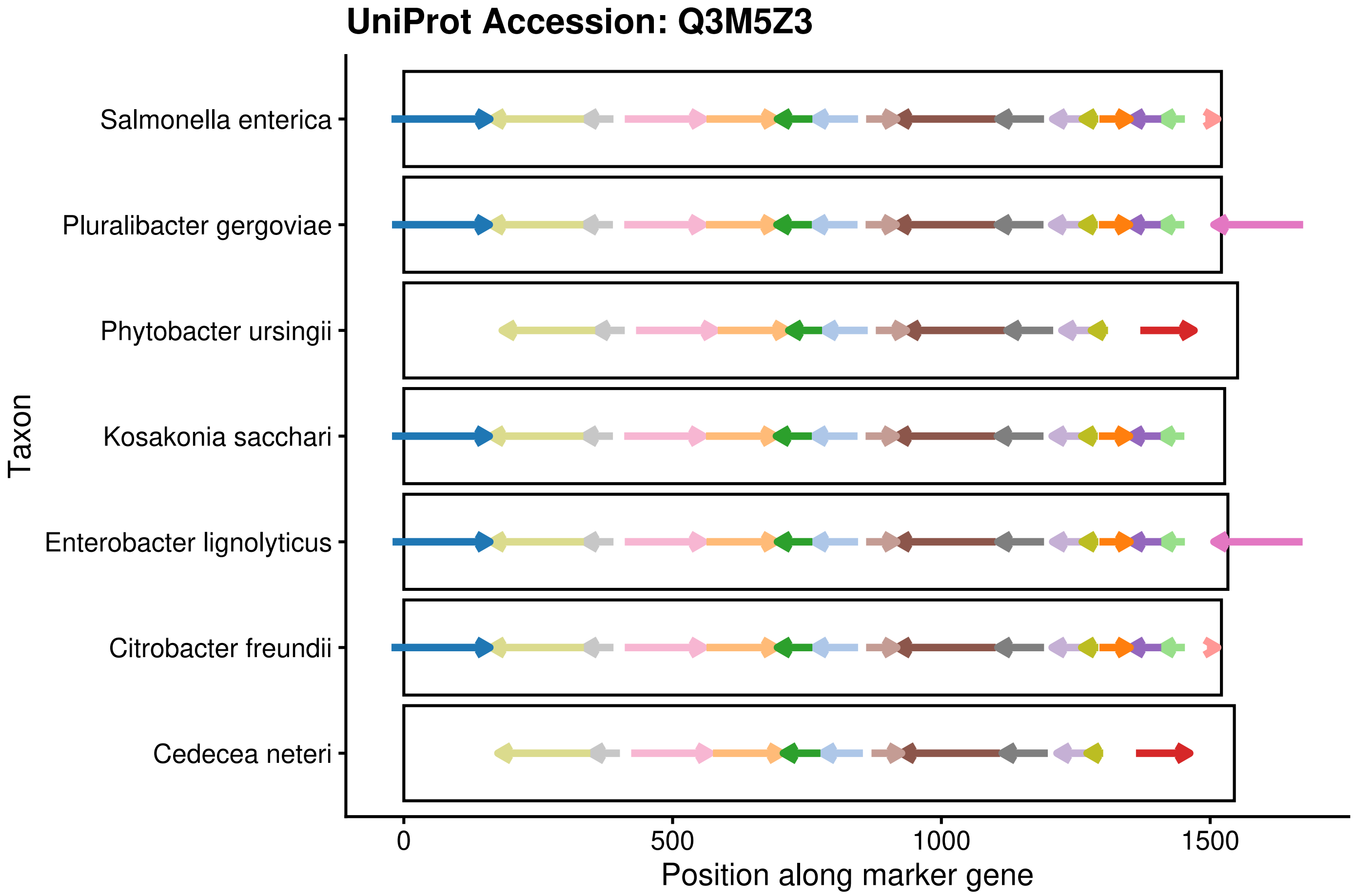
UniProt Accession: Q3M5Z3
Salmonella enterica
Pluralibacter gergoviae
Phytobacter ursingii
Kosakonia sacchari
Enterobacter lignolyticus
Citrobacter freundii
Cedecea neteri
Taxon
0
500
1000
1500
Position along marker gene

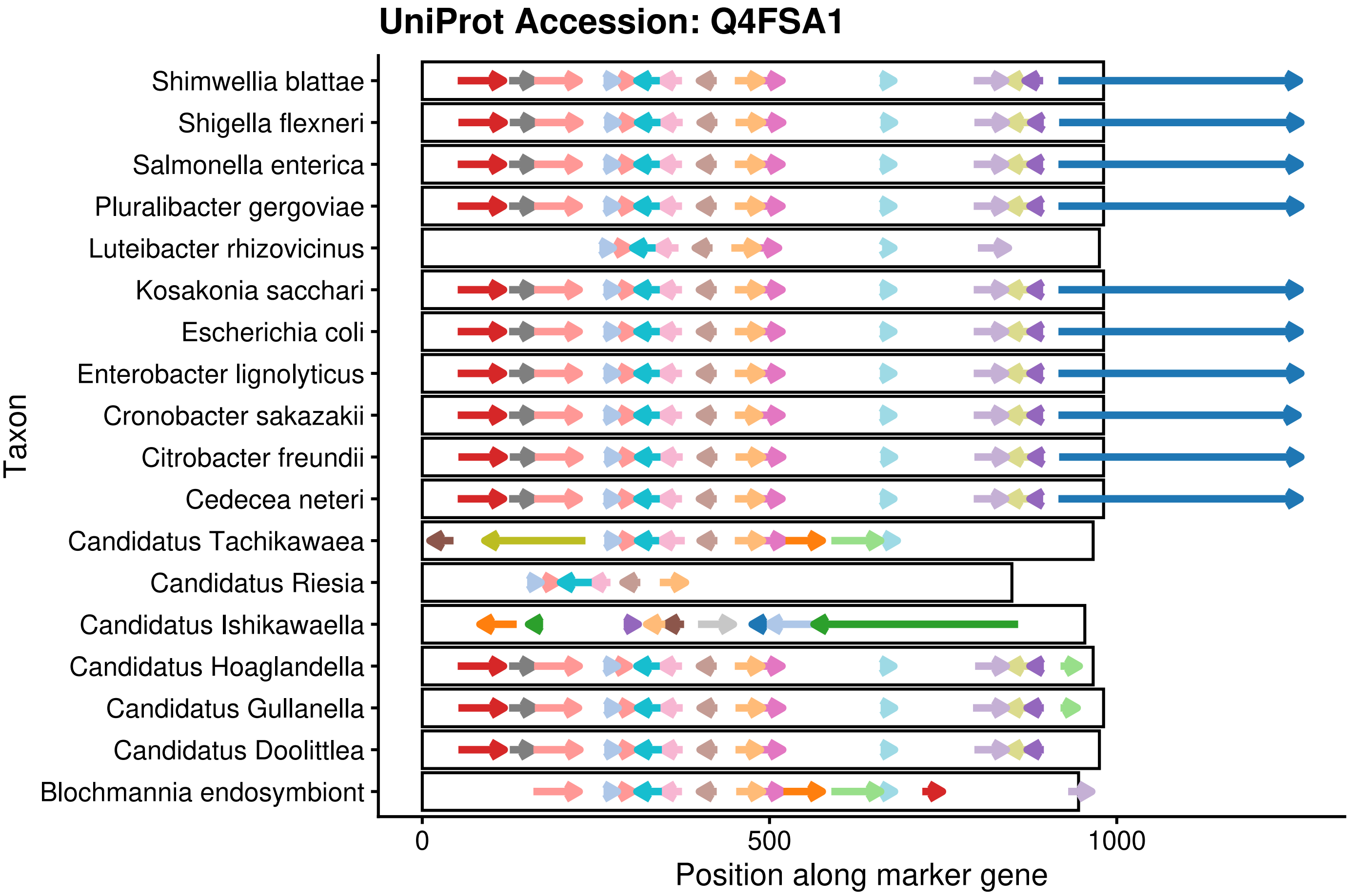

UniProt Accession: Q4FSA1
Taxon
Shimwellia blattae
Shigella flexneri
Salmonella enterica
Pluralibacter gergoviae
Luteibacter rhizovicinus
Kosakonia sacchari
Escherichia coli
Enterobacter lignolyticus
Cronobacter sakazakii
Citrobacter freundii
Cedecea neteri
Candidatus Tachikawaea
Candidatus Riesia
Candidatus Ishikawaella
Candidatus Hoaglandella
Candidatus Gullanella
Candidatus Doolittlea
Blochmannia endosymbiont
0
500
1000
Position along marker gene

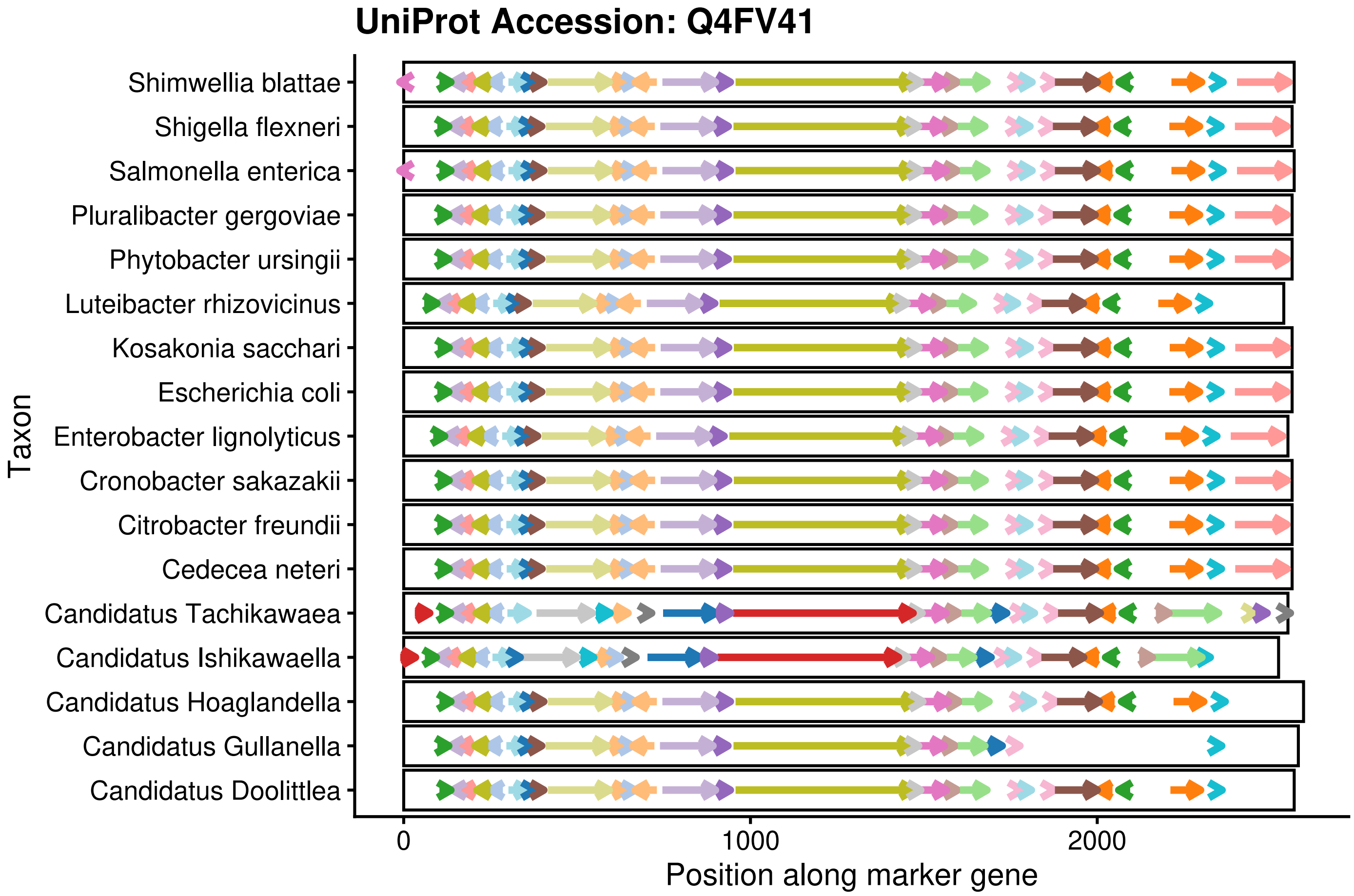
UniProt Accession: Q4FV41
Taxon
Shimwellia blattae
Shigella flexneri
Salmonella enterica
Pluralibacter gergoviae
Phytobacter ursingii
Luteibacter rhizovicinus
Kosakonia sacchari
Escherichia coli
Enterobacter lignolyticus
Cronobacter sakazakii
Citrobacter freundii
Cedecea neteri
Candidatus Tachikawaea
Candidatus Ishikawaella
Candidatus Hoaglandella
Candidatus Gullanella
Candidatus Doolittlea
0
1000
2000
Position along marker gene

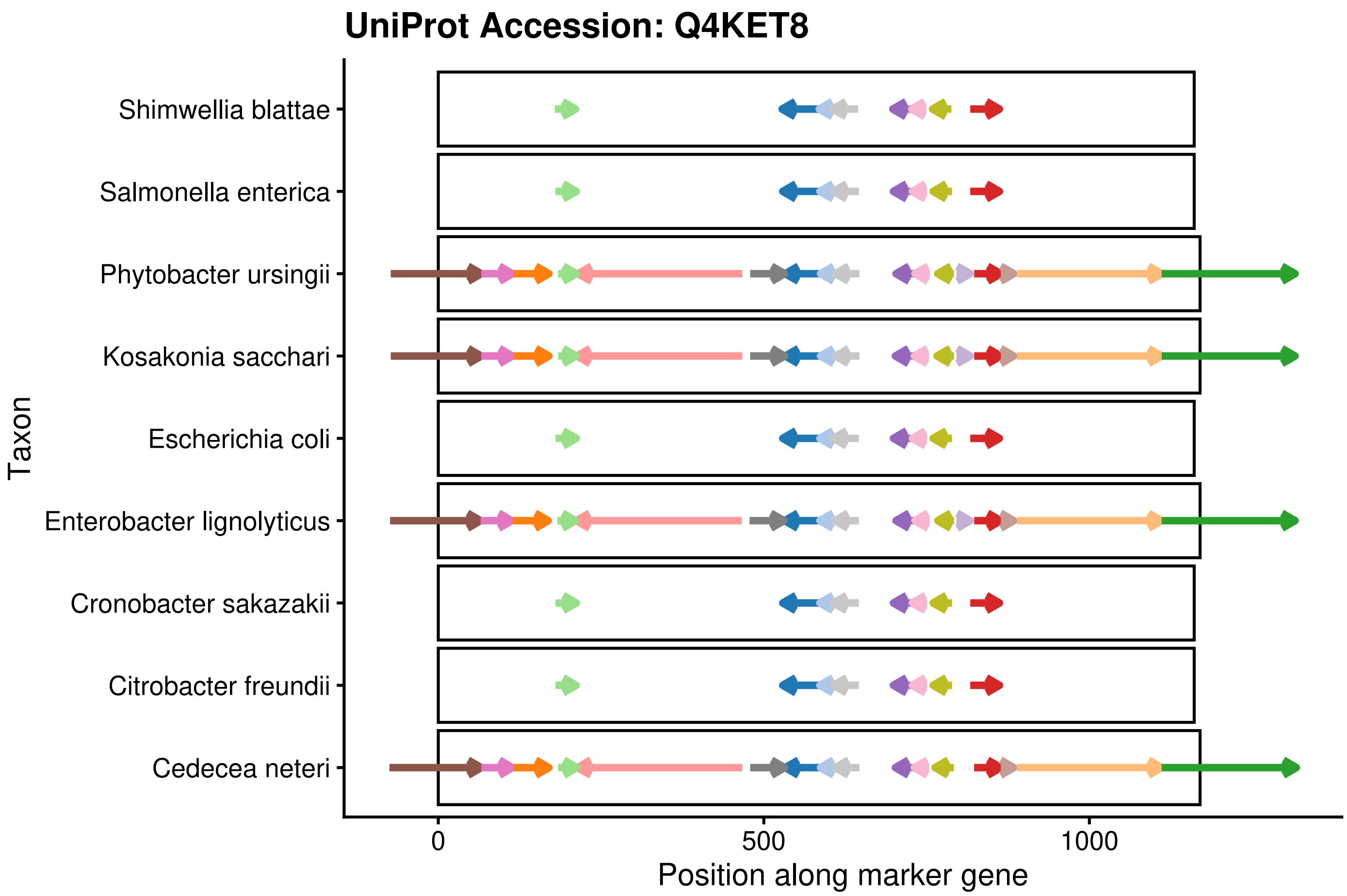

UniProt Accession: Q4KET8
Shimwellia blattae
Salmonella enterica
Phytobacter ursingii
Kosakonia sacchari
Escherichia coli
Enterobacter lignolyticus
Cronobacter sakazakii
Citrobacter freundii
Cedecea neteri
Taxon
0
500
1000
Position along marker gene

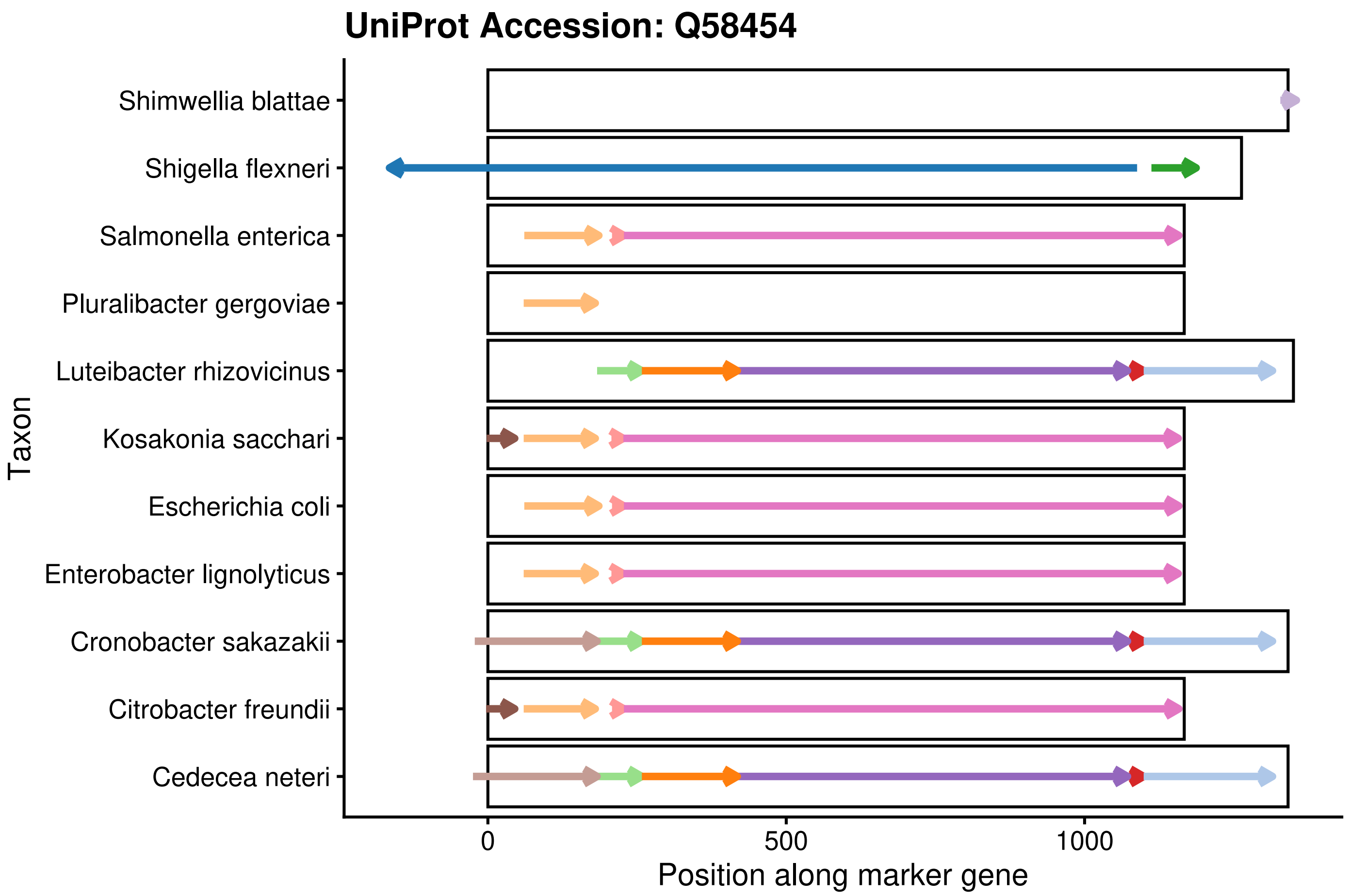
UniProt Accession: Q58454
Taxon
Shimwellia blattae
Shigella flexneri
Salmonella enterica
Pluralibacter gergoviae
Luteibacter rhizovicinus
Kosakonia sacchari
Escherichia coli
Enterobacter lignolyticus
Cronobacter sakazakii
Citrobacter freundii
Cedecea neteri
0
500
1000
Position along marker gene

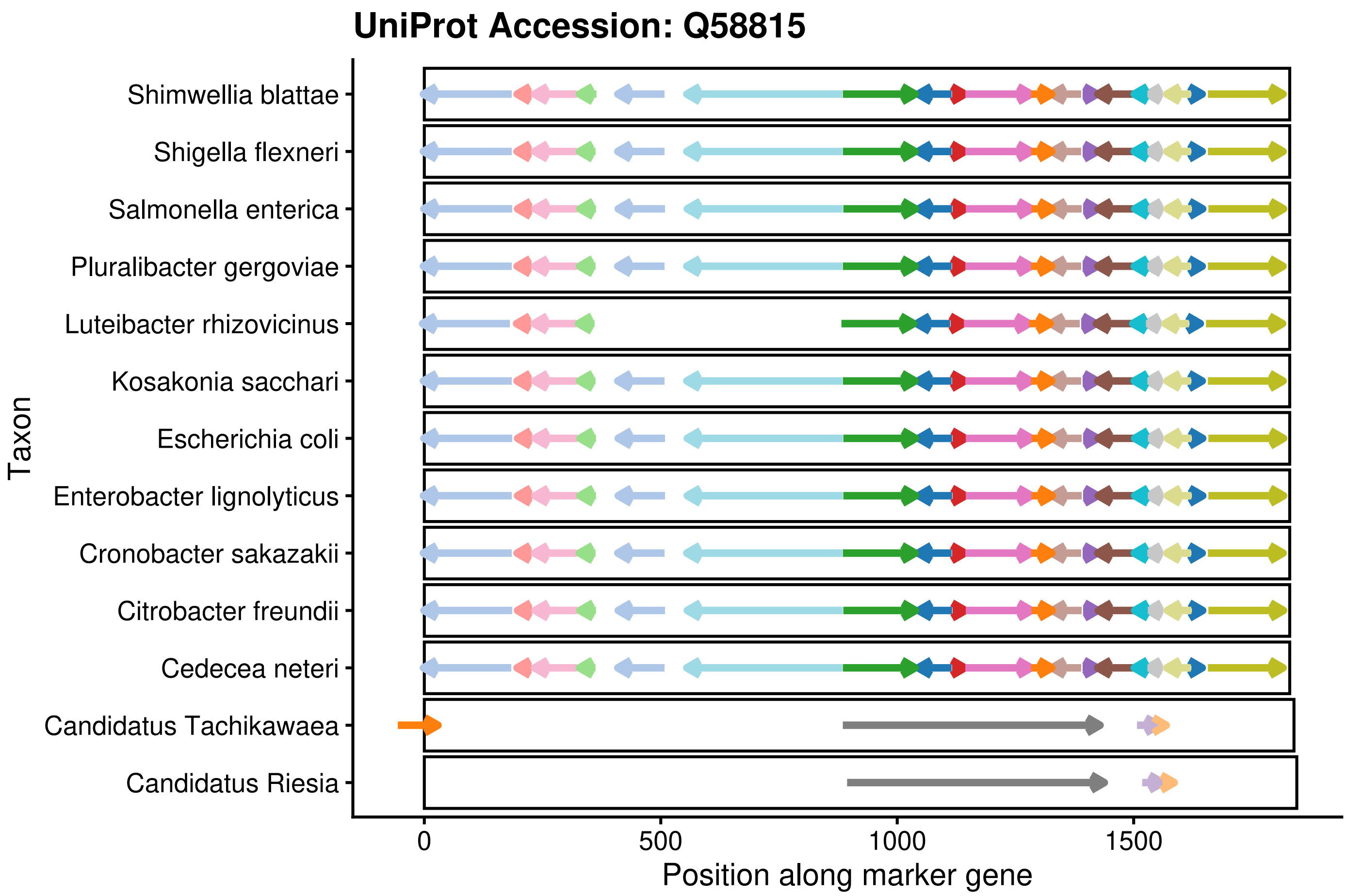
UniProt Accession: Q58815
Shimwellia blattae
Shigella flexneri
Salmonella enterica
Pluralibacter gergoviae
Luteibacter rhizovicinus
Kosakonia sacchari
Escherichia coli
Enterobacter lignolyticus
Cronobacter sakazakii
Citrobacter freundii
Cedecea neteri
Candidatus Tachikawaea
Candidatus Riesia
Taxon
0
500
1000
1500
Position along marker gene

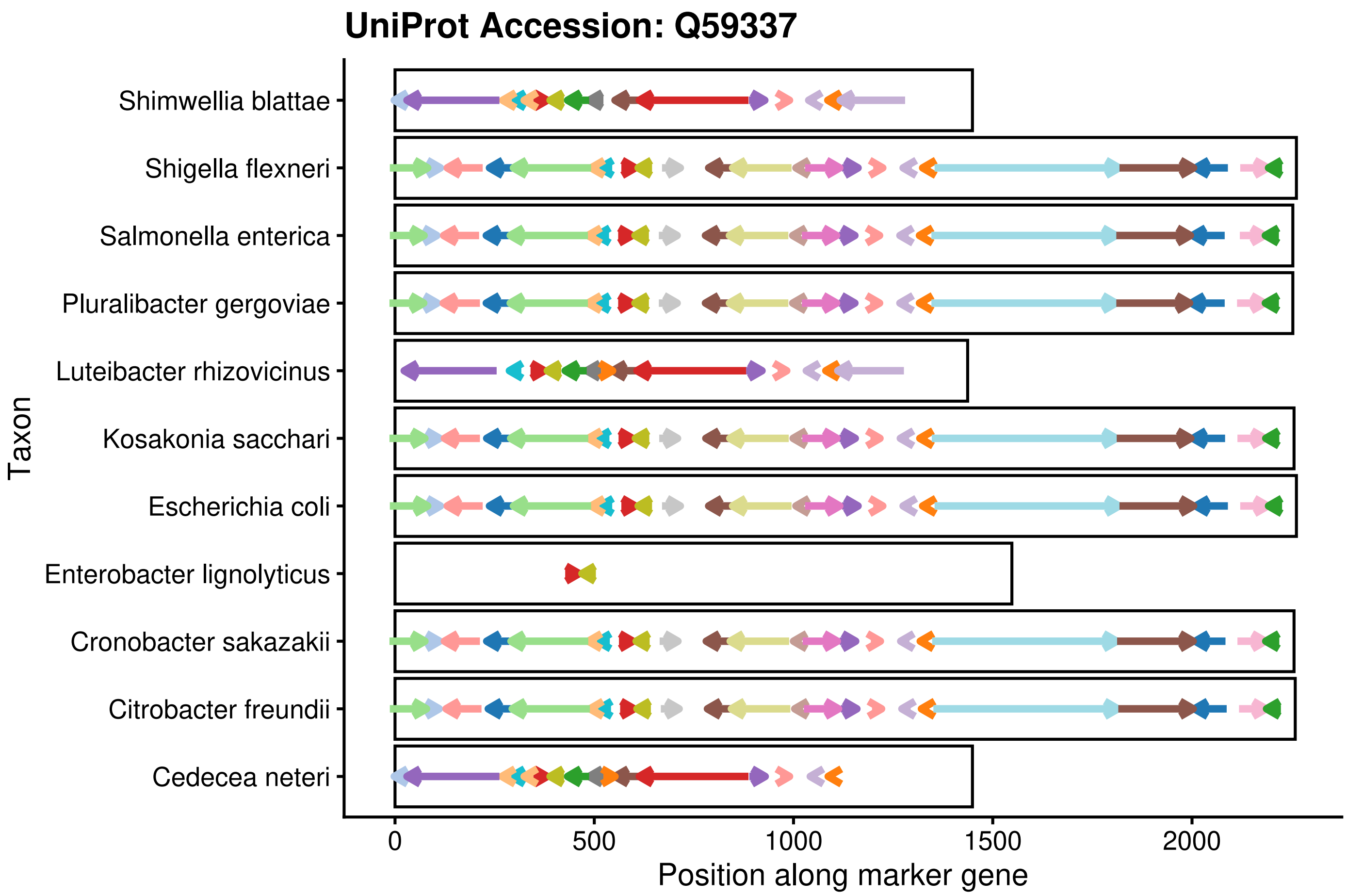
UniProt Accession: Q59337
Shimwellia blattae
Shigella flexneri
Salmonella enterica
Pluralibacter gergoviae
Luteibacter rhizovicinus
Kosakonia sacchari
Escherichia coli
Enterobacter lignolyticus
Cronobacter sakazakii
Citrobacter freundii
Cedecea neteri
Taxon
0
500
1000
1500
2000
Position along marker gene

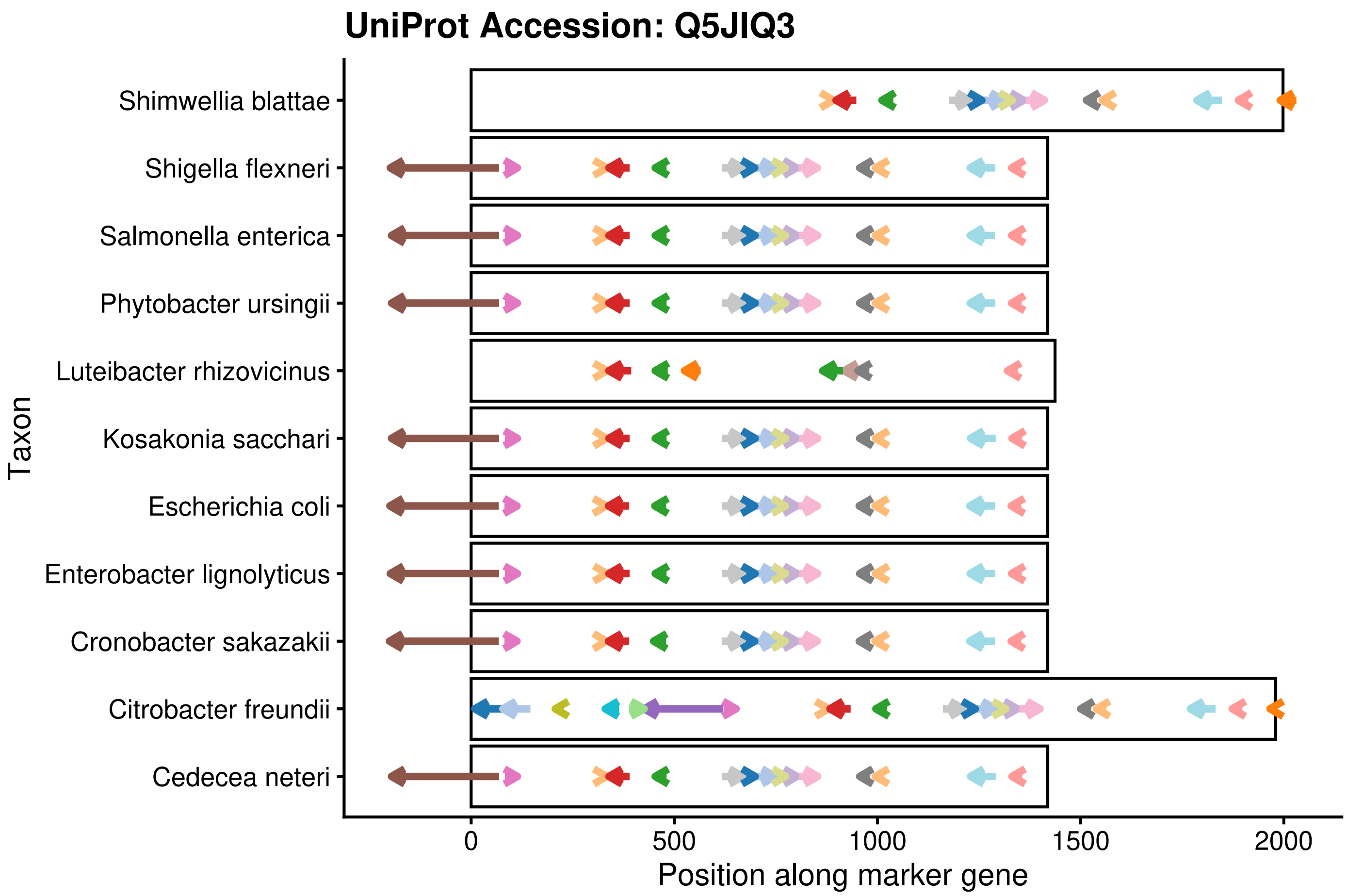
UniProt Accession: Q5JIQ3
Taxon
Shimwellia blattae
Shigella flexneri
Salmonella enterica
Phytobacter ursingii
Luteibacter rhizovicinus
Kosakonia sacchari
Escherichia coli
Enterobacter lignolyticus
Cronobacter sakazakii
Citrobacter freundii
Cedecea neteri
0
500
1000
1500
2000
Position along marker gene

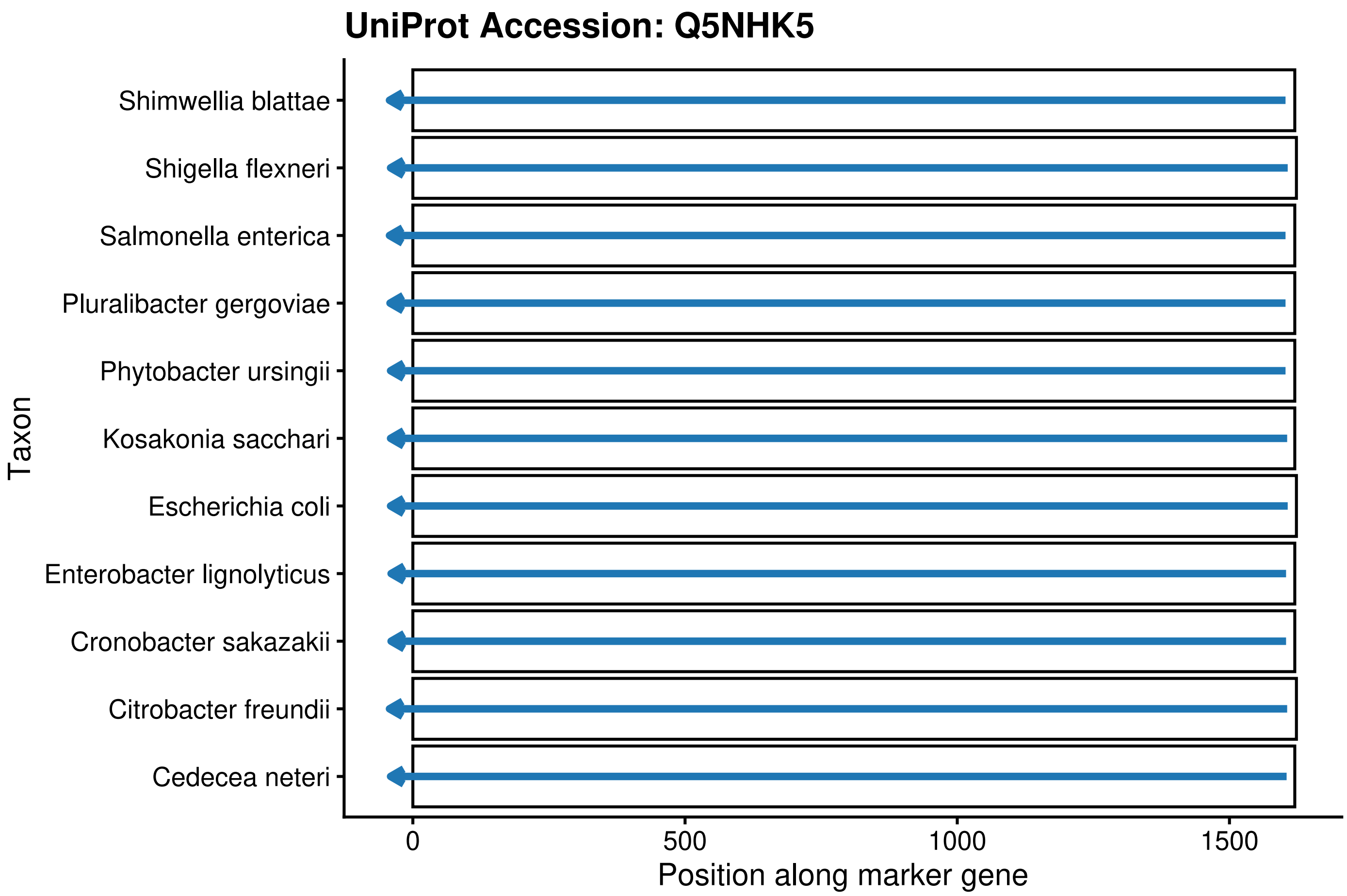
UniProt Accession: Q5NHK5
Taxon
Shimwellia blattae
Shigella flexneri
Salmonella enterica
Pluralibacter gergoviae
Phytobacter ursingii
Kosakonia sacchari
Escherichia coli
Enterobacter lignolyticus
Cronobacter sakazakii
Citrobacter freundii
Cedecea neteri
0
500
1000
1500
Position along marker gene

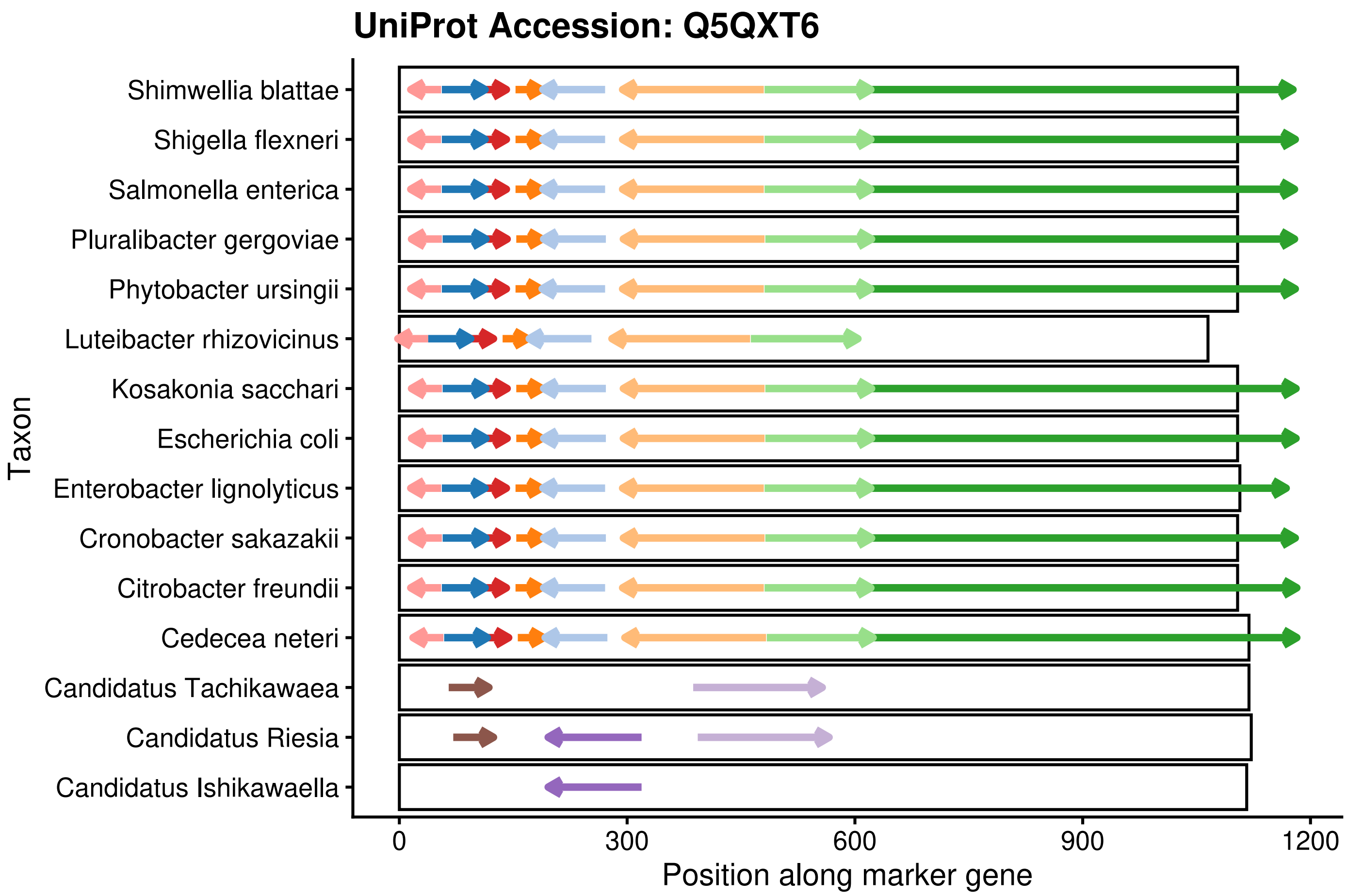
UniProt Accession: Q5QXT6
Taxon
Shimwellia blattae
Shigella flexneri
Salmonella enterica
Pluralibacter gergoviae
Phytobacter ursingii
Luteibacter rhizovicinus
Kosakonia sacchari
Escherichia coli
Enterobacter lignolyticus
Cronobacter sakazakii
Citrobacter freundii
Cedecea neteri
Candidatus Tachikawaea
Candidatus Riesia
Candidatus Ishikawaella
0
300
600
900
1200
Position along marker gene

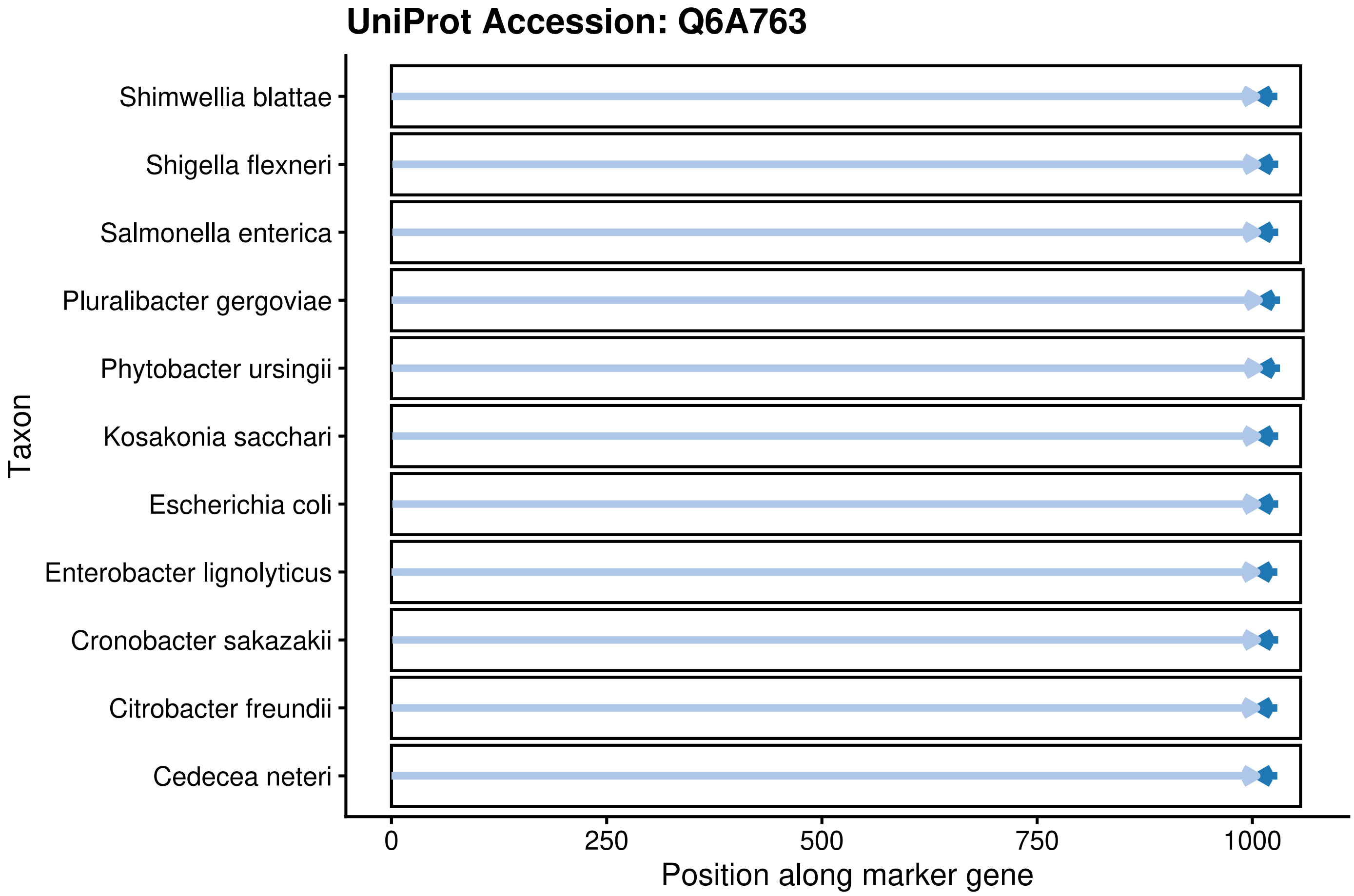
UniProt Accession: Q6A763
Taxon
Shimwellia blattae
Shigella flexneri
Salmonella enterica
Pluralibacter gergoviae
Phytobacter ursingii
Kosakonia sacchari
Escherichia coli
Enterobacter lignolyticus
Cronobacter sakazakii
Citrobacter freundii
Cedecea neteri
0
250
500
750
1000
Position along marker gene

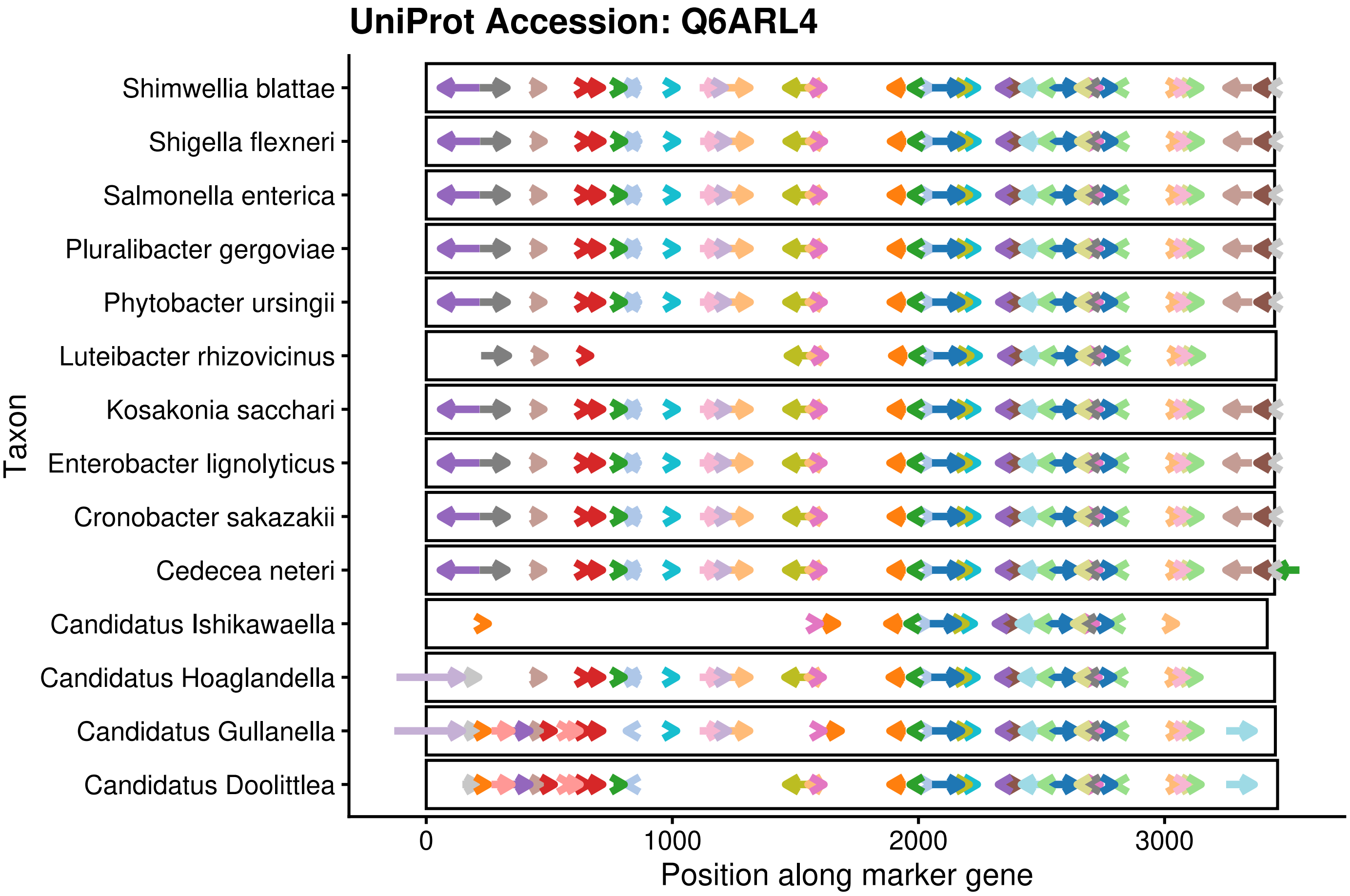
UniProt Accession: Q6ARL4
Taxon
Shimwellia blattae
Shigella flexneri
Salmonella enterica
Pluralibacter gergoviae
Phytobacter ursingii
Luteibacter rhizovicinus
Kosakonia sacchari
Enterobacter lignolyticus
Cronobacter sakazakii
Cedecea neteri
Candidatus Ishikawaella
Candidatus Hoaglandella
Candidatus Gullanella
Candidatus Doolittlea
0
1000
2000
3000
Position along marker gene

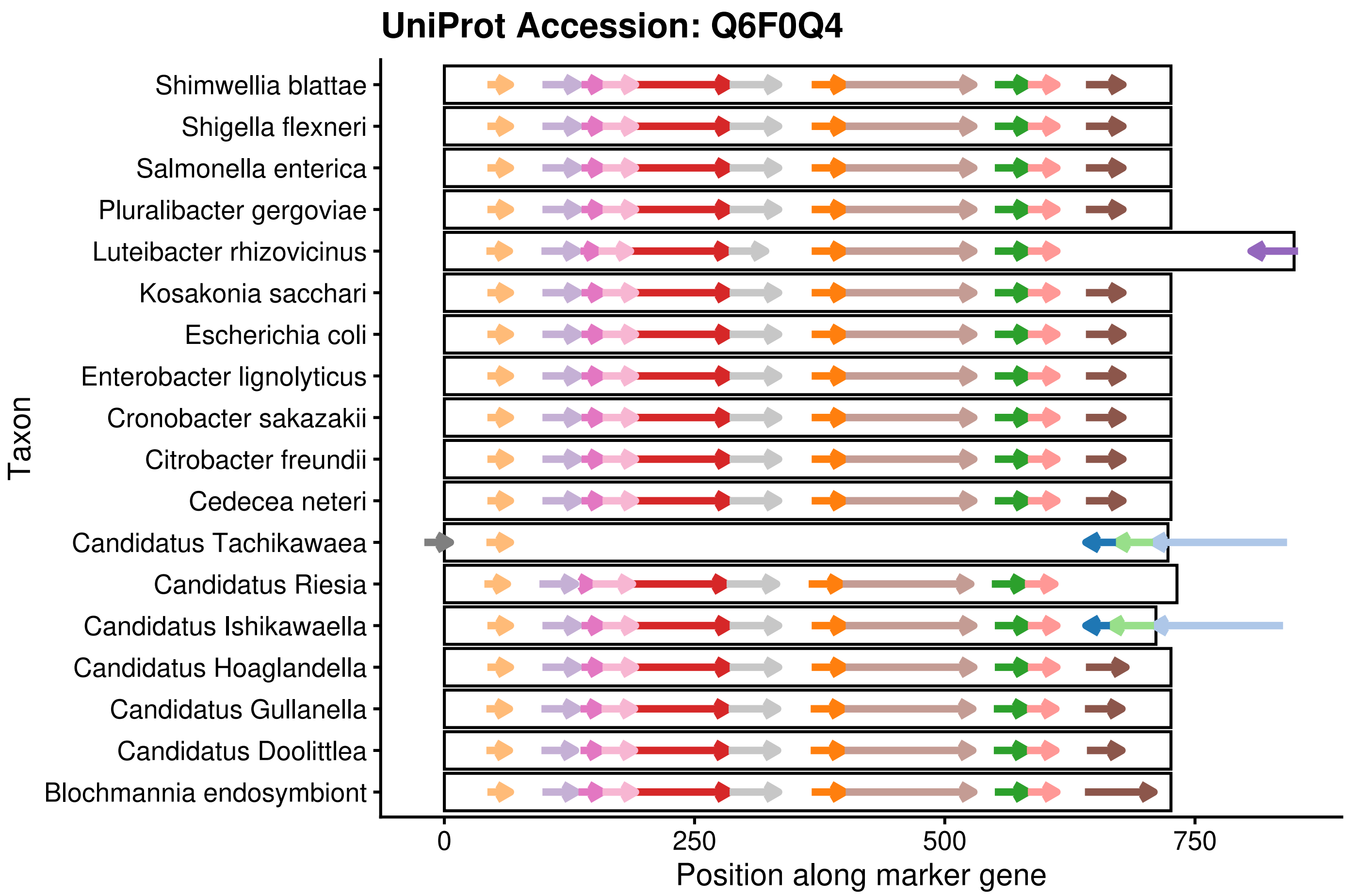
UniProt Accession: Q6F0Q4
Shimwellia blattae
Shigella flexneri
Salmonella enterica
Pluralibacter gergoviae
Luteibacter rhizovicinus
Kosakonia sacchari
Escherichia coli
Enterobacter lignolyticus
Cronobacter sakazakii
Citrobacter freundii
Cedecea neteri
Candidatus Tachikawaea
Candidatus Riesia
Candidatus Ishikawaella
Candidatus Hoaglandella
Candidatus Gullanella
Candidatus Doolittlea
Blochmannia endosymbiont
Taxon
0
250
500
750
Position along marker gene

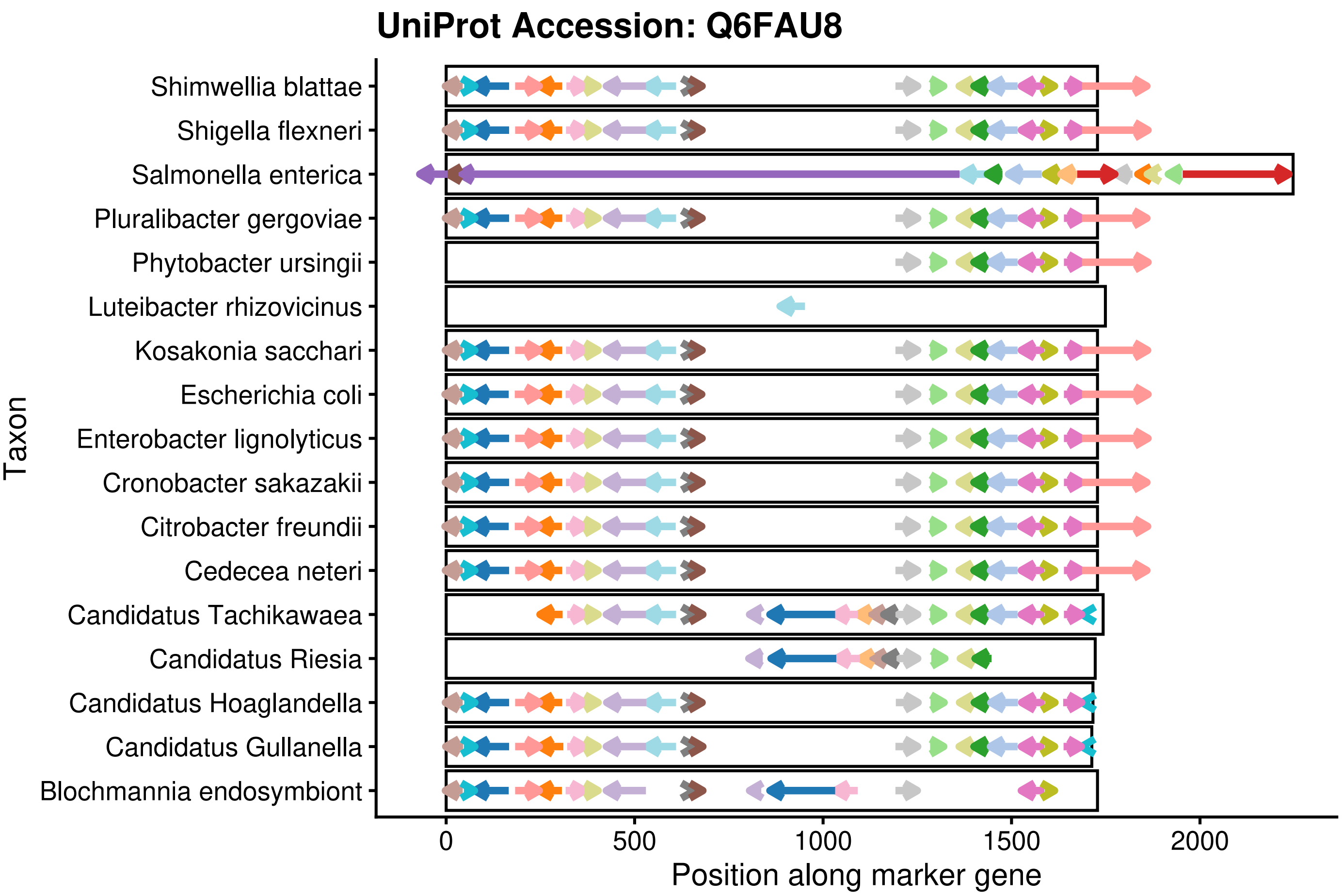
UniProt Accession: Q6FAU8
Shimwellia blattae
Shigella flexneri
Salmonella enterica
Pluralibacter gergoviae
Phytobacter ursingii
Luteibacter rhizovicinus
Kosakonia sacchari
Escherichia coli
Enterobacter lignolyticus
Cronobacter sakazakii
Citrobacter freundii
Cedecea neteri
Candidatus Tachikawaea
Candidatus Riesia
Candidatus Hoaglandella
Candidatus Gullanella
Blochmannia endosymbiont
Taxon
0
500
1000
1500
2000
Position along marker gene

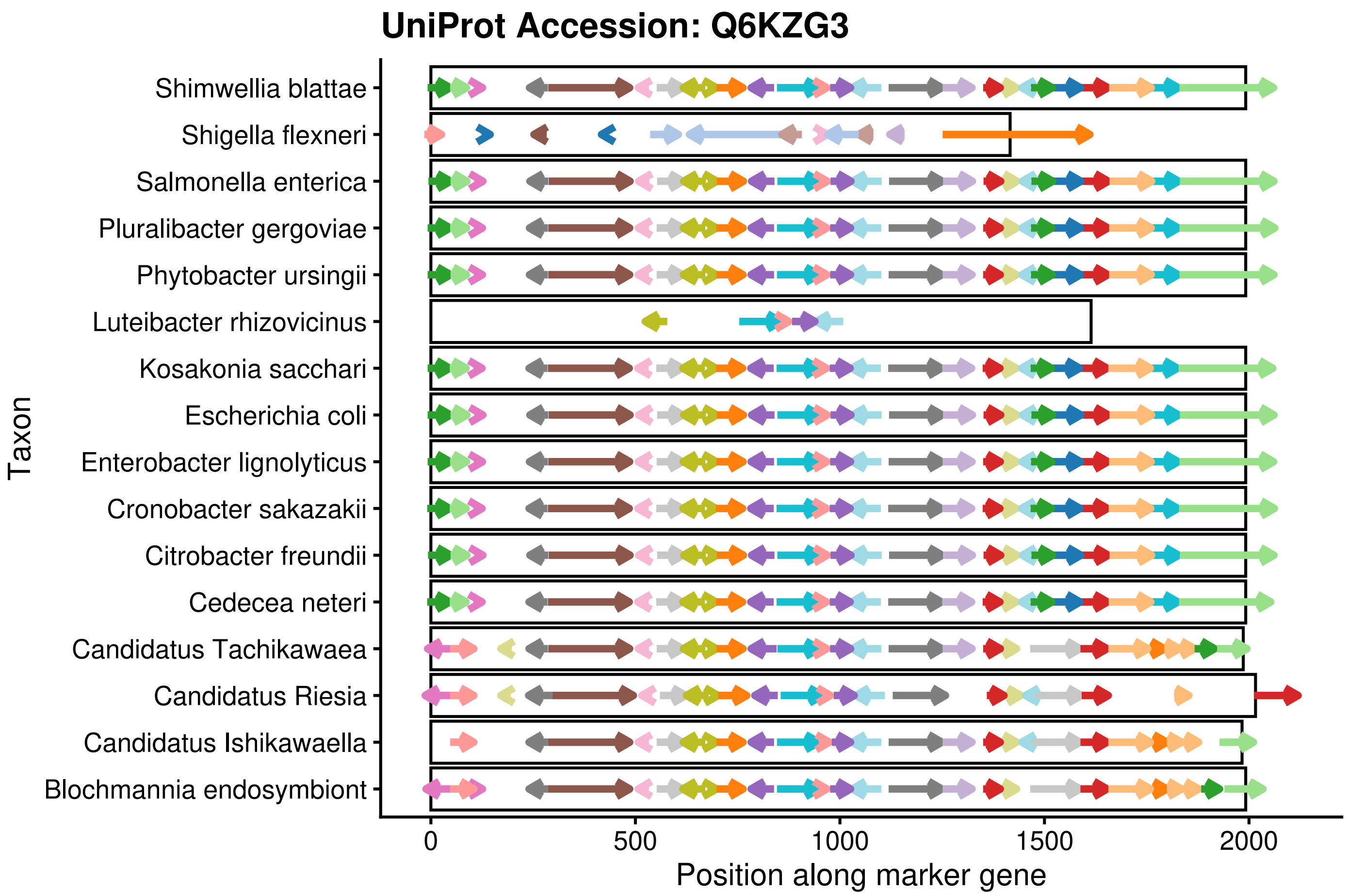

UniProt Accession: Q6KZG3
Taxon
Shimwellia blattae
Shigella flexneri
Salmonella enterica
Pluralibacter gergoviae
Phytobacter ursingii
Luteibacter rhizovicinus
Kosakonia sacchari
Escherichia coli
Enterobacter lignolyticus
Cronobacter sakazakii
Citrobacter freundii
Cedecea neteri
Candidatus Tachikawaea
Candidatus Riesia
Candidatus Ishikawaella
Blochmannia endosymbiont
0
500
1000
1500
2000
Position along marker gene

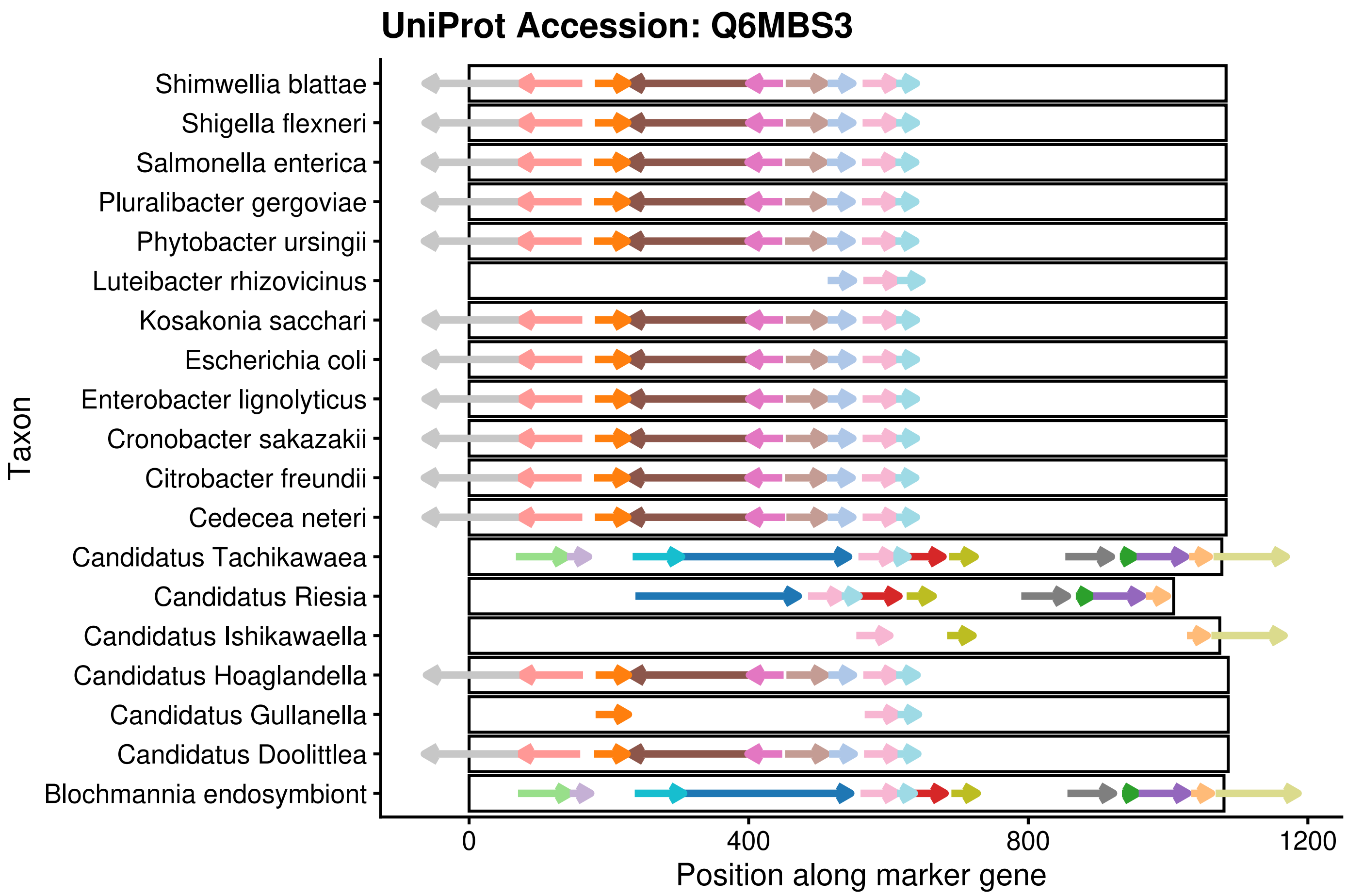

UniProt Accession: Q6MBS3
Taxon
Shimwellia blattae
Shigella flexneri
Salmonella enterica
Pluralibacter gergoviae
Phytobacter ursingii
Luteibacter rhizovicinus
Kosakonia sacchari
Escherichia coli
Enterobacter lignolyticus
Cronobacter sakazakii
Citrobacter freundii
Cedecea neteri
Candidatus Tachikawaea
Candidatus Riesia
Candidatus Ishikawaella
Candidatus Hoaglandella
Candidatus Gullanella
Candidatus Doolittlea
Blochmannia endosymbiont
0
400
800
1200
Position along marker gene

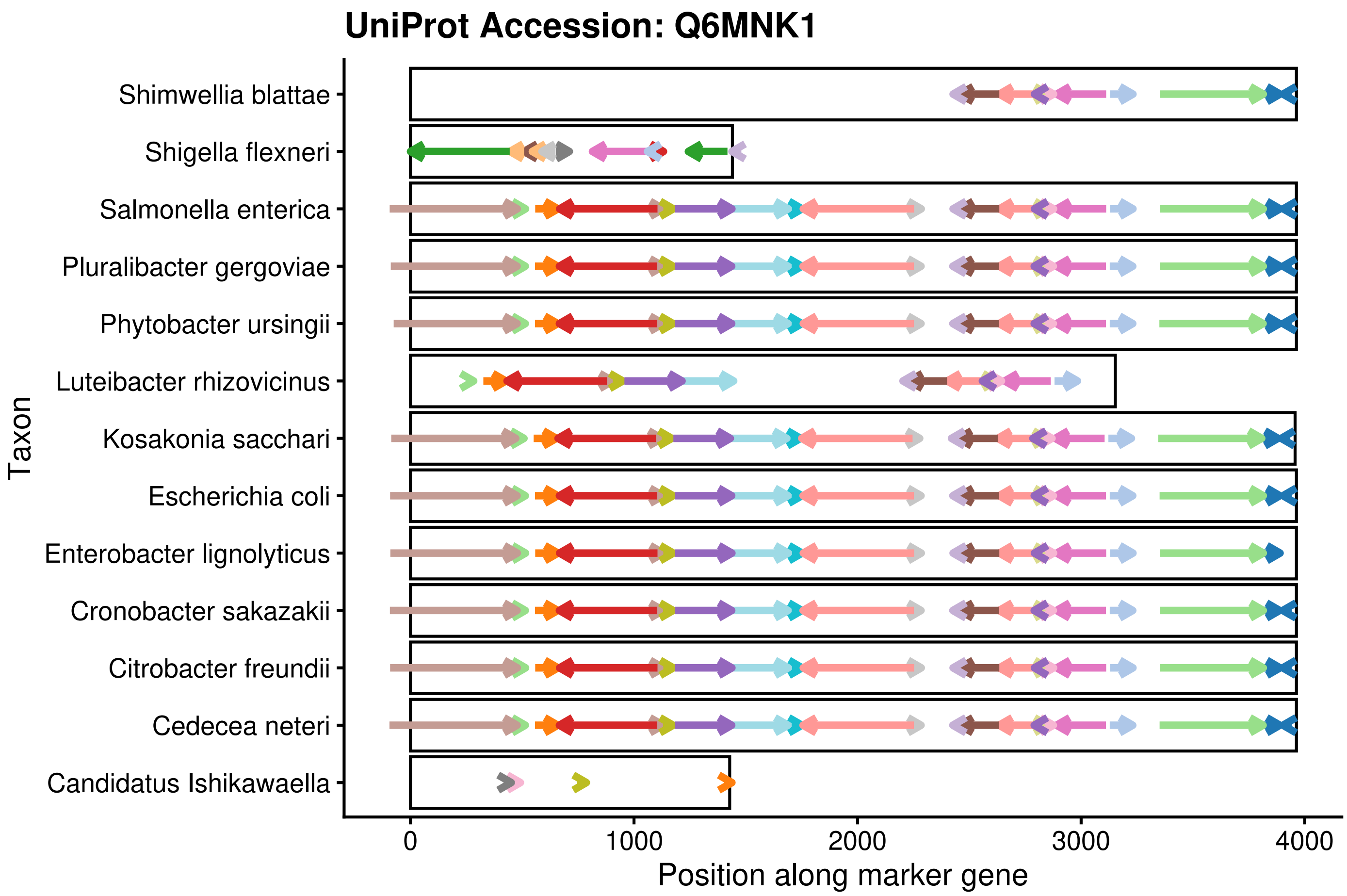

UniProt Accession: Q6MNK1
Shimwellia blattae
Shigella flexneri
Salmonella enterica
Pluralibacter gergoviae
Phytobacter ursingii
Luteibacter rhizovicinus
Kosakonia sacchari
Escherichia coli
Enterobacter lignolyticus
Cronobacter sakazakii
Citrobacter freundii
Cedecea neteri
Candidatus Ishikawaella
Taxon
0
1000
2000
3000
4000
Position along marker gene

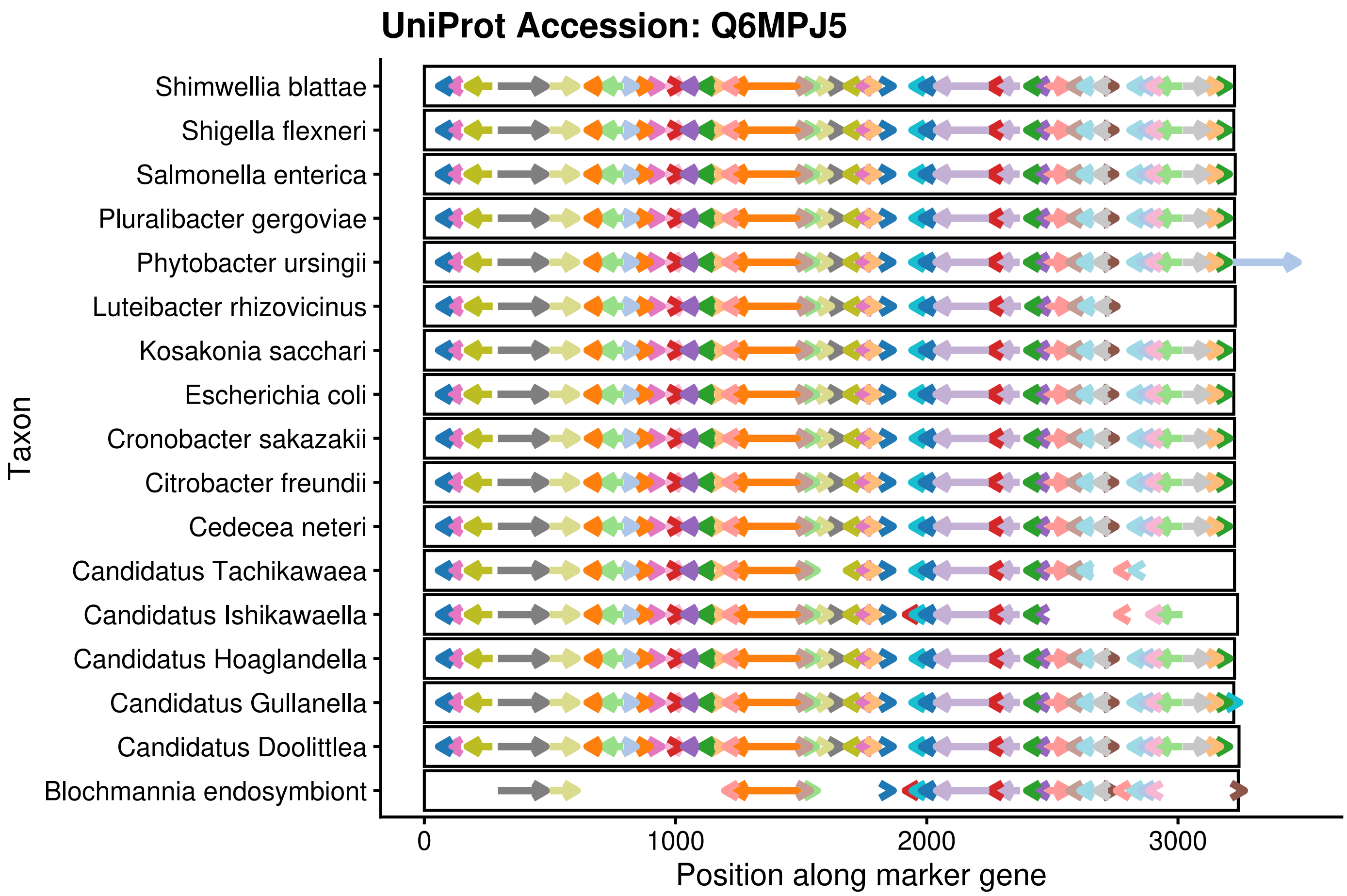
UniProt Accession: Q6MPJ5
Shimwellia blattae
Shigella flexneri
Salmonella enterica
Pluralibacter gergoviae
Phytobacter ursingii
Luteibacter rhizovicinus
Kosakonia sacchari
Escherichia coli
Cronobacter sakazakii
Citrobacter freundii
Cedecea neteri
Candidatus Tachikawaea
Candidatus Ishikawaella
Candidatus Hoaglandella
Candidatus Gullanella
Candidatus Doolittlea
Blochmannia endosymbiont
Taxon
0
1000
2000
3000
Position along marker gene

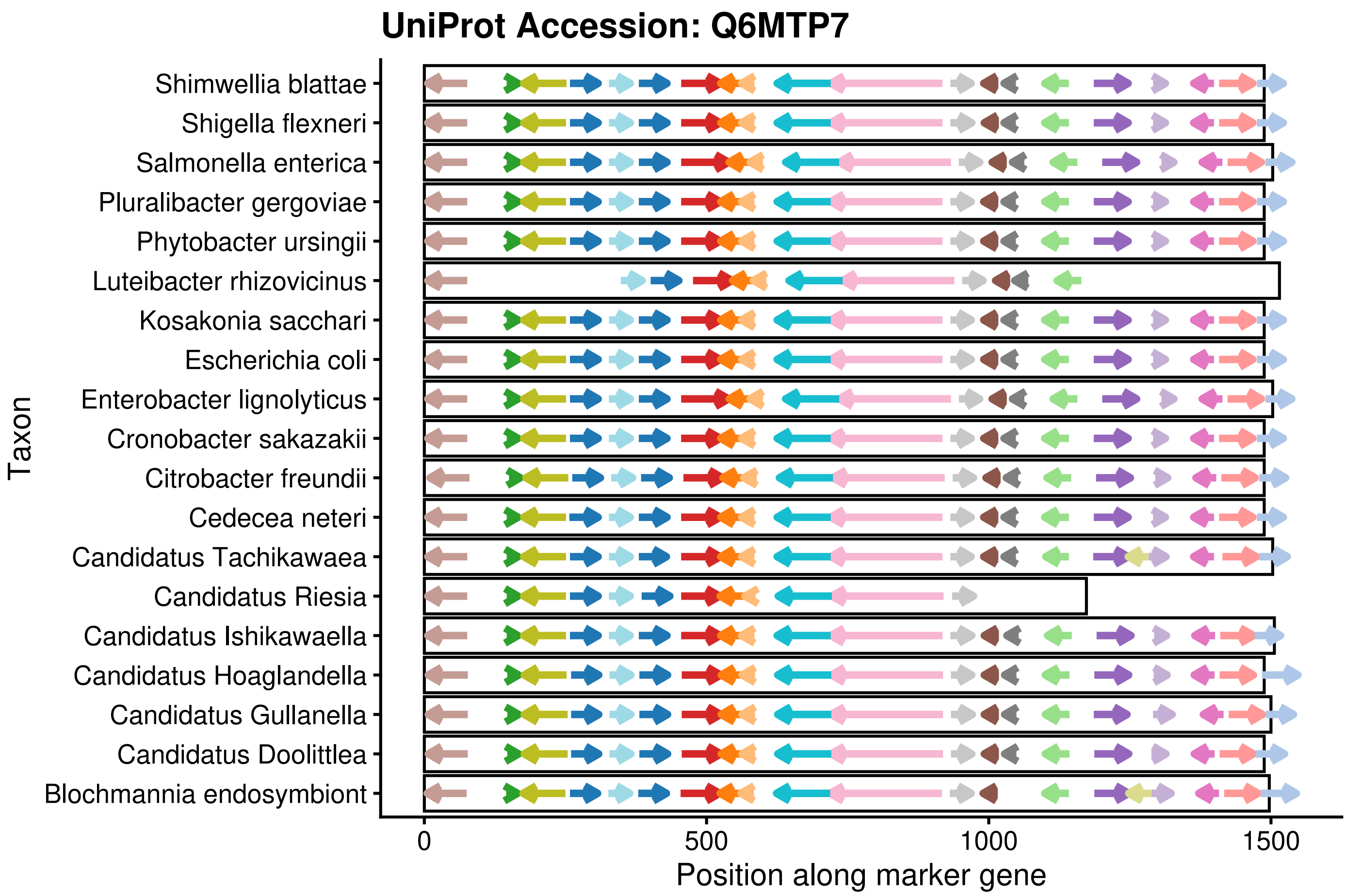
UniProt Accession: Q6MTP7
Shimwellia blattae
Shigella flexneri
Salmonella enterica
Pluralibacter gergoviae
Phytobacter ursingii
Luteibacter rhizovicinus
Kosakonia sacchari
Escherichia coli
Enterobacter lignolyticus
Cronobacter sakazakii
Citrobacter freundii
Cedecea neteri
Candidatus Tachikawaea
Candidatus Riesia
Candidatus Ishikawaella
Candidatus Hoaglandella
Candidatus Gullanella
Candidatus Doolittlea
Blochmannia endosymbiont
Taxon
0
500
1000
1500
Position along marker gene

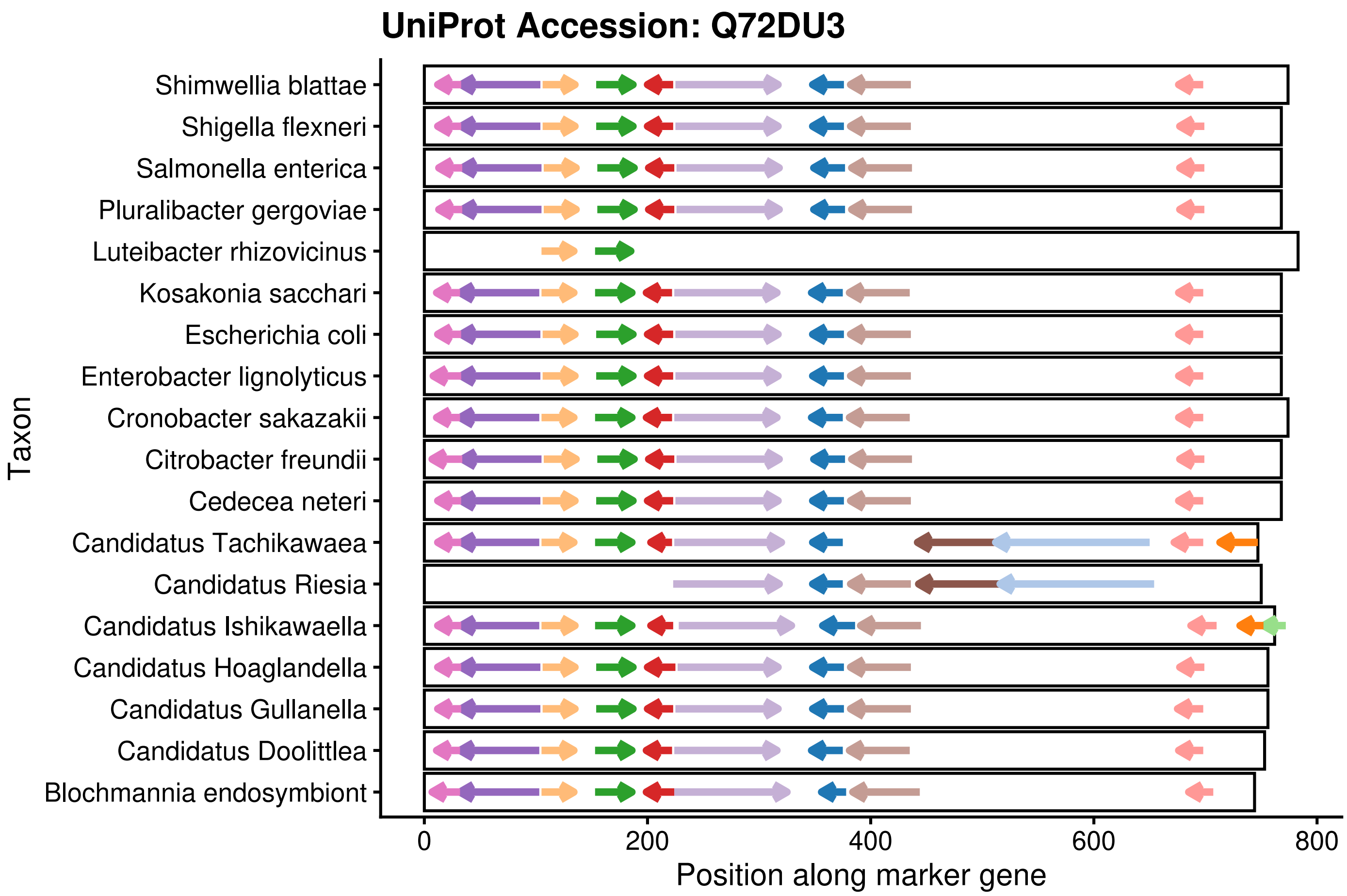

UniProt Accession: Q72DU3
Taxon
Shimwellia blattae
Shigella flexneri
Salmonella enterica
Pluralibacter gergoviae
Luteibacter rhizovicinus
Kosakonia sacchari
Escherichia coli
Enterobacter lignolyticus
Cronobacter sakazakii
Citrobacter freundii
Cedecea neteri
Candidatus Tachikawaea
Candidatus Riesia
Candidatus Ishikawaella
Candidatus Hoaglandella
Candidatus Gullanella
Candidatus Doolittlea
Blochmannia endosymbiont
0
200
400
600
800
Position along marker gene

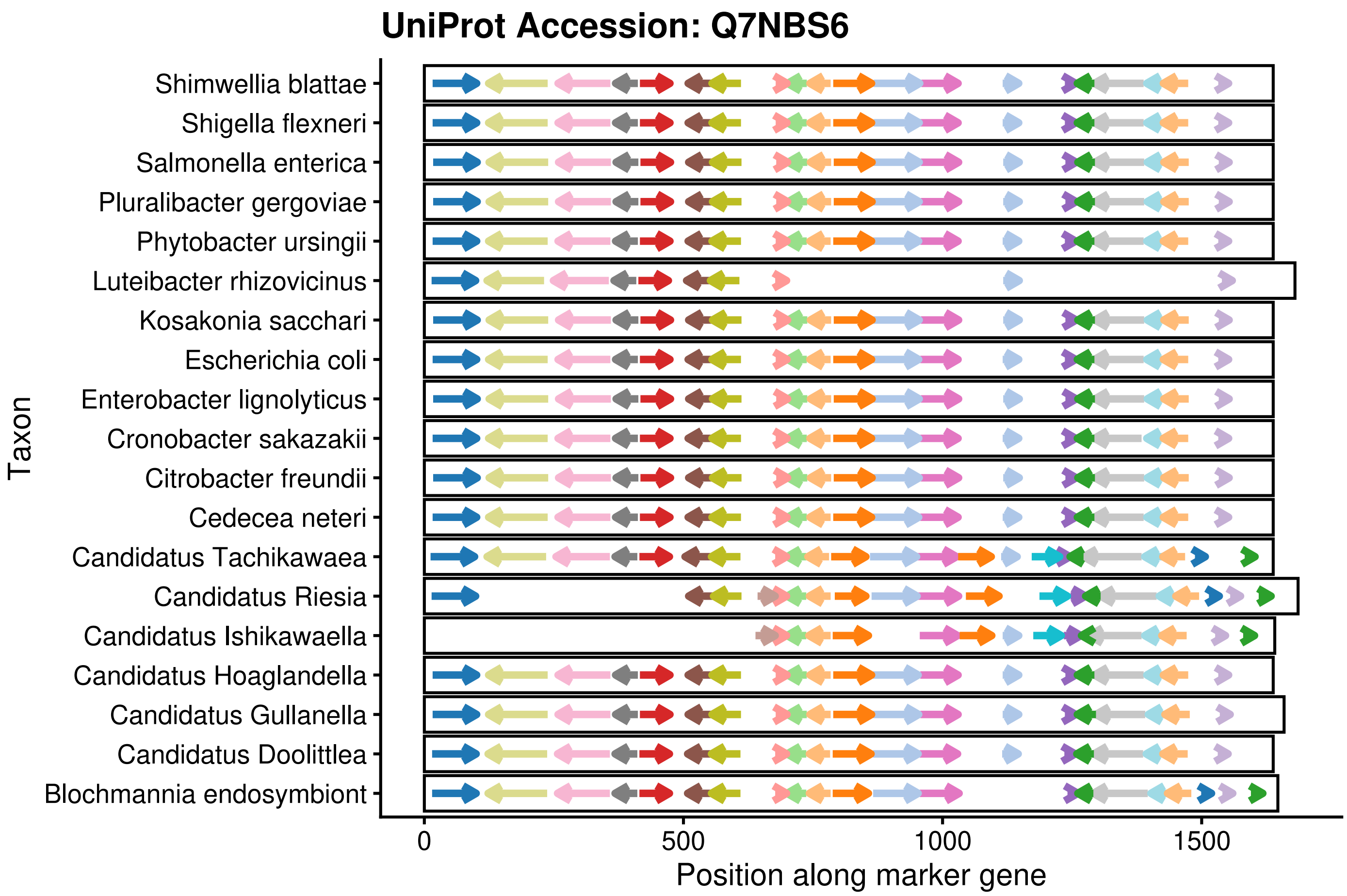
UniProt Accession: Q7NBS6
Taxon
Shimwellia blattae
Shigella flexneri
Salmonella enterica
Pluralibacter gergoviae
Phytobacter ursingii
Luteibacter rhizovicinus
Kosakonia sacchari
Escherichia coli
Enterobacter lignolyticus
Cronobacter sakazakii
Citrobacter freundii
Cedecea neteri
Candidatus Tachikawaea
Candidatus Riesia
Candidatus Ishikawaella
Candidatus Hoaglandella
Candidatus Gullanella
Candidatus Doolittlea
Blochmannia endosymbiont
0
500
1000
1500
Position along marker gene

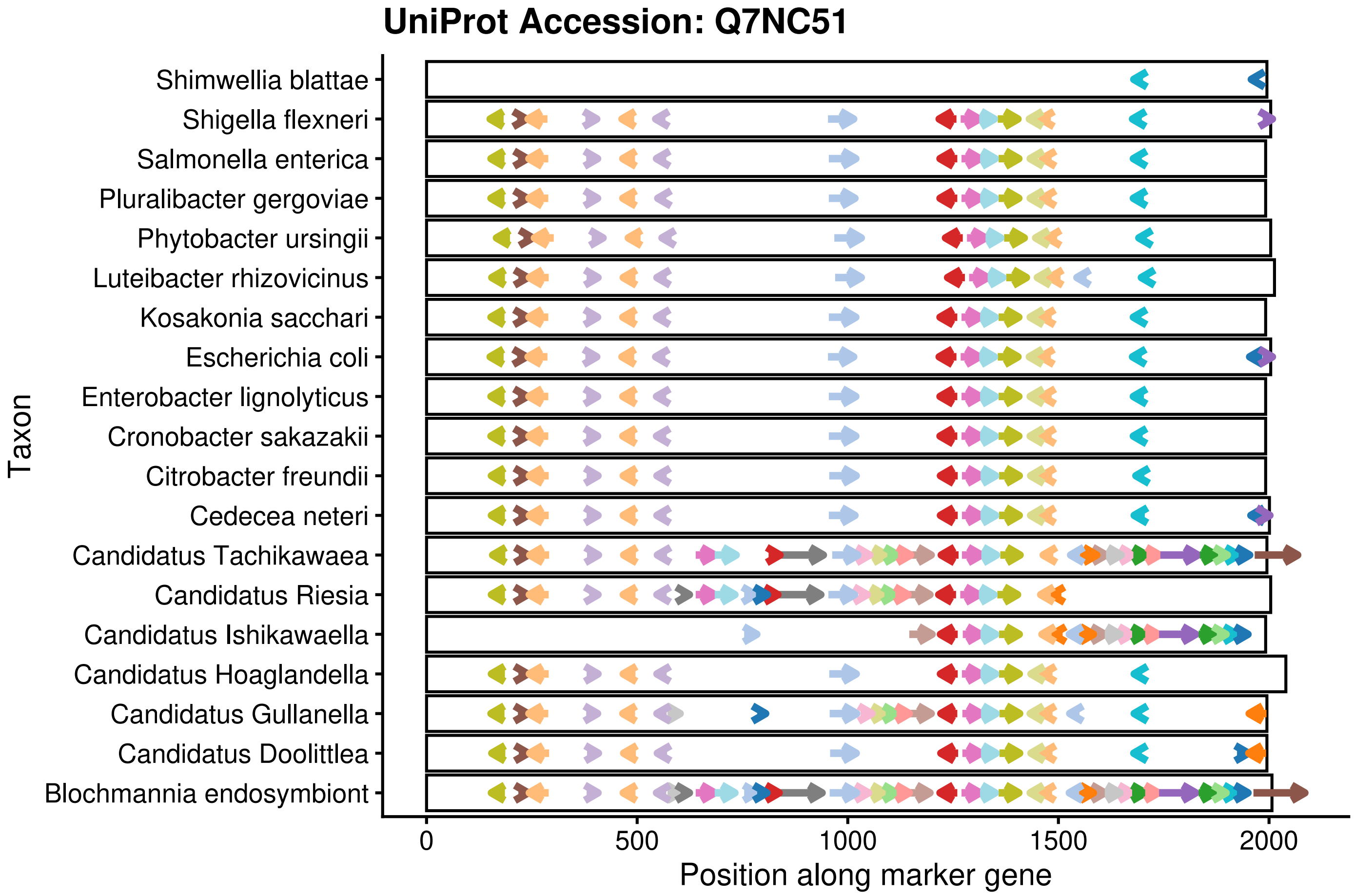
UniProt Accession: Q7NC51
Taxon
Shimwellia blattae
Shigella flexneri
Salmonella enterica
Pluralibacter gergoviae
Phytobacter ursingii
Luteibacter rhizovicinus
Kosakonia sacchari
Escherichia coli
Enterobacter lignolyticus
Cronobacter sakazakii
Citrobacter freundii
Cedecea neteri
Candidatus Tachikawaea
Candidatus Riesia
Candidatus Ishikawaella
Candidatus Hoaglandella
Candidatus Gullanella
Candidatus Doolittlea
Blochmannia endosymbiont
0
500
1000
1500
2000
Position along marker gene

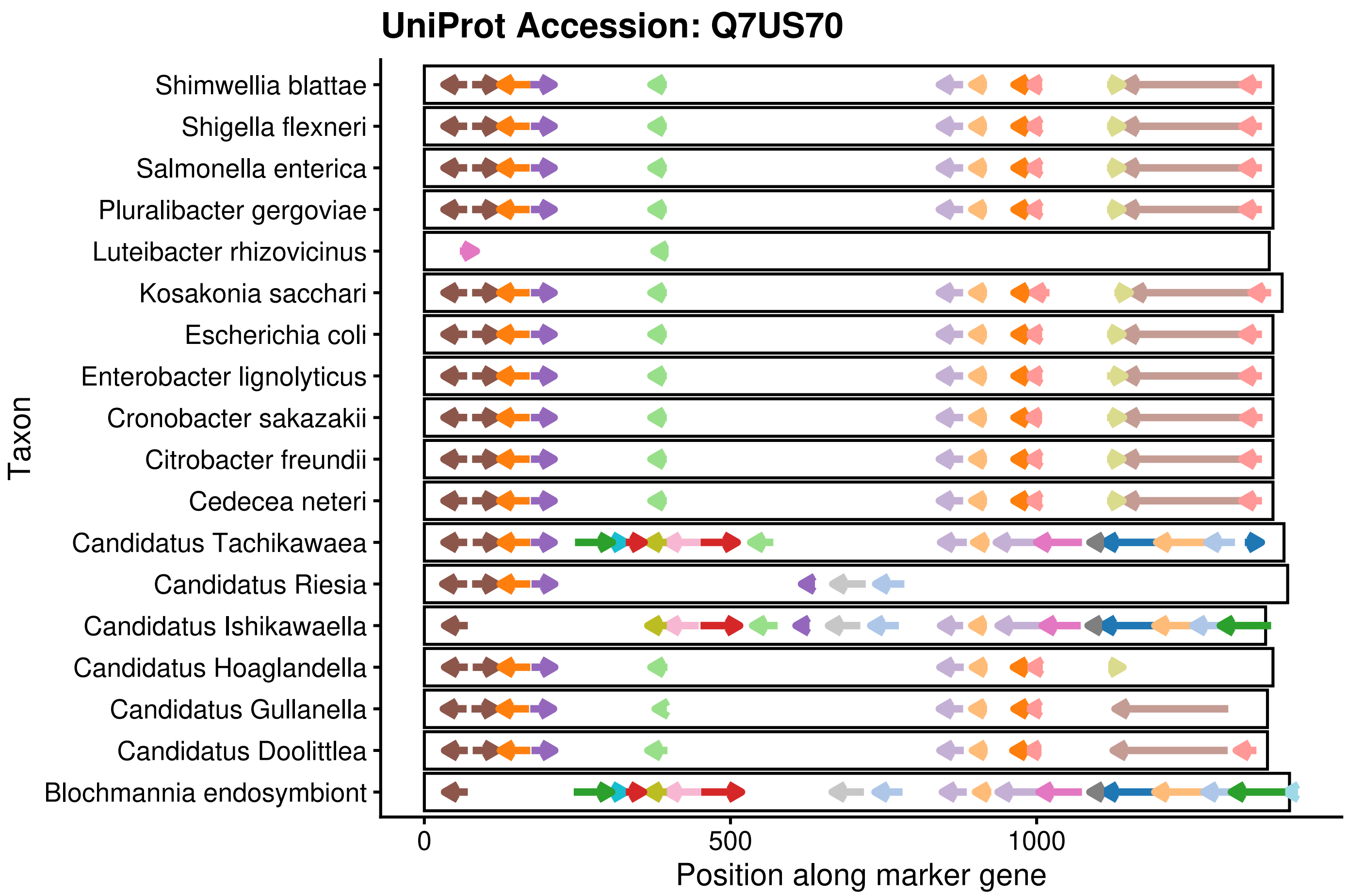
UniProt Accession: Q7US70
Taxon
Shimwellia blattae
Shigella flexneri
Salmonella enterica
Pluralibacter gergoviae
Luteibacter rhizovicinus
Kosakonia sacchari
Escherichia coli
Enterobacter lignolyticus
Cronobacter sakazakii
Citrobacter freundii
Cedecea neteri
Candidatus Tachikawaea
Candidatus Riesia
Candidatus Ishikawaella
Candidatus Hoaglandella
Candidatus Gullanella
Candidatus Doolittlea
Blochmannia endosymbiont
0
500
1000
Position along marker gene

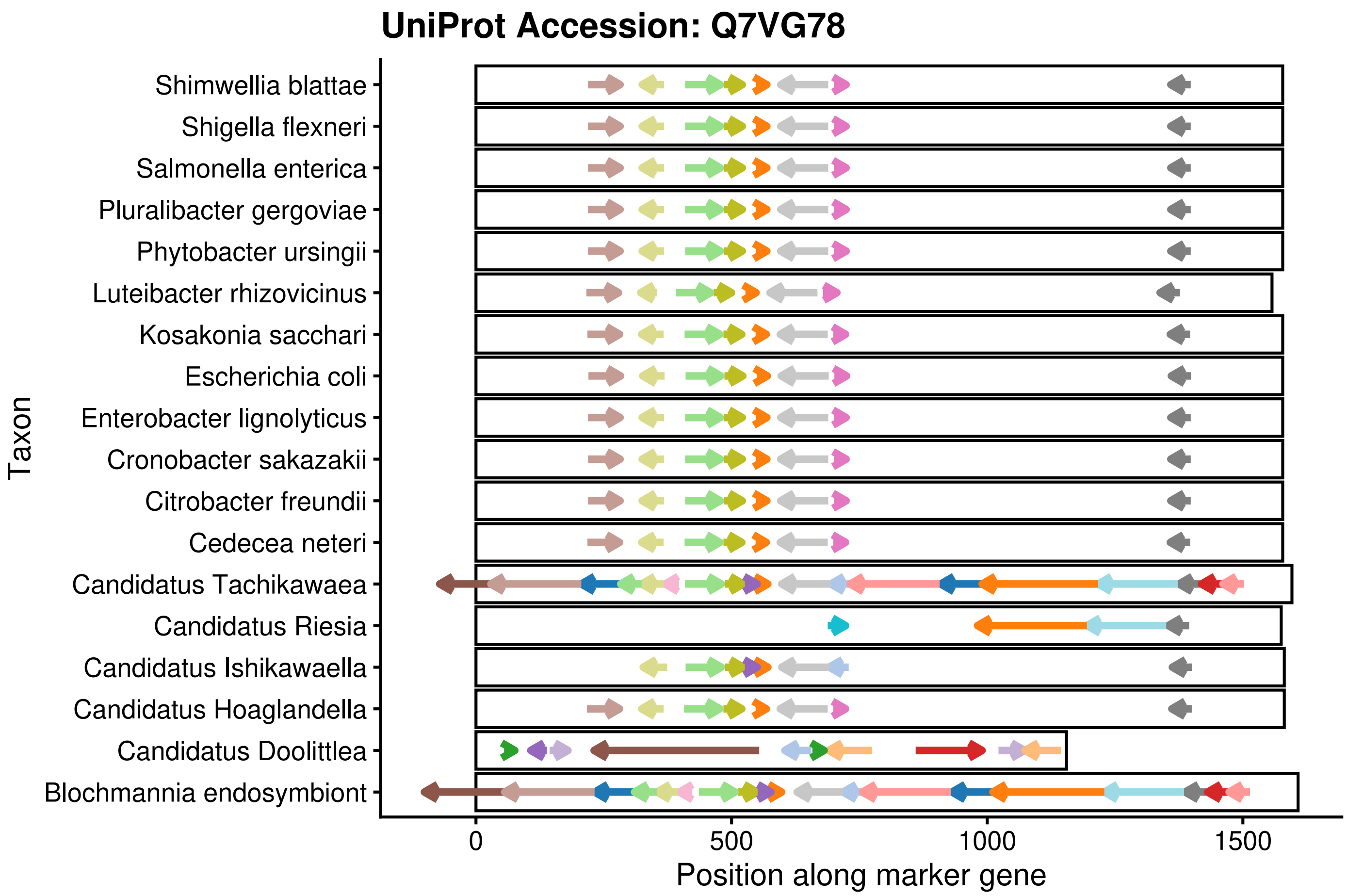
UniProt Accession: Q7VG78
Shimwellia blattae
Shigella flexneri
Salmonella enterica
Pluralibacter gergoviae
Phytobacter ursingii
Luteibacter rhizovicinus
Kosakonia sacchari
Escherichia coli
Enterobacter lignolyticus
Cronobacter sakazakii
Citrobacter freundii
Cedecea neteri
Candidatus Tachikawaea
Candidatus Riesia
Candidatus Ishikawaella
Candidatus Hoaglandella
Candidatus Doolittlea
Blochmannia endosymbiont
Taxon
0
500
1000
1500
Position along marker gene

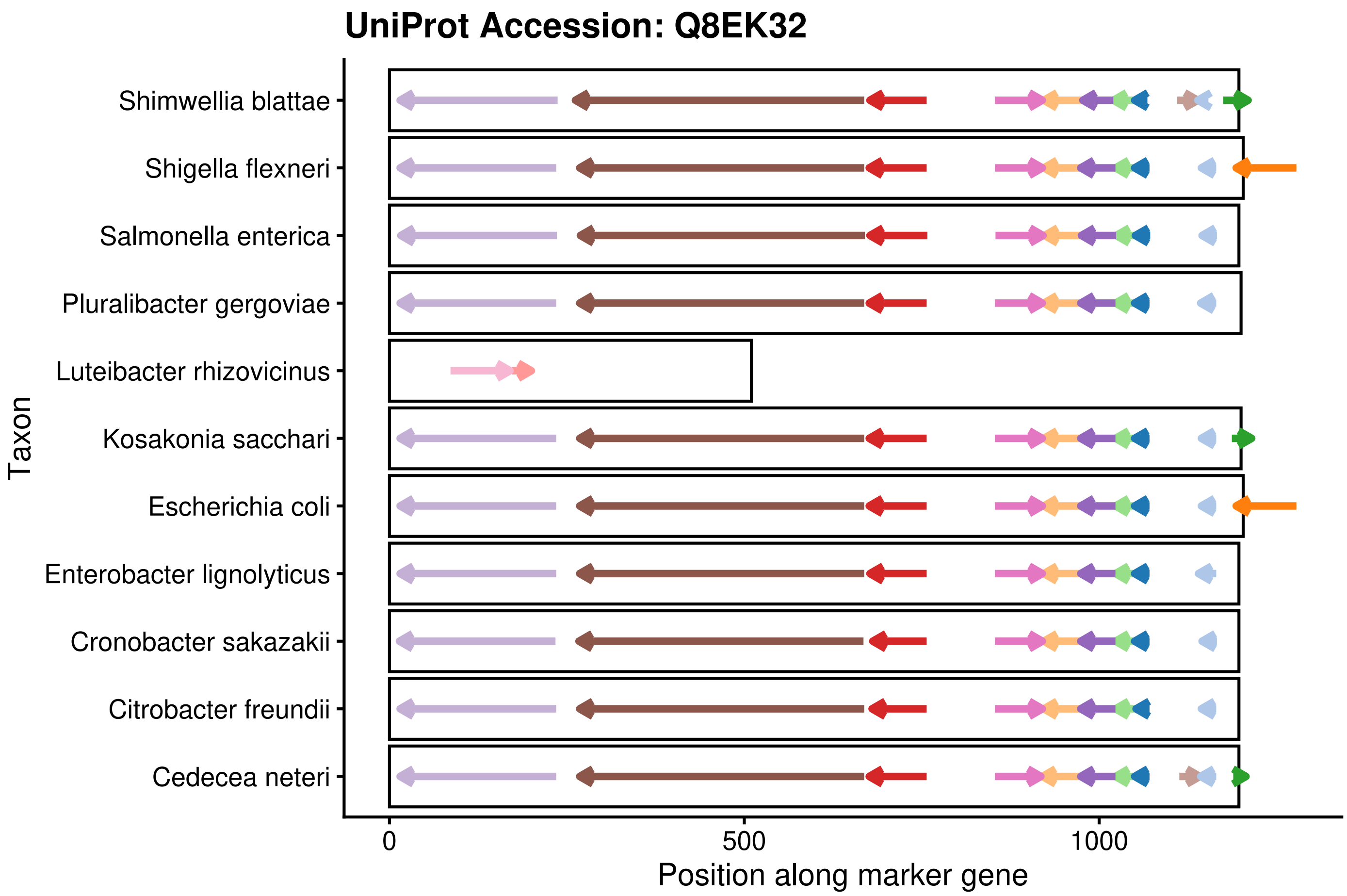

UniProt Accession: Q8EK32
Taxon
Shimwellia blattae
Shigella flexneri
Salmonella enterica
Pluralibacter gergoviae
Luteibacter rhizovicinus
Kosakonia sacchari
Escherichia coli
Enterobacter lignolyticus
Cronobacter sakazakii
Citrobacter freundii
Cedecea neteri
0
500
1000
Position along marker gene

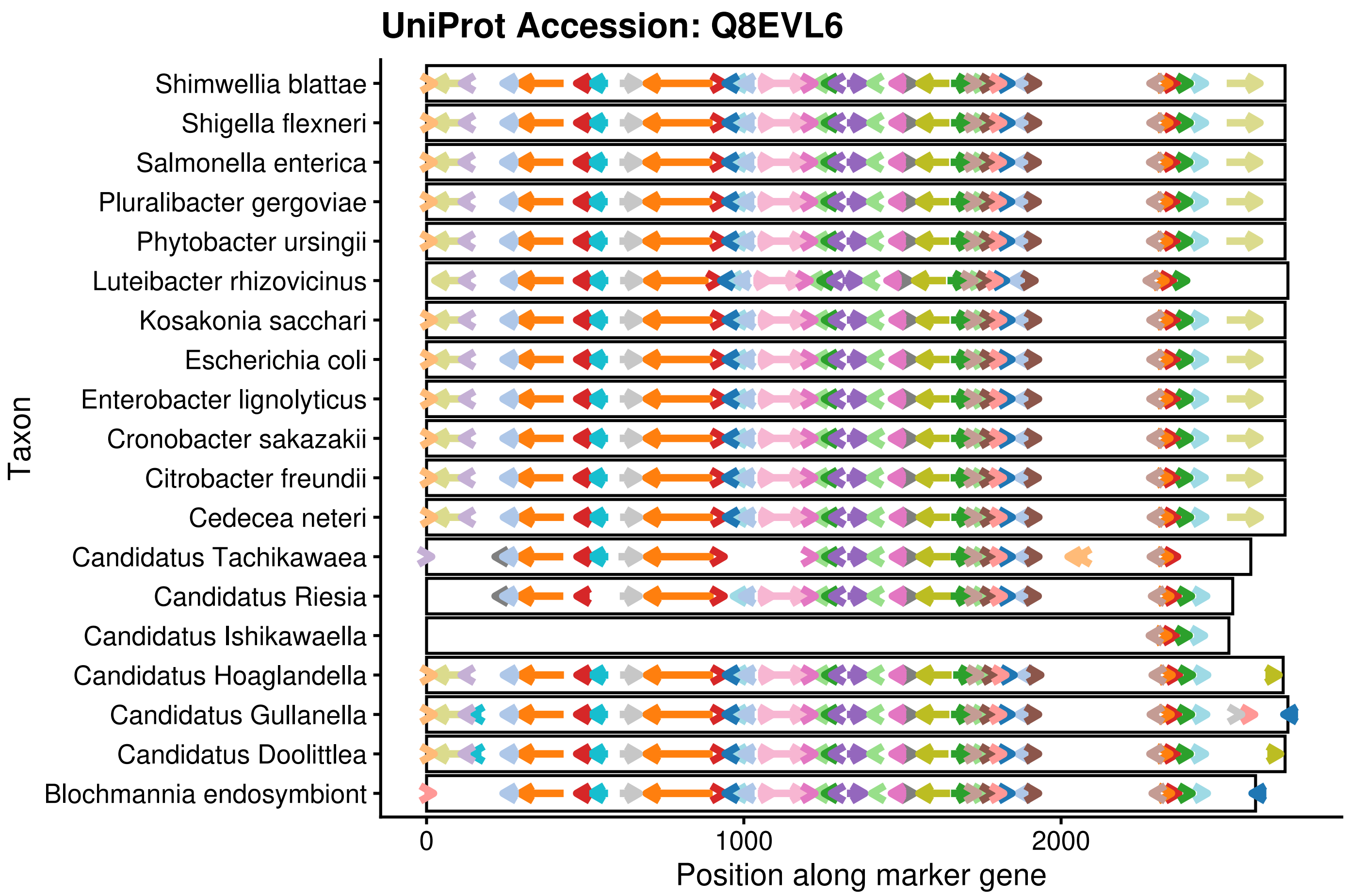
UniProt Accession: Q8EVL6
Taxon
Shimwellia blattae
Shigella flexneri
Salmonella enterica
Pluralibacter gergoviae
Phytobacter ursingii
Luteibacter rhizovicinus
Kosakonia sacchari
Escherichia coli
Enterobacter lignolyticus
Cronobacter sakazakii
Citrobacter freundii
Cedecea neteri
Candidatus Tachikawaea
Candidatus Riesia
Candidatus Ishikawaella
Candidatus Hoaglandella
Candidatus Gullanella
Candidatus Doolittlea
Blochmannia endosymbiont
0
1000
2000
Position along marker gene

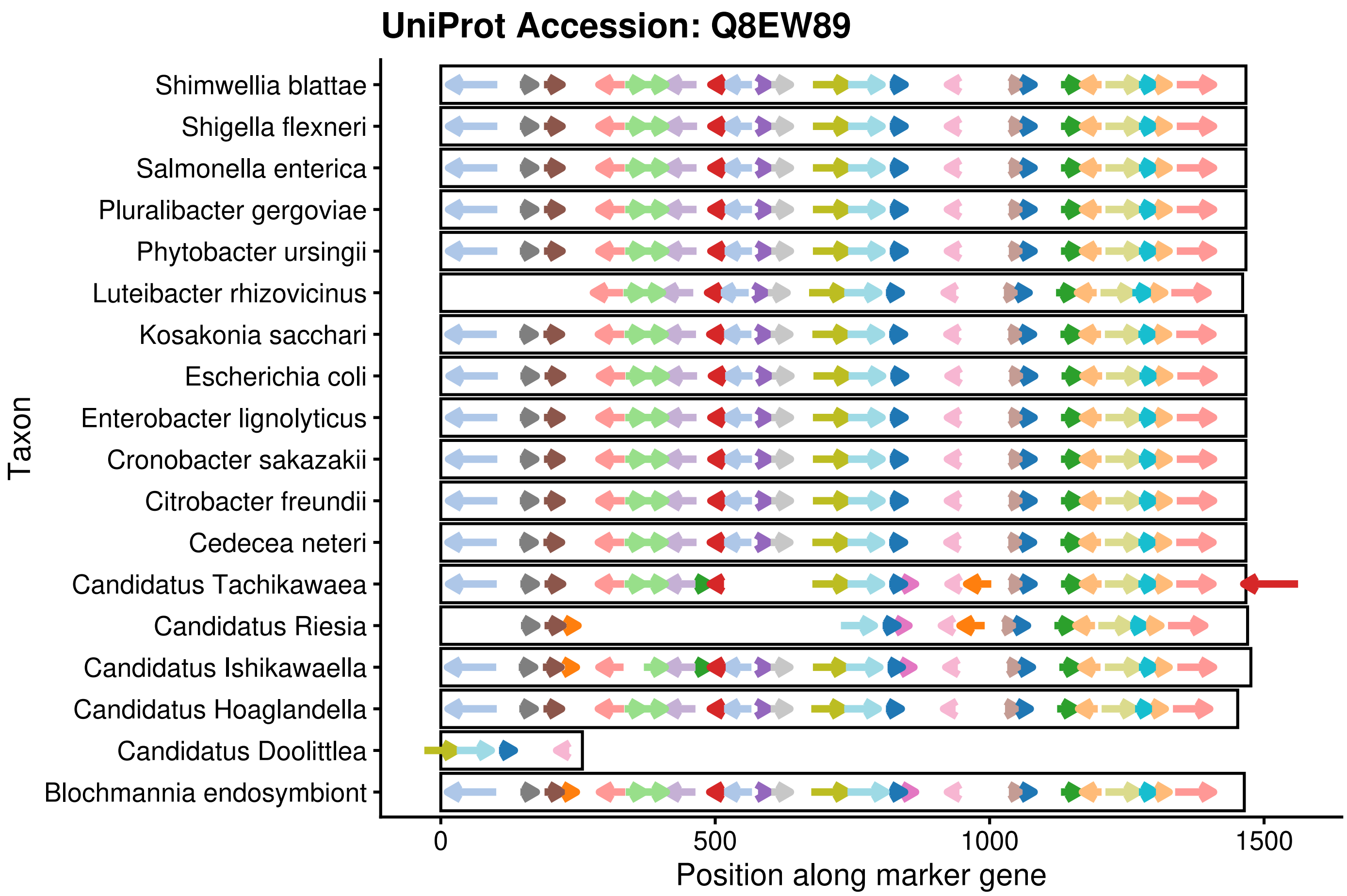

UniProt Accession: Q8EW89
Taxon
Shimwellia blattae
Shigella flexneri
Salmonella enterica
Pluralibacter gergoviae
Phytobacter ursingii
Luteibacter rhizovicinus
Kosakonia sacchari
Escherichia coli
Enterobacter lignolyticus
Cronobacter sakazakii
Citrobacter freundii
Cedecea neteri
Candidatus Tachikawaea
Candidatus Riesia
Candidatus Ishikawaella
Candidatus Hoaglandella
Candidatus Doolittlea
Blochmannia endosymbiont
0
500
1000
1500
Position along marker gene

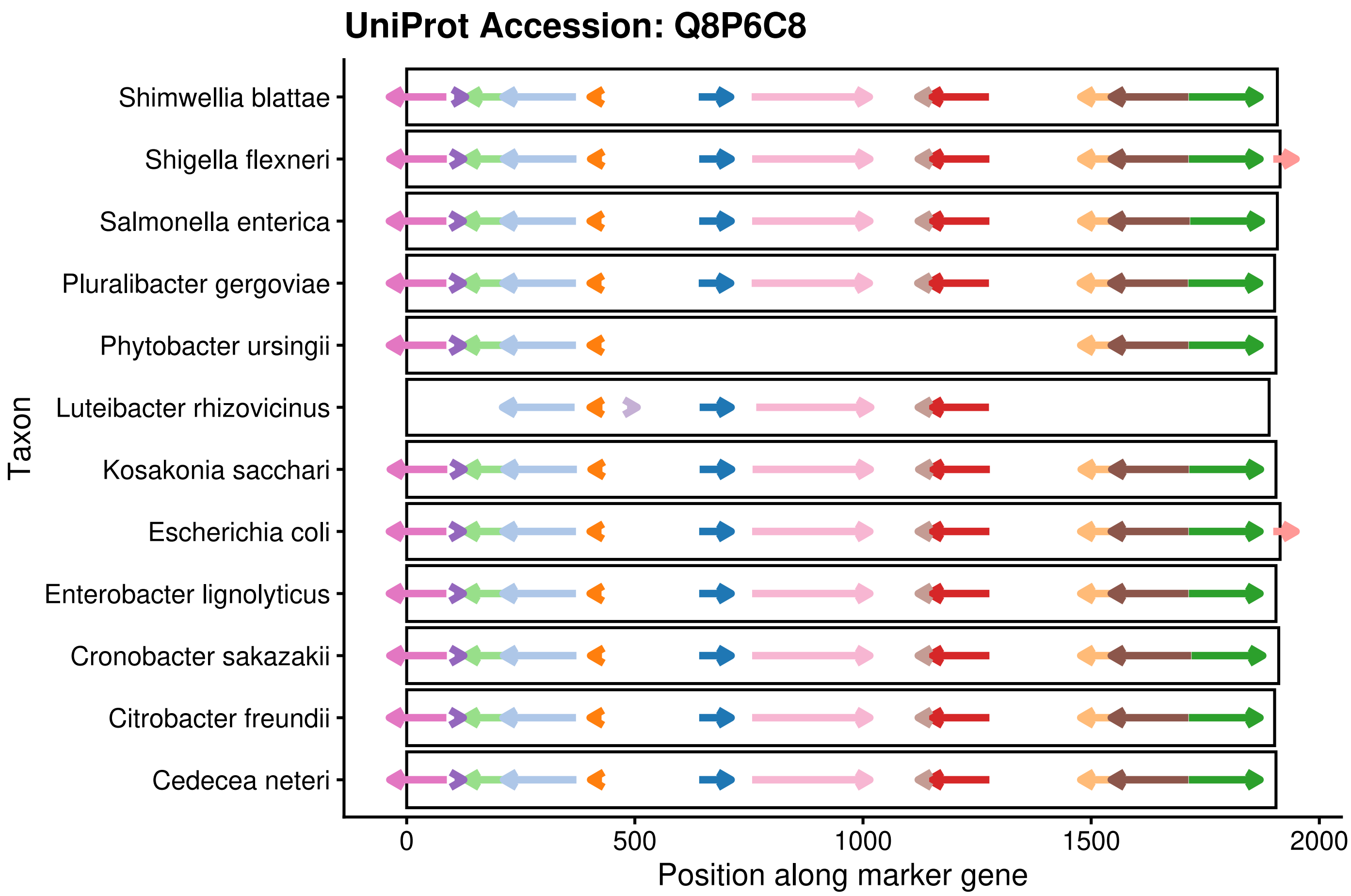
UniProt Accession: Q8P6C8
Taxon
Shimwellia blattae
Shigella flexneri
Salmonella enterica
Pluralibacter gergoviae
Phytobacter ursingii
Luteibacter rhizovicinus
Kosakonia sacchari
Escherichia coli
Enterobacter lignolyticus
Cronobacter sakazakii
Citrobacter freundii
Cedecea neteri
0
500
1000
1500
2000
Position along marker gene

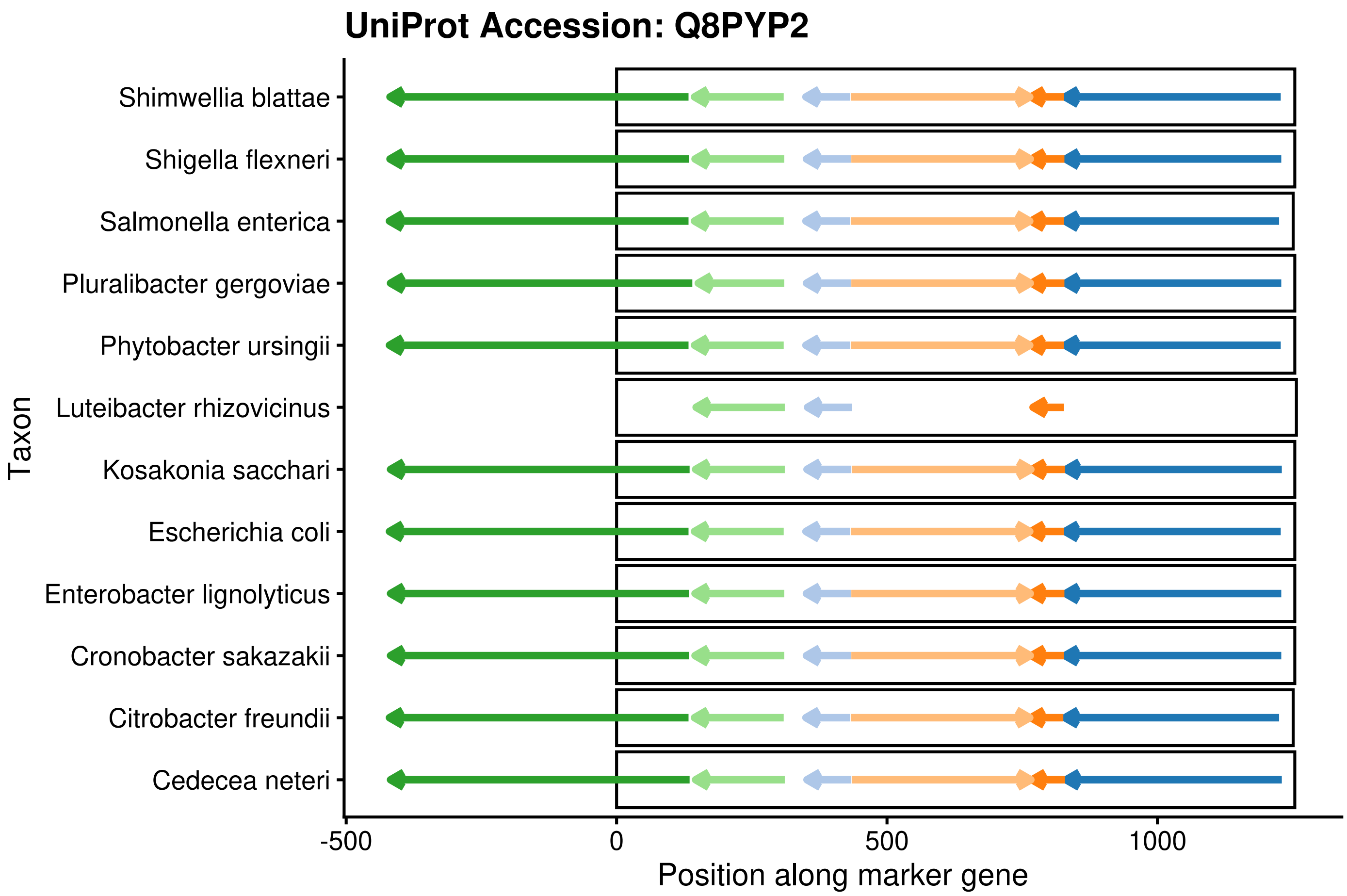
UniProt Accession: Q8PYP2
Taxon
Shimwellia blattae
Shigella flexneri
Salmonella enterica
Pluralibacter gergoviae
Phytobacter ursingii
Luteibacter rhizovicinus
Kosakonia sacchari
Escherichia coli
Enterobacter lignolyticus
Cronobacter sakazakii
Citrobacter freundii
Cedecea neteri
-500
0
500
1000
Position along marker gene

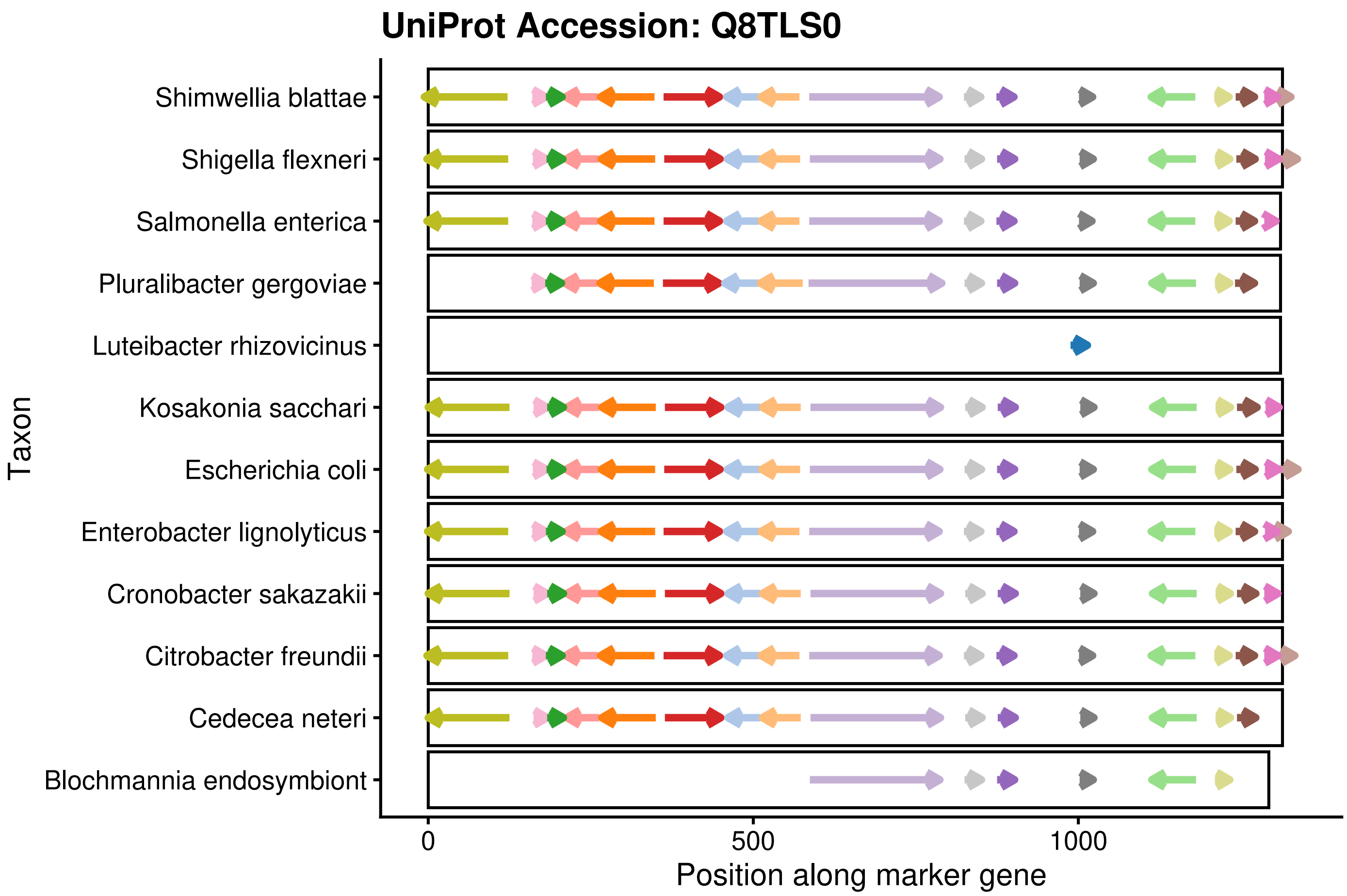

UniProt Accession: Q8TLS0
Taxon
Shimwellia blattae
Shigella flexneri
Salmonella enterica
Pluralibacter gergoviae
Luteibacter rhizovicinus
Kosakonia sacchari
Escherichia coli
Enterobacter lignolyticus
Cronobacter sakazakii
Citrobacter freundii
Cedecea neteri
Blochmannia endosymbiont
0
500
1000
Position along marker gene

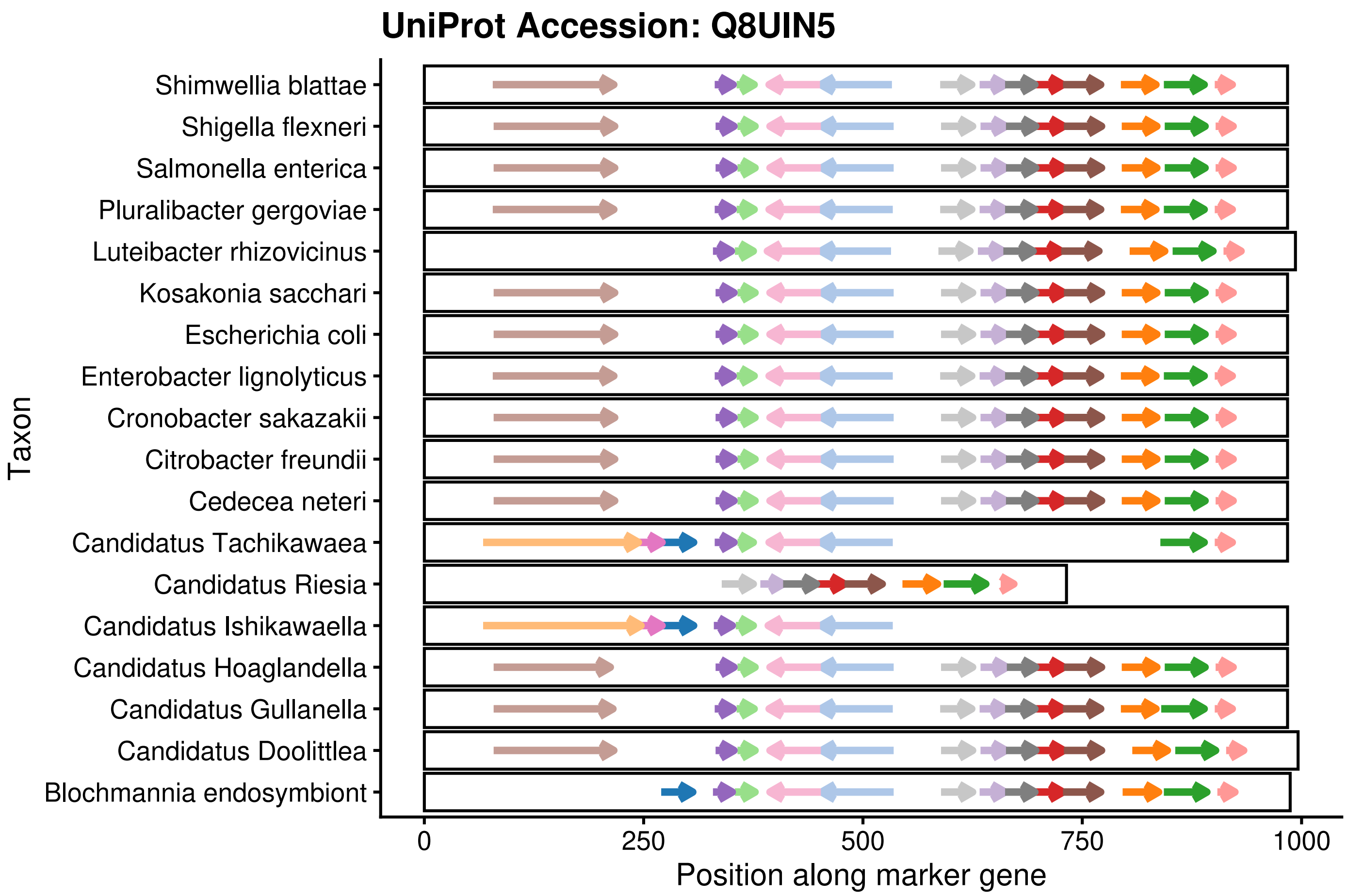
UniProt Accession: Q8UIN5
Taxon
Shimwellia blattae
Shigella flexneri
Salmonella enterica
Pluralibacter gergoviae
Luteibacter rhizovicinus
Kosakonia sacchari
Escherichia coli
Enterobacter lignolyticus
Cronobacter sakazakii
Citrobacter freundii
Cedecea neteri
Candidatus Tachikawaea
Candidatus Riesia
Candidatus Ishikawaella
Candidatus Hoaglandella
Candidatus Gullanella
Candidatus Doolittlea
Blochmannia endosymbiont
0
250
500
750
1000
Position along marker gene

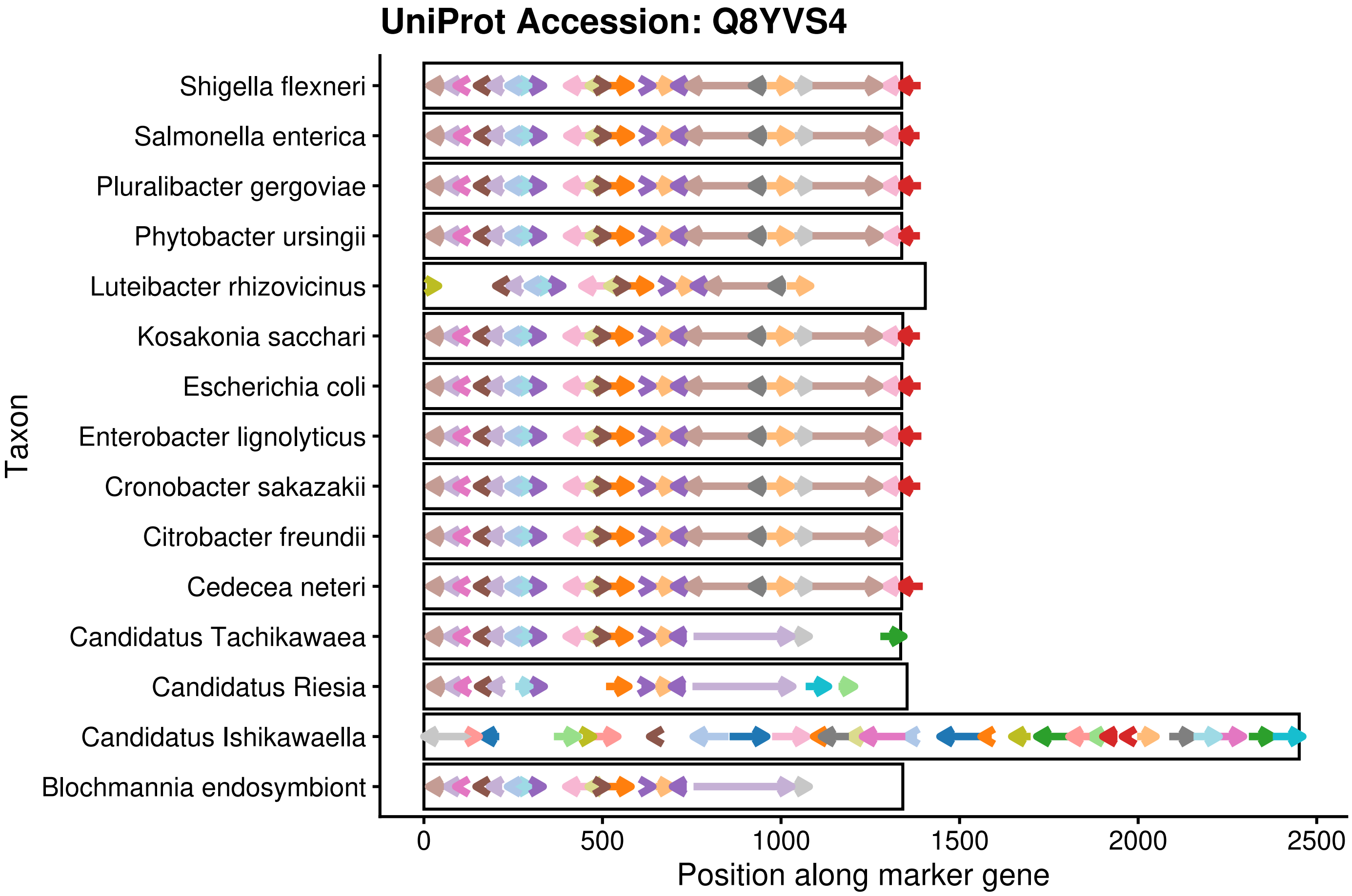

UniProt Accession: Q8YVS4
Shigella flexneri
Salmonella enterica
Pluralibacter gergoviae
Phytobacter ursingii
Luteibacter rhizovicinus
Kosakonia sacchari
Escherichia coli
Enterobacter lignolyticus
Cronobacter sakazakii
Citrobacter freundii
Cedecea neteri
Candidatus Tachikawaea
Candidatus Riesia
Candidatus Ishikawaella
Blochmannia endosymbiont
Taxon
0
500
1000
1500
2000
2500
Position along marker gene

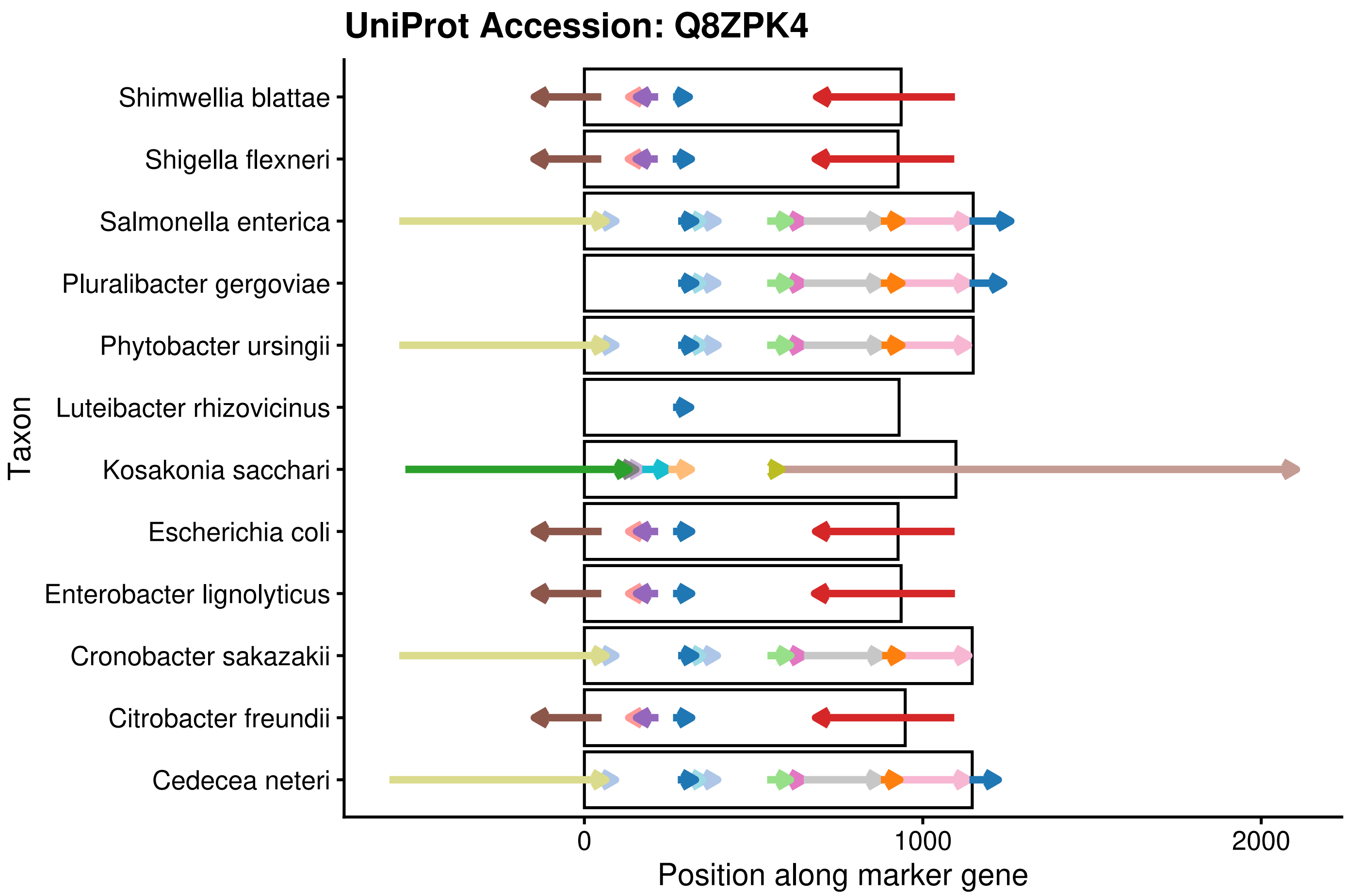
UniProt Accession: Q8ZPK4
Taxon
Shimwellia blattae
Shigella flexneri
Salmonella enterica
Pluralibacter gergoviae
Phytobacter ursingii
Luteibacter rhizovicinus
Kosakonia sacchari
Escherichia coli
Enterobacter lignolyticus
Cronobacter sakazakii
Citrobacter freundii
Cedecea neteri
0
1000
2000
Position along marker gene

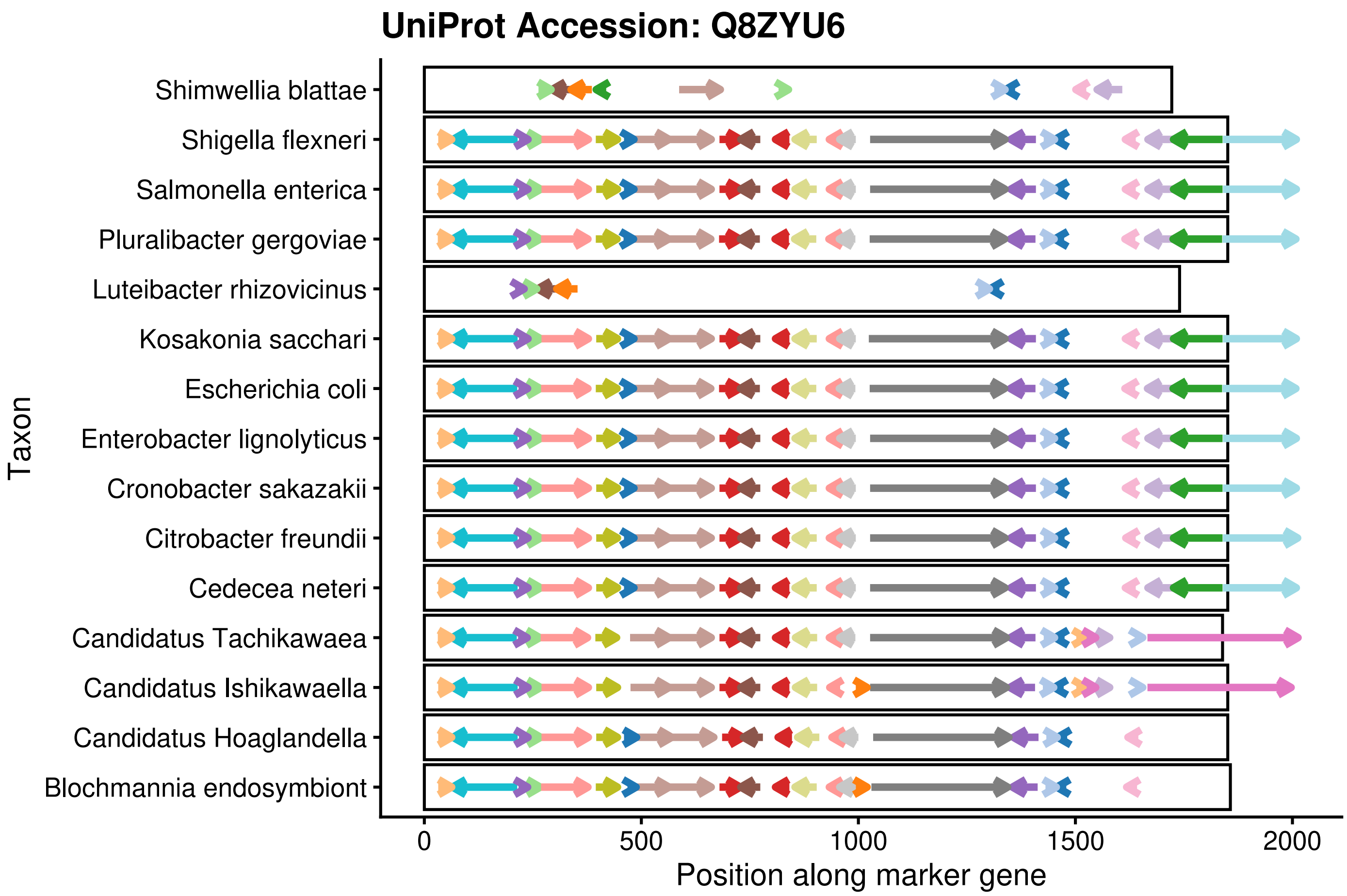
UniProt Accession: Q8ZYU6
Shimwellia blattae
Shigella flexneri
Salmonella enterica
Pluralibacter gergoviae
Luteibacter rhizovicinus
Kosakonia sacchari
Escherichia coli
Enterobacter lignolyticus
Cronobacter sakazakii
Citrobacter freundii
Cedecea neteri
Candidatus Tachikawaea
Candidatus Ishikawaella
Candidatus Hoaglandella
Blochmannia endosymbiont
Taxon
0
500
1000
1500
2000
Position along marker gene

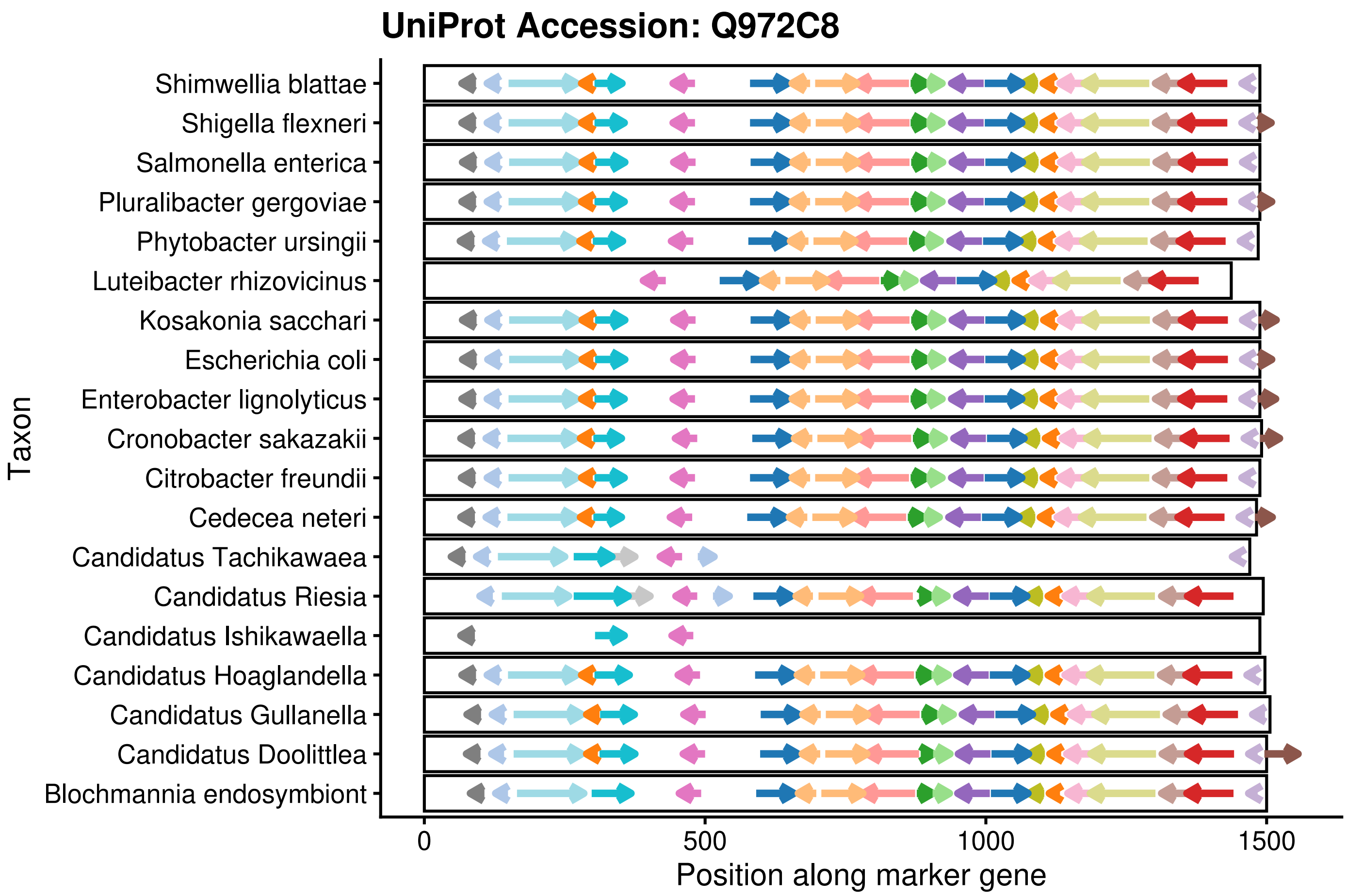
UniProt Accession: Q972C8
Shimwellia blattae
Shigella flexneri
Salmonella enterica
Pluralibacter gergoviae
Phytobacter ursingii
Luteibacter rhizovicinus
Kosakonia sacchari
Escherichia coli
Enterobacter lignolyticus
Cronobacter sakazakii
Citrobacter freundii
Cedecea neteri
Candidatus Tachikawaea
Candidatus Riesia
Candidatus Ishikawaella
Candidatus Hoaglandella
Candidatus Gullanella
Candidatus Doolittlea
Blochmannia endosymbiont
Taxon
0
500
1000
1500
Position along marker gene

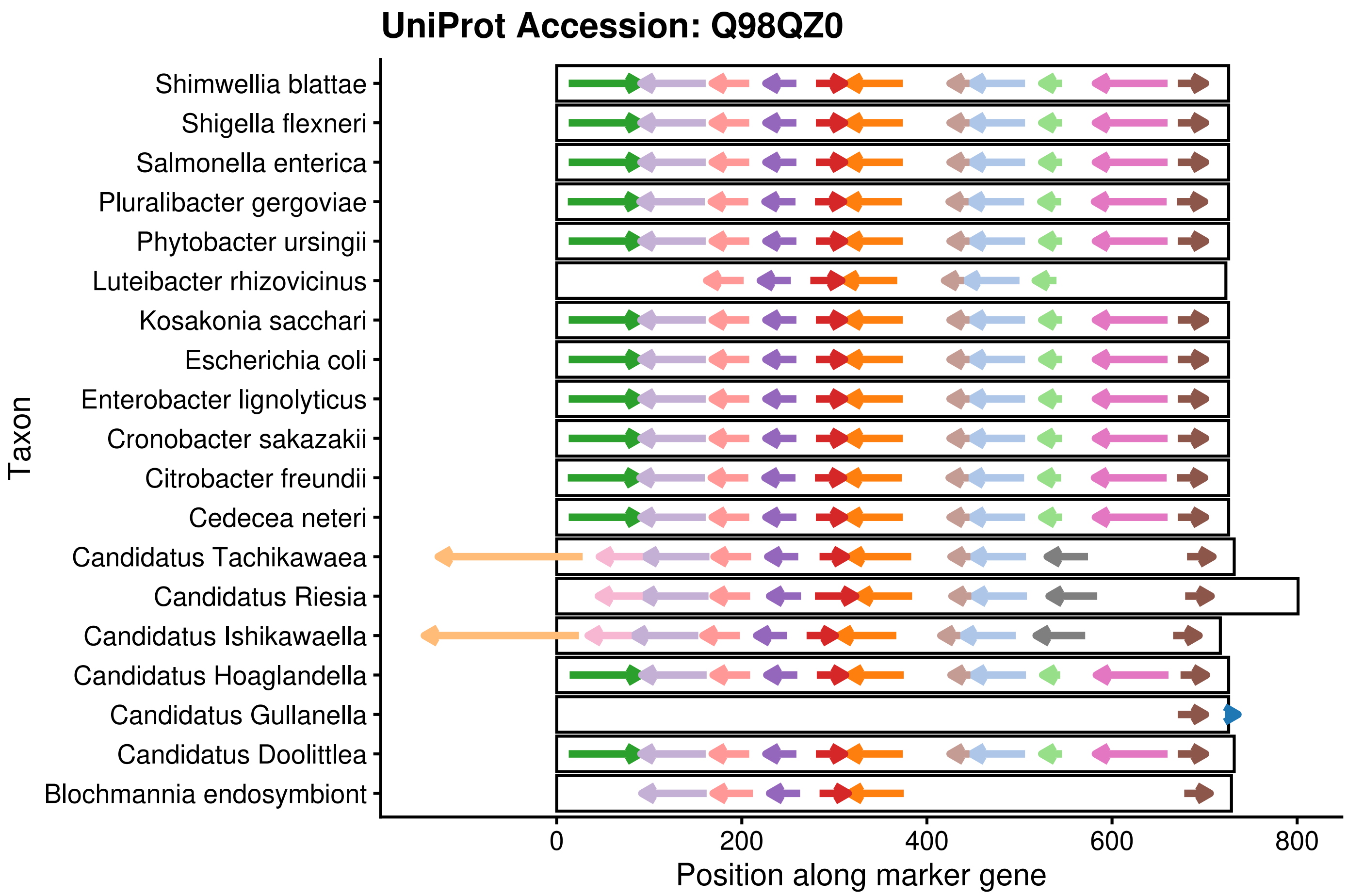
UniProt Accession: Q98QZ0
Shimwellia blattae
Shigella flexneri
Salmonella enterica
Pluralibacter gergoviae
Phytobacter ursingii
Luteibacter rhizovicinus
Kosakonia sacchari
Escherichia coli
Enterobacter lignolyticus
Cronobacter sakazakii
Citrobacter freundii
Cedecea neteri
Candidatus Tachikawaea
Candidatus Riesia
Candidatus Ishikawaella
Candidatus Hoaglandella
Candidatus Gullanella
Candidatus Doolittlea
Blochmannia endosymbiont
Taxon
0
200
400
600
800
Position along marker gene

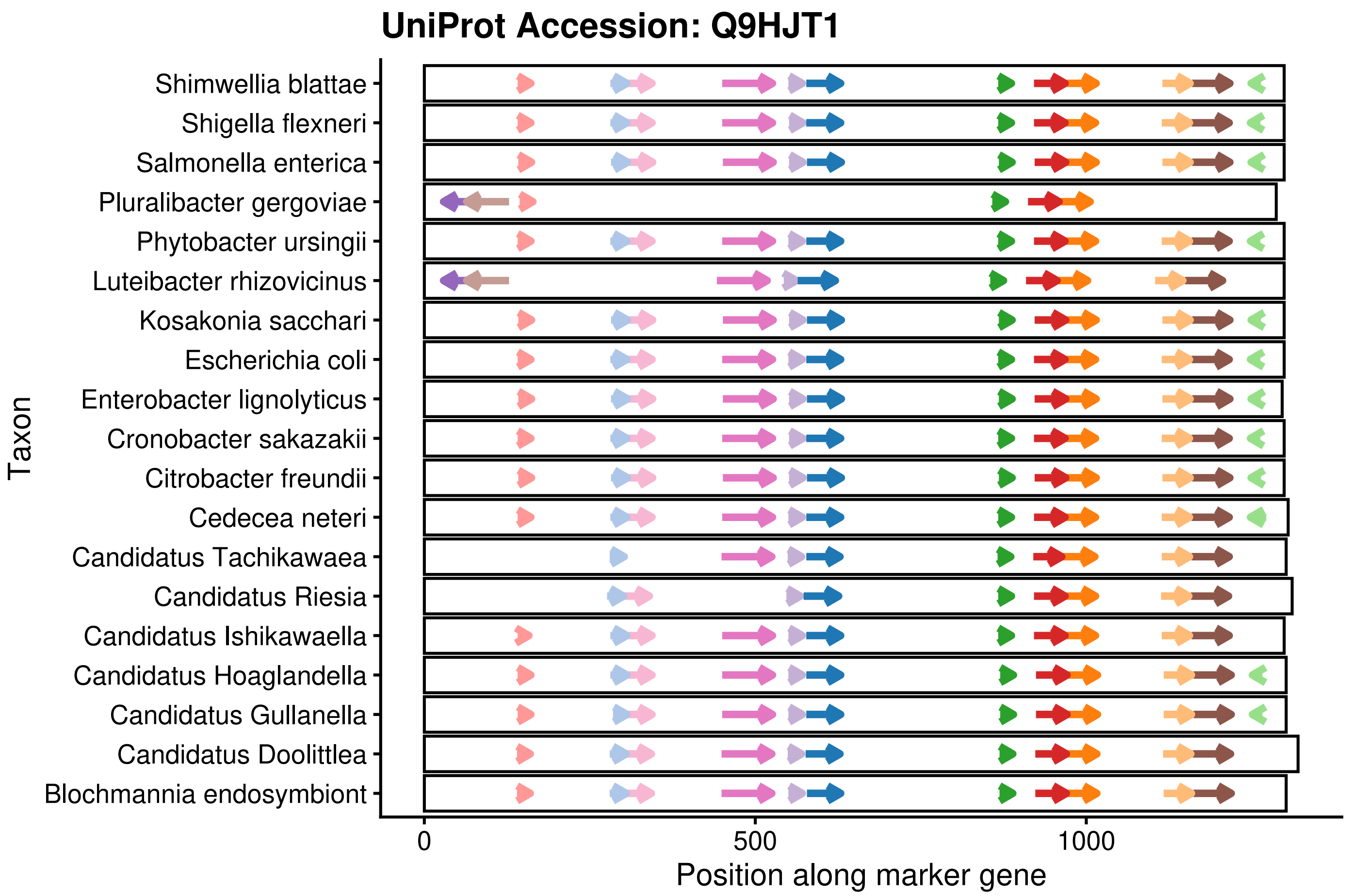

UniProt Accession: Q9HJT1
Taxon
Shimwellia blattae
Shigella flexneri
Salmonella enterica
Pluralibacter gergoviae
Phytobacter ursingii
Luteibacter rhizovicinus
Kosakonia sacchari
Escherichia coli
Enterobacter lignolyticus
Cronobacter sakazakii
Citrobacter freundii
Cedecea neteri
Candidatus Tachikawaea
Candidatus Riesia
Candidatus Ishikawaella
Candidatus Hoaglandella
Candidatus Gullanella
Candidatus Doolittlea
Blochmannia endosymbiont
0
500
1000
Position along marker gene

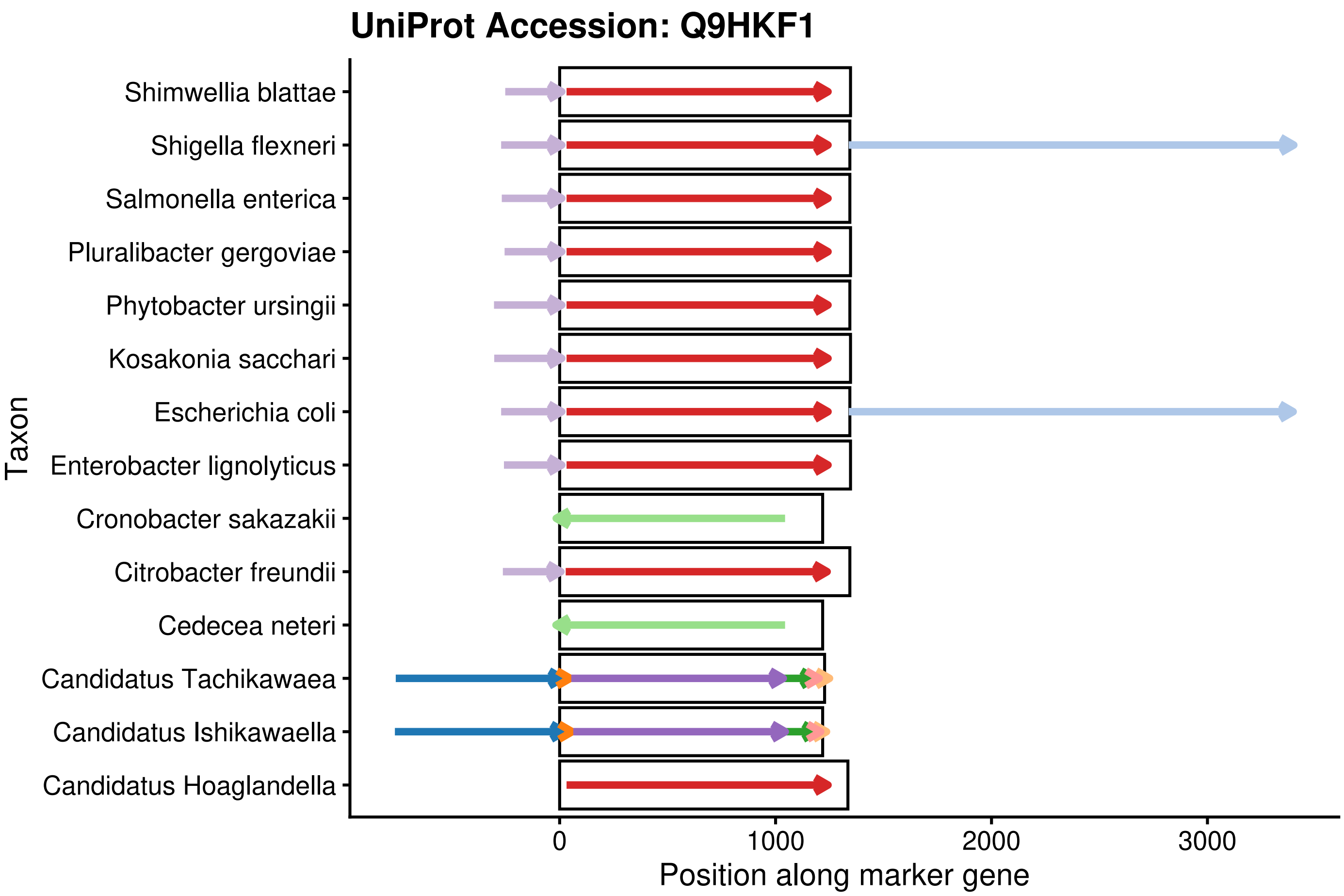

UniProt Accession: Q9HKF1
Taxon
Shimwellia blattae
Shigella flexneri
Salmonella enterica
Pluralibacter gergoviae
Phytobacter ursingii
Kosakonia sacchari
Escherichia coli
Enterobacter lignolyticus
Cronobacter sakazakii
Citrobacter freundii
Cedecea neteri
Candidatus Tachikawaea
Candidatus Ishikawaella
Candidatus Hoaglandella
0
1000
2000
3000
Position along marker gene

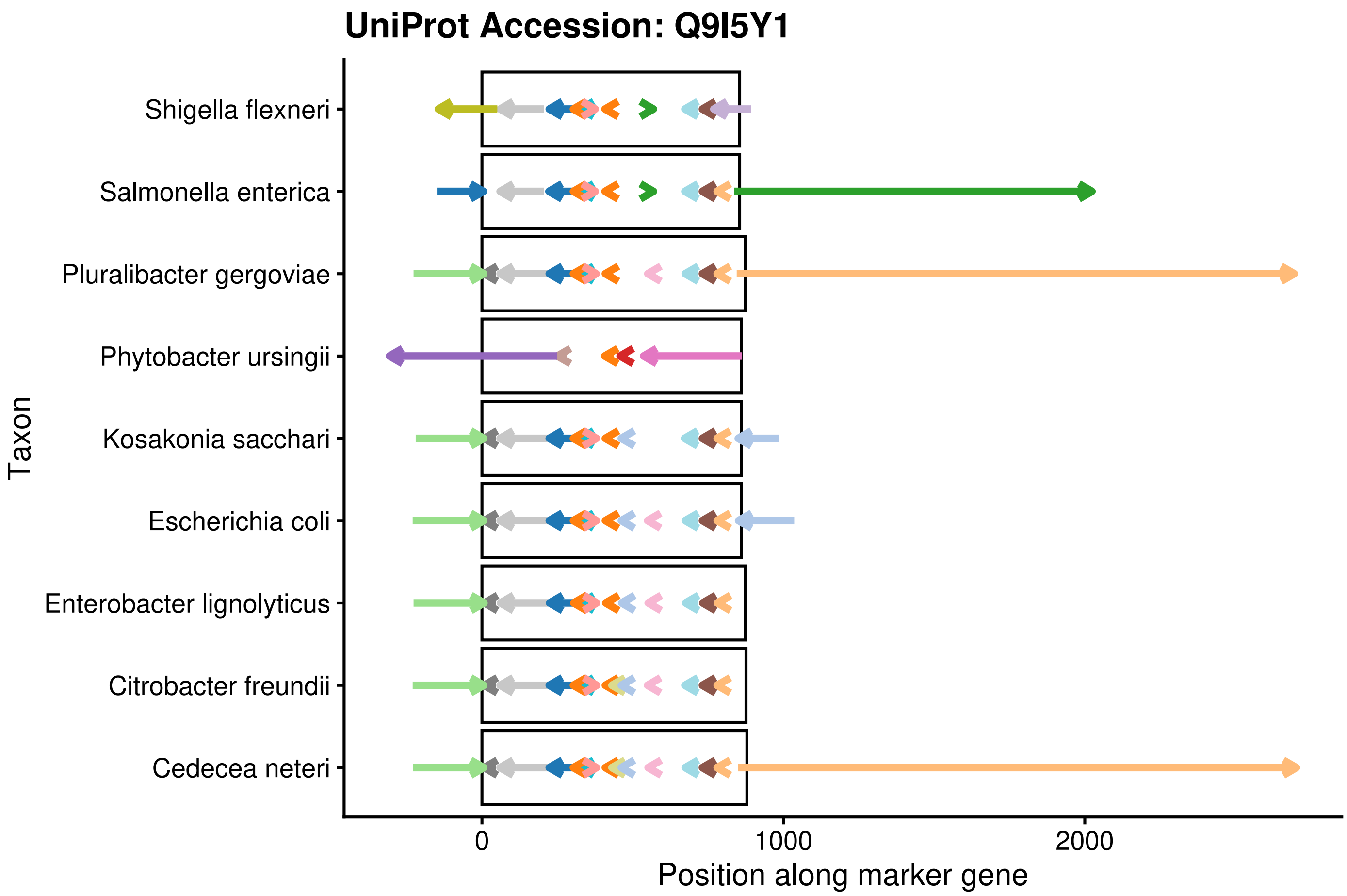

UniProt Accession: Q9I5Y1
Shigella flexneri
Salmonella enterica
Pluralibacter gergoviae
Phytobacter ursingii
Kosakonia sacchari
Escherichia coli
Enterobacter lignolyticus
Citrobacter freundii
Cedecea neteri
Taxon
0
1000
2000
Position along marker gene

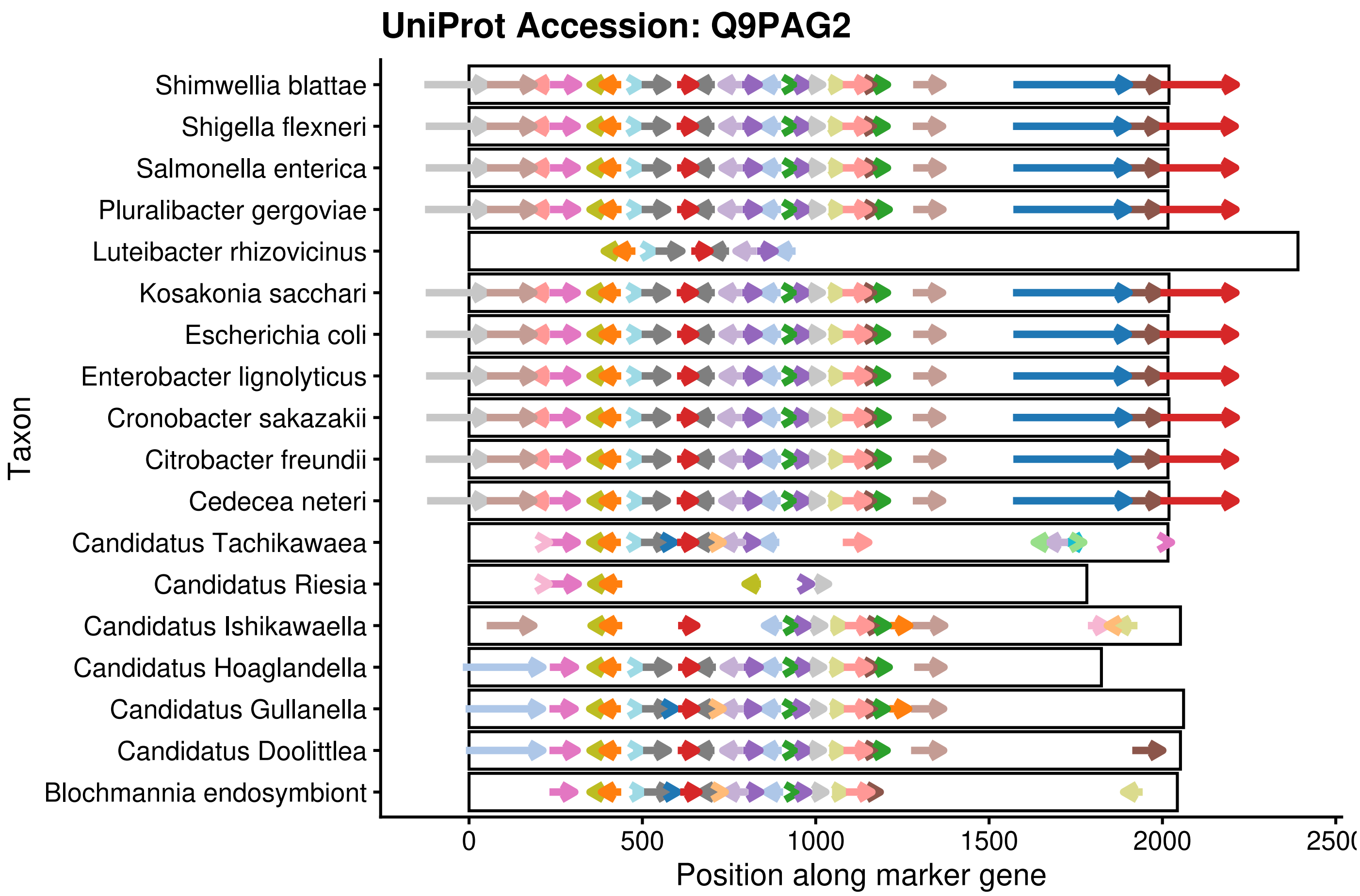
UniProt Accession: Q9PAG2
Taxon
Shimwellia blattae
Shigella flexneri
Salmonella enterica
Pluralibacter gergoviae
Luteibacter rhizovicinus
Kosakonia sacchari
Escherichia coli
Enterobacter lignolyticus
Cronobacter sakazakii
Citrobacter freundii
Cedecea neteri
Candidatus Tachikawaea
Candidatus Riesia
Candidatus Ishikawaella
Candidatus Hoaglandella
Candidatus Gullanella
Candidatus Doolittlea
Blochmannia endosymbiont
0
500
1000
1500
2000
Position along marker gene

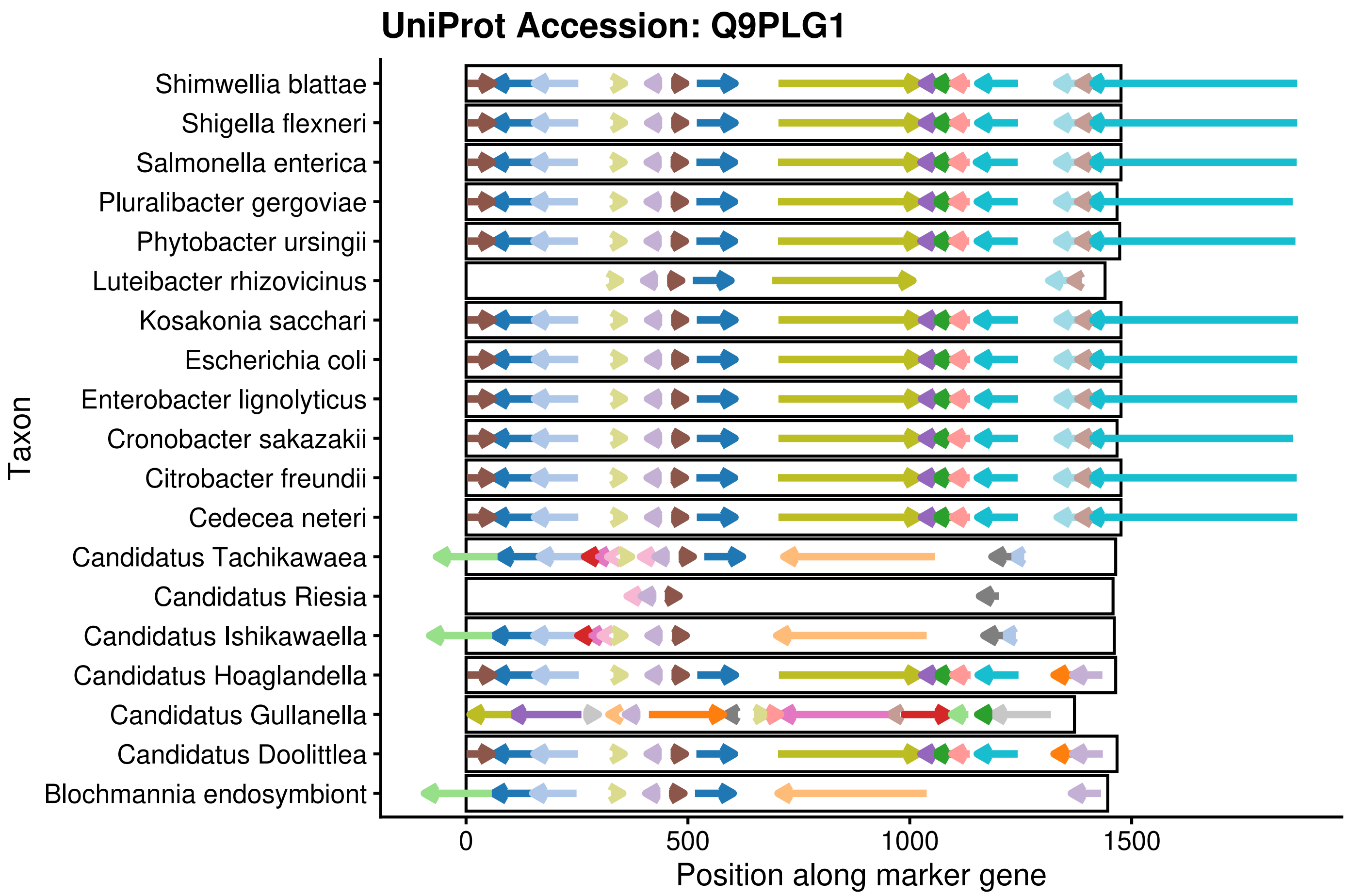
UniProt Accession: Q9PLG1
Shimwellia blattae
Shigella flexneri
Salmonella enterica
Pluralibacter gergoviae
Phytobacter ursingii
Luteibacter rhizovicinus
Kosakonia sacchari
Escherichia coli
Enterobacter lignolyticus
Cronobacter sakazakii
Citrobacter freundii
Cedecea neteri
Candidatus Tachikawaea
Candidatus Riesia
Candidatus Ishikawaella
Candidatus Hoaglandella
Candidatus Gullanella
Candidatus Doolittlea
Blochmannia endosymbiont
Taxon
0
500
1000
1500
Position along marker gene

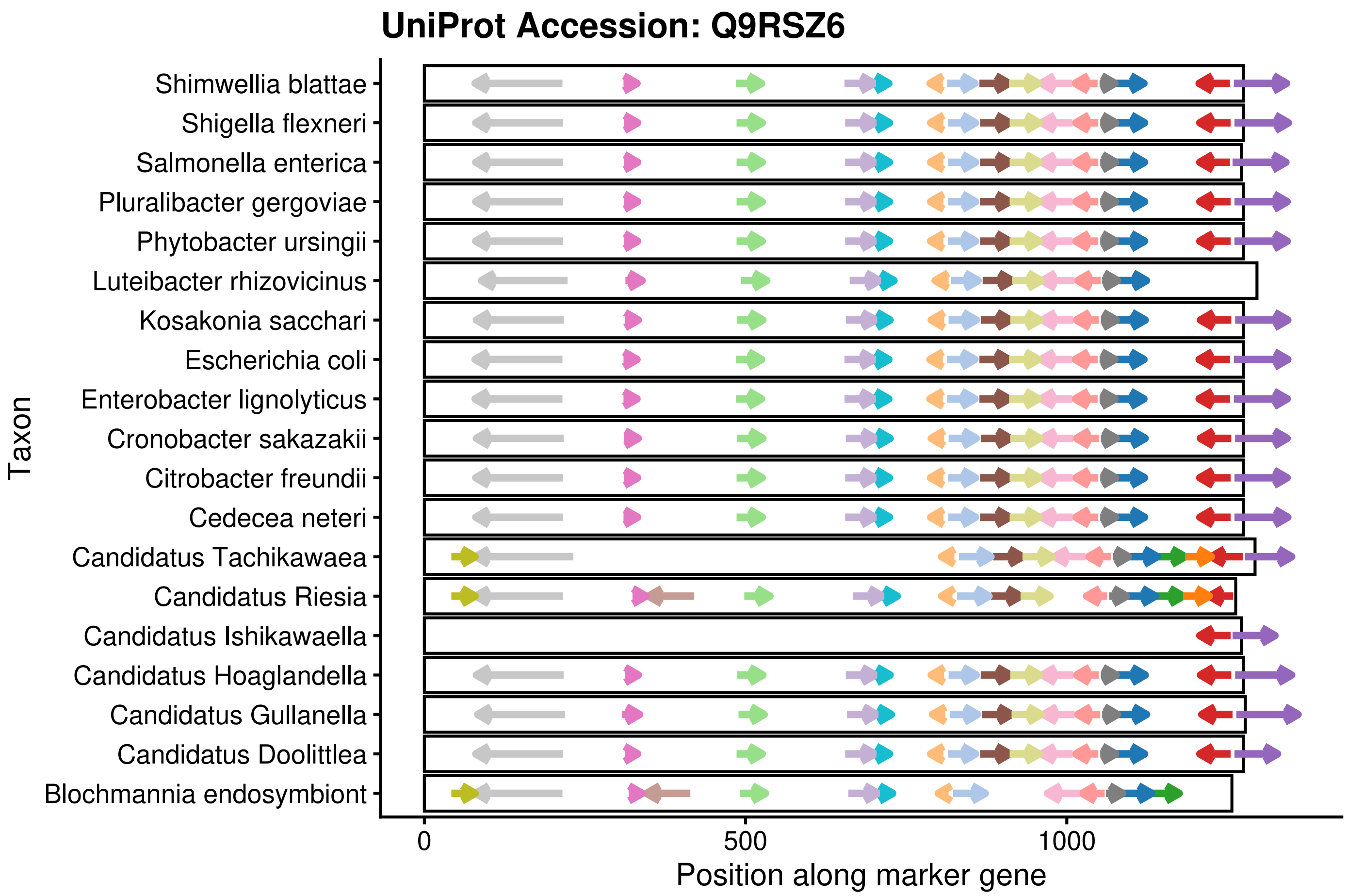
UniProt Accession: Q9RSZ6
Taxon
Shimwellia blattae
Shigella flexneri
Salmonella enterica
Pluralibacter gergoviae
Phytobacter ursingii
Luteibacter rhizovicinus
Kosakonia sacchari
Escherichia coli
Enterobacter lignolyticus
Cronobacter sakazakii
Citrobacter freundii
Cedecea neteri
Candidatus Tachikawaea
Candidatus Riesia
Candidatus Ishikawaella
Candidatus Hoaglandella
Candidatus Gullanella
Candidatus Doolittlea
Blochmannia endosymbiont
0
500
1000
Position along marker gene

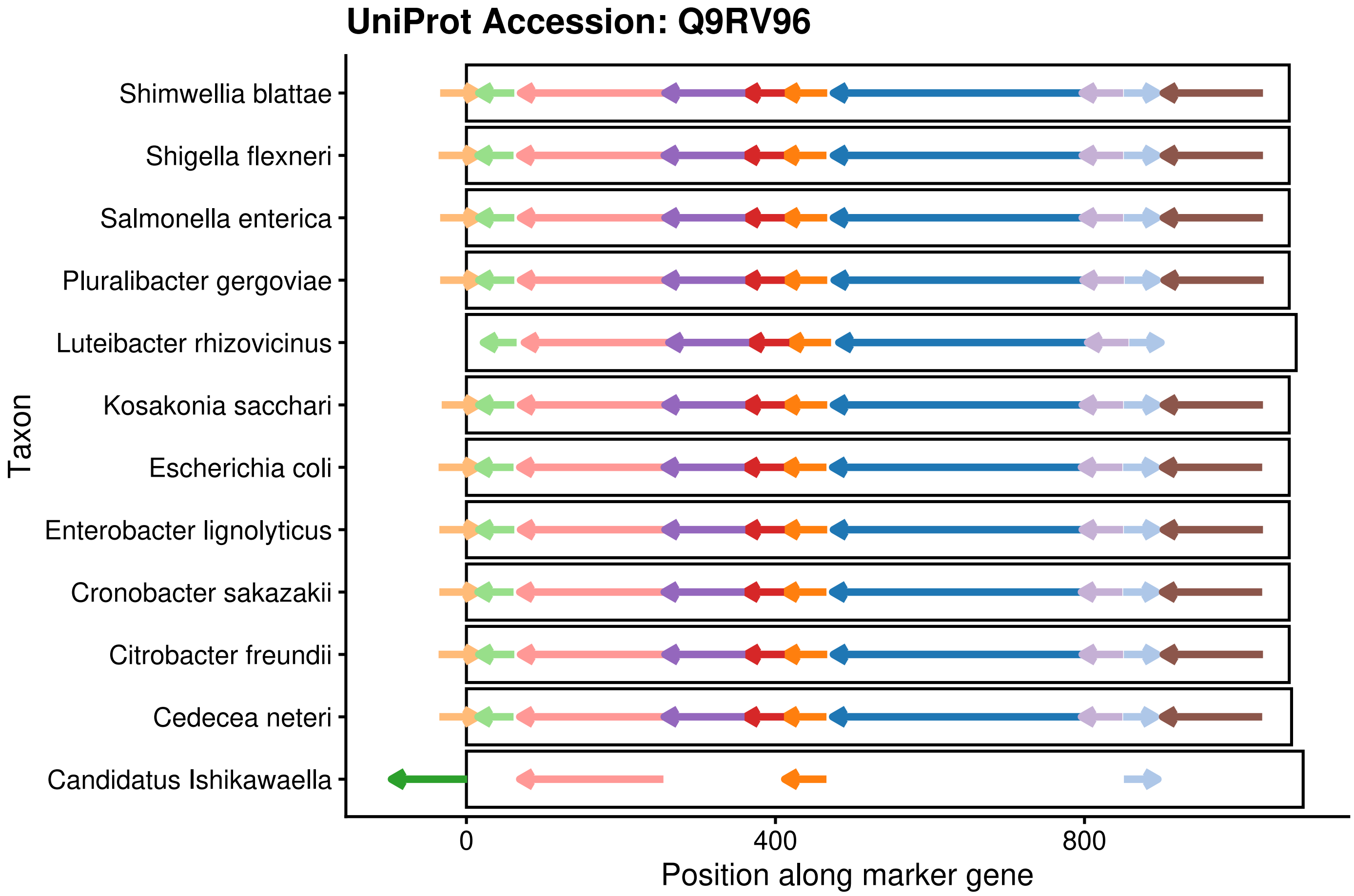
UniProt Accession: Q9RV96
Shimwellia blattae
Shigella flexneri
Salmonella enterica
Pluralibacter gergoviae
Luteibacter rhizovicinus
Kosakonia sacchari
Escherichia coli
Enterobacter lignolyticus
Cronobacter sakazakii
Citrobacter freundii
Cedecea neteri
Candidatus Ishikawaella
Taxon
0
400
800
Position along marker gene

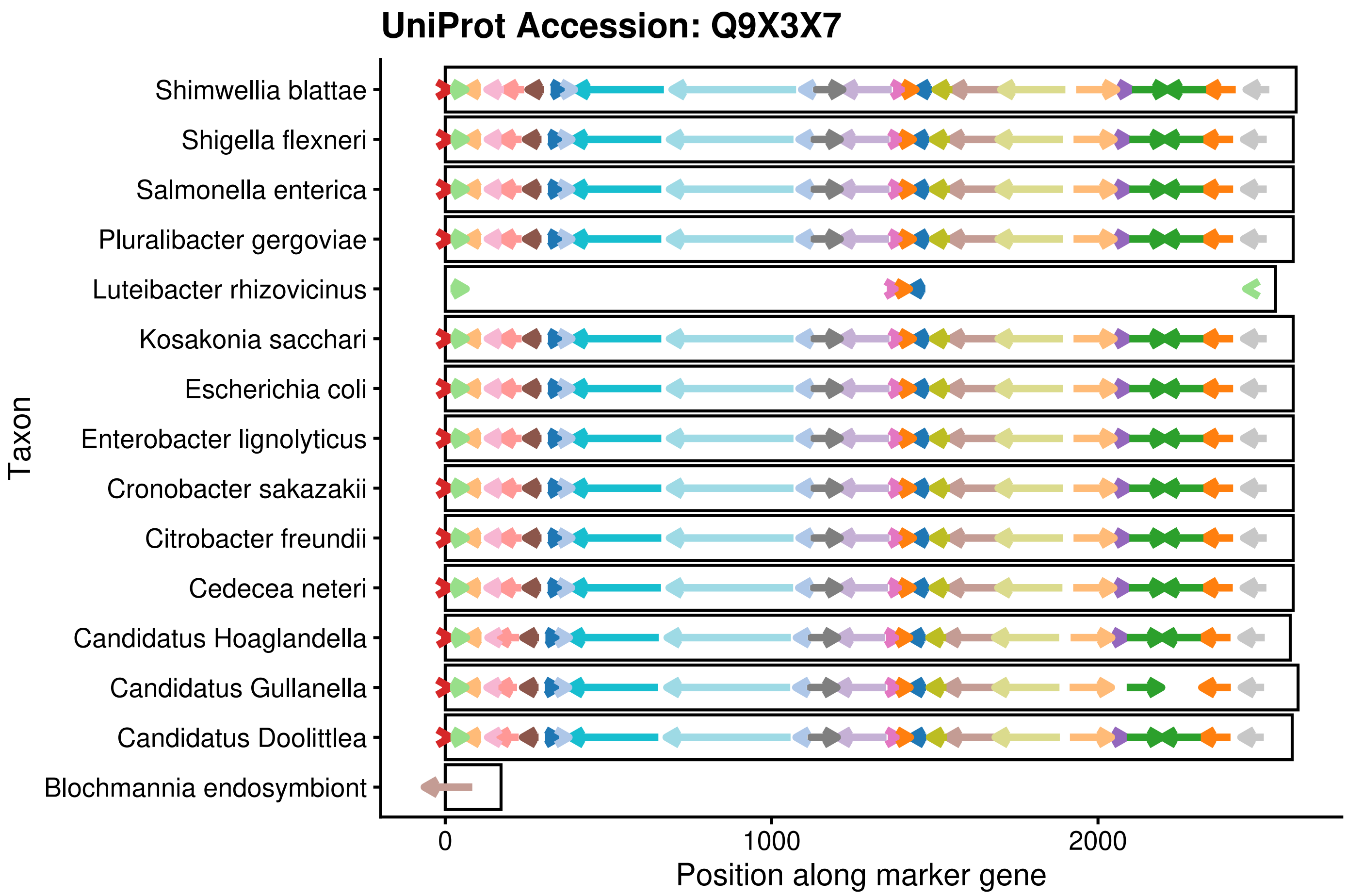
UniProt Accession: Q9X3X7
Taxon
Shimwellia blattae
Shigella flexneri
Salmonella enterica
Pluralibacter gergoviae
Luteibacter rhizovicinus
Kosakonia sacchari
Escherichia coli
Enterobacter lignolyticus
Cronobacter sakazakii
Citrobacter freundii
Cedecea neteri
Candidatus Hoaglandella
Candidatus Gullanella
Candidatus Doolittlea
Blochmannia endosymbiont
0
1000
2000
Position along marker gene

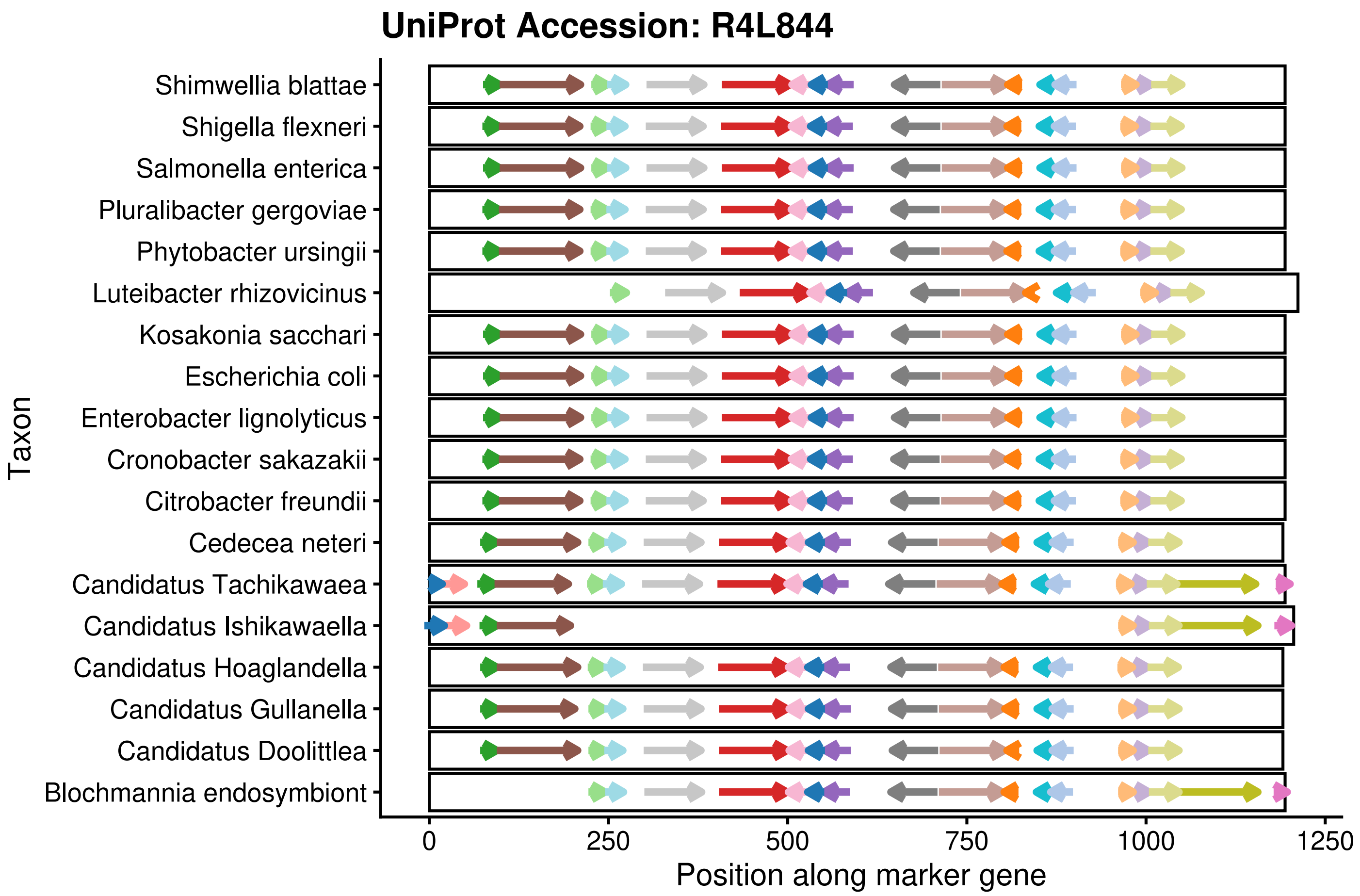
UniProt Accession: R4L844
Shimwellia blattae
Shigella flexneri
Salmonella enterica
Pluralibacter gergoviae
Phytobacter ursingii
Luteibacter rhizovicinus
Kosakonia sacchari
Escherichia coli
Enterobacter lignolyticus
Cronobacter sakazakii
Citrobacter freundii
Cedecea neteri
Candidatus Tachikawaea
Candidatus Ishikawaella
Candidatus Hoaglandella
Candidatus Gullanella
Candidatus Doolittlea
Blochmannia endosymbiont
Taxon
0
250
500
750
1000
1250
Position along marker gene

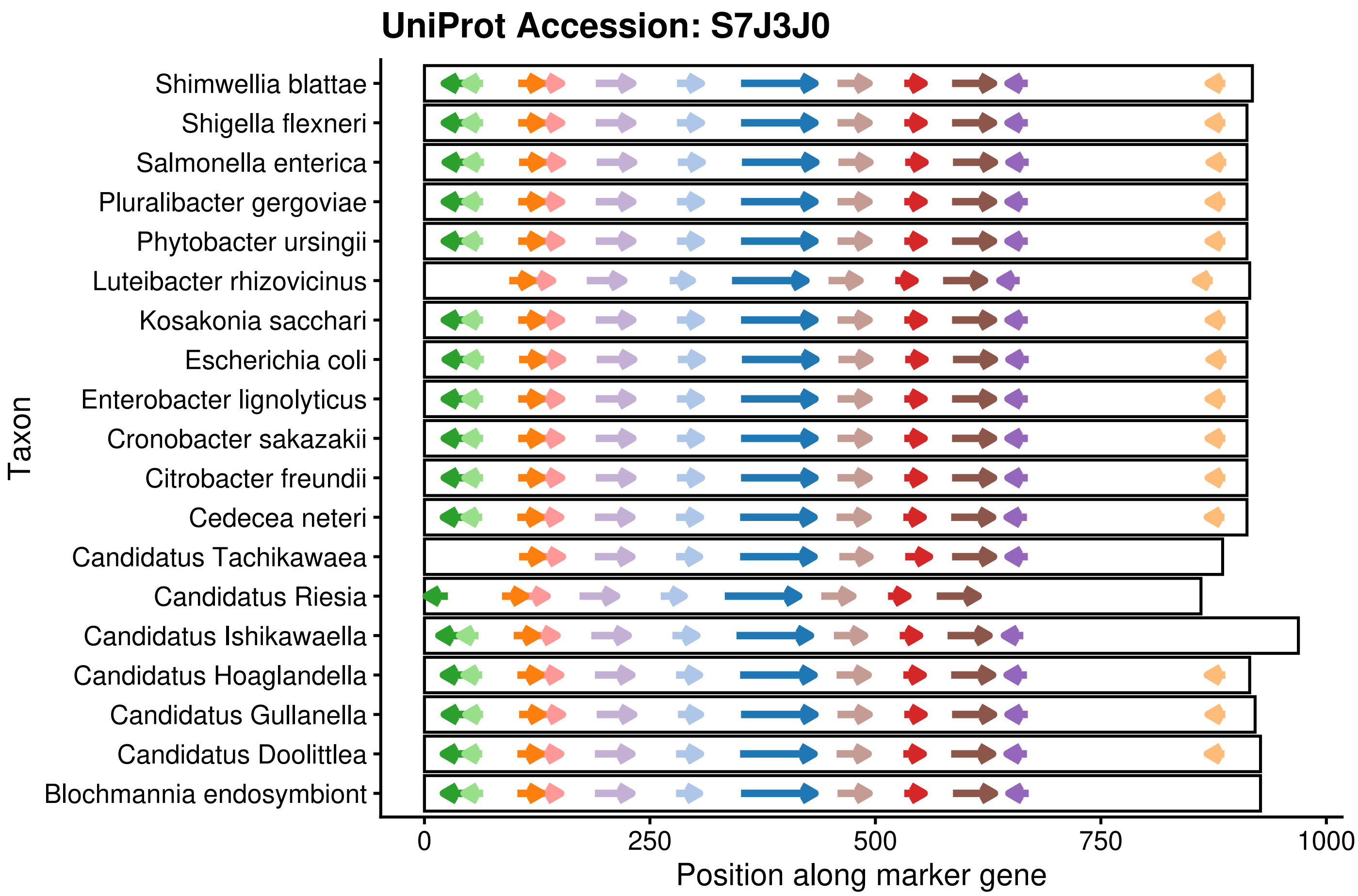
UniProt Accession: S7J3J0
Taxon
Shimwellia blattae
Shigella flexneri
Salmonella enterica
Pluralibacter gergoviae
Phytobacter ursingii
Luteibacter rhizovicinus
Kosakonia sacchari
Escherichia coli
Enterobacter lignolyticus
Cronobacter sakazakii
Citrobacter freundii
Cedecea neteri
Candidatus Tachikawaea
Candidatus Riesia
Candidatus Ishikawaella
Candidatus Hoaglandella
Candidatus Gullanella
Candidatus Doolittlea
Blochmannia endosymbiont
0
250
500
750
1000
Position along marker gene

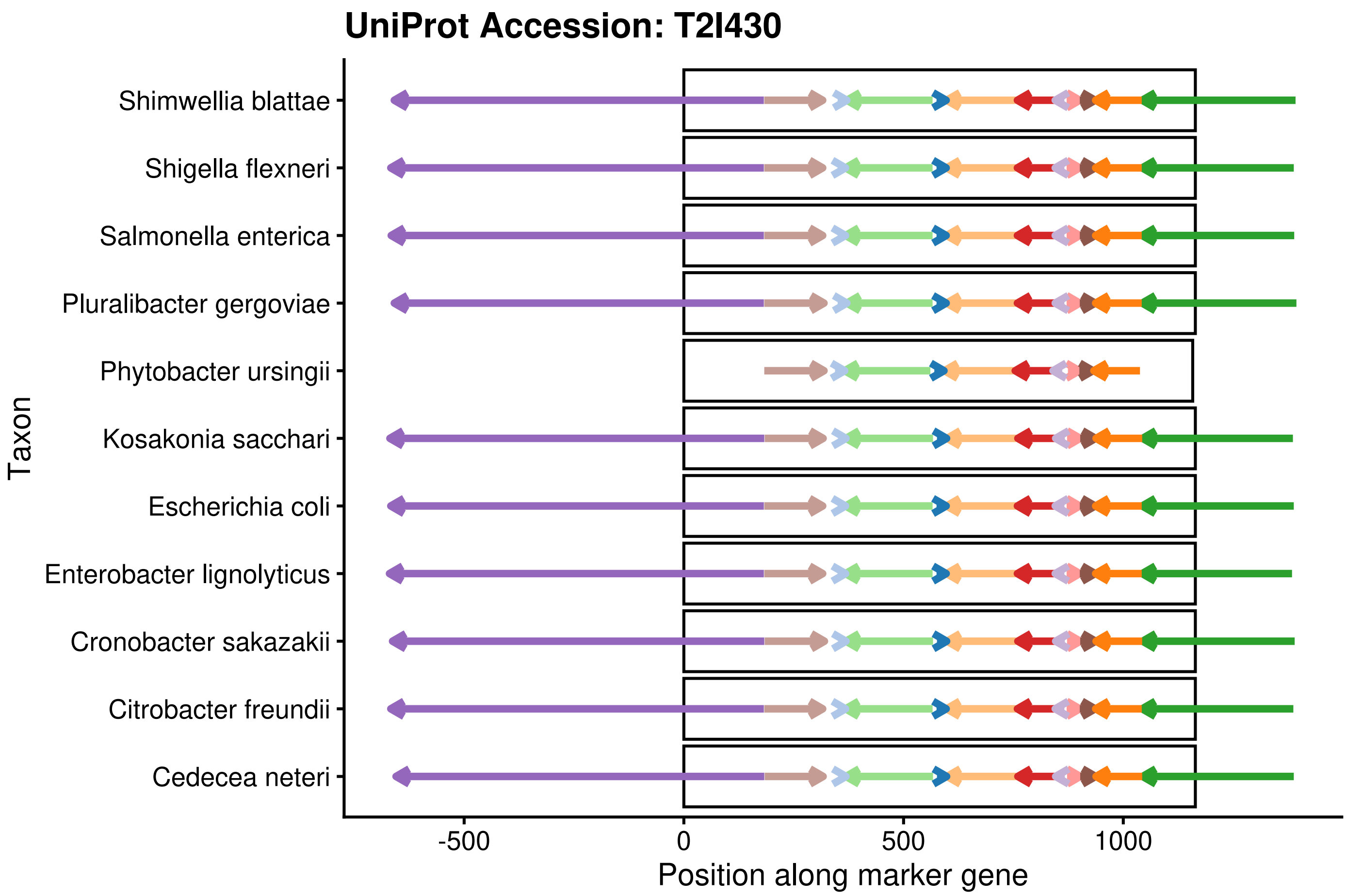
UniProt Accession: T2I430
Taxon
Shimwellia blattae
Shigella flexneri
Salmonella enterica
Pluralibacter gergoviae
Phytobacter ursingii
Kosakonia sacchari
Escherichia coli
Enterobacter lignolyticus
Cronobacter sakazakii
Citrobacter freundii
Cedecea neteri
-500
0
500
1000
Position along marker gene

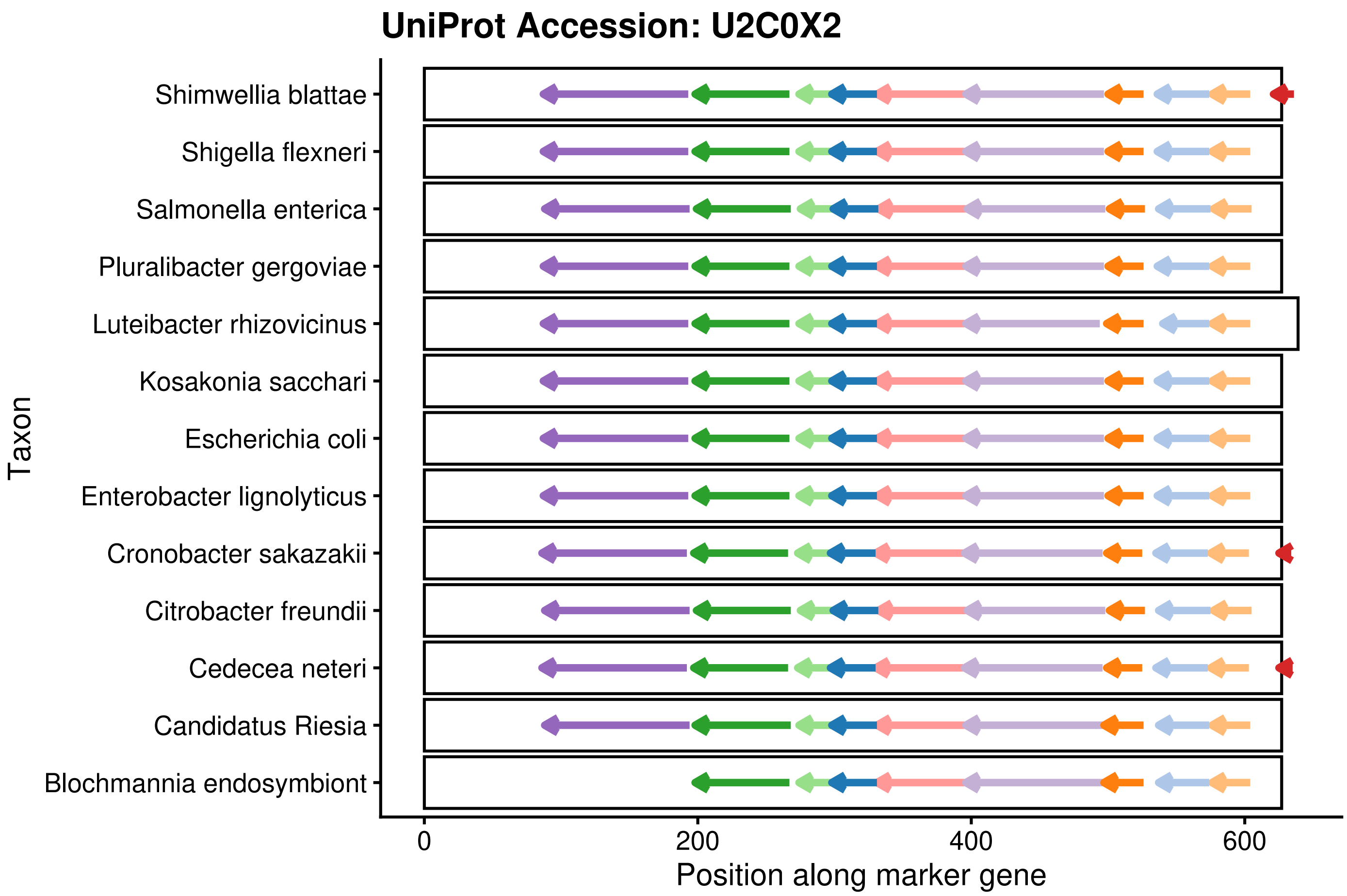
UniProt Accession: U2C0X2
Taxon
Shimwellia blattae
Shigella flexneri
Salmonella enterica
Pluralibacter gergoviae
Luteibacter rhizovicinus
Kosakonia sacchari
Escherichia coli
Enterobacter lignolyticus
Cronobacter sakazakii
Citrobacter freundii
Cedecea neteri
Candidatus Riesia
Blochmannia endosymbiont
0
200
400
600
Position along marker gene

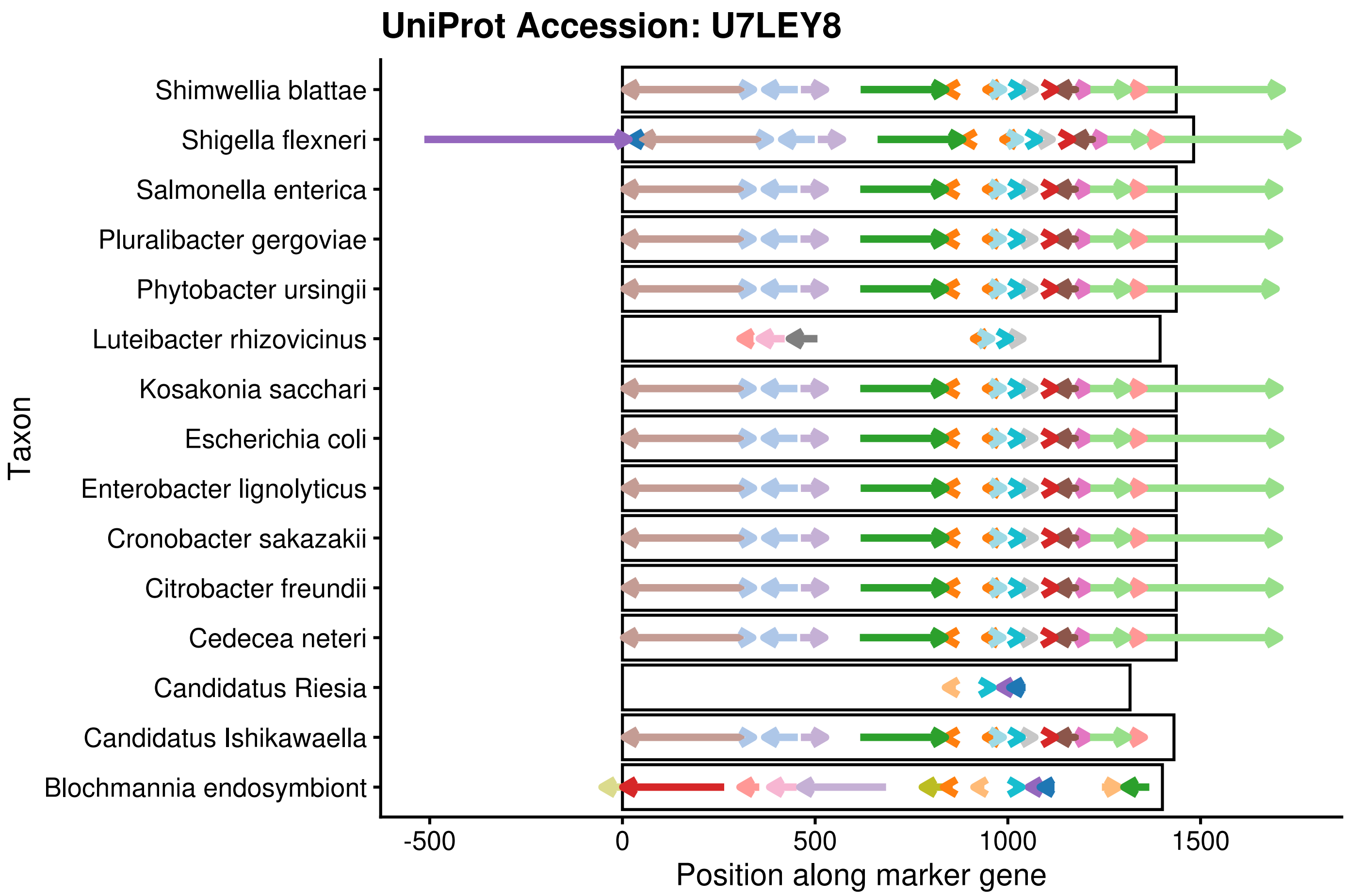

UniProt Accession: U7LEY8
Shimwellia blattae
Shigella flexneri
Salmonella enterica
Pluralibacter gergoviae
Phytobacter ursingii
Luteibacter rhizovicinus
Kosakonia sacchari
Escherichia coli
Enterobacter lignolyticus
Cronobacter sakazakii
Citrobacter freundii
Cedecea neteri
Candidatus Riesia
Candidatus Ishikawaella
Blochmannia endosymbiont
Taxon
-500
0
500
1000
1500
Position along marker gene

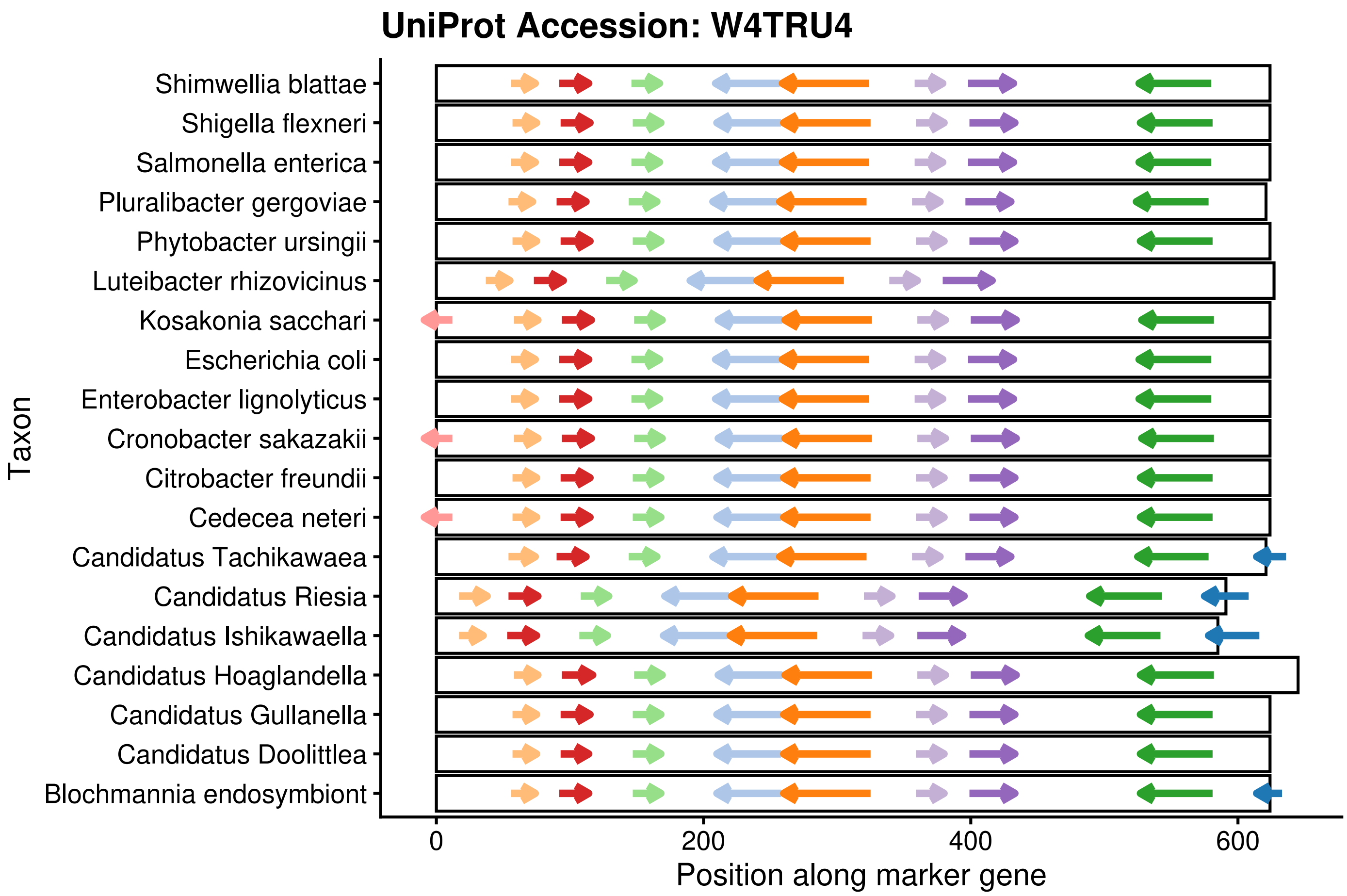
UniProt Accession: W4TRU4
Shimwellia blattae
Shigella flexneri
Salmonella enterica
Pluralibacter gergoviae
Phytobacter ursingii
Luteibacter rhizovicinus
Kosakonia sacchari
Escherichia coli
Enterobacter lignolyticus
Cronobacter sakazakii
Citrobacter freundii
Cedecea neteri
Candidatus Tachikawaea
Candidatus Riesia
Candidatus Ishikawaella
Candidatus Hoaglandella
Candidatus Gullanella
Candidatus Doolittlea
Blochmannia endosymbiont
Taxon
0
200
400
600
Position along marker gene

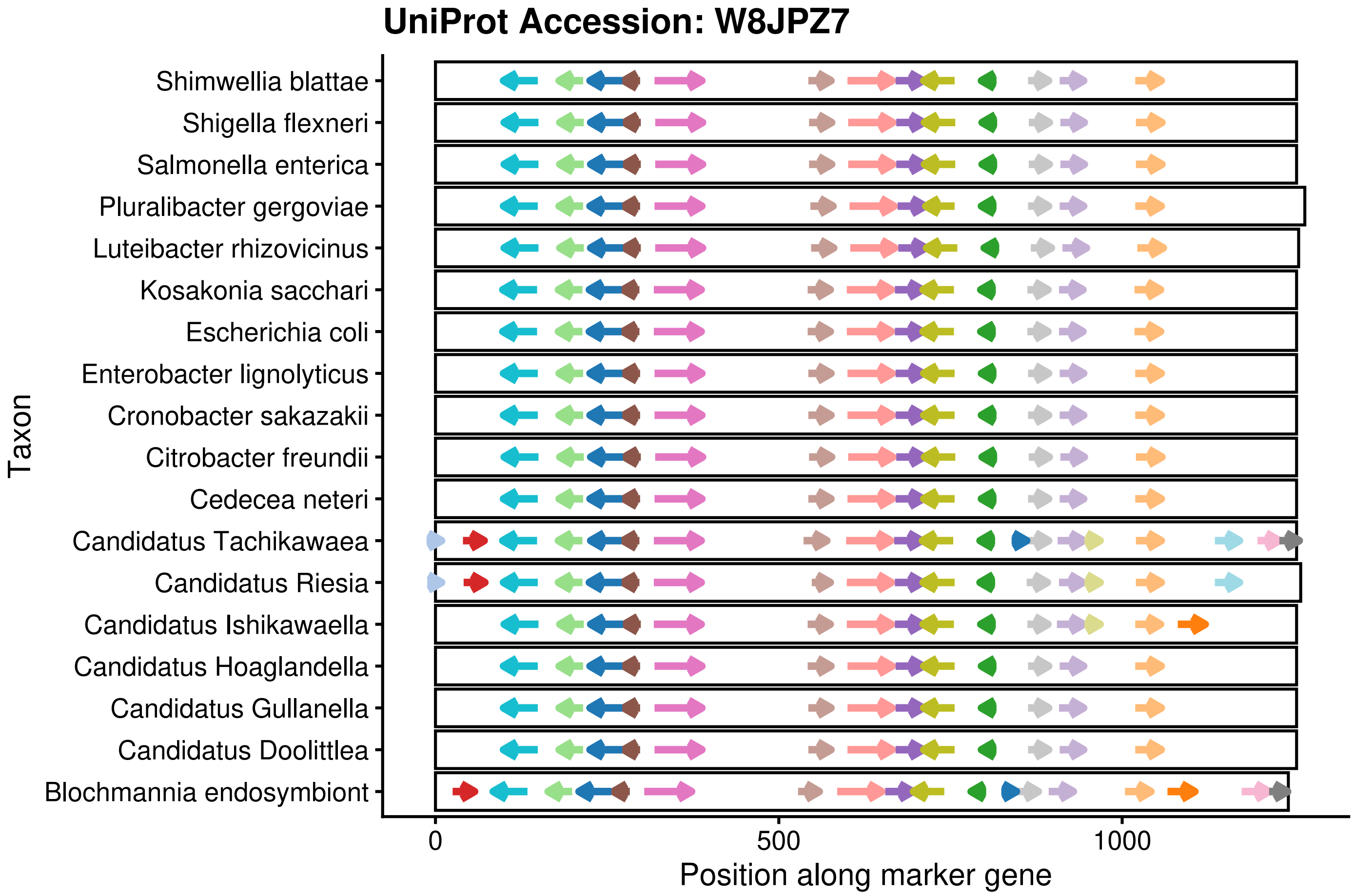
UniProt Accession: W8JPZ7
Shimwellia blattae
Shigella flexneri
Salmonella enterica
Pluralibacter gergoviae
Luteibacter rhizovicinus
Kosakonia sacchari
Escherichia coli
Enterobacter lignolyticus
Cronobacter sakazakii
Citrobacter freundii
Cedecea neteri
Candidatus Tachikawaea
Candidatus Riesia
Candidatus Ishikawaella
Candidatus Hoaglandella
Candidatus Gullanella
Candidatus Doolittlea
Blochmannia endosymbiont
Taxon
0
500
1000
Position along marker gene

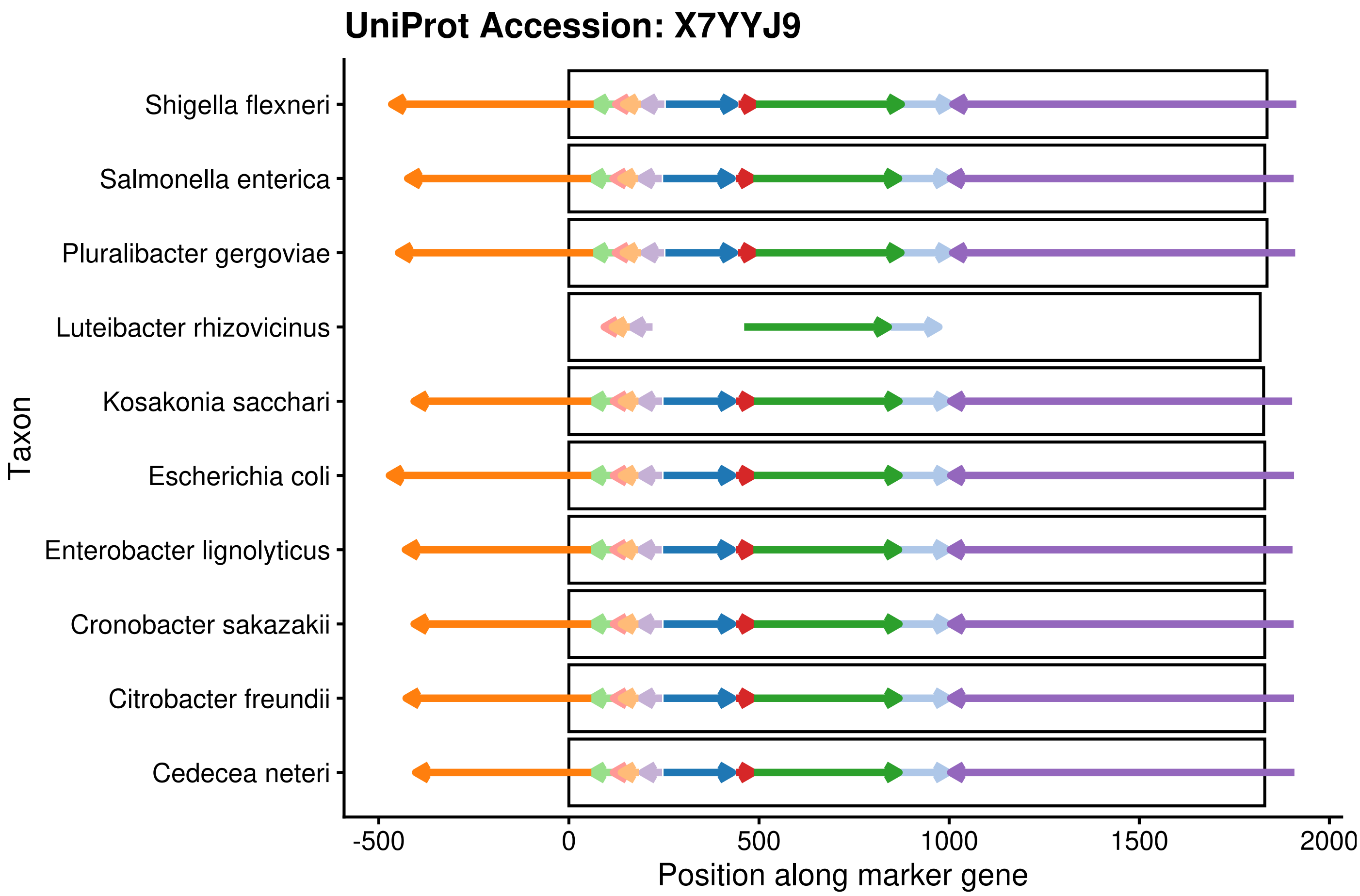
UniProt Accession: X7YYJ9
Taxon
Shigella flexneri
Salmonella enterica
Pluralibacter gergoviae
Luteibacter rhizovicinus
Kosakonia sacchari
Escherichia coli
Enterobacter lignolyticus
Cronobacter sakazakii
Citrobacter freundii
Cedecea neteri
-500
0
500
1000
1500
2000
Position along marker gene
