# Supplementary material for: Annotation-free delineation of prokaryotic homology groups
Source: PLoS Comput Biol. 2022 Jun 8;18(6):e1010216. doi: 10.1371/journal.pcbi.1010216 (PMC9212150; doi:10.1371/journal.pcbi.1010216)
Supplement: Attachment — Submitted filename: MHG_Reviewer_Comments.docx [file pcbi.1010216.s002.docx]

Reviewer #1: Yin et al. introduced an algorithm for identifying maximal homologous groups of sequences (MHGs) from prokaryotic genomes. The method starts with pairwise alignments among genomes, and piles up homologous segments using a network approach to generate longer MHGs. They suggested that MHGs are advantageous for phylogenetic inference over traditional marker genes which are prone to the impact of horizontal gene transfer and recombination events. The MHG method they developed does not rely on the annotation of protein-coding genes. They were shown to cover higher proportion of the genomes and be less biased in terms of function. The authors applied this new method to 19 Enterobacteriaceae species previously analyzed in a marker gene-based phylogenomic study. They found that MHGs granted higher accuracy and robustness compared with markers. Collectively, they recommended MHGs for phylogenomic studies of prokaryotes.

This is a very interesting method and direction of phylogenetic research. It provides new evidence for a plausible solution to the philosophic question of the unit of evolution. It comes with a software tool, in addition to an algorithm, which can be used by the research community. This work is potentially impactful to the field of phylogenetics. The authors concluded that it provides a “starting point” for future researches along the line, which I largely agree and appreciate. I will be amazed to see how gene-free phylogenetics will expand in the future, and I believe that the current work sets an important milestone for it.

With that being said, I think the current work is still quite primitive in the breath and depth of the analysis. Admittedly, phylogenetics is a large question, and it shouldn’t be a requirement for researchers with novel thoughts to take care of all plausible questions before publishing their result. I still would like to point out some of the questions which I think are most relevant, important and “good-to-have”, and hope that the authors can address these without substantial amount of additional efforts.

We thank this reviewer for their accurate summary above, and the constructive comments and suggestions they make in their review. We agree that our work takes the first steps towards a more accurate and informative framework that places prokaryotic comparative genomics on a sounder footing. Following up this work with broader and deeper analyses is something we fully intend to work on in the years to come, in parallel with improving the scalability and performance of our method.

One potential mechanismal problem of the use of MHGs, as I would propose, is that MHGs can span over multiple genes and intergenic regions of the genome, and these nucleotide elements may not follow the same evolutionary model. Current phylogenetic methods, such as maximum likelihood (as the authors used), assume that all sites in an MSA can be modeled using a distribution of mutation rates. If there are obvious heterougenous regions in the MSA, the typical method is to “partition” the MSA and apply separate evolutionary models to individual partitions. In the MHG study, this was apparently not considered, and consideration of partitions will revert MHG back to traditional marker-based protocols. I will be curious to see a comparative analysis of MHGs splitting into gene / intergenic regions and how that impacts phylogenetic inference.

The reviewer here raises an excellent point, which we have explored with additional analysis. This involved repeating the analysis of phylogenetic congruence on two subsets of MHGs; a subset that only overlaps genic sequences, and a subset that only overlaps intergenic sequences. Our initial analysis of congruence was restricted to MHGs longer than 400bp, but only one such intergenic-only MHG exists, so we instead selected MHGs between 100 and 400bp, making 172 intergenic-only MHGs available (and 3485 genic-only MHGs). The species tree inferred from intergenic MHGs has only 11 out of 19 taxa since we had to exclude the endosymbionts and the outgroup *Luteibacter rhizovicinus* (see our next response for more detail on this taxon). The splits inferred from these shorter length MHGs were poorly supported by both gene and intergenic sequences, with the exception of grouping *Escherichia coli* and *Shigelli flexneri*.

The low number and short length of intergenic-only MHGs suggests these did not contribute much signal to the analysis of congruence, and to confirm this we recalculated the MHG species tree with any MHGs containing intergenic sequences excluded. Our expanded analysis with an additional figure is now included at the end of the results section.

One potential disadvantage of this method, as I would imagine, is that it may struggle with a set of taxa with high divergence, because nucleotide-level homology is hard to identify using BLASTn when the evolutionary distance is large. In this work, the authors only tested the method of a family-level taxon set. They mentioned in line 77: “when the divergence is low enough for sequence homology to be identifiable at a nucleotide level…” I will be curious about the performance in more diverse datasets, such as order- or class-level taxon sets. It may or may not be necessary to add new analyses, but some discussion should be favorable.

We thank the reviewer for this suggestion, and thanks to a fortuitous occurrence we can go beyond discussion. In the process of revising the manuscript, we found one of the 19 genome assemblies we included (GCF_001887595.1) was mislabelled by Zhu *et al*. as *Klebsiella michiganensis*. We used the taxonomic assignment of that previous paper but it turns out that the assembly is from *Luteibacter rhizovicinus*, a species from a different order of Gammaproteobacteria. This serendipitous discovery means we can now report the performance of our method across taxonomic orders, and we have added the following to the main text:

“Fewer MHGs contained sequences from L. rhizovicinus, although the proportion of genic sequences covered by MHGs converged with other taxa for MHGs with more genomes included. By contrast, there was almost no intergenic coverage, even for MHGs with 2 or more genomes represented. This is due to the fact that L. rhizovicinus is highly divergent from the other genomes in our analysis, being from an entirely different family within gamma-proteobacteria (Rhodanobacteraceae). Nonetheless, the substantial coverage of genic sequences demonstrates the suitability of using MHGs for interfamily analysis.”

We are currently undertaking a study comparing the performance of methods for inferring homology across multiple genomes at different levels of phylogenetic divergence, and hope this will be of interest to the reviewer once it is finished.

One thing the authors haven’t discussed (only briefly mentioned in the Discussion (line 380) is the scalability of this algorithm. Because it relies on all-vs-all BLAST, I guess that the computational complexity is at least O(n^2). I futher guess that alternatives like Usearch and Minimap can accelerate this process, although may or may not change the scaling factor. The test dataset only has 19 genomes, which is a very small one in modern phylogenomics. The readers may be curious about how many taxa can they include in such an analysis. Even further optimization is not introduced in the current work, I would suggest that the authors at least make some discussion and point to future directions.

Scalability is indeed the factor which limits the application of the current version of the tool, and we have concluded that the time complexity is indeed O(n^2). We have added the following description of time complexity to the manuscript:

“The first stage, all-vs-all BLAST search has a time complexity O(n^2) where n is the number of genomes, since every genome is queried by every genome. In a hypothetical scenario where all genes are present as single-copy orthologs in every genome, the alignment graph to be walked in the second stage will have O(n^2) edges. Since every edge is traversed once, this stage also has a time complexity of O(n^2).

When an edge is visited that leads to an ortholog from a new species being added to an existing MHG, this genome will be segmented at that locus and the ortholog incorporated into the MHG through backtracking. A new R-tree—used to store the segmentation of each genome—will need to be constructed which in the worst case takes O(s log s) time where s is the number of segments, which will be proportional to the number of single-copy orthologs. The backtracking process is of time complexity O(m) where m is the number of sequences in the MHG, which will grow with n. However, as n increases, an increasing proportion of edges will not lead to the incorporation of new sequences, hence R-tree construction and backtracking can be discounted.

For any edge visited, the R-trees of the source and destination genomes are queried. The time complexity of these queries is bounded by O(log s) and O(s), but since this will not depend on the number of genomes in this hypothetical scenario, the overall time complexity relative to the number of genomes is O(n^2). Under more realistic conditions the time complexity becomes much more difficult to analyze as the number of segments in each genome will depend on both the number of genomes and multifarious evolutionary processes.”

We have also added discussion of the current bottleneck and future optimization direction in the revised manuscript:

“The bottleneck of the current MHG tool is the polynomial (under ideal conditions) growth in runtime with the number of genomes. One avenue for alleviating this is replacing the all-vs-all BLAST with a subset of pairwise (genome by genome) BLAST queries, since MHGs are transitive in nature, complete connectivity between genomes is not required. This could use a phylogenetic guide tree, although rigorous study is needed to determine the best approach of choosing the subset of pairwise queries to minimize any loss of sensitivity. As with many algorithms, a divide-and-conquer strategy may also improve walltime performance, although not overall CPU time or power consumption.”

In the simulation study, the accuracy of the phylogenetic trees was evaluated using the following (line 259): “For the non-recombining segments, bootstrap support for true branches was higher than incorrect branches, suggesting that bootstrap support values for inferred MHG trees can be used to identify true splits.” While I can understand its logic, I do not see this as a compelling approach. Higher bootstrap from non-recombining segments is largely anticipated, but whether this can reflect “true” evolutionary history is a totally independent question. Use of semi-real datasets (as in contrast to purely simulated dataset) for validating a phylogenetic approach does have the problem of not being able to identify the grouth truth, and I doubt if the current solution is a complete solution. One thing that would be good to add is to introduce some biology – Enterobacteriaceae is a well-studied taxonomic group, with numerous literatures discussing its classification and evolution. The authors may read some literatures and compare the phylogenetic trees (marker- or MHG-based) with the knowledge base. If they can identify some well-known evolutionary pattern supported by one tree but not the other, it could be interesting and informative to the readers.

We have clarified our statement to make clear that bootstrap support values may be used as a measure of certainty in the local phylogeny rather than the clonal frame, when applied to non-recombining segments. We believe this is an accurate statement because although the initial species tree was derived from the assembled bacterial genomes, the simulation study we performed was a simulation where the ground truth is known for the local phylogenies.

Putting our biological results in the context of existing literature is a very helpful suggestion. The major difference between our marker and MHG phylogenies is within the endosymbiont clade, so we performed a literature search on those endosymbionts, including their taxonomy and host ranges. The following paragraph has been added to the discussion:

“Of particular interest is our findings on the evolution of Enterobacteriaceae endosymbionts. Both marker and MHG phylogenies strongly support the monophyly of this group, in contrast to a previous concatenation analysis which claimed it has multiple origins. These vertically transmitted endosymbionts provide essential amino acids to their insect hosts, and transmission may occur externally after oviposition. Neither marker nor MHG phylogeny is compatible with pure vertical transmission however, with the mealybug endosymbionts *Candidatus* Hoaglandella, Doolittlea and Gullanella being paraphyletic or polyphyletic. Based on the placement of those taxa, we hypothesize that this symbiotic relationship originated in mealybugs before diversifying to other insects. The major difference between phylogenies is that the MHG phylogeny is incompatible with this diversification occurring within a single lineage, as the EQP-IC measure strongly rejects a clade containing the four non-mealybug endosymbionts.“

With regard to the terminology – is “homologous” in MHG actually “orthologous”? Because HGTs and recombinations within the same gene family (i.e., same evolutionary origin, despite old or young) do not break homology; they are just not orthologous which is the assumption for tree building.

MHGs are defined as including homologous sequences, including paralogs. We designed our method to include paralogous as well as orthologous sequenced, and have added the following sentence to the introduction to clarify this point:

“Sequences assigned to each MHG using our method may be orthologous or paralogous, due to hidden paralogy and because we incorporate every sequence identified as homologous from each genome, which necessarily includes duplications.”

Line 35: There are two “even”s.

Corrected

Finally, I suggest that the authors make more description of the software tool they developed. I checked the GitHub repository and found that it has a decent command-line interface and documentation. seemingly ready for end users. If that’s the case, the audience may want to know because they will be prone to give it a try.

This is definitely a helpful suggestion that we hope will grow and benefit our user base. We have updated our Github repository to explain more details about how to use our method, including as part of the pipeline with blastn that is described in our paper. In addition to genomes provided for testing purposes, we also added introductions about how to interpret the output.

Reviewer #2: In the manuscript titled "Annotation-free delineation of prokaryotic homology groups", Yin et al. present a pipeline for annotating sequences to identify maximal homologous group (termed MHGs). These MHGs can be interpreted as non-recombining blocks (or at least no evidence of recombination from the sequences), which is important for estimating gene trees with high fidelity and which has downstream impacts on understanding species relationships at the genome scale. Appropriate non-recombining blocks are particularly difficult to identify for genomes separated by deep timescales, as may be found through comparison of highly divergent prokaryotic taxa.

The manuscript is easy to understand, with clear visualizations describing the authors' proposed pipeline, which makes it accessible to the non-expert computational biologist. The authors also released open-source software on GitHub, ensuring that their method is easily applied by the broader scientific community. Overall, I believe this is a quality study. I have mostly minor concerns that I list below, which relate to certain analysis, presentation, or experimental choices

We thank the reviewer for their careful reading and constructive assessment of our paper.

1. On lines 199-201, the authors should motivate their choices for the BLASTn parameters either by citation or by providing their reasoning.

The intuition behind for choosing BLASTn parameters is definitely an important aspect which directly affects the performance of MHG, we have put the following explanations in the revised manuscript:

“These parameter values were chosen so that our method will work even for very divergent genomes. A shorter word size than the default enables BLASTn to align regions sharing shorter exactly matched k-mers, and choosing the smallest possible mismatch and gap penalties should maximize the lengths of locally homologous alignments identified by BLASTn.”

2. On lines 208-210, for simulation of marker genes, the component gene trees were obtained by performing a nearest-neighbor interchange (NNI) operation on the immediately preceding component tree (or initial tree if first component gene tree). My understanding is that subtree prune and regraft (SPR) is a better model of recombination than is NNI. Why not use SPR here instead of NNI? If SPR would make identification of MHGs more difficult, then I think it would be worth understanding performance under such a setting.

We picked NNI over SPR because it is the more realistic tree operation for simulating recombination, since SPR ignores the relatedness of taxa, and will just as likely recombine segments between very distant and highly related species. NNI on the other hand only modifies the phylogeny by swapping (closely related) “sibling” with “uncle” taxa. We have added a sentence to our methods explaining this reasoning.

We agree that an expanded simulation study of homology group identification and misspecification would be of interest to the broader phylogenomics community. This could include considering different levels of genome divergence and recombination probabilities, whether by using SPR or some other method. While the simulations in our current manuscript are intended to contextualize our empirical findings rather than be a standalone work, we do intend to follow this up with just such a broader study in the future.

3. On lines 211-214, the authors simulated 15 component gene trees that were then used to evolve 100 base sequence alignments to generate an overall sequence of length 1.5 kb. These sequences evolved with equal transition rates and base frequencies. What is the motivation for these particular parameter choices? Are these consistent with the empirical results obtained from the prokaryotic data? That is, are the transition rates and base frequencies close to genome expectations from the empirical data, and are 1.5 kb sequences a reasonable size to understand method performance from the simulated data? Motivation for these parameters or citations should be provided to ensure that the simulated setting is realistic.

We chose 1.5kb because it is close to the average length of marker genes included in our study, which we calculated as being 1503bp. The choice of 100bp was made to ensure phylogenetic histories of each segment were to an extent still resolvable while simulating a substantial number of recombination events within each gene. The choice of a Jukes-Cantor substitution model was made conservatively, since exploring the effects of unequal base frequencies, transition rates etc. is outside the scope of our current study. We now explain these choices in the methods, and thank the reviewer for prompting us to include these (and the NNI move) justifications in our manuscript.

4. On line 232, internal branch lengths less than 0.0005 were treated as unresolved. What is the motivation for this number? Is it based on fewer than one mutation across the 1.5 kb sequence? That is, 1/1500 is approximately 0.00067, which is maybe how 0.0005 was derived?

That is exactly what our intuition behind was, we now explain it in the manuscript as follows:

“This threshold value is smaller than the reciprocal of the average marker gene length of 1,503bp, so branch lengths below this threshold imply no mutations occurred along this branch (at least under a Jukes-Cantor model) and the relationship between the four subtrees adjacent to this branch is impossible to resolve.”

5. On lines 276-280, coverage information is highlighted relating to K. michiganensis being poor. However, from Figure 4, it appears as if roughly half the sampled species in the empirical analysis have similarly poor coverage compared to K. michiganensis. Because of this, I am unsure why K. michiganensis is specifically being highlighted here.

While revising the manuscript, we discovered that this genome actually corresponds to *Luteibacter rhizovicinus*, another species of Gammaproteobacteria but one from a different family to the other 18. See our response to reviewer 1 for more on this discovery. As such we believe its coverage relative to other species is worth highlighting, since it illustrates the performance of our method at more divergent timescales. The sentences the reviewer points out have also been rewritten to make this point.

6. In Figure 4, why is gene length almost the same size as genome length? Is this because the intergenic regions are small, and so the genome is fairly gene dense?

As the reviewer deduced, this is due to the fact that prokaryotes have consistently high gene density. We have added this explanation to the main text with references.

7. In Figure 5, the y-axis should read "Percentage of all genes" rather than proportion. Also, how are the GO categories on the x-axis ordered? Why not order them by over- to under-representation of markers, so it is easier to follow the way at which the results are described in the text for this figure.

We thank the reviewer for both suggestions, which we agree improves Figure 5 and hence we have adopted them.

8. In Figure 8, why not plot cumulative distribution functions (CDFs) instead of estimated density plots derived from smoothing kernels? This approach would encode all the necessary information to compare the distributions, without having to smooth the data with a kernel density estimator. That is, we will see the actual data rather than smoothed data.

As with figure 5, we agree this change is an improvement and hence we have adopted it. We have rewritten the figure caption to reflect the new distribution.

Reviewer #3: This paper is a scholarly attempt to address the problem of phylogenetic inference in the case where different genes and gene fragments have different evolutionary histories, due to a variety of mechanisms by which DNA can be transmitted non vertically. It shows in particular that marker gene methods are quite biased and can easily end up including genes with conflicted signals that can bias the results. The paper is honest about its limitations, which I take to be a lack of scaleability.

We thank the reviewer for their concise and informative summary of our paper. Indeed, improving scalability is an immediate goal for us in building upon the current method.

My first main comment is that the paper shows how the method works in principle to identify genome alignments, but does little to show us what this looks like in practice. I.e. what do MHG blocks look like in practice for the real data as opposed to the conceptual data shown in figure 1. This may be difficult to look at for the full 19 species dataset, in which case a smaller one could be used for this purpose.

Thank the reviewer for suggesting the visualization of MHGs in real data. In the revised manuscript, we provide supplementary information which shows graphically the overlap of MHGs with each identified marker gene. This should provide readers with an intuitive understanding of how the output of the method looks in practice. We reference this material in the main text as part of our improved explanation of the bootstrap support based comparison of marker gene and MHG splits (which we revised in response to the reviewer’s second main comment).

My second main comment is that the section "correspondence between markers and MHGs" and the two main text figures associated with it (figures 6 and 7) is hard to understand. Full concordance of genes seems perhaps unrealistic in a dataset with 19 taxa, while large parts of the tree are likely to be concordant between every methods. So it is easy to quantify concordance in a way that is either over-sensitive to small differences or is insensitive to fairly substantial ones. I find this section very technical and do not have too much orientation about what individual datapoints mean. Maybe there is a way of choosing a simplified example, either with less taxa, and explaining the metrics in more concrete terms to make it clearer what is going on.

We thank the reviewer for pointing out how our original explanation did not convey our full understanding about this conflict. We have revised this section to more clearly explain why the joint distribution of bootstrap support goes beyond a small amount of conflict between markers and MHGs.
